# Supplementary material for: In vitro and ex vivo proteomics of Mycobacterium marinum biofilms and the development of biofilm-binding synthetic nanobodies
Source: mSystems. 2023 May 15;8(3):e01073-22. doi: 10.1128/msystems.01073-22 (PMC10308901; doi:10.1128/msystems.01073-22)
Supplement: Table S1 — All identified proteins captured by streptavidin from the biotinylated and non-biotinylated intact and lysed biofilms. Secretory/subcellular location for each protein was predicted using EMBOSS Pepstats (pI, Mw, protein length), SignalP 6.0 (Moonlighters, T7SS, TAT, SecI-III), TMHMM (no. transmembrane domains). Protein sequences for each protein were retrieved from UniProt (https://www.uniprot.org/uploadlists/) with the aid of the UniProt IDs. IDs regarding GroEL1 and GroEL2 are indicated with bold letters and shaded in red. Cells in raw intensity value-associated columns shaded in grey indicate proteins that were not detected or were detected in only one of the replicate samples. [file msystems.01073-22-s0001.pdf]

TABLE S1. All identified proteins captured by streptavidin from the biotinylated and non-biotinylated intact and lysed biofilms. Secretory/subcellular location for each protein was predicted using EMBOSS Pepstats (pI, Mw, protein length), SignalP6.0 (Moonlighters, T7SS, TAT, SecI-III), TMHMM (no. transmembrane domains). Protein sequences for each protein were retrieved from UniProt (https://www.uniprot.org/uploadlists/) with the aid of the UniProt IDs. IDs regarding GroEL1 and GroEL2 are indicated with bold letters and shaded in red. Cells in raw intensity value-associated columns shaded in grey indicate proteins that were not detected or where detected in only one of the replicate samples.

ID	Protein name	pI	MW k(Da)	Seq. Length	TMHMM No. TMDs	SignalP6.0 NO_SP=not secreted SP=secreted out	OTHER Moonlighters=light green T7SS=dark green	Sec/SPI (light green)	LIPO Sec/SPII (light green)	Tat/SPI (light green)	TAT-LIPO Sec/SPII (light green)	No. proteins	Razor + uniq. Pept.	Biot-Intact-Biofilm<br>Avr. Raw int values	Non-Biot-Intact-Biofilm<br>Avr. Raw int values	Fold-change<br>Biot-vs-non-Biot	Fold-change<br>Non-Biot-vs-Biot
**A0A117DW44**	**60 kDa GroEL2 chaperonin**	**4,6**	**56,5**	541	0	NO_SP	1.000089	0.000000	0.000000	0.000000	0.000000	12	43	**159493333,3**	**1728300**	**92,28**	**0,01**
**B2HDZ3**	**Biotin carboxyl carrier protein**	**5,1**	**63,7**	597	0	NO_SP	1.000064	0.000002	0.000000	0.000000	0.000000	4	35	**116845333,3**	**80507667**	**1,45**	**0,69**
B2HSL3	Elongation factor Tu	5,0	**43,8**	396	0	NO_SP	1.000057	0.000000	0.000000	0.000000	0.000000	4	26	**116596666,7**	**1558667**	**74,81**	**0,01**
A0A2Z5YJX5	ATP synthase subunit alpha	4,9	**59,4**	549	0	NO_SP	0.999904	0.000124	0.000006	0.000000	0.000000	4	39	**76866000**	**1999400**	**38,44**	**0,03**
A0A2Z5YCS7	Alanine dehydrogenase	6,1	**38,6**	371	0	NO_SP	1.000024	0.000004	0.000000	0.000000	0.000000	4	22	**76459333,33**	**1093530**	**69,92**	**0,01**
**B2HD10**	**60 kDa GroEL1 chaperonin**	**4,5**	**55,9**	539	0	NO_SP	1.000080	0.000000	0.000000	0.000000	0.000000	2	33	**66767666,67**	**646350**	**103,30**	**0,01**
A0A2Z5YD42	Multifunctional mycocerosic acid synthase	4,7	**222,9**	2099	0	NO_SP	0.683434	0.006676	0.309597	0.000050	0.000037	4	84	**65638000**	**2817967**	**23,29**	**0,04**
B2HSJ4	DNA-directed RNA polymerase subunit beta	6,2	**146,8**	1316	0	NO_SP	1.000047	0.000000	0.000000	0.000000	0.000000	5	75	**59059000**	**2478367**	**23,83**	**0,04**
B2HJ41	ATP-dependent protease ATP-binding subunit ClpC1	5,6	**93,7**	848	0	NO_SP	1.000074	0.000001	0.000000	0.000000	0.000000	4	50	**58427000**	**786107**	**74,32**	**0,01**
B2HQK2	ATP synthase subunit beta	4,6	**52,7**	483	0	NO_SP	1.000036	0.000000	0.000000	0.000000	0.000000	4	30	**57232666,67**	**1979533**	**28,91**	**0,03**
B2HSL2	Elongation factor G	4,7	**77,0**	701	0	NO_SP	1.000083	0.000000	0.000000	0.000000	0.000000	3	40	**41424000**	**1028703**	**40,27**	**0,02**
A0A2Z5YAJ7	DNA-directed RNA polymerase subunit alpha	4,4	**37,7**	347	0	NO_SP	0.999041	0.000999	0.000008	0.000002	0.000001	3	28	**37300333,33**	**586083**	**63,64**	**0,02**
A0A3E2N0M2	Dihydrolipoyl dehydrogenase	6,0	**49,4**	464	0	NO_SP	1.000076	0.000001	0.000000	0.000000	0.000000	4	30	**37075666,67**	**142405**	**260,35**	**0,00**
B2HD57	L-lactate dehydrogenase (Cytochrome) LldD2	8,7	**45,6**	414	0	NO_SP	1.000041	0.000000	0.000000	0.000000	0.000000	4	24	**36264000**	**3012400**	**12,04**	**0,08**
A0A2Z5Y928	Chaperone protein DnaK	4,4	**66,4**	622	0	NO_SP	1.000058	0.000000	0.000000	0.000000	0.000000	4	45	**34613333,33**	**141908**	**243,91**	**0,00**
A0A2Z5YDP2	Glyceraldehyde-3-phosphate dehydrogenase	5,2	**36,9**	346	0	NO_SP	1.000048	0.000000	0.000000	0.000000	0.000000	3	14	**34552333,33**	**527030**	**65,56**	**0,02**
A0A2Z5YKE5	Type I polyketide synthase	4,7	**326,1**	3072	0	NO_SP	1.000073	0.000001	0.000000	0.000000	0.000000	4	102	**34228000**	**1493533**	**22,92**	**0,04**
B2HIH0	D-3-phosphoglycerate dehydrogenase	4,6	**54,2**	528	0	NO_SP	1.000049	0.000008	0.000000	0.000000	0.000000	4	26	**32785333,33**	**618240**	**53,03**	**0,02**
B2HED1	Succinate--CoA ligase [ADP-forming] subunit beta	4,5	**40,8**	387	0	NO_SP	1.000079	0.000000	0.000000	0.000000	0.000000	4	20	**32629333,33**	**485807**	**67,17**	**0,01**
A0A2Z5YJR6	Malate dehydrogenase	4,6	**34,6**	329	0	NO_SP	1.000059	0.000000	0.000000	0.000000	0.000000	3	20	**32545000**	**831667**	**39,13**	**0,03**
B2HN30	Acyl-CoA dehydrogenase FadE5	4,8	**66,4**	611	0	NO_SP	1.000048	0.000000	0.000000	0.000000	0.000000	4	29	**32233333,33**	**943787**	**34,15**	**0,03**
B2HP50	S-adenosylmethionine synthase	4,8	**43,1**	403	0	NO_SP	1.000039	0.000001	0.000000	0.000000	0.000000	4	28	**32228000**	**455310**	**70,78**	**0,01**
A0A2Z5Y8M9	Phosphoenolpyruvate carboxykinase [GTP]	4,5	**67,8**	609	0	NO_SP	1.000021	0.000000	0.000000	0.000000	0.000000	4	30	**32101000**	**698923**	**45,93**	**0,02**
A0A2Z5YDT4	ATPase	6,0	**40,0**	371	0	NO_SP	1.000026	0.000000	0.000000	0.000000	0.000000	3	18	**31917333,33**	**623190**	**51,22**	**0,02**
A0A2Z5YPA6	ESX-1 secretion-associated protein EspF	5,6	**10,6**	103	0	NO_SP	1.000035	0.000001	0.000000	0.000000	0.000000	4	8	**31057000**	**1162967**	**26,70**	**0,04**
A0A2Z5YDP5	Polyketide synthase	4,6	**222,6**	2090	0	NO_SP	0.878957	0.003429	0.117426	0.000018	0.000011	3	70	**31051000**	**692623**	**44,83**	**0,02**
A0A2Z5YMM5	3-hydroxyacyl-CoA dehydrogenase	5,0	**75,7**	714	0	NO_SP	1.000064	0.000000	0.000000	0.000000	0.000000	4	41	**30534000**	**511807**	**59,66**	**0,02**
B2HSJ3	DNA-directed RNA polymerase subunit beta	4,7	**129,5**	1176	0	NO_SP	1.000047	0.000001	0.000000	0.000000	0.000000	1	67	**29346666,67**	**753913**	**38,93**	**0,03**
B2HD60	Alkyl hydroperoxide reductase C	4,3	**21,5**	195	0	NO_SP	1.000015	0.000005	0.000000	0.000000	0.000000	6	15	**29089000**	**692923**	**41,98**	**0,02**
B2HIL7	Phenolphthiocerol synthesis polyketide synthase type I Pks15/1	4,5	**217,7**	2104	0	NO_SP	1.000030	0.000003	0.000000	0.000000	0.000000	4	47	**28928333,33**	**973297**	**29,72**	**0,03**
A0A2Z5YE47	(2R,3S)-2-methylisocitrate dehydratase	4,6	**101,8**	939	0	NO_SP	1.000052	0.000000	0.000000	0.000000	0.000000	4	32	**28779000**	**861077**	**33,42**	**0,03**
B2HQZ7	Ribosomal protein S1 RpsA	4,5	**53,1**	481	0	NO_SP	1.000072	0.000000	0.000000	0.000000	0.000000	4	32	**27619000**	**256067**	**107,86**	**0,01**
A0A2Z5YP37	Polyketide synthase	4,6	**191,1**	1782	0	NO_SP	1.000068	0.000000	0.000000	0.000000	0.000000	4	65	**26325000**	**1600307**	**16,45**	**0,06**
B2HGY4	Dihydrolipoamide acetyltransferase component of pyruvate dehydrogenase c	4,4	**60,7**	588	0	NO_SP	1.000044	0.000000	0.000000	0.000000	0.000000	4	22	**26059000**	**441963**	**58,96**	**0,02**
A0A2Z5YFA5	Malate synthase G	4,7	**79,0**	727	0	NO_SP	1.000044	0.000000	0.000000	0.000000	0.000000	9	31	**25285666,67**	**607090**	**41,65**	**0,02**
A0A2Z5YCX7	Polyribonucleotide nucleotidyltransferase	4,5	**80,6**	762	0	NO_SP	1.000049	0.000003	0.000000	0.000000	0.000000	4	36	**24891333,33**	**436187**	**57,07**	**0,02**
A0A2Z5YC93	Electron transfer flavoprotein subunit alpha	4,4	**31,7**	318	0	NO_SP	1.000039	0.000000	0.000000	0.000000	0.000000	3	14	**24622000**	**302183**	**81,48**	**0,01**
A0A2Z5YFU6	Catalase-peroxidase	4,8	**77,5**	708	0	NO_SP	0.999907	0.000115	0.000010	0.000000	0.000000	4	28	**24283333,33**	**706997**	**34,35**	**0,03**
B2HDS7	O-acetylhomoserine sulfhydrylase MetC	5,2	**47,4**	449	0	NO_SP	1.000006	0.000022	0.000001	0.000000	0.000000	4	20	**22993666,67**	**219530**	**104,74**	**0,01**
B2HHR6	3-oxoacyl-[acyl-carrier protein] synthase 1 KasA	4,9	**43,7**	416	0	NO_SP	1.000042	0.000001	0.000000	0.000000	0.000000	4	20	**22912666,67**	**188286**	**121,69**	**0,01**
B2HGQ8	Antigen 84	4,4	**28,7**	264	0	NO_SP	1.000059	0.000001	0.000000	0.000000	0.000000	4	15	**22882666,67**	**350540**	**65,28**	**0,02**
A0A2Z5YAR9	30S ribosomal protein S3	10,7	**30,2**	275	0	NO_SP	1.000059	0.000001	0.000000	0.000000	0.000000	3	14	**22809666,67**	**1851433**	**12,32**	**0,08**
B2HEP6	Adenosylhomocysteinase	4,8	**53,9**	492	0	NO_SP	1.000045	0.000002	0.000000	0.000000	0.000000	3	25	**22771000**	**809813**	**28,12**	**0,04**
A0A2Z5YK73	Transcription termination factor Rho	5,9	**66,9**	621	0	NO_SP	0.999853	0.000166	0.000011	0.000001	0.000000	3	41	**22394666,67**	**236920**	**94,52**	**0,01**
A0A100I851	Succinate--CoA ligase [ADP-forming] subunit alpha	6,1	**30,8**	300	0	NO_SP	1.000064	0.000000	0.000000	0.000000	0.000000	3	20	**22251000**	**316690**	**70,26**	**0,01**
B2HIK4	Pyruvate carboxylase	5,3	**121,0**	1131	0	NO_SP	1.000063	0.000000	0.000000	0.000000	0.000000	4	51	**21684000**	**6494400**	**3,34**	**0,30**
A0A2Z5YPN8	Long-chain-fatty-acid--AMP ligase FadD32	6,2	**68,9**	629	0	NO_SP	1.000092	0.000000	0.000000	0.000000	0.000000	4	24	**20771333,33**	**473680**	**43,85**	**0,02**
B2HT60	Acyl-[acyl-carrier protein] desaturase DesA2	4,5	**31,3**	275	0	NO_SP	1.000033	0.000000	0.000000	0.000000	0.000000	4	19	**20393666,67**	**939830**	**21,70**	**0,05**
A0A2Z5YFQ2	Isocitrase	5,4	**85,2**	762	0	NO_SP	1.000038	0.000000	0.000000	0.000000	0.000000	4	40	**19676000**	**1044943**	**18,83**	**0,05**
B2HEK7	Propionyl-CoA carboxylase beta chain 5 AccD5	4,8	**58,9**	546	0	NO_SP	1.000058	0.000001	0.000000	0.000000	0.000000	4	21	**19277666,67**	**533020**	**36,17**	**0,03**
B2HLS8	Short-chain type dehydrogenase/reductase	5,1	**29,8**	286	0	NO_SP	1.000042	0.000002	0.000000	0.000000	0.000000	4	13	**19170333,33**	**548707**	**34,94**	**0,03**
A0A2Z5Y8J9	Dihydroxy-acid dehydratase	5,0	**58,6**	564	0	NO_SP	1.000059	0.000000	0.000000	0.000000	0.000000	4	22	**18215000**	**4463633**	**4,08**	**0,25**
A0A2Z5YA35	50S ribosomal protein L7/L12	4,3	**13,4**	130	0	NO_SP	1.000067	0.000000	0.000000	0.000000	0.000000	4	11	**18193333,33**	**449873**	**40,44**	**0,02**
B2HF81	Citrate synthase	5,4	**48,2**	431	0	NO_SP	1.000028	0.000011	0.000000	0.000000	0.000000	4	21	**18156000**	**672790**	**26,99**	**0,04**
A0A100I7X3	PPE family protein	4,9	**14,3**	147	0	NO_SP	1.000006	0.000027	0.000003	0.000000	0.000000	7	1	**17942000**	**11468200**	**1,56**	**0,64**
B2HGH4	Acyl-CoA dehydrogenase FadE23	5,1	**43,5**	401	0	NO_SP	1.000069	0.000000	0.000000	0.000000	0.000000	3	15	**17756166,67**	**7300333**	**2,43**	**0,41**
B2HCX3	30S ribosomal protein S4	10,4	**23,4**	201	0	NO_SP	1.000034	0.000000	0.000000	0.000000	0.000000	4	14	**17518333,33**	**2309700**	**7,58**	**0,13**
A0A2Z5YBU2	Protein translocase subunit SecA	4,8	**104,9**	938	0	NO_SP	1.000042	0.000000	0.000000	0.000000	0.000000	5	42	**17213000**	**265160**	**64,92**	**0,02**
B2HJN3	Elongation factor Ts	4,8	**28,5**	271	0	NO_SP	1.000041	0.000003	0.000000	0.000000	0.000000	3	19	**16864000**	**368383**	**45,78**	**0,02**
A0A2Z5YHF6	Glutamine synthetase	4,7	**53,5**	478	0	NO_SP	1.000051	0.000000	0.000000	0.000000	0.000000	3	23	**16708333,33**	**386307**	**43,25**	**0,02**
B2HCT8	30S ribosomal protein S5	10,6	**22,9**	224	0	NO_SP	0.999616	0.000353	0.000027	0.000001	0.000001	3	11	**16707533,33**	**700593**	**23,85**	**0,04**
A0A2Z5YK62	Multifunctional fusion protein	4,8	**47,9**	445	1	NO_SP	1.000050	0.000000	0.000000	0.000000	0.000000	4	25	**16630000**	**723820**	**22,98**	**0,04**
A0A2Z5YLZ1	TIGR04255 family protein	4,8	**29,7**	270	0	NO_SP	1.000067	0.000000	0.000000	0.000000	0.000000	4	13	**16348000**	**715687**	**22,84**	**0,04**
A0A2Z5YJV4	Alanine aminopeptidase	4,6	**95,1**	871	0	NO_SP	1.000048	0.000003	0.000000	0.000000	0.000000	4	34	**16281666,67**	**585450**	**27,81**	**0,04**
A0A3E2N1P5	2-hydroxy-3-oxoadipate synthase	6,3	**137,4**	1245	0	NO_SP	1.000062	0.000000	0.000000	0.000000	0.000000	4	44	**16241000**	**604633**	**26,86**	**0,04**

A0A2Z5YHF0	Acyl carrier protein	3,8	**12,5**	115	0	NO_SP	1.000052	0.000001	0.000000	0.000000	0.000000	3	8	**16148000**	**516117**	**31,29**	**0,03**
A0A2Z5YPB5	ESAT-6-like protein	4,4	**10,0**	95	0	NO_SP	1.000054	0.000000	0.000000	0.000000	0.000000	8	5	**15886666,67**	**140700**	**112,91**	**0,01**
A0A117DTM1	50S ribosomal protein L5	10,6	**20,6**	183	0	NO_SP	1.000058	0.000000	0.000000	0.000000	0.000000	3	7	**15833400**	**7973067**	**1,99**	**0,50**
A0A2Z5YLT1	Acetyl-CoA acetyltransferase	4,6	**40,0**	381	0	NO_SP	1.000045	0.000000	0.000000	0.000000	0.000000	3	18	**15791000**	**464643**	**33,99**	**0,03**
A0A2Z5YH83	3-oxoacyl-[acyl-carrier-protein] synthase 2	5,8	**44,2**	417	0	NO_SP	0.998399	0.001623	0.000017	0.000003	0.000001	3	20	**15749666,67**	**150453**	**104,68**	**0,01**
A0A2Z5Y9S2	Mycolic acid methyltransferase MmaA1	4,6	**33,0**	286	0	NO_SP	1.000064	0.000004	0.000000	0.000000	0.000000	3	15	**15512266,67**	**643817**	**24,09**	**0,04**
A0A2Z5YI10	Chaperone protein HtpG	4,5	**72,8**	648	0	NO_SP	1.000054	0.000000	0.000000	0.000000	0.000000	4	35	**15413033,33**	**426313**	**36,15**	**0,03**
A0A2Z5YA71	Putative ABC transporter ATP-binding protein	5,0	**35,9**	328	0	NO_SP	1.000040	0.000000	0.000000	0.000000	0.000000	3	14	**15138033,33**	**448420**	**33,76**	**0,03**
A0A2Z5YA32	Light-repressed protein A	7,6	**29,6**	267	0	NO_SP	1.000011	0.000043	0.000003	0.000000	0.000000	3	12	**15100100**	**159940**	**94,41**	**0,01**
B2HDI5	Enolase	4,3	**44,8**	428	0	NO_SP	1.000041	0.000001	0.000000	0.000000	0.000000	3	15	**14715000**	**122895**	**119,74**	**0,01**
A0A2Z5Y7T8	Isocitrate dehydrogenase [NADP]	5,0	**82,4**	745	0	NO_SP	1.000063	0.000000	0.000000	0.000000	0.000000	3	32	**14483266,67**	**397957**	**36,39**	**0,03**
A0A117DVM5	ATP-dependent Clp protease proteolytic subunit	4,6	**21,2**	195	0	NO_SP	1.000053	0.000000	0.000000	0.000000	0.000000	3	7	**14241800**	**340370**	**41,84**	**0,02**
B2HIM7	Phenolpthiocerol synthesis type-I polyketide synthase PpsE	4,9	**158,0**	1481	0	NO_SP	1.000023	0.000020	0.000007	0.000000	0.000000	3	46	**14144600**	**510250**	**27,72**	**0,04**
B2HGW3	Cytochrome bc1 complex Rieske iron-sulfur subunit	6,5	**43,1**	394	3	NO_SP	0.999557	0.000333	0.000073	0.000002	0.000001	4	19	**14084533,33**	**527410**	**26,71**	**0,04**
A0A2Z5YHL1	Long-chain-fatty-acid--CoA ligase	5,6	**64,6**	600	0	NO_SP	0.999984	0.000061	0.000002	0.000000	0.000000	7	29	**13970333,33**	**520923**	**26,82**	**0,04**
A0A2Z5Y912	(Fe-S)-binding protein	6,7	**102,8**	970	3	NO_SP	0.998135	0.001708	0.000079	0.000001	0.000000	4	26	**13890366,67**	**334520**	**41,52**	**0,02**
A0A2Z5YEA0	Long-chain-fatty-acid--AMP ligase FadD28	5,1	**63,1**	585	0	NO_SP	1.000054	0.000001	0.000000	0.000000	0.000000	2	30	**13868333,33**	**218403**	**63,50**	**0,02**
A0A2Z5YIL7	Glutamate dehydrogenase	5,1	**176,5**	1612	0	NO_SP	1.000057	0.000001	0.000000	0.000000	0.000000	4	51	**13573666,67**	**572030**	**23,73**	**0,04**
B2HSI8	50S ribosomal protein L10	5,0	**21,1**	206	0	NO_SP	1.000062	0.000000	0.000000	0.000000	0.000000	4	12	**13242400**	**235713**	**56,18**	**0,02**
B2HN36	Succinate dehydrogenase (Iron-sulfur subunit), SdhA_1	5,8	**70,5**	642	0	NO_SP	1.000052	0.000002	0.000000	0.000000	0.000000	4	26	**13036200**	**144863**	**89,99**	**0,01**
A0A2Z5YC88	Ketol-acid reductoisomerase (NADP(+))	4,7	**37,0**	343	0	NO_SP	1.000060	0.000000	0.000000	0.000000	0.000000	3	16	**12953400**	**147743**	**87,68**	**0,01**
A0A2Z5YMV3	ATP-dependent zinc metalloprotease FtsH	5,4	**79,9**	741	2	NO_SP	0.960544	0.018415	0.020608	0.000036	0.000028	4	32	**12781000**	**251507**	**50,82**	**0,02**
A0A124BWX6	Acyl-CoA dehydrogenase	5,5	**64,4**	573	0	NO_SP	0.999846	0.000174	0.000006	0.000001	0.000000	3	26	**12727833,33**	**265023**	**48,03**	**0,02**
A0A2Z5Y7E5	DNA gyrase subunit B	6,4	**76,1**	692	0	NO_SP	1.000049	0.000000	0.000000	0.000000	0.000000	7	26	**12633433,33**	**1967867**	**6,42**	**0,16**
A0A117DTU2	Ferritin	4,6	**21,5**	191	0	NO_SP	1.000040	0.000001	0.000000	0.000000	0.000000	3	8	**12561833,33**	**84383**	**148,87**	**0,01**
B2HSZ6	NADH dehydrogenase Ndh	9,2	**49,7**	461	1	NO_SP	1.000056	0.000002	0.000000	0.000000	0.000000	3	15	**12551466,67**	**347573**	**36,11**	**0,03**
A0A2Z5YEF1	Uncharacterized protein	10,8	**11,5**	105	0	NO_SP	1.000054	0.000000	0.000000	0.000000	0.000000	3	11	**12521166,67**	**2009120**	**6,23**	**0,16**
A0A2Z5YCP0	30S ribosomal protein S2	6,7	**30,0**	276	0	NO_SP	1.000066	0.000000	0.000000	0.000000	0.000000	3	17	**12510466,67**	**645960**	**19,37**	**0,05**
A0A3E2MRS7	Glutamate synthase [NADPH] large chain	5,8	**165,8**	1527	0	NO_SP	1.000058	0.000000	0.000000	0.000000	0.000000	4	44	**12353633,33**	**489803**	**25,22**	**0,04**
B2HEL3	Acyl-CoA dehydrogenase FadE25	4,9	**41,5**	389	0	NO_SP	1.000046	0.000000	0.000000	0.000000	0.000000	5	15	**12075700**	**286647**	**42,13**	**0,02**
B2HCW1	3-hydroxyisobutyrate dehydrogenase	5,2	**29,4**	294	0	NO_SP	1.000023	0.000006	0.000000	0.000000	0.000000	4	7	**12053666,67**	**8793433**	**1,37**	**0,73**
A0A2Z5Y8S3	3-oxoacyl-ACP reductase	6,1	**46,8**	454	0	NO_SP	1.000041	0.000000	0.000000	0.000000	0.000000	4	26	**11927900**	**202630**	**58,87**	**0,02**
B2HCT5	30S ribosomal protein S8	10,5	**14,4**	132	0	NO_SP	1.000062	0.000000	0.000000	0.000000	0.000000	4	8	**11792533,33**	**532760**	**22,13**	**0,05**
A0A2Z5YN34	Crp/Fnr family transcriptional regulator	10,5	**24,7**	224	0	NO_SP	1.000070	0.000000	0.000000	0.000000	0.000000	1	8	**11569300**	**441897**	**26,18**	**0,04**
A0A2Z5YE17	Transketolase	4,6	**75,2**	700	0	NO_SP	1.000031	0.000001	0.000000	0.000000	0.000000	4	25	**11210333,33**	**279277**	**40,14**	**0,02**
A0A2Z5YAJ1	Inosine-5-monophosphate dehydrogenase	6,4	**55,4**	532	0	NO_SP	1.000063	0.000002	0.000000	0.000000	0.000000	4	22	**10895533,33**	**63128**	**172,60**	**0,01**
A0A2Z5YIH3	Glycine--tRNA ligase	5,2	**52,3**	459	0	NO_SP	1.000067	0.000002	0.000000	0.000000	0.000000	4	22	**10857600**	**168617**	**64,39**	**0,02**
A0A2Z5YC17	Putative oxidoreductase	4,5	**29,8**	281	0	NO_SP	1.000052	0.000008	0.000000	0.000000	0.000000	4	10	**10807800**	**158702**	**68,10**	**0,01**
B2HI51	DNA gyrase subunit A	5,3	**92,8**	839	0	NO_SP	1.000081	0.000000	0.000000	0.000000	0.000000	5	35	**10462166,67**	**226510**	**46,19**	**0,02**
A0A2Z5YJ61	Energy-dependent translational throttle protein EttA	4,9	**61,9**	558	0	NO_SP	1.000042	0.000000	0.000000	0.000000	0.000000	4	23	**10446633,33**	**281053**	**37,17**	**0,03**
B2HNG4	ATP-dependent Clp protease proteolytic subunit	4,8	**23,3**	211	0	NO_SP	1.000047	0.000000	0.000000	0.000000	0.000000	3	9	**10247866,67**	**77201**	**132,74**	**0,01**
A0A2Z5YA28	Hydroxymycolate synthase MmaA4	5,0	**34,4**	299	0	NO_SP	1.000060	0.000000	0.000000	0.000000	0.000000	4	17	**10095766,67**	**301150**	**33,52**	**0,03**
A0A2Z5YHM6	Phosphoenolpyruvate synthase	4,7	**88,6**	806	0	NO_SP	1.000064	0.000000	0.000000	0.000000	0.000000	4	29	**10026266,67**	**235453**	**42,58**	**0,02**
A0A2Z5YCB0	Phthiocerol synthesis polyketide synthase type I PpsC	4,5	**232,4**	2201	0	NO_SP	0.999811	0.000189	0.000029	0.000001	0.000000	4	55	**9962133,333**	**334050**	**29,82**	**0,03**
A0A2Z5YE10	Enoyl-[acyl-carrier-protein] reductase [NADH]	5,3	**28,7**	269	0	NO_SP	1.000078	0.000000	0.000000	0.000000	0.000000	3	11	**9694366,667**	**320643**	**30,23**	**0,03**
A0A2Z5YDI5	Acyl-CoA dehydrogenase	4,7	**65,8**	609	0	NO_SP	1.000068	0.000004	0.000000	0.000000	0.000000	4	21	**9673800**	**174757**	**55,36**	**0,02**
B2HLZ7	RNA polymerase sigma factor SigA	4,3	**54,7**	501	0	NO_SP	1.000051	0.000000	0.000000	0.000000	0.000000	3	21	**9650433,333**	**167637**	**57,57**	**0,02**
B2HMJ8	Propionyl-CoA carboxylase beta chain 4 AccD4_1	5,2	**56,9**	526	0	NO_SP	1.000053	0.000000	0.000000	0.000000	0.000000	4	24	**9645366,667**	**166727**	**57,85**	**0,02**
B2HT54	Fumarate hydratase class II	4,7	**50,2**	476	0	NO_SP	1.000024	0.000011	0.000001	0.000000	0.000000	3	12	**9643733,333**	**294483**	**32,75**	**0,03**
B2HDU3	Succinate dehydrogenase flavoprotein subunit	6,2	**64,3**	584	0	NO_SP	1.000024	0.000012	0.000001	0.000000	0.000000	4	22	**9584166,667**	**219957**	**43,57**	**0,02**
B2HQT2	Isocitrase	5,6	**47,3**	428	0	NO_SP	1.000081	0.000000	0.000000	0.000000	0.000000	3	19	**9575266,667**	**159533**	**60,02**	**0,02**
A0A3E2MSW2	Putative acyltransferase	4,6	**48,0**	456	0	NO_SP	1.000071	0.000000	0.000000	0.000000	0.000000	4	19	**9564266,667**	**110300**	**86,71**	**0,01**
A0A2Z5YCW3	Phthiocerol/phenolphthiocerol synthesis polyketide synthase type I PpsD	5,1	**191,7**	1806	0	NO_SP	1.000012	0.000022	0.000000	0.000000	0.000000	4	52	**9533733,333**	**253873**	**37,55**	**0,03**
A0A3E2MQB8	Phosphoglycerate kinase	4,7	**43,0**	416	0	NO_SP	1.000049	0.000000	0.000000	0.000000	0.000000	4	18	**9465066,667**	**153869**	**61,51**	**0,02**
B2HCV1	DNA topoisomerase (ATP-hydrolyzing)	4,6	**23,7**	221	0	NO_SP	1.000031	0.000002	0.000000	0.000000	0.000000	4	15	**9455000**	**111947**	**84,46**	**0,01**
A0A2Z5YCV1	Long-chain-fatty-acid--AMP ligase FadD28	4,8	**62,4**	580	0	NO_SP	1.000041	0.000001	0.000000	0.000000	0.000000	4	20	**9149533,333**	**870110**	**10,52**	**0,10**
A0A100HZX3	50S ribosomal protein L14	10,7	**13,3**	122	0	NO_SP	1.000072	0.000000	0.000000	0.000000	0.000000	3	5	**9142033,333**	**386677**	**23,64**	**0,04**
B2HMA0	Acyl-[acyl-carrier protein] desaturase DesA1_1	5,8	**39,2**	338	0	NO_SP	1.000060	0.000000	0.000000	0.000000	0.000000	2	18	**9105100**	**120334**	**75,67**	**0,01**
A0A2Z5YED3	Cyanophycin synthase	4,7	**97,5**	911	0	NO_SP	1.000037	0.000000	0.000000	0.000000	0.000000	3	42	**9096833,333**	**231557**	**39,29**	**0,03**
A0A2Z5YHD7	Pyruvate dehydrogenase E1 component	6,1	**103,4**	929	0	NO_SP	1.000036	0.000004	0.000000	0.000000	0.000000	1	28	**9086866,667**	**49826**	**182,37**	**0,01**
B2HGH5	Acyl-CoA dehydrogenase FadE24	5,4	**49,8**	465	0	NO_SP	1.000016	0.000042	0.000000	0.000000	0.000000	4	13	**9022133,333**	**119670**	**75,39**	**0,01**
A0A100I9T1	Inositol-3-phosphate synthase	5,0	**40,4**	372	0	NO_SP	0.999947	0.000121	0.000000	0.000000	0.000000	3	21	**8952833,333**	**96608**	**92,67**	**0,01**
A0A100I2P8	Enoyl-CoA hydratase EchA6	5,4	**26,2**	243	0	NO_SP	1.000061	0.000005	0.000000	0.000000	0.000000	3	13	**8917666,667**	**207517**	**42,97**	**0,02**
B2HHR8	Acetyl/propionyl-CoA carboxylase (Beta subunit) AccD6	6,1	**50,1**	473	0	NO_SP	0.999946	0.000090	0.000001	0.000000	0.000000	4	23	**8907266,667**	**268413**	**33,18**	**0,03**
A0A3E2MQH1	Carbamoyl-phosphate synthase large chain	4,6	**119,7**	1121	0	NO_SP	0.968585	0.030538	0.000699	0.000074	0.000048	5	33	**8906333,333**	**153237**	**58,12**	**0,02**
A0A2Z5YKW6	Aldehyde-alcohol dehydrogenase	4,9	**95,1**	889	0	NO_SP	0.998912	0.001111	0.000006	0.000002	0.000001	6	31	**8881933,333**	**99370**	**89,38**	**0,01**
A0A2Z5YJL0	ATP synthase gamma chain	5,0	**33,7**	304	0	NO_SP	1.000070	0.000000	0.000000	0.000000	0.000000	4	19	**8784500**	**263413**	**33,35**	**0,03**
A0A2Z5YAZ9	Acid phosphatase	6,2	**29,1**	275	0	NO_SP	0.999062	0.000970	0.000009	0.000002	0.000001	4	10	**8745600**	**227537**	**38,44**	**0,03**
B2HSL1	30S ribosomal protein S7	11,1	**17,6**	156	0	NO_SP	1.000059	0.000000	0.000000	0.000000	0.000000	3	9	**8742900**	**2296567**	**3,81**	**0,26**
A0A2Z5YES1	Acyl-CoA synthetase	5,6	**107,2**	994	0	NO_SP	1.000040	0.000000	0.000000	0.000000	0.000000	4	31	**8662366,667**	**215103**	**40,27**	**0,02**
A0A2Z5YMT0	DNA topoisomerase 1	5,7	**95,8**	873	0	NO_SP	1.000077	0.000000	0.000000	0.000000	0.000000	4	34	**8658966,667**	**48822**	**177,36**	**0,01**
A0A2Z5YA54	Methoxy mycolic acid synthase MmaA3	6,3	**33,6**	293	0	NO_SP	1.000075	0.000002	0.000000	0.000000	0.000000	4	17	**8445000**	**214907**	**39,30**	**0,03**
A0A2Z5Y8U8	Type VII secretion protein EccC	6,2	**146,1**	1333	1	NO_SP	0.999869	0.000174	0.000000	0.000000	0.000000	4	36	**8434600**	**184973**	**45,60**	**0,02**
B2HDJ1	Ribose-phosphate pyrophosphokinase	6,5	**35,5**	326	0	NO_SP	1.000053	0.000000	0.000000	0.000000	0.000000	3	14	**8353766,667**	**347363**	**24,05**	**0,04**
B2HII3	DNA-binding protein HU homolog	12,4	**22,8**	220	0	NO_SP	1.000057	0.000000	0.000000	0.000000	0.000000	2	7	**8331300**	**1500897**	**5,55**	**0,18**
A0A2Z5YEW0	L-gulono-1,4-lactone dehydrogenase	6,4	**48,1**	428	0	NO_SP	1.000063	0.000000	0.000000	0.000000	0.000000	3	19	**8312133,333**	**192787**	**43,12**	**0,02**
A0A117DU97	Oxidoreductase	5,2	**23,1**	223	0	NO_SP	1.000052	0.000000	0.000000	0.000000	0.000000	3	16	**8308700**	**62963**	**131,96**	**0,01**

B2HK64	Acetyl-coenzyme A synthetase	4,8	**71,3**	651	0	NO_SP	0.999957	0.000093	0.000001	0.000000	0.000000	4	25	**8307333,333**	**301440**	**27,56**	**0,04**
B2HIF9	Aspartyl/glutamyl-tRNA(Asn/Gln) amidotransferase subunit B	4,8	**54,2**	502	0	NO_SP	1.000005	0.000035	0.000000	0.000000	0.000000	3	21	**8208833,333**	**58642**	**139,98**	**0,01**
Q7X511	Antigen 85A	7,3	**35,6**	337	1	TAT	0.005698	0.187998	0.001421	0.747623	0.056711	11	10	**8159766,667**	**60369**	**135,17**	**0,01**
B2HF44	Polyketide synthase, Pks12	4,7	**434,3**	4187	0	NO_SP	1.000035	0.000006	0.000000	0.000000	0.000000	1	68	**8087300**	**152710**	**52,96**	**0,02**
A0A2Z5YNV9	ESX-1 secretion system protein EccA1	4,7	**62,2**	573	0	NO_SP	1.000043	0.000003	0.000000	0.000000	0.000000	2	25	**8066766,667**	**172197**	**46,85**	**0,02**
A0A2Z5YHP6	Probable cytosol aminopeptidase	6,4	**53,6**	519	0	NO_SP	0.999977	0.000067	0.000002	0.000000	0.000000	4	19	**8032900**	**556867**	**14,43**	**0,07**
B5TV81	ESAT-6-like protein	4,3	**10,7**	100	0	NO_SP	1.000062	0.000000	0.000000	0.000000	0.000000	5	7	**7964666,667**	**242013**	**32,91**	**0,03**
A0A2Z5YEQ1	Argininosuccinate synthase	4,8	**43,9**	398	0	NO_SP	1.000044	0.000000	0.000000	0.000000	0.000000	3	21	**7905700**	**115532**	**68,43**	**0,01**
A0A3E2MZU6	p-hydroxybenzoic acid--AMP ligase FadD22	4,6	**75,2**	702	0	NO_SP	1.000033	0.000009	0.000000	0.000000	0.000000	4	23	**7852000**	**104337**	**75,26**	**0,01**
B2HPD4	3-oxoacyl-[acyl-carrier protein] reductase, FabG1	5,7	**26,7**	255	0	NO_SP	1.000058	0.000001	0.000000	0.000000	0.000000	3	11	**7820233,333**	**2931763**	**2,67**	**0,37**
A0A2Z5YEU3	Type VII secretion protein EccC	5,1	**144,2**	1308	0	NO_SP	1.000037	0.000000	0.000000	0.000000	0.000000	4	35	**7739533,333**	**306967**	**25,21**	**0,04**
B2HD09	10 kDa chaperonin	4,3	**10,7**	100	0	NO_SP	1.000052	0.000001	0.000000	0.000000	0.000000	3	8	**7702800**	**163597**	**47,08**	**0,02**
B2HN29	Acetyl-CoA acyltransferase FadA2	6,5	**46,5**	441	0	NO_SP	1.000067	0.000001	0.000000	0.000000	0.000000	4	15	**7698066,667**	**279433**	**27,55**	**0,04**
A0A3E2MP68	Aldehyde dehydrogenase	9,4	**53,4**	493	0	NO_SP	1.000073	0.000000	0.000000	0.000000	0.000000	4	23	**7611966,667**	**171153**	**44,47**	**0,02**
A0A2Z5YA84	50S ribosomal protein L23	10,9	**11,0**	100	0	NO_SP	1.000081	0.000000	0.000000	0.000000	0.000000	3	5	**7483433,333**	**2702667**	**2,77**	**0,36**
B2HGR3	Cell division protein FtsZ	4,3	**39,2**	386	0	NO_SP	1.000065	0.000001	0.000000	0.000000	0.000000	4	17	**7398600**	**85148**	**86,89**	**0,01**
B2HMG4	Ribonuclease E Rne	4,1	**110,2**	1017	0	NO_SP	1.000059	0.000000	0.000000	0.000000	0.000000	2	27	**7347800**	**124671**	**58,94**	**0,02**
A0A2Z5Y9I0	Protein GrpE	4,4	**22,7**	217	0	NO_SP	1.000066	0.000000	0.000000	0.000000	0.000000	3	12	**7292366,667**	**33831**	**215,55**	**0,00**
A0A117DWS6	UPF0182 protein MPS_3353	9,2	**107,3**	987	7	NO_SP	1.000032	0.000000	0.000000	0.000000	0.000000	3	23	**7273600**	**163413**	**44,51**	**0,02**
A0A2Z5Y8T5	Membrane protein	4,4	**45,6**	421	2	NO_SP	0.938918	0.058344	0.001057	0.000268	0.000174	4	20	**7270966,667**	**279233**	**26,04**	**0,04**
B2HR04	Iron-regulated conserved protein	5,6	**15,2**	146	0	NO_SP	1.000041	0.000000	0.000000	0.000000	0.000000	3	12	**7213933,333**	**228127**	**31,62**	**0,03**
A0A2Z5YEI8	Triosephosphate isomerase	5,3	**27,3**	261	0	NO_SP	1.000057	0.000000	0.000000	0.000000	0.000000	3	17	**7204066,667**	**159836**	**45,07**	**0,02**
B2HJ43	Lysine--tRNA ligase	4,7	**55,5**	498	0	NO_SP	1.000049	0.000000	0.000000	0.000000	0.000000	5	19	**7161766,667**	**163553**	**43,79**	**0,02**
B2HRJ3	Uroporphyrin-III C-methyltransferase HemD	5,0	**58,2**	558	0	NO_SP	0.999994	0.000043	0.000001	0.000000	0.000000	4	16	**7158333,333**	**172433**	**41,51**	**0,02**
A0A2Z5Y9J9	Calcium dodecin	8,6	**8,0**	71	0	NO_SP	1.000050	0.000000	0.000000	0.000000	0.000000	3	5	**7130233,333**	**19512**	**365,43**	**0,00**
A0A2Z5Y8H0	Mammalian cell entry protein	5,1	**54,4**	517	1	NO_SP	0.999944	0.000068	0.000001	0.000001	0.000000	4	17	**7115666,667**	**217604**	**32,70**	**0,03**
A0A2Z5YBD4	Ribosome hibernation promoting factor	6,6	**26,2**	229	0	NO_SP	1.000052	0.000000	0.000000	0.000000	0.000000	3	7	**7022633,333**	**165940**	**42,32**	**0,02**
B2HPB5	Nitrogen fixation related protein	4,3	**17,4**	163	0	NO_SP	1.000074	0.000001	0.000000	0.000000	0.000000	4	2	**6977000**	**7036367**	**0,99**	**1,01**
B2HSN0	30S ribosomal protein S10	9,9	**11,4**	101	0	NO_SP	1.000064	0.000000	0.000000	0.000000	0.000000	3	6	**6975466,667**	**368773**	**18,92**	**0,05**
A0A100I1Z7	Acyl-CoA dehydrogenase, short-chain specific	4,8	**75,8**	706	0	NO_SP	1.000067	0.000001	0.000000	0.000000	0.000000	4	27	**6957466,667**	**149327**	**46,59**	**0,02**
A0A3E2MVH0	Pyruvate kinase	4,9	**50,8**	472	0	NO_SP	1.000039	0.000000	0.000000	0.000000	0.000000	4	21	**6953866,667**	**91355**	**76,12**	**0,01**
A0A2Z5YAJ5	(3R)-hydroxyacyl-ACP dehydratase subunit HadB	6,5	**15,0**	142	0	NO_SP	1.000059	0.000007	0.000000	0.000000	0.000000	3	5	**6932000**	**300600**	**23,06**	**0,04**
A0A2Z5YF84	ESX-5 secretion-associated protein EspG5	4,6	**32,4**	300	0	NO_SP	1.000062	0.000000	0.000000	0.000000	0.000000	3	10	**6918133,333**	**192513**	**35,94**	**0,03**
A0A3E2MYK8	50S ribosomal protein L2	11,9	**30,4**	280	0	NO_SP	1.000067	0.000000	0.000000	0.000000	0.000000	4	8	**6868866,667**	**709063**	**9,69**	**0,10**
B2HIB8	Single-stranded DNA-binding protein	4,8	**17,9**	175	0	NO_SP	1.000039	0.000001	0.000000	0.000000	0.000000	4	7	**6727933,333**	**265030**	**25,39**	**0,04**
A0A2Z5YDP4	TGc domain-containing protein	5,0	**121,9**	1111	0	NO_SP	1.000036	0.000000	0.000000	0.000000	0.000000	5	36	**6688133,333**	**84705**	**78,96**	**0,01**
A0A2Z5YAP9	Putative oxidoreductase	5,1	**38,5**	370	0	NO_SP	0.998684	0.001325	0.000005	0.000002	0.000001	4	15	**6666466,667**	**68414**	**97,44**	**0,01**
A0A3E2N1W6	Carbonic anhydrase	5,8	**21,7**	208	0	NO_SP	1.000059	0.000000	0.000000	0.000000	0.000000	4	6	**6604266,667**	**198530**	**33,27**	**0,03**
A0A3E2MZV5	Doxorubicin resistance ATP-binding protein DrrA	5,9	**35,8**	331	0	NO_SP	1.000050	0.000000	0.000000	0.000000	0.000000	4	23	**6575600**	**71457**	**92,02**	**0,01**
A0A2Z5YC91	Long-chain-fatty-acid--AMP ligase FadD29	5,2	**67,7**	622	0	NO_SP	1.000047	0.000001	0.000000	0.000000	0.000000	4	20	**6575466,667**	**113762**	**57,80**	**0,02**
A0A100I554	Ribonucleoside-diphosphate reductase	6,5	**79,2**	693	0	NO_SP	1.000071	0.000000	0.000000	0.000000	0.000000	3	24	**6565266,667**	**191333**	**34,31**	**0,03**
B2HPV3	Fructose-bisphosphate aldolase	4,8	**36,6**	344	0	NO_SP	1.000064	0.000000	0.000000	0.000000	0.000000	4	16	**6540866,667**	**71626**	**91,32**	**0,01**
A0A2Z5YLB1	Magnesium chelatase	5,0	**50,0**	464	0	NO_SP	1.000050	0.000000	0.000000	0.000000	0.000000	4	18	**6517166,667**	**3954**	**1648,19**	**0,00**
B2HSN1	50S ribosomal protein L3	10,8	**23,0**	217	0	NO_SP	1.000043	0.000000	0.000000	0.000000	0.000000	3	9	**6506566,667**	**365997**	**17,78**	**0,06**
A0A2Z5YM62	Universal stress protein	5,5	**32,0**	304	0	NO_SP	1.000053	0.000000	0.000000	0.000000	0.000000	4	9	**6444766,667**	**27595**	**233,55**	**0,00**
A0A2Z5YGJ3	Pup--protein ligase	6,5	**50,6**	447	0	NO_SP	1.000053	0.000000	0.000000	0.000000	0.000000	3	19	**6441800**	**166892**	**38,60**	**0,03**
B2HS42	Iron-sulfur cluster carrier protein	5,5	**40,7**	386	0	NO_SP	1.000036	0.000000	0.000000	0.000000	0.000000	3	12	**6419866,667**	**88085**	**72,88**	**0,01**
A0A2Z5YN15	Amidohydrolase	4,8	**43,7**	391	0	NO_SP	1.000061	0.000000	0.000000	0.000000	0.000000	3	3	**6365733,333**	**6852800**	**0,93**	**1,08**
B2HRY7	Succinate-semialdehyde dehydrogenase [NADP+] dependent (SsdH) GabD2	6,5	**55,2**	518	0	NO_SP	1.000069	0.000000	0.000000	0.000000	0.000000	3	12	**6335366,667**	**127280**	**49,78**	**0,02**
A0A2Z5YEY2	Putative RNA binding protein, contains S1 domain	6,0	**84,6**	787	0	NO_SP	1.000068	0.000000	0.000000	0.000000	0.000000	4	31	**6301300**	**141407**	**44,56**	**0,02**
A0A2Z5Y8F9	NAD(P) transhydrogenase subunit alpha	4,4	**37,3**	362	0	NO_SP	0.999247	0.000780	0.000005	0.000001	0.000001	4	15	**6263733,333**	**65780**	**95,22**	**0,01**
B2HNC1	Alanine--tRNA ligase	5,2	**96,5**	901	0	NO_SP	1.000071	0.000000	0.000000	0.000000	0.000000	3	22	**6257300**	**98219**	**63,71**	**0,02**
A0A100I1G9	UDP-glucose 4-epimerase	5,7	**33,6**	314	0	NO_SP	1.000042	0.000002	0.000000	0.000000	0.000000	3	12	**6221666,667**	**124123**	**50,12**	**0,02**
A0A2Z5YDK2	Sugar ABC transporter ATP-binding protein	7,3	**39,0**	360	0	NO_SP	1.000054	0.000000	0.000000	0.000000	0.000000	4	15	**6203333,333**	**175220**	**35,40**	**0,03**
A0A2Z5YJW4	2,3,4,5-tetrahydropyridine-2,6-dicarboxylate N-succinyltransferase	5,3	**32,2**	314	0	NO_SP	0.970470	0.029152	0.000280	0.000046	0.000029	4	16	**6176133,333**	**84171**	**73,38**	**0,01**
B2HET8	Conserved hypothetical secreted protein	3,8	**7,3**	71	0	NO_SP	0.999635	0.000413	0.000010	0.000001	0.000000	4	3	**6122366,667**	**1571620**	**3,90**	**0,26**
A0A2Z5Y9K5	Heparin-binding hemagglutinin	9,3	**21,9**	201	0	NO_SP	1.000028	0.000000	0.000000	0.000000	0.000000	3	8	**6100133,333**	**177780**	**34,31**	**0,03**
B2HL06	Protein RecA	5,2	**36,9**	346	0	NO_SP	1.000075	0.000000	0.000000	0.000000	0.000000	4	21	**6062400**	**9263**	**654,45**	**0,00**
A0A2Z5YDG4	Pyridoxal 5-phosphate synthase subunit PdxS	5,1	**33,4**	317	0	NO_SP	1.000029	0.000000	0.000000	0.000000	0.000000	3	11	**6043866,667**	**47365**	**127,60**	**0,01**
A0A2Z5YDG5	Transaldolase	4,4	**40,1**	373	0	NO_SP	1.000070	0.000000	0.000000	0.000000	0.000000	4	18	**6037600**	**230137**	**26,23**	**0,04**
A0A2Z5Y8H1	Lipoprotein	9,0	**43,0**	401	0	LIPO	0.070051	0.275289	0.405563	0.005242	0.242380	4	14	**6013066,667**	**211190**	**28,47**	**0,04**
A0A2Z5YFR9	Thiol peroxidase	4,2	**17,2**	167	0	NO_SP	0.999939	0.000102	0.000003	0.000000	0.000000	4	8	**6012000**	**65594**	**91,65**	**0,01**
B2HHG7	NADP-dependent alcohol dehydrogenase Adh	5,4	**37,2**	346	0	NO_SP	1.000070	0.000000	0.000000	0.000000	0.000000	4	20	**5996300**	**58310**	**102,83**	**0,01**
A0A2Z5YJX9	ATP-dependent Clp protease ATP-binding subunit ClpX	4,8	**46,8**	426	0	NO_SP	1.000043	0.000000	0.000000	0.000000	0.000000	3	20	**5993300**	**96569**	**62,06**	**0,02**
A0A2Z5YJU6	Homoserine dehydrogenase	4,5	**45,8**	441	0	NO_SP	1.000051	0.000000	0.000000	0.000000	0.000000	3	15	**5989300**	**134441**	**44,55**	**0,02**
A0A100HZD7	Indole-3-glycerol phosphate synthase	5,2	**27,7**	267	0	NO_SP	1.000060	0.000000	0.000000	0.000000	0.000000	3	17	**5949333,333**	**79048**	**75,26**	**0,01**
A0A2Z5YE68	Modulator of FtsH protease HflK	5,1	**41,8**	384	1	NO_SP	0.898437	0.100313	0.000312	0.000190	0.000132	3	11	**5936000**	**176953**	**33,55**	**0,03**
A0A2Z5Y8I5	Mammalian cell entry protein	7,4	**37,7**	346	1	NO_SP	0.988331	0.010923	0.000097	0.000059	0.000037	3	11	**5920900**	**267300**	**22,15**	**0,05**
A0A2Z5Y7E0	Beta sliding clamp	4,4	**40,5**	385	0	NO_SP	1.000033	0.000021	0.000001	0.000000	0.000000	4	10	**5901633,333**	**27056**	**218,13**	**0,00**
B2HQU4	Conserved membrane protein	7,4	**36,6**	348	1	NO_SP	1.000068	0.000000	0.000000	0.000000	0.000000	4	17	**5883066,667**	**115335**	**51,01**	**0,02**
B2HGU5	Phospho-2-dehydro-3-deoxyheptonate aldolase	5,4	**50,6**	462	0	NO_SP	1.000057	0.000000	0.000000	0.000000	0.000000	4	15	**5849166,667**	**107489**	**54,42**	**0,02**
A0A3E2MQK9	Aspartate--tRNA(Asp/Asn) ligase	4,9	**64,2**	591	0	NO_SP	0.999395	0.000595	0.000047	0.000001	0.000001	4	18	**5797500**	**111795**	**51,86**	**0,02**
A0A2Z5YA62	UPF0336 protein DAVIS_03420	4,6	**18,7**	166	0	NO_SP	1.000046	0.000002	0.000000	0.000000	0.000000	1	8	**5780366,667**	**109648**	**52,72**	**0,02**
A0A2Z5YKL3	Polyketide synthase	4,9	**58,5**	544	0	NO_SP	1.000045	0.000008	0.000000	0.000000	0.000000	4	17	**5755400**	**80193**	**71,77**	**0,01**
A0A2Z5Y8P1	Succinate-semialdehyde dehydrogenase [NADP(+)] 1	4,5	**48,6**	458	0	NO_SP	1.000006	0.000040	0.000000	0.000000	0.000000	1	17	**5750900**	**153466**	**37,47**	**0,03**
A0A3E2MQN4	4-aminobutyrate aminotransferase PuuE	4,7	**47,1**	446	0	NO_SP	1.000064	0.000001	0.000000	0.000000	0.000000	4	23	**5745000**	**55120**	**104,23**	**0,01**
A0A2Z5YPF9	Putative ATPase	4,4	**33,7**	318	0	NO_SP	1.000054	0.000012	0.000000	0.000000	0.000000	4	13	**5714066,667**	**95186**	**60,03**	**0,02**

B2HPS9	Chaperone protein ClpB	4,8	**92,8**	848	0	NO_SP	1.000066	0.000000	0.000000	0.000000	0.000000	4	26	**5687666,667**	**14980**	**379,68**	**0,00**
A0A2Z5YEL3	Phenylalanine--tRNA ligase beta subunit	4,8	**88,0**	829	0	NO_SP	1.000050	0.000000	0.000000	0.000000	0.000000	5	26	**5674200**	**84634**	**67,04**	**0,01**
A0A2Z5YCG4	Translation initiation factor IF-2	6,8	**98,3**	949	0	NO_SP	1.000025	0.000000	0.000000	0.000000	0.000000	3	27	**5660300**	**92583**	**61,14**	**0,02**
A0A3E2MX79	LGFP repeat protein	4,3	**80,6**	766	2	SP	0.000270	0.999047	0.000173	0.000203	0.000160	4	20	**5622466,667**	**29366**	**191,46**	**0,01**
B2HQT3	3-hydroxybutyryl-CoA dehydrogenase FadB2	5,2	**30,9**	288	0	NO_SP	1.000062	0.000000	0.000000	0.000000	0.000000	4	15	**5617066,667**	**121442**	**46,25**	**0,02**
B2HQM5	Phosphomethylpyrimidine synthase	5,1	**59,4**	546	0	NO_SP	0.999314	0.000645	0.000062	0.000001	0.000001	5	17	**5615300**	**29947**	**187,51**	**0,01**
B2HEQ0	Two-component sensory transduction transcriptional regulatory protein MtrA	5,4	**24,9**	225	0	NO_SP	1.000075	0.000000	0.000000	0.000000	0.000000	5	11	**5596200**	**47844**	**116,97**	**0,01**
A0A2Z5YPV4	ESX-1 secretion-associated protein EspG1	5,9	**29,7**	279	0	NO_SP	1.000051	0.000002	0.000000	0.000000	0.000000	5	11	**5516200**	**73823**	**74,72**	**0,01**
A0A2Z5YM82	DNA-binding response regulator	5,4	**26,7**	240	0	NO_SP	1.000049	0.000000	0.000000	0.000000	0.000000	3	12	**5513966,667**	**46016**	**119,83**	**0,01**
B2HGW4	Cytochrome bc1 complex cytochrome b subunit	7,6	**62,2**	561	8	NO_SP	0.999976	0.000039	0.000000	0.000000	0.000000	4	10	**5512566,667**	**237157**	**23,24**	**0,04**
A0A2Z5Y8S5	Fumarate reductase	6,9	**28,6**	248	0	NO_SP	1.000048	0.000000	0.000000	0.000000	0.000000	3	15	**5503266,667**	**114818**	**47,93**	**0,02**
A0A2Z5YJ80	Acyl-CoA dehydrogenase	5,0	**41,4**	388	0	NO_SP	1.000073	0.000000	0.000000	0.000000	0.000000	4	15	**5501033,333**	**70159**	**78,41**	**0,01**
A0A100IF67	3-hydroxyacyl-CoA dehydrogenase	5,4	**26,3**	253	0	NO_SP	1.000029	0.000007	0.000000	0.000000	0.000000	3	16	**5482533,333**	**62900**	**87,16**	**0,01**
B2HQV4	2,3-bisphosphoglycerate-dependent phosphoglycerate mutase	5,0	**27,5**	251	0	NO_SP	1.000061	0.000001	0.000000	0.000000	0.000000	4	16	**5463900**	**45879**	**119,09**	**0,01**
A0A2Z5YDG9	35 kDa protein	6,1	**29,2**	271	0	NO_SP	1.000038	0.000000	0.000000	0.000000	0.000000	4	17	**5456200**	**72985**	**74,76**	**0,01**
A0A100I232	Glycosyl transferase	6,6	**70,3**	626	0	NO_SP	0.999273	0.000526	0.000137	0.000011	0.000003	4	20	**5444266,667**	**106840**	**50,96**	**0,02**
B2HS44	Serine protease HtrA (DegP protein)	4,7	**52,0**	504	1	NO_SP	1.000059	0.000000	0.000000	0.000000	0.000000	4	20	**5422466,667**	**9038**	**599,94**	**0,00**
A0A100I2N1	Phosphoserine aminotransferase	4,5	**40,0**	376	0	NO_SP	1.000038	0.000001	0.000000	0.000000	0.000000	3	13	**5417733,333**	**77202**	**70,18**	**0,01**
A0A2Z5YJX3	L-3-phosphoserine phosphatase	10,4	**69,2**	649	0	NO_SP	1.000068	0.000001	0.000000	0.000000	0.000000	4	20	**5375633,333**	**261177**	**20,58**	**0,05**
B2HKE9	Conserved transcriptional regulator	5,4	**46,7**	428	0	NO_SP	1.000053	0.000000	0.000000	0.000000	0.000000	4	14	**5356166,667**	**87734**	**61,05**	**0,02**
B2HQ37	HpcH_HpaI domain-containing protein	5,2	**29,5**	266	0	NO_SP	1.000063	0.000000	0.000000	0.000000	0.000000	3	9	**5342966,667**	**88189**	**60,59**	**0,02**
A0A100I7H0	Cytochrome aa3 subunit 2	5,9	**36,5**	325	2	NO_SP	0.999845	0.000184	0.000000	0.000000	0.000000	4	11	**5314333,333**	**17120**	**310,41**	**0,00**
B2HKD3	2-isopropylmalate synthase	4,6	**66,1**	606	0	NO_SP	1.000075	0.000000	0.000000	0.000000	0.000000	1	20	**5282400**	**49002**	**107,80**	**0,01**
A0A2Z5YIC9	Assimilatory sulfite reductase (ferredoxin)	5,7	**61,9**	555	0	NO_SP	0.999763	0.000229	0.000008	0.000001	0.000000	4	20	**5261533,333**	**24973**	**210,69**	**0,00**
A0A3E2N2M0	Arsenical pump-driving ATPase	6,4	**42,1**	392	0	NO_SP	1.000058	0.000002	0.000000	0.000000	0.000000	5	20	**5253833,333**	**424360**	**12,38**	**0,08**
B2HNV0	Fatty-acid-CoA ligase FadD2	5,8	**60,3**	557	0	NO_SP	1.000073	0.000000	0.000000	0.000000	0.000000	4	18	**5180666,667**	**155267**	**33,37**	**0,03**
A0A3E2MZU5	Trans-acting enoyl reductase	8,9	**45,0**	418	0	NO_SP	1.000044	0.000000	0.000000	0.000000	0.000000	4	16	**5167066,667**	**144323**	**35,80**	**0,03**
B2HGQ3	Mycobactin synthetase protein B	4,8	**741,8**	6887	0	NO_SP	1.000051	0.000000	0.000000	0.000000	0.000000	8	49	**5160733,333**	**126116**	**40,92**	**0,02**
B2HNL4	Short-chain type dehydrogenase/reductase	8,1	**27,9**	268	0	NO_SP	1.000051	0.000002	0.000000	0.000000	0.000000	4	18	**5109100**	**85304**	**59,89**	**0,02**
B2HNJ2	Diacylglycerol O-acyltransferase	7,2	**51,8**	487	0	NO_SP	1.000060	0.000000	0.000000	0.000000	0.000000	4	13	**5102100**	**184937**	**27,59**	**0,04**
A0A2Z5YFC2	VWFA domain-containing protein	4,3	**75,9**	735	1	NO_SP	1.000050	0.000002	0.000000	0.000000	0.000000	4	16	**5100166,667**	**4751700**	**1,07**	**0,93**
A0A2Z5YC67	Signal recognition particle protein	8,1	**55,0**	526	0	NO_SP	1.000041	0.000000	0.000000	0.000000	0.000000	3	16	**5077300**	**178343**	**28,47**	**0,04**
A0A2Z5YL76	Bifunctional 2-hydroxyhepta-2,4-diene-1,7-dioate isomerase/cyclase/dehydra	4,4	**69,2**	643	0	NO_SP	1.000058	0.000004	0.000001	0.000000	0.000000	4	6	**5062766,667**	**5107033**	**0,99**	**1,01**
A0A2Z5YCQ2	Putative lipoprotein LppX	5,0	**24,0**	229	1	LIPO	0.000000	0.000001	1.000073	0.000000	0.000000	3	13	**5033633,333**	**142694**	**35,28**	**0,03**
B2HP63	Riboflavin biosynthesis protein RibD	6,7	**35,3**	339	0	NO_SP	1.000069	0.000000	0.000000	0.000000	0.000000	2	15	**5026900**	**31342**	**160,39**	**0,01**
A0A2Z5YEG5	CTP synthase	5,7	**63,7**	583	0	NO_SP	1.000099	0.000000	0.000000	0.000000	0.000000	3	20	**4997800**	**72920**	**68,54**	**0,01**
A0A3E2N3B0	Uncharacterized protein	4,7	**15,9**	155	0	NO_SP	1.000068	0.000001	0.000000	0.000000	0.000000	4	6	**4991033,333**	**67776**	**73,64**	**0,01**
B2HI01	Acetohydroxyacid synthase IlvX	4,7	**52,4**	515	0	NO_SP	1.000072	0.000001	0.000000	0.000000	0.000000	4	14	**4974166,667**	**66900**	**74,35**	**0,01**
A0A2Z5YCQ5	Polyphosphate kinase	5,6	**81,4**	731	0	NO_SP	0.998423	0.001605	0.000003	0.000002	0.000001	8	22	**4942700**	**66969**	**73,81**	**0,01**
A0A2Z5Y9E2	Putative lipoprotein aminopeptidase LpqL	4,6	**51,5**	490	0	LIPO	0.000099	0.000007	0.999929	0.000000	0.000000	4	11	**4938866,667**	**27504**	**179,57**	**0,01**
B2HJA8	Uncharacterized protein	5,2	**37,1**	343	0	NO_SP	0.999213	0.000800	0.000015	0.000004	0.000003	1	10	**4936766,667**	**18296**	**269,83**	**0,00**
A0A2Z5YIA6	CP_ATPgrasp_1 domain-containing protein	5,1	**57,3**	514	0	NO_SP	1.000069	0.000000	0.000000	0.000000	0.000000	2	18	**4929933,333**	**64710**	**76,18**	**0,01**
A0A2Z5YPI5	Aspartokinase	4,8	**44,4**	421	0	NO_SP	1.000071	0.000000	0.000000	0.000000	0.000000	4	13	**4911566,667**	**78792**	**62,34**	**0,02**
A0A2Z5YIP1	2-oxoglutarate oxidoreductase subunit KorB	5,4	**39,0**	363	0	NO_SP	1.000063	0.000000	0.000000	0.000000	0.000000	3	18	**4910800**	**70518**	**69,64**	**0,01**
A0A2Z5YCF8	Thioredoxin-like_fold domain-containing protein	6,2	**27,1**	255	1	NO_SP	1.000064	0.000003	0.000000	0.000000	0.000000	4	13	**4874466,667**	**84302**	**57,82**	**0,02**
A0A2Z5YLX4	Phosphoribosylformylglycinamidine synthase subunit PurL	4,5	**80,6**	765	0	NO_SP	1.000051	0.000003	0.000000	0.000000	0.000000	3	21	**4842033,333**	**60089**	**80,58**	**0,01**
A0A2Z5YL93	Long-chain-acyl-CoA synthetase	8,2	**63,8**	593	0	NO_SP	1.000042	0.000001	0.000000	0.000000	0.000000	4	24	**4829766,667**	**110390**	**43,75**	**0,02**
A0A2Z5Y7T5	LLM class F420-dependent oxidoreductase	5,5	**37,5**	346	0	NO_SP	1.000025	0.000000	0.000000	0.000000	0.000000	3	16	**4769500**	**51687**	**92,28**	**0,01**
B2HQL7	Threonine synthase	6,6	**37,4**	360	0	NO_SP	1.000044	0.000000	0.000000	0.000000	0.000000	3	17	**4737133,333**	**43019**	**110,12**	**0,01**
B2HMN2	Superoxide dismutase	6,1	**23,2**	207	0	NO_SP	1.000039	0.000004	0.000000	0.000000	0.000000	11	10	**4707633,333**	**6064**	**776,28**	**0,00**
B2HIN1	Phenolpthiocerol synthesis type-I polyketide synthase PpsA	4,8	**169,7**	1602	0	NO_SP	1.000061	0.000001	0.000000	0.000000	0.000000	1	33	**4705800**	**68722**	**68,48**	**0,01**
A0A2Z5YK33	ATP-dependent RNA helicase DeaD	9,5	**62,6**	571	0	NO_SP	1.000019	0.000003	0.000000	0.000000	0.000000	4	22	**4698466,667**	**23567**	**199,37**	**0,01**
A0A2Z5Y9Q8	F420-dependent glucose-6-phosphate dehydrogenase	5,2	**37,3**	336	0	NO_SP	1.000073	0.000001	0.000000	0.000000	0.000000	4	15	**4681500**	**83246**	**56,24**	**0,02**
B2HSY6	6-phosphogluconate dehydrogenase, decarboxylating	5,2	**51,4**	483	0	NO_SP	1.000087	0.000000	0.000000	0.000000	0.000000	4	18	**4671100**	**51545**	**90,62**	**0,01**
A0A2Z5YNL6	3-oxosteroid 1-dehydrogenase	7,6	**61,4**	567	0	NO_SP	1.000017	0.000023	0.000001	0.000000	0.000000	4	6	**4664200**	**1807**	**2581,90**	**0,00**
A0A2Z5YNG5	Putative acyl-[acyl-carrier-protein] desaturase DesA1	6,5	**39,0**	338	0	NO_SP	1.000048	0.000013	0.000000	0.000000	0.000000	4	14	**4652833,333**	**66169**	**70,32**	**0,01**
A0A2Z5YAH2	Ferredoxin reductase	4,6	**42,4**	401	0	NO_SP	1.000061	0.000000	0.000000	0.000000	0.000000	4	18	**4652066,667**	**23651**	**196,70**	**0,01**
A0A2Z5YCB6	50S ribosomal protein L28	11,9	**6,9**	64	0	NO_SP	1.000034	0.000000	0.000000	0.000000	0.000000	3	5	**4642866,667**	**2681000**	**1,73**	**0,58**
B2HKS8	ABC-type sugar transport protein	8,0	**48,2**	437	0	TATLIPO	0.000000	0.000000	0.000086	0.000035	0.999891	5	12	**4626466,667**	**162950**	**28,39**	**0,04**
A0A2Z5YHF1	Flavoprotein	5,5	**55,4**	526	0	NO_SP	1.000072	0.000000	0.000000	0.000000	0.000000	4	17	**4624066,667**	**85687**	**53,96**	**0,02**
A0A2Z5YDR3	Probable cell division protein WhiA	10,7	**35,1**	327	0	NO_SP	1.000062	0.000000	0.000000	0.000000	0.000000	4	13	**4616000**	**121393**	**38,03**	**0,03**
A0A3E2MWX0	Proline--tRNA ligase	4,7	**63,1**	578	0	NO_SP	1.000032	0.000000	0.000000	0.000000	0.000000	4	26	**4597300**	**55366**	**83,03**	**0,01**
A0A2Z5YB44	ATP-dependent RNA helicase RhlE	8,6	**55,4**	517	0	NO_SP	1.000064	0.000001	0.000000	0.000000	0.000000	4	15	**4597033,333**	**73594**	**62,46**	**0,02**
A0A2Z5YKK2	Beta-thionase	5,1	**48,6**	464	0	NO_SP	1.000071	0.000000	0.000000	0.000000	0.000000	4	17	**4586600**	**91974**	**49,87**	**0,02**
B2HQ93	Biotin synthase	4,4	**37,6**	349	0	NO_SP	1.000041	0.000003	0.000000	0.000000	0.000000	4	12	**4583166,667**	**84000**	**54,56**	**0,02**
A0A2Z5YAM6	50S ribosomal protein L6	10,8	**19,5**	179	0	NO_SP	1.000070	0.000002	0.000000	0.000000	0.000000	3	9	**4568866,667**	**425880**	**10,73**	**0,09**
A0A2Z5YJ93	Biotin carboxyl carrier protein	4,9	**71,6**	669	0	NO_SP	1.000056	0.000000	0.000000	0.000000	0.000000	6	21	**4567933,333**	**2788667**	**1,64**	**0,61**
A0A3E2MVF9	Phosphoribosyl isomerase A	4,5	**25,5**	244	0	NO_SP	0.999995	0.000046	0.000001	0.000000	0.000000	4	11	**4552000**	**55911**	**81,42**	**0,01**
A0A2Z5YG04	Glycine dehydrogenase (decarboxylating)	5,1	**100,5**	945	0	NO_SP	1.000039	0.000002	0.000000	0.000000	0.000000	4	26	**4543733,333**	**79564**	**57,11**	**0,02**
B2HT74	Cystathionine gamma-synthase MetB (Cgs)	5,5	**40,7**	388	0	NO_SP	0.999952	0.000075	0.000001	0.000000	0.000000	4	11	**4533300**	**0**	**#JAKO/0!**	**0,00**
B2HMS7	Conserved hypothetical transmembrane protein	4,2	**43,6**	418	0	NO_SP	1.000031	0.000010	0.000000	0.000000	0.000000	5	7	**4526033,333**	**1510**	**2997,37**	**0,00**
A0A2Z5Y7L0	30S ribosomal protein S6	8,5	**10,9**	96	0	NO_SP	1.000069	0.000000	0.000000	0.000000	0.000000	3	8	**4518666,667**	**205897**	**21,95**	**0,05**
A0A2Z5YM93	Phosphoribosylformylglycinamidine synthase subunit PurQ	4,6	**23,6**	224	0	NO_SP	1.000057	0.000001	0.000000	0.000000	0.000000	3	11	**4516466,667**	**77233**	**58,48**	**0,02**
A0A2Z5YNP4	Aspartate-semialdehyde dehydrogenase	4,7	**36,9**	352	0	NO_SP	1.000076	0.000000	0.000000	0.000000	0.000000	4	14	**4505433,333**	**31071**	**145,00**	**0,01**
B2HQ15	UDP-glucose 6-dehydrogenase, UdgL	5,4	**46,2**	418	0	NO_SP	1.000063	0.000001	0.000000	0.000000	0.000000	4	15	**4499966,667**	**74041**	**60,78**	**0,02**
A0A2Z5YCF0	Uncharacterized protein	6,7	**29,9**	265	0	NO_SP	1.000045	0.000000	0.000000	0.000000	0.000000	4	12	**4495066,667**	**522800**	**8,60**	**0,12**
A0A2Z5YAI5	Transcription termination/antitermination protein NusG	4,4	**25,6**	237	0	NO_SP	1.000041	0.000000	0.000000	0.000000	0.000000	3	12	**4462400**	**27571**	**161,85**	**0,01**

A0A2Z5YBY0	Putative NAD-dependent oxidoreductase	5,4	**29,9**	282	0	NO_SP	1.000040	0.000000	0.000000	0.000000	0.000000	3	14	**4451033,333**	**9844**	**452,17**	**0,00**
B2HR14	50S ribosomal protein L20	11,6	**14,5**	129	0	NO_SP	1.000052	0.000000	0.000000	0.000000	0.000000	3	4	**4446266,667**	**1174333**	**3,79**	**0,26**
A0A2Z5YBN5	Cell division ATP-binding protein FtsE	10,0	**25,5**	229	0	NO_SP	1.000079	0.000000	0.000000	0.000000	0.000000	3	12	**4409633,333**	**259433**	**17,00**	**0,06**
B2HD59	Alkyl hydroperoxide reductase AhpD	4,9	**18,6**	178	0	NO_SP	1.000075	0.000000	0.000000	0.000000	0.000000	3	7	**4393366,667**	**28091**	**156,40**	**0,01**
B2HIN0	Phenolpthiocerol synthesis type-I polyketide synthase PpsB	4,7	**161,7**	1524	0	NO_SP	0.987299	0.012357	0.000213	0.000051	0.000026	1	21	**4383533,333**	**78554**	**55,80**	**0,02**
A0A3E2MPR5	Acetolactate synthase isozyme 2 large subunit	4,9	**64,5**	606	0	NO_SP	1.000016	0.000010	0.000000	0.000000	0.000000	4	19	**4351033,333**	**47920**	**90,80**	**0,01**
A0A2Z5YF25	Tryptophan synthase beta chain	5,9	**45,1**	425	0	NO_SP	1.000073	0.000000	0.000000	0.000000	0.000000	4	12	**4349733,333**	**140427**	**30,98**	**0,03**
A0A2Z5YLN8	Bifunctional purine biosynthesis protein PurH	5,6	**55,5**	523	0	NO_SP	1.000041	0.000000	0.000000	0.000000	0.000000	4	15	**4347200**	**75354**	**57,69**	**0,02**
A0A2Z5YA72	Mycolic acid methyltransferase MmaA1	4,8	**32,7**	286	0	NO_SP	1.000068	0.000000	0.000000	0.000000	0.000000	4	7	**4321800**	**168220**	**25,69**	**0,04**
A0A3E2MTE3	UPF0336 protein DAVIS_03422	4,3	**17,6**	159	0	NO_SP	1.000041	0.000007	0.000000	0.000000	0.000000	4	10	**4270466,667**	**24525**	**174,13**	**0,01**
A0A2Z5YI00	Branched-chain-amino-acid aminotransferase	4,9	**39,8**	368	0	NO_SP	1.000020	0.000017	0.000000	0.000000	0.000000	3	13	**4261233,333**	**86601**	**49,21**	**0,02**
B2HDR8	Bifunctional protein FolD	6,6	**29,7**	281	0	NO_SP	1.000071	0.000000	0.000000	0.000000	0.000000	4	13	**4258966,667**	**13803**	**308,55**	**0,00**
B2HDW7	Glycerol-3-phosphate dehydrogenase	6,2	**63,3**	585	0	NO_SP	0.999936	0.000095	0.000002	0.000000	0.000000	3	17	**4250000**	**106840**	**39,78**	**0,03**
A0A2Z5YH98	AAA ATPase forming ring-shaped complexes	4,5	**67,5**	609	0	NO_SP	1.000052	0.000000	0.000000	0.000000	0.000000	4	19	**4247833,333**	**50335**	**84,39**	**0,01**
A0A2Z5YFV7	Peptidase_M28 domain-containing protein	6,4	**43,4**	401	0	NO_SP	1.000039	0.000002	0.000000	0.000000	0.000000	4	10	**4235600**	**84632**	**50,05**	**0,02**
B2HQF7	Ribonuclease PH	5,2	**28,8**	276	0	NO_SP	1.000061	0.000001	0.000000	0.000000	0.000000	3	10	**4207100**	**51584**	**81,56**	**0,01**
A0A2Z5YNY7	Universal stress protein	5,3	**32,4**	306	0	NO_SP	1.000054	0.000000	0.000000	0.000000	0.000000	3	10	**4187033,333**	**14267**	**293,48**	**0,00**
A0A2Z5YF08	ESX-5 secretion system protein EccA5	5,0	**66,2**	596	0	NO_SP	0.999742	0.000292	0.000003	0.000000	0.000000	4	23	**4185533,333**	**90953**	**46,02**	**0,02**
B2HD25	Cholesterol oxidase ChoD	9,5	**63,4**	581	0	NO_SP	1.000038	0.000003	0.000000	0.000000	0.000000	3	15	**4164666,667**	**883903**	**4,71**	**0,21**
A0A2Z5YNS3	Uracil-xanthine permease	6,7	**61,3**	578	12	NO_SP	0.999863	0.000160	0.000000	0.000000	0.000000	4	2	**4146466,667**	**11465**	**361,67**	**0,00**
A0A100I200	NADH-dependent glutamate synthase	4,7	**50,6**	466	0	NO_SP	1.000032	0.000000	0.000000	0.000000	0.000000	3	17	**4144033,333**	**40967**	**101,16**	**0,01**
A0A2Z5YHY9	Adenosine kinase	4,4	**34,4**	324	0	NO_SP	0.998435	0.001557	0.000013	0.000004	0.000002	3	10	**4139633,333**	**82749**	**50,03**	**0,02**
A0A100I516	Electron transfer flavoprotein subunit beta	4,4	**27,3**	259	0	NO_SP	1.000056	0.000004	0.000000	0.000000	0.000000	3	15	**4084066,667**	**79783**	**51,19**	**0,02**
A0A2Z5YAY4	Succinate dehydrogenase iron-sulfur subunit	7,0	**30,0**	270	0	NO_SP	0.999583	0.000336	0.000066	0.000004	0.000001	3	12	**4083900**	**109174**	**37,41**	**0,03**
B2HJQ1	4-hydroxy-3-methylbut-2-en-1-yl diphosphate synthase (flavodoxin)	4,9	**40,6**	387	0	NO_SP	1.000019	0.000014	0.000000	0.000000	0.000000	5	16	**4071700**	**50627**	**80,43**	**0,01**
A0A2Z5YPL5	Thioredoxin reductase	4,4	**35,2**	332	0	NO_SP	1.000016	0.000035	0.000001	0.000000	0.000000	4	12	**4067700**	**43091**	**94,40**	**0,01**
A0A2Z5YK79	GTP-binding protein	4,8	**63,3**	588	0	NO_SP	1.000068	0.000000	0.000000	0.000000	0.000000	1	15	**4063833,333**	**58729**	**69,20**	**0,01**
B2HL73	Mycobactin synthetase protein B	5,1	**344,8**	3168	0	NO_SP	1.000018	0.000014	0.000000	0.000000	0.000000	1	1	**4061500**	**4270891**	**0,95**	**1,05**
A0A2Z5YJX8	Uncharacterized protein	4,8	**17,9**	163	0	NO_SP	1.000052	0.000000	0.000000	0.000000	0.000000	4	7	**4057666,667**	**74908**	**54,17**	**0,02**
A0A2Z5YAY3	NAD(P)H-quinone dehydrogenase	6,4	**49,3**	470	0	NO_SP	1.000040	0.000000	0.000000	0.000000	0.000000	4	17	**4057166,667**	**46856**	**86,59**	**0,01**
A0A2Z5YBZ0	CoA ester lyase	4,5	**32,8**	305	0	NO_SP	1.000060	0.000001	0.000000	0.000000	0.000000	3	13	**4049233,333**	**25721**	**157,43**	**0,01**
A0A2Z5YIU9	Lectin	4,2	**21,9**	207	0	NO_SP	1.000042	0.000001	0.000000	0.000000	0.000000	3	8	**4041133,333**	**28458**	**142,00**	**0,01**
A0A2Z5YEE8	Glycerol-3-phosphate acyltransferase	5,8	**69,7**	624	0	NO_SP	1.000074	0.000002	0.000000	0.000000	0.000000	3	20	**4027166,667**	**77779**	**51,78**	**0,02**
B2HIL3	Ketoreductase	9,7	**41,9**	385	0	NO_SP	0.999975	0.000060	0.000003	0.000000	0.000000	4	18	**4012533,333**	**73653**	**54,48**	**0,02**
A0A2Z5YDX3	Universal stress protein	5,0	**27,9**	264	0	NO_SP	1.000047	0.000000	0.000000	0.000000	0.000000	4	12	**3981633,333**	**57576**	**69,15**	**0,01**
A0A3E2MPT4	Putative NAD(P)H nitroreductase acg	5,4	**55,0**	494	0	NO_SP	1.000077	0.000000	0.000000	0.000000	0.000000	4	9	**3979266,667**	**21508**	**185,01**	**0,01**
B2HS28	Conserved transmembrane ATP-binding protein ABC transporter	8,1	**93,3**	877	6	NO_SP	1.000074	0.000001	0.000000	0.000000	0.000000	4	20	**3976833,333**	**130063**	**30,58**	**0,03**
A0A100I423	Cell wall synthesis protein CwsA	11,8	**15,2**	142	1	NO_SP	1.000052	0.000001	0.000000	0.000000	0.000000	3	4	**3973833,333**	**523803**	**7,59**	**0,13**
A0A2Z5YPD8	Protein kinase domain-containing protein	5,5	**143,4**	1300	0	NO_SP	0.999939	0.000106	0.000003	0.000000	0.000000	1	22	**3932166,667**	**4592000**	**0,86**	**1,17**
B2HSU6	ESX-5 secretion system protein EccD5	9,4	**53,5**	503	10	NO_SP	1.000026	0.000003	0.000000	0.000000	0.000000	4	7	**3926500**	**194370**	**20,20**	**0,05**
A0A100I4B9	Transmembrane transport protein	9,3	**101,1**	952	10	NO_SP	0.915548	0.068178	0.009024	0.000711	0.000386	4	22	**3920800**	**148963**	**26,32**	**0,04**
A0A2Z5YCE3	Phthiotriol/phenolphthiotriol dimycocerosates methyltransferase	4,9	**27,0**	241	0	NO_SP	1.000070	0.000000	0.000000	0.000000	0.000000	4	9	**3898666,667**	**180937**	**21,55**	**0,05**
A0A2Z5YAL3	Neutral ceramidase	6,3	**68,8**	637	0	NO_SP	1.000043	0.000002	0.000000	0.000000	0.000000	4	20	**3824700**	**28417**	**134,59**	**0,01**
A0A2Z5YGC2	Chromosome partition protein Smc	5,0	**125,0**	1120	0	NO_SP	1.000043	0.000006	0.000000	0.000000	0.000000	3	36	**3822000**	**72505**	**52,71**	**0,02**
A0A2Z5YE99	DNA polymerase I	4,7	**98,1**	899	0	NO_SP	1.000027	0.000000	0.000000	0.000000	0.000000	4	29	**3815966,667**	**60473**	**63,10**	**0,02**
A0A2Z5YNZ0	Type VII secretion protein EccE	10,0	**48,5**	444	1	NO_SP	1.000086	0.000000	0.000000	0.000000	0.000000	3	13	**3812600**	**155420**	**24,53**	**0,04**
A0A2Z5YKR2	Ribosome-binding ATPase YchF	4,6	**39,3**	366	0	NO_SP	1.000055	0.000000	0.000000	0.000000	0.000000	4	13	**3792266,667**	**7686**	**493,39**	**0,00**
A0A100I690	GMP synthase [glutamine-hydrolyzing]	4,9	**56,2**	525	0	NO_SP	1.000056	0.000000	0.000000	0.000000	0.000000	4	17	**3786100**	**65461**	**57,84**	**0,02**
A0A2Z5YN99	Phosphoribosylamine--glycine ligase	4,9	**43,2**	422	0	NO_SP	1.000037	0.000014	0.000000	0.000000	0.000000	4	14	**3780400**	**50496**	**74,87**	**0,01**
A0A2Z5YFZ0	Protein translocase subunit SecA	4,6	**84,9**	778	0	NO_SP	1.000044	0.000000	0.000000	0.000000	0.000000	4	25	**3771733,333**	**45512**	**82,87**	**0,01**
A0A2Z5YG25	Proteasome accessory factor PafA2	5,3	**54,9**	502	0	NO_SP	1.000038	0.000002	0.000000	0.000000	0.000000	3	18	**3759833,333**	**23576**	**159,48**	**0,01**
A0A3E2MPC2	Mce related protein	5,2	**46,5**	434	1	NO_SP	1.000019	0.000003	0.000000	0.000000	0.000000	4	13	**3754800**	**152107**	**24,69**	**0,04**
A0A2Z5YCW9	Glutamyl-tRNA(Gln) amidotransferase subunit A	4,7	**51,3**	493	0	NO_SP	1.000031	0.000009	0.000000	0.000000	0.000000	4	12	**3745733,333**	**52623**	**71,18**	**0,01**
A0A3E2MYG7	Putative acetyl-CoA acetyltransferase	4,7	**40,5**	393	0	NO_SP	1.000059	0.000000	0.000000	0.000000	0.000000	3	17	**3744533,333**	**35149**	**106,53**	**0,01**
A0A2Z5YFT6	8-oxoguanine deaminase	4,8	**49,1**	462	0	NO_SP	0.998917	0.001011	0.000073	0.000002	0.000001	4	10	**3737733,333**	**75270**	**49,66**	**0,02**
A0A2Z5YN48	Putative thiosulfate sulfurtransferase 1	4,9	**30,9**	280	0	NO_SP	1.000039	0.000001	0.000000	0.000000	0.000000	3	10	**3735233,333**	**119848**	**31,17**	**0,03**
A0A2Z5YNJ0	Putative metallophosphoesterase	10,0	**36,4**	337	1	NO_SP	0.996330	0.003611	0.000030	0.000018	0.000009	3	14	**3727100**	**60226**	**61,89**	**0,02**
B2HF84	Citrate synthase (unknown stereospecificity)	5,3	**40,1**	373	0	NO_SP	1.000064	0.000001	0.000000	0.000000	0.000000	4	12	**3724866,667**	**81160**	**45,90**	**0,02**
A0A2Z5YJ26	2-oxoglutarate oxidoreductase subunit KorA	4,9	**69,2**	653	0	NO_SP	1.000053	0.000004	0.000000	0.000000	0.000000	4	22	**3724300**	**52906**	**70,39**	**0,01**
A0A2Z5YLF5	Putative L-ascorbate-6-phosphate lactonase UlaG	6,2	**40,5**	372	0	NO_SP	0.634295	0.360737	0.002201	0.001114	0.000625	3	10	**3718766,667**	**943880**	**3,94**	**0,25**
B2HJW1	Heat shock protein HspX_1	4,7	**15,9**	143	0	NO_SP	1.000061	0.000001	0.000000	0.000000	0.000000	5	7	**3710800**	**23908**	**155,21**	**0,01**
A0A2Z5YDT5	Quinone reductase	5,0	**34,4**	336	0	NO_SP	1.000064	0.000000	0.000000	0.000000	0.000000	3	11	**3696133,333**	**21926**	**168,57**	**0,01**
A0A100I5K9	Mammalian cell entry protein	5,0	**52,9**	507	1	SP	0.157723	0.841155	0.000333	0.000255	0.000240	4	12	**3684400**	**179093**	**20,57**	**0,05**
A0A2Z5YAD0	50S ribosomal protein L1	10,3	**24,8**	235	0	NO_SP	1.000057	0.000000	0.000000	0.000000	0.000000	4	11	**3675033,333**	**259446**	**14,16**	**0,07**
B2HHK6	Multimeric flavodoxin WrbA	5,0	**27,5**	250	0	NO_SP	1.000064	0.000000	0.000000	0.000000	0.000000	4	14	**3658166,667**	**48004**	**76,21**	**0,01**
A0A3E2MP52	Stearoyl-CoA 9-desaturase electron transfer partner	6,5	**38,8**	365	0	NO_SP	1.000032	0.000020	0.000000	0.000000	0.000000	5	14	**3647066,667**	**109885**	**33,19**	**0,03**
A0A2Z5YC33	Carrier domain-containing protein	4,5	**153,4**	1486	0	NO_SP	1.000028	0.000001	0.000000	0.000000	0.000000	1	9	**3639700**	**128929**	**28,23**	**0,04**
A0A2Z5YDW8	Glucose-6-phosphate 1-dehydrogenase	5,2	**57,7**	517	0	NO_SP	0.999773	0.000226	0.000002	0.000000	0.000000	4	24	**3635600**	**133590**	**27,21**	**0,04**
A0A2Z5YHU6	Cysteine synthase	4,7	**32,5**	310	0	NO_SP	1.000064	0.000000	0.000000	0.000000	0.000000	3	17	**3611200**	**48338**	**74,71**	**0,01**
A0A2Z5YN72	Diacylglycerol O-acyltransferase	6,9	**48,4**	450	0	NO_SP	1.000043	0.000003	0.000000	0.000000	0.000000	4	16	**3607700**	**26189**	**137,75**	**0,01**
B2HF47	Polyprenol-monophosphomannose synthase, Ppm1B	7,3	**29,4**	268	0	NO_SP	1.000066	0.000000	0.000000	0.000000	0.000000	4	11	**3538500**	**92571**	**38,22**	**0,03**
B2HMU3	MCE-family protein Mce1D	4,8	**57,2**	535	1	NO_SP	0.999943	0.000077	0.000001	0.000000	0.000000	4	21	**3517466,667**	**346007**	**10,17**	**0,10**
B2HEM8	D-alpha-D-mannose-1-phosphate guanylyltransferase ManB	5,2	**37,7**	358	0	NO_SP	1.000049	0.000000	0.000000	0.000000	0.000000	3	14	**3511733,333**	**26826**	**130,91**	**0,01**
A0A2Z5YKQ1	3-hydroxyisobutyryl-CoA hydrolase	4,5	**36,7**	349	0	NO_SP	1.000060	0.000000	0.000000	0.000000	0.000000	4	11	**3510466,667**	**33453**	**104,94**	**0,01**
A0A2Z5YLQ5	Putative enoyl-CoA hydratase echA8	5,0	**27,1**	257	0	NO_SP	1.000052	0.000000	0.000000	0.000000	0.000000	4	7	**3495466,667**	**36698**	**95,25**	**0,01**
B2HT53	Fructose-1,6-bisphosphatase	4,5	**37,8**	362	0	NO_SP	0.997647	0.002386	0.000004	0.000003	0.000002	4	14	**3471033,333**	**282478**	**12,29**	**0,08**
A0A2Z5YDQ3	Amino acid decarboxylase	5,7	**105,6**	943	0	NO_SP	0.957751	0.042070	0.000076	0.000049	0.000032	3	21	**3465733,333**	**95985**	**36,11**	**0,03**

A0A2Z5YHY7	Universal stress protein	4,9	**31,8**	298	0	NO_SP	1.000063	0.000000	0.000000	0.000000	0.000000	4	12	**3448533,333**	**36106**	**95,51**	**0,01**
A0A2Z5YHM1	F420-dependent oxidoreductase	6,1	**30,3**	276	0	NO_SP	1.000086	0.000000	0.000000	0.000000	0.000000	4	11	**3434000**	**26444**	**129,86**	**0,01**
B2HH29	Phosphoribosylformylglycinamidine cyclo-ligase	4,9	**38,7**	367	0	NO_SP	1.000027	0.000006	0.000000	0.000000	0.000000	4	15	**3424100**	**70031**	**48,89**	**0,02**
B2HNJ0	Glycerol-3-phosphate acyltransferase	8,9	**88,2**	788	0	NO_SP	1.000046	0.000000	0.000000	0.000000	0.000000	4	27	**3418033,333**	**99403**	**34,39**	**0,03**
B2HHM2	Glutamine synthetase	5,1	**49,7**	446	0	NO_SP	1.000039	0.000000	0.000000	0.000000	0.000000	3	15	**3404600**	**25409**	**133,99**	**0,01**
A0A2Z5YKU5	ATP synthase subunit b	4,6	**18,1**	170	1	NO_SP	1.000060	0.000011	0.000000	0.000000	0.000000	4	6	**3394733,333**	**190700**	**17,80**	**0,06**
A0A3E2MY80	Serine hydroxymethyltransferase	6,6	**49,8**	471	0	NO_SP	0.999142	0.000873	0.000014	0.000002	0.000001	4	12	**3372766,667**	**44166**	**76,37**	**0,01**
B2HQZ0	Integral membrane cytochrome D ubiquinol oxidase (Subunit I) CydA	8,4	**54,2**	485	9	NO_SP	1.000062	0.000000	0.000000	0.000000	0.000000	3	8	**3356300**	**69599**	**48,22**	**0,02**
A0A3E2MU78	Sporulation initiation inhibitor protein Soj	5,4	**31,2**	287	0	NO_SP	1.000039	0.000001	0.000000	0.000000	0.000000	4	13	**3348166,667**	**71737**	**46,67**	**0,02**
A0A2Z5YH42	Proteasome subunit alpha	4,7	**29,4**	267	0	NO_SP	0.998215	0.001737	0.000045	0.000002	0.000002	3	8	**3347633,333**	**65094**	**51,43**	**0,02**
A7BJ00	Chaperone protein DnaJ	7,9	**41,6**	396	0	NO_SP	1.000063	0.000000	0.000000	0.000000	0.000000	3	16	**3325533,333**	**18319**	**181,53**	**0,01**
A0A2Z5Y9W2	Histidine phosphatase family protein	6,7	**22,3**	202	0	NO_SP	1.000074	0.000000	0.000000	0.000000	0.000000	4	9	**3309133,333**	**2765033**	**1,20**	**0,84**
B2HDS6	Homoserine O-acetyltransferase	5,8	**40,2**	379	0	NO_SP	1.000052	0.000001	0.000000	0.000000	0.000000	4	14	**3304466,667**	**49798**	**66,36**	**0,02**
B2HJ65	Hypoxanthine phosphoribosyltransferase	4,5	**22,3**	202	0	NO_SP	0.999634	0.000394	0.000002	0.000000	0.000000	5	7	**3293866,667**	**81535**	**40,40**	**0,02**
A0A3E2MYS0	Methylenetetrahydromethanopterin reductase	6,1	**30,9**	286	0	NO_SP	0.999991	0.000045	0.000001	0.000000	0.000000	4	6	**3280400**	**27214**	**120,54**	**0,00**
A0A2Z5YM52	Aldehyde dehydrogenase	4,9	**54,9**	507	0	NO_SP	1.000048	0.000007	0.000000	0.000000	0.000000	3	10	**3279933,333**	**86342**	**37,99**	**0,03**
B2HJX0	Uncharacterized protein	6,3	**15,5**	139	0	NO_SP	1.000068	0.000000	0.000000	0.000000	0.000000	3	7	**3275900**	**3845**	**851,95**	**0,00**
A0A2Z5YNR7	ESX-1 secretion-associated protein EspB	4,3	**46,9**	454	0	NO_SP	1.000006	0.000036	0.000000	0.000000	0.000000	3	16	**3271433,333**	**72605**	**45,06**	**0,02**
B2HHJ7	Mycobactin synthetase protein B	6,3	**1070,3**	9858	0	NO_SP	1.000070	0.000000	0.000000	0.000000	0.000000	25	23	**3263833,333**	**157020**	**20,79**	**0,05**
A0A2Z5YB07	HAMP domain-containing protein	6,3	**96,1**	909	2	NO_SP	0.998036	0.001979	0.000000	0.000000	0.000000	3	23	**3241600**	**25950**	**124,92**	**0,01**
B2HNQ3	Conserved hypothetical alanine and proline rich membrane protein	5,3	**91,8**	879	0	NO_SP	1.000066	0.000000	0.000000	0.000000	0.000000	4	14	**3229033,333**	**108611**	**29,73**	**0,03**
A0A2Z5YGP6	Putative oxidoreductase EphD	9,3	**63,9**	594	1	NO_SP	1.000070	0.000000	0.000000	0.000000	0.000000	3	18	**3215933,333**	**87977**	**36,55**	**0,03**
A0A2Z5Y9V8	AAA_31 domain-containing protein	5,9	**47,1**	438	0	NO_SP	1.000056	0.000000	0.000000	0.000000	0.000000	4	13	**3209033,333**	**39504**	**81,23**	**0,01**
A0A117DTS6	Glycosyl hydrolase family 15	6,1	**67,0**	588	0	NO_SP	1.000049	0.000001	0.000000	0.000000	0.000000	4	21	**3203766,667**	**46189**	**69,36**	**0,01**
A0A100I0M9	Membrane protein	4,2	**25,3**	238	3	NO_SP	1.000044	0.000003	0.000000	0.000000	0.000000	3	4	**3195666,667**	**72747**	**43,93**	**0,02**
B2HQS0	Aldehyde dehydrogenase, PutA_1	4,7	**54,7**	507	0	NO_SP	1.000030	0.000024	0.000001	0.000000	0.000000	3	16	**3191466,667**	**57000**	**55,99**	**0,02**
A0A2Z5YBI2	Chromosome partitioning protein ParA	6,3	**28,3**	266	0	NO_SP	1.000043	0.000001	0.000000	0.000000	0.000000	3	8	**3176866,667**	**63381**	**50,12**	**0,02**
A0A3E2MS33	Putative decaprenylphosphoryl-beta-D-ribose oxidase	6,2	**50,2**	463	0	NO_SP	0.992500	0.007507	0.000031	0.000007	0.000004	4	16	**3168233,333**	**46951**	**67,48**	**0,01**
A0A2Z5Y9U7	Cyclopropane mycolic acid synthase	6,3	**32,8**	287	0	NO_SP	1.000041	0.000000	0.000000	0.000000	0.000000	4	16	**3161733,333**	**90813**	**34,82**	**0,03**
A0A2Z5Y8C9	Fatty acid desaturase	6,7	**42,9**	377	3	NO_SP	1.000042	0.000002	0.000000	0.000000	0.000000	3	9	**3156400**	**131811**	**23,95**	**0,04**
A0A2Z5YAA4	50S ribosomal protein L18	12,2	**14,6**	135	0	NO_SP	1.000048	0.000008	0.000000	0.000000	0.000000	3	3	**3154433,333**	**1254800**	**2,51**	**0,40**
A0A3E2MZP3	Acetolactate synthase	4,8	**64,2**	600	0	NO_SP	1.000084	0.000000	0.000000	0.000000	0.000000	3	16	**3151933,333**	**49479**	**63,70**	**0,02**
A0A3E2N045	ESX-1 secretion system protein EccA1	4,8	**64,9**	599	0	NO_SP	1.000046	0.000000	0.000000	0.000000	0.000000	4	17	**3141166,667**	**41436**	**75,81**	**0,01**
A0A2Z5YGG4	DUF4333 domain-containing protein	6,9	**10,9**	103	1	NO_SP	0.918201	0.080818	0.000248	0.000167	0.000111	4	6	**3135500**	**92127**	**34,03**	**0,03**
A0A2Z5YCC6	Universal stress protein	8,4	**27,8**	264	0	NO_SP	1.000047	0.000001	0.000000	0.000000	0.000000	2	10	**3130500**	**44343**	**70,60**	**0,01**
B2HRD2	Non-specific serine/threonine protein kinase	5,3	**63,6**	596	1	NO_SP	1.000060	0.000000	0.000000	0.000000	0.000000	4	16	**3114933,333**	**27406**	**113,66**	**0,01**
A0A2Z5YCV5	Mycothione reductase	5,4	**49,5**	459	0	NO_SP	1.000068	0.000001	0.000000	0.000000	0.000000	4	13	**3096533,333**	**20528**	**150,85**	**0,01**
A0A2Z5YBG4	Uncharacterized protein	4,8	**30,8**	281	0	NO_SP	1.000037	0.000001	0.000000	0.000000	0.000000	3	10	**3077800**	**64222**	**47,92**	**0,02**
A0A2Z5YDF8	Uncharacterized protein	5,1	**38,6**	356	0	NO_SP	1.000051	0.000000	0.000000	0.000000	0.000000	4	13	**3065033,333**	**16704**	**183,49**	**0,01**
A0A100I0G8	Serine--tRNA ligase	4,8	**45,3**	419	0	NO_SP	1.000037	0.000000	0.000000	0.000000	0.000000	4	12	**3050200**	**43430**	**70,23**	**0,01**
B2HMA7	Phosphoadenosine phosphosulfate reductase	4,5	**26,7**	249	0	NO_SP	0.999959	0.000104	0.000000	0.000000	0.000000	8	8	**3049333,333**	**62892**	**48,49**	**0,02**
A0A100I2Q3	Histidine kinase	5,9	**44,4**	411	1	NO_SP	1.000060	0.000000	0.000000	0.000000	0.000000	4	13	**3042733,333**	**117767**	**25,84**	**0,04**
A0A2Z5YDT2	Riboflavin biosynthesis protein RibBA	5,3	**46,0**	425	0	NO_SP	1.000037	0.000001	0.000000	0.000000	0.000000	3	14	**3012366,667**	**10511**	**286,59**	**0,00**
A0A2Z5Y7V2	Acyl-CoA dehydrogenase	6,1	**43,8**	403	0	NO_SP	1.000056	0.000000	0.000000	0.000000	0.000000	3	15	**3004800**	**19724**	**152,34**	**0,01**
B2HCZ2	Phosphoglucosamine mutase	4,6	**45,4**	445	0	NO_SP	1.000046	0.000002	0.000000	0.000000	0.000000	4	13	**2976700**	**138658**	**21,47**	**0,05**
A0A3E2MRX1	UDP-galactopyranose mutase	4,9	**46,0**	402	0	NO_SP	0.999805	0.000133	0.000100	0.000000	0.000000	4	10	**2970300**	**15815**	**187,82**	**0,01**
A0A2Z5YMC1	Phosphate-binding protein PstS	4,7	**33,8**	329	0	NO_SP	1.000038	0.000003	0.000000	0.000000	0.000000	4	10	**2968833,333**	**48251**	**61,53**	**0,02**
B2HGY0	Aminomethyltransferase	5,0	**38,6**	367	0	NO_SP	1.000013	0.000021	0.000000	0.000000	0.000000	3	6	**2957633,333**	**55316**	**53,47**	**0,02**
A0A2Z5YFC8	Putative acetolactate synthase	5,7	**58,9**	553	0	NO_SP	1.000033	0.000003	0.000000	0.000000	0.000000	4	17	**2951533,333**	**3108**	**949,63**	**0,00**
A0A2Z5YAC9	50S ribosomal protein L11	10,2	**15,0**	142	0	NO_SP	1.000071	0.000000	0.000000	0.000000	0.000000	4	5	**2944166,667**	**107231**	**27,46**	**0,04**
A0A2Z5YKU3	Glucose-6-phosphate isomerase	5,1	**60,3**	554	0	NO_SP	1.000065	0.000000	0.000000	0.000000	0.000000	4	17	**2941666,667**	**67983**	**43,27**	**0,02**
A0A2Z5Y9C0	Phosphate acetyltransferase	4,9	**73,8**	699	0	NO_SP	1.000048	0.000000	0.000000	0.000000	0.000000	4	21	**2936066,667**	**57202**	**51,33**	**0,02**
A0A3E2MTF6	50S ribosomal protein L33	10,6	**6,5**	55	0	NO_SP	1.000021	0.000000	0.000000	0.000000	0.000000	3	2	**2929653,333**	**420137**	**6,97**	**0,14**
A0A100I003	30S ribosomal protein S13	11,5	**12,7**	110	0	NO_SP	1.000060	0.000000	0.000000	0.000000	0.000000	3	7	**2925166,667**	**338660**	**8,64**	**0,12**
A0A2Z5YIW6	Acyl-CoA dehydrogenase	4,8	**42,5**	388	0	NO_SP	1.000072	0.000000	0.000000	0.000000	0.000000	4	14	**2918633,333**	**14193**	**205,64**	**0,00**
A0A2Z5YAM4	Glutamine--fructose-6-phosphate aminotransferase [isomerizing]	5,2	**68,7**	635	0	NO_SP	1.000058	0.000001	0.000000	0.000000	0.000000	3	21	**2915566,667**	**16836**	**173,17**	**0,01**
A0A2Z5YNM3	Putative arabinosyltransferase A	9,8	**116,2**	1094	12	SP	0.002013	0.997092	0.000233	0.000225	0.000194	4	14	**2907333,333**	**16862**	**172,42**	**0,01**
A0A2Z5YDQ8	Lipoarabinomannan carrier protein LprG	5,8	**24,6**	236	0	TATLIPO	0.000000	0.000001	0.020063	0.000010	0.979923	3	7	**2899966,667**	**16435**	**176,45**	**0,01**
B2HQX9	Tryptophan synthase alpha chain	4,6	**28,0**	269	0	NO_SP	1.000064	0.000001	0.000000	0.000000	0.000000	3	11	**2898933,333**	**38077**	**76,13**	**0,01**
A0A2Z5YNT0	Putative arabinosyltransferase B	9,7	**116,1**	1075	12	NO_SP	1.000039	0.000013	0.000000	0.000000	0.000000	4	17	**2895900**	**30718**	**94,27**	**0,01**
A0A2Z5Y8P3	Lysophospholipase	5,9	**30,0**	279	0	NO_SP	1.000069	0.000000	0.000000	0.000000	0.000000	3	13	**2888800**	**6646**	**434,68**	**0,00**
A0A2Z5YFW5	Nitrate ABC transporter substrate-binding protein	5,4	**66,4**	624	0	NO_SP	1.000089	0.000000	0.000000	0.000000	0.000000	3	19	**2880533,333**	**29066**	**99,10**	**0,01**
B2HLU7	NAD(P) transhydrogenase subunit beta	6,3	**48,4**	474	9	NO_SP	0.999994	0.000000	0.000000	0.000000	0.000000	4	10	**2879933,333**	**59164**	**48,68**	**0,02**
A0A2Z5YHE9	Diacylglycerol O-acyltransferase	6,2	**49,6**	463	0	NO_SP	1.000054	0.000000	0.000000	0.000000	0.000000	5	10	**2878233,333**	**190928**	**15,07**	**0,07**
B2HIS7	Dihydroorotate dehydrogenase	5,4	**36,2**	336	0	NO_SP	1.000063	0.000000	0.000000	0.000000	0.000000	4	14	**2875533,333**	**335370**	**8,57**	**0,12**
A0A2Z5YJG4	Arginine--tRNA ligase	5,2	**59,7**	550	0	NO_SP	1.000071	0.000000	0.000000	0.000000	0.000000	4	22	**2872233,333**	**28898**	**99,39**	**0,01**
A0A2Z5YAK4	50S ribosomal protein L4	10,8	**23,8**	224	0	NO_SP	1.000057	0.000001	0.000000	0.000000	0.000000	3	7	**2866900**	**193303**	**14,83**	**0,07**
A0A2Z5Y981	Glycolipid sulfotransferase	6,1	**36,5**	324	0	NO_SP	1.000072	0.000000	0.000000	0.000000	0.000000	8	15	**2862800**	**461957**	**6,20**	**0,16**
A0A100IFA3	Formaldehyde dehydrogenase	4,4	**35,8**	342	0	NO_SP	1.000038	0.000016	0.000000	0.000000	0.000000	4	15	**2861833,333**	**13023**	**219,76**	**0,00**
B2HP49	Coenzyme A biosynthesis bifunctional protein CoaBC	6,8	**43,4**	413	0	NO_SP	1.000046	0.000001	0.000000	0.000000	0.000000	4	14	**2845333,333**	**23496**	**121,10**	**0,01**
B2HQ06	Conserved hypothetical membrane protein	7,8	**11,5**	110	2	NO_SP	1.000065	0.000011	0.000000	0.000000	0.000000	4	3	**2838333,333**	**153060**	**18,54**	**0,05**
A0A2Z5YDG6	AMP-dependent synthetase	5,5	**67,4**	621	0	NO_SP	0.995965	0.003866	0.000127	0.000013	0.000006	5	18	**2818166,667**	**71027**	**39,68**	**0,03**
A0A2Z5YE48	3-dehydroquinate synthase	4,9	**39,8**	362	0	NO_SP	1.000075	0.000000	0.000000	0.000000	0.000000	4	12	**2812433,333**	**46279**	**60,77**	**0,02**
A0A2Z5Y835	Ketoacyl reductase	7,4	**27,6**	267	0	NO_SP	1.000045	0.000000	0.000000	0.000000	0.000000	4	10	**2812300**	**28837**	**97,52**	**0,01**
B2HSU8	ESX-5 secretion system protein EccE5	6,0	**44,0**	403	2	NO_SP	1.000011	0.000001	0.000000	0.000000	0.000000	4	9	**2807433,333**	**108658**	**25,84**	**0,04**
A0A3E2MYV6	Glutamate decarboxylase	5,0	**51,1**	461	0	NO_SP	0.999969	0.000083	0.000000	0.000000	0.000000	4	19	**2804133,333**	**9626**	**291,30**	**0,00**
A0A3E2N170	Glucose-6-phosphate 1-dehydrogenase	5,6	**52,8**	469	0	NO_SP	1.000034	0.000000	0.000000	0.000000	0.000000	4	15	**2803733,333**	**15706**	**178,51**	**0,01**

A0A2Z5YJ38	Phosphoriboisomerase B	5,5	**17,0**	159	0	NO_SP	1.000078	0.000001	0.000000	0.000000	0.000000	4	10	**2803333,333**	**81465**	**34,41**	**0,03**
A0A2Z5YCZ3	4-hydroxy-tetrahydrodipicolinate reductase	5,3	**25,1**	240	0	NO_SP	1.000020	0.000019	0.000000	0.000000	0.000000	4	6	**2794966,667**	**12926**	**216,22**	**0,00**
A0A2Z5YH04	Iron-sulfur cluster insertion protein ErpA	4,0	**12,5**	118	0	NO_SP	1.000058	0.000010	0.000000	0.000000	0.000000	3	5	**2793366,667**	**78227**	**35,71**	**0,03**
B2HHK8	Oxaloacetate decarboxylase	4,8	**23,3**	219	0	NO_SP	1.000054	0.000000	0.000000	0.000000	0.000000	3	10	**2785533,333**	**9193**	**302,99**	**0,00**
A0A2Z5YNR3	Type VII secretion protein EccCa	8,0	**80,9**	746	2	NO_SP	0.999981	0.000064	0.000000	0.000000	0.000000	5	20	**2769266,667**	**109209**	**25,36**	**0,04**
B2HST3	ESX-5 secretion system ATPase EccB5	6,4	**54,1**	507	1	NO_SP	0.998409	0.001572	0.000007	0.000001	0.000001	4	9	**2755933,333**	**21431**	**128,60**	**0,01**
A0A3E2MMQ6	Putative copper-exporting P-type ATPase V	6,2	**77,8**	732	0	NO_SP	1.000038	0.000006	0.000000	0.000000	0.000000	5	19	**2749766,667**	**130584**	**21,06**	**0,05**
A0A2Z5YCY4	Signal recognition particle receptor FtsY	4,5	**46,6**	448	1	NO_SP	1.000087	0.000000	0.000000	0.000000	0.000000	4	12	**2742366,667**	**8201**	**334,39**	**0,00**
B2HQL4	50S ribosomal protein L31	9,6	**8,9**	81	0	NO_SP	1.000057	0.000000	0.000000	0.000000	0.000000	3	4	**2736120**	**37315**	**73,32**	**0,01**
A0A2Z5Y9Q5	Fatty acyl-CoA reductase	7,7	**33,3**	312	0	NO_SP	1.000051	0.000000	0.000000	0.000000	0.000000	3	15	**2735133,333**	**59911**	**45,65**	**0,02**
A0A2Z5YDX7	Carbamoyl-phosphate synthase small chain	5,6	**38,1**	359	0	NO_SP	1.000058	0.000005	0.000000	0.000000	0.000000	4	10	**2731866,667**	**16876**	**161,88**	**0,01**
A0A2Z5YE83	Ketoacyl reductase	7,2	**28,2**	267	0	NO_SP	1.000047	0.000000	0.000000	0.000000	0.000000	3	12	**2709766,667**	**14894**	**181,94**	**0,01**
A0A2Z5YKQ5	Diaminopimelate decarboxylase	5,0	**50,4**	472	0	NO_SP	0.991564	0.008129	0.000218	0.000031	0.000014	3	16	**2699966,667**	**39974**	**67,54**	**0,01**
A0A2Z5YGI2	ATP phosphoribosyltransferase	4,9	**30,6**	284	0	NO_SP	1.000055	0.000001	0.000000	0.000000	0.000000	3	13	**2689466,667**	**27080**	**99,31**	**0,01**
A0A100IBA5	Aminotransferase	4,7	**51,6**	486	0	NO_SP	1.000055	0.000010	0.000000	0.000000	0.000000	3	14	**2684000**	**1174**	**2286,66**	**0,00**
A0A100IFY9	Alanine and proline rich protein	5,8	**75,8**	738	0	NO_SP	1.000070	0.000002	0.000000	0.000000	0.000000	4	13	**2683433,333**	**61387**	**43,71**	**0,02**
B2HDV2	Probable allantoicase	5,7	**35,0**	319	0	NO_SP	1.000042	0.000001	0.000000	0.000000	0.000000	4	12	**2662800**	**56849**	**46,84**	**0,02**
A0A2Z5Y8F1	Acyl-CoA dehydrogenase	6,3	**45,1**	410	0	NO_SP	1.000050	0.000000	0.000000	0.000000	0.000000	4	11	**2662466,667**	**26571**	**100,20**	**0,01**
A0A117DY41	Cysteine desulfurase	5,8	**41,2**	391	0	NO_SP	1.000047	0.000002	0.000000	0.000000	0.000000	3	9	**2658600**	**87837**	**30,27**	**0,03**
A0A3E2MZY6	Carnitinyl-CoA dehydratase	5,2	**27,1**	260	0	NO_SP	1.000044	0.000003	0.000000	0.000000	0.000000	4	10	**2652000**	**69412**	**38,21**	**0,03**
B2HCZ0	50S ribosomal protein L13	10,5	**16,2**	147	0	NO_SP	1.000053	0.000001	0.000000	0.000000	0.000000	4	7	**2649600**	**185253**	**14,30**	**0,07**
A0A2Z5YAK8	3-hydroxyacyl-thioester dehydratase HtdY	5,2	**34,8**	327	0	NO_SP	0.987126	0.012261	0.000504	0.000036	0.000021	1	10	**2644733,333**	**3809**	**694,40**	**0,00**
A0A2Z5YFE5	Glycogen accumulation regulator GarA	4,1	**17,2**	162	0	NO_SP	1.000037	0.000002	0.000000	0.000000	0.000000	3	11	**2637033,333**	**0**	**#JAKO/0!**	**0,00**
A0A2Z5YIM7	Pyruvate-flavodoxin oxidoreductase	5,8	**128,8**	1193	0	NO_SP	1.000014	0.000009	0.000000	0.000000	0.000000	4	29	**2636733,333**	**3742**	**704,58**	**0,00**
B2HF73	FAD_binding_2 domain-containing protein	6,8	**60,6**	556	0	NO_SP	1.000011	0.000034	0.000003	0.000000	0.000000	4	18	**2618400**	**31994**	**81,84**	**0,01**
B2HK75	Conserved protein with endoribonuclease L-PSP domain	4,8	**15,2**	151	0	NO_SP	1.000033	0.000005	0.000000	0.000000	0.000000	4	6	**2607366,667**	**40956**	**63,66**	**0,02**
A0A2Z5YIF6	Valine--tRNA ligase	4,7	**98,3**	886	0	NO_SP	1.000055	0.000002	0.000000	0.000000	0.000000	3	24	**2599766,667**	**67970**	**38,25**	**0,03**
A0A2Z5Y7F0	Anthranilate synthase component 2	6,2	**24,5**	228	0	NO_SP	1.000055	0.000000	0.000000	0.000000	0.000000	3	8	**2591533,333**	**1397**	**1855,65**	**0,00**
B2HSN8	50S ribosomal protein L16	11,7	**15,7**	138	0	NO_SP	0.999863	0.000159	0.000006	0.000000	0.000000	3	4	**2590900**	**284020**	**9,12**	**0,11**
B2HP96	OXPP cycle protein OpcA	4,7	**32,7**	303	0	NO_SP	1.000071	0.000000	0.000000	0.000000	0.000000	4	12	**2588033,333**	**0**	**#JAKO/0!**	**0,00**
A0A2Z5YJG3	TPM_phosphatase domain-containing protein	5,4	**69,7**	662	2	SP	0.001963	0.996852	0.000318	0.000311	0.000260	4	19	**2558133,333**	**14975**	**170,83**	**0,01**
B2HF07	3-(3-hydroxy-phenyl) propionate hydroxylase	8,8	**57,2**	514	1	NO_SP	1.000051	0.000011	0.000000	0.000000	0.000000	2	1	**2554966,667**	**3544233**	**0,72**	**1,39**
A0A2Z5YCX8	Uncharacterized protein	7,4	**49,5**	450	0	NO_SP	0.955711	0.043884	0.000162	0.000086	0.000057	3	15	**2547400**	**39812**	**63,99**	**0,02**
B2HGG3	NADPH quinone oxidoreductase FadB4	5,2	**33,3**	323	0	NO_SP	1.000062	0.000004	0.000000	0.000000	0.000000	4	8	**2541966,667**	**40609**	**62,60**	**0,02**
A0A100I7F0	Cytochrome bc1 complex cytochrome c subunit	6,2	**24,9**	240	1	NO_SP	0.917474	0.082079	0.000149	0.000133	0.000081	4	4	**2534166,667**	**2629**	**963,94**	**0,00**
B2HJK1	Nitrogen regulatory protein P-II GlnB	5,9	**12,2**	112	0	NO_SP	1.000064	0.000000	0.000000	0.000000	0.000000	4	6	**2524133,333**	**17846**	**141,44**	**0,01**
A0A2Z5YCS0	Thioesterase TesA	4,9	**27,6**	249	0	NO_SP	1.000039	0.000000	0.000000	0.000000	0.000000	3	10	**2513366,667**	**27099**	**92,75**	**0,01**
A0A2Z5YBY6	3-isopropylmalate dehydrogenase	5,1	**36,1**	339	0	NO_SP	1.000052	0.000001	0.000000	0.000000	0.000000	3	8	**2511033,333**	**17383**	**144,46**	**0,01**
A0A2Z5YHT0	Uncharacterized protein	4,8	**56,2**	520	0	NO_SP	1.000043	0.000001	0.000000	0.000000	0.000000	4	17	**2510566,667**	**55276**	**45,42**	**0,02**
A0A2Z5YN23	DNA polymerase III subunit gamma/tau	5,3	**66,4**	621	0	NO_SP	1.000065	0.000001	0.000000	0.000000	0.000000	3	17	**2507033,333**	**7076**	**354,30**	**0,00**
A0A2Z5YAQ5	Putative glycosyl hydrolase	5,5	**88,4**	794	0	NO_SP	1.000088	0.000001	0.000000	0.000000	0.000000	4	22	**2505866,667**	**40058**	**62,56**	**0,02**
B2HMI8	Short-chain type dehydrogenase/reductase	7,4	**27,3**	254	0	NO_SP	1.000059	0.000000	0.000000	0.000000	0.000000	3	11	**2494366,667**	**84322**	**29,58**	**0,03**
A0A2Z5YI46	PhoH-like protein	6,4	**37,8**	352	0	NO_SP	0.999911	0.000137	0.000000	0.000000	0.000000	4	11	**2489966,667**	**19464**	**127,92**	**0,00**
B2HQX5	Anthranilate synthase component 1	4,6	**55,4**	512	0	NO_SP	0.999988	0.000044	0.000002	0.000000	0.000000	4	13	**2487766,667**	**26923**	**92,40**	**0,01**
B2HLY8	Iron-dependent repressor and activator IdeR	5,2	**25,3**	230	0	NO_SP	1.000077	0.000000	0.000000	0.000000	0.000000	3	8	**2478800**	**16551**	**149,76**	**0,01**
B2HEF7	Non-specific serine/threonine protein kinase	6,4	**73,4**	698	1	NO_SP	1.000050	0.000000	0.000000	0.000000	0.000000	4	15	**2477900**	**25991**	**95,34**	**0,01**
B2HFS9	Conserved protein	9,9	**14,9**	136	0	NO_SP	1.000029	0.000000	0.000000	0.000000	0.000000	3	8	**2473400**	**88819**	**27,85**	**0,04**
A0A2Z5YCX2	Transcription termination/antitermination protein NusA	6,3	**37,9**	347	0	NO_SP	1.000054	0.000000	0.000000	0.000000	0.000000	3	12	**2472833,333**	**26609**	**92,93**	**0,01**
A0A2Z5YBT1	LLM class F420-dependent oxidoreductase	4,5	**29,6**	275	0	NO_SP	1.000045	0.000001	0.000000	0.000000	0.000000	3	11	**2465866,667**	**18304**	**134,71**	**0,01**
B2HGW1	Cytochrome aa3 subunit 3	9,5	**22,4**	203	5	NO_SP	1.000043	0.000002	0.000000	0.000000	0.000000	1	1	**2464833,333**	**129177**	**19,08**	**0,05**
A0A117DTZ4	Antigen 85-C	5,0	**33,4**	312	0	NO_SP	1.000051	0.000000	0.000000	0.000000	0.000000	3	6	**2454100**	**17058**	**143,87**	**0,01**
B2HHU8	Conserved hypothetical oxidoreductase	4,5	**41,9**	388	0	NO_SP	1.000034	0.000008	0.000000	0.000000	0.000000	4	14	**2428366,667**	**22467**	**108,09**	**0,01**
B2HI46	Chromosomal replication initiator protein DnaA	5,3	**56,6**	510	0	NO_SP	1.000033	0.000007	0.000000	0.000000	0.000000	5	17	**2426200**	**10886**	**222,87**	**0,00**
A0A2Z5YJV6	Glycogen phosphorylase	5,3	**96,3**	867	0	NO_SP	1.000046	0.000001	0.000000	0.000000	0.000000	4	21	**2419733,333**	**30805**	**78,55**	**0,01**
B2HLR1	Zinc-dependent alcohol dehydrogenase	4,6	**36,4**	347	0	NO_SP	1.000041	0.000000	0.000000	0.000000	0.000000	4	14	**2415033,333**	**9411**	**256,62**	**0,00**
A0A2Z5Y9H5	ATPase	4,6	**75,2**	724	0	NO_SP	0.999930	0.000120	0.000002	0.000000	0.000000	4	20	**2408800**	**110778**	**21,74**	**0,05**
A0A2Z5YJI1	Thioredoxin domain-containing protein	4,3	**33,2**	314	0	NO_SP	1.000047	0.000001	0.000000	0.000000	0.000000	4	11	**2406400**	**22526**	**106,83**	**0,01**
B2HNC6	Chorismate synthase	6,0	**42,5**	407	0	NO_SP	1.000021	0.000027	0.000003	0.000000	0.000000	4	17	**2388133,333**	**39456**	**60,53**	**0,02**
A0A2Z5YHS2	Glutamine synthetase	5,0	**78,5**	725	0	NO_SP	1.000061	0.000000	0.000000	0.000000	0.000000	4	16	**2388066,667**	**121520**	**19,65**	**0,05**
A0A2Z5Y7K7	Leucine--tRNA ligase	4,9	**109,9**	1003	0	NO_SP	0.999003	0.000904	0.000083	0.000009	0.000003	4	19	**2385766,667**	**42873**	**55,65**	**0,02**
A0A2Z5YB08	Putative thiosulfate sulfurtransferase SseA	5,0	**33,5**	301	0	NO_SP	1.000055	0.000001	0.000000	0.000000	0.000000	4	12	**2385133,333**	**40616**	**58,72**	**0,02**
B2HIH4	Glutamate--tRNA ligase	5,1	**53,7**	489	0	NO_SP	1.000048	0.000001	0.000000	0.000000	0.000000	2	15	**2380500**	**22770**	**104,55**	**0,01**
A0A2Z5YG14	Putative oxidoreductase	5,4	**49,4**	475	0	NO_SP	1.000078	0.000000	0.000000	0.000000	0.000000	4	19	**2376900**	**490000**	**4,85**	**0,21**
B2HRH2	Short-chain type dehydrogenase/reductase	8,8	**29,1**	277	0	NO_SP	1.000049	0.000000	0.000000	0.000000	0.000000	7	10	**2376600**	**121542**	**19,55**	**0,05**
A0A3E2N089	N-acetylglucosamine repressor	10,4	**45,4**	433	0	NO_SP	0.999674	0.000355	0.000006	0.000001	0.000000	4	15	**2366900**	**52894**	**44,75**	**0,02**
A0A2Z5YMB2	Adenylosuccinate lyase	6,2	**51,2**	475	0	NO_SP	1.000047	0.000000	0.000000	0.000000	0.000000	4	16	**2366466,667**	**60126**	**39,36**	**0,03**
B2HKR4	Uroporphyrinogen III methylase	6,6	**42,0**	401	0	NO_SP	1.000064	0.000000	0.000000	0.000000	0.000000	4	14	**2363166,667**	**19187**	**123,16**	**0,01**
A0A2Z5Y8C3	Quinone oxidoreductase	5,4	**33,4**	322	0	NO_SP	1.000071	0.000000	0.000000	0.000000	0.000000	4	10	**2362800**	**14709**	**160,64**	**0,01**
A0A2Z5YN85	Penicillin-insensitive transglycosylase	5,3	**84,4**	803	1	NO_SP	0.966744	0.005773	0.000603	0.000048	0.000024	3	17	**2351433,333**	**18417**	**127,68**	**0,01**
A0A2Z5Y9G7	Peptidase	4,6	**77,4**	698	0	NO_SP	1.000056	0.000003	0.000000	0.000000	0.000000	4	14	**2338333,333**	**60671**	**38,54**	**0,03**
A0A2Z5YDE4	Threonine--tRNA ligase	5,2	**77,2**	691	0	NO_SP	0.978451	0.021404	0.000065	0.000041	0.000020	4	16	**2326466,667**	**34044**	**68,34**	**0,01**
B2HMG3	50S ribosomal protein L21	10,6	**11,0**	103	0	NO_SP	1.000078	0.000000	0.000000	0.000000	0.000000	3	6	**2322700**	**267910**	**8,67**	**0,12**
A0A2Z5YK83	ATPase AAA	5,2	**73,3**	694	0	NO_SP	1.000053	0.000000	0.000000	0.000000	0.000000	2	21	**2319933,333**	**51411**	**45,12**	**0,02**
B2HJX9	Nitroreductase domain-containing protein	6,2	**35,7**	333	0	NO_SP	1.000028	0.000011	0.000000	0.000000	0.000000	4	11	**2318366,667**	**35137**	**65,98**	**0,02**
B2HHZ6	Acyl-CoA dehydrogenase FadE26	5,7	**43,1**	392	0	NO_SP	1.000085	0.000000	0.000000	0.000000	0.000000	4	12	**2316966,667**	**9854**	**235,12**	**0,00**
A0A2Z5YML2	Acetyl-CoA acetyltransferase	4,9	**40,8**	386	0	NO_SP	0.999997	0.000045	0.000000	0.000000	0.000000	4	14	**2315033,333**	**4466**	**518,40**	**0,00**
A0A2Z5Y933	GH16 domain-containing protein	4,3	**24,7**	218	0	NO_SP	1.000040	0.000000	0.000000	0.000000	0.000000	4	5	**2311566,667**	**44853**	**51,54**	**0,02**

B2HGV2	Conserved protein	4,4	**16,2**	144	0	NO_SP	1.000060	0.000000	0.000000	0.000000	0.000000	3	8	**2307966,667**	**16529**	**139,63**	**0,01**
A0A2Z5Y9I1	NADH dehydrogenase NdhA	8,6	**50,8**	471	1	NO_SP	1.000066	0.000004	0.000000	0.000000	0.000000	4	17	**2285466,667**	**56257**	**40,63**	**0,02**
A0A2Z5YNA2	Uncharacterized protein	8,9	**15,3**	145	0	NO_SP	1.000032	0.000014	0.000000	0.000000	0.000000	4	8	**2281866,667**	**11062**	**206,28**	**0,00**
B2HQP4	Phosphatidylserine decarboxylase proenzyme	10,6	**25,2**	240	0	NO_SP	0.990153	0.007113	0.002353	0.000137	0.000098	4	11	**2277833,333**	**73266**	**31,09**	**0,03**
A0A2Z5YDN3	Uncharacterized protein	5,1	**96,4**	891	0	NO_SP	1.000035	0.000015	0.000000	0.000000	0.000000	5	22	**2277700**	**13082**	**174,11**	**0,01**
B2HLK1	Enoyl-CoA hydratase, EchA21	4,8	**29,0**	274	0	NO_SP	1.000020	0.000000	0.000000	0.000000	0.000000	4	10	**2265900**	**28873**	**78,48**	**0,01**
A0A2Z5YKR7	Methionine--tRNA ligase	5,2	**58,0**	518	0	NO_SP	1.000029	0.000021	0.000000	0.000000	0.000000	4	16	**2264500**	**5319**	**425,74**	**0,00**
A0A2Z5Y7R9	Uncharacterized protein	4,5	**26,8**	252	0	NO_SP	1.000058	0.000002	0.000000	0.000000	0.000000	4	10	**2261000**	**2591**	**872,75**	**0,00**
B2HEX1	Oxidoreductase	9,5	**33,9**	323	0	NO_SP	1.000072	0.000000	0.000000	0.000000	0.000000	4	8	**2258556,667**	**25514**	**88,52**	**0,01**
B2HT03	Acetyl-CoA acetyltransferase FadA6_3	5,0	**39,9**	382	0	NO_SP	1.000031	0.000003	0.000000	0.000000	0.000000	4	12	**2257700**	**4489**	**502,98**	**0,00**
A0A2Z5YAT2	Tryptophan--tRNA ligase	5,6	**36,6**	339	0	NO_SP	0.999484	0.000533	0.000007	0.000001	0.000001	3	10	**2245500**	**2309**	**972,44**	**0,00**
B2HNT0	Hydrolase	6,3	**44,1**	406	0	NO_SP	1.000056	0.000001	0.000000	0.000000	0.000000	4	11	**2245033,333**	**31916**	**70,34**	**0,01**
A0A2Z5YPD6	ParB domain-containing protein	6,0	**30,8**	284	0	NO_SP	1.000004	0.000031	0.000000	0.000000	0.000000	1	13	**2242066,667**	**173**	**12935,00**	**0,00**
A0A100I0J2	Integral membrane indolylacetylinositol arabinosyltransferase	9,8	**115,0**	1073	14	NO_SP	0.681156	0.317671	0.000297	0.000317	0.000209	5	12	**2239566,667**	**41480**	**53,99**	**0,02**
A0A2Z5YG19	Cytochrome P450	5,0	**46,9**	424	0	NO_SP	1.000064	0.000000	0.000000	0.000000	0.000000	4	10	**2228766,667**	**50625**	**44,02**	**0,02**
B2HPT1	Conserved secreted protein	10,4	**32,2**	284	1	NO_SP	1.000080	0.000000	0.000000	0.000000	0.000000	3	8	**2219433,333**	**61139**	**36,30**	**0,03**
A0A100I473	Chromosome (Plasmid) partitioning protein	5,0	**31,3**	291	0	NO_SP	1.000069	0.000000	0.000000	0.000000	0.000000	4	12	**2217566,667**	**11330**	**195,73**	**0,00**
A0A2Z5YCV2	1-deoxy-D-xylulose-5-phosphate synthase	6,0	**67,7**	637	0	NO_SP	1.000055	0.000000	0.000000	0.000000	0.000000	4	18	**2216766,667**	**20886**	**106,13**	**0,01**
A0A2Z5YAU8	30S ribosomal protein S9	10,5	**16,9**	152	0	NO_SP	1.000084	0.000000	0.000000	0.000000	0.000000	3	7	**2214233,333**	**242493**	**9,13**	**0,11**
A0A2Z5Y7L4	Penicillin-insensitive transglycosylase	6,2	**86,7**	823	1	NO_SP	1.000052	0.000000	0.000000	0.000000	0.000000	3	19	**2207000**	**25296**	**87,25**	**0,01**
A0A2Z5YEW3	Isoleucine--tRNA ligase	4,9	**119,3**	1066	0	NO_SP	1.000063	0.000000	0.000000	0.000000	0.000000	4	22	**2203366,667**	**24549**	**89,76**	**0,01**
A0A2Z5YIL4	Enoyl reductase	7,8	**44,5**	418	0	NO_SP	1.000075	0.000000	0.000000	0.000000	0.000000	4	13	**2201400**	**53198**	**41,38**	**0,02**
A0A2Z5YMQ6	Cysteine--tRNA ligase	5,7	**50,1**	452	0	NO_SP	1.000070	0.000000	0.000000	0.000000	0.000000	4	15	**2200900**	**24868**	**88,50**	**0,01**
A0A2Z5YDX4	DNA polymerase III subunit alpha	5,6	**128,2**	1171	0	NO_SP	1.000044	0.000001	0.000000	0.000000	0.000000	4	24	**2199033,333**	**41289**	**53,26**	**0,02**
B2HQ76	Fatty-acid-CoA ligase, FadD11	4,6	**65,4**	608	0	NO_SP	1.000056	0.000002	0.000000	0.000000	0.000000	5	7	**2198466,667**	**51622**	**42,59**	**0,02**
B2HM79	Chaperone protein DnaJ	6,7	**40,0**	378	0	NO_SP	1.000068	0.000000	0.000000	0.000000	0.000000	4	13	**2191000**	**28672**	**76,42**	**0,01**
A0A2Z5YGI0	Oxidoreductase	7,9	**31,1**	293	0	NO_SP	1.000072	0.000000	0.000000	0.000000	0.000000	4	12	**2188133,333**	**28145**	**77,75**	**0,01**
A0A2Z5Y8B3	Maltokinase	4,6	**49,3**	455	0	NO_SP	1.000059	0.000001	0.000000	0.000000	0.000000	4	12	**2156866,667**	**10838**	**199,01**	**0,00**
A0A2Z5YNY9	Putative coenzyme F420-dependent oxidoreductase	4,8	**37,3**	345	0	NO_SP	0.999653	0.000375	0.000015	0.000001	0.000000	4	12	**2134600**	**27404**	**77,89**	**0,01**
B2HQ16	UDP-glucose 4-epimerase, GalE5	5,1	**37,7**	339	0	NO_SP	1.000057	0.000000	0.000000	0.000000	0.000000	3	9	**2119876,667**	**21144**	**100,26**	**0,01**
A0A2Z5YCU2	Potassium transporter TrkA	4,8	**22,7**	213	0	NO_SP	1.000055	0.000001	0.000000	0.000000	0.000000	4	7	**2099233,333**	**10137**	**207,09**	**0,00**
A0A2Z5YNU7	DNA integrity scanning protein DisA	4,9	**39,1**	358	0	NO_SP	1.000062	0.000000	0.000000	0.000000	0.000000	5	10	**2090533,333**	**20050**	**104,26**	**0,01**
A0A2Z5YHG6	Carboxylesterase A	6,6	**55,8**	518	1	LIPO	0.000045	0.000491	0.999479	0.000007	0.000004	3	13	**2083966,667**	**10985**	**189,71**	**0,01**
B2HIB9	30S ribosomal protein S18 1	11,3	**9,5**	84	0	NO_SP	1.000024	0.000000	0.000000	0.000000	0.000000	3	2	**2077233,333**	**613220**	**3,39**	**0,30**
A0A100IF16	Nucleoid-associated protein Lsr2	10,6	**11,2**	105	0	NO_SP	1.000044	0.000000	0.000000	0.000000	0.000000	3	4	**2074400**	**823710**	**2,52**	**0,40**
B2HKB0	Methanol dehydrogenase transcriptional regulatory protein MoxR2	5,7	**34,6**	325	0	NO_SP	1.000040	0.000016	0.000000	0.000000	0.000000	4	11	**2066533,333**	**70125**	**29,47**	**0,03**
B2HPW4	Adenylosuccinate synthetase	5,5	**46,7**	432	0	NO_SP	1.000035	0.000005	0.000000	0.000000	0.000000	4	13	**2057666,667**	**14102**	**145,91**	**0,01**
A0A2Z5Y8V8	Type VII secretion protein EccE	7,2	**36,7**	337	2	NO_SP	1.000010	0.000002	0.000000	0.000000	0.000000	4	7	**2056533,333**	**2621**	**784,58**	**0,00**
A0A2Z5YAB8	Enoyl-CoA hydratase	5,6	**24,4**	231	0	NO_SP	1.000045	0.000000	0.000000	0.000000	0.000000	4	9	**2047300**	**17483**	**117,10**	**0,01**
A0A2Z5YAQ8	30S ribosomal protein S17	11,1	**13,1**	119	0	NO_SP	1.000041	0.000000	0.000000	0.000000	0.000000	4	6	**2045033,333**	**693347**	**2,95**	**0,34**
B2HR07	UvrABC system protein A	6,5	**106,3**	971	0	NO_SP	1.000063	0.000000	0.000000	0.000000	0.000000	3	22	**2042433,333**	**28417**	**71,87**	**0,01**
B2HSP5	Conserved short-chain dehydrogenase	9,8	**26,9**	265	0	NO_SP	1.000071	0.000000	0.000000	0.000000	0.000000	3	7	**2039233,333**	**43703**	**46,66**	**0,02**
A0A2Z5YEM3	Tyrosine--tRNA ligase	5,8	**44,8**	411	0	NO_SP	1.000062	0.000001	0.000000	0.000000	0.000000	2	11	**2037733,333**	**37220**	**54,75**	**0,02**
A0A2Z5Y992	Biotin carboxylase	4,7	**46,2**	446	0	NO_SP	1.000071	0.000000	0.000000	0.000000	0.000000	4	13	**2031300**	**38138**	**53,26**	**0,02**
A0A2Z5YDV8	DUF58 domain-containing protein	9,8	**35,2**	324	0	NO_SP	1.000047	0.000000	0.000000	0.000000	0.000000	3	10	**2020033,333**	**77845**	**25,95**	**0,04**
A0A3E2MRH8	Cell wall synthesis protein Wag31	4,5	**26,9**	245	0	NO_SP	1.000054	0.000000	0.000000	0.000000	0.000000	4	11	**2012700**	**11708**	**171,90**	**0,01**
B2HCW3	Methylmalonate semialdehyde dehydrogenase, MmsA	5,1	**54,3**	506	0	NO_SP	0.998523	0.000948	0.000347	0.000018	0.000006	4	15	**2010433,333**	**5066**	**396,88**	**0,00**
A0A2Z5YF02	Uncharacterized protein	4,4	**14,2**	123	0	NO_SP	1.000049	0.000000	0.000000	0.000000	0.000000	3	5	**2008900**	**85257**	**23,56**	**0,04**
A0A2Z5YE61	Phenylalanine--tRNA ligase alpha subunit	5,1	**38,2**	347	0	NO_SP	1.000047	0.000000	0.000000	0.000000	0.000000	4	9	**2008766,667**	**9047**	**222,03**	**0,00**
A0A2Z5YNZ6	ESX-1 secretion system protein EccCb1	6,5	**63,6**	582	0	NO_SP	1.000052	0.000000	0.000000	0.000000	0.000000	5	14	**2007323,333**	**35506**	**56,54**	**0,02**
A0A2Z5Y9G3	Superoxide dismutase [Cu-Zn]	4,9	**19,3**	193	0	NO_SP	1.000111	0.000003	0.000000	0.000000	0.000000	4	8	**2005533,333**	**90255**	**22,22**	**0,05**
A0A2Z5YEX6	Fatty acyl-CoA reductase	8,5	**36,3**	338	0	NO_SP	1.000060	0.000000	0.000000	0.000000	0.000000	3	9	**1995053,333**	**56042**	**35,60**	**0,03**
A0A2Z5YF81	N-acetyl-gamma-glutamyl-phosphate reductase	6,2	**36,3**	350	0	NO_SP	1.000055	0.000001	0.000000	0.000000	0.000000	4	9	**1993066,667**	**32914**	**60,55**	**0,02**
B2HMC7	Alpha-E domain-containing protein	6,0	**36,3**	325	0	NO_SP	1.000060	0.000000	0.000000	0.000000	0.000000	3	11	**1992466,667**	**54496**	**36,56**	**0,03**
A0A2Z5Y9R4	UPF0336 protein MMRN_08410	5,0	**18,3**	166	0	NO_SP	1.000005	0.000047	0.000001	0.000000	0.000000	4	6	**1985266,667**	**12413**	**159,93**	**0,01**
A0A2Z5YG32	Uncharacterized protein	6,6	**94,0**	886	0	NO_SP	1.000045	0.000001	0.000000	0.000000	0.000000	4	17	**1984500**	**29762**	**66,68**	**0,01**
A0A2Z5YF30	Vitamin B12 transport ATP-binding protein BacA	8,9	**68,9**	617	6	NO_SP	1.000042	0.000000	0.000000	0.000000	0.000000	4	14	**1984133,333**	**122574**	**16,19**	**0,06**
B2HNT3	Chromosome partitioning protein ParA	4,6	**39,9**	377	0	NO_SP	0.996643	0.003370	0.000012	0.000007	0.000003	4	9	**1980066,667**	**7265**	**272,56**	**0,00**
A0A2Z5YDY3	Uncharacterized protein	6,2	**28,7**	261	0	NO_SP	1.000052	0.000000	0.000000	0.000000	0.000000	3	6	**1979366,667**	**16239**	**121,89**	**0,01**
A0A2Z5YM24	6-aminohexanoate-cyclic-dimer hydrolase	5,4	**52,5**	497	0	NO_SP	1.000058	0.000002	0.000000	0.000000	0.000000	4	13	**1976333,333**	**20842**	**94,82**	**0,01**
A0A3E2MQ25	Phenolphthiocerol synthesis polyketide synthase type I Pks15/1	4,8	**220,2**	2114	2	NO_SP	0.999579	0.000395	0.000035	0.000003	0.000001	1	29	**1974100**	**7788**	**253,48**	**0,00**
A0A2Z5YBC6	Uncharacterized protein	4,2	**49,2**	466	0	NO_SP	0.999804	0.000200	0.000008	0.000002	0.000001	3	11	**1973553,333**	**21889**	**90,16**	**0,01**
B2HM80	Heat-inducible transcription repressor HrcA	5,0	**36,6**	343	0	NO_SP	1.000043	0.000000	0.000000	0.000000	0.000000	3	14	**1970833,333**	**601**	**3277,62**	**0,00**
A0A2Z5YEF0	Uncharacterized protein family UPF0051	4,7	**42,7**	399	0	NO_SP	1.000063	0.000001	0.000000	0.000000	0.000000	4	15	**1966433,333**	**4272**	**460,30**	**0,00**
A0A3E2MWJ6	Phosphoribosylaminoimidazole-succinocarboxamide synthase	4,6	**32,8**	297	0	NO_SP	1.000017	0.000030	0.000001	0.000000	0.000000	4	11	**1961566,667**	**2059**	**952,48**	**0,00**
B2HMI4	PHB domain-containing protein	4,5	**40,4**	377	0	NO_SP	1.000040	0.000000	0.000000	0.000000	0.000000	4	16	**1960533,333**	**20236**	**96,89**	**0,01**
A0A100IEF6	Medium chain fatty-acid-CoA ligase FadD14	5,2	**58,9**	539	0	NO_SP	0.999874	0.000158	0.000003	0.000000	0.000000	4	11	**1954833,333**	**53028**	**36,86**	**0,03**
A0A2Z5YJS3	Adenylate/guanylate cyclase domain-containing protein	4,8	**79,7**	730	2	NO_SP	0.999957	0.000047	0.000000	0.000000	0.000000	5	15	**1952266,667**	**30250**	**64,54**	**0,02**
B2HJW4	Uncharacterized protein	4,9	**35,4**	326	0	NO_SP	1.000045	0.000001	0.000000	0.000000	0.000000	2	6	**1951066,667**	**28512**	**68,43**	**0,01**
A0A2Z5YIR9	AAA domain-containing protein	4,8	**31,9**	291	0	NO_SP	1.000050	0.000009	0.000000	0.000000	0.000000	4	11	**1947466,667**	**28394**	**68,59**	**0,01**
A0A2Z5YKA9	6-phosphogluconate dehydrogenase (Decarboxylating)	4,8	**36,2**	340	0	NO_SP	1.000042	0.000000	0.000000	0.000000	0.000000	3	14	**1946703,333**	**0**	**#JAKO/0!**	**0,00**
A0A2Z5YD99	Ubiquinone biosynthesis O-methyltransferase	5,1	**27,4**	249	0	NO_SP	1.000063	0.000000	0.000000	0.000000	0.000000	3	11	**1933466,667**	**7975**	**242,45**	**0,00**
A0A2Z5YE95	Bifunctional (P)ppGpp synthase/hydrolase relA	7,3	**87,1**	787	0	NO_SP	1.000035	0.000000	0.000000	0.000000	0.000000	3	22	**1929100**	**3532**	**546,20**	**0,00**
A0A2Z5YAX4	dTDP-glucose 4,6-dehydratase	5,2	**37,3**	331	0	NO_SP	1.000049	0.000003	0.000000	0.000000	0.000000	4	11	**1914500**	**7927**	**241,53**	**0,00**
A0A2Z5YE90	Toxin	4,6	**15,4**	143	0	NO_SP	1.000072	0.000000	0.000000	0.000000	0.000000	3	4	**1914466,667**	**8833**	**216,73**	**0,00**
A0A3E2N046	ESX-1 secretion system protein eccB1	6,7	**55,6**	533	1	NO_SP	1.000061	0.000000	0.000000	0.000000	0.000000	5	15	**1905770**	**15956**	**119,44**	**0,01**
A0A2Z5Y896	Acyl-CoA synthetase	6,0	**56,8**	544	0	NO_SP	1.000045	0.000015	0.000000	0.000000	0.000000	4	14	**1898300**	**21248**	**89,34**	**0,01**

A0A3E2N2Z8	CHAD domain protein	8,9	57,6	530	0	NO_SP	1.000069	0.000000	0.000000	0.000000	0.000000	3	14	1897466,667	12826	147,94	0,01
A0A2Z5YC71	Ribonucleoside-diphosphate reductase subunit beta	4,3	36,9	324	0	NO_SP	1.000059	0.000003	0.000000	0.000000	0.000000	3	14	1895166,667	4578	413,93	0,00
A0A2Z5YDI3	Chlorite dismutase	5,6	26,4	231	0	NO_SP	1.000053	0.000002	0.000000	0.000000	0.000000	3	9	1894766,667	37152	51,00	0,02
A0A2Z5YE23	Uncharacterized protein	7,0	55,4	497	0	NO_SP	1.000085	0.000000	0.000000	0.000000	0.000000	4	15	1893333,333	48408	39,11	0,03
A0A2Z5YGU3	UDP-N-acetylglucosamine--N-acetylmuramyl-(pentapeptide) pyrophosphoryl-	9,9	41,1	399	0	NO_SP	0.999993	0.000036	0.000000	0.000000	0.000000	4	10	1892500	3402	556,23	0,00
A0A2Z5YNY5	CbiA domain-containing protein	7,0	35,1	329	0	NO_SP	1.000049	0.000002	0.000000	0.000000	0.000000	3	10	1876433,333	10639	176,37	0,00
A0A2Z5YEV2	Quinolinate synthase	5,0	37,6	352	0	NO_SP	1.000051	0.000000	0.000000	0.000000	0.000000	3	11	1875110	23823	78,71	0,01
A0A2Z5YCR1	Uncharacterized protein	9,3	9,7	84	2	NO_SP	1.000040	0.000007	0.000000	0.000000	0.000000	1	1	1874833,333	80293	23,35	0,04
A0A2Z5YEB0	Histidinol dehydrogenase	5,0	42,7	411	0	NO_SP	1.000066	0.000002	0.000000	0.000000	0.000000	4	12	1870233,333	23347	80,10	0,01
A0A2Z5Y8K5	Zinc metalloprotease	4,7	74,3	664	0	NO_SP	1.000060	0.000000	0.000000	0.000000	0.000000	5	20	1853333,333	16515	112,22	0,01
A0A100IF17	Type III pantothenate kinase	5,6	28,1	263	0	NO_SP	1.000012	0.000048	0.000001	0.000000	0.000000	3	7	1833800	14580	125,77	0,01
A0A2Z5YPZ4	CbiA domain-containing protein	6,6	25,6	234	0	NO_SP	1.000048	0.000000	0.000000	0.000000	0.000000	1	6	1833366,667	1974	928,82	0,00
A0A2Z5YD64	Chromosome partition protein Smc	4,9	131,3	1206	0	NO_SP	0.818867	0.180433	0.000359	0.000133	0.000093	4	26	1825500	4843	376,91	0,00
B2HJP0	Ribosome-recycling factor	6,0	21,0	185	0	NO_SP	1.000057	0.000000	0.000000	0.000000	0.000000	3	10	1815966,667	7636	237,82	0,00
A0A2Z5YCI7	Acyltransferase PapA5	4,6	45,4	414	0	NO_SP	1.000058	0.000000	0.000000	0.000000	0.000000	4	13	1811333,333	54193	33,42	0,03
A0A2Z5YAC4	50S ribosomal protein L22	11,9	19,5	185	0	NO_SP	1.000053	0.000000	0.000000	0.000000	0.000000	4	7	1808463,333	817060	2,21	0,45
A0A2Z5Y9Q7	Universal stress protein	6,2	30,0	287	0	NO_SP	1.000069	0.000000	0.000000	0.000000	0.000000	3	13	1808226,667	2720	664,82	0,00
A0A2Z5YJX7	Multifunctional fusion protein	6,7	68,0	616	0	NO_SP	0.999966	0.000085	0.000000	0.000000	0.000000	4	17	1803833,333	16636	108,43	0,01
B2HS37	Conserved hypothetical membrane protein	8,9	18,5	175	2	NO_SP	1.000066	0.000002	0.000000	0.000000	0.000000	4	5	1802633,333	32790	54,98	0,02
A0A2Z5YE07	Carboxymuconolactone decarboxylase family protein	4,8	20,6	187	0	NO_SP	1.000039	0.000000	0.000000	0.000000	0.000000	3	11	1799400	93907	19,16	0,05
B2HH92	Hypothetical alanine and valine rich protein	8,5	32,3	296	2	NO_SP	1.000026	0.000000	0.000000	0.000000	0.000000	4	6	1796200	88387	20,32	0,05
A0A100ICL5	50S ribosomal protein L27	11,9	9,3	88	0	NO_SP	1.000053	0.000000	0.000000	0.000000	0.000000	3	4	1795566,667	1438733	1,25	0,80
B2HCV5	Adenylate kinase	4,7	20,1	181	0	NO_SP	1.000061	0.000000	0.000000	0.000000	0.000000	7	9	1792166,667	44898	39,92	0,03
A0A2Z5YC50	Long-chain-fatty-acid--AMP ligase FadD26	4,9	62,9	584	0	NO_SP	1.000042	0.000002	0.000000	0.000000	0.000000	3	13	1787910	1888	947,05	0,00
B2HR28	Acetylglutamate kinase	5,0	30,8	293	0	NO_SP	1.000056	0.000000	0.000000	0.000000	0.000000	4	11	1785490	3071	581,35	0,00
A0A2Z5YBS9	NAD-binding protein of Kef-type K+ transporter	6,6	38,0	356	0	NO_SP	0.999757	0.000237	0.000000	0.000000	0.000000	3	11	1777900	63999	27,78	0,04
A0A3E2MMU4	Arylsulfatase	4,7	105,1	967	0	NO_SP	1.000078	0.000001	0.000000	0.000000	0.000000	4	19	1772600	10253	172,88	0,01
A0A3E2N2H3	UDP-N-acetylglucosamine 4,6-dehydratase (Inverting)	4,9	34,0	325	0	NO_SP	1.000056	0.000000	0.000000	0.000000	0.000000	4	12	1769466,667	9693	182,54	0,01
B2HEV4	Molybdenum cofactor biosynthesis protein MoeB1	4,9	42,3	393	1	NO_SP	1.000003	0.000040	0.000000	0.000000	0.000000	3	10	1769233,333	45767	38,66	0,03
B2HKV8	Conserved protein	8,5	16,7	154	0	NO_SP	1.000054	0.000004	0.000000	0.000000	0.000000	3	10	1767433,333	18300	96,58	0,01
A0A2Z5YH96	Bifunctional glutamine synthetase adenylyltransferase/adenylyl-removing enz	7,0	108,6	995	0	NO_SP	1.000038	0.000004	0.000000	0.000000	0.000000	4	20	1759433,333	17078	103,02	0,01
B2HHI6	Transferase	10,2	27,5	245	0	NO_SP	0.999852	0.000129	0.000016	0.000001	0.000000	4	8	1758033,333	58740	29,93	0,03
A0A2Z5YK94	Putative peroxiredoxin	8,9	17,0	157	0	NO_SP	1.000074	0.000001	0.000000	0.000000	0.000000	4	9	1755966,667	0	#JAKO/0!	0,00
A0A100HYY8	Protein translocase subunit SecD	9,5	63,6	608	6	NO_SP	0.861570	0.135505	0.002227	0.000207	0.000140	4	7	1748333,333	73522	23,78	0,04
A0A2Z5Y7G7	Serine/threonine-protein kinase PknB	5,5	66,5	626	1	NO_SP	1.000053	0.000000	0.000000	0.000000	0.000000	4	14	1746200	5708	305,90	0,00
A0A2Z5YD90	Polyphosphate glucokinase	5,3	27,5	264	0	NO_SP	1.000071	0.000000	0.000000	0.000000	0.000000	4	11	1739866,667	15648	111,19	0,01
A0A2Z5YAY9	Malonyl-[acyl-carrier protein] O-methyltransferase	5,1	26,4	245	0	NO_SP	1.000053	0.000002	0.000000	0.000000	0.000000	2	7	1735500	770750	2,25	0,44
A0A3E2MYP7	Methionine aminopeptidase	5,1	27,6	266	0	NO_SP	1.000025	0.000026	0.000000	0.000000	0.000000	4	10	1728370	0	#JAKO/0!	0,00
B2HDV7	Conserved protein	3,8	48,5	441	0	NO_SP	1.000055	0.000000	0.000000	0.000000	0.000000	4	8	1727700	19881	86,90	0,01
A0A2Z5YD54	Uncharacterized protein	7,0	110,0	1035	0	NO_SP	0.987299	0.012357	0.000213	0.000051	0.000026	1	4	1727100	0	#JAKO/0!	0,00
A0A2Z5YE40	Putative FeS assembly protein SufB	5,0	53,2	481	0	NO_SP	1.000012	0.000018	0.000000	0.000000	0.000000	4	14	1721136,667	4951	347,67	0,00
B2HLY6	Soluble pyridine nucleotide transhydrogenase SthA	5,5	50,9	471	0	NO_SP	1.000046	0.000000	0.000000	0.000000	0.000000	4	9	1719266,667	16147	106,48	0,01
A0A2Z5YEC0	Cyanophycinase	5,0	76,8	734	0	NO_SP	1.000080	0.000000	0.000000	0.000000	0.000000	3	14	1717940	86288	19,91	0,05
A0A2Z5YF88	Copper transporter MctB	4,8	31,9	317	1	NO_SP	0.871124	0.019098	0.008837	0.000210	0.000108	3	8	1717466,667	33477	51,30	0,02
A0A3E2MYN4	Protease 4	4,9	63,7	600	0	NO_SP	0.985762	0.014121	0.000061	0.000038	0.000015	5	12	1711000	1985	862,04	0,00
A0A2Z5Y887	Haloacid dehalogenase	5,0	166,9	1620	3	NO_SP	0.991236	0.008692	0.000066	0.000015	0.000006	4	23	1697150	24967	67,98	0,01
B2HQ40	Nucleotidyltransferase	6,0	28,2	245	0	NO_SP	1.000066	0.000000	0.000000	0.000000	0.000000	3	7	1696733,333	19421	87,37	0,01
B2HNI5	Uncharacterized protein	6,9	15,8	138	0	NO_SP	1.000057	0.000000	0.000000	0.000000	0.000000	4	9	1692713,333	13435	125,99	0,01
A0A2Z5YDT6	Protein-export membrane protein SecF	9,9	47,6	446	6	NO_SP	1.000068	0.000001	0.000000	0.000000	0.000000	3	8	1690166,667	23465	72,03	0,01
B2HQB1	Uncharacterized protein	4,1	11,9	114	0	NO_SP	1.000045	0.000000	0.000000	0.000000	0.000000	3	4	1689766,667	104957	16,10	0,06
B2HJL7	50S ribosomal protein L19	10,8	13,0	113	0	NO_SP	1.000054	0.000000	0.000000	0.000000	0.000000	3	5	1688066,667	160460	10,52	0,10
A0A2Z5YJQ4	Acyl-CoA dehydrogenase	4,7	39,9	372	0	NO_SP	1.000063	0.000001	0.000000	0.000000	0.000000	4	13	1685226,667	3039	554,61	0,00
B2HDH7	ESAT-6-like protein	4,6	9,9	94	0	NO_SP	1.000051	0.000000	0.000000	0.000000	0.000000	7	4	1681800	18693	89,97	0,01
B2HRU9	GTPase Der	9,2	50,9	469	0	NO_SP	1.000070	0.000000	0.000000	0.000000	0.000000	4	16	1678433,333	34357	48,85	0,02
B2HK33	Aminotransferase (Adenosylmethionine-8-amino-7-oxononanoate) BioA	4,9	49,0	451	0	NO_SP	1.000015	0.000000	0.000000	0.000000	0.000000	4	14	1677060	0	#JAKO/0!	0,00
A0A2Z5YL59	ATP-dependent DNA helicase	5,1	84,9	769	0	NO_SP	1.000070	0.000001	0.000000	0.000000	0.000000	4	23	1676433,333	63339	26,47	0,04
A0A2Z5YI85	Gamma-glutamyltranspeptidase	5,8	66,5	646	1	LIPO	0.000000	0.000001	1.000044	0.000000	0.000000	4	12	1671133,333	0	#JAKO/0!	0,00
A0A3E2N158	Glutamate-pyruvate aminotransferase AlaA	4,9	47,0	423	0	NO_SP	1.000054	0.000000	0.000000	0.000000	0.000000	4	11	1659723,333	17945	92,49	0,01
A0A2Z5YNF4	Hydrolase	4,7	68,6	624	0	NO_SP	1.000036	0.000004	0.000000	0.000000	0.000000	3	20	1655150	19805	83,57	0,01
A0A2Z5YKT5	Bifunctional protein GlmU	5,4	51,2	492	0	NO_SP	1.000054	0.000001	0.000000	0.000000	0.000000	4	12	1655026,667	2205	750,58	0,00
B2HS75	AB hydrolase-1 domain-containing protein	6,8	32,4	302	0	NO_SP	1.000056	0.000003	0.000000	0.000000	0.000000	4	10	1654833,333	14767	112,06	0,01
A0A2Z5Y8G1	Phosphotyrosine protein phosphatase	4,8	30,2	276	0	NO_SP	1.000059	0.000000	0.000000	0.000000	0.000000	4	12	1654100	156295	10,58	0,09
A0A2Z5YDU0	6,7-dimethyl-8-ribityllumazine synthase	4,4	16,4	160	0	NO_SP	1.000055	0.000002	0.000000	0.000000	0.000000	3	7	1652066,667	985	1677,11	0,00
A0A100I666	Lipoprotein, LpqD	6,2	23,2	217	0	LIPO	0.000516	0.000000	0.999515	0.000000	0.000000	3	7	1651433,333	25003	66,05	0,02
A0A3E2MQG1	Carboxylesterase NlhH	4,8	32,5	307	0	NO_SP	1.000056	0.000000	0.000000	0.000000	0.000000	4	8	1650810	5080	324,95	0,00
A0A2Z5YL20	Uncharacterized protein	4,9	60,1	552	0	LIPO	0.240577	0.060752	0.698282	0.000135	0.000127	4	11	1642866,667	11898	138,08	0,01
B2HMC9	30S ribosomal protein S20	12,2	9,6	87	0	NO_SP	1.000041	0.000000	0.000000	0.000000	0.000000	3	3	1636033,333	1129567	1,45	0,69
A0A2Z5YDD9	Uncharacterized protein	9,7	25,2	226	0	NO_SP	0.995097	0.004765	0.000072	0.000027	0.000012	4	10	1629786,667	46098	35,36	0,03
A0A2Z5YPK8	ESX-1 secretion system protein eccB1	6,5	51,3	481	1	NO_SP	0.999960	0.000051	0.000000	0.000000	0.000000	5	10	1624533,333	28069	57,88	0,02
B2HEB5	Biotin carboxyl carrier protein	5,0	70,1	662	0	NO_SP	1.000028	0.000006	0.000000	0.000000	0.000000	1	16	1624233,333	795190	2,04	0,49
B2HJ83	Conserved protein	5,2	38,0	343	0	NO_SP	1.000060	0.000000	0.000000	0.000000	0.000000	3	12	1622666,667	0	#JAKO/0!	0,00
A0A2Z5YJF1	Ribonuclease BN	4,9	27,2	255	0	NO_SP	1.000075	0.000000	0.000000	0.000000	0.000000	3	6	1610250	7051	228,39	0,00
A0A2Z5YC86	Demethylrebeccamycin-D-glucose O-methyltransferase	5,1	36,2	327	0	NO_SP	0.999833	0.000194	0.000001	0.000000	0.000000	3	11	1607466,667	9380	171,38	0,01
A0A2Z5YFM8	Lipase LipD	6,4	45,0	427	0	NO_SP	0.999994	0.000056	0.000001	0.000000	0.000000	3	11	1601503,333	6751	237,22	0,00
A0A3E2MQF7	Ribosomal RNA small subunit methyltransferase B	7,0	48,7	457	0	NO_SP	1.000044	0.000002	0.000000	0.000000	0.000000	4	15	1601133,333	16963	94,39	0,01
A0A3E2MS03	Putative Rieske 2Fe-2S iron-sulfur protein	4,7	58,1	516	0	NO_SP	1.000039	0.000008	0.000000	0.000000	0.000000	4	12	1600766,667	25551	62,65	0,02
A0A3E2MUB7	Histidine kinase	5,8	55,6	514	2	NO_SP	0.859892	0.001034	0.000371	0.000010	0.000005	5	15	1599813,333	19139	83,59	0,01

A0A2Z5YK99	3 beta-hydroxysteroid dehydrogenase/Delta 5-->4-isomerase	7,0	**39,5**	360	0	NO_SP	1.000069	0.000000	0.000000	0.000000	0.000000	4	8	**1596500**	**19807**	**80,60**	**0,01**
A0A2Z5YCE0	Lipase	6,5	**37,3**	353	0	NO_SP	1.000017	0.000011	0.000000	0.000000	0.000000	4	9	**1593150**	**8114**	**196,35**	**0,01**
A0A2Z5YNJ9	Acyl-CoA dehydrogenase	4,6	**38,3**	350	0	NO_SP	1.000058	0.000000	0.000000	0.000000	0.000000	3	11	**1582166,667**	**30979**	**51,07**	**0,02**
A0A2Z5YCH3	Phthiocerol dimycocerosate exporter MmpL7	6,9	**93,6**	919	12	NO_SP	1.000057	0.000004	0.000000	0.000000	0.000000	4	15	**1582140**	**50381**	**31,40**	**0,03**
B2HPL1	Conserved membrane protein	10,2	**8,3**	75	1	NO_SP	0.999862	0.000146	0.000000	0.000000	0.000000	3	2	**1575533,333**	**136989**	**11,50**	**0,09**
A0A100I248	S-adenosyl-L-methionine-dependent methyltransferase	7,9	**31,9**	277	0	NO_SP	1.000052	0.000000	0.000000	0.000000	0.000000	4	10	**1564246,667**	**33449**	**46,77**	**0,02**
B2HFY1	L-cysteine:1D-myo-inositol 2-amino-2-deoxy-alpha-D-glucopyranoside ligase	5,1	**44,7**	411	0	NO_SP	1.000045	0.000010	0.000001	0.000000	0.000000	4	14	**1563750**	**9636**	**162,28**	**0,01**
A0A2Z5YD85	Ribonuclease D	5,1	**47,1**	435	0	NO_SP	0.999311	0.000684	0.000034	0.000001	0.000001	4	10	**1563133,333**	**13799**	**113,28**	**0,01**
A0A2Z5YKQ6	Short-chain dehydrogenase	10,1	**31,2**	293	0	NO_SP	1.000039	0.000000	0.000000	0.000000	0.000000	3	10	**1560233,333**	**37815**	**41,26**	**0,02**
A0A2Z5Y7L5	50S ribosomal protein L9	10,0	**16,2**	152	0	NO_SP	1.000047	0.000001	0.000000	0.000000	0.000000	3	4	**1557480**	**152402**	**10,22**	**0,10**
A0A100I6N5	Serine protease	4,7	**35,6**	364	1	NO_SP	0.547762	0.449126	0.000933	0.000765	0.000548	4	6	**1556966,667**	**0**	**#JAKO/0!**	**0,00**
A0A2Z5YIE1	Sulfate/thiosulfate import ATP-binding protein CysA	6,6	**38,2**	363	0	NO_SP	1.000072	0.000000	0.000000	0.000000	0.000000	4	12	**1556330**	**3046**	**510,97**	**0,00**
A0A2Z5Y9B3	Peptidase M28	6,2	**50,2**	490	0	LIPO	0.000000	0.000000	1.000026	0.000000	0.000000	4	13	**1550813,333**	**0**	**#JAKO/0!**	**0,00**
A0A2Z5YKB7	SBP_bac_5 domain-containing protein	5,7	**59,0**	552	0	LIPO	0.000000	0.000000	1.000084	0.000000	0.000000	4	12	**1543000**	**20237**	**76,25**	**0,01**
B2HMN9	4-hydroxy-4-methyl-2-oxoglutarate aldolase	4,5	**16,9**	165	0	NO_SP	1.000043	0.000000	0.000000	0.000000	0.000000	4	5	**1541166,667**	**5969**	**258,20**	**0,00**
A0A2Z5YD66	LexA repressor	4,5	**25,7**	244	0	NO_SP	1.000029	0.000007	0.000000	0.000000	0.000000	3	7	**1531166,667**	**13540**	**113,09**	**0,01**
A0A100I0P5	Type VII secretion-associated serine protease mycosin	6,5	**42,3**	423	1	NO_SP	1.000038	0.000006	0.000000	0.000000	0.000000	4	7	**1528013,333**	**4176**	**365,87**	**0,00**
B2HME6	Methyltransf_25 domain-containing protein	4,9	**38,5**	345	0	NO_SP	1.000049	0.000000	0.000000	0.000000	0.000000	4	12	**1522643,333**	**2455**	**620,24**	**0,00**
A0A2Z5YFF5	Urease accessory protein UreG	5,8	**23,6**	223	0	NO_SP	1.000042	0.000000	0.000000	0.000000	0.000000	3	7	**1521180**	**23800**	**63,92**	**0,02**
B2HL54	Conserved membrane protein	4,5	**48,6**	457	0	NO_SP	0.999845	0.000143	0.000024	0.000001	0.000000	4	12	**1520850**	**9902**	**153,59**	**0,01**
A0A2Z5YLD6	Acetyl-CoA acetyltransferase	4,8	**43,3**	411	0	NO_SP	1.000056	0.000000	0.000000	0.000000	0.000000	4	11	**1518866,667**	**0**	**#JAKO/0!**	**0,00**
B2HNS5	Conserved transmembrane protein	6,9	**122,4**	1180	15	NO_SP	0.999986	0.000038	0.000000	0.000000	0.000000	4	12	**1518806,667**	**556**	**2731,83**	**0,00**
A0A100HZJ7	Glycosyl transferase, family 2	7,0	**34,7**	314	2	NO_SP	1.000069	0.000000	0.000000	0.000000	0.000000	3	4	**1515963,333**	**27636**	**54,86**	**0,02**
B2HHR4	Malonyl CoA-acyl carrier protein transacylase FabD	4,6	**31,1**	302	0	NO_SP	0.991296	0.008516	0.000123	0.000028	0.000012	4	9	**1515710**	**8963**	**169,10**	**0,00**
A0A2Z5YNQ1	ThiF domain-containing protein	5,3	**76,0**	704	0	NO_SP	0.999649	0.000255	0.000078	0.000004	0.000002	3	14	**1515700**	**45959**	**32,98**	**0,03**
A0A2Z5Y8V1	Glyco_hydro_3 domain-containing protein	4,6	**35,1**	336	0	NO_SP	1.000060	0.000000	0.000000	0.000000	0.000000	2	7	**1515433,333**	**0**	**#JAKO/0!**	**0,00**
A0A2Z5YHT4	Pribosyltran domain-containing protein	5,2	**71,6**	656	0	NO_SP	1.000064	0.000000	0.000000	0.000000	0.000000	4	18	**1515140**	**15939**	**95,06**	**0,01**
B2HKJ8	Uncharacterized protein	10,3	**18,2**	163	0	NO_SP	1.000054	0.000001	0.000000	0.000000	0.000000	3	7	**1513533,333**	**22123**	**68,41**	**0,01**
A0A2Z5YAT3	N-acyl-L-amino acid amidohydrolase	5,2	**41,5**	389	0	NO_SP	1.000058	0.000000	0.000000	0.000000	0.000000	4	10	**1507920**	**74768**	**20,17**	**0,05**
B2HHY2	Trehalose-6-phosphate synthase	6,6	**56,1**	500	0	NO_SP	0.999195	0.000633	0.000124	0.000007	0.000002	4	9	**1506066,667**	**6579**	**228,92**	**0,00**
A0A2Z5YD19	Uncharacterized protein	4,3	**26,3**	247	0	NO_SP	1.000057	0.000013	0.000000	0.000000	0.000000	4	9	**1502433,333**	**20726**	**72,49**	**0,01**
A0A2Z5YLI1	Thiolase_N domain-containing protein	5,0	**31,6**	299	0	NO_SP	1.000049	0.000000	0.000000	0.000000	0.000000	1	1	**1502033,333**	**51100**	**29,39**	**0,03**
A0A2Z5YC38	FeIII-dicitrate-binding periplasmic lipoprotein	4,6	**33,9**	327	0	NO_SP	0.998805	0.001213	0.000015	0.000002	0.000001	4	8	**1499833,333**	**2483**	**603,98**	**0,00**
A0A2Z5YMP1	Hydroxyacylglutathione hydrolase	4,5	**20,6**	193	0	NO_SP	0.999790	0.000221	0.000010	0.000000	0.000000	3	5	**1488496,667**	**537509**	**2,77**	**0,36**
A0A3E2MR68	Transcriptional regulatory protein EmbR	7,0	**42,0**	386	0	NO_SP	1.000062	0.000000	0.000000	0.000000	0.000000	4	12	**1479176,667**	**51514**	**28,71**	**0,03**
B2HM63	Uncharacterized protein	5,2	**31,3**	297	0	NO_SP	1.000059	0.000001	0.000000	0.000000	0.000000	4	10	**1479133,333**	**597**	**2477,61**	**0,00**
A0A2Z5YHT2	Pyruvate dehydrogenase E1 component subunit beta	4,9	**34,7**	325	0	NO_SP	0.999035	0.000854	0.000098	0.000008	0.000004	4	12	**1478166,667**	**7910**	**186,87**	**0,01**
A0A2Z5YN35	Inorganic pyrophosphatase	4,4	**18,6**	162	0	NO_SP	1.000082	0.000000	0.000000	0.000000	0.000000	3	9	**1477900**	**7451**	**198,36**	**0,00**
B2HD82	Acyl-CoA:diacylglycerol acyltransferase	5,3	**34,4**	325	1	TAT	0.000831	0.340169	0.000384	0.654493	0.003944	3	6	**1475600**	**14630**	**100,86**	**0,01**
A0A2Z5YM50	Phosphate import ATP-binding protein PstB	5,4	**28,4**	258	0	NO_SP	1.000078	0.000000	0.000000	0.000000	0.000000	3	9	**1474013,333**	**1532**	**962,40**	**0,00**
A0A2Z5YEZ0	Acyl-CoA thioesterase 2	6,3	**33,7**	301	0	NO_SP	1.000051	0.000000	0.000000	0.000000	0.000000	3	10	**1470446,667**	**13996**	**105,06**	**0,01**
A0A2Z5YM67	Molybdopterin molybdenumtransferase	7,0	**44,7**	429	0	NO_SP	0.999507	0.000526	0.000001	0.000001	0.000000	3	9	**1466466,667**	**8639**	**169,74**	**0,01**
A0A2Z5YET7	TPPK_C domain-containing protein	7,0	**44,9**	414	1	NO_SP	1.000060	0.000001	0.000000	0.000000	0.000000	3	16	**1462566,667**	**11390**	**128,41**	**0,01**
B2HKH1	Serine hydroxymethyltransferase	5,5	**45,5**	425	0	NO_SP	1.000047	0.000003	0.000000	0.000000	0.000000	4	15	**1462486,667**	**12980**	**112,67**	**0,01**
A0A2Z5YF13	Oxidoreductase	8,9	**41,6**	378	0	NO_SP	1.000061	0.000000	0.000000	0.000000	0.000000	5	11	**1458226,667**	**76380**	**19,09**	**0,05**
B2HPC4	ATP-binding protein ABC transporter	5,2	**58,6**	542	0	NO_SP	1.000051	0.000007	0.000000	0.000000	0.000000	3	14	**1455256,667**	**201527**	**7,22**	**0,14**
A0A2Z5YI97	Elongation factor 4	4,9	**64,1**	579	0	NO_SP	1.000067	0.000000	0.000000	0.000000	0.000000	4	15	**1454410**	**9720**	**149,63**	**0,01**
A0A2Z5YB43	Phosphoenolpyruvate transferase	5,2	**35,2**	329	0	NO_SP	1.000045	0.000001	0.000000	0.000000	0.000000	4	13	**1453690**	**44795**	**32,45**	**0,03**
B2HEU3	Acetyltransferase	5,0	**17,8**	160	0	NO_SP	1.000027	0.000002	0.000000	0.000000	0.000000	4	6	**1448880**	**30361**	**47,72**	**0,02**
A0A2Z5YEY9	Uncharacterized protein	5,0	**20,4**	187	0	NO_SP	1.000080	0.000000	0.000000	0.000000	0.000000	4	7	**1447663,333**	**21252**	**68,12**	**0,01**
A0A3E2MWE7	Diacylglycerol O-acyltransferase	5,9	**53,6**	496	0	NO_SP	1.000058	0.000000	0.000000	0.000000	0.000000	4	10	**1447403,333**	**15604**	**92,76**	**0,01**
B2HRM4	3-oxoacyl-[acyl-carrier-protein] synthase 3	4,5	**34,8**	335	0	NO_SP	1.000006	0.000067	0.000000	0.000000	0.000000	4	9	**1446390**	**18399**	**78,61**	**0,01**
A0A2Z5YL36	Serine hydrolase	4,7	**55,0**	515	0	LIPO	0.000011	0.000001	1.000051	0.000000	0.000000	4	11	**1441380**	**2057**	**700,79**	**0,00**
A0A2Z5Y8E3	3-alpha-hydroxysteroid dehydrogenase	4,8	**24,3**	229	0	NO_SP	1.000064	0.000001	0.000000	0.000000	0.000000	4	9	**1441100**	**0**	**#JAKO/0!**	**0,00**
A0A100I697	O-phosphoserine phosphohydrolase	4,4	**42,9**	405	0	NO_SP	1.000056	0.000000	0.000000	0.000000	0.000000	4	10	**1436896,667**	**27765**	**51,75**	**0,02**
B2HNF5	Type I modular polyketide synthase	5,5	**582,2**	5495	0	NO_SP	1.000043	0.000000	0.000000	0.000000	0.000000	2	2	**1435066,667**	**1617077**	**0,89**	**1,13**
A0A2Z5YB50	dTDP-4-dehydrorhamnose reductase	4,6	**29,5**	288	0	NO_SP	1.000028	0.000006	0.000000	0.000000	0.000000	2	9	**1433356,667**	**4626**	**309,84**	**0,00**
A0A2Z5YJ37	Putative succinyl-CoA:3-ketoacid coenzyme A transferase subunit A	5,1	**26,4**	248	0	NO_SP	1.000051	0.000011	0.000000	0.000000	0.000000	4	7	**1432783,333**	**1911**	**749,87**	**0,00**
A0A2Z5Y859	Putative dehydrogenase	9,4	**57,0**	537	0	NO_SP	0.999985	0.000051	0.000001	0.000000	0.000000	4	14	**1432420**	**8567**	**167,21**	**0,01**
A0A2Z5YEI5	Arginine biosynthesis bifunctional protein ArgJ	5,0	**43,0**	420	0	NO_SP	1.000038	0.000000	0.000000	0.000000	0.000000	4	11	**1431056,667**	**12626**	**113,34**	**0,01**
A0A2Z5YGZ5	Anthranilate phosphoribosyltransferase	4,8	**34,1**	331	0	NO_SP	1.000064	0.000000	0.000000	0.000000	0.000000	3	9	**1428833,333**	**2887**	**494,95**	**0,00**
A0A2Z5YHI6	Diguanylate cyclase	5,1	**59,3**	549	0	NO_SP	1.000041	0.000000	0.000000	0.000000	0.000000	4	16	**1428580**	**32139**	**44,45**	**0,02**
B2HD34	Oxidoreductase	5,2	**33,4**	307	0	NO_SP	0.999346	0.000635	0.000041	0.000002	0.000001	4	13	**1428406,667**	**12270**	**116,41**	**0,01**
A0A2Z5YB31	Marine proteobacterial sortase target protein	4,6	**77,1**	731	0	NO_SP	1.000059	0.000001	0.000000	0.000000	0.000000	3	11	**1428400**	**11504**	**124,17**	**0,01**
A0A2Z5YNC6	Uncharacterized protein	4,2	**13,6**	127	0	NO_SP	0.841549	0.155627	0.001323	0.000537	0.000299	4	2	**1425606,667**	**67625**	**21,08**	**0,05**
A0A2Z5Y7L1	Transcriptional regulator	6,0	**17,9**	167	0	NO_SP	1.000025	0.000000	0.000000	0.000000	0.000000	4	6	**1417383,333**	**32138**	**44,10**	**0,02**
A0A3E2N132	Chaperone protein DnaK	4,5	**61,4**	610	1	NO_SP	1.000022	0.000000	0.000000	0.000000	0.000000	3	9	**1414973,333**	**3377**	**418,97**	**0,00**
A0A2Z5YA94	Arylsulfatase AtsA	5,0	**86,3**	785	0	NO_SP	1.000041	0.000026	0.000001	0.000000	0.000000	4	12	**1413026,667**	**13776**	**102,57**	**0,01**
A0A2Z5YHP4	Putative oxidoreductase	10,2	**34,9**	323	0	NO_SP	1.000042	0.000000	0.000000	0.000000	0.000000	4	9	**1412433,333**	**20016**	**70,57**	**0,01**
A0A3E2MUK4	50S ribosomal protein L32	12,8	**6,5**	57	0	NO_SP	1.000032	0.000000	0.000000	0.000000	0.000000	3	3	**1410833,333**	**966313**	**1,46**	**0,68**
A0A2Z5YK14	Non-specific serine/threonine protein kinase	9,8	**71,6**	663	1	NO_SP	1.000063	0.000000	0.000000	0.000000	0.000000	4	11	**1407506,667**	**7924**	**177,63**	**0,01**
B2HSR8	Conserved protein	7,0	**45,7**	413	0	NO_SP	1.000055	0.000001	0.000000	0.000000	0.000000	4	7	**1407223,333**	**9294**	**151,41**	**0,01**
A0A2Z5YNJ5	1-acyl-sn-glycerol-3-phosphate acyltransferase	10,6	**28,5**	259	0	NO_SP	1.000066	0.000000	0.000000	0.000000	0.000000	4	5	**1407070**	**28347**	**49,64**	**0,02**
B2HIF8	ATP-dependent 6-phosphofructokinase	6,4	**37,2**	343	0	NO_SP	0.999979	0.000057	0.000002	0.000000	0.000000	4	10	**1402160**	**6121**	**229,07**	**0,00**
B2HHG9	Cytochrome c oxidase subunit 1	7,1	**63,7**	573	12	NO_SP	1.000017	0.000019	0.000000	0.000000	0.000000	4	4	**1401983,333**	**0**	**#JAKO/0!**	**0,00**
A0A100I1I7	Luciferase family protein	5,7	**27,1**	247	0	NO_SP	1.000066	0.000003	0.000000	0.000000	0.000000	4	12	**1398160**	**14008**	**99,81**	**0,01**
B2HHH8	Conserved ATP-binding protein ABC transporter	4,9	**30,4**	280	0	NO_SP	1.000054	0.000000	0.000000	0.000000	0.000000	4	10	**1397740**	**6678**	**209,31**	**0,00**

A0A2Z5YK19	Putative ABC transporter ATP-binding protein	5,3	**65,6**	612	0	NO_SP	1.000060	0.000000	0.000000	0.000000	0.000000	4	17	**1396490**	**1555**	**897,95**	**0,00**
A0A100IAZ6	Methionine aminopeptidase	4,7	**29,5**	268	0	NO_SP	1.000053	0.000001	0.000000	0.000000	0.000000	3	4	**1390270**	**34625**	**40,15**	**0,02**
A0A2Z5YK22	Acetyl-/propionyl-CoA carboxylase subunit beta	6,3	**56,5**	526	0	NO_SP	1.000046	0.000007	0.000000	0.000000	0.000000	4	17	**1388210**	**22110**	**62,79**	**0,02**
A0A2Z5YL21	EspC protein homolog	5,6	**10,9**	103	0	NO_SP	1.000045	0.000002	0.000000	0.000000	0.000000	3	2	**1384633,333**	**0**	**#JAKO/0!**	**0,00**
A0A2Z5YN08	Uncharacterized protein	10,1	**18,9**	172	2	NO_SP	0.998248	0.001704	0.000044	0.000005	0.000003	3	3	**1379466,667**	**13245**	**104,15**	**0,01**
B2HPY0	Acyl-CoA dehydrogenase FadE7	6,2	**42,4**	395	0	NO_SP	0.999952	0.000082	0.000000	0.000000	0.000000	3	10	**1375430**	**9088**	**151,34**	**0,01**
A0A2Z5YGU2	NADP-dependent oxidoreductase	4,5	**35,4**	337	0	NO_SP	1.000067	0.000000	0.000000	0.000000	0.000000	3	9	**1373673,333**	**13591**	**101,08**	**0,01**
B2HHL5	Conserved transmembrane protein	12,0	**26,8**	250	2	NO_SP	0.999835	0.000174	0.000000	0.000000	0.000000	3	6	**1371923,333**	**68337**	**20,08**	**0,05**
A0A2Z5YN33	Putative fatty acid methyltransferase	6,3	**48,6**	439	0	NO_SP	1.000041	0.000027	0.000000	0.000000	0.000000	4	15	**1364106,667**	**1148121**	**1,19**	**0,84**
A0A2Z5YBS2	4-hydroxyacetophenone monooxygenase	7,3	**57,7**	516	0	NO_SP	0.999185	0.000861	0.000004	0.000002	0.000001	3	12	**1360563,333**	**11376**	**119,60**	**0,01**
A0A2Z5YHJ8	Uncharacterized protein	6,4	**36,2**	329	0	NO_SP	1.000046	0.000016	0.000000	0.000000	0.000000	4	14	**1359263,333**	**0**	**#JAKO/0!**	**0,00**
A0A2Z5YAC5	Uncharacterized protein	6,8	**30,3**	258	1	NO_SP	1.000033	0.000000	0.000000	0.000000	0.000000	4	8	**1354320**	**21348**	**63,44**	**0,02**
B2HKT1	Enoyl-CoA hydratase, EchA16_2	5,1	**26,5**	249	0	NO_SP	1.000072	0.000000	0.000000	0.000000	0.000000	4	6	**1351746,667**	**8891**	**152,03**	**0,01**
A0A117DTJ1	HIT family hydrolase	5,6	**14,8**	134	0	NO_SP	1.000057	0.000000	0.000000	0.000000	0.000000	3	6	**1351686,667**	**15203**	**88,91**	**0,01**
B2HMK5	Conserved transmembrane protein	7,7	**32,7**	302	8	NO_SP	0.999996	0.000018	0.000000	0.000000	0.000000	4	5	**1351000**	**57652**	**23,43**	**0,04**
A0A124BUY4	Serine/threonine phosphatase PstP	4,7	**54,0**	515	1	NO_SP	1.000018	0.000058	0.000000	0.000000	0.000000	4	8	**1346946,667**	**26194**	**51,42**	**0,02**
A0A2Z5YIU0	Acetate--CoA ligase	5,7	**65,4**	599	0	NO_SP	1.000087	0.000000	0.000000	0.000000	0.000000	4	14	**1346756,667**	**306**	**4400,69**	**0,00**
B2HEA6	Large-conductance mechanosensitive channel	5,8	**16,1**	151	2	NO_SP	0.834358	0.033901	0.000606	0.000235	0.000176	3	7	**1345960**	**75637**	**17,79**	**0,06**
A0A3E2MWL1	Protease 2	4,8	**79,1**	705	0	NO_SP	1.000070	0.000000	0.000000	0.000000	0.000000	4	14	**1344756,667**	**3927**	**342,44**	**0,00**
B2HNX1	Conserved transmembrane protein	6,5	**47,8**	472	11	NO_SP	0.999959	0.000080	0.000002	0.000000	0.000000	4	3	**1343646,667**	**34639**	**38,79**	**0,03**
A0A2Z5YBS0	HTH-type transcriptional repressor KstR2	7,1	**25,0**	227	0	NO_SP	1.000035	0.000000	0.000000	0.000000	0.000000	3	7	**1341966,667**	**0**	**#JAKO/0!**	**0,00**
B2HH08	Phosphate-specific transport system accessory protein PhoU	5,2	**24,7**	222	0	NO_SP	1.000040	0.000011	0.000000	0.000000	0.000000	4	10	**1337603,333**	**1472**	**908,82**	**0,00**
A0A3E2MRC4	Membrane transport protein mmpL8	7,2	**108,0**	998	11	NO_SP	1.000060	0.000002	0.000000	0.000000	0.000000	4	13	**1336393,333**	**12464**	**107,22**	**0,01**
B2HNG6	Trigger factor	4,0	**52,4**	484	0	NO_SP	1.000046	0.000000	0.000000	0.000000	0.000000	4	9	**1335556,667**	**39759**	**33,59**	**0,03**
A0A2Z5YGM0	UDP-N-acetylmuramate--L-alanine ligase	6,0	**49,0**	474	0	NO_SP	1.000068	0.000000	0.000000	0.000000	0.000000	4	8	**1334276,667**	**4248**	**314,09**	**0,00**
A0A2Z5YJY8	NADPH-dependent 2,4-dienoyl-CoA reductase	8,2	**72,6**	674	0	NO_SP	1.000047	0.000000	0.000000	0.000000	0.000000	5	14	**1331906,667**	**2297497**	**0,58**	**1,72**
B2HQ41	Predicted pyridoxal phosphate-dependent enzyme	6,7	**49,2**	447	0	NO_SP	1.000075	0.000000	0.000000	0.000000	0.000000	3	13	**1327430**	**0**	**#JAKO/0!**	**0,00**
A0A2Z5YFK3	CinA-like protein	4,8	**45,1**	425	0	NO_SP	1.000064	0.000002	0.000000	0.000000	0.000000	4	13	**1327093,333**	**4326**	**306,74**	**0,00**
A0A2Z5Y7G5	Cold shock-like protein CspD	4,7	**14,7**	135	0	NO_SP	1.000061	0.000000	0.000000	0.000000	0.000000	3	4	**1326433,333**	**2539**	**522,53**	**0,00**
A0A2Z5YK93	Acyl-CoA synthetase	6,2	**49,8**	472	0	NO_SP	0.576145	0.421444	0.000662	0.000856	0.000398	3	14	**1325366,667**	**67266**	**19,70**	**0,05**
A0A2Z5YGA8	Proteasome subunit beta	4,6	**25,7**	245	0	NO_SP	1.000029	0.000006	0.000000	0.000000	0.000000	4	9	**1324630**	**43019**	**30,79**	**0,03**
B2HRP5	Bromoperoxidase BpoC	5,8	**28,6**	262	0	NO_SP	1.000054	0.000000	0.000000	0.000000	0.000000	4	6	**1324110**	**30872**	**42,89**	**0,02**
A0A117DTI1	L-threonine dehydratase	4,4	**45,0**	426	0	NO_SP	1.000067	0.000000	0.000000	0.000000	0.000000	3	10	**1317476,667**	**7564**	**174,18**	**0,01**
A0A124BUA3	Anti-anti-sigma regulatory factor	4,7	**30,2**	273	0	NO_SP	1.000068	0.000000	0.000000	0.000000	0.000000	4	6	**1314016,667**	**0**	**#JAKO/0!**	**0,00**
A0A2Z5YGI3	Uncharacterized protein	4,9	**67,0**	619	0	NO_SP	1.000043	0.000001	0.000000	0.000000	0.000000	1	15	**1312256,667**	**8166**	**160,71**	**0,01**
B2HHN2	RNase H domain-containing protein	8,1	**40,6**	374	0	NO_SP	1.000030	0.000030	0.000001	0.000000	0.000000	4	11	**1308346,667**	**43243**	**30,26**	**0,03**
A0A2Z5YCM5	30S ribosomal protein S16	10,5	**17,4**	161	0	NO_SP	1.000048	0.000000	0.000000	0.000000	0.000000	1	5	**1303946,667**	**86845**	**15,01**	**0,07**
A0A3E2MQD9	Diacylglycerol O-acyltransferase	6,5	**48,2**	440	0	NO_SP	1.000065	0.000001	0.000000	0.000000	0.000000	4	10	**1300950**	**67268**	**19,34**	**0,05**
A0A3E2MRB3	Apolipoprotein N-acyltransferase	4,6	**73,6**	692	8	NO_SP	1.000051	0.000000	0.000000	0.000000	0.000000	4	9	**1297426,667**	**17339**	**74,83**	**0,01**
A0A2Z5YE78	Acetylornithine aminotransferase	5,3	**40,7**	396	0	NO_SP	1.000073	0.000000	0.000000	0.000000	0.000000	3	10	**1295086,667**	**0**	**#JAKO/0!**	**0,00**
A0A117DZ84	Cyclase	4,2	**15,9**	146	0	NO_SP	1.000035	0.000007	0.000000	0.000000	0.000000	3	2	**1293783,333**	**2974**	**435,02**	**0,00**
A0A2Z5YGD2	Uncharacterized protein	4,7	**274,6**	2641	0	NO_SP	1.000071	0.000000	0.000000	0.000000	0.000000	7	5	**1293003,333**	**31901**	**40,53**	**0,02**
B2HFB2	DNA helicase	5,8	**60,8**	549	0	NO_SP	1.000047	0.000004	0.000001	0.000000	0.000000	3	15	**1291496,667**	**10696**	**120,75**	**0,01**
A0A2Z5YD67	GTPase HflX	5,1	**53,6**	504	0	NO_SP	1.000054	0.000001	0.000000	0.000000	0.000000	3	16	**1288546,667**	**5185**	**248,53**	**0,00**
A0A2Z5Y7G9	Non-specific serine/threonine protein kinase	11,3	**47,4**	446	1	NO_SP	1.000061	0.000000	0.000000	0.000000	0.000000	4	9	**1286086,667**	**30235**	**42,54**	**0,02**
B2HDH8	Two-component transcriptional regulator TrcR	7,5	**28,7**	254	0	NO_SP	1.000052	0.000000	0.000000	0.000000	0.000000	4	7	**1284166,667**	**20273**	**63,34**	**0,02**
B2HJM0	Uncharacterized protein	4,3	**12,2**	101	0	NO_SP	1.000086	0.000000	0.000000	0.000000	0.000000	4	8	**1283496,667**	**9149**	**140,29**	**0,01**
B2HQ55	Rhamnosyl transferase WbbL2	8,0	**28,3**	255	0	NO_SP	1.000042	0.000002	0.000000	0.000000	0.000000	4	9	**1281760**	**48448**	**26,46**	**0,04**
B2HK30	Saccharopine dehydrogenase	4,6	**44,0**	405	0	NO_SP	0.999277	0.000554	0.000203	0.000002	0.000001	4	11	**1280050**	**11556**	**110,77**	**0,01**
A0A3E2N340	Anaerobic sulfite reductase subunit A	5,0	**40,5**	374	0	NO_SP	1.000042	0.000000	0.000000	0.000000	0.000000	4	7	**1274556,667**	**4377**	**291,22**	**0,00**
A0A2Z5YKZ9	Transcription-repair-coupling factor	5,2	**131,5**	1222	0	NO_SP	0.999926	0.000092	0.000001	0.000000	0.000000	4	19	**1272246,667**	**3583**	**355,13**	**0,00**
A0A2Z5YDC0	Enoyl-CoA hydratase	5,0	**29,2**	269	0	NO_SP	1.000107	0.000000	0.000000	0.000000	0.000000	4	7	**1268136,667**	**5908**	**214,66**	**0,00**
B2HH94	Uncharacterized protein	10,8	**13,4**	132	1	NO_SP	1.000037	0.000001	0.000000	0.000000	0.000000	4	4	**1262830**	**76240**	**16,56**	**0,06**
A0A2Z5YL48	Transcriptional regulatory protein PrrA	4,7	**25,3**	236	0	NO_SP	1.000047	0.000001	0.000000	0.000000	0.000000	3	7	**1261040**	**16716**	**75,44**	**0,01**
A0A100I3A9	TetR family transcriptional regulator	5,3	**19,7**	176	0	NO_SP	1.000055	0.000003	0.000000	0.000000	0.000000	4	7	**1259600**	**6851**	**183,85**	**0,00**
A0A2Z5YEK7	Putative transcriptional regulatory protein pdtaR	4,5	**23,0**	208	0	NO_SP	1.000058	0.000000	0.000000	0.000000	0.000000	1	9	**1255166,667**	**14968**	**83,86**	**0,01**
A0A3E2MWT1	Probable dual-specificity RNA methyltransferase RlmN	8,4	**39,7**	364	0	NO_SP	1.000060	0.000000	0.000000	0.000000	0.000000	2	9	**1245073,333**	**33667**	**36,98**	**0,03**
B2HQB5	Non-specific serine/threonine protein kinase	7,4	**53,4**	504	1	NO_SP	1.000058	0.000000	0.000000	0.000000	0.000000	5	6	**1241170**	**23085**	**53,77**	**0,02**
B2HT21	Transcriptional regulatory protein (Probably TetR-family)	5,4	**21,7**	195	0	NO_SP	1.000055	0.000001	0.000000	0.000000	0.000000	4	5	**1239893,333**	**24638**	**50,32**	**0,02**
A0A2Z5YDX9	Methionyl-tRNA formyltransferase	7,9	**32,6**	312	0	NO_SP	1.000045	0.000000	0.000000	0.000000	0.000000	4	7	**1236560**	**29295**	**42,21**	**0,02**
B2HLN9	Elongation factor G FusA2	4,7	**75,9**	718	0	NO_SP	1.000048	0.000014	0.000000	0.000000	0.000000	4	14	**1230026,667**	**992357**	**1,24**	**0,81**
A0A2Z5YDV1	Acyl-CoA synthetase	5,3	**58,8**	544	0	NO_SP	1.000031	0.000004	0.000000	0.000000	0.000000	3	10	**1220346,667**	**22378**	**54,53**	**0,02**
A0A124BW47	Non-ribosomal peptide synthetase	6,8	**138,2**	1306	5	NO_SP	1.000057	0.000000	0.000000	0.000000	0.000000	4	19	**1218923,333**	**21731**	**56,09**	**0,02**
B2HIG6	Acetohydroxy-acid synthase small subunit	9,8	**18,3**	169	0	NO_SP	1.000052	0.000000	0.000000	0.000000	0.000000	4	7	**1218563,333**	**13253**	**91,95**	**0,01**
A0A3E2MZU0	PGL/p-HBAD biosynthesis rhamnosyltransferase	9,4	**46,3**	420	0	NO_SP	1.000097	0.000000	0.000000	0.000000	0.000000	4	7	**1217520**	**77269**	**15,76**	**0,06**
B2HQV0	Short-chain type oxidoreductase	5,6	**26,1**	250	0	NO_SP	1.000034	0.000014	0.000001	0.000000	0.000000	4	6	**1216310**	**4050**	**300,33**	**0,00**
B2HT71	Mycothiol S-conjugate amidase	5,1	**32,7**	290	0	NO_SP	1.000041	0.000010	0.000000	0.000000	0.000000	4	10	**1212796,667**	**12862**	**94,29**	**0,01**
B2HJ72	Glycosyl transferase	8,2	**25,3**	233	0	NO_SP	1.000061	0.000000	0.000000	0.000000	0.000000	4	7	**1212506,667**	**57968**	**20,92**	**0,05**
A0A100I4V0	Arylamine N-acetyltransferase	4,9	**30,7**	280	0	NO_SP	1.000058	0.000000	0.000000	0.000000	0.000000	3	8	**1209336,667**	**27820**	**43,47**	**0,02**
A0A2Z5YCG7	Carrier domain-containing protein	4,7	**105,3**	981	0	NO_SP	1.000049	0.000000	0.000000	0.000000	0.000000	1	2	**1205106,667**	**39432**	**30,56**	**0,03**
A0A2Z5YDR7	ABC transporter ATP-binding protein	5,4	**28,0**	260	0	NO_SP	1.000026	0.000001	0.000000	0.000000	0.000000	3	8	**1203980**	**5078**	**237,11**	**0,00**
A0A2Z5YFU2	Mycobactin synthetase protein B	4,7	**50,9**	473	0	NO_SP	1.000050	0.000008	0.000001	0.000000	0.000000	5	7	**1203930**	**36841**	**32,68**	**0,03**
A0A2Z5Y839	Putative dioxygenase	5,3	**32,3**	289	0	NO_SP	1.000059	0.000000	0.000000	0.000000	0.000000	4	7	**1200863,333**	**3320**	**361,70**	**0,00**
A0A2Z5Y9V9	Uncharacterized protein	10,3	**59,0**	555	12	NO_SP	0.999937	0.000055	0.000000	0.000000	0.000000	4	6	**1199326,667**	**1799**	**666,79**	**0,00**
A0A2Z5YEK1	Lysine--tRNA ligase	7,4	**121,1**	1105	7	NO_SP	1.000028	0.000011	0.000000	0.000000	0.000000	3	15	**1194730**	**2394**	**499,00**	**0,00**
A0A2Z5YHL5	ABC transporter	4,1	**14,2**	139	0	NO_SP	1.000047	0.000000	0.000000	0.000000	0.000000	5	7	**1194086,667**	**1910**	**625,31**	**0,00**
A0A3E2MNW7	2,3-dehydroadipyl-CoA hydratase	5,6	**28,7**	266	0	NO_SP	1.000061	0.000000	0.000000	0.000000	0.000000	4	8	**1191880**	**8772**	**135,87**	**0,01**

A0A2Z5Y9V7	Hydrolase	5,3	**29,0**	280	0	NO_SP	1.000024	0.000036	0.000001	0.000000	0.000000	4	11	**1188026,667**	**964**	**1232,90**	**0,00**
A0A100I195	Uncharacterized protein	4,8	**103,8**	983	1	NO_SP	1.000049	0.000012	0.000000	0.000000	0.000000	1	1	**1183590**	**818400**	**1,45**	**0,69**
B2HPZ5	Serine/threonine-protein kinase PknG	5,1	**83,3**	767	0	NO_SP	1.000028	0.000004	0.000000	0.000000	0.000000	5	13	**1179100**	**35686**	**33,04**	**0,03**
A0A3E2MPD4	NAGPA domain-containing protein	8,5	**39,8**	375	0	SP	0.423205	0.513250	0.061821	0.000623	0.000446	4	10	**1178706,667**	**0**	**#JAKO/0!**	**0,00**
B2HJ53	GTP cyclohydrolase 1	7,4	**22,3**	202	0	NO_SP	1.000051	0.000027	0.000000	0.000000	0.000000	3	6	**1177143,333**	**75674**	**15,56**	**0,06**
A0A100HZQ6	Alcohol dehydrogenase B	4,8	**39,8**	375	0	NO_SP	1.000055	0.000001	0.000000	0.000000	0.000000	4	9	**1176800**	**3958**	**297,31**	**0,00**
A0A2Z5YFJ8	Carboxymuconolactone decarboxylase	4,4	**18,2**	164	0	NO_SP	1.000031	0.000004	0.000000	0.000000	0.000000	3	7	**1176403,333**	**5089**	**231,17**	**0,00**
A0A2Z5YCK9	3-isopropylmalate dehydratase small subunit	4,7	**21,8**	198	0	NO_SP	1.000044	0.000000	0.000000	0.000000	0.000000	4	8	**1175733,333**	**0**	**#JAKO/0!**	**0,00**
A0A2Z5Y9B0	Long-chain-fatty-acid--AMP ligase FadD30	5,7	**64,2**	589	0	NO_SP	1.000074	0.000002	0.000000	0.000000	0.000000	3	10	**1173020**	**25021**	**46,88**	**0,02**
A0A100HZC1	TobH protein	4,5	**37,6**	369	0	NO_SP	1.000030	0.000009	0.000000	0.000000	0.000000	2	11	**1170963,333**	**857**	**1366,83**	**0,00**
B2HKL5	5-methylthioadenosine/S-adenosylhomocysteine nucleosidase	4,6	**26,9**	251	0	NO_SP	1.000065	0.000002	0.000000	0.000000	0.000000	4	8	**1170723,333**	**113172**	**10,34**	**0,10**
B2HL18	Acyl-CoA dehydrogenase FadE20	5,0	**43,1**	386	0	NO_SP	1.000047	0.000000	0.000000	0.000000	0.000000	3	7	**1168973,333**	**12254**	**95,39**	**0,01**
A0A2Z5YDK0	TGc domain-containing protein	4,5	**34,8**	314	0	NO_SP	1.000053	0.000001	0.000000	0.000000	0.000000	5	8	**1165336,667**	**1786**	**652,63**	**0,00**
B2HDU8	Adenosine deaminase	4,9	**39,7**	362	0	NO_SP	1.000043	0.000000	0.000000	0.000000	0.000000	4	10	**1163623,333**	**1283**	**906,81**	**0,00**
A0A2Z5YIP6	Protein-glutamine gamma-glutamyltransferase	6,8	**31,0**	279	0	NO_SP	0.999888	0.000163	0.000006	0.000000	0.000000	3	7	**1163586,667**	**2700**	**430,94**	**0,00**
B2HND1	Cytoplasmic peptidase PepQ	5,7	**39,0**	372	0	NO_SP	1.000063	0.000003	0.000000	0.000000	0.000000	2	8	**1158990**	**12221**	**94,84**	**0,01**
A0A2Z5YG97	5-3 exonuclease	4,5	**32,3**	306	0	NO_SP	1.000069	0.000000	0.000000	0.000000	0.000000	2	8	**1157523,333**	**4858**	**238,27**	**0,00**
A0A2Z5YH06	Putative asparagine synthetase [glutamine-hydrolyzing]	6,5	**73,6**	664	0	SP	0.132043	0.866410	0.000655	0.000421	0.000254	4	12	**1156163,333**	**24626**	**46,95**	**0,00**
B2HS65	Succinyl-diaminopimelate desuccinylase DapE	4,9	**37,5**	354	0	NO_SP	1.000059	0.000001	0.000000	0.000000	0.000000	4	11	**1154610**	**8278**	**139,49**	**0,01**
A0A2Z5YJN4	ABC transporter ATP-binding protein	9,5	**85,3**	790	6	NO_SP	0.999913	0.000117	0.000005	0.000000	0.000000	4	12	**1154300**	**10146**	**113,77**	**0,01**
A0A117DYQ0	UPF0145 protein MPS_3990	4,8	**13,6**	127	0	NO_SP	1.000062	0.000002	0.000000	0.000000	0.000000	3	6	**1152733,333**	**0**	**#JAKO/0!**	**0,00**
B2HHJ2	ATP-binding protein ABC transporter	6,3	**34,7**	321	0	NO_SP	1.000022	0.000003	0.000000	0.000000	0.000000	2	15	**1144923,333**	**12640**	**90,58**	**0,01**
A0A2Z5Y9X7	1,4-dihydroxy-2-naphthoyl-CoA synthase	6,6	**33,4**	300	0	NO_SP	1.000079	0.000000	0.000000	0.000000	0.000000	3	11	**1144366,667**	**159**	**7194,41**	**0,00**
A0A2Z5YNN9	ABC transporter ATP-binding protein	6,0	**30,5**	280	0	NO_SP	1.000036	0.000000	0.000000	0.000000	0.000000	3	8	**1144103,333**	**6086**	**188,00**	**0,01**
A0A2Z5YGF8	Uncharacterized protein	5,3	**26,3**	248	0	NO_SP	1.000033	0.000023	0.000001	0.000000	0.000000	4	11	**1139423,333**	**2985**	**381,78**	**0,00**
A0A2Z5Y958	Carbon monoxide dehydrogenase subunit G (CoxG)	5,1	**24,0**	224	1	NO_SP	1.000074	0.000000	0.000000	0.000000	0.000000	3	6	**1135580**	**7623**	**148,97**	**0,01**
B2HSD6	Alpha-methylacyl-CoA racemase Mcr	5,4	**39,0**	365	0	NO_SP	1.000075	0.000000	0.000000	0.000000	0.000000	4	7	**1134160**	**0**	**#JAKO/0!**	**0,00**
A0A2Z5YCN7	DNA translocase FtsK	4,8	**86,1**	806	6	NO_SP	0.999720	0.000288	0.000000	0.000000	0.000000	4	11	**1131266,667**	**6813**	**166,05**	**0,01**
A0A2Z5YEZ5	Gamma-aminobutyraldehyde dehydrogenase	4,7	**52,1**	481	0	NO_SP	1.000060	0.000000	0.000000	0.000000	0.000000	4	7	**1130673,333**	**3659**	**309,04**	**0,00**
B2HQW6	Pyrroline-5-carboxylate reductase	4,6	**30,1**	294	0	NO_SP	1.000032	0.000000	0.000000	0.000000	0.000000	3	6	**1125766,667**	**9083**	**123,94**	**0,01**
B2HN27	Conserved membrane protein	9,8	**30,6**	283	0	NO_SP	0.999838	0.000160	0.000010	0.000001	0.000000	5	5	**1121816,667**	**6019**	**186,38**	**0,01**
A0A2Z5YMW8	D-alanyl-D-alanine carboxypeptidase DacB	8,3	**46,9**	461	1	NO_SP	0.987459	0.009459	0.000159	0.000098	0.000045	3	10	**1121200**	**15883**	**70,59**	**0,01**
B2HQT1	Uncharacterized protein	6,0	**30,2**	264	0	NO_SP	1.000083	0.000000	0.000000	0.000000	0.000000	3	7	**1120606,667**	**22856**	**49,03**	**0,02**
A0A3E2MWR3	Methionine synthase	4,5	**136,1**	1244	0	NO_SP	1.000070	0.000000	0.000000	0.000000	0.000000	4	19	**1120483,333**	**7481**	**149,79**	**0,01**
A0A2Z5YJT1	UDP-N-acetylglucosamine 1-carboxyvinyltransferase	4,8	**41,7**	392	0	NO_SP	0.999074	0.000964	0.000006	0.000001	0.000001	5	8	**1119493,333**	**3223**	**347,36**	**0,00**
A0A3E2N0V3	Uncharacterized protein	4,7	**52,3**	487	1	LIPO	0.000000	0.000040	1.000019	0.000000	0.000000	4	9	**1117963,333**	**11492**	**97,28**	**0,01**
A0A3E2MQ13	Phosphoglucomutase	5,2	**57,4**	543	0	NO_SP	1.000066	0.000003	0.000000	0.000000	0.000000	4	11	**1116576,667**	**0**	**#JAKO/0!**	**0,00**
A0A2Z5YNP0	Zinc-dependent alcohol dehydrogenase	5,4	**37,0**	353	0	NO_SP	1.000073	0.000001	0.000000	0.000000	0.000000	4	10	**1115670**	**0**	**#JAKO/0!**	**0,00**
B2HH14	Transcriptional regulatory protein	5,4	**28,8**	264	0	NO_SP	0.925033	0.074227	0.000347	0.000152	0.000084	4	6	**1112960**	**10898**	**102,12**	**0,01**
A0A2Z5YEC6	Polyketide synthase-like Pks10	4,5	**37,0**	353	0	NO_SP	1.000071	0.000000	0.000000	0.000000	0.000000	3	8	**1110540**	**11807**	**94,06**	**0,01**
B2HR12	Translation initiation factor IF-3	9,9	**18,9**	167	0	NO_SP	1.000048	0.000000	0.000000	0.000000	0.000000	4	4	**1108466,667**	**58485**	**18,95**	**0,05**
A0A2Z5Y8J2	DUF3533 domain-containing protein	8,4	**45,9**	435	6	NO_SP	0.995767	0.003960	0.000091	0.000013	0.000010	4	8	**1105700**	**27527**	**40,17**	**0,02**
B2HQB7	Peroxidoxin BcpB	7,2	**16,5**	152	0	NO_SP	1.000005	0.000040	0.000001	0.000000	0.000000	4	5	**1104833,333**	**10935**	**101,04**	**0,01**
B2HSN5	30S ribosomal protein S19	11,2	**10,8**	93	0	NO_SP	1.000046	0.000000	0.000000	0.000000	0.000000	3	4	**1103633,333**	**456493**	**2,42**	**0,41**
A0A2Z5YEP8	Uncharacterized protein	7,3	**28,5**	261	0	NO_SP	1.000038	0.000000	0.000000	0.000000	0.000000	3	6	**1097153,333**	**22381**	**49,02**	**0,02**
B2HQ26	Pyridoxal phosphate-dependent enzyme, WecE	6,2	**42,5**	382	0	NO_SP	0.999674	0.000316	0.000014	0.000003	0.000001	5	9	**1096080**	**25378**	**43,19**	**0,02**
A0A2Z5YH90	Protein PafC	4,4	**34,5**	322	0	NO_SP	1.000046	0.000001	0.000000	0.000000	0.000000	4	7	**1092996,667**	**11277**	**96,92**	**0,01**
B2HP92	Phosphoenolpyruvate carboxylase	5,3	**102,7**	935	0	NO_SP	1.000057	0.000000	0.000000	0.000000	0.000000	4	18	**1092963,333**	**8550**	**127,84**	**0,01**
A0A3E2MRQ8	Putative phenylalanine aminotransferase	6,7	**38,5**	361	0	NO_SP	1.000051	0.000004	0.000000	0.000000	0.000000	4	10	**1092163,333**	**11254**	**97,05**	**0,01**
A0A2Z5Y9R7	Glutamate-1-semialdehyde 2,1-aminomutase	5,4	**45,7**	443	0	NO_SP	0.995496	0.004461	0.000057	0.000008	0.000005	4	10	**1089953,333**	**3606**	**302,26**	**0,00**
B2HJ06	Acyl-CoA dehydrogenase	6,1	**43,2**	394	0	NO_SP	1.000039	0.000001	0.000000	0.000000	0.000000	4	6	**1088973,333**	**29490**	**36,93**	**0,03**
B2HEW2	Non-specific serine/threonine protein kinase	6,8	**69,7**	654	1	NO_SP	1.000026	0.000000	0.000000	0.000000	0.000000	6	11	**1084590**	**5206**	**208,33**	**0,00**
A0A2Z5YI92	Diacylglycerol O-acyltransferase	6,1	**49,8**	463	0	NO_SP	1.000046	0.000000	0.000000	0.000000	0.000000	3	10	**1079620**	**10345**	**104,36**	**0,01**
A0A2Z5YG29	Putative thiosulfate sulfurtransferase SseB	5,3	**29,9**	284	0	NO_SP	1.000038	0.000012	0.000000	0.000000	0.000000	4	9	**1079293,333**	**13861**	**77,87**	**0,01**
A0A2Z5YIS5	Cyclopropane mycolic acid synthase MmaA2	5,2	**33,2**	289	0	NO_SP	1.000056	0.000000	0.000000	0.000000	0.000000	4	4	**1078893,333**	**19172**	**56,28**	**0,02**
A0A2Z5YGZ7	Polyketide synthase	5,3	**54,3**	511	0	NO_SP	1.000044	0.000001	0.000000	0.000000	0.000000	4	10	**1078276,667**	**17321**	**62,25**	**0,02**
B2HQS9	Conserved protein	10,4	**21,2**	188	0	NO_SP	0.999595	0.000380	0.000005	0.000001	0.000001	3	6	**1077100**	**30662**	**35,13**	**0,03**
A0A2Z5YGN9	Alpha-L-fucosidase	5,9	**55,5**	489	0	NO_SP	1.000007	0.000007	0.000001	0.000000	0.000000	3	11	**1075686,667**	**9827**	**109,46**	**0,01**
A0A2Z5YLA9	VOC domain-containing protein	4,6	**16,1**	152	0	NO_SP	1.000061	0.000001	0.000000	0.000000	0.000000	3	4	**1074186,667**	**4686**	**229,22**	**0,00**
B2HRU7	Pseudouridine synthase	9,5	**27,7**	253	0	NO_SP	1.000038	0.000000	0.000000	0.000000	0.000000	3	7	**1073003,333**	**0**	**#JAKO/0!**	**0,00**
B2HME3	Phosphoglycerate mutase, Gpm_1	5,9	**24,2**	224	0	NO_SP	1.000054	0.000000	0.000000	0.000000	0.000000	4	9	**1067090**	**3240**	**329,38**	**0,00**
A0A100I595	Peptidyl-prolyl cis-trans isomerase	6,6	**18,5**	176	0	NO_SP	1.000052	0.000000	0.000000	0.000000	0.000000	4	4	**1066110**	**16229**	**65,69**	**0,00**
A0A2Z5YEE7	Polyketide synthase	4,9	**221,2**	2120	0	NO_SP	1.000057	0.000001	0.000000	0.000000	0.000000	4	23	**1065673,333**	**0**	**#JAKO/0!**	**0,00**
A0A2Z5YBA0	Putative sensor histidine kinase pdtaS	4,9	**53,7**	499	0	NO_SP	1.000025	0.000001	0.000000	0.000000	0.000000	3	10	**1061973,333**	**11434**	**92,88**	**0,01**
A0A2Z5YA53	Glyoxalase II	4,8	**25,4**	232	0	NO_SP	1.000066	0.000001	0.000000	0.000000	0.000000	4	5	**1059460**	**0**	**#JAKO/0!**	**0,00**
A0A3E2MVP6	Alpha-1,4-glucan:maltose-1-phosphate maltosyltransferase	5,2	**77,5**	695	0	NO_SP	1.000054	0.000003	0.000000	0.000000	0.000000	4	16	**1058326,667**	**8366**	**126,50**	**0,01**
A0A2Z5YDH4	Spermidine/putrescine ABC transporter ATP-binding protein	4,8	**34,3**	319	0	NO_SP	1.000048	0.000000	0.000000	0.000000	0.000000	4	9	**1055370**	**9748**	**108,27**	**0,01**
A0A2Z5YIQ4	N-acetyltransferase Eis	7,9	**40,5**	373	0	NO_SP	1.000045	0.000001	0.000000	0.000000	0.000000	4	11	**1053036,667**	**11584**	**90,90**	**0,01**
A0A2Z5YB21	Aldolase	5,0	**45,0**	427	0	NO_SP	1.000032	0.000021	0.000000	0.000000	0.000000	4	10	**1051826,667**	**568**	**1852,79**	**0,00**
A0A100IHD3	Uridylate kinase	7,3	**25,1**	235	0	NO_SP	1.000034	0.000000	0.000000	0.000000	0.000000	4	6	**1049876,667**	**28558**	**36,76**	**0,03**
A0A100IC31	Acyl dehydratase	6,1	**35,0**	330	0	NO_SP	1.000020	0.000023	0.000000	0.000000	0.000000	4	11	**1047370**	**0**	**#JAKO/0!**	**0,00**
A0A2Z5YNP7	MPT51/MPB51 antigen	6,1	**30,6**	298	1	SP	0.000316	0.999021	0.000164	0.000189	0.000147	4	5	**1044906,667**	**0**	**#JAKO/0!**	**0,00**
B2HCX0	50S ribosomal protein L36	11,6	**4,3**	37	0	NO_SP	1.000003	0.000000	0.000001	0.000000	0.000000	2	2	**1041710**	**319580**	**3,26**	**0,31**
B2HJW6	Pyruvate dehydrogenase E1 component subunit alpha	4,9	**36,6**	334	0	NO_SP	1.000047	0.000000	0.000000	0.000000	0.000000	4	7	**1041153,333**	**36683**	**28,38**	**0,04**
B2HH30	Amidophosphoribosyltransferase	5,1	**54,3**	508	0	NO_SP	1.000060	0.000000	0.000000	0.000000	0.000000	3	13	**1034923,333**	**9128**	**113,38**	**0,01**
A0A2Z5Y9W0	Geranylgeranyl pyrophosphate synthase	4,7	**35,7**	335	0	NO_SP	1.000063	0.000003	0.000000	0.000000	0.000000	3	10	**1034360**	**1720**	**601,28**	**0,00**
A0A2Z5Y9F4	Putative glutamate--cysteine ligase 2	4,9	**39,7**	355	0	NO_SP	1.000067	0.000000	0.000000	0.000000	0.000000	4	8	**1033296,667**	**13436**	**76,91**	**0,01**

A0A2Z5YNT3	Uncharacterized protein	12,0	**23,8**	218	0	NO_SP	1.000062	0.000000	0.000000	0.000000	0.000000	4	5	**1030890**	**54246**	**19,00**	**0,05**
A0A100HZS5	D-alanine--D-alanine ligase	4,6	**38,1**	358	0	NO_SP	1.000036	0.000004	0.000000	0.000000	0.000000	3	7	**1030600**	**9035**	**114,07**	**0,01**
A0A2Z5YI20	Allophanate hydrolase	4,7	**58,4**	568	0	NO_SP	1.000048	0.000001	0.000000	0.000000	0.000000	3	13	**1030410**	**5305**	**194,22**	**0,00**
B2HQW2	Conserved protein	5,3	**36,6**	344	0	NO_SP	1.000046	0.000000	0.000000	0.000000	0.000000	4	12	**1029300**	**12947**	**79,50**	**0,01**
A0A3E2MN12	CCA-adding enzyme	6,2	**53,6**	481	0	NO_SP	1.000073	0.000000	0.000000	0.000000	0.000000	3	9	**1027130**	**826**	**1243,25**	**0,00**
B2HMG5	Nucleoside diphosphate kinase	5,0	**14,7**	136	0	NO_SP	1.000066	0.000000	0.000000	0.000000	0.000000	3	6	**1010960**	**8621**	**117,27**	**0,01**
A0A2Z5YHS5	NADH oxidoreductase	5,1	**40,0**	385	0	NO_SP	1.000047	0.000002	0.000000	0.000000	0.000000	4	10	**1008796,667**	**808**	**1248,97**	**0,00**
B2HT51	Conserved protein	5,4	**27,9**	266	0	NO_SP	1.000015	0.000012	0.000000	0.000000	0.000000	3	5	**1008370**	**845**	**1193,48**	**0,00**
A0A2Z5YFR4	LuxR family transcriptional regulator	5,2	**94,9**	872	0	NO_SP	1.000018	0.000000	0.000000	0.000000	0.000000	7	15	**1004730**	**16615**	**60,47**	**0,02**
A0A3E2MW16	Succinyl-diaminopimelate desuccinylase	4,6	**46,1**	441	0	NO_SP	1.000085	0.000000	0.000000	0.000000	0.000000	4	9	**1003443,333**	**0**	**#JAKO/0!**	**0,00**
B2HQX3	Phosphoserine phosphatase SerB1	6,2	**32,2**	303	1	NO_SP	1.000054	0.000002	0.000000	0.000000	0.000000	4	7	**1003016,667**	**0**	**#JAKO/0!**	**0,00**
A0A2Z5YC21	Long-chain-fatty-acid--CoA ligase	4,7	**53,5**	495	0	NO_SP	1.000059	0.000001	0.000000	0.000000	0.000000	3	10	**1002090**	**21175**	**47,32**	**0,02**
B2HRF6	Oxidoreductase	4,3	**41,8**	378	0	NO_SP	0.999865	0.000133	0.000024	0.000000	0.000000	5	7	**1001146,667**	**0**	**#JAKO/0!**	**0,00**
A0A3E2MVF5	Histidinol-phosphate aminotransferase	5,2	**40,9**	382	0	NO_SP	1.000039	0.000000	0.000000	0.000000	0.000000	4	11	**1001010**	**1114**	**898,22**	**0,00**
A0A2Z5YCP4	PPOX class F420-dependent enzyme	7,4	**19,2**	173	0	NO_SP	1.000062	0.000002	0.000000	0.000000	0.000000	4	4	**998833,3333**	**73017**	**13,68**	**0,00**
A0A2Z5YEA6	RNA methyltransferase	7,0	**28,3**	275	0	NO_SP	1.000058	0.000000	0.000000	0.000000	0.000000	4	7	**998000**	**21109**	**47,28**	**0,02**
B2HPZ3	Acetate kinase	5,5	**41,6**	388	0	NO_SP	1.000065	0.000000	0.000000	0.000000	0.000000	4	9	**995463,3333**	**0**	**#JAKO/0!**	**0,00**
B2HM75	Conserved protein	6,2	**21,4**	190	0	NO_SP	1.000054	0.000000	0.000000	0.000000	0.000000	3	4	**994076,6667**	**4741**	**209,69**	**0,00**
A0A2Z5YEJ8	6-phosphogluconolactonase	4,5	**26,0**	247	0	NO_SP	1.000059	0.000001	0.000000	0.000000	0.000000	4	7	**994056,6667**	**2570**	**386,78**	**0,00**
A0A2Z5YM99	FMN_red domain-containing protein	4,9	**26,9**	244	0	NO_SP	1.000056	0.000000	0.000000	0.000000	0.000000	4	8	**991656,6667**	**13086**	**75,78**	**0,01**
B2HKV0	30S ribosomal protein S15	11,1	**10,4**	89	0	NO_SP	1.000066	0.000001	0.000000	0.000000	0.000000	3	4	**990513,3333**	**258477**	**3,83**	**0,26**
B2HJV3	Uncharacterized protein	5,2	**52,5**	482	0	NO_SP	1.000064	0.000000	0.000000	0.000000	0.000000	4	13	**988740**	**6140**	**161,02**	**0,01**
A0A2Z5YKE2	Uncharacterized protein	5,3	**63,9**	581	5	NO_SP	0.999975	0.000024	0.000000	0.000000	0.000000	4	6	**988710**	**16762**	**58,98**	**0,02**
B2HGV1	Uncharacterized protein	4,7	**42,5**	399	0	NO_SP	0.999966	0.000074	0.000005	0.000000	0.000000	4	10	**986330**	**1534**	**642,91**	**0,00**
B2HCW5	dTDP-4-dehydrorhamnose 3,5-epimerase	4,6	**22,1**	201	0	NO_SP	1.000061	0.000000	0.000000	0.000000	0.000000	4	6	**985293,3333**	**3154**	**312,42**	**0,00**
A0A3E2MPZ5	Glyoxalase-like domain protein	5,7	**31,1**	288	0	NO_SP	1.000061	0.000001	0.000000	0.000000	0.000000	4	9	**983093,3333**	**4155**	**236,58**	**0,00**
A0A2Z5YBI8	Sensor domain-containing protein	5,6	**24,8**	230	1	LIPO	0.001844	0.174203	0.823721	0.000176	0.000049	4	7	**982223,3333**	**1835**	**535,27**	**0,00**
A0A3E2MSS4	Alpha-keto-acid decarboxylase	4,5	**60,2**	566	0	NO_SP	1.000062	0.000001	0.000000	0.000000	0.000000	5	11	**981406,6667**	**0**	**#JAKO/0!**	**0,00**
A0A3E2MZE1	Metallo-beta-lactamase superfamily protein	5,0	**68,2**	626	0	NO_SP	1.000039	0.000000	0.000000	0.000000	0.000000	4	12	**979486,6667**	**5320**	**184,11**	**0,01**
B2HCX2	30S ribosomal protein S11	12,1	**14,7**	138	0	NO_SP	1.000049	0.000000	0.000000	0.000000	0.000000	3	3	**976846,6667**	**113864**	**8,58**	**0,12**
B2HN77	Peptidyl-prolyl cis-trans isomerase	9,7	**31,0**	295	1	NO_SP	0.999962	0.000069	0.000000	0.000000	0.000000	4	5	**975776,6667**	**28698**	**34,00**	**0,03**
A0A2Z5Y9F5	Hydroxymethylpyrimidine kinase	6,0	**27,9**	270	0	NO_SP	0.994627	0.005325	0.000018	0.000015	0.000008	2	8	**966716,6667**	**8212**	**117,72**	**0,01**
A0A2Z5YB18	Uncharacterized protein	4,6	**26,7**	246	0	NO_SP	1.000043	0.000000	0.000000	0.000000	0.000000	4	7	**964916,6667**	**0**	**#JAKO/0!**	**0,00**
A0A3E2MZG9	Acryloyl-CoA reductase (NADH)	5,4	**76,7**	721	0	NO_SP	1.000053	0.000000	0.000000	0.000000	0.000000	4	15	**963220**	**8063**	**119,47**	**0,01**
B2HDI2	Ppx-GppA domain-containing protein	4,6	**32,8**	315	0	NO_SP	1.000032	0.000002	0.000000	0.000000	0.000000	4	6	**962470**	**2115**	**455,05**	**0,00**
A0A3E2MYR8	Acyl-CoA dehydrogenase	4,8	**41,7**	390	0	NO_SP	1.000044	0.000000	0.000000	0.000000	0.000000	4	11	**959343,3333**	**1774**	**540,67**	**0,00**
A0A2Z5YBC1	Alcohol dehydrogenase	5,9	**34,0**	332	0	NO_SP	1.000055	0.000001	0.000000	0.000000	0.000000	3	7	**958923,3333**	**8269**	**115,97**	**0,01**
A0A2Z5YB59	Phosphomannomutase/phosphoglucomutase	4,4	**48,9**	466	0	NO_SP	1.000060	0.000000	0.000000	0.000000	0.000000	3	9	**958506,6667**	**4452**	**215,32**	**0,00**
B2HIY4	Electron transfer protein FdxB	6,4	**75,3**	673	4	NO_SP	0.999971	0.000054	0.000000	0.000000	0.000000	4	11	**957290**	**0**	**#JAKO/0!**	**0,00**
B2HMP0	Monooxygenase EthA	8,7	**54,9**	489	0	NO_SP	0.998558	0.000694	0.000759	0.000004	0.000002	4	9	**956253,3333**	**5731**	**166,85**	**0,01**
A0A2Z5YAK5	Cyclopropane mycolic acid synthase MmaA2	5,0	**32,9**	288	0	NO_SP	1.000046	0.000000	0.000000	0.000000	0.000000	8	9	**955013,3333**	**9275**	**102,97**	**0,01**
B2HEV5	Uncharacterized protein	5,9	**31,0**	284	0	NO_SP	1.000041	0.000004	0.000000	0.000000	0.000000	4	5	**954966,6667**	**23332**	**40,93**	**0,02**
A0A3E2MWE2	Putative succinyl-CoA:3-ketoacid coenzyme A transferase subunit B	4,7	**22,7**	215	0	NO_SP	1.000054	0.000000	0.000000	0.000000	0.000000	2	3	**952933,3333**	**18537**	**51,41**	**0,02**
A0A2Z5YCY1	NAD(P)H:quinone oxidoreductase type IV	5,8	**21,2**	200	0	NO_SP	1.000075	0.000000	0.000000	0.000000	0.000000	4	5	**952773,3333**	**134**	**7092,08**	**0,00**
B2HHS0	Glycerol-3-phosphate dehydrogenase	5,7	**53,7**	511	0	NO_SP	0.997408	0.002617	0.000010	0.000006	0.000002	4	11	**951676,6667**	**12474**	**76,29**	**0,01**
B2HR01	UvrABC system protein B	4,8	**81,1**	726	0	NO_SP	1.000077	0.000000	0.000000	0.000000	0.000000	4	15	**950383,3333**	**17816**	**53,34**	**0,02**
B2HJ46	Pantothenate synthetase	7,1	**33,3**	315	0	NO_SP	1.000068	0.000000	0.000000	0.000000	0.000000	4	8	**950306,6667**	**0**	**#JAKO/0!**	**0,00**
B2HLY0	Transcriptional repressor NrdR	6,9	**22,8**	205	0	NO_SP	1.000049	0.000006	0.000000	0.000000	0.000000	3	5	**948966,6667**	**9944**	**95,43**	**0,01**
A0A2Z5YBF7	Peptide chain release factor 2	4,5	**41,5**	371	0	NO_SP	1.000070	0.000000	0.000000	0.000000	0.000000	3	11	**944443,3333**	**0**	**#JAKO/0!**	**0,00**
B2HM54	Uncharacterized protein	4,8	**9,9**	93	0	NO_SP	1.000062	0.000001	0.000000	0.000000	0.000000	4	3	**943773,3333**	**19197**	**49,16**	**0,02**
A0A2Z5YHG4	Putative cystathionine beta-lyase	5,4	**43,1**	399	0	NO_SP	1.000057	0.000000	0.000000	0.000000	0.000000	4	10	**943246,6667**	**3366**	**280,26**	**0,00**
A0A2Z5YC66	tRNA-specific 2-thiouridylase MnmA	6,5	**37,6**	357	0	NO_SP	1.000019	0.000007	0.000000	0.000000	0.000000	4	12	**938940**	**10800**	**86,94**	**0,01**
B2HJJ5	Ribonuclease 3	5,0	**25,4**	241	0	NO_SP	1.000045	0.000000	0.000000	0.000000	0.000000	4	7	**937993,3333**	**0**	**#JAKO/0!**	**0,00**
B2HFI0	Conserved ATP-binding protein ABC transporter	6,9	**49,1**	447	0	NO_SP	1.000031	0.000000	0.000000	0.000000	0.000000	3	11	**937863,3333**	**0**	**#JAKO/0!**	**0,00**
A0A3E2MRE4	Diacylglycerol O-acyltransferase	9,0	**49,8**	452	0	NO_SP	1.000054	0.000001	0.000000	0.000000	0.000000	4	7	**936623,3333**	**14266**	**65,65**	**0,02**
A0A2Z5YD60	Histidine--tRNA ligase	4,9	**45,1**	420	0	NO_SP	1.000070	0.000000	0.000000	0.000000	0.000000	4	11	**935630**	**267243**	**3,50**	**0,29**
A0A2Z5Y7R8	Putative oxidoreductase	6,7	**79,2**	727	0	NO_SP	1.000085	0.000001	0.000000	0.000000	0.000000	4	13	**934650**	**2671**	**349,86**	**0,00**
B2HQL3	Peptide chain release factor 1	4,7	**39,0**	357	0	NO_SP	1.000056	0.000000	0.000000	0.000000	0.000000	4	6	**932050**	**0**	**#JAKO/0!**	**0,00**
A0A2Z5YD49	Carboxylesterase A	5,0	**53,9**	523	1	LIPO	0.000006	0.000161	0.999868	0.000000	0.000000	1	10	**931710**	**0**	**#JAKO/0!**	**0,00**
B2HGE2	NADH-quinone oxidoreductase	5,0	**85,2**	805	0	NO_SP	1.000040	0.000000	0.000000	0.000000	0.000000	3	15	**930733,3333**	**1483**	**627,43**	**0,00**
B2HQW9	UDP-glucose 4-epimerase GalE2	10,8	**40,7**	377	0	NO_SP	1.000066	0.000001	0.000000	0.000000	0.000000	4	9	**928816,6667**	**16088**	**57,73**	**0,02**
A0A2Z5YBA3	3-phosphoshikimate 1-carboxyvinyltransferase	5,4	**43,0**	416	0	NO_SP	1.000040	0.000002	0.000000	0.000000	0.000000	4	9	**928343,3333**	**60382**	**15,37**	**0,07**
A0A2Z5YDP6	Putative diacylated glycolipid transporter LprF	6,5	**35,1**	339	0	NO_SP	1.000070	0.000001	0.000000	0.000000	0.000000	4	7	**925043,3333**	**2677**	**345,57**	**0,00**
B2HFB0	Uncharacterized protein	4,1	**7,4**	69	0	NO_SP	1.000039	0.000002	0.000000	0.000000	0.000000	3	2	**922956,6667**	**0**	**#JAKO/0!**	**0,00**
B2HMD0	DNA polymerase III, delta subunit HolA	5,3	**34,2**	325	0	NO_SP	1.000054	0.000001	0.000000	0.000000	0.000000	4	11	**920210**	**2761**	**333,32**	**0,00**
A0A3E2MSQ1	Helicase_C_3 domain-containing protein	5,2	**79,4**	752	0	NO_SP	1.000043	0.000001	0.000000	0.000000	0.000000	4	14	**917230**	**27621**	**33,21**	**0,03**
B2HK82	Cysteine synthase CysK	5,1	**39,9**	368	0	NO_SP	1.000043	0.000001	0.000000	0.000000	0.000000	3	8	**916640**	**33746**	**27,16**	**0,04**
A0A2Z5Y8D7	S-adenosyl-L-methionine-dependent methyltransferase	4,7	**34,9**	313	0	NO_SP	1.000035	0.000003	0.000000	0.000000	0.000000	3	13	**915410**	**6206**	**147,50**	**0,01**
A0A2Z5YH07	Epimerase family protein	6,5	**31,9**	303	1	NO_SP	1.000017	0.000026	0.000000	0.000000	0.000000	3	6	**914870**	**7038**	**129,98**	**0,00**
B2HM64	Methyltransferase	10,2	**29,2**	274	0	NO_SP	1.000056	0.000001	0.000000	0.000000	0.000000	4	7	**914250**	**38262**	**23,89**	**0,04**
B2HJK2	Bifunctional uridylyltransferase/uridylyl-removing enzyme	5,4	**86,7**	812	0	NO_SP	1.000050	0.000000	0.000000	0.000000	0.000000	4	18	**913576,6667**	**9008**	**101,42**	**0,00**
A0A3E2MUI3	Arginine deiminase	5,0	**43,4**	402	0	NO_SP	1.000046	0.000000	0.000000	0.000000	0.000000	4	9	**913023,3333**	**1204**	**758,64**	**0,00**
A0A3E2MMM3	Isonitrile hydratase	4,6	**22,8**	212	0	NO_SP	1.000044	0.000000	0.000000	0.000000	0.000000	4	8	**912930**	**1915**	**476,71**	**0,00**
A0A2Z5YMF9	2-amino-3-ketobutyrate CoA ligase	6,0	**43,2**	414	0	NO_SP	1.000051	0.000001	0.000000	0.000000	0.000000	3	12	**912413,3333**	**3911**	**233,31**	**0,00**
B2HNK3	Citrate (Pro-3s)-lyase (Beta subunit) CitE	4,9	**29,0**	273	0	NO_SP	1.000036	0.000003	0.000000	0.000000	0.000000	4	6	**910660**	**6646**	**137,03**	**0,01**
A0A2Z5YKR3	Molybdenum cofactor biosynthesis protein	4,0	**18,4**	181	0	NO_SP	1.000038	0.000001	0.000000	0.000000	0.000000	3	6	**907516,6667**	**10577**	**85,80**	**0,01**
A0A2Z5YBZ4	3-isopropylmalate dehydratase large subunit	5,2	**50,4**	473	0	NO_SP	1.000047	0.000000	0.000000	0.000000	0.000000	4	11	**904566,6667**	**0**	**#JAKO/0!**	**0,00**

A0A3E2MY12	NAD(+) diphosphatase	4,6	33,4	308	0	NO_SP	1.000038	0.000006	0.000000	0.000000	0.000000	2	7	903900	6340	142,57	0,00
A0A2Z5YAU5	Phosphate-specific transport system accessory protein PhoU	5,0	24,2	221	0	NO_SP	1.000063	0.000002	0.000000	0.000000	0.000000	3	8	903160	1982	455,77	0,00
B2HHU5	Histidine kinase	6,3	51,2	481	2	NO_SP	0.809796	0.025841	0.036345	0.000373	0.000157	4	11	902180	10006	90,16	0,01
A0A2Z5YP48	ATPase	7,6	53,6	497	0	NO_SP	1.000049	0.000000	0.000000	0.000000	0.000000	1	9	900250	7283	123,60	0,01
A0A2Z5YJR0	Dihydrofolate synthase	4,3	50,6	484	0	NO_SP	1.000050	0.000000	0.000000	0.000000	0.000000	4	8	897733,3333	6339	141,62	0,01
A0A2Z5YNG8	Phosphatase PAP2 family protein	11,9	18,3	181	1	NO_SP	0.999903	0.000120	0.000000	0.000000	0.000000	4	5	896926,6667	19390	46,26	0,02
A0A2Z5Y8Q8	Diacylglycerol O-acyltransferase	6,7	52,8	479	0	NO_SP	1.000055	0.000000	0.000000	0.000000	0.000000	3	12	896553,3333	10186	88,02	0,01
A0A100I1R2	Uncharacterized protein	4,3	25,8	231	0	NO_SP	1.000062	0.000000	0.000000	0.000000	0.000000	3	6	894970	5762	155,31	0,01
A0A100ICJ8	Oxidoreductase	7,6	33,7	318	0	NO_SP	1.000045	0.000000	0.000000	0.000000	0.000000	4	10	892790	0	#JAKO/0!	0,00
B2HS22	Non-specific serine/threonine protein kinase	6,4	50,3	476	1	NO_SP	1.000032	0.000007	0.000001	0.000000	0.000000	4	7	891836,6667	4056	219,90	0,00
A0A3E2MPB8	Uncharacterized protein	4,0	28,2	267	1	NO_SP	1.000065	0.000001	0.000000	0.000000	0.000000	5	7	889560	8774	101,39	0,01
A0A2Z5YED9	Putative glycosyl hydrolase	5,6	135,2	1218	0	NO_SP	1.000062	0.000000	0.000000	0.000000	0.000000	4	17	887290	891	995,91	0,00
A0A2Z5YHD8	Transcriptional regulator	4,4	38,2	356	0	NO_SP	1.000062	0.000002	0.000000	0.000000	0.000000	4	7	881196,6667	4794	183,82	0,01
A0A100I065	Phosphoserine phosphatase	7,1	30,8	285	1	NO_SP	0.999895	0.000092	0.000021	0.000001	0.000000	1	7	880456,6667	744	1182,77	0,00
B2HRJ1	Glutamyl-tRNA reductase	6,3	49,2	473	0	NO_SP	1.000066	0.000002	0.000000	0.000000	0.000000	4	9	880250	61727	14,26	0,00
B2HPD3	UPF0353 protein MMAR_2288	9,5	35,9	335	3	NO_SP	1.000072	0.000001	0.000000	0.000000	0.000000	4	9	880116,6667	1639	536,98	0,00
A0A2Z5YD25	Limonene-1,2-epoxide hydrolase	6,7	16,1	143	0	NO_SP	1.000059	0.000000	0.000000	0.000000	0.000000	4	3	880026,6667	64346	13,68	0,07
A0A117DTQ6	Anti-sigma factor antagonist	4,3	11,7	113	0	NO_SP	1.000054	0.000000	0.000000	0.000000	0.000000	4	2	878813,3333	7503	117,13	0,01
B2HH42	29 kDa antigen Cfp29	4,5	29,0	268	0	NO_SP	1.000039	0.000000	0.000000	0.000000	0.000000	4	11	876506,6667	591	1483,34	0,00
A0A2Z5Y7N5	(R)-hydratase	5,4	36,2	334	0	NO_SP	1.000046	0.000003	0.000000	0.000000	0.000000	3	1	874910	7063	123,87	0,01
A0A2Z5YH91	Monoamine oxidase	8,2	48,2	450	0	NO_SP	1.000060	0.000000	0.000000	0.000000	0.000000	4	6	873770	14048	62,20	0,02
B2HFW5	tRNA (adenine(58)-N(1))-methyltransferase TrmI	7,6	30,4	280	0	NO_SP	1.000068	0.000001	0.000000	0.000000	0.000000	4	7	873470	4361	200,30	0,00
B2HMD7	Uncharacterized protein	5,0	36,8	349	0	NO_SP	1.000035	0.000000	0.000000	0.000000	0.000000	5	5	873153,3333	15625	55,88	0,02
A0A2Z5YDH3	Zinc metalloprotease	9,6	41,5	392	6	NO_SP	1.000032	0.000001	0.000000	0.000000	0.000000	4	6	871033,3333	9429	92,38	0,01
A0A2Z5YA90	Cytochrome c biogenesis protein	9,2	56,2	524	4	NO_SP	1.000004	0.000006	0.000000	0.000000	0.000000	3	9	869306,6667	149	5852,21	0,00
A0A3E2N2Y5	Acetolactate synthase	5,3	60,3	558	0	NO_SP	1.000044	0.000000	0.000000	0.000000	0.000000	4	8	868440	2288	379,63	0,00
B2HGT0	Conserved protein	4,7	32,9	298	0	NO_SP	1.000044	0.000001	0.000000	0.000000	0.000000	4	10	866736,6667	627	1382,06	0,00
A0A2Z5YB11	Aldehyde dehydrogenase (NAD(+))	4,8	52,8	504	0	NO_SP	1.000064	0.000000	0.000000	0.000000	0.000000	4	9	866306,6667	24181	35,83	0,03
A0A2Z5YG20	Pyridine nucleotide-disulfide oxidoreductase	6,2	50,4	467	0	NO_SP	1.000059	0.000002	0.000000	0.000000	0.000000	4	9	865980	7182	120,57	0,01
A0A2Z5YEW6	Imidazole glycerol phosphate synthase subunit HisF	4,5	26,7	261	0	NO_SP	1.000063	0.000000	0.000000	0.000000	0.000000	3	7	865520	2249	384,90	0,00
B2HR99	Uncharacterized protein	5,3	23,3	208	0	NO_SP	1.000064	0.000001	0.000000	0.000000	0.000000	4	6	863836,6667	4761	181,45	0,01
B2HSM1	L-lactate dehydrogenase (Cytochrome) LldD1	9,3	41,7	390	0	NO_SP	1.000050	0.000001	0.000000	0.000000	0.000000	4	13	861953,3333	10433	82,62	0,01
A0A3E2MNV9	Alkanal monooxygenase alpha chain	6,6	36,8	336	0	NO_SP	0.996783	0.002960	0.000164	0.000062	0.000015	4	5	861060	118117	7,29	0,14
A0A2Z5YNC0	Uncharacterized protein	4,5	24,8	242	0	NO_SP	0.989195	0.010763	0.000027	0.000018	0.000009	4	7	857150	6779	126,44	0,00
B2HD76	Uncharacterized protein	7,9	16,3	144	0	NO_SP	1.000044	0.000000	0.000000	0.000000	0.000000	4	5	856660	30350	28,23	0,04
B2HRQ2	Demethylmenaquinone methyltransferase	10,0	25,1	231	0	NO_SP	1.000022	0.000014	0.000000	0.000000	0.000000	3	8	856103,3333	0	#JAKO/0!	0,00
A0A3E2MZ11	Fatty acyl-CoA reductase	9,3	33,6	317	0	NO_SP	1.000057	0.000000	0.000000	0.000000	0.000000	4	7	853460	418602	2,04	0,49
B2HKC0	Histidine N-alpha-methyltransferase	4,6	35,4	321	0	NO_SP	1.000063	0.000000	0.000000	0.000000	0.000000	4	9	853003,3333	151	5638,70	0,00
A0A2Z5YL42	Toxin	8,7	15,9	148	0	NO_SP	1.000062	0.000000	0.000000	0.000000	0.000000	3	4	852623,3333	24891	34,25	0,03
B2HSF9	Cyanate hydratase	4,9	17,3	158	0	NO_SP	1.000063	0.000000	0.000000	0.000000	0.000000	3	5	852506,6667	12018	70,93	0,01
A0A100IFB7	Pantothenate kinase	9,5	34,5	302	0	NO_SP	1.000077	0.000000	0.000000	0.000000	0.000000	3	6	852033,3333	2912	292,58	0,00
B2HH22	Amino acid aminotransferase, PabC	6,2	31,4	296	0	NO_SP	1.000085	0.000000	0.000000	0.000000	0.000000	4	10	847676,6667	15970	53,08	0,02
A0A2Z5YAP4	ATP/GTP-binding protein	6,2	21,7	200	0	NO_SP	1.000045	0.000000	0.000000	0.000000	0.000000	3	9	845826,6667	0	#JAKO/0!	0,00
B2HHL4	Lipoyl synthase	6,7	36,0	324	0	NO_SP	1.000070	0.000000	0.000000	0.000000	0.000000	4	7	842950	1362	619,04	0,00
B2HR06	Conserved protein	4,6	24,1	225	0	NO_SP	1.000075	0.000001	0.000000	0.000000	0.000000	4	5	842476,6667	0	#JAKO/0!	0,00
A0A2Z5YD95	2,5-diamino-6-ribosylamino-4(3H)-pyrimidinone 5-phosphate reductase	5,4	27,7	260	0	NO_SP	0.999857	0.000195	0.000001	0.000000	0.000000	3	9	842233,3333	2706	311,24	0,00
A0A2Z5Y8Z7	Succinate dehydrogenase membrane anchor subunit	9,9	31,3	273	5	NO_SP	1.000032	0.000017	0.000000	0.000000	0.000000	3	2	840766,6667	20461	41,09	0,02
B2HGE0	NADH-quinone oxidoreductase subunit I	4,9	20,2	181	0	NO_SP	1.000035	0.000002	0.000000	0.000000	0.000000	4	6	839040	320501	2,62	0,38
A0A3E2MMQ8	N5-carboxyaminoimidazole ribonucleotide synthase	5,6	45,4	424	0	NO_SP	1.000050	0.000000	0.000000	0.000000	0.000000	4	6	838740	0	#JAKO/0!	0,00
B2HIM5	Daunorubicin-DIM-transport integral membrane protein ABC transporter DrrI	10,7	31,0	289	6	NO_SP	0.999925	0.000086	0.000000	0.000000	0.000000	4	5	838286,6667	14374	58,32	0,02
A0A2Z5YKV8	UTP--glucose-1-phosphate uridylyltransferase	4,7	32,2	305	0	NO_SP	0.999985	0.000057	0.000000	0.000000	0.000000	3	6	836173,3333	0	#JAKO/0!	0,00
A0A3E2MPT8	Redox sensor histidine kinase response regulator DevS	4,6	62,7	585	0	NO_SP	1.000067	0.000000	0.000000	0.000000	0.000000	4	13	834260	2951	282,71	0,00
A0A2Z5YCU5	Bifunctional oligoribonuclease and PAP phosphatase NrnA	4,5	33,8	324	0	NO_SP	1.000048	0.000004	0.000000	0.000000	0.000000	4	5	833813,3333	3883	214,75	0,00
A0A2Z5YJK3	Phosphohistidine phosphatase	9,0	21,1	198	0	NO_SP	0.999758	0.000303	0.000001	0.000001	0.000000	3	6	831610	4391	189,37	0,01
A0A2Z5YH52	Short-chain dehydrogenase	9,1	28,8	275	0	NO_SP	1.000029	0.000002	0.000000	0.000000	0.000000	4	7	830946,6667	11071	75,05	0,01
B2HFZ4	Conserved protein	4,7	48,8	451	0	NO_SP	1.000058	0.000001	0.000000	0.000000	0.000000	4	9	830703,3333	0	#JAKO/0!	0,00
A0A3E2MNN7	Uncharacterized protein	6,5	33,4	311	0	NO_SP	0.993406	0.006042	0.000458	0.000025	0.000012	4	9	827346,6667	10381	79,70	0,01
A0A124BUD8	Maltose alpha-D-glucosyltransferase	4,6	67,8	594	0	NO_SP	1.000056	0.000000	0.000000	0.000000	0.000000	3	9	826473,3333	11140	74,19	0,00
A0A3E2MY59	Uncharacterized protein	5,1	19,8	183	0	NO_SP	1.000000	0.000040	0.000000	0.000000	0.000000	4	4	825663,3333	37835	21,82	0,05
A0A2Z5YNX0	ESX-1 secretion system protein EccD1	6,5	54,3	511	11	NO_SP	0.966037	0.033805	0.000074	0.000047	0.000029	3	5	823180	12992	63,36	0,02
B2HLH0	Uncharacterized protein	4,9	22,1	199	0	NO_SP	1.000047	0.000000	0.000000	0.000000	0.000000	4	5	822890	0	#JAKO/0!	0,00
B2HJ20	Uncharacterized tRNA/rRNA methyltransferase MMAR_5079	10,9	32,5	310	0	NO_SP	1.000062	0.000000	0.000000	0.000000	0.000000	4	6	821156,6667	2820	291,27	0,00
B2HR65	NAD kinase	4,8	32,9	307	0	NO_SP	1.000061	0.000001	0.000000	0.000000	0.000000	4	5	820873,3333	8110	101,21	0,01
A0A2Z5YAS6	Allantoinase	6,4	33,3	297	0	NO_SP	1.000043	0.000001	0.000000	0.000000	0.000000	4	4	820623,3333	5307	154,62	0,00
A0A2Z5YBR8	Choline-sulfatase	5,7	66,8	603	0	NO_SP	1.000068	0.000001	0.000000	0.000000	0.000000	3	13	820300	2547	322,04	0,00
A0A3E2MZ54	Serine/threonine-protein kinase PknH	5,6	168,5	1584	0	NO_SP	1.000061	0.000000	0.000000	0.000000	0.000000	4	7	820253,3333	11313	72,51	0,01
A0A2Z5Y9Z9	Hydrolase	5,3	44,3	420	0	NO_SP	0.999812	0.000144	0.000039	0.000001	0.000000	4	8	816326,6667	0	#JAKO/0!	0,00
A0A2Z5YCV7	4-hydroxy-tetrahydrodipicolinate synthase	5,1	31,0	303	0	NO_SP	1.000029	0.000013	0.000000	0.000000	0.000000	4	6	813110	12291	66,16	0,00
B2HMG0	Glutamate 5-kinase	6,5	37,9	368	0	NO_SP	1.000057	0.000004	0.000000	0.000000	0.000000	3	7	812600	7237	112,29	0,01
A0A100IAL6	Histidine kinase	5,8	56,0	518	2	NO_SP	0.924237	0.052364	0.001492	0.000221	0.000157	3	10	810763,3333	57035	14,22	0,07
A0A2Z5YF64	Uncharacterized protein	8,5	22,7	209	0	NO_SP	0.999913	0.000130	0.000001	0.000000	0.000000	4	7	810660	11081	73,16	0,01
B2HKE0	Nucleoid-associated protein MMAR_5232	4,7	14,6	147	0	NO_SP	1.000048	0.000000	0.000000	0.000000	0.000000	3	3	808533,3333	25820	31,31	0,03
B2HKB1	Conserved membrane protein	11,8	47,5	440	0	NO_SP	0.995950	0.004004	0.000022	0.000009	0.000006	3	8	806830	7820	103,18	0,01
A0A3E2MYB2	Uncharacterized protein	4,2	36,8	352	0	NO_SP	1.000049	0.000001	0.000000	0.000000	0.000000	4	7	806433,3333	2576	313,11	0,00
A0A2Z5YJX1	Oxidoreductase	4,5	47,4	455	0	NO_SP	1.000040	0.000005	0.000000	0.000000	0.000000	3	10	805540	213	3786,50	0,00
A0A2Z5YB01	Alpha/beta hydrolase	10,5	28,9	279	0	NO_SP	0.999992	0.000055	0.000002	0.000000	0.000000	4	8	800263,3333	23517	34,03	0,03
B2HFJ0	SnoaL-like domain-containing protein	6,8	16,6	151	0	NO_SP	1.000012	0.000018	0.000001	0.000000	0.000000	4	5	800256,6667	414987	1,93	0,52

B2HSU7	Proline rich membrane-anchored mycosin MycP5	5,9	**59,8**	580	2	SP	0.001179	0.997669	0.000223	0.000451	0.000240	4	10	**799046,6667**	**0**	**#JAKO/0!**	**0,00**
B2HQJ9	Corrinoid adenosyltransferase	4,9	**21,2**	197	0	NO_SP	1.000051	0.000000	0.000000	0.000000	0.000000	4	5	**798396,6667**	**0**	**#JAKO/0!**	**0,00**
B2HK68	Membrane-associated serine protease	9,1	**41,2**	400	4	NO_SP	1.000030	0.000000	0.000000	0.000000	0.000000	4	7	**796503,3333**	**6363**	**125,18**	**0,01**
A0A2Z5YD82	TRAM domain-containing protein	7,1	**42,1**	401	0	NO_SP	0.998301	0.001693	0.000030	0.000004	0.000002	4	11	**792813,3333**	**1456**	**544,69**	**0,00**
A0A3E2MMT3	HTH-type transcriptional regulator BetI	5,2	**25,1**	224	0	NO_SP	1.000073	0.000000	0.000000	0.000000	0.000000	2	8	**792506,6667**	**2830**	**280,06**	**0,00**
A0A3E2MNP2	Magnesium-transporting ATPase, P-type 1	5,9	**87,1**	818	10	NO_SP	1.000068	0.000000	0.000000	0.000000	0.000000	4	8	**790116,6667**	**1428**	**553,21**	**0,00**
B2HL65	Transmembrane ATP-binding protein ABC transorter	6,9	**72,2**	690	4	NO_SP	0.999821	0.000205	0.000000	0.000000	0.000000	4	10	**784260**	**5924**	**132,38**	**0,01**
A0A100I8G7	N5,N10-methylene tetrahydromethanopterin reductase	5,1	**27,9**	255	0	NO_SP	1.000045	0.000014	0.000000	0.000000	0.000000	5	7	**780510**	**3907**	**199,79**	**0,01**
A0A2Z5Y7P8	Haloalkane dehalogenase	5,1	**36,4**	325	0	NO_SP	1.000065	0.000000	0.000000	0.000000	0.000000	3	8	**780376,6667**	**0**	**#JAKO/0!**	**0,00**
A0A100I3J2	Uncharacterized protein	4,7	**68,2**	609	0	NO_SP	1.000036	0.000000	0.000000	0.000000	0.000000	4	7	**779713,3333**	**345**	**2259,17**	**0,00**
A0A3E2MNS6	Long-chain-fatty-acid--CoA ligase FadD17	4,9	**57,8**	530	0	NO_SP	1.000066	0.000000	0.000000	0.000000	0.000000	4	9	**777676,6667**	**2894**	**268,73**	**0,00**
A0A100I4M1	Delta(24)-sterol reductase	6,6	**48,1**	426	0	NO_SP	1.000052	0.000001	0.000000	0.000000	0.000000	4	7	**777376,6667**	**12886**	**60,33**	**0,02**
A0A2Z5Y8H5	HTH-type transcriptional repressor KstR2	6,3	**23,3**	207	0	NO_SP	1.000059	0.000000	0.000000	0.000000	0.000000	3	7	**777360**	**4488**	**173,22**	**0,01**
A0A2Z5YMY6	DNA polymerase III subunit delta	6,8	**33,7**	318	0	NO_SP	1.000068	0.000000	0.000000	0.000000	0.000000	4	11	**775413,3333**	**10846**	**71,49**	**0,01**
B2HNI1	Cholesterol oxidase ChoD_1	7,2	**62,6**	579	0	NO_SP	1.000016	0.000020	0.000000	0.000000	0.000000	4	11	**775136,6667**	**5601**	**138,40**	**0,01**
A0A2Z5YII2	Probable molybdenum cofactor guanylyltransferase	5,0	**21,1**	201	0	NO_SP	1.000032	0.000001	0.000000	0.000000	0.000000	4	7	**773563,3333**	**3720**	**207,93**	**0,00**
A0A2Z5YPF2	Helicase ATP-binding domain-containing protein	6,2	**95,1**	869	0	NO_SP	1.000033	0.000004	0.000000	0.000000	0.000000	1	15	**769110**	**16219**	**47,42**	**0,02**
A0A2Z5YMW7	Uncharacterized protein	5,3	**46,5**	431	2	NO_SP	1.000034	0.000000	0.000000	0.000000	0.000000	4	6	**768796,6667**	**5713**	**134,56**	**0,01**
A0A2Z5YNB0	Probable hercynylcysteine sulfoxide lyase	4,5	**40,4**	383	0	NO_SP	0.998971	0.001058	0.000008	0.000002	0.000001	4	8	**768786,6667**	**0**	**#JAKO/0!**	**0,00**
A0A2Z5YK43	Magnesium transport protein CorA	5,3	**41,6**	366	2	NO_SP	1.000048	0.000003	0.000000	0.000000	0.000000	4	8	**766263,3333**	**6944**	**110,35**	**0,01**
B2HMP6	Immunogenic protein Mpt64	4,8	**25,3**	230	1	SP	0.000257	0.999163	0.000151	0.000154	0.000133	3	6	**766166,6667**	**20357**	**37,64**	**0,00**
A0A100I4E0	MCE associated membrane protein	4,3	**27,7**	264	1	NO_SP	1.000044	0.000001	0.000000	0.000000	0.000000	4	5	**765290**	**20510**	**37,31**	**0,03**
A0A2Z5YND5	Prephenate dehydrogenase	4,9	**30,3**	292	0	NO_SP	1.000023	0.000006	0.000000	0.000000	0.000000	4	6	**764816,6667**	**0**	**#JAKO/0!**	**0,00**
A0A2Z5YGQ5	UDP-N-acetylmuramoyl-L-alanyl-D-glutamate--2,6-diaminopimelate ligase	5,6	**53,0**	518	0	NO_SP	1.000005	0.000038	0.000001	0.000000	0.000000	4	11	**762693,3333**	**1611**	**473,44**	**0,00**
A0A2Z5YG95	Cobalamin biosynthesis protein CobN	5,0	**128,9**	1190	0	NO_SP	0.999969	0.000072	0.000000	0.000000	0.000000	4	15	**761960**	**124**	**6141,37**	**0,00**
A0A2Z5YA01	Oxidoreductase	4,8	**28,4**	270	0	NO_SP	1.000005	0.000046	0.000001	0.000000	0.000000	4	5	**761403,3333**	**5519**	**137,95**	**0,00**
B2HI63	Penicillin-binding protein PbpA	6,3	**51,4**	491	1	SP	0.230970	0.741600	0.025881	0.000488	0.000394	3	12	**761290**	**9124**	**83,44**	**0,01**
A0A2Z5YC01	PNPLA domain-containing protein	5,8	**61,7**	563	0	NO_SP	1.000066	0.000000	0.000000	0.000000	0.000000	4	9	**756530**	**2085**	**362,90**	**0,00**
B2HMG1	GTPase Obg	5,5	**50,6**	479	0	NO_SP	1.000045	0.000001	0.000000	0.000000	0.000000	4	4	**753603,3333**	**1166**	**646,26**	**0,00**
A0A2Z5YCU7	3-ketoacyl-ACP reductase	5,6	**26,9**	260	0	NO_SP	1.000045	0.000000	0.000000	0.000000	0.000000	3	7	**752633,3333**	**11568**	**65,06**	**0,02**
B2HM00	Conserved hypothetical secreted protein	5,0	**22,8**	217	1	NO_SP	0.999921	0.000096	0.000000	0.000000	0.000000	3	6	**751830**	**8498**	**88,47**	**0,01**
A0A2Z5Y8D8	Oxidoreductase	5,4	**46,8**	446	0	NO_SP	1.000042	0.000002	0.000000	0.000000	0.000000	4	11	**750073,3333**	**2178**	**344,31**	**0,00**
A0A2Z5Y895	Oxalyl-CoA decarboxylase	5,9	**61,1**	587	0	NO_SP	1.000038	0.000002	0.000000	0.000000	0.000000	4	12	**747846,6667**	**12184**	**61,38**	**0,02**
B2HP26	Glucose-1-phosphate thymidylyltransferase	5,4	**31,7**	288	0	NO_SP	1.000060	0.000000	0.000000	0.000000	0.000000	4	9	**746340**	**0**	**#JAKO/0!**	**0,00**
B2HEC0	Uncharacterized protein	9,3	**22,0**	200	0	NO_SP	1.000062	0.000013	0.000000	0.000000	0.000000	4	5	**745726,6667**	**6742**	**110,61**	**0,01**
A0A2Z5YKZ7	Dioxygenase	4,7	**57,3**	506	0	NO_SP	1.000086	0.000000	0.000000	0.000000	0.000000	3	9	**745080**	**303390**	**2,46**	**0,41**
B2HFC4	Uncharacterized protein	5,3	**15,9**	146	0	NO_SP	1.000075	0.000000	0.000000	0.000000	0.000000	4	6	**744920**	**12482**	**59,68**	**0,02**
B2HDC8	Conserved protein	10,3	**16,7**	155	0	NO_SP	1.000048	0.000002	0.000000	0.000000	0.000000	4	5	**743043,3333**	**32244**	**23,04**	**0,04**
A0A3E2MU13	DNA repair protein RecN	4,8	**61,2**	581	0	NO_SP	1.000009	0.000031	0.000000	0.000000	0.000000	4	10	**741453,3333**	**0**	**#JAKO/0!**	**0,00**
A0A2Z5Y8S2	PEP phosphonomutase and related enzymes	5,1	**27,0**	256	0	NO_SP	1.000031	0.000011	0.000000	0.000000	0.000000	4	8	**740706,6667**	**2400**	**308,65**	**0,00**
B2HCW9	Translation initiation factor IF-1	9,9	**8,5**	73	0	NO_SP	1.000076	0.000000	0.000000	0.000000	0.000000	3	3	**740236,6667**	**59756**	**12,39**	**0,08**
A0A3E2MVJ2	Dephospho-CoA kinase	6,2	**44,5**	408	0	NO_SP	0.999730	0.000296	0.000009	0.000000	0.000000	4	10	**739730**	**4849**	**152,55**	**0,01**
B2HM01	Conserved protein	4,0	**10,9**	100	0	NO_SP	1.000057	0.000001	0.000000	0.000000	0.000000	4	6	**739160**	**1128**	**655,42**	**0,00**
B2HIF0	DNA ligase	5,1	**75,1**	687	0	NO_SP	1.000062	0.000000	0.000000	0.000000	0.000000	3	11	**738280**	**0**	**#JAKO/0!**	**0,00**
A0A2Z5YI79	Dormancy associated translation inhibitor	10,7	**26,7**	244	0	NO_SP	0.906597	0.092838	0.000226	0.000144	0.000080	1	4	**737733,3333**	**30323**	**24,33**	**0,04**
A0A100I760	Peptide deformylase	4,8	**17,0**	158	0	NO_SP	1.000045	0.000001	0.000000	0.000000	0.000000	3	4	**737240**	**2907**	**253,64**	**0,00**
B2HQ31	Uncharacterized protein	4,7	**25,0**	212	0	NO_SP	1.000044	0.000000	0.000000	0.000000	0.000000	3	7	**736590**	**4637**	**158,85**	**0,01**
A0A2Z5YHX9	Phenazine biosynthesis protein PhzF	4,7	**29,9**	280	0	NO_SP	1.000057	0.000001	0.000000	0.000000	0.000000	4	5	**735900**	**9353**	**78,68**	**0,01**
A0A2Z5YEA7	Nucleotide-binding protein DAVIS_04514	6,7	**33,2**	306	0	NO_SP	0.999926	0.000131	0.000000	0.000000	0.000000	3	7	**735553,3333**	**0**	**#JAKO/0!**	**0,00**
B2HED9	Conserved secreted protein	8,1	**26,4**	249	0	NO_SP	1.000047	0.000000	0.000000	0.000000	0.000000	3	4	**735496,6667**	**18790**	**39,14**	**0,03**
A0A2Z5YDN9	Membrane protein	6,0	**38,7**	355	1	NO_SP	0.999907	0.000115	0.000008	0.000001	0.000000	4	10	**733273,3333**	**18723**	**39,16**	**0,03**
B2HFI1	Uncharacterized protein	5,6	**6,2**	57	0	NO_SP	1.000033	0.000000	0.000000	0.000000	0.000000	3	3	**732846,6667**	**18675**	**39,24**	**0,03**
A0A100I434	FHA domain containing protein	11,5	**15,6**	141	0	NO_SP	0.986801	0.012602	0.000471	0.000037	0.000023	3	4	**730790**	**25338**	**28,84**	**0,03**
A0A3E2MMK3	Acyltransferase papA3	7,5	**64,1**	588	0	NO_SP	1.000015	0.000012	0.000002	0.000000	0.000000	4	13	**730663,3333**	**34032**	**21,47**	**0,05**
A0A3E2MT15	Putative transcriptional regulatory protein NarL	7,0	**23,0**	216	0	NO_SP	1.000035	0.000000	0.000000	0.000000	0.000000	3	7	**729390**	**1866**	**390,96**	**0,00**
A0A2Z5YG73	Chromosome partitioning protein ParA	6,4	**29,3**	272	0	NO_SP	1.000046	0.000000	0.000000	0.000000	0.000000	4	8	**729046,6667**	**27760**	**26,26**	**0,04**
A0A3E2MSZ3	NmrA-like family protein	4,9	**26,0**	250	0	NO_SP	1.000017	0.000035	0.000002	0.000000	0.000000	1	6	**728163,3333**	**12709**	**57,30**	**0,02**
A0A2Z5YDU4	Cysteine desulfurase	5,7	**44,8**	417	0	NO_SP	1.000049	0.000000	0.000000	0.000000	0.000000	4	9	**727046,6667**	**0**	**#JAKO/0!**	**0,00**
A0A3E2MYE7	Uncharacterized protein	8,1	**24,7**	234	2	NO_SP	1.000064	0.000013	0.000000	0.000000	0.000000	4	7	**726420**	**1734**	**418,99**	**0,00**
B2HRA9	Sulfate adenylyltransferase subunit 2	6,6	**34,9**	310	0	NO_SP	1.000038	0.000000	0.000000	0.000000	0.000000	4	6	**725433,3333**	**6531**	**111,08**	**0,01**
A0A2Z5YII5	GTPase Era	6,4	**32,8**	300	0	NO_SP	1.000063	0.000000	0.000000	0.000000	0.000000	4	6	**725336,6667**	**11584**	**62,62**	**0,00**
B2HIS9	ATP-dependent 6-phosphofructokinase	5,8	**39,8**	377	0	NO_SP	1.000072	0.000002	0.000000	0.000000	0.000000	4	10	**724203,3333**	**11954**	**60,58**	**0,02**
A0A2Z5YLI0	Pyruvate, phosphate dikinase	4,6	**52,8**	497	0	NO_SP	1.000067	0.000000	0.000000	0.000000	0.000000	4	11	**723920**	**24813**	**29,17**	**0,03**
B2HL70	Acyl-CoA dehydrogenase	5,2	**71,8**	647	0	NO_SP	1.000063	0.000000	0.000000	0.000000	0.000000	4	12	**723413,3333**	**1427**	**506,90**	**0,00**
B2HHW3	Uncharacterized protein	4,6	**9,5**	87	0	NO_SP	1.000063	0.000000	0.000000	0.000000	0.000000	4	4	**721403,3333**	**31162**	**23,15**	**0,04**
B2HSQ0	Uncharacterized protein	9,7	**35,8**	332	0	NO_SP	0.999839	0.000193	0.000002	0.000000	0.000000	4	6	**720876,6667**	**13742**	**52,46**	**0,02**
A0A2Z5YDL4	dUTP diphosphatase	7,3	**12,2**	119	0	NO_SP	0.563428	0.409852	0.009067	0.011761	0.003521	4	5	**717686,6667**	**1291**	**555,87**	**0,00**
B2HLY3	Conserved hypothetical hydrolase	9,3	**36,9**	341	0	NO_SP	1.000018	0.000000	0.000000	0.000000	0.000000	3	9	**716840**	**14439**	**49,65**	**0,02**
A0A2Z5Y7I4	Uncharacterized protein	10,5	**26,0**	248	0	NO_SP	1.000047	0.000001	0.000000	0.000000	0.000000	4	5	**716546,6667**	**1862**	**384,85**	**0,00**
A0A3E2MTI3	Protein translocase subunit SecE	10,1	**17,3**	162	1	NO_SP	1.000061	0.000000	0.000000	0.000000	0.000000	4	6	**716420**	**2678**	**267,53**	**0,00**
A0A3E2MT77	Diacylglycerol O-acyltransferase	6,8	**49,8**	454	0	NO_SP	1.000050	0.000000	0.000000	0.000000	0.000000	4	10	**714526,6667**	**8354**	**85,53**	**0,01**
A0A100IAG4	Acid phosphatase	6,0	**22,0**	199	0	NO_SP	1.000039	0.000008	0.000000	0.000000	0.000000	3	6	**714520**	**1925**	**371,25**	**0,00**
B2HFZ1	Dihydroorotate dehydrogenase (quinone)	8,1	**37,9**	360	0	NO_SP	1.000067	0.000003	0.000000	0.000000	0.000000	4	6	**710223,3333**	**5065**	**140,21**	**0,00**
A0A2Z5Y8A2	Uncharacterized protein	5,6	**17,8**	161	0	NO_SP	0.999888	0.000139	0.000003	0.000000	0.000000	3	6	**709950**	**128**	**5566,34**	**0,00**
A0A2Z5YBE1	NADPH oxidoreductase	9,3	**41,4**	384	0	NO_SP	1.000051	0.000000	0.000000	0.000000	0.000000	3	8	**708923,3333**	**11377**	**62,31**	**0,00**
A0A2Z5YCZ5	Glycerol-3-phosphate dehydrogenase [NAD(P)+]	5,7	**34,2**	335	0	NO_SP	1.000051	0.000001	0.000000	0.000000	0.000000	3	8	**708696,6667**	**0**	**#JAKO/0!**	**0,00**
B2HKI0	Glutamine-transport ATP-binding protein ABC transporter	4,7	**35,9**	330	0	NO_SP	1.000041	0.000000	0.000000	0.000000	0.000000	3	8	**707910**	**1051**	**673,67**	**0,00**

B2HMU7	Conserved Mce associated transmembrane protein	7,0	**36,3**	329	3	NO_SP	1.000036	0.000003	0.000000	0.000000	0.000000	4	3	**707886,6667**	**12053**	**58,73**	**0,02**
A0A3E2N0M9	Membrane transport protein mmpL8	6,5	**109,2**	1000	12	NO_SP	0.999924	0.000084	0.000000	0.000000	0.000000	5	8	**707446,6667**	**2457**	**287,87**	**0,00**
A0A3E2MV92	Nitronate monooxygenase	5,7	**39,1**	370	0	NO_SP	1.000060	0.000000	0.000000	0.000000	0.000000	4	9	**706990**	**0**	**#JAKO/0!**	**0,00**
B2HP66	Riboflavin synthase alpha chain RibC	4,4	**21,9**	207	0	NO_SP	1.000034	0.000017	0.000000	0.000000	0.000000	4	7	**705713,3333**	**2161**	**326,59**	**0,00**
A0A117DZ92	PE-PGRS family protein	3,7	**31,5**	318	0	NO_SP	1.000064	0.000003	0.000000	0.000000	0.000000	4	3	**702810**	**51413**	**13,67**	**0,07**
B2HML7	Uncharacterized protein	9,7	**12,6**	109	0	NO_SP	1.000046	0.000000	0.000000	0.000000	0.000000	4	4	**701916,6667**	**10032**	**69,96**	**0,01**
A0A2Z5YFP9	Arylsulfatase	4,8	**85,3**	775	0	NO_SP	0.999479	0.000485	0.000065	0.000002	0.000001	5	9	**700773,3333**	**902**	**776,68**	**0,00**
A0A3E2MNC5	6-N-hydroxylaminopurine resistance protein	6,6	**23,7**	221	0	NO_SP	0.995133	0.004850	0.000025	0.000007	0.000004	4	6	**700386,6667**	**0**	**#JAKO/0!**	**0,00**
A0A2Z5YAJ4	Protein kinase domain-containing protein	6,6	**50,2**	453	0	NO_SP	1.000059	0.000000	0.000000	0.000000	0.000000	4	9	**699323,3333**	**62135**	**11,25**	**0,09**
A0A3E2MXT8	Succinyl-CoA:(R)-benzylsuccinate CoA-transferase subunit BbsF	5,3	**42,8**	401	0	NO_SP	1.000033	0.000005	0.000000	0.000000	0.000000	4	10	**698583,3333**	**0**	**#JAKO/0!**	**0,00**
A0A2Z5Y915	ESX-3 secretion-associated protein EspG3	4,5	**32,1**	298	0	NO_SP	1.000056	0.000002	0.000000	0.000000	0.000000	4	10	**697853,3333**	**13157**	**53,04**	**0,02**
A0A2Z5Y7J8	Uncharacterized protein	4,5	**27,5**	257	0	NO_SP	1.000010	0.000016	0.000000	0.000000	0.000000	1	6	**696796,6667**	**0**	**#JAKO/0!**	**0,00**
A0A100I966	cAMP-binding protein	4,7	**20,8**	190	0	NO_SP	1.000079	0.000000	0.000000	0.000000	0.000000	4	7	**695643,3333**	**5567**	**124,96**	**0,00**
A0A2Z5YDN8	Alpha-(1->3)-arabinofuranosyltransferase	9,7	**49,9**	436	8	NO_SP	0.999551	0.000436	0.000004	0.000001	0.000001	3	5	**694750**	**2956**	**235,03**	**0,00**
A0A3E2MWE9	Arylsulfatase	5,7	**87,7**	804	0	NO_SP	1.000051	0.000000	0.000000	0.000000	0.000000	4	10	**693746,6667**	**1115**	**621,95**	**0,00**
B2HGU9	1-acylglycerol-3-phosphate O-acyltransferase	9,8	**26,6**	243	0	SP	0.300725	0.696978	0.001505	0.000247	0.000230	3	4	**692840**	**22262**	**31,12**	**0,03**
B2HDL2	Ribosomal RNA small subunit methyltransferase I	5,1	**29,7**	284	0	NO_SP	0.999932	0.000110	0.000000	0.000000	0.000000	4	6	**692726,6667**	**6522**	**106,22**	**0,01**
B2HJA5	Uncharacterized protein	8,7	**38,3**	363	0	NO_SP	1.000050	0.000001	0.000000	0.000000	0.000000	3	8	**691633,3333**	**2986**	**231,65**	**0,00**
A0A2Z5YK61	Epoxide hydrolase	7,5	**33,3**	301	0	NO_SP	1.000036	0.000000	0.000000	0.000000	0.000000	3	8	**690863,3333**	**2459**	**280,90**	**0,00**
A0A2Z5YP73	S1 motif domain-containing protein	7,5	**49,7**	450	0	NO_SP	1.000050	0.000000	0.000000	0.000000	0.000000	1	9	**690610**	**0**	**#JAKO/0!**	**0,00**
A0A100I8T0	Biotin carboxylase-like protein	5,2	**53,9**	480	0	NO_SP	1.000042	0.000002	0.000000	0.000000	0.000000	4	11	**688610**	**798**	**862,99**	**0,00**
A0A2Z5YMW9	SIMPL domain-containing protein	4,6	**24,3**	235	0	LIPO	0.000000	0.000001	1.000070	0.000000	0.000000	4	5	**686646,6667**	**21470**	**31,98**	**0,03**
A0A2Z5YAB0	MDMPI_N domain-containing protein	7,4	**22,2**	209	0	NO_SP	0.999746	0.000272	0.000008	0.000001	0.000000	4	7	**685973,3333**	**10933**	**62,75**	**0,01**
B2HM08	Trk system potassium uptake protein TrkA	4,2	**23,7**	220	0	NO_SP	1.000062	0.000002	0.000000	0.000000	0.000000	3	4	**685306,6667**	**0**	**#JAKO/0!**	**0,00**
B2HDS9	Conserved membrane protein	7,1	**29,2**	266	0	NO_SP	1.000066	0.000001	0.000000	0.000000	0.000000	4	4	**685103,3333**	**17387**	**39,40**	**0,03**
A0A100I6V6	NADPH:adrenodoxin oxidoreductase FprA_1	5,1	**49,4**	456	0	NO_SP	1.000043	0.000000	0.000000	0.000000	0.000000	3	7	**684876,6667**	**0**	**#JAKO/0!**	**0,00**
B2HEB0	Mycobacterial persistence regulator MprA	4,8	**26,0**	232	0	NO_SP	1.000056	0.000000	0.000000	0.000000	0.000000	3	4	**684380**	**9467**	**72,29**	**0,01**
B2HNS4	Conserved hypothetical secreted protein	5,0	**83,5**	793	2	SP	0.002842	0.995736	0.000562	0.000333	0.000253	5	7	**683866,6667**	**316**	**2162,93**	**0,00**
A0A2Z5YLP7	Amidohydro-rel domain-containing protein	4,5	**47,2**	425	0	NO_SP	1.000028	0.000002	0.000000	0.000000	0.000000	4	7	**683630**	**7112**	**96,12**	**0,01**
B2HNQ0	PPE family protein	3,9	**37,2**	367	0	NO_SP	0.999867	0.000193	0.000002	0.000000	0.000000	4	3	**683206,6667**	**17155**	**39,83**	**0,03**
A0A2Z5YIK1	DNA primase	6,5	**71,2**	656	0	NO_SP	1.000050	0.000001	0.000000	0.000000	0.000000	3	10	**682043,3333**	**0**	**#JAKO/0!**	**0,00**
A0A2Z5YN68	Alpha/beta hydrolase	10,8	**36,0**	321	0	NO_SP	0.999931	0.000050	0.000015	0.000001	0.000000	4	8	**681996,6667**	**2015**	**338,53**	**0,00**
A0A2Z5YJI9	Uncharacterized protein	6,1	**29,9**	263	0	NO_SP	1.000041	0.000001	0.000000	0.000000	0.000000	2	7	**681870**	**2476**	**275,40**	**0,00**
A0A2Z5YE69	Succinylglutamate desuccinylase	5,6	**38,3**	363	0	NO_SP	1.000063	0.000000	0.000000	0.000000	0.000000	3	10	**678390**	**5411**	**125,37**	**0,01**
A0A2Z5YN76	Glutamate--cysteine ligase EgtA	4,6	**45,1**	429	0	NO_SP	0.867857	0.131091	0.000299	0.000332	0.000171	4	9	**678023,3333**	**273**	**2486,27**	**0,00**
B2HH33	Lipoprotein	4,7	**44,7**	423	0	LIPO	0.000000	0.000003	1.000076	0.000000	0.000000	2	11	**677510**	**14241**	**47,57**	**0,02**
A0A2Z5YCC3	Low molecular weight protein antigen 6	11,6	**12,3**	115	1	NO_SP	0.999608	0.000421	0.000002	0.000001	0.000001	4	3	**677373,3333**	**6339**	**106,86**	**0,01**
A0A2Z5YN88	Ubiquinone biosynthesis O-methyltransferase	4,6	**24,7**	232	0	NO_SP	1.000043	0.000002	0.000000	0.000000	0.000000	3	5	**677243,3333**	**0**	**#JAKO/0!**	**0,00**
B2HJC8	Zinc-containing alcohol dehydrogenase NAD-dependent, AdhD_1	5,6	**38,7**	369	0	NO_SP	1.000056	0.000000	0.000000	0.000000	0.000000	4	8	**677170**	**329**	**2059,92**	**0,00**
B2HPV1	Glycosyl hydrolase	7,0	**40,9**	371	0	NO_SP	1.000055	0.000001	0.000000	0.000000	0.000000	4	6	**676046,6667**	**0**	**#JAKO/0!**	**0,00**
B2HQW1	Uncharacterized protein	5,8	**29,4**	264	0	NO_SP	1.000060	0.000001	0.000000	0.000000	0.000000	4	9	**675790**	**662**	**1020,37**	**0,00**
A0A2Z5YN11	Endonuclease III	7,1	**24,9**	226	0	NO_SP	1.000075	0.000001	0.000000	0.000000	0.000000	4	6	**675216,6667**	**14293**	**47,24**	**0,02**
B2HPY9	Polyketide synthase Pks6	5,0	**252,3**	2414	0	NO_SP	1.000042	0.000002	0.000000	0.000000	0.000000	3	7	**671690**	**0**	**#JAKO/0!**	**0,00**
A0A2Z5Y9L4	Uncharacterized protein	4,2	**37,6**	356	3	NO_SP	1.000071	0.000000	0.000000	0.000000	0.000000	4	3	**671403,3333**	**0**	**#JAKO/0!**	**0,00**
A0A2Z5YDV7	Uncharacterized protein	5,6	**30,6**	273	0	NO_SP	1.000076	0.000000	0.000000	0.000000	0.000000	4	7	**670720**	**4236**	**158,35**	**0,01**
A0A2Z5YBL8	Histidinol-phosphatase	4,8	**28,1**	262	0	NO_SP	1.000070	0.000001	0.000000	0.000000	0.000000	3	5	**669000**	**3637**	**183,93**	**0,00**
A0A100I111	Benzoquinone methyltransferase	4,7	**23,7**	223	0	NO_SP	1.000076	0.000002	0.000000	0.000000	0.000000	4	2	**668660**	**0**	**#JAKO/0!**	**0,00**
A0A3E2MQ23	Putative ABC transporter ATP-binding protein YjjK	6,0	**64,5**	589	0	NO_SP	0.999990	0.000051	0.000003	0.000000	0.000000	1	9	**667163,3333**	**228**	**2926,63**	**0,00**
A0A2Z5YJK4	Mycobactin synthetase protein B	4,7	**182,2**	1670	0	NO_SP	1.000074	0.000001	0.000000	0.000000	0.000000	4	18	**665873,3333**	**24264**	**27,44**	**0,00**
A0A2Z5YMJ7	Phosphoserine aminotransferase	4,8	**43,0**	405	0	NO_SP	0.992281	0.007687	0.000020	0.000013	0.000009	1	1	**665603,3333**	**0**	**#JAKO/0!**	**0,00**
A0A2Z5YIG9	Ribosomal silencing factor RsfS	4,3	**14,2**	129	0	NO_SP	1.000070	0.000000	0.000000	0.000000	0.000000	3	4	**664116,6667**	**14567**	**45,59**	**0,02**
A0A2Z5YDI0	Adenine phosphoribosyltransferase	4,6	**18,9**	184	0	NO_SP	1.000054	0.000000	0.000000	0.000000	0.000000	4	5	**663660**	**0**	**#JAKO/0!**	**0,00**
A0A2Z5YAD1	50S ribosomal protein L29	4,9	**9,2**	80	0	NO_SP	1.000069	0.000000	0.000000	0.000000	0.000000	3	5	**662320**	**97321**	**6,81**	**0,15**
A0A2Z5YEM0	Phosphatase	4,6	**35,1**	336	0	NO_SP	1.000054	0.000000	0.000000	0.000000	0.000000	3	5	**662130**	**2930**	**225,99**	**0,00**
A0A2Z5YC90	Uncharacterized protein	7,1	**45,5**	438	0	LIPO	0.000036	0.000500	0.999477	0.000009	0.000002	4	7	**660730**	**242**	**2731,53**	**0,00**
A0A2Z5YIM0	Uncharacterized protein	6,2	**23,9**	220	0	NO_SP	1.000036	0.000000	0.000000	0.000000	0.000000	4	6	**660600**	**0**	**#JAKO/0!**	**0,00**
A0A3E2MQ52	Uncharacterized protein	8,8	**68,6**	634	9	SP	0.422634	0.571438	0.002735	0.000864	0.000693	5	8	**659333,3333**	**6883**	**95,79**	**0,01**
B2HGQ5	Conserved protein	9,9	**34,1**	313	0	NO_SP	1.000043	0.000001	0.000000	0.000000	0.000000	3	4	**657253,3333**	**5029**	**130,69**	**0,01**
A0A2Z5Y7Y4	L-serine dehydratase	4,8	**48,2**	461	0	NO_SP	1.000045	0.000001	0.000000	0.000000	0.000000	4	7	**656703,3333**	**5831**	**112,62**	**0,01**
B2HMK4	Conserved transmembrane protein	9,7	**70,1**	639	10	NO_SP	0.996958	0.000117	0.000042	0.000001	0.000000	4	6	**655630**	**8864**	**73,97**	**0,01**
A0A2Z5Y7G0	DUF3566 domain-containing protein	8,5	**30,8**	300	2	NO_SP	1.000022	0.000005	0.000000	0.000000	0.000000	3	5	**655196,6667**	**1704**	**384,45**	**0,00**
B2HM10	Conserved integral membrane alanine, valine and leucine rich protein	10,2	**71,8**	665	11	NO_SP	0.999607	0.000385	0.000001	0.000000	0.000000	4	4	**654800**	**2987**	**219,18**	**0,00**
A0A2Z5YAW1	Bifunctional NAD(P)H-hydrate repair enzyme	6,0	**47,2**	473	0	NO_SP	1.000061	0.000002	0.000000	0.000000	0.000000	4	7	**654570**	**1826**	**358,51**	**0,00**
A0A2Z5YF07	Uncharacterized protein	11,1	**15,1**	136	0	NO_SP	1.000068	0.000000	0.000000	0.000000	0.000000	3	5	**653480**	**149153**	**4,38**	**0,23**
B2HLB3	Amidase AmiA2	9,1	**52,3**	499	0	NO_SP	1.000056	0.000000	0.000000	0.000000	0.000000	4	9	**653220**	**3379**	**193,31**	**0,01**
A0A3E2MX17	Protease 3	7,4	**47,8**	438	0	NO_SP	1.000058	0.000002	0.000000	0.000000	0.000000	3	8	**651823,3333**	**14093**	**46,25**	**0,02**
B2HIA3	Short-chain type oxidoreductase	4,7	**23,3**	221	0	NO_SP	0.999990	0.000025	0.000000	0.000000	0.000000	4	4	**651683,3333**	**17443**	**37,36**	**0,03**
B2HSC6	Conserved protein	5,7	**16,3**	147	0	NO_SP	1.000046	0.000000	0.000000	0.000000	0.000000	3	7	**650656,6667**	**7867**	**82,70**	**0,00**
A0A2Z5YH77	Cytochrome c oxidase subunit 1	7,5	**64,1**	577	12	NO_SP	0.999981	0.000031	0.000000	0.000000	0.000000	3	3	**650310**	**29872**	**21,77**	**0,05**
A0A2Z5YKC1	RDD domain-containing protein	8,7	**25,2**	237	2	NO_SP	1.000044	0.000016	0.000000	0.000000	0.000000	4	4	**649766,6667**	**0**	**#JAKO/0!**	**0,00**
A0A2Z5YEL6	Uncharacterized protein	9,2	**70,4**	628	8	NO_SP	1.000062	0.000000	0.000000	0.000000	0.000000	4	6	**648493,3333**	**27351**	**23,71**	**0,04**
A0A2Z5YIV0	NAD-reducing hydrogenase HoxS subunit delta	5,9	**26,8**	251	0	NO_SP	1.000041	0.000001	0.000000	0.000000	0.000000	3	5	**647233,3333**	**5652**	**114,52**	**0,01**
A0A2Z5Y993	Thiosulfate sulfurtransferase GlpE	4,6	**15,0**	142	0	NO_SP	1.000036	0.000000	0.000000	0.000000	0.000000	3	3	**646566,6667**	**0**	**#JAKO/0!**	**0,00**
B2HSR6	CMD domain-containing protein	5,9	**12,6**	120	0	NO_SP	1.000041	0.000001	0.000000	0.000000	0.000000	3	4	**645500**	**0**	**#JAKO/0!**	**0,00**
A0A2Z5Y8C2	Oxidoreductase	8,7	**37,2**	340	0	NO_SP	1.000062	0.000000	0.000000	0.000000	0.000000	4	8	**645263,3333**	**12112**	**53,28**	**0,02**
B2HDI7	MazG domain-containing protein	4,4	**39,7**	366	0	NO_SP	1.000058	0.000018	0.000001	0.000000	0.000000	5	4	**644933,3333**	**2447**	**263,51**	**0,00**
B2HQT9	Transcriptional regulatory protein	5,0	**15,1**	138	0	NO_SP	1.000062	0.000000	0.000000	0.000000	0.000000	3	3	**644320**	**7707**	**83,60**	**0,01**

B2HNG9	DNA-(apurinic or apyrimidinic site) lyase	9,9	29,2	268	0	NO_SP	1.000040	0.000001	0.000000	0.000000	0.000000	4	4	643690	28791	22,36	0,04
B2HQV6	Two-component sensory transduction protein RegX3	4,6	24,8	227	0	NO_SP	1.000074	0.000000	0.000000	0.000000	0.000000	4	8	642726,6667	0	#JAKO/0!	0,00
B2HF55	Putative_PNPOx domain-containing protein	10,6	14,2	129	0	NO_SP	1.000082	0.000000	0.000000	0.000000	0.000000	4	5	641910	33779	19,00	0,05
A0A2Z5YDU1	Esterase	10,5	47,0	425	0	NO_SP	0.999463	0.000503	0.000028	0.000003	0.000001	4	10	641723,3333	11678	54,95	0,02
A0A3E2MZ53	Glutamate synthase [NADPH] small chain	5,9	61,4	561	0	NO_SP	1.000044	0.000004	0.000000	0.000000	0.000000	4	11	639620	0	#JAKO/0!	0,00
A0A3E2N328	Putative enoyl-CoA hydratase echA8	6,5	27,8	258	0	NO_SP	1.000044	0.000000	0.000000	0.000000	0.000000	4	6	638396,6667	0	#JAKO/0!	0,00
A0A2Z5Y8K1	Alcohol dehydrogenase	6,0	38,8	380	0	NO_SP	1.000063	0.000000	0.000000	0.000000	0.000000	4	8	637200	0	#JAKO/0!	0,00
A0A2Z5YHR6	Fatty-acid--CoA ligase	6,3	66,1	615	0	NO_SP	1.000022	0.000030	0.000002	0.000000	0.000000	4	6	636553,3333	7845	81,15	0,01
A0A2Z5YP92	Uncharacterized protein	5,6	61,0	546	0	NO_SP	1.000081	0.000002	0.000000	0.000000	0.000000	1	9	635620	11396	55,78	0,00
B2HQU8	UDP-N-acetylenolpyruvoylglucosamine reductase	5,5	38,2	366	0	NO_SP	0.999903	0.000148	0.000000	0.000000	0.000000	5	8	635586,6667	1258	505,30	0,00
B2HJM8	Mycobactin utilization protein ViuB	5,6	30,6	282	0	NO_SP	1.000057	0.000001	0.000000	0.000000	0.000000	4	6	634863,3333	0	#JAKO/0!	0,00
A0A100I3S8	Sugar-transport ATP-binding protein ABC transporter SugC	5,9	42,7	391	0	NO_SP	1.000069	0.000000	0.000000	0.000000	0.000000	4	8	633343,3333	0	#JAKO/0!	0,00
B2HML2	Acyltransferase	9,9	28,9	261	0	NO_SP	1.000033	0.000001	0.000000	0.000000	0.000000	4	5	632436,6667	3447	183,50	0,01
A0A2Z5YD79	RNA polymerase sigma factor	6,7	36,4	323	0	NO_SP	1.000049	0.000003	0.000000	0.000000	0.000000	4	7	629446,6667	3778	166,59	0,00
A0A2Z5YEF5	Imidazole glycerol phosphate synthase subunit HisH	7,8	22,0	210	0	NO_SP	1.000047	0.000000	0.000000	0.000000	0.000000	3	6	628906,6667	17242	36,47	0,03
A0A100HZF9	Glycogen debranching enzyme	4,7	80,8	719	0	NO_SP	1.000021	0.000038	0.000002	0.000000	0.000000	4	9	628843,3333	12588	49,96	0,02
B2HM81	Uncharacterized protein	8,5	12,0	104	0	NO_SP	1.000043	0.000000	0.000000	0.000000	0.000000	4	3	626866,6667	265	2362,56	0,00
A0A3E2MQN5	Carboxylic acid reductase	4,7	128,0	1174	0	NO_SP	1.000080	0.000000	0.000000	0.000000	0.000000	4	14	625776,6667	0	#JAKO/0!	0,00
B2HNE6	Aspartate carbamoyltransferase	6,6	33,8	320	0	NO_SP	1.000036	0.000004	0.000000	0.000000	0.000000	4	7	624920	7943	78,67	0,01
A0A2Z5YG56	Mercuric reductase	5,4	48,9	459	0	NO_SP	1.000091	0.000001	0.000000	0.000000	0.000000	4	10	624813,3333	2465	253,50	0,00
B2HEK8	Biotin--protein ligase	5,4	28,9	276	0	NO_SP	1.000077	0.000000	0.000000	0.000000	0.000000	4	5	623840	12608	49,48	0,02
B2HNS9	Thioredoxin	4,4	12,5	117	0	NO_SP	1.000056	0.000001	0.000000	0.000000	0.000000	3	3	620336,6667	4552	136,27	0,00
B2HHG1	Transcriptional regulatory protein (Probably AsnC-family)	6,9	27,9	247	1	NO_SP	1.000067	0.000001	0.000000	0.000000	0.000000	2	6	619920	11443	54,17	0,02
B2HKW0	Short-chain dehydrogenase EphD_1	9,3	61,8	586	0	NO_SP	1.000071	0.000000	0.000000	0.000000	0.000000	4	10	619410	0	#JAKO/0!	0,00
B2HCX7	D-serine/D-alanine/glycine transporter, CycA	7,8	52,6	491	12	NO_SP	1.000028	0.000003	0.000000	0.000000	0.000000	4	2	619246,6667	287	2154,73	0,00
A0A3E2MUG0	50S ribosomal protein L25	4,2	22,3	214	0	NO_SP	0.999987	0.000050	0.000001	0.000000	0.000000	4	4	617583,3333	53838	11,47	0,09
B2HDK2	Deoxyribonuclease TatD	5,0	30,6	282	0	NO_SP	0.991809	0.008179	0.000024	0.000010	0.000006	4	7	616816,6667	225	2747,31	0,00
B2HGE5	NADH-quinone oxidoreductase subunit D	4,8	47,5	432	0	NO_SP	1.000046	0.000000	0.000000	0.000000	0.000000	4	8	616493,3333	3374	182,72	0,00
B2HIW7	Hydrolase	4,8	36,3	333	0	NO_SP	1.000031	0.000000	0.000000	0.000000	0.000000	3	6	615560	20033	30,73	0,03
A0A124BW62	4-diphosphocytidyl-2-C-methyl-D-erythritol kinase	5,4	31,9	309	0	NO_SP	1.000058	0.000000	0.000000	0.000000	0.000000	3	10	615086,6667	0	#JAKO/0!	0,00
A0A2Z5YJJ4	Thioesterase	5,1	15,0	144	0	NO_SP	1.000057	0.000001	0.000000	0.000000	0.000000	5	4	614530	0	#JAKO/0!	0,00
A0A3E2MT87	Uncharacterized protein	4,8	53,1	481	0	NO_SP	1.000048	0.000000	0.000000	0.000000	0.000000	4	12	614273,3333	263	2331,47	0,00
A0A2Z5YHU8	ATPase P	5,1	79,7	762	6	NO_SP	1.000022	0.000000	0.000000	0.000000	0.000000	4	11	612526,6667	1248	490,70	0,00
B2HDS8	Isocitrate dehydrogenase [NADP]	6,2	45,6	408	0	NO_SP	1.000068	0.000000	0.000000	0.000000	0.000000	5	8	611273,3333	4525	135,09	0,01
B2HT58	PhoH-like protein PhoH2	6,4	46,9	433	0	NO_SP	1.000049	0.000001	0.000000	0.000000	0.000000	3	11	610436,6667	1187	514,38	0,00
B2HP81	Conserved hypothetical membrane protein	8,2	64,7	582	9	NO_SP	1.000009	0.000014	0.000000	0.000000	0.000000	4	6	609616,6667	5514	110,56	0,00
B2HE96	Conserved protein	4,7	19,5	182	0	NO_SP	1.000046	0.000000	0.000000	0.000000	0.000000	4	4	607760	18361	33,10	0,03
B2HIK6	Phosphopantetheine adenylyltransferase	5,0	17,1	157	0	NO_SP	1.000032	0.000000	0.000000	0.000000	0.000000	4	8	607576,6667	0	#JAKO/0!	0,00
A0A2Z5YMP5	4,5-9,10-diseco-3-hydroxy-5,9,17-trioxoandrosta-1 (10),2-diene-4-oate hydrola	7,0	32,6	295	0	NO_SP	1.000070	0.000000	0.000000	0.000000	0.000000	3	8	607030	952	637,41	0,00
B2HGX3	Glycerate kinase	4,6	38,1	381	0	NO_SP	1.000062	0.000013	0.000000	0.000000	0.000000	5	10	605066,6667	0	#JAKO/0!	0,00
A0A3E2MP32	Copper-exporting P-type ATPase A	5,7	78,3	757	7	NO_SP	1.000069	0.000001	0.000000	0.000000	0.000000	8	9	604936,6667	18982	31,87	0,03
B2HJ28	Conserved membrane protein	5,9	27,9	273	1	NO_SP	1.000009	0.000020	0.000000	0.000000	0.000000	4	8	604846,6667	0	#JAKO/0!	0,00
A0A2Z5YD02	Alpha/beta hydrolase	7,3	46,5	417	0	NO_SP	1.000051	0.000008	0.000000	0.000000	0.000000	4	5	603933,3333	4692	128,70	0,01
B2HND2	Elongation factor P	5,3	20,4	187	0	NO_SP	1.000059	0.000000	0.000000	0.000000	0.000000	4	5	603410	0	#JAKO/0!	0,00
B2HJ81	Cold shock protein A CspA_1	5,0	7,3	67	0	NO_SP	1.000056	0.000000	0.000000	0.000000	0.000000	4	3	602890	25067	24,05	0,04
A0A2Z5YDZ9	CDP-glucose 4,6-dehydratase	4,9	36,6	338	0	NO_SP	0.500450	0.498210	0.000766	0.000202	0.000172	3	11	602813,3333	6516	92,51	0,01
B2HG44	Transcriptional regulatory protein	5,4	24,1	217	0	NO_SP	1.000072	0.000000	0.000000	0.000000	0.000000	4	6	602683,3333	5180	116,34	0,01
A0A3E2MZL4	Epimerase family protein	9,8	48,9	451	0	NO_SP	1.000041	0.000000	0.000000	0.000000	0.000000	4	6	601260	1771	339,45	0,00
A0A124BU59	Prolipoprotein diacylglyceryl transferase	6,9	36,7	340	5	NO_SP	1.000021	0.000006	0.000000	0.000000	0.000000	4	3	600510	17252	34,81	0,03
A0A100I8G4	Tuberculin related peptide	4,0	15,1	145	0	NO_SP	0.842914	0.148932	0.007446	0.000209	0.000143	4	3	599466,6667	14408	41,61	0,02
A0A2Z5YKL5	Exodeoxyribonuclease 7 large subunit	10,0	44,4	415	0	NO_SP	1.000053	0.000003	0.000000	0.000000	0.000000	4	5	599253,3333	244694	2,45	0,41
A0A2Z5YD94	Uroporphyrinogen III methylase	6,2	40,1	383	0	NO_SP	0.999493	0.000556	0.000001	0.000001	0.000000	1	1	598073,3333	0	#JAKO/0!	0,00
B2HCU0	50S ribosomal protein L15	11,4	15,6	146	0	NO_SP	1.000045	0.000000	0.000000	0.000000	0.000000	3	6	598020	196653	3,04	0,33
B2HID8	Conserved protein	7,0	58,4	531	0	NO_SP	1.000071	0.000001	0.000000	0.000000	0.000000	4	7	597696,6667	4345	137,57	0,01
A0A2Z5Y806	Aminomethyltransferase	5,1	40,0	375	0	NO_SP	1.000023	0.000034	0.000000	0.000000	0.000000	4	8	597293,3333	795	750,97	0,00
A0A2Z5YMC4	M18 family aminopeptidase	6,6	42,7	400	0	NO_SP	1.000039	0.000001	0.000000	0.000000	0.000000	3	9	596570	0	#JAKO/0!	0,00
B2HMT8	Conserved hypothetical integral membrane protein YrbE1A	8,6	28,0	267	6	NO_SP	1.000005	0.000032	0.000000	0.000000	0.000000	4	4	594880	3457	172,10	0,01
B2HCX5	50S ribosomal protein L17	7,0	20,2	189	0	NO_SP	1.000053	0.000000	0.000000	0.000000	0.000000	4	3	594466,6667	80055	7,43	0,13
A0A117DX40	Antibiotic biosynthesis monooxygenase	5,1	11,0	102	0	NO_SP	1.000058	0.000001	0.000000	0.000000	0.000000	3	4	592633,3333	22201	26,69	0,04
B2HF96	Conserved protein	4,1	28,1	267	0	NO_SP	1.000052	0.000010	0.000000	0.000000	0.000000	4	5	592460	0	#JAKO/0!	0,00
B2HT73	Transcription elongation factor GreA	4,6	18,0	164	0	NO_SP	1.000038	0.000000	0.000000	0.000000	0.000000	3	7	592140	6392	92,64	0,01
A0A2Z5YCA6	2-hydroxyhepta-2,4-diene-1,7-dioate isomerase	4,9	27,9	265	0	NO_SP	1.000072	0.000002	0.000000	0.000000	0.000000	3	5	591973,3333	0	#JAKO/0!	0,00
A0A2Z5YDZ3	Uncharacterized protein	4,2	19,3	183	1	NO_SP	1.000050	0.000000	0.000000	0.000000	0.000000	3	5	591933,3333	4403	134,45	0,01
A0A2Z5YA98	Membrane protein	8,8	28,9	269	2	NO_SP	1.000034	0.000000	0.000000	0.000000	0.000000	4	7	591646,6667	0	#JAKO/0!	0,00
B2HQ90	8-amino-7-oxononanoate synthase	6,3	40,2	389	0	NO_SP	1.000053	0.000000	0.000000	0.000000	0.000000	3	9	591513,3333	4353	135,88	0,01
A0A2Z5YIG1	Uncharacterized protein	7,3	16,8	155	0	NO_SP	1.000040	0.000000	0.000000	0.000000	0.000000	1	6	590520	4380	134,83	0,01
B2HHQ2	Nucleoside-diphosphate-sugar epimerase	6,5	35,6	333	0	NO_SP	1.000034	0.000000	0.000000	0.000000	0.000000	4	1	590096,6667	1804	327,18	0,00
B2HQJ2	Endonuclease NucS	5,2	24,8	223	0	NO_SP	1.000056	0.000000	0.000000	0.000000	0.000000	3	6	589186,6667	8042	73,27	0,01
B2HGR8	UDP-N-acetylmuramoylalanine--D-glutamate ligase	4,8	50,1	488	0	NO_SP	1.000030	0.000019	0.000000	0.000000	0.000000	4	9	589143,3333	9441	62,40	0,02
A0A2Z5YLT3	Arylsulfatase	4,9	58,5	516	0	NO_SP	1.000062	0.000002	0.000000	0.000000	0.000000	4	11	589116,6667	0	#JAKO/0!	0,00
B2HMU8	Conserved Mce associated protein	7,0	20,3	185	1	NO_SP	0.999360	0.000619	0.000009	0.000002	0.000001	3	4	588496,6667	3464	169,89	0,00
A0A3E2MQY1	Long-chain-fatty-acid--CoA ligase FadD19	4,7	58,9	545	0	NO_SP	1.000039	0.000004	0.000000	0.000000	0.000000	4	7	588120	6384	92,12	0,01
A0A2Z5YB30	Uncharacterized protein	5,6	11,1	103	0	NO_SP	1.000082	0.000000	0.000000	0.000000	0.000000	3	4	588016,6667	94812	6,20	0,16
A0A2Z5YM42	Zinc ribbon domain protein	7,6	11,6	111	0	NO_SP	1.000049	0.000000	0.000000	0.000000	0.000000	2	5	587450	4966	118,31	0,01
A0A2Z5Y9Z4	Oxidoreductase	10,3	31,4	290	0	NO_SP	1.000062	0.000000	0.000000	0.000000	0.000000	4	6	586813,3333	4256	137,88	0,01
A0A3E2MQR5	zinc-ribbon_6 domain-containing protein	5,2	39,0	351	0	NO_SP	1.000032	0.000009	0.000001	0.000000	0.000000	5	9	586330	680	862,21	0,00
B2HMI5	GreA_GreB domain-containing protein	4,6	17,4	161	0	NO_SP	1.000065	0.000000	0.000000	0.000000	0.000000	3	5	585053,3333	1424	410,99	0,00

A0A2Z5YL55	Glucose-1-phosphate adenylyltransferase	4,9	**43,6**	404	0	NO_SP	1.000053	0.000007	0.000000	0.000000	0.000000	3	6	**584550**	**0**	**#JAKO/0!**	**0,00**
A0A2Z5YFK2	HTH OST-type domain-containing protein	5,7	**31,4**	288	0	NO_SP	1.000045	0.000004	0.000000	0.000000	0.000000	4	5	**582340**	**3908**	**149,03**	**0,01**
A0A100I9F0	Acyl-CoA dehydrogenase	6,5	**56,3**	519	0	NO_SP	1.000054	0.000000	0.000000	0.000000	0.000000	4	9	**581130**	**1232**	**471,85**	**0,00**
A0A100HZA5	Anti-sigma-F factor RsbW	4,8	**15,6**	145	0	NO_SP	1.000071	0.000000	0.000000	0.000000	0.000000	3	1	**579636,6667**	**13695**	**42,32**	**0,02**
A0A117E045	Bacterial regulatory s, tetR family protein	5,2	**24,3**	216	0	NO_SP	1.000066	0.000000	0.000000	0.000000	0.000000	3	6	**577026,6667**	**0**	**#JAKO/0!**	**0,00**
B2HGE6	NADH-quinone oxidoreductase subunit C	4,9	**27,0**	238	0	NO_SP	1.000047	0.000000	0.000000	0.000000	0.000000	4	9	**577003,3333**	**2694**	**214,21**	**0,00**
A0A2Z5Y8F7	Serine protease PepA	4,3	**33,4**	343	0	SP	0.000248	0.999093	0.000149	0.000188	0.000156	4	4	**576456,6667**	**3541**	**162,81**	**0,00**
B2HIJ2	Uracil-DNA glycosylase	7,8	**24,6**	227	0	NO_SP	1.000089	0.000000	0.000000	0.000000	0.000000	4	4	**576250**	**7707**	**74,77**	**0,01**
A0A100HZI2	Uncharacterized protein	4,8	**48,7**	445	0	NO_SP	1.000056	0.000000	0.000000	0.000000	0.000000	3	7	**575866,6667**	**2366**	**243,36**	**0,00**
A0A2Z5YGW1	Nicotinate-nucleotide--dimethylbenzimidazole phosphoribosyltransferase	4,5	**36,2**	358	0	NO_SP	1.000046	0.000002	0.000000	0.000000	0.000000	4	8	**574496,6667**	**1934**	**297,10**	**0,00**
B2HH04	HTH tetR-type domain-containing protein	7,3	**23,5**	213	0	NO_SP	1.000059	0.000002	0.000000	0.000000	0.000000	4	4	**573406,6667**	**4983**	**115,08**	**0,01**
A0A3E2MQ58	Methylmalonyl-CoA mutase large subunit	5,2	**80,8**	753	0	NO_SP	0.998907	0.001112	0.000018	0.000003	0.000001	4	10	**572413,3333**	**1378**	**415,45**	**0,00**
A0A2Z5YBK6	Bacterial regulatory protein, tetR family	6,2	**23,5**	216	0	NO_SP	1.000048	0.000000	0.000000	0.000000	0.000000	3	4	**570646,6667**	**0**	**#JAKO/0!**	**0,00**
A0A2Z5YNA7	Cytochrome P450	4,8	**48,7**	442	0	NO_SP	1.000034	0.000001	0.000000	0.000000	0.000000	4	7	**569746,6667**	**911**	**625,52**	**0,00**
A0A3E2MX16	3-oxoadipyl-CoA/3-oxo-5,6-dehydrosuberyl-CoA thiolase	5,1	**43,0**	400	0	NO_SP	0.999752	0.000262	0.000025	0.000001	0.000000	4	8	**569310**	**1014**	**561,25**	**0,00**
B2HD68	Glutamine synthetase GlnA3	5,0	**47,8**	453	0	NO_SP	0.997927	0.002112	0.000007	0.000003	0.000002	2	5	**568873,3333**	**13594**	**41,85**	**0,02**
A0A3E2MZD4	6-hydroxyparomomycin C oxidase	4,6	**69,0**	657	0	NO_SP	1.000044	0.000008	0.000000	0.000000	0.000000	4	9	**567726,6667**	**897**	**632,87**	**0,00**
A0A3E2MNF2	Succinyl-CoA:(R)-benzylsuccinate CoA-transferase subunit BbsF	5,3	**86,5**	814	0	NO_SP	1.000057	0.000000	0.000000	0.000000	0.000000	6	12	**567323,3333**	**8566**	**66,23**	**0,02**
A0A2Z5YBQ0	NADH-quinone oxidoreductase subunit F	6,1	**48,2**	448	0	NO_SP	1.000069	0.000001	0.000000	0.000000	0.000000	4	9	**567166,6667**	**0**	**#JAKO/0!**	**0,00**
B2HJ25	Conserved hypothetical lipoprotein LpqE	5,6	**19,4**	185	0	LIPO	0.000000	0.000000	1.000015	0.000000	0.000000	3	4	**566730**	**0**	**#JAKO/0!**	**0,00**
A0A2Z5YG91	Protein PafB	5,3	**35,5**	332	0	NO_SP	1.000037	0.000000	0.000000	0.000000	0.000000	4	7	**566436,6667**	**1505**	**376,27**	**0,00**
A0A2Z5YA43	Uncharacterized protein	10,4	**17,7**	161	0	NO_SP	1.000050	0.000001	0.000000	0.000000	0.000000	4	5	**566410**	**34963**	**16,20**	**0,06**
A0A3E2MZ61	Assimilatory sulfite reductase (ferredoxin)	5,7	**62,3**	555	0	NO_SP	1.000044	0.000005	0.000000	0.000000	0.000000	4	8	**563446,6667**	**0**	**#JAKO/0!**	**0,00**
B2HK40	Amidase AmiC_1	5,2	**50,9**	478	0	NO_SP	1.000041	0.000001	0.000000	0.000000	0.000000	4	9	**562963,3333**	**2275**	**247,44**	**0,00**
B2HCT0	Conserved transmembrane protein	5,3	**34,2**	312	4	NO_SP	1.000004	0.000031	0.000000	0.000000	0.000000	3	6	**560330**	**7257**	**77,21**	**0,01**
A0A100I552	Uncharacterized protein	5,8	**15,8**	146	0	NO_SP	1.000066	0.000001	0.000000	0.000000	0.000000	3	6	**559770**	**1678**	**333,51**	**0,00**
B2HRK6	Metal cation-transporting P-type ATPase F, CtpF	5,1	**95,0**	905	8	NO_SP	1.000026	0.000003	0.000000	0.000000	0.000000	4	8	**556910**	**2125**	**262,03**	**0,00**
B2HMF5	Glutamine-dependent NAD(+) synthetase	5,1	**74,9**	680	0	NO_SP	1.000048	0.000005	0.000001	0.000000	0.000000	4	8	**556326,6667**	**2504**	**222,15**	**0,00**
A0A3E2MQK5	Endolytic murein transglycosylase	8,3	**45,5**	418	1	NO_SP	0.999932	0.000071	0.000000	0.000000	0.000000	4	9	**555870**	**1664**	**334,02**	**0,00**
B2HHZ4	Short-chain type dehydrogenase/reductase	4,9	**30,9**	303	0	NO_SP	0.999877	0.000153	0.000000	0.000000	0.000000	1	7	**555783,3333**	**8487**	**65,48**	**0,02**
B2HQ27	Conserved hypothetical acyltransferase	6,0	**24,0**	221	0	NO_SP	1.000084	0.000000	0.000000	0.000000	0.000000	4	5	**553668**	**0**	**#JAKO/0!**	**0,00**
B2HJ55	Epoxide hydrolase EphA	4,8	**35,7**	322	0	NO_SP	1.000051	0.000001	0.000000	0.000000	0.000000	4	6	**553423,3333**	**6471**	**85,53**	**0,01**
A0A2Z5YNT9	Uncharacterized protein	4,6	**50,6**	476	0	NO_SP	1.000063	0.000000	0.000000	0.000000	0.000000	4	7	**552863,3333**	**0**	**#JAKO/0!**	**0,00**
B2HSB3	1D-myo-inositol 2-acetamido-2-deoxy-alpha-D-glucopyranoside deacetylase	4,7	**32,0**	301	0	NO_SP	0.999956	0.000078	0.000000	0.000000	0.000000	4	6	**552173,3333**	**1707**	**323,40**	**0,00**
B2HJF3	Conserved protein	7,2	**21,6**	188	0	NO_SP	1.000068	0.000000	0.000000	0.000000	0.000000	3	6	**551946,6667**	**5911**	**93,37**	**0,01**
B2HDZ1	Transcriptional regulatory protein	6,5	**20,4**	189	0	NO_SP	0.999929	0.000053	0.000007	0.000000	0.000000	3	1	**551866,6667**	**11916**	**46,31**	**0,02**
A0A2Z5YE60	Peptidoglycan endopeptidase RipA	6,9	**47,1**	446	0	SP	0.000754	0.998446	0.000197	0.000217	0.000186	4	4	**551596,6667**	**0**	**#JAKO/0!**	**0,00**
B2HLC6	Conserved protein	10,7	**14,5**	127	0	NO_SP	1.000066	0.000000	0.000000	0.000000	0.000000	3	2	**550196,6667**	**62566**	**8,79**	**0,11**
B2HEM3	Conserved protein	8,6	**9,7**	92	0	NO_SP	0.990336	0.009238	0.000310	0.000035	0.000021	3	4	**549875,4667**	**92433**	**5,95**	**0,00**
A0A2Z5YE57	Uncharacterized protein	4,4	**31,6**	307	0	NO_SP	1.000029	0.000003	0.000000	0.000000	0.000000	4	6	**549383,3333**	**0**	**#JAKO/0!**	**0,00**
A0A2Z5YIF3	Putative monooxygenase	5,0	**58,9**	551	0	NO_SP	1.000078	0.000001	0.000000	0.000000	0.000000	3	5	**549333,3333**	**7092**	**77,46**	**0,01**
A0A2Z5YD07	DhaL domain-containing protein	4,3	**56,5**	564	0	NO_SP	1.000037	0.000000	0.000000	0.000000	0.000000	3	5	**548913,3333**	**0**	**#JAKO/0!**	**0,00**
A0A2Z5YAN4	tRNA N6-adenosine threonylcarbamoyltransferase	5,8	**34,9**	343	0	NO_SP	1.000010	0.000007	0.000000	0.000000	0.000000	4	8	**548910**	**40280**	**13,63**	**0,07**
B2HD75	Short-chain type dehydrogenase/reductase	9,9	**29,8**	277	0	NO_SP	1.000051	0.000001	0.000000	0.000000	0.000000	4	6	**548636,6667**	**0**	**#JAKO/0!**	**0,00**
B2HKA9	Conserved protein	10,1	**40,7**	379	1	NO_SP	0.999959	0.000038	0.000001	0.000000	0.000000	4	5	**548490**	**0**	**#JAKO/0!**	**0,00**
A0A124BVE6	Enoyl-CoA hydratase	5,3	**24,6**	224	0	NO_SP	1.000044	0.000001	0.000000	0.000000	0.000000	4	4	**546446,6667**	**868**	**629,43**	**0,00**
A0A100IH80	4-hydroxy-3-methylbut-2-enyl diphosphate reductase	4,6	**31,8**	292	0	NO_SP	1.000038	0.000000	0.000000	0.000000	0.000000	3	5	**544383,3333**	**2125**	**256,19**	**0,00**
B2HI98	UPF0301 protein MMAR_0053	6,0	**21,8**	201	0	NO_SP	1.000061	0.000000	0.000000	0.000000	0.000000	2	4	**544070**	**0**	**#JAKO/0!**	**0,00**
A0A2Z5YFD0	Esterase	4,8	**15,5**	146	0	NO_SP	1.000087	0.000000	0.000000	0.000000	0.000000	4	4	**543966,6667**	**0**	**#JAKO/0!**	**0,00**
A0A3E2N031	Diacylglycerol O-acyltransferase	7,8	**48,6**	446	0	NO_SP	0.999785	0.000202	0.000026	0.000001	0.000000	4	7	**543360**	**12397**	**43,83**	**0,02**
B2HGR2	Purine nucleoside phosphorylase	9,9	**27,2**	262	0	NO_SP	1.000052	0.000004	0.000000	0.000000	0.000000	1	8	**543113,6667**	**4852**	**111,94**	**0,00**
A0A2Z5YJA2	Oligoribonuclease	4,3	**24,2**	222	0	NO_SP	1.000043	0.000001	0.000000	0.000000	0.000000	3	4	**542980**	**11216**	**48,41**	**0,02**
B2HDS2	Fatty acyl-AMP ligase FadD28_1 and polyketide synthase	5,7	**340,8**	3259	0	NO_SP	1.000067	0.000000	0.000000	0.000000	0.000000	6	17	**541843,3333**	**4093**	**132,39**	**0,01**
A0A3E2MX26	Carboxymethylenebutenolidase	4,7	**26,5**	246	0	NO_SP	1.000035	0.000013	0.000000	0.000000	0.000000	4	6	**541256,6667**	**5759**	**93,98**	**0,00**
B2HT00	Oxidoreductase	10,6	**36,2**	338	0	NO_SP	1.000031	0.000001	0.000000	0.000000	0.000000	4	6	**539880**	**13689**	**39,44**	**0,03**
A0A2Z5YF75	Putative cytochrome P450 143	6,6	**43,6**	391	0	NO_SP	1.000055	0.000000	0.000000	0.000000	0.000000	3	6	**539403,3333**	**0**	**#JAKO/0!**	**0,00**
A0A2Z5Y9C8	Thiazole synthase	4,4	**26,9**	263	0	NO_SP	1.000070	0.000000	0.000000	0.000000	0.000000	3	6	**538366,6667**	**735**	**732,87**	**0,00**
A0A3E2MY67	Polyphosphate kinase 2 (PPK2)	10,0	**33,7**	290	0	NO_SP	1.000044	0.000000	0.000000	0.000000	0.000000	4	6	**537633,3333**	**12738**	**42,21**	**0,02**
A0A2Z5YNC8	Lipid II isoglutaminyl synthase (glutamine-hydrolyzing) subunit GatD	6,6	**24,6**	235	0	NO_SP	1.000051	0.000000	0.000000	0.000000	0.000000	4	5	**537536,6667**	**0**	**#JAKO/0!**	**0,00**
B2HNC8	3-dehydroquinate synthase	5,1	**38,2**	362	0	NO_SP	1.000049	0.000000	0.000000	0.000000	0.000000	3	6	**536260**	**0**	**#JAKO/0!**	**0,00**
B2HCX6	tRNA pseudouridine synthase	10,0	**26,4**	237	0	NO_SP	0.998023	0.001979	0.000017	0.000003	0.000002	4	5	**536000**	**0**	**#JAKO/0!**	**0,00**
B2HNL6	DUF853 domain-containing protein	5,1	**57,1**	535	0	NO_SP	1.000084	0.000001	0.000000	0.000000	0.000000	4	9	**535506,6667**	**539**	**993,70**	**0,00**
A0A2Z5YI52	Uncharacterized protein	4,8	**103,8**	983	1	NO_SP	1.000051	0.000005	0.000000	0.000000	0.000000	2	10	**535293,3333**	**251690**	**2,13**	**0,47**
A0A2Z5YMN6	Aminotransferase	4,8	**41,8**	391	0	NO_SP	1.000043	0.000002	0.000000	0.000000	0.000000	4	9	**535046,6667**	**0**	**#JAKO/0!**	**0,00**
A0A2Z5YFE6	Membrane protein	4,5	**49,1**	460	2	NO_SP	0.855791	0.140698	0.000446	0.000472	0.000334	4	8	**534320**	**475**	**1125,49**	**0,00**
A0A2Z5Y8Q4	Uncharacterized protein	4,7	**17,9**	166	0	NO_SP	0.684757	0.313724	0.000470	0.000450	0.000253	4	3	**530697,6667**	**0**	**#JAKO/0!**	**0,00**
B2HS39	Transport transmembrane protein	4,6	**47,6**	435	0	NO_SP	1.000065	0.000000	0.000000	0.000000	0.000000	4	7	**530633,3333**	**162**	**3266,51**	**0,00**
A0A2Z5YP01	Galactofuranosyl transferase GlfT1	8,8	**34,3**	309	0	NO_SP	1.000049	0.000000	0.000000	0.000000	0.000000	4	7	**529570**	**1942**	**272,70**	**0,00**
B2HSJ8	Probable endonuclease 4	5,3	**27,0**	252	0	NO_SP	1.000072	0.000000	0.000000	0.000000	0.000000	4	7	**527656,6667**	**0**	**#JAKO/0!**	**0,00**
A0A2Z5YAR6	Thymidine phosphorylase	4,9	**45,0**	433	0	NO_SP	1.000040	0.000002	0.000000	0.000000	0.000000	4	9	**526820**	**0**	**#JAKO/0!**	**0,00**
B2HJN4	Amidase AmiC	7,9	**51,0**	472	0	NO_SP	1.000035	0.000022	0.000002	0.000000	0.000000	4	7	**526810**	**1900**	**277,28**	**0,00**
A0A3E2MR18	ATP synthase I chain	10,4	**16,3**	156	4	NO_SP	1.000015	0.000001	0.000000	0.000000	0.000000	3	1	**523843,3333**	**7781**	**67,33**	**0,01**
A0A2Z5YCT4	NUDIX domain-containing protein	4,3	**28,8**	262	0	NO_SP	1.000028	0.000004	0.000000	0.000000	0.000000	4	5	**522733,3333**	**0**	**#JAKO/0!**	**0,00**
B2HT68	SnoaL-like domain-containing protein	4,6	**13,7**	127	0	NO_SP	1.000041	0.000000	0.000000	0.000000	0.000000	4	4	**522540**	**5593**	**93,42**	**0,01**
A0A2Z5Y8N3	Aldehyde dehydrogenase	4,5	**51,7**	488	0	NO_SP	1.000069	0.000000	0.000000	0.000000	0.000000	5	6	**521510**	**0**	**#JAKO/0!**	**0,00**
A0A2Z5YDT3	Uncharacterized protein	4,1	**35,4**	328	0	NO_SP	0.997386	0.002617	0.000013	0.000006	0.000002	1	1	**520916,6667**	**1674**	**311,11**	**0,00**
A0A2Z5YE89	Thiol reductant ABC exporter subunit CydC	7,6	**58,8**	564	5	NO_SP	1.000054	0.000004	0.000000	0.000000	0.000000	4	8	**520313,3333**	**2187**	**237,86**	**0,00**

B2HL11	Non-specific serine/threonine protein kinase	6,7	**64,2**	601	1	NO_SP	1.000036	0.000000	0.000000	0.000000	0.000000	5	6	**519530**	**1961**	**264,89**	**0,00**
A0A2Z5YD27	Biotin carboxyl carrier protein	5,4	**113,4**	1077	0	NO_SP	1.000034	0.000005	0.000000	0.000000	0.000000	4	12	**518706,6667**	**135950**	**3,82**	**0,26**
A0A2Z5Y9R9	Thiol-disulfide oxidoreductase ResA	6,2	**21,8**	203	0	LIPO	0.000000	0.000000	1.000047	0.000000	0.000000	3	5	**518596,6667**	**3882**	**133,58**	**0,01**
A0A2Z5YNN4	Uncharacterized protein	4,6	**26,8**	257	0	NO_SP	0.999875	0.000174	0.000001	0.000000	0.000000	4	4	**518320**	**1882**	**275,37**	**0,00**
A0A2Z5YHF4	Haloalkane dehalogenase	8,4	**32,8**	297	0	NO_SP	1.000046	0.000000	0.000000	0.000000	0.000000	4	6	**517903,3333**	**3919**	**132,15**	**0,01**
A0A3E2MNS3	ABC transporter ATP-binding/permease protein	8,1	**94,1**	884	6	NO_SP	1.000039	0.000007	0.000000	0.000000	0.000000	1	1	**516870**	**0**	**#JAKO/0!**	**0,00**
A0A2Z5Y8L1	Putative transport protein MmpL11	6,9	**105,1**	986	10	NO_SP	0.948589	0.050431	0.000761	0.000084	0.000047	4	9	**516493,3333**	**861**	**599,92**	**0,00**
B2HKI8	Transcriptional regulatory protein	5,4	**21,9**	201	0	NO_SP	1.000055	0.000001	0.000000	0.000000	0.000000	4	5	**516183,3333**	**671704**	**0,77**	**1,30**
A0A2Z5YD08	Signal peptidase I	5,2	**30,8**	287	1	NO_SP	0.998810	0.001194	0.000002	0.000001	0.000000	3	3	**515830**	**10807**	**47,73**	**0,02**
B2HS47	Putative O-methyltransferase MMAR_4217	4,5	**23,0**	224	0	NO_SP	0.997089	0.002852	0.000020	0.000029	0.000007	3	5	**515816,6667**	**742**	**694,80**	**0,00**
A0A2Z5YE85	Epimerase domain-containing protein	5,3	**33,4**	310	0	NO_SP	1.000051	0.000004	0.000000	0.000000	0.000000	4	7	**515170**	**0**	**#JAKO/0!**	**0,00**
B2HF29	Conserved membrane protein	4,8	**34,4**	321	0	NO_SP	1.000045	0.000000	0.000000	0.000000	0.000000	3	6	**513623,3333**	**0**	**#JAKO/0!**	**0,00**
A0A2Z5YHK5	Oxidoreductase	6,0	**30,1**	285	0	NO_SP	0.999991	0.000034	0.000000	0.000000	0.000000	4	6	**513396,6667**	**3060**	**167,76**	**0,01**
A0A2Z5YGI9	Uncharacterized protein	5,0	**34,2**	338	0	LIPO	0.000000	0.000000	1.000068	0.000000	0.000000	4	6	**513346,6667**	**0**	**#JAKO/0!**	**0,00**
B2HQN2	Exodeoxyribonuclease III	4,9	**30,3**	276	0	NO_SP	1.000043	0.000000	0.000000	0.000000	0.000000	4	4	**512786,6667**	**6926**	**74,04**	**0,01**
A0A2Z5YDT7	ATPase AAA	6,7	**47,4**	452	0	NO_SP	0.999825	0.000183	0.000014	0.000001	0.000000	3	9	**511526,6667**	**0**	**#JAKO/0!**	**0,00**
A0A2Z5YHC1	Peptidase M23	5,6	**47,1**	444	0	LIPO	0.000035	0.001549	0.998448	0.000008	0.000002	4	7	**511023,3333**	**0**	**#JAKO/0!**	**0,00**
A0A2Z5YAM8	Alanine racemase	6,2	**41,0**	388	0	NO_SP	0.999576	0.000434	0.000004	0.000000	0.000000	4	7	**511020**	**379**	**1347,98**	**0,00**
B2HMB4	Sulfate-binding lipoprotein SubI	5,1	**37,3**	349	0	LIPO	0.000080	0.005532	0.994404	0.000006	0.000006	4	6	**510833,3333**	**0**	**#JAKO/0!**	**0,00**
B2HN47	Acyl-CoA thioesterase II TesB2	5,7	**31,2**	281	0	NO_SP	1.000090	0.000000	0.000000	0.000000	0.000000	4	7	**509776,6667**	**0**	**#JAKO/0!**	**0,00**
B2HEB6	Acyl-CoA dehydrogenase FadE12	5,4	**41,7**	388	0	NO_SP	1.000060	0.000000	0.000000	0.000000	0.000000	5	7	**509223,3333**	**0**	**#JAKO/0!**	**0,00**
B2HE09	Short-chain type dehydrogenase/reductase	5,0	**26,8**	255	0	NO_SP	1.000044	0.000005	0.000000	0.000000	0.000000	4	7	**509086,6667**	**0**	**#JAKO/0!**	**0,00**
A0A2Z5YHV0	Uncharacterized protein	7,0	**59,8**	526	0	NO_SP	1.000049	0.000000	0.000000	0.000000	0.000000	1	7	**508790**	**1914**	**265,81**	**0,00**
B2HJ47	Conserved hypothetical alanine and leucine-rich protein	5,8	**31,6**	308	0	NO_SP	1.000069	0.000000	0.000000	0.000000	0.000000	3	4	**508180**	**3716**	**136,74**	**0,00**
A0A3E2MMV6	Uncharacterized protein	4,5	**16,4**	149	0	NO_SP	1.000069	0.000000	0.000000	0.000000	0.000000	4	5	**508146,6667**	**6434**	**78,98**	**0,01**
A0A2Z5YKQ4	Wzz domain-containing protein	10,5	**48,4**	461	1	NO_SP	0.608720	0.343943	0.040331	0.002309	0.001122	4	6	**505516,6667**	**8261**	**61,20**	**0,00**
A0A2Z5YK69	Uncharacterized protein	4,9	**22,0**	201	0	NO_SP	1.000055	0.000001	0.000000	0.000000	0.000000	3	7	**501360**	**0**	**#JAKO/0!**	**0,00**
A0A2Z5YBL0	Peptidyl-prolyl cis-trans isomerase	4,9	**13,1**	124	0	NO_SP	1.000065	0.000001	0.000000	0.000000	0.000000	3	4	**500703,3333**	**0**	**#JAKO/0!**	**0,00**
A0A100I0J3	Arabinofuranosyltransferase AftA	10,0	**69,3**	644	13	NO_SP	1.000037	0.000000	0.000000	0.000000	0.000000	4	4	**500103,3333**	**5828**	**85,81**	**0,00**
A0A2Z5YN61	Fido domain-containing protein	6,3	**26,2**	256	0	NO_SP	1.000034	0.000005	0.000000	0.000000	0.000000	4	6	**499986,6667**	**0**	**#JAKO/0!**	**0,00**
B2HM72	Oxidoreductase	5,0	**30,6**	286	0	NO_SP	1.000059	0.000000	0.000000	0.000000	0.000000	4	3	**499030**	**0**	**#JAKO/0!**	**0,00**
B2HT95	Acid phosphatase	4,5	**33,1**	298	0	NO_SP	1.000080	0.000000	0.000000	0.000000	0.000000	4	4	**498133,3333**	**2760**	**180,51**	**0,01**
A0A100I428	Lipoprotein	4,0	**16,1**	154	0	NO_SP	1.000044	0.000000	0.000000	0.000000	0.000000	4	2	**497380**	**25372**	**19,60**	**0,05**
A0A3E2MNC9	Acylamidase	5,4	**48,7**	468	0	NO_SP	1.000045	0.000001	0.000000	0.000000	0.000000	4	7	**497366,6667**	**0**	**#JAKO/0!**	**0,00**
B2HD42	Alanine and proline-rich secreted protein Apa	4,5	**33,9**	334	1	SP	0.020603	0.857315	0.007576	0.109280	0.004734	4	4	**497323,3333**	**3744**	**132,84**	**0,00**
A0A124BX64	Heat shock protein HtpX	5,6	**32,9**	301	1	NO_SP	1.000078	0.000002	0.000000	0.000000	0.000000	3	5	**496103,3333**	**2176**	**227,94**	**0,00**
B2HHS3	DAGKc domain-containing protein	4,9	**34,2**	320	0	NO_SP	1.000032	0.000001	0.000000	0.000000	0.000000	4	4	**496103,3333**	**0**	**#JAKO/0!**	**0,00**
A0A2Z5YNZ1	Uncharacterized protein	6,9	**33,5**	304	0	NO_SP	1.000058	0.000000	0.000000	0.000000	0.000000	1	7	**495853,3333**	**1824**	**271,85**	**0,00**
B2HPT3	Orotate phosphoribosyltransferase	6,2	**18,8**	179	0	NO_SP	0.999913	0.000120	0.000006	0.000000	0.000000	3	2	**494606,6667**	**1647**	**300,24**	**0,00**
A0A2Z5YHN5	Putative HTH-type transcriptional regulator	10,9	**32,8**	312	0	NO_SP	0.995897	0.004087	0.000015	0.000007	0.000004	4	9	**493920**	**244**	**2020,54**	**0,00**
B2HJX4	Coenzyme F420-reducing hydrogenase, alpha subunit	5,0	**47,2**	430	0	NO_SP	1.000085	0.000000	0.000000	0.000000	0.000000	4	7	**492780**	**464**	**1062,22**	**0,00**
A0A124BV82	Membrane protein	4,4	**18,7**	173	0	NO_SP	1.000065	0.000001	0.000000	0.000000	0.000000	4	3	**491933,3333**	**0**	**#JAKO/0!**	**0,00**
A0A100IFB9	Deoxyguanosinetriphosphate triphosphohydrolase-like protein	6,5	**46,1**	425	0	NO_SP	1.000042	0.000000	0.000000	0.000000	0.000000	3	9	**491013,3333**	**224**	**2192,02**	**0,00**
A0A117DXB8	Acyltransferase	6,3	**34,7**	318	0	NO_SP	1.000063	0.000000	0.000000	0.000000	0.000000	4	5	**490756,6667**	**3034**	**161,77**	**0,01**
A0A2Z5YL82	Uncharacterized protein	5,6	**69,7**	663	2	SP	0.000420	0.998801	0.000199	0.000219	0.000185	3	5	**490080**	**0**	**#JAKO/0!**	**0,00**
A0A2Z5YBE9	Putative NAD(P)H nitroreductase	6,7	**35,9**	332	0	NO_SP	1.000070	0.000000	0.000000	0.000000	0.000000	3	8	**490033,3333**	**0**	**#JAKO/0!**	**0,00**
B2HP20	dCTP deaminase, dUMP-forming	6,3	**20,7**	190	0	NO_SP	1.000077	0.000000	0.000000	0.000000	0.000000	3	3	**485813,3333**	**0**	**#JAKO/0!**	**0,00**
A0A2Z5YGY0	Uncharacterized protein	4,7	**159,4**	1546	0	NO_SP	1.000035	0.000006	0.000000	0.000000	0.000000	1	4	**481722,6667**	**0**	**#JAKO/0!**	**0,00**
B2HH20	UPF0678 fatty acid-binding protein-like protein MMAR_4871	4,4	**24,0**	223	0	NO_SP	0.998534	0.001416	0.000033	0.000006	0.000004	3	6	**481386,6667**	**0**	**#JAKO/0!**	**0,00**
B2HH76	Dehydrogenase/reductase	5,7	**26,6**	250	0	NO_SP	1.000072	0.000001	0.000000	0.000000	0.000000	4	4	**481083,3333**	**3726**	**129,12**	**0,01**
A0A3E2MVZ0	Uncharacterized protein	4,1	**30,3**	274	1	NO_SP	1.000065	0.000000	0.000000	0.000000	0.000000	3	4	**479506,6667**	**1947**	**246,23**	**0,00**
A0A117DTP4	Adenylate cyclase	6,9	**53,5**	500	6	NO_SP	1.000039	0.000004	0.000000	0.000000	0.000000	4	4	**478806,6667**	**8156**	**58,70**	**0,02**
A0A3E2MRU5	Prephenate dehydratase	4,5	**34,5**	332	0	NO_SP	1.000058	0.000004	0.000000	0.000000	0.000000	4	4	**477990**	**0**	**#JAKO/0!**	**0,00**
A0A2Z5YPL6	TIGR03086 family protein	4,7	**20,6**	194	0	NO_SP	1.000075	0.000003	0.000000	0.000000	0.000000	3	2	**476820**	**221**	**2154,73**	**0,00**
A0A2Z5YGK8	Mycobactin synthetase protein B	4,6	**107,1**	987	0	NO_SP	1.000069	0.000000	0.000000	0.000000	0.000000	1	3	**476536,6667**	**0**	**#JAKO/0!**	**0,00**
B2HT37	Conserved hypothetical alanine and proline rich protein	5,4	**69,5**	676	0	NO_SP	1.000043	0.000003	0.000000	0.000000	0.000000	2	2	**476456,6667**	**0**	**#JAKO/0!**	**0,00**
A0A2Z5YPY8	R3H domain-containing protein	4,2	**19,8**	182	0	NO_SP	0.999983	0.000048	0.000000	0.000000	0.000000	4	6	**476420**	**40480**	**11,77**	**0,08**
A0A2Z5YJS0	Acyl-CoA dehydrogenase	6,0	**43,8**	397	0	NO_SP	1.000057	0.000000	0.000000	0.000000	0.000000	3	7	**475926,6667**	**680**	**700,17**	**0,00**
A0A2Z5YNR1	Enoyl-CoA hydratase	5,1	**26,0**	248	0	NO_SP	1.000063	0.000001	0.000000	0.000000	0.000000	3	5	**475263,3333**	**6654**	**71,43**	**0,01**
A0A117DY36	Uncharacterized protein	7,7	**17,4**	168	0	LIPO	0.000117	0.000112	0.999813	0.000000	0.000000	3	3	**475147,6667**	**0**	**#JAKO/0!**	**0,00**
B2HKU8	Transcriptional repressor SirR	4,9	**25,2**	230	0	NO_SP	1.000064	0.000000	0.000000	0.000000	0.000000	4	5	**473920**	**0**	**#JAKO/0!**	**0,00**
B2HHA7	Lysine N-acyltransferase MbtK	4,7	**23,5**	207	0	NO_SP	1.000048	0.000003	0.000000	0.000000	0.000000	4	6	**472793,3333**	**8225**	**57,48**	**0,02**
A0A100IB01	GCN5-related N-acetyltransferase	5,4	**29,1**	270	0	NO_SP	1.000061	0.000000	0.000000	0.000000	0.000000	4	3	**471586,6667**	**10813**	**43,61**	**0,00**
A0A2Z5YIC5	Gamma-glutamyl phosphate reductase	5,4	**44,5**	423	0	NO_SP	1.000058	0.000004	0.000000	0.000000	0.000000	3	5	**469463,3333**	**2286**	**205,37**	**0,00**
A0A2Z5Y8M6	AMP-dependent ligase	4,8	**52,7**	495	0	NO_SP	0.996284	0.003642	0.000044	0.000026	0.000008	4	8	**468840**	**0**	**#JAKO/0!**	**0,00**
A0A2Z5YJY5	AAA_15 domain-containing protein	5,2	**93,5**	876	0	NO_SP	1.000056	0.000000	0.000000	0.000000	0.000000	3	14	**468296,6667**	**0**	**#JAKO/0!**	**0,00**
A0A2Z5YDZ6	Cell division protein ZapE	6,2	**37,4**	346	0	NO_SP	1.000067	0.000000	0.000000	0.000000	0.000000	4	7	**466856,6667**	**0**	**#JAKO/0!**	**0,00**
B2HEN8	Mannose-6-phosphate isomerase	4,4	**43,8**	408	0	NO_SP	1.000047	0.000018	0.000000	0.000000	0.000000	4	8	**466743,3333**	**0**	**#JAKO/0!**	**0,00**
B2HFU9	ATP-dependent DNA helicase HelY	7,4	**100,6**	918	0	NO_SP	1.000047	0.000000	0.000000	0.000000	0.000000	4	9	**466466,6667**	**3266**	**142,83**	**0,01**
B2HMM2	Conserved hypothetical membrane protein	4,4	**48,5**	463	1	NO_SP	0.999941	0.000075	0.000000	0.000000	0.000000	4	5	**465240**	**0**	**#JAKO/0!**	**0,00**
B2HJU5	Acyltransferase ElaA	6,1	**17,7**	156	0	NO_SP	1.000033	0.000000	0.000000	0.000000	0.000000	3	3	**464226,6667**	**0**	**#JAKO/0!**	**0,00**
A0A3E2MYB3	Molybdopterin biosynthesis protein MoeB	4,7	**13,8**	127	0	NO_SP	1.000020	0.000000	0.000000	0.000000	0.000000	4	4	**464173,3333**	**10023**	**46,31**	**0,02**
A0A2Z5YB47	PPOX class F420-dependent oxidoreductase	5,1	**14,9**	136	0	NO_SP	1.000046	0.000000	0.000000	0.000000	0.000000	3	3	**464060**	**15985**	**29,03**	**0,03**
A0A3E2MMP0	Putative amino acid permease YhdG	9,8	**52,2**	494	12	NO_SP	0.999979	0.000035	0.000000	0.000000	0.000000	4	3	**464000**	**12248**	**37,88**	**0,03**
A0A2Z5Y7U6	Membrane protein	4,8	**15,6**	143	0	NO_SP	1.000055	0.000000	0.000000	0.000000	0.000000	3	2	**463690**	**15591**	**29,74**	**0,03**
B2HIC1	Replicative DNA helicase	5,4	**50,7**	460	0	NO_SP	1.000053	0.000000	0.000000	0.000000	0.000000	3	5	**463686,6667**	**73393**	**6,32**	**0,00**
A0A2Z5YF12	DUF4185 domain-containing protein	7,0	**36,7**	334	0	NO_SP	1.000066	0.000001	0.000000	0.000000	0.000000	4	5	**463193,3333**	**1089**	**425,18**	**0,00**

A0A124BWW5	Membrane protein	9,7	**19,1**	178	2	NO_SP	1.000044	0.000001	0.000000	0.000000	0.000000	3	3	**463180**	**9169**	**50,51**	**0,02**
A0A2Z5YPK7	tRNA adenosine deaminase	3,8	**18,4**	173	0	NO_SP	1.000041	0.000001	0.000000	0.000000	0.000000	4	3	**462790**	**21131**	**21,90**	**0,05**
B2HGT1	Geranylgeranyl pyrophosphate synthetase IdsA2	5,7	**36,4**	340	0	NO_SP	1.000043	0.000002	0.000000	0.000000	0.000000	3	5	**462293,3333**	**7385**	**62,60**	**0,02**
B2HR61	Conserved protein	6,7	**29,5**	277	0	NO_SP	0.999997	0.000052	0.000000	0.000000	0.000000	3	7	**461840**	**0**	**#JAKO/0!**	**0,00**
A0A2Z5YBX4	Carbon starvation protein A	8,5	**80,5**	758	15	NO_SP	0.999958	0.000050	0.000000	0.000000	0.000000	3	7	**461143,3333**	**13141**	**35,09**	**0,03**
B2HGR0	Cell division protein SepF	5,3	**25,7**	223	0	NO_SP	1.000033	0.000000	0.000000	0.000000	0.000000	3	3	**461080**	**1434**	**321,51**	**0,00**
B2HN43	Acyltransferase	8,5	**35,0**	316	0	NO_SP	1.000046	0.000013	0.000000	0.000000	0.000000	4	4	**460276,6667**	**10970**	**41,96**	**0,02**
A0A3E2MZV9	Chorismate pyruvate-lyase	6,9	**22,7**	202	0	NO_SP	1.000053	0.000000	0.000000	0.000000	0.000000	3	5	**460200**	**483**	**952,47**	**0,00**
B2HSP2	Uncharacterized protein	4,0	**14,3**	138	0	NO_SP	0.998075	0.001943	0.000013	0.000003	0.000001	3	5	**459000**	**3176**	**144,52**	**0,01**
B2HMJ6	Acyl-CoA dehydrogenase FadE35	6,3	**65,2**	598	0	NO_SP	1.000051	0.000000	0.000000	0.000000	0.000000	4	9	**457780**	**6627**	**69,08**	**0,01**
B2HCV3	Putative S-adenosyl-L-methionine-dependent methyltransferase MMAR_1069	4,3	**34,3**	316	0	NO_SP	0.885787	0.111745	0.001757	0.000246	0.000155	4	5	**455736,6667**	**0**	**#JAKO/0!**	**0,00**
B2HEQ9	Conserved protein	4,5	**17,3**	160	0	NO_SP	1.000049	0.000000	0.000000	0.000000	0.000000	3	2	**455623,3333**	**10319**	**44,16**	**0,02**
B2HKU9	Riboflavin biosynthesis protein	6,4	**35,1**	324	0	NO_SP	1.000030	0.000000	0.000000	0.000000	0.000000	4	5	**455293,3333**	**3979**	**114,43**	**0,01**
A0A2Z5YGE9	UDP-N-acetylmuramoyl-tripeptide--D-alanyl-D-alanine ligase	4,9	**53,1**	525	0	NO_SP	0.999993	0.000055	0.000005	0.000000	0.000000	4	7	**454726,6667**	**0**	**#JAKO/0!**	**0,00**
A0A2Z5YEP0	TatD family hydrolase	4,9	**31,6**	283	0	NO_SP	1.000073	0.000000	0.000000	0.000000	0.000000	4	8	**454720**	**2897**	**156,95**	**0,01**
B2HDC7	Monooxygenase	6,1	**42,1**	387	0	NO_SP	1.000046	0.000000	0.000000	0.000000	0.000000	4	6	**453823,3333**	**12023**	**37,74**	**0,03**
A0A3E2MP02	Putative sulfoacetate--CoA ligase	6,7	**55,8**	528	0	NO_SP	1.000042	0.000000	0.000000	0.000000	0.000000	4	8	**453473,3333**	**0**	**#JAKO/0!**	**0,00**
B2HEU4	Isochorismate synthase	5,0	**38,2**	366	0	NO_SP	1.000036	0.000013	0.000001	0.000000	0.000000	4	7	**453126,6667**	**939**	**482,67**	**0,00**
B2HPV0	Membrane-anchored adenylyl cyclase Cya_1	5,2	**47,6**	441	6	NO_SP	0.999822	0.000195	0.000000	0.000000	0.000000	4	7	**452396,6667**	**0**	**#JAKO/0!**	**0,00**
B2HDK0	Ribosomal RNA small subunit methyltransferase A	6,1	**29,6**	274	0	NO_SP	1.000023	0.000023	0.000001	0.000000	0.000000	3	6	**452380**	**5310**	**85,20**	**0,01**
A0A117DYY4	Serine O-acetyltransferase	7,2	**24,3**	228	0	NO_SP	1.000057	0.000001	0.000000	0.000000	0.000000	3	3	**452160**	**16845**	**26,84**	**0,00**
A0A2Z5YDV0	Putative enoyl-CoA hydratase echA12	6,1	**28,7**	269	0	NO_SP	1.000059	0.000000	0.000000	0.000000	0.000000	3	6	**451936,6667**	**0**	**#JAKO/0!**	**0,00**
A0A2Z5YG60	Beta-lactamase	4,7	**29,2**	271	0	NO_SP	1.000041	0.000001	0.000000	0.000000	0.000000	5	6	**451453,3333**	**0**	**#JAKO/0!**	**0,00**
B2HDI9	Transcriptional regulatory protein (Probably TetR-family)	5,7	**23,0**	209	0	NO_SP	1.000053	0.000000	0.000000	0.000000	0.000000	3	4	**451306,6667**	**1915**	**235,63**	**0,00**
A0A2Z5YIZ0	S-adenosyl-L-methionine-dependent methyltransferase	4,3	**33,8**	310	0	NO_SP	0.982920	0.016792	0.000181	0.000047	0.000027	4	5	**451016,6667**	**0**	**#JAKO/0!**	**0,00**
A0A2Z5YBD7	Bac_luciferase domain-containing protein	6,2	**33,2**	310	0	NO_SP	0.997067	0.002953	0.000011	0.000011	0.000004	4	7	**450860**	**57559**	**7,83**	**0,13**
A0A2Z5Y9T1	Porphobilinogen deaminase	4,6	**32,9**	317	0	NO_SP	1.000058	0.000000	0.000000	0.000000	0.000000	4	7	**449140**	**0**	**#JAKO/0!**	**0,00**
B2HPS8	Flavohemoglobin	7,4	**44,0**	393	0	NO_SP	1.000100	0.000000	0.000000	0.000000	0.000000	4	8	**448770**	**0**	**#JAKO/0!**	**0,00**
A0A2Z5YL87	Enoyl-CoA hydratase	4,8	**26,5**	254	0	NO_SP	1.000051	0.000002	0.000000	0.000000	0.000000	3	6	**448533,3333**	**0**	**#JAKO/0!**	**0,00**
A0A2Z5YKW4	Putative UbiE/COQ5 methyltransferase	6,6	**26,8**	248	0	NO_SP	1.000053	0.000002	0.000000	0.000000	0.000000	4	6	**447976,6667**	**0**	**#JAKO/0!**	**0,00**
A0A2Z5YAY8	Bifunctional F420 biosynthesis protein FbiB	5,3	**48,4**	454	0	NO_SP	1.000043	0.000019	0.000000	0.000000	0.000000	3	8	**447763,3333**	**0**	**#JAKO/0!**	**0,00**
B2HMF1	Adenylate or guanylate cyclase	5,1	**80,2**	730	2	NO_SP	0.999881	0.000121	0.000000	0.000000	0.000000	4	10	**447730**	**0**	**#JAKO/0!**	**0,00**
B2HMM4	Phosphoglycerate mutase	4,6	**24,3**	228	0	NO_SP	1.000057	0.000001	0.000000	0.000000	0.000000	4	5	**446743,3333**	**597**	**748,48**	**0,00**
B2HJU6	Magnesium chelatase	7,4	**65,6**	616	0	NO_SP	0.999956	0.000076	0.000002	0.000000	0.000000	4	7	**445143,3333**	**0**	**#JAKO/0!**	**0,00**
A0A2Z5Y7M8	D-Ala-D-Ala carboxypeptidase	4,4	**45,9**	435	0	LIPO	0.006743	0.322155	0.670649	0.000097	0.000347	3	4	**444996,6667**	**14136**	**31,48**	**0,03**
A0A2Z5YM06	Putative cytochrome P450 126	4,9	**46,1**	418	0	NO_SP	1.000041	0.000000	0.000000	0.000000	0.000000	4	9	**444560**	**32288**	**13,77**	**0,07**
B2HDW2	Peptidase M20 domain-containing protein 2	4,7	**40,6**	393	0	NO_SP	1.000073	0.000002	0.000000	0.000000	0.000000	4	5	**443043,3333**	**68360**	**6,48**	**0,15**
A0A2Z5YIZ1	Phospholipase C	6,4	**56,6**	521	0	TAT	0.000000	0.000000	0.000000	0.999986	0.000009	4	4	**442903,3333**	**1987**	**222,95**	**0,00**
A0A2Z5Y7U2	Beta-ketoadipyl-CoA thiolase	6,3	**42,1**	404	0	NO_SP	1.000021	0.000001	0.000000	0.000000	0.000000	4	8	**442660**	**0**	**#JAKO/0!**	**0,00**
A0A2Z5YMI6	Type III restriction endonuclease subunit R	4,7	**100,6**	907	0	NO_SP	1.000108	0.000000	0.000000	0.000000	0.000000	1	8	**442076,6667**	**161426**	**2,74**	**0,37**
B2HGF2	Conserved protein	6,5	**19,3**	179	0	NO_SP	1.000055	0.000000	0.000000	0.000000	0.000000	4	3	**441960**	**0**	**#JAKO/0!**	**0,00**
A0A3E2MPX4	Carboxylate-amine ligase YbdK	6,1	**54,5**	491	0	NO_SP	1.000051	0.000001	0.000000	0.000000	0.000000	4	6	**441953,3333**	**55891**	**7,91**	**0,13**
A0A3E2MSS2	Peptidoglycan-binding protein ArfA	4,5	**34,1**	332	1	NO_SP	0.999983	0.000062	0.000000	0.000000	0.000000	4	5	**440363,3333**	**0**	**#JAKO/0!**	**0,00**
A0A2Z5YD98	ANTAR domain protein	4,4	**27,7**	261	0	NO_SP	1.000036	0.000004	0.000000	0.000000	0.000000	3	6	**440226,6667**	**624327**	**0,71**	**1,42**
A0A100I7Y0	Uncharacterized protein	7,5	**30,4**	277	0	NO_SP	1.000067	0.000000	0.000000	0.000000	0.000000	3	6	**439606,6667**	**0**	**#JAKO/0!**	**0,00**
B2HHN0	Uncharacterized protein	5,1	**29,9**	269	0	NO_SP	1.000037	0.000000	0.000000	0.000000	0.000000	5	6	**439416,6667**	**519**	**846,66**	**0,00**
B2HSX1	Conserved protein	4,8	**26,4**	253	1	NO_SP	0.999935	0.000096	0.000000	0.000000	0.000000	4	6	**439400**	**1577**	**278,68**	**0,00**
A0A2Z5Y7F5	FHA domain-containing protein FhaA	4,3	**58,4**	546	0	NO_SP	1.000059	0.000000	0.000000	0.000000	0.000000	4	6	**437566,6667**	**0**	**#JAKO/0!**	**0,00**
A0A3E2MZH9	Protein NrdI	6,9	**16,6**	152	0	NO_SP	1.000064	0.000000	0.000000	0.000000	0.000000	4	5	**436523,3333**	**0**	**#JAKO/0!**	**0,00**
B2HR71	Tyrosine recombinase XerD	10,1	**33,5**	313	0	NO_SP	1.000055	0.000000	0.000000	0.000000	0.000000	4	10	**435746,6667**	**1360**	**320,34**	**0,00**
A0A2Z5YBV9	ATP-dependent zinc metalloprotease FtsH	5,5	**44,2**	411	0	NO_SP	1.000078	0.000001	0.000000	0.000000	0.000000	3	7	**435683,3333**	**12603**	**34,57**	**0,00**
A0A124BW96	Mce associated membrane protein	4,1	**25,2**	233	1	NO_SP	1.000065	0.000000	0.000000	0.000000	0.000000	4	3	**435553,3333**	**3995**	**109,02**	**0,01**
A0A2Z5Y9I5	DinB superfamily protein	4,8	**18,6**	171	0	NO_SP	1.000059	0.000000	0.000000	0.000000	0.000000	3	5	**433310**	**433**	**1000,18**	**0,00**
A0A3E2MQP2	Putative D,D-dipeptide-binding periplasmic protein DdpA	5,7	**59,6**	570	0	LIPO	0.025651	0.464006	0.506833	0.000476	0.002655	4	9	**432340**	**0**	**#JAKO/0!**	**0,00**
B2HD91	Conserved secreted protein	4,1	**7,4**	72	0	NO_SP	1.000072	0.000003	0.000000	0.000000	0.000000	3	4	**432116,6667**	**108936**	**3,97**	**0,25**
B2HGF8	Conserved protein	4,4	**30,1**	298	0	NO_SP	1.000060	0.000000	0.000000	0.000000	0.000000	3	5	**432090**	**3552**	**121,65**	**0,00**
A0A2Z5YAN3	Enoyl-CoA hydratase	6,0	**26,7**	257	0	NO_SP	1.000050	0.000001	0.000000	0.000000	0.000000	4	5	**432010**	**9805**	**44,06**	**0,02**
A0A2Z5YB88	Putative aminoglycoside phosphotransferase	4,8	**39,1**	345	0	NO_SP	1.000031	0.000005	0.000000	0.000000	0.000000	4	7	**431890**	**0**	**#JAKO/0!**	**0,00**
B2HLR2	Conserved protein	4,9	**7,3**	70	0	NO_SP	1.000055	0.000001	0.000000	0.000000	0.000000	3	3	**431583,3333**	**2953**	**146,16**	**0,00**
A0A2Z5YGB3	Methyltranfer_dom domain-containing protein	5,2	**45,5**	408	0	NO_SP	1.000046	0.000000	0.000000	0.000000	0.000000	4	8	**430690**	**1203**	**357,93**	**0,00**
A0A2Z5YD88	Inositol-1-monophosphatase	4,5	**29,8**	289	0	NO_SP	1.000065	0.000001	0.000000	0.000000	0.000000	4	6	**429796,6667**	**0**	**#JAKO/0!**	**0,00**
B2HP82	Dehydrogenase	7,8	**50,2**	473	0	NO_SP	1.000042	0.000006	0.000000	0.000000	0.000000	3	6	**429725,3333**	**0**	**#JAKO/0!**	**0,00**
A0A2Z5YM80	Long-chain-fatty-acid--CoA/3-oxocholest-4-en-26-o ate--CoA ligase	5,0	**59,8**	548	0	NO_SP	1.000037	0.000003	0.000000	0.000000	0.000000	4	8	**429600**	**2340**	**183,58**	**0,00**
B2HGJ3	Conserved transmembrane protein	6,0	**35,3**	328	3	NO_SP	1.000038	0.000014	0.000000	0.000000	0.000000	3	4	**429420**	**3964**	**108,33**	**0,00**
A0A2Z5YI17	Hydroxylase	4,6	**27,7**	261	0	NO_SP	1.000042	0.000005	0.000000	0.000000	0.000000	1	6	**429356,6667**	**0**	**#JAKO/0!**	**0,00**
B2HE56	Nucleoside-diphosphate-sugar epimerase WcaG-like protein	5,3	**32,3**	300	0	NO_SP	1.000056	0.000000	0.000000	0.000000	0.000000	4	4	**427966,6667**	**2722**	**157,22**	**0,00**
B2HSZ9	Conserved hypothetical membrane protein	9,8	**19,1**	169	3	NO_SP	0.999996	0.000000	0.000000	0.000000	0.000000	3	2	**427643,3333**	**25338**	**16,88**	**0,06**
A0A2Z5YBK1	DNA helicase	6,6	**76,9**	709	0	NO_SP	1.000047	0.000000	0.000000	0.000000	0.000000	3	9	**426563,3333**	**0**	**#JAKO/0!**	**0,00**
B2HGR1	Pyridoxal phosphate homeostasis protein	8,8	**28,2**	261	0	NO_SP	1.000055	0.000001	0.000000	0.000000	0.000000	4	6	**426420**	**2213**	**192,73**	**0,01**
A0A2Z5YG58	Urease subunit alpha	5,4	**60,9**	577	0	NO_SP	1.000052	0.000002	0.000000	0.000000	0.000000	4	7	**426350**	**1365**	**312,31**	**0,00**
A0A2Z5YMZ7	Epimerase domain-containing protein	6,1	**24,3**	228	0	NO_SP	1.000073	0.000000	0.000000	0.000000	0.000000	1	2	**425586,6667**	**0**	**#JAKO/0!**	**0,00**
A0A2Z5YI73	Uncharacterized protein	10,7	**8,6**	81	0	NO_SP	0.719901	0.259574	0.017161	0.000928	0.000615	4	3	**423046,6667**	**9130**	**46,34**	**0,02**
B2HLK2	Lipase LipE	7,9	**45,2**	414	0	NO_SP	1.000074	0.000000	0.000000	0.000000	0.000000	4	5	**421956,6667**	**2561**	**164,73**	**0,00**
A0A2Z5YE77	Methylmalonyl-CoA mutase	4,5	**65,6**	625	0	NO_SP	1.000064	0.000000	0.000000	0.000000	0.000000	4	7	**421140**	**1133**	**371,81**	**0,00**
A0A117DWF4	Membrane protein	4,9	**21,4**	197	4	NO_SP	1.000007	0.000025	0.000000	0.000000	0.000000	4	4	**420866,6667**	**0**	**#JAKO/0!**	**0,00**
B2HPN1	Transcriptional regulatory protein (FadR family)	6,6	**29,9**	273	0	NO_SP	1.000052	0.000000	0.000000	0.000000	0.000000	3	7	**420393,3333**	**0**	**#JAKO/0!**	**0,00**
A0A2Z5YHR1	Acyl-CoA dehydrogenase	4,9	**42,3**	392	0	NO_SP	1.000051	0.000006	0.000000	0.000000	0.000000	3	6	**419230**	**742**	**565,18**	**0,00**

B2HEL7	Carbonic anhydrase	6,5	**79,0**	748	11	NO_SP	1.000026	0.000018	0.000000	0.000000	0.000000	4	4	**418876,6667**	**1522**	**275,30**	**0,00**
B2HFX9	FAD-dependent oxidoreductase	4,7	**43,2**	401	0	NO_SP	1.000038	0.000009	0.000000	0.000000	0.000000	4	7	**418303,3333**	**8139**	**51,39**	**0,02**
B2HME2	Uncharacterized protein	8,1	**27,5**	251	0	NO_SP	1.000045	0.000011	0.000000	0.000000	0.000000	4	5	**418113,3333**	**0**	**#JAKO/0!**	**0,00**
B2HSV7	NADH dehydrogenase	4,9	**42,2**	399	0	NO_SP	0.999042	0.000951	0.000042	0.000002	0.000001	5	7	**417230**	**3139**	**132,91**	**0,00**
A0A2Z5YNW9	FHA domain-containing protein	5,6	**37,0**	348	0	NO_SP	1.000067	0.000001	0.000000	0.000000	0.000000	5	6	**417230**	**0**	**#JAKO/0!**	**0,00**
B2HN31	Oxidoreductase	6,1	**17,1**	162	0	NO_SP	1.000058	0.000003	0.000000	0.000000	0.000000	3	5	**417033,3333**	**0**	**#JAKO/0!**	**0,00**
A0A117DY21	ABC transporter permease	9,3	**35,9**	344	4	NO_SP	0.996411	0.002884	0.000132	0.000030	0.000016	5	5	**416630**	**0**	**#JAKO/0!**	**0,00**
A0A2Z5YAX8	ABC transporter ATP-binding/permease Rv1747	8,4	**93,4**	872	5	NO_SP	1.000051	0.000002	0.000000	0.000000	0.000000	4	8	**415593,3333**	**941**	**441,85**	**0,00**
A0A2Z5YAH3	Alpha/beta hydrolase	5,5	**53,6**	506	0	SP	0.003461	0.502485	0.493519	0.000211	0.000159	4	8	**414606,6667**	**0**	**#JAKO/0!**	**0,00**
A0A2Z5YK11	Luciferase	5,2	**31,1**	290	0	NO_SP	1.000058	0.000001	0.000000	0.000000	0.000000	4	9	**414590**	**0**	**#JAKO/0!**	**0,00**
A0A117DY00	Membrane protein	9,4	**22,8**	213	1	NO_SP	0.999744	0.000273	0.000000	0.000000	0.000000	4	4	**413746,6667**	**7071**	**58,51**	**0,02**
A0A2Z5YDC8	Peptide-methionine (R)-S-oxide reductase	6,3	**16,4**	146	0	NO_SP	1.000052	0.000000	0.000000	0.000000	0.000000	3	4	**413166,6667**	**9597**	**43,05**	**0,02**
B2HID2	Uncharacterized protein	8,9	**14,9**	135	0	NO_SP	1.000034	0.000004	0.000000	0.000000	0.000000	3	3	**411016,6667**	**0**	**#JAKO/0!**	**0,00**
A0A2Z5Y9Z5	Monooxygenase	7,9	**56,1**	502	0	NO_SP	0.995744	0.003719	0.000489	0.000031	0.000010	5	6	**410066,6667**	**0**	**#JAKO/0!**	**0,00**
B2HSK1	Enoyl-CoA hydratase, EchA4	6,3	**33,8**	312	0	NO_SP	1.000053	0.000000	0.000000	0.000000	0.000000	3	6	**409170**	**0**	**#JAKO/0!**	**0,00**
A0A100I8J2	Alpha/beta hydrolase	5,0	**32,7**	303	0	NO_SP	1.000047	0.000000	0.000000	0.000000	0.000000	4	5	**408110**	**0**	**#JAKO/0!**	**0,00**
B2HEA9	Histidine kinase	9,6	**54,6**	511	2	NO_SP	0.974587	0.001273	0.000036	0.000011	0.000007	3	8	**408036,6667**	**5104**	**79,95**	**0,01**
B2HQK1	ATP synthase epsilon chain	4,2	**13,0**	121	0	NO_SP	1.000064	0.000000	0.000000	0.000000	0.000000	3	4	**407856,6667**	**0**	**#JAKO/0!**	**0,00**
B2HER2	Diacylglycerol O-acyltransferase	9,1	**51,4**	472	0	NO_SP	1.000063	0.000000	0.000000	0.000000	0.000000	3	6	**407710**	**491**	**830,54**	**0,00**
B2HNE7	Dihydroorotase	5,0	**45,4**	430	0	NO_SP	1.000064	0.000001	0.000000	0.000000	0.000000	4	6	**406110**	**0**	**#JAKO/0!**	**0,00**
B2HGJ6	Cell division protein FtsX	9,2	**32,8**	297	4	NO_SP	0.776980	0.026599	0.001115	0.000273	0.000141	3	3	**406100**	**735**	**552,19**	**0,00**
B2HEL2	N5-carboxyaminoimidazole ribonucleotide mutase	5,7	**17,6**	171	0	NO_SP	1.000070	0.000000	0.000000	0.000000	0.000000	4	4	**405943,3333**	**841**	**482,86**	**0,00**
B2HJF2	N-acetyltransferase Eis	6,2	**43,9**	405	0	NO_SP	1.000042	0.000000	0.000000	0.000000	0.000000	4	3	**404660**	**129896**	**3,12**	**0,32**
B2HSX9	Haloalkane dehalogenase	6,8	**32,5**	290	0	NO_SP	1.000062	0.000000	0.000000	0.000000	0.000000	3	6	**403613,3333**	**0**	**#JAKO/0!**	**0,00**
B2HK22	Non-specific serine/threonine protein kinase	5,1	**112,8**	1038	0	NO_SP	1.000063	0.000001	0.000000	0.000000	0.000000	7	13	**403503,3333**	**1532**	**263,30**	**0,00**
A0A2Z5Y9D5	Uncharacterized protein	8,4	**47,4**	439	4	NO_SP	0.994296	0.005511	0.000029	0.000012	0.000010	3	7	**403200**	**0**	**#JAKO/0!**	**0,00**
B2HHR3	Uncharacterized protein	5,3	**46,8**	436	0	NO_SP	1.000051	0.000010	0.000000	0.000000	0.000000	4	8	**403106,6667**	**0**	**#JAKO/0!**	**0,00**
A0A2Z5YB99	N-acetylglucosaminyl-diphospho-decaprenol L-rhamnosyltransferase	9,2	**32,7**	294	0	NO_SP	1.000034	0.000000	0.000000	0.000000	0.000000	3	7	**402976,6667**	**1677**	**240,30**	**0,00**
B2HMT0	Uncharacterized protein	3,6	**19,9**	185	0	NO_SP	0.999946	0.000069	0.000004	0.000000	0.000000	4	3	**401699,6667**	**0**	**#JAKO/0!**	**0,00**
A0A100I119	Cytochrome P450 135B1	7,1	**35,1**	321	0	NO_SP	1.000047	0.000000	0.000000	0.000000	0.000000	4	5	**401523,3333**	**0**	**#JAKO/0!**	**0,00**
B2HH98	Uncharacterized protein	7,3	**15,1**	139	0	NO_SP	1.000052	0.000000	0.000000	0.000000	0.000000	4	3	**400986,6667**	**0**	**#JAKO/0!**	**0,00**
B2HS58	DNA-3-methyladenine glycosylase I TagA	8,3	**21,7**	192	0	NO_SP	1.000040	0.000014	0.000000	0.000000	0.000000	3	6	**400803,3333**	**0**	**#JAKO/0!**	**0,00**
A0A2Z5YH82	Lactamase_B domain-containing protein	4,6	**22,3**	207	0	NO_SP	1.000061	0.000000	0.000000	0.000000	0.000000	4	2	**400546,6667**	**0**	**#JAKO/0!**	**0,00**
B2HJ82	DEAD/DEAH box helicase	5,7	**82,1**	771	0	NO_SP	0.964455	0.035197	0.000206	0.000058	0.000031	4	8	**400010**	**9889**	**40,45**	**0,00**
B2HG62	Oxidase	6,1	**58,7**	552	0	TATLIPO	0.000004	0.000005	0.003070	0.004853	0.992055	5	6	**399756,6667**	**2228**	**179,41**	**0,00**
B2HG47	Short-chain type dehydrogenase/reductase	6,0	**29,4**	276	0	NO_SP	1.000052	0.000000	0.000000	0.000000	0.000000	4	5	**398376,6667**	**1832**	**217,50**	**0,00**
A0A2Z5YL38	Membrane protein	10,7	**44,1**	394	4	NO_SP	1.000010	0.000001	0.000000	0.000000	0.000000	4	5	**398243,3333**	**2436**	**163,48**	**0,01**
B2HQF6	Conserved hypothetical membrane protein	8,0	**19,2**	180	4	NO_SP	0.999998	0.000006	0.000000	0.000000	0.000000	3	2	**398000**	**27007**	**14,74**	**0,07**
A0A3E2N1F1	Bacterial regulatory protein, tetR family	10,4	**22,4**	204	0	NO_SP	1.000058	0.000001	0.000000	0.000000	0.000000	3	5	**395760**	**0**	**#JAKO/0!**	**0,00**
A0A2Z5YDR5	Pyridoxamine 5-phosphate oxidase	5,5	**21,3**	191	0	NO_SP	1.000081	0.000001	0.000000	0.000000	0.000000	3	5	**394526,6667**	**0**	**#JAKO/0!**	**0,00**
B2HGG2	PPE family protein, PPE28	3,9	**67,6**	660	1	NO_SP	1.000049	0.000002	0.000000	0.000000	0.000000	5	2	**394106,6667**	**11569**	**34,06**	**0,00**
B2HHC6	Aldehyde dehydrogenase	7,4	**55,5**	515	0	NO_SP	1.000069	0.000003	0.000000	0.000000	0.000000	5	4	**393213,3333**	**12847**	**30,61**	**0,03**
A0A3E2MR56	Uncharacterized protein	8,0	**19,8**	188	1	LIPO	0.000088	0.012107	0.987783	0.000013	0.000031	4	5	**393173,3333**	**4670**	**84,19**	**0,00**
A0A2Z5YJM7	Mycobactin import ATP-binding/permease protein IrtA	6,7	**94,6**	874	6	NO_SP	1.000052	0.000001	0.000000	0.000000	0.000000	4	5	**392983,3333**	**0**	**#JAKO/0!**	**0,00**
A0A2Z5YKN2	Uncharacterized protein	7,2	**46,0**	441	0	NO_SP	0.999736	0.000258	0.000033	0.000001	0.000000	4	7	**392836,6667**	**1278**	**307,33**	**0,00**
A0A2Z5YMW6	Mce family protein Mce4C	6,3	**37,5**	353	1	NO_SP	1.000035	0.000003	0.000000	0.000000	0.000000	4	6	**392323,3333**	**1011**	**387,90**	**0,00**
A0A2Z5Y8W6	Uncharacterized protein	8,0	**12,6**	119	1	NO_SP	0.999945	0.000057	0.000011	0.000000	0.000000	4	2	**391680**	**0**	**#JAKO/0!**	**0,00**
B2HRJ7	Mycobactin synthetase protein B	6,1	**949,6**	8759	0	NO_SP	1.000030	0.000000	0.000000	0.000000	0.000000	4	5	**389560**	**1524**	**255,64**	**0,00**
A0A2Z5YEZ1	Alpha-1,4 glucan phosphorylase	5,3	**94,2**	838	0	NO_SP	0.999683	0.000387	0.000000	0.000000	0.000000	4	8	**388663,3333**	**1883**	**206,39**	**0,00**
B2HSR5	DUF302 domain-containing protein	4,5	**15,0**	141	0	NO_SP	1.000033	0.000000	0.000000	0.000000	0.000000	3	3	**388086,6667**	**940**	**412,81**	**0,00**
A0A2Z5YDJ3	DUF4439 domain-containing protein	8,8	**35,2**	340	1	TATLIPO	0.000000	0.000000	0.000720	0.000020	0.999242	4	4	**385903,3333**	**0**	**#JAKO/0!**	**0,00**
A0A117DUZ0	Amidase	7,4	**36,2**	338	0	NO_SP	0.996634	0.003337	0.000014	0.000007	0.000004	4	7	**385730**	**2850**	**135,35**	**0,01**
A0A2Z5YEM7	Methyltransferase	4,9	**25,7**	236	0	NO_SP	1.000062	0.000002	0.000000	0.000000	0.000000	2	9	**385616,6667**	**0**	**#JAKO/0!**	**0,00**
B2HQ61	Conserved polyketide synthase associated protein PapA3	6,4	**56,1**	502	0	NO_SP	1.000052	0.000001	0.000000	0.000000	0.000000	3	6	**385150**	**0**	**#JAKO/0!**	**0,00**
A0A2Z5YBA9	Uncharacterized protein	4,8	**25,0**	231	0	NO_SP	1.000055	0.000031	0.000000	0.000000	0.000000	3	2	**384960**	**4557**	**84,48**	**0,01**
B2HMW8	Conserved transmembrane protein	7,9	**24,4**	229	3	NO_SP	1.000034	0.000001	0.000000	0.000000	0.000000	4	4	**384826,6667**	**824**	**467,14**	**0,00**
A0A2Z5YBD5	Endopeptidase La	4,5	**33,2**	321	0	NO_SP	0.743225	0.255968	0.000337	0.000168	0.000128	4	9	**384723,3333**	**0**	**#JAKO/0!**	**0,00**
A0A3E2MR48	HTH-type transcriptional regulator CymR	5,7	**17,0**	159	0	NO_SP	1.000036	0.000000	0.000000	0.000000	0.000000	4	4	**384703,3333**	**1725**	**223,06**	**0,00**
A0A2Z5Y8H7	RNA polymerase sigma factor	5,4	**38,8**	353	0	NO_SP	0.999889	0.000158	0.000001	0.000000	0.000000	4	7	**384443,3333**	**557**	**690,82**	**0,00**
A0A2Z5Y8T1	Membrane protein	9,1	**145,5**	1387	9	NO_SP	1.000033	0.000014	0.000000	0.000000	0.000000	4	7	**384403,3333**	**0**	**#JAKO/0!**	**0,00**
A0A2Z5YP05	ESX-1 secretion-associated protein EspL	4,5	**12,3**	115	0	NO_SP	1.000047	0.000000	0.000000	0.000000	0.000000	4	4	**383603,3333**	**1133**	**338,45**	**0,00**
A0A2Z5YJM5	Heme chaperone HemW	5,0	**43,7**	405	0	NO_SP	0.996969	0.003047	0.000020	0.000005	0.000003	4	5	**383303,3333**	**0**	**#JAKO/0!**	**0,00**
A0A100IF85	CysQ protein	5,1	**24,9**	237	0	NO_SP	1.000033	0.000001	0.000000	0.000000	0.000000	3	3	**382933,3333**	**0**	**#JAKO/0!**	**0,00**
B2HRQ7	Protease HtpX homolog	10,0	**30,9**	286	4	NO_SP	1.000044	0.000000	0.000000	0.000000	0.000000	4	4	**381643,3333**	**7077**	**53,93**	**0,00**
B2HJT4	Nickel cation-binding GTPase, HypB	4,9	**27,9**	255	0	NO_SP	0.999759	0.000249	0.000015	0.000000	0.000000	4	5	**380560**	**0**	**#JAKO/0!**	**0,00**
A0A2Z5YNW1	Chromosome partitioning protein ParA	4,8	**25,9**	235	0	NO_SP	1.000067	0.000000	0.000000	0.000000	0.000000	1	5	**380020**	**0**	**#JAKO/0!**	**0,00**
B2HS00	Trk system potassium uptake protein TrkA	4,8	**25,5**	240	0	NO_SP	1.000071	0.000001	0.000000	0.000000	0.000000	3	4	**379396,6667**	**750**	**506,15**	**0,00**
A0A2Z5YCG1	Acylphosphatase	5,2	**25,1**	230	0	NO_SP	1.000034	0.000002	0.000000	0.000000	0.000000	4	8	**379396,6667**	**0**	**#JAKO/0!**	**0,00**
B2HNU6	AHS1 domain-containing protein	6,2	**24,1**	223	0	NO_SP	1.000039	0.000001	0.000000	0.000000	0.000000	3	4	**375953,3333**	**1992**	**188,74**	**0,00**
A0A2Z5Y9Q4	Molybdopterin molybdenumtransferase	5,2	**41,6**	401	0	NO_SP	1.000026	0.000005	0.000000	0.000000	0.000000	3	5	**375156,6667**	**2824**	**132,85**	**0,00**
A0A2Z5Y857	Membrane protein	6,7	**105,3**	970	11	NO_SP	0.999976	0.000053	0.000000	0.000000	0.000000	4	6	**373476,6667**	**0**	**#JAKO/0!**	**0,00**
B2HJE6	Transcriptional regulator	8,0	**17,5**	163	0	NO_SP	1.000046	0.000000	0.000000	0.000000	0.000000	3	3	**373060**	**0**	**#JAKO/0!**	**0,00**
A0A2Z5YGA6	Uncharacterized protein	6,1	**124,2**	1118	0	NO_SP	1.000029	0.000000	0.000000	0.000000	0.000000	1	11	**372033,3333**	**357**	**1040,66**	**0,00**
A0A2Z5YAM3	DNA-directed RNA polymerase subunit beta	4,7	**124,2**	1126	0	NO_SP	1.000032	0.000000	0.000000	0.000000	0.000000	13	2	**370615,3333**	**379**	**977,70**	**0,00**
A0A2Z5YA52	Acyl-CoA synthetase	5,5	**56,8**	528	0	NO_SP	1.000037	0.000000	0.000000	0.000000	0.000000	4	4	**370403,3333**	**250**	**1481,85**	**0,00**
A0A100IEE5	Deazaflavin-dependent oxidoreductase, nitroreductase family protein	6,6	**13,1**	119	0	NO_SP	1.000072	0.000000	0.000000	0.000000	0.000000	4	2	**369926,6667**	**48603**	**7,61**	**0,13**
A0A2Z5YCA2	Uncharacterized protein	4,4	**14,3**	128	0	NO_SP	1.000067	0.000000	0.000000	0.000000	0.000000	3	3	**368570**	**0**	**#JAKO/0!**	**0,00**

B2HQE8	3-oxoacyl-[acyl-carrier protein] reductase FabG	5,9	25,9	248	0	NO_SP	1.000079	0.000001	0.000000	0.000000	0.000000	4	6	367516,6667	2323	158,19	0,00
B2HI79	Osmoprotectant (Glycine betaine/carnitine/choline/L-proline) transport ATP-l	4,7	43,5	399	0	NO_SP	1.000041	0.000001	0.000000	0.000000	0.000000	4	8	367250	0	#JAKO/0!	0,00
B2HGV5	Conserved hypothetical glycosyltransferase	9,8	41,7	385	0	NO_SP	1.000073	0.000000	0.000000	0.000000	0.000000	4	6	366680	8823	41,56	0,02
A0A117DYW1	TetR family transcriptional regulator	4,7	21,9	197	0	NO_SP	1.000038	0.000000	0.000000	0.000000	0.000000	3	3	365930	0	#JAKO/0!	0,00
A0A2Z5YDN7	Uncharacterized protein	4,8	31,1	294	1	NO_SP	0.999838	0.000189	0.000000	0.000000	0.000000	4	3	365450	0	#JAKO/0!	0,00
A0A2Z5YGN6	Uncharacterized protein	5,4	10,0	95	0	NO_SP	1.000034	0.000001	0.000000	0.000000	0.000000	4	4	365410	0	#JAKO/0!	0,00
B2HP64	Aminoglycosides/tetracycline-transport integral membrane protein	7,0	54,3	517	14	NO_SP	1.000033	0.000000	0.000000	0.000000	0.000000	4	3	364743,3333	9439	38,64	0,03
A0A2Z5YL00	Uncharacterized protein	5,5	72,7	658	0	NO_SP	1.000071	0.000000	0.000000	0.000000	0.000000	4	7	364106,6667	0	#JAKO/0!	0,00
A0A2Z5YDY7	Pyridoxal 5-phosphate synthase subunit PdxT	6,8	20,5	191	0	NO_SP	1.000057	0.000001	0.000000	0.000000	0.000000	3	2	363966,6667	0	#JAKO/0!	0,00
B2HEC4	Phosphoribosylglycinamide formyltransferase	7,1	22,2	215	0	NO_SP	1.000058	0.000005	0.000000	0.000000	0.000000	4	4	363716,6667	1607	226,37	0,00
B2HNT6	Foldase YidC	10,3	41,3	371	5	NO_SP	0.999369	0.000645	0.000008	0.000000	0.000000	4	6	363696,6667	9691	37,53	0,03
B2HNY8	PKS_ER domain-containing protein	4,7	36,0	339	0	NO_SP	1.000045	0.000000	0.000000	0.000000	0.000000	3	4	363266,6667	2292	158,50	0,00
A0A3E2N0A6	Putative L,D-transpeptidase LppS	4,8	44,6	420	0	LIPO	0.012079	0.003317	0.984583	0.000008	0.000013	4	3	362380	3470	104,44	0,01
A0A2Z5YIV7	Ribonuclease Z	5,1	29,2	279	0	NO_SP	1.000057	0.000000	0.000000	0.000000	0.000000	4	5	361363,3333	0	#JAKO/0!	0,00
B2HM55	Uncharacterized protein	6,5	46,3	436	0	NO_SP	1.000047	0.000000	0.000000	0.000000	0.000000	4	4	360853,3333	0	#JAKO/0!	0,00
A0A3E2MRE9	Carboxymethylenebutenolidase	7,3	24,8	236	0	NO_SP	1.000052	0.000002	0.000000	0.000000	0.000000	4	2	360496,6667	1443	249,88	0,00
B2HE81	Conserved hypothetical O-methyltransferase	4,7	24,4	227	0	NO_SP	1.000026	0.000004	0.000000	0.000000	0.000000	4	3	359163,3333	0	#JAKO/0!	0,00
B2HRM7	UDP-glucose 4-epimerase, GalE3	6,6	36,4	346	0	NO_SP	0.999869	0.000157	0.000003	0.000000	0.000000	4	5	358890	777	462,19	0,00
A0A2Z5YE58	Quinolinate phosphoribosyltransferase [decarboxylating]	4,7	30,3	285	0	NO_SP	1.000063	0.000006	0.000000	0.000000	0.000000	3	3	358330	11961	29,96	0,03
A0A2Z5YE22	Putative F420H(2)-dependent quinone reductase	5,3	16,4	145	0	NO_SP	1.000065	0.000000	0.000000	0.000000	0.000000	3	9	358046,6667	975	367,27	0,00
A0A2Z5YL45	Polyketide cyclase	10,1	17,1	151	0	NO_SP	1.000067	0.000000	0.000000	0.000000	0.000000	3	3	357910	0	#JAKO/0!	0,00
B2HNW1	Putative S-adenosyl-L-methionine-dependent methyltransferase MMAR_0539	4,5	32,8	301	0	NO_SP	0.999958	0.000099	0.000001	0.000000	0.000000	4	7	357823,3333	24862	14,39	0,07
B2HJU8	Uncharacterized protein	6,9	15,6	144	0	NO_SP	1.000067	0.000000	0.000000	0.000000	0.000000	3	3	357500	3507	101,94	0,01
B2HSY3	Uncharacterized protein	5,9	37,3	350	3	NO_SP	0.983170	0.015579	0.000139	0.000104	0.000067	4	5	357223,3333	1558	229,26	0,00
B2HQ45	SAM-dependent methyltransferase	5,3	24,2	218	0	NO_SP	1.000052	0.000001	0.000000	0.000000	0.000000	3	4	357113,3333	0	#JAKO/0!	0,00
A0A2Z5YMR9	Uncharacterized protein	9,7	7,8	67	1	NO_SP	1.000011	0.000026	0.000000	0.000000	0.000000	4	1	354823,3333	16432	21,59	0,05
B2HRF4	Uncharacterized protein	10,5	17,0	150	0	NO_SP	1.000046	0.000000	0.000000	0.000000	0.000000	3	5	354073,3333	29887	11,85	0,08
A0A3E2MX49	4-demethylrebeccamycin synthase	5,9	39,8	388	0	NO_SP	0.999975	0.000081	0.000001	0.000000	0.000000	5	5	353710	0	#JAKO/0!	0,00
B2HNH2	DSBA domain-containing protein	4,7	22,8	207	0	NO_SP	1.000058	0.000000	0.000000	0.000000	0.000000	3	4	351560	0	#JAKO/0!	0,00
A0A100HZ65	Succinate dehydrogenase	7,5	12,1	108	3	NO_SP	1.000047	0.000000	0.000000	0.000000	0.000000	4	2	351163,3333	0	#JAKO/0!	0,00
A0A124BUY3	Acetyltransferase	4,1	23,0	216	0	NO_SP	0.999987	0.000044	0.000001	0.000000	0.000000	4	2	350626,6667	0	#JAKO/0!	0,00
A0A124BUV9	NADP-dependent malic enzyme	4,5	37,1	359	0	NO_SP	1.000059	0.000003	0.000000	0.000000	0.000000	4	5	350596,6667	0	#JAKO/0!	0,00
B2HQA7	Inositol-monophosphatase ImpA	4,7	28,1	270	0	NO_SP	1.000078	0.000000	0.000000	0.000000	0.000000	3	4	350270	0	#JAKO/0!	0,00
A0A2Z5YP85	HTH tetR-type domain-containing protein	4,4	20,8	197	0	NO_SP	1.000047	0.000000	0.000000	0.000000	0.000000	1	5	349653,3333	0	#JAKO/0!	0,00
B2HFV7	Prokaryotic ubiquitin-like protein Pup	3,8	6,9	64	0	NO_SP	1.000030	0.000001	0.000000	0.000000	0.000000	4	3	349050	13754	25,38	0,04
A0A3E2MVB6	Uncharacterized protein	10,4	26,5	235	0	NO_SP	0.998580	0.001340	0.000058	0.000008	0.000004	4	2	347793,3333	1618	214,99	0,00
A0A100IA38	Cytokinin riboside 5-monophosphate phosphoribohydrolase	4,5	17,3	161	0	NO_SP	1.000057	0.000000	0.000000	0.000000	0.000000	4	5	347096,6667	996	348,56	0,00
A0A100ICE6	Lipoprotein	5,2	40,3	380	0	LIPO	0.015524	0.000062	0.984429	0.000000	0.000000	4	3	347086,6667	0	#JAKO/0!	0,00
B2HT55	Membrane glycine and proline rich protein	4,3	32,8	326	1	NO_SP	1.000064	0.000001	0.000000	0.000000	0.000000	4	2	347060	0	#JAKO/0!	0,00
A0A2Z5YMQ8	Uncharacterized protein	8,1	38,9	355	0	NO_SP	1.000057	0.000002	0.000000	0.000000	0.000000	4	3	346683,3333	10545	32,88	0,00
A0A2Z5YHN4	Dehydrogenase	4,8	48,7	461	0	NO_SP	1.000052	0.000001	0.000000	0.000000	0.000000	4	3	346600	0	#JAKO/0!	0,00
A0A2Z5YF18	Transcriptional regulator	6,1	20,0	184	0	NO_SP	1.000054	0.000000	0.000000	0.000000	0.000000	3	5	346516,6667	316	1097,65	0,00
B2HS50	Unidentified antibiotic-transport ATP-binding protein ABC transporter	6,1	34,0	315	0	NO_SP	1.000062	0.000000	0.000000	0.000000	0.000000	4	4	345326,6667	0	#JAKO/0!	0,00
A0A2Z5YH33	Putative dipeptidase PepE	4,6	39,2	375	0	NO_SP	0.999967	0.000083	0.000001	0.000000	0.000000	4	3	345053,3333	489	705,53	0,00
B2HIZ7	DSBA domain-containing protein	4,8	22,0	203	0	NO_SP	1.000036	0.000003	0.000000	0.000000	0.000000	5	6	344750	0	#JAKO/0!	0,00
B2HQ12	Uncharacterized protein	5,5	7,7	70	0	NO_SP	1.000057	0.000000	0.000000	0.000000	0.000000	3	2	344690	0	#JAKO/0!	0,00
A0A2Z5YNP8	VOC domain-containing protein	4,3	15,3	139	0	NO_SP	1.000071	0.000000	0.000000	0.000000	0.000000	4	4	344333,3333	2185	157,62	0,00
A0A2Z5Y7Q6	NADPH:quinone reductase	5,0	33,2	313	0	NO_SP	1.000049	0.000000	0.000000	0.000000	0.000000	4	5	343920	3098	111,02	0,00
A0A2Z5YKS2	1,4-alpha-glucan branching enzyme GlgB	5,2	81,5	731	0	NO_SP	1.000057	0.000001	0.000000	0.000000	0.000000	4	8	343463,3333	558	615,56	0,00
A0A2Z5YKD3	Uncharacterized protein	4,1	23,8	220	1	NO_SP	0.999853	0.000105	0.000013	0.000001	0.000000	3	3	342360	0	#JAKO/0!	0,00
B2HD73	Cytochrome P450 140A5 Cyp140A5	8,0	48,8	438	0	NO_SP	1.000048	0.000001	0.000000	0.000000	0.000000	4	5	342053,3333	0	#JAKO/0!	0,00
B2HID9	Methyltransf_11 domain-containing protein	4,3	29,9	272	0	NO_SP	1.000047	0.000001	0.000000	0.000000	0.000000	3	4	340943,3333	4705	72,47	0,01
A0A2Z5Y7J9	Putative HTH-type transcriptional regulator	8,9	28,5	254	0	NO_SP	1.000048	0.000000	0.000000	0.000000	0.000000	4	5	340723,3333	0	#JAKO/0!	0,00
A0A2Z5YC95	Membrane protein	9,4	71,7	698	12	NO_SP	0.986048	0.013091	0.000398	0.000144	0.000076	3	6	340320	2488	136,80	0,01
A0A2Z5YIR8	Mycobactin synthetase protein B	5,0	164,3	1512	0	NO_SP	1.000052	0.000000	0.000000	0.000000	0.000000	4	9	340046,6667	828	410,63	0,00
B2HSC8	O-methyltransferase Omt_2	8,7	30,7	278	0	NO_SP	1.000046	0.000010	0.000001	0.000000	0.000000	4	6	339700	2736	124,15	0,00
A0A2Z5YGN1	Aldehyde dehydrogenase	6,0	56,1	517	0	NO_SP	0.999148	0.000825	0.000050	0.000003	0.000001	4	4	338930	471	718,99	0,00
B2HCV4	Protein translocase subunit SecY	9,7	47,7	441	9	NO_SP	0.685289	0.313696	0.000306	0.000218	0.000204	3	5	338916,6667	0	#JAKO/0!	0,00
A0A2Z5YM05	Uncharacterized protein	7,2	17,4	158	1	NO_SP	0.945773	0.053433	0.000242	0.000241	0.000120	4	5	338136,6667	3143	107,60	0,00
B2HNY7	Conserved membrane protein	5,1	24,4	239	1	NO_SP	1.000032	0.000000	0.000000	0.000000	0.000000	3	1	337966,6667	7129	47,40	0,02
A0A2Z5YGE1	DUF2314 domain-containing protein	4,5	46,0	424	0	NO_SP	1.000030	0.000009	0.000000	0.000000	0.000000	3	2	336820	0	#JAKO/0!	0,00
A0A2Z5YHH7	Alkanal monooxygenase alpha chain	5,1	36,4	335	0	NO_SP	1.000057	0.000007	0.000000	0.000000	0.000000	3	6	336250	0	#JAKO/0!	0,00
A0A100I5Z3	DNA-binding protein	7,8	13,6	122	0	NO_SP	1.000057	0.000000	0.000000	0.000000	0.000000	3	5	336050	0	#JAKO/0!	0,00
B2HLQ6	Coenzyme F420-dependent oxidoreductase	6,7	37,7	352	0	NO_SP	1.000003	0.000041	0.000001	0.000000	0.000000	4	4	336006,6667	0	#JAKO/0!	0,00
B2HLB0	Uncharacterized protein	5,9	15,7	146	0	NO_SP	1.000062	0.000000	0.000000	0.000000	0.000000	4	5	335880	0	#JAKO/0!	0,00
A0A2Z5YGP0	Phthiotriol/phenolphthiotriol dimycocerosates methyltransferase	7,0	29,6	259	0	NO_SP	1.000067	0.000000	0.000000	0.000000	0.000000	4	3	335776,6667	2645	126,95	0,00
B2HLR6	SnoaL-like domain-containing protein	4,6	18,9	168	0	NO_SP	1.000006	0.000027	0.000001	0.000000	0.000000	4	2	335775	0	#JAKO/0!	0,00
A0A3E2MQ11	Uncharacterized protein	9,4	40,4	368	0	NO_SP	1.000056	0.000000	0.000000	0.000000	0.000000	4	4	335770	5038	66,65	0,02
B2HQI1	DUF2017 domain-containing protein	5,3	21,6	195	0	NO_SP	1.000046	0.000000	0.000000	0.000000	0.000000	4	4	335466,6667	4610	72,78	0,01
A0A100I0E8	3-dehydroquinate dehydratase	6,2	15,2	142	1	NO_SP	1.000046	0.000001	0.000000	0.000000	0.000000	4	3	334946,6667	1140	293,80	0,00
B2HN62	Holliday junction ATP-dependent DNA helicase RuvA	6,2	20,3	197	0	NO_SP	1.000041	0.000001	0.000000	0.000000	0.000000	3	5	334776,6667	19908	16,82	0,06
B2HDV9	Uracil phosphoribosyltransferase	4,8	21,8	207	0	NO_SP	1.000036	0.000004	0.000000	0.000000	0.000000	4	3	333820	0	#JAKO/0!	0,00
A0A2Z5YMQ7	Bifunctional cephalosporin acylase/gamma-glutamyltranspeptidase	6,2	56,9	533	0	NO_SP	1.000074	0.000004	0.000000	0.000000	0.000000	4	5	333586,6667	1452	229,76	0,00
A0A2Z5YE71	DUF4178 domain-containing protein	4,3	22,0	202	1	NO_SP	1.000064	0.000000	0.000000	0.000000	0.000000	3	3	332576,6667	2497	133,18	0,00
B2HD66	Bacterioferritin	4,3	18,3	159	0	NO_SP	1.000083	0.000000	0.000000	0.000000	0.000000	4	6	331866,6667	19099	17,38	0,06
A0A2Z5YDN4	Putative gluconeogenesis factor	7,7	37,6	358	0	NO_SP	1.000056	0.000000	0.000000	0.000000	0.000000	4	7	331850	0	#JAKO/0!	0,00
B2HR33	Argininosuccinate lyase	4,9	49,9	470	0	NO_SP	1.000026	0.000005	0.000000	0.000000	0.000000	4	5	331320	6399	51,78	0,02

B2HEE0	DNA-(apurinic or apyrimidinic site) lyase	9,5	**30,4**	287	0	NO_SP	1.000026	0.000005	0.000000	0.000000	0.000000	4	7	**330220**	**0**	**#JAKO/0!**	**0,00**
A0A2Z5YM53	Biotin carboxyl carrier protein	4,8	**54,0**	504	0	NO_SP	1.000063	0.000003	0.000000	0.000000	0.000000	1	1	**330126,6667**	**226060**	**1,46**	**0,68**
B2HP79	PlsC domain-containing protein	6,1	**29,4**	267	0	NO_SP	1.000059	0.000000	0.000000	0.000000	0.000000	4	2	**329838,3333**	**0**	**#JAKO/0!**	**0,00**
A0A2Z5YF04	Lipid-transfer protein	5,7	**42,4**	400	0	NO_SP	1.000051	0.000000	0.000000	0.000000	0.000000	3	7	**329660**	**0**	**#JAKO/0!**	**0,00**
A0A2Z5YJN9	Uncharacterized protein	4,8	**69,7**	653	0	NO_SP	1.000072	0.000000	0.000000	0.000000	0.000000	3	8	**328406,6667**	**0**	**#JAKO/0!**	**0,00**
B2HLS6	Putative S-adenosyl-L-methionine-dependent methyltransferase MMAR_0358	4,4	**34,1**	311	0	NO_SP	0.999931	0.000099	0.000001	0.000000	0.000000	3	5	**327960**	**2066**	**158,78**	**0,00**
A0A3E2MSZ8	3-alpha-(Or 20-beta)-hydroxysteroid dehydrogenase	6,5	**27,9**	267	0	NO_SP	1.000056	0.000001	0.000000	0.000000	0.000000	4	4	**327903,3333**	**0**	**#JAKO/0!**	**0,00**
B2HNA7	Conserved protein	5,9	**19,4**	171	0	NO_SP	1.000048	0.000000	0.000000	0.000000	0.000000	4	3	**327253,3333**	**0**	**#JAKO/0!**	**0,00**
A0A2Z5YBI6	Uncharacterized protein	6,5	**42,3**	404	1	NO_SP	0.946933	0.043180	0.001165	0.000486	0.000237	3	9	**327093,3333**	**0**	**#JAKO/0!**	**0,00**
B2HP45	Orotidine 5-phosphate decarboxylase	4,4	**27,8**	278	0	NO_SP	1.000036	0.000024	0.000000	0.000000	0.000000	4	3	**326860**	**0**	**#JAKO/0!**	**0,00**
A0A3E2N1T6	Dihydropteroate synthase	5,1	**28,6**	275	0	NO_SP	1.000041	0.000005	0.000000	0.000000	0.000000	4	4	**325976,6667**	**13691**	**23,81**	**0,04**
A0A2Z5YDQ4	Uncharacterized protein	9,1	**24,8**	223	0	NO_SP	1.000060	0.000001	0.000000	0.000000	0.000000	4	6	**325753,3333**	**1318**	**247,17**	**0,00**
B2HKY3	Acyl-CoA dehydrogenase FadE18_1	4,8	**37,6**	367	0	NO_SP	1.000057	0.000000	0.000000	0.000000	0.000000	4	6	**325736,6667**	**0**	**#JAKO/0!**	**0,00**
B2HJ50	2-amino-4-hydroxy-6-hydroxymethyldihydropteridine pyrophosphokinase	5,2	**18,7**	175	0	NO_SP	1.000054	0.000001	0.000000	0.000000	0.000000	3	3	**324566,6667**	**3243**	**100,09**	**0,01**
B2HLP9	Acyl-CoA dehydrogenase FadE1	6,8	**48,4**	431	0	NO_SP	1.000062	0.000000	0.000000	0.000000	0.000000	4	6	**324416,6667**	**0**	**#JAKO/0!**	**0,00**
A0A2Z5YD69	Catalase-related peroxidase	8,9	**33,4**	315	0	NO_SP	1.000032	0.000000	0.000000	0.000000	0.000000	4	5	**324350**	**2622**	**123,71**	**0,01**
A0A2Z5YDU9	Putative GTPase	6,4	**36,3**	336	0	NO_SP	1.000046	0.000004	0.000000	0.000000	0.000000	4	4	**323460**	**0**	**#JAKO/0!**	**0,00**
A0A3E2MR16	Homoserine kinase	4,7	**32,6**	314	0	NO_SP	0.990110	0.009880	0.000019	0.000022	0.000010	4	4	**322680**	**1198**	**269,43**	**0,00**
B2HJV7	Two-component transcriptional regulatory protein	5,2	**23,3**	217	0	NO_SP	1.000053	0.000000	0.000000	0.000000	0.000000	3	3	**320990**	**2740**	**117,15**	**0,01**
A0A2Z5Y9E8	Uncharacterized protein	5,1	**14,6**	139	0	NO_SP	1.000033	0.000000	0.000000	0.000000	0.000000	4	3	**320700**	**0**	**#JAKO/0!**	**0,00**
A0A124BW14	Phosphate regulon sensor protein	6,3	**43,9**	406	1	NO_SP	0.999942	0.000072	0.000002	0.000001	0.000000	4	9	**320313,3333**	**0**	**#JAKO/0!**	**0,00**
A0A2Z5YL01	IclR family transcriptional regulator	4,3	**27,7**	267	0	NO_SP	1.000063	0.000000	0.000000	0.000000	0.000000	4	9	**319593,3333**	**0**	**#JAKO/0!**	**0,00**
A0A2Z5YDB3	Uncharacterized protein	8,2	**19,8**	181	0	NO_SP	1.000073	0.000000	0.000000	0.000000	0.000000	4	5	**318880**	**0**	**#JAKO/0!**	**0,00**
B2HRB7	Dehydrogenase fad flavoprotein Gmc oxidoreductase	6,5	**56,9**	529	0	NO_SP	1.000038	0.000001	0.000000	0.000000	0.000000	4	5	**318613,3333**	**0**	**#JAKO/0!**	**0,00**
B2HR30	Ornithine carbamoyltransferase	5,1	**33,4**	308	0	NO_SP	1.000080	0.000000	0.000000	0.000000	0.000000	4	4	**318543,3333**	**0**	**#JAKO/0!**	**0,00**
A0A2Z5YFY9	Peptidyl-prolyl cis-trans isomerase	7,6	**19,7**	202	0	SP	0.142375	0.817796	0.036009	0.002610	0.000702	3	3	**318423,3333**	**0**	**#JAKO/0!**	**0,00**
A0A3E2MXT3	Acyl-CoA dehydrogenase	6,4	**47,2**	412	0	NO_SP	1.000061	0.000000	0.000000	0.000000	0.000000	4	8	**316756,6667**	**30637**	**10,34**	**0,10**
A0A3E2N0U5	Lipase 2	8,3	**26,6**	250	0	NO_SP	1.000049	0.000002	0.000000	0.000000	0.000000	4	3	**316756,6667**	**437**	**725,45**	**0,00**
A0A100IB28	Putative glutamine amidotransferase	4,9	**24,5**	232	0	NO_SP	1.000035	0.000004	0.000000	0.000000	0.000000	4	4	**316443,3333**	**0**	**#JAKO/0!**	**0,00**
A0A3E2MWD9	Putative ligase	5,1	**58,0**	549	0	NO_SP	1.000058	0.000007	0.000000	0.000000	0.000000	4	7	**316120**	**0**	**#JAKO/0!**	**0,00**
A0A100I3L7	Ribonuclease J	6,3	**58,2**	543	0	NO_SP	1.000051	0.000000	0.000000	0.000000	0.000000	4	6	**315253,3333**	**1058**	**297,89**	**0,00**
A0A3E2MX71	PAC2 family protein	4,3	**35,8**	325	0	NO_SP	1.000055	0.000000	0.000000	0.000000	0.000000	4	6	**315050**	**0**	**#JAKO/0!**	**0,00**
A0A3E2MPK8	Coproporphyrinogen III oxidase	5,7	**47,1**	455	0	NO_SP	1.000068	0.000001	0.000000	0.000000	0.000000	3	7	**314900**	**1552**	**202,92**	**0,00**
A0A2Z5YCI6	Transport permease protein	9,2	**30,0**	276	6	NO_SP	0.999925	0.000096	0.000000	0.000000	0.000000	4	2	**314503,3333**	**8177**	**38,46**	**0,03**
A0A2Z5YAS3	Oxidoreductase	8,5	**48,9**	483	0	TAT	0.000000	0.000000	0.000000	0.999989	0.000000	4	7	**312976,6667**	**2241**	**139,64**	**0,00**
A0A100I1N3	Conserved regulatory protein	6,1	**19,3**	178	0	NO_SP	0.999708	0.000250	0.000043	0.000001	0.000000	3	4	**311903,3333**	**0**	**#JAKO/0!**	**0,00**
A0A2Z5YME4	Deazaflavin-dependent nitroreductase	10,1	**17,3**	151	0	NO_SP	1.000051	0.000001	0.000000	0.000000	0.000000	3	5	**311656,6667**	**18949**	**16,45**	**0,06**
A0A2Z5Y9A0	Beta-lactamase domain-containing protein	5,0	**43,5**	403	0	LIPO	0.000001	0.000000	1.000083	0.000000	0.000000	4	3	**311343,3333**	**0**	**#JAKO/0!**	**0,00**
B2HI07	Cytochrome P450 142A3 Cyp142A3	4,5	**45,1**	401	0	NO_SP	1.000047	0.000003	0.000000	0.000000	0.000000	4	7	**311096,6667**	**3798**	**81,91**	**0,01**
A0A2Z5Y8L5	tRNA (guanine-N(7)-)-methyltransferase	8,5	**29,6**	272	0	NO_SP	1.000064	0.000001	0.000000	0.000000	0.000000	4	4	**310396,6667**	**3203**	**96,89**	**0,00**
A0A100I262	tRNA-2-methylthio-N(6)-dimethylallyladenosine synthase	6,6	**52,2**	490	0	NO_SP	1.000034	0.000004	0.000000	0.000000	0.000000	4	5	**309836,6667**	**0**	**#JAKO/0!**	**0,00**
B2HS78	Uncharacterized protein	5,2	**16,1**	142	0	NO_SP	0.999999	0.000033	0.000000	0.000000	0.000000	3	2	**308170**	**215514**	**1,43**	**0,70**
B2HH15	Conserved protein	5,8	**28,7**	270	1	NO_SP	0.897484	0.088640	0.005043	0.000589	0.000336	4	3	**306203,3333**	**0**	**#JAKO/0!**	**0,00**
A0A2Z5YBF6	Acetyl-CoA acetyltransferase	6,5	**40,7**	393	0	NO_SP	1.000049	0.000003	0.000000	0.000000	0.000000	3	4	**306146,6667**	**0**	**#JAKO/0!**	**0,00**
A0A2Z5Y966	Inner membrane protein YqjA	7,5	**25,0**	230	5	NO_SP	1.000030	0.000002	0.000000	0.000000	0.000000	3	2	**306126,6667**	**0**	**#JAKO/0!**	**0,00**
B2HM23	Uroporphyrinogen decarboxylase	6,3	**37,6**	353	0	NO_SP	1.000047	0.000000	0.000000	0.000000	0.000000	4	4	**305850**	**0**	**#JAKO/0!**	**0,00**
A0A117DUQ7	Conserved lipoprotein, LppF	4,5	**44,1**	420	0	LIPO	0.199116	0.308045	0.490457	0.000827	0.000725	4	6	**304860**	**0**	**#JAKO/0!**	**0,00**
B2HPT2	Ketoacyl reductase	6,3	**26,9**	257	0	NO_SP	1.000074	0.000001	0.000000	0.000000	0.000000	3	4	**304630**	**4456**	**68,37**	**0,01**
B2HF05	2,3-dihydroxybiphenyl-1,2-dioxygenase BphC_1	6,5	**42,9**	390	0	NO_SP	1.000066	0.000000	0.000000	0.000000	0.000000	1	2	**304080**	**137290**	**2,21**	**0,45**
B2HQ91	ATP-dependent dethiobiotin synthetase BioD	4,7	**22,7**	226	0	NO_SP	1.000025	0.000014	0.000001	0.000000	0.000000	3	3	**304070**	**9046**	**33,61**	**0,03**
A0A2Z5YN66	Hercynine oxygenase	5,1	**48,3**	433	0	NO_SP	1.000037	0.000000	0.000000	0.000000	0.000000	4	6	**303760**	**0**	**#JAKO/0!**	**0,00**
B2HKC1	Gamma-glutamyl-hercynylcysteine sulfoxide hydrolase	4,7	**25,5**	237	0	NO_SP	0.999772	0.000261	0.000005	0.000001	0.000000	4	4	**303583,3333**	**0**	**#JAKO/0!**	**0,00**
B2HIS2	Diacylglycerol O-acyltransferase	6,7	**50,0**	466	0	NO_SP	1.000048	0.000000	0.000000	0.000000	0.000000	5	5	**303284**	**0**	**#JAKO/0!**	**0,00**
A0A2Z5YN71	2-C-methyl-D-erythritol 4-phosphate cytidylyltransferase	6,8	**23,3**	227	0	NO_SP	1.000043	0.000002	0.000000	0.000000	0.000000	3	6	**302700**	**0**	**#JAKO/0!**	**0,00**
A0A2Z5YB91	CoA transferase	6,4	**42,4**	395	0	NO_SP	1.000042	0.000002	0.000000	0.000000	0.000000	3	5	**302080**	**1151**	**262,40**	**0,00**
A0A3E2MMR9	RNA polymerase sigma factor SigF	5,0	**27,0**	241	0	NO_SP	1.000060	0.000000	0.000000	0.000000	0.000000	4	3	**301983,3333**	**5944**	**50,80**	**0,02**
A0A117DY22	Ammonium transporter	5,6	**48,4**	471	11	NO_SP	1.000030	0.000000	0.000000	0.000000	0.000000	3	2	**301953,3333**	**10577**	**28,55**	**0,04**
A0A2Z5YMQ0	Uncharacterized protein	5,3	**25,2**	231	0	NO_SP	0.827349	0.169344	0.001749	0.000791	0.000335	1	5	**301843,3333**	**34364**	**8,78**	**0,11**
A0A2Z5YNS0	DUF4328 domain-containing protein	6,2	**28,1**	262	4	NO_SP	0.999975	0.000016	0.000000	0.000000	0.000000	4	4	**301573,3333**	**0**	**#JAKO/0!**	**0,00**
A0A3E2N2J5	Glycerol kinase	4,7	**55,1**	508	0	NO_SP	1.000061	0.000000	0.000000	0.000000	0.000000	4	7	**300820**	**0**	**#JAKO/0!**	**0,00**
A0A100IHL8	Conserved hypothetical transmembrane protein	9,1	**33,0**	310	5	NO_SP	0.916938	0.036055	0.003005	0.026945	0.015460	4	4	**299743,3333**	**0**	**#JAKO/0!**	**0,00**
B2HMT9	Conserved hypothetical integral membrane protein YrbE1B	9,2	**30,4**	289	5	NO_SP	1.000054	0.000001	0.000000	0.000000	0.000000	3	2	**299706,6667**	**0**	**#JAKO/0!**	**0,00**
B2HEV3	ZnMc domain-containing protein	7,1	**37,6**	341	0	NO_SP	0.999467	0.000488	0.000039	0.000005	0.000002	3	4	**299503,3333**	**0**	**#JAKO/0!**	**0,00**
A0A2Z5Y980	Uncharacterized protein	6,0	**27,8**	252	0	NO_SP	1.000004	0.000048	0.000000	0.000000	0.000000	4	5	**299384,3333**	**189**	**1583,35**	**0,00**
B2HPV5	Conserved membrane protein	4,6	**29,5**	271	1	NO_SP	0.999202	0.000715	0.000087	0.000003	0.000001	4	3	**299260**	**0**	**#JAKO/0!**	**0,00**
B2HEW6	1-aminocyclopropane-1-carboxylate deaminase	9,9	**35,5**	329	0	NO_SP	1.000060	0.000000	0.000000	0.000000	0.000000	4	5	**299193,3333**	**6733**	**44,43**	**0,02**
A0A2Z5YIM6	Cutinase	4,5	**22,4**	221	0	SP	0.007063	0.991862	0.000347	0.000272	0.000214	4	2	**298686,6667**	**0**	**#JAKO/0!**	**0,00**
A0A2Z5YJH4	X-Pro dipeptidyl-peptidase	6,2	**65,5**	596	0	NO_SP	0.999956	0.000094	0.000001	0.000000	0.000000	4	6	**298140**	**281**	**1062,05**	**0,00**
B2HSB0	5-amino-6-(D-ribitylamino)uracil--L-tyrosine 4-hydroxyphenyl transferase	6,4	**92,1**	856	0	NO_SP	1.000023	0.000014	0.000000	0.000000	0.000000	4	7	**296516,6667**	**468**	**633,90**	**0,00**
A0A3E2MXT6	Putative response regulatory protein	4,6	**14,3**	133	0	NO_SP	1.000075	0.000000	0.000000	0.000000	0.000000	4	2	**296206,6667**	**8436**	**35,11**	**0,03**
B2HNK4	Conserved protein	7,0	**18,4**	168	0	NO_SP	1.000051	0.000000	0.000000	0.000000	0.000000	5	2	**296173,3333**	**2104**	**140,78**	**0,01**
A0A3E2N2X9	GTP cyclohydrolase 1 type 2 homolog	4,9	**39,7**	378	0	NO_SP	1.000040	0.000009	0.000000	0.000000	0.000000	4	5	**295883,3333**	**3074**	**96,27**	**0,01**
A0A2Z5YB15	NADH oxidase	8,6	**43,0**	397	0	NO_SP	1.000052	0.000001	0.000000	0.000000	0.000000	3	7	**295883,3333**	**0**	**#JAKO/0!**	**0,00**
B2HMW1	Uncharacterized protein	7,9	**10,5**	96	0	NO_SP	1.000060	0.000000	0.000000	0.000000	0.000000	3	2	**295871,2333**	**7656**	**38,64**	**0,03**
A0A2Z5YH85	zf-RING_7 domain-containing protein	4,7	**26,9**	245	0	NO_SP	1.000052	0.000000	0.000000	0.000000	0.000000	4	5	**295803,3333**	**3125**	**94,66**	**0,01**
B2HEC7	Conserved hypothetical oxidoreductase	5,8	**30,3**	277	0	NO_SP	0.999496	0.000461	0.000054	0.000003	0.000002	3	4	**295753,3333**	**0**	**#JAKO/0!**	**0,00**
A0A2Z5YD20	Phosphatidyl-myo-inositol mannosyltransferase	5,6	**39,7**	370	0	NO_SP	1.000083	0.000000	0.000000	0.000000	0.000000	4	5	**295563,3333**	**0**	**#JAKO/0!**	**0,00**

A0A2Z5YL31	STAS domain protein	8,1	**27,1**	254	0	NO_SP	0.999969	0.000058	0.000001	0.000000	0.000000	3	6	**294810**	**12190**	**24,18**	**0,00**
A0A2Z5YD37	Antibiotic biosynthesis monooxygenase	4,4	**11,0**	104	0	NO_SP	1.000049	0.000012	0.000000	0.000000	0.000000	3	4	**294190**	**0**	**#JAKO/0!**	**0,00**
A0A2Z5YB52	Lipoprotein LpqB	5,3	**61,3**	586	0	LIPO	0.000000	0.000000	1.000051	0.000000	0.000000	4	5	**294046,6667**	**0**	**#JAKO/0!**	**0,00**
A0A2Z5YLG6	LysR family transcriptional regulator	4,6	**26,1**	250	0	NO_SP	1.000017	0.000035	0.000002	0.000000	0.000000	3	1	**294013,3333**	**0**	**#JAKO/0!**	**0,00**
A0A2Z5YG83	Uncharacterized protein	5,6	**24,4**	223	0	NO_SP	1.000069	0.000000	0.000000	0.000000	0.000000	1	4	**293960**	**0**	**#JAKO/0!**	**0,00**
B2HK74	Conserved hypothetical Zn-dependent hydrolase	5,0	**28,8**	268	0	NO_SP	1.000031	0.000010	0.000000	0.000000	0.000000	5	5	**293830**	**0**	**#JAKO/0!**	**0,00**
B2HJP9	1-deoxy-D-xylulose 5-phosphate reductoisomerase	4,5	**41,8**	402	0	NO_SP	1.000068	0.000002	0.000000	0.000000	0.000000	4	4	**293563,3333**	**464**	**632,45**	**0,00**
A0A2Z5YIN4	3-oxoacyl-(Acyl carrier protein) synthase III	5,2	**34,2**	316	0	NO_SP	1.000055	0.000000	0.000000	0.000000	0.000000	3	5	**293110**	**0**	**#JAKO/0!**	**0,00**
B2HSX4	Conserved hypothetical regulatory protein	7,0	**26,6**	251	0	NO_SP	1.000048	0.000000	0.000000	0.000000	0.000000	3	5	**292740**	**0**	**#JAKO/0!**	**0,00**
Q9L8K4	PE-PGRS homolog MAG24-2	3,8	**54,6**	556	0	NO_SP	0.999967	0.000080	0.000000	0.000000	0.000000	5	3	**292357,3333**	**0**	**#JAKO/0!**	**0,00**
A0A2Z5Y967	Steroid monooxygenase	4,8	**92,2**	850	0	NO_SP	0.999811	0.000222	0.000011	0.000001	0.000000	4	7	**292036,6667**	**2506**	**116,53**	**0,01**
A0A2Z5Y9C3	Uncharacterized protein	7,1	**21,9**	211	0	NO_SP	1.000063	0.000000	0.000000	0.000000	0.000000	3	5	**291600**	**11216**	**26,00**	**0,00**
B2HRJ4	Delta-aminolevulinic acid dehydratase	4,6	**34,5**	327	0	NO_SP	1.000050	0.000001	0.000000	0.000000	0.000000	4	4	**290780**	**14428**	**20,15**	**0,05**
B2HKZ4	Short chain dehydrogenase	4,5	**28,5**	277	0	NO_SP	1.000047	0.000004	0.000000	0.000000	0.000000	4	4	**290561**	**0**	**#JAKO/0!**	**0,00**
A0A100I0C5	Ribosom_S30AE_C domain-containing protein	10,0	**28,3**	258	0	NO_SP	0.999972	0.000074	0.000000	0.000000	0.000000	2	1	**289620**	**0**	**#JAKO/0!**	**0,00**
B2HLM9	Uncharacterized protein	4,7	**43,2**	406	0	NO_SP	1.000051	0.000010	0.000000	0.000000	0.000000	4	7	**289256**	**0**	**#JAKO/0!**	**0,00**
A0A2Z5YM91	Diacylglycerol O-acyltransferase	7,2	**29,7**	277	0	NO_SP	1.000067	0.000000	0.000000	0.000000	0.000000	1	2	**289254**	**0**	**#JAKO/0!**	**0,00**
A0A2Z5YI64	Putative polyketide synthase MbtD	4,9	**108,9**	1024	0	NO_SP	1.000048	0.000006	0.000001	0.000000	0.000000	4	9	**289073,3333**	**2916**	**99,13**	**0,00**
B2HSQ4	Conserved membrane protein	4,3	**20,8**	206	0	LIPO	0.000000	0.000001	1.000078	0.000000	0.000000	2	5	**288843,3333**	**0**	**#JAKO/0!**	**0,00**
A0A2Z5YLS1	SIR2_2 domain-containing protein	5,4	**32,1**	287	0	NO_SP	1.000069	0.000000	0.000000	0.000000	0.000000	1	5	**288556,6667**	**0**	**#JAKO/0!**	**0,00**
B2HQR1	Conserved transmembrane transport protein MmpL5_2	6,8	**105,8**	965	11	NO_SP	1.000038	0.000012	0.000000	0.000000	0.000000	4	5	**288536,6667**	**0**	**#JAKO/0!**	**0,00**
A0A2Z5YBU7	Alkyldihydroxyacetonephosphate synthase	6,1	**56,7**	530	0	NO_SP	1.000039	0.000000	0.000000	0.000000	0.000000	4	6	**288400**	**73693**	**3,91**	**0,00**
B2HK91	STAS domain-containing protein	5,7	**13,9**	129	0	NO_SP	1.000048	0.000000	0.000000	0.000000	0.000000	3	4	**288226,6667**	**0**	**#JAKO/0!**	**0,00**
A0A2Z5YFD2	EthD domain-containing protein	4,6	**25,6**	237	0	NO_SP	1.000046	0.000001	0.000000	0.000000	0.000000	4	6	**287840**	**7133**	**40,36**	**0,02**
A0A2Z5YES3	Aldose 1-epimerase	6,3	**42,2**	397	0	LIPO	0.000000	0.000005	1.000015	0.000000	0.000016	4	4	**287150**	**0**	**#JAKO/0!**	**0,00**
A0A100I1X1	Metallophosphoesterase	7,4	**34,8**	297	0	NO_SP	1.000032	0.000000	0.000000	0.000000	0.000000	3	3	**287116,6667**	**0**	**#JAKO/0!**	**0,00**
B2HRM6	S-methyl-5-thioadenosine phosphorylase	6,3	**28,1**	264	0	NO_SP	1.000066	0.000000	0.000000	0.000000	0.000000	4	3	**286760**	**0**	**#JAKO/0!**	**0,00**
A0A2Z5YAT0	Coenzyme F420:L-glutamate ligase	6,5	**24,0**	214	0	NO_SP	1.000045	0.000000	0.000000	0.000000	0.000000	3	5	**286236,6667**	**1627**	**175,98**	**0,00**
B2HGA7	L-asparagine permease AnsP1	9,2	**53,0**	494	12	NO_SP	1.000028	0.000014	0.000000	0.000000	0.000000	4	6	**286106,6667**	**2745**	**104,24**	**0,00**
A0A100IC87	Mammalian cell entry protein	5,2	**47,1**	443	1	NO_SP	0.991633	0.007710	0.000252	0.000056	0.000030	4	4	**285720**	**7418**	**38,52**	**0,03**
A0A2Z5YAE9	Uncharacterized protein	7,4	**24,0**	234	0	LIPO	0.001239	0.154454	0.844186	0.000103	0.000030	2	2	**285326,6667**	**929**	**307,07**	**0,00**
A0A2Z5YBE4	Succinate dehydrogenase	9,8	**17,9**	155	2	NO_SP	0.999975	0.000049	0.000000	0.000000	0.000000	3	2	**285103,3333**	**15292**	**18,64**	**0,05**
A0A2Z5YIQ5	Universal stress protein	4,8	**26,9**	254	0	NO_SP	1.000053	0.000001	0.000000	0.000000	0.000000	4	5	**284740**	**0**	**#JAKO/0!**	**0,00**
A0A100I1P2	Short-chain dehydrogenase	7,6	**29,3**	282	0	NO_SP	1.000089	0.000006	0.000000	0.000000	0.000000	4	3	**283960**	**8377**	**33,90**	**0,03**
A0A2Z5Y7U3	(R)-benzylsuccinyl-CoA dehydrogenase	6,8	**45,1**	407	0	NO_SP	1.000028	0.000000	0.000000	0.000000	0.000000	3	6	**283663,3333**	**0**	**#JAKO/0!**	**0,00**
A0A2Z5YP22	Uncharacterized protein	4,9	**29,6**	273	0	NO_SP	1.000050	0.000000	0.000000	0.000000	0.000000	1	2	**283663,3333**	**0**	**#JAKO/0!**	**0,00**
A0A3E2MX06	4-phosphopantetheinyl transferase Npt	5,1	**24,2**	223	0	NO_SP	1.000046	0.000002	0.000000	0.000000	0.000000	4	3	**283646,6667**	**0**	**#JAKO/0!**	**0,00**
A0A2Z5Y8J0	Long-chain-fatty-acid--CoA ligase	5,1	**59,4**	549	0	NO_SP	1.000003	0.000047	0.000000	0.000000	0.000000	4	5	**282863,3333**	**0**	**#JAKO/0!**	**0,00**
A0A2Z5YKI9	Uncharacterized protein	4,2	**25,8**	241	1	NO_SP	1.000050	0.000002	0.000000	0.000000	0.000000	4	2	**282656,6667**	**0**	**#JAKO/0!**	**0,00**
A0A100I358	2-nitropropane dioxygenase	4,4	**34,3**	332	0	NO_SP	1.000053	0.000001	0.000000	0.000000	0.000000	4	4	**282023,3333**	**0**	**#JAKO/0!**	**0,00**
A0A100IG11	Glycerol-3-phosphate ABC transporter	8,0	**31,3**	278	4	NO_SP	0.719403	0.273772	0.002730	0.001190	0.001217	3	2	**281816,6667**	**17076**	**16,50**	**0,06**
A0A3E2N2C1	Putative phosphoserine phosphatase 2	6,2	**22,4**	202	0	NO_SP	0.999988	0.000043	0.000009	0.000000	0.000000	4	4	**281750**	**6000**	**46,96**	**0,02**
A0A2Z5YKW2	Phosphate-binding protein PstS	5,1	**34,8**	332	0	NO_SP	0.999864	0.000162	0.000001	0.000000	0.000000	4	5	**281113,3333**	**0**	**#JAKO/0!**	**0,00**
B2HQT8	Conserved transmembrane protein	6,9	**47,5**	427	3	NO_SP	0.999997	0.000017	0.000000	0.000000	0.000000	5	5	**281096,6667**	**14322**	**19,63**	**0,05**
A0A2Z5YP79	LLM class F420-dependent oxidoreductase	4,9	**30,9**	283	0	NO_SP	1.000069	0.000003	0.000000	0.000000	0.000000	3	5	**280679**	**0**	**#JAKO/0!**	**0,00**
A0A2Z5YDU8	Uncharacterized protein	6,5	**15,4**	144	0	NO_SP	1.000060	0.000000	0.000000	0.000000	0.000000	5	4	**280326,6667**	**0**	**#JAKO/0!**	**0,00**
A0A2Z5YGA2	Phosphotransferase	4,5	**72,8**	666	0	NO_SP	1.000082	0.000000	0.000000	0.000000	0.000000	4	7	**279636,6667**	**0**	**#JAKO/0!**	**0,00**
A0A100I0T4	Peptide methionine sulfoxide reductase MsrA	5,6	**17,5**	155	0	NO_SP	1.000061	0.000000	0.000000	0.000000	0.000000	4	5	**279916,6667**	**0**	**#JAKO/0!**	**0,00**
A0A2Z5YEY6	Uncharacterized protein	8,8	**46,3**	428	0	NO_SP	1.000070	0.000000	0.000000	0.000000	0.000000	4	4	**278900**	**1254**	**222,35**	**0,00**
A0A124BV17	Zn-dependent hydrolase	7,2	**33,0**	303	0	NO_SP	0.999951	0.000087	0.000001	0.000000	0.000000	4	3	**278276,6667**	**5630**	**49,43**	**0,02**
B2HDQ8	Robl_LC7 domain-containing protein	5,7	**15,1**	143	0	NO_SP	1.000021	0.000002	0.000000	0.000000	0.000000	4	3	**277490**	**0**	**#JAKO/0!**	**0,00**
B2HT70	Conserved protein	4,1	**10,4**	99	1	NO_SP	1.000044	0.000018	0.000000	0.000000	0.000000	3	1	**277086,6667**	**0**	**#JAKO/0!**	**0,00**
Q8GAR7	60 kDa chaperonin (Fragment)	4,7	**57,5**	548	0	NO_SP	1.000058	0.000000	0.000000	0.000000	0.000000	1	2	**277050**	**0**	**#JAKO/0!**	**0,00**
A0A2Z5YLG4	Putative acyl-CoA dehydrogenase FadE10	6,7	**70,7**	650	0	NO_SP	1.000046	0.000000	0.000000	0.000000	0.000000	4	5	**276663,3333**	**0**	**#JAKO/0!**	**0,00**
A0A2Z5Y8W7	Uncharacterized protein	5,0	**44,9**	409	0	NO_SP	0.999739	0.000301	0.000005	0.000000	0.000000	4	5	**276553,3333**	**0**	**#JAKO/0!**	**0,00**
B2HS56	Glycosyl transferase	5,9	**41,1**	387	0	NO_SP	1.000055	0.000000	0.000000	0.000000	0.000000	5	4	**276390**	**727**	**380,25**	**0,00**
A0A2Z5YC99	Heme-binding protein	6,2	**22,9**	213	0	NO_SP	0.999603	0.000350	0.000054	0.000002	0.000001	3	4	**275566,6667**	**811**	**340,00**	**0,00**
A0A2Z5YIR4	Dihydrolipoamide acetyltransferase component of pyruvate dehydrogenase c	9,0	**43,6**	413	0	NO_SP	1.000053	0.000000	0.000000	0.000000	0.000000	4	4	**275329**	**0**	**#JAKO/0!**	**0,00**
A0A100I801	Membrane protein	5,5	**14,7**	125	0	NO_SP	0.999251	0.000739	0.000017	0.000003	0.000002	4	5	**275043,3333**	**0**	**#JAKO/0!**	**0,00**
A0A2Z5YPB9	Non-specific serine/threonine protein kinase	6,2	**78,2**	736	1	NO_SP	1.000047	0.000003	0.000000	0.000000	0.000000	2	4	**274491,3333**	**0**	**#JAKO/0!**	**0,00**
B2HI25	4-hydroxy-2-oxovalerate aldolase 2	5,6	**36,8**	347	0	NO_SP	1.000052	0.000000	0.000000	0.000000	0.000000	3	6	**274440**	**0**	**#JAKO/0!**	**0,00**
B2HP53	Lipase LipH	4,5	**34,5**	323	0	NO_SP	1.000036	0.000000	0.000000	0.000000	0.000000	3	4	**274110**	**0**	**#JAKO/0!**	**0,00**
A0A2Z5YB51	FMN-binding glutamate synthase family protein	7,3	**53,6**	509	0	NO_SP	1.000047	0.000001	0.000000	0.000000	0.000000	4	5	**271817,3333**	**0**	**#JAKO/0!**	**0,00**
A0A2Z5YNK8	Nucleoid-associated protein	5,9	**14,9**	132	0	NO_SP	1.000027	0.000000	0.000000	0.000000	0.000000	3	4	**271343,3333**	**10733**	**25,28**	**0,04**
A0A117DWH7	Cell division protein FtsQ	5,0	**32,9**	307	1	NO_SP	1.000074	0.000000	0.000000	0.000000	0.000000	4	4	**270860,6667**	**185**	**1464,19**	**0,00**
B2HPU8	Ethanolamine ammonia-lyase large subunit, EutB	4,4	**49,5**	469	0	NO_SP	1.000014	0.000024	0.000002	0.000000	0.000000	4	5	**270513,3333**	**0**	**#JAKO/0!**	**0,00**
B2HNR1	Membrane-anchored serine protease (Mycosin), MycP3_1	5,6	**47,7**	468	2	SP	0.012797	0.984022	0.002384	0.000293	0.000249	3	6	**270296,6667**	**203**	**1331,29**	**0,00**
B2HSX5	Conserved protein	4,1	**18,0**	164	0	NO_SP	1.000044	0.000000	0.000000	0.000000	0.000000	3	5	**269913,3333**	**2068**	**130,51**	**0,00**
A0A2Z5YCJ7	Carrier domain-containing protein	4,6	**64,4**	604	0	NO_SP	1.000041	0.000003	0.000000	0.000000	0.000000	3	2	**269473,3333**	**1017**	**264,93**	**0,00**
A0A2Z5Y7I6	UPF0301 protein MMRN_00520	6,1	**19,5**	179	0	NO_SP	1.000046	0.000000	0.000000	0.000000	0.000000	1	1	**269340**	**0**	**#JAKO/0!**	**0,00**
A0A100IAZ2	Uncharacterized protein	7,9	**21,0**	194	0	NO_SP	1.000041	0.000010	0.000000	0.000000	0.000000	4	6	**268600**	**540**	**497,50**	**0,00**
A0A2Z5YEH0	Adenylate cyclase	8,6	**26,7**	250	0	NO_SP	0.999745	0.000294	0.000002	0.000000	0.000000	4	5	**268520**	**0**	**#JAKO/0!**	**0,00**
A0A3E2MMR7	Cocaine esterase	8,6	**63,0**	568	0	NO_SP	1.000023	0.000020	0.000000	0.000000	0.000000	4	5	**268443,3333**	**0**	**#JAKO/0!**	**0,00**
B2HSA4	Aminotransferase	6,0	**38,3**	365	0	NO_SP	1.000052	0.000001	0.000000	0.000000	0.000000	3	1	**268423,3333**	**0**	**#JAKO/0!**	**0,00**
A0A2Z5Y9M1	Cyclopropane mycolic acid synthase 2	4,9	**35,0**	303	0	NO_SP	1.000050	0.000000	0.000000	0.000000	0.000000	4	5	**268106,6667**	**0**	**#JAKO/0!**	**0,00**
B2HIK8	Formyltetrahydrofolate deformylase	6,6	**32,9**	298	0	NO_SP	1.000046	0.000002	0.000000	0.000000	0.000000	3	5	**267911,6667**	**0**	**#JAKO/0!**	**0,00**
B2HQH3	Glutamate racemase	5,1	**28,6**	271	0	NO_SP	1.000046	0.000001	0.000000	0.000000	0.000000	3	7	**266450**	**0**	**#JAKO/0!**	**0,00**

B2HFU3	Conserved alanine and proline rich protein	4,5	86,2	858	0	NO_SP	0.998327	0.001716	0.000003	0.000002	0.000001	3	1	266030	0	#JAKO/0!	0,00
A0A3E2MTR7	2-dehydropantoate 2-reductase	6,4	30,8	300	0	NO_SP	1.000044	0.000011	0.000000	0.000000	0.000000	4	4	265410	6779	39,15	0,03
B2HQ24	Uncharacterized protein	4,7	33,5	302	0	NO_SP	1.000036	0.000000	0.000000	0.000000	0.000000	3	6	265235,3333	992	267,45	0,00
B2HKB7	Uncharacterized protein	4,7	55,3	493	0	NO_SP	1.000079	0.000000	0.000000	0.000000	0.000000	2	5	265054,3333	0	#JAKO/0!	0,00
B2HJL5	tRNA (guanine-N(1)-)-methyltransferase	5,0	25,5	230	0	NO_SP	1.000040	0.000000	0.000000	0.000000	0.000000	4	5	265023,3333	0	#JAKO/0!	0,00
A0A2Z5YFD7	Anti-sigma factor antagonist	8,5	15,0	143	0	NO_SP	1.000012	0.000057	0.000000	0.000000	0.000000	2	4	264480	0	#JAKO/0!	0,00
B2HHS5	Uncharacterized protein	4,7	18,1	170	0	NO_SP	0.999982	0.000081	0.000002	0.000000	0.000000	3	2	264326,6667	2180	121,27	0,01
A0A2Z5YCQ9	F5/8 type C domain-containing protein	6,1	98,4	904	1	SP	0.149947	0.477736	0.011344	0.337527	0.022692	4	8	264193,3333	167	1580,04	0,00
B2HM12	Antibiotic-transport ATP-binding protein ABC transporter	5,6	32,5	299	0	NO_SP	1.000078	0.000000	0.000000	0.000000	0.000000	5	5	263380	0	#JAKO/0!	0,00
A0A2Z5YGR0	Uncharacterized protein	5,1	60,2	535	0	NO_SP	1.000054	0.000000	0.000000	0.000000	0.000000	1	5	262490	79115	3,32	0,30
A0A2Z5YB16	Amidohydrolase	4,7	59,7	551	0	NO_SP	1.000050	0.000000	0.000000	0.000000	0.000000	4	5	261865,6667	0	#JAKO/0!	0,00
B2HJK3	Conserved hypothetical alanine and arginine rich protein	6,6	63,0	578	0	NO_SP	1.000038	0.000001	0.000000	0.000000	0.000000	4	5	261850	0	#JAKO/0!	0,00
A0A3E2MXX0	F420-dependent glucose-6-phosphate dehydrogenase	6,1	35,9	332	0	NO_SP	0.994396	0.005358	0.000190	0.000027	0.000009	4	6	261766,6667	0	#JAKO/0!	0,00
B2HQK8	ATP synthase subunit a	6,3	27,4	250	5	NO_SP	1.000062	0.000003	0.000000	0.000000	0.000000	4	3	261570	0	#JAKO/0!	0,00
A0A2Z5YHV2	Uncharacterized protein	4,6	42,5	375	0	NO_SP	1.000053	0.000003	0.000000	0.000000	0.000000	2	6	261506,6667	811	322,61	0,00
B2HRC4	Transmembrane ATP-binding protein ABC transporter	10,1	69,0	640	5	NO_SP	0.999854	0.000156	0.000000	0.000000	0.000000	4	2	261017,7333	0	#JAKO/0!	0,00
B2HSW3	Conserved hypothetical flavoprotein	5,6	50,4	480	1	NO_SP	0.982764	0.005346	0.011753	0.000047	0.000016	4	5	260990	7548	34,58	0,03
A0A2Z5YDR4	Preprotein translocase subunit YajC	4,1	12,5	113	1	NO_SP	0.999731	0.000305	0.000006	0.000002	0.000001	3	3	260860	0	#JAKO/0!	0,00
A0A3E2MYZ2	Pyruvate dehydrogenase E1 component	6,0	103,3	929	0	NO_SP	1.000036	0.000004	0.000000	0.000000	0.000000	3	1	258040	352963	0,73	1,37
A0A100IHB0	SAM-dependent methyltransferase	4,6	22,9	204	0	NO_SP	1.000030	0.000000	0.000000	0.000000	0.000000	4	4	257421	0	#JAKO/0!	0,00
B2HI19	Conserved hypothetical oxidoreductase	4,9	45,1	394	0	NO_SP	1.000042	0.000000	0.000000	0.000000	0.000000	4	4	256743,3333	0	#JAKO/0!	0,00
B2HDW1	Purine nucleoside phosphorylase	6,1	27,8	273	0	NO_SP	1.000015	0.000008	0.000000	0.000000	0.000000	3	3	256600	0	#JAKO/0!	0,00
A0A2Z5YBM0	Uncharacterized protein	6,9	27,1	244	0	NO_SP	1.000002	0.000041	0.000000	0.000000	0.000000	3	3	256567,3333	2366	108,44	0,01
A0A100I085	Selenide, water dikinase	4,6	31,2	305	0	NO_SP	0.999978	0.000049	0.000000	0.000000	0.000000	4	3	256433,3333	0	#JAKO/0!	0,00
A0A2Z5Y851	Acyl-CoA synthetase	4,6	55,6	531	0	NO_SP	1.000047	0.000000	0.000000	0.000000	0.000000	4	7	256392,6667	1378	186,07	0,01
A0A2Z5YI55	CHAD domain-containing protein	9,8	45,0	413	0	NO_SP	1.000052	0.000004	0.000000	0.000000	0.000000	1	1	256123,3333	5749	44,55	0,00
A0A2Z5Y9V1	2-succinyl-5-enolpyruvyl-6-hydroxy-3-cyclohexene-1-carboxylate synthase	6,7	54,9	526	0	NO_SP	0.984027	0.012580	0.003257	0.000058	0.000032	4	6	256003,3333	0	#JAKO/0!	0,00
A0A2Z5YJJ0	Putative ATP-dependent helicase DinG homolog	5,0	70,6	673	0	NO_SP	1.000044	0.000011	0.000000	0.000000	0.000000	3	7	255906,6667	965	265,32	0,00
B2HM85	Mycobactin synthetase protein B	4,7	126,2	1163	0	NO_SP	1.000063	0.000001	0.000000	0.000000	0.000000	4	7	255800	0	#JAKO/0!	0,00
A0A2Z5YMD6	Acyl-CoA dehydrogenase	4,5	38,1	365	0	NO_SP	1.000036	0.000000	0.000000	0.000000	0.000000	4	5	255710	2507	102,01	0,00
B2HSF4	Putative S-adenosyl-L-methionine-dependent methyltransferase MMAR_0955	4,5	34,7	313	0	NO_SP	1.000038	0.000003	0.000000	0.000000	0.000000	4	4	255353,3333	0	#JAKO/0!	0,00
A0A2Z5Y9X6	C-type cytochrome biogenesis protein CcsB	9,9	36,0	328	8	NO_SP	1.000079	0.000001	0.000000	0.000000	0.000000	2	2	255300	0	#JAKO/0!	0,00
A0A2Z5YNV8	Mce family protein Mce4B	5,0	34,6	326	0	NO_SP	1.000055	0.000001	0.000000	0.000000	0.000000	4	3	254633,3333	5070	50,22	0,02
A0A2Z5YLB4	Phytase-like domain-containing protein	4,5	49,4	472	0	NO_SP	0.999885	0.000156	0.000002	0.000000	0.000000	4	4	254573,3333	0	#JAKO/0!	0,00
B2HRW9	Conserved hypothetical membrane protein	10,0	29,2	263	6	NO_SP	1.000075	0.000000	0.000000	0.000000	0.000000	4	1	254506,6667	0	#JAKO/0!	0,00
A0A2Z5YHG8	Methyltransf_11 domain-containing protein	8,6	23,5	208	0	NO_SP	1.000053	0.000000	0.000000	0.000000	0.000000	3	5	254140	0	#JAKO/0!	0,00
A0A2Z5YM48	Uncharacterized protein	7,9	18,5	178	1	NO_SP	1.000051	0.000003	0.000000	0.000000	0.000000	4	2	253945	0	#JAKO/0!	0,00
B2HES0	Uncharacterized protein	5,5	35,1	330	0	NO_SP	1.000087	0.000001	0.000000	0.000000	0.000000	3	3	253903,3333	0	#JAKO/0!	0,00
A0A2Z5Y8L9	NYN domain-containing protein	4,4	26,4	245	0	NO_SP	1.000036	0.000006	0.000000	0.000000	0.000000	4	6	252236,6667	0	#JAKO/0!	0,00
A0A2Z5YG55	Uncharacterized protein	9,0	32,2	292	0	NO_SP	0.999681	0.000313	0.000008	0.000001	0.000001	3	5	252203,3333	546	461,77	0,00
A0A2Z5YMZ5	Acyl-CoA dehydrogenase	5,5	42,9	388	0	NO_SP	1.000053	0.000000	0.000000	0.000000	0.000000	4	4	251833,3333	2296	109,66	0,00
B2HQF1	Transmembrane ATP-binding protein ABC transporter	6,4	61,0	579	5	NO_SP	1.000067	0.000002	0.000000	0.000000	0.000000	4	5	251710	6810	36,96	0,00
B2HGI1	Two-component transcriptional regulatory protein DevR	5,9	23,4	216	0	NO_SP	1.000026	0.000001	0.000000	0.000000	0.000000	3	3	251700	3916	64,27	0,02
B2HT66	Isoprenyl transferase	5,0	29,7	262	0	NO_SP	1.000062	0.000000	0.000000	0.000000	0.000000	4	4	251536,6667	1345	187,02	0,00
A0A3E2N2S0	Putative Nudix hydrolase NudL	5,1	27,1	252	0	NO_SP	1.000034	0.000001	0.000000	0.000000	0.000000	4	4	250536,6667	113	2222,12	0,00
A0A100I2X3	3-oxoacyl-ACP synthase	7,0	38,4	359	0	NO_SP	1.000046	0.000000	0.000000	0.000000	0.000000	3	4	250448,3333	0	#JAKO/0!	0,00
B2HHT7	DapB_N domain-containing protein	4,6	39,2	361	0	NO_SP	1.000053	0.000000	0.000000	0.000000	0.000000	3	5	250206,6667	0	#JAKO/0!	0,00
B2HT89	PNPLA domain-containing protein	4,9	37,1	354	0	NO_SP	1.000026	0.000002	0.000000	0.000000	0.000000	4	5	250033,3333	7582	32,98	0,00
A0A100I6G3	Glutamine ABC transporter	4,9	34,0	313	0	LIPO	0.000011	0.000069	0.999974	0.000000	0.000000	4	5	249650	1235	202,17	0,00
B2HHQ9	Peroxiredoxin AhpE	4,6	16,7	153	0	NO_SP	0.999844	0.000173	0.000005	0.000000	0.000000	4	2	249323,3333	1416	176,09	0,00
A0A2Z5YEB9	Putative L-asparaginase	4,7	32,3	318	0	NO_SP	0.999976	0.000065	0.000000	0.000000	0.000000	4	8	248270,3333	0	#JAKO/0!	0,00
B2HNU4	Aminoglycoside 2-N-acetyltransferase Aac	6,0	19,7	181	0	NO_SP	1.000037	0.000006	0.000000	0.000000	0.000000	3	3	247933,3333	0	#JAKO/0!	0,00
B2HGZ9	Putative S-adenosyl-L-methionine-dependent methyltransferase MMAR_4850	4,3	33,1	301	0	NO_SP	0.877523	0.120699	0.001024	0.000322	0.000172	4	4	247756,6667	0	#JAKO/0!	0,00
A0A2Z5YC03	Thiamine-monophosphate kinase	4,4	27,9	273	0	NO_SP	1.000058	0.000003	0.000000	0.000000	0.000000	4	6	247563,3333	0	#JAKO/0!	0,00
A0A2Z5YPG7	Histidine kinase	7,5	53,7	504	2	NO_SP	0.923062	0.067290	0.000658	0.000594	0.000282	2	4	246816,6667	0	#JAKO/0!	0,00
B2HRR5	UPF0234 protein MMAR_0920	6,0	18,1	163	0	NO_SP	1.000049	0.000000	0.000000	0.000000	0.000000	4	6	246346,6667	4475	55,05	0,00
A0A3E2MT44	Isopentenyl-diphosphate delta-isomerase	5,4	36,5	348	0	NO_SP	1.000047	0.000002	0.000000	0.000000	0.000000	4	5	246273,3333	956	257,62	0,00
B2HLI7	19 kDa lipoprotein antigen LpqH	4,6	9,6	99	0	NO_SP	0.999194	0.000813	0.000031	0.000001	0.000001	3	1	245600	0	#JAKO/0!	0,00
A0A2Z5YKM5	Dihydropteroate synthase	6,2	30,9	291	0	NO_SP	1.000011	0.000029	0.000002	0.000000	0.000000	4	4	245403,3333	0	#JAKO/0!	0,00
A0A2Z5YBY2	Protein adenylyltransferase SelO	4,8	53,0	487	0	NO_SP	0.999918	0.000103	0.000007	0.000000	0.000000	4	7	245296,6667	810	302,72	0,00
A0A2Z5YL46	Carboxylic ester hydrolase	8,6	56,9	529	0	NO_SP	1.000058	0.000000	0.000000	0.000000	0.000000	4	6	245100	0	#JAKO/0!	0,00
B2HEB3	Acyl-CoA dehydrogenase FadE13	5,7	40,8	382	0	NO_SP	1.000052	0.000000	0.000000	0.000000	0.000000	4	6	245043,3333	0	#JAKO/0!	0,00
A0A100IB02	Alpha/beta hydrolase	5,3	18,2	173	0	NO_SP	1.000052	0.000003	0.000000	0.000000	0.000000	4	4	245030	0	#JAKO/0!	0,00
A0A2Z5YJF5	DUF3817 domain-containing protein	9,8	13,4	120	3	NO_SP	1.000031	0.000002	0.000000	0.000000	0.000000	1	1	244996,6667	0	#JAKO/0!	0,00
B2HEU1	DAGKc domain-containing protein	9,6	34,3	321	0	NO_SP	1.000049	0.000000	0.000000	0.000000	0.000000	4	4	244900	1515	161,64	0,01
A0A2Z5YE65	Membrane protein	5,3	22,2	215	4	NO_SP	1.000056	0.000000	0.000000	0.000000	0.000000	4	3	243806,6667	0	#JAKO/0!	0,00
B2HJS0	Hydrogenase-forming protein, HypD	6,7	41,8	382	0	NO_SP	1.000034	0.000000	0.000000	0.000000	0.000000	4	5	242070	0	#JAKO/0!	0,00
A0A3E2MS30	L-lysine N6-monooxygenase MbtG	5,2	47,2	434	0	NO_SP	0.999962	0.000080	0.000001	0.000000	0.000000	4	3	241363,3333	0	#JAKO/0!	0,00
A0A2Z5YMA2	Copper-sensing transcriptional repressor CsoR	6,9	12,8	117	0	NO_SP	1.000048	0.000000	0.000000	0.000000	0.000000	3	3	241343,3333	2122	113,72	0,00
A0A117DX00	Uncharacterized protein	10,7	10,8	102	0	NO_SP	0.983064	0.015631	0.001180	0.000031	0.000021	3	2	240890	15278	15,77	0,06
A0A2Z5YK05	GP-PDE domain-containing protein	4,9	29,9	282	0	NO_SP	0.999560	0.000479	0.000001	0.000001	0.000000	4	3	240436,6667	0	#JAKO/0!	0,00
A0A2Z5YM76	Mce associated protein	10,0	17,2	157	1	NO_SP	1.000020	0.000010	0.000006	0.000000	0.000000	4	2	240426,6667	0	#JAKO/0!	0,00
B2HR05	Conserved protein	4,6	14,4	130	0	NO_SP	1.000066	0.000000	0.000000	0.000000	0.000000	3	4	239983,3333	1110	216,14	0,00
A0A2Z5YFM3	Uncharacterized protein	5,0	29,2	270	0	NO_SP	0.988479	0.011219	0.000279	0.000023	0.000012	4	4	239730	0	#JAKO/0!	0,00
A0A100I8P2	MCE family lipoprotein LprN	5,1	39,5	372	0	LIPO	0.000008	0.000000	1.000046	0.000000	0.000000	4	4	239669	0	#JAKO/0!	0,00
B2HHJ0	Tryptophan-rich sensory protein	11,6	17,1	159	3	SP	0.489339	0.507574	0.001390	0.000603	0.000410	3	2	239350	0	#JAKO/0!	0,00
A0A2Z5YI29	PE domain-containing protein	10,6	55,3	571	0	NO_SP	1.000017	0.000006	0.000000	0.000000	0.000000	2	4	239293,3333	0	#JAKO/0!	0,00

B2HQV9	Uncharacterized protein	7,6	**35,2**	323	0	NO_SP	1.000029	0.000000	0.000000	0.000000	0.000000	4	4	**239043,3333**	**0**	**#JAKO/0!**	**0,00**
A0A2Z5YL74	Fatty-acid-CoA racemase	5,7	**37,9**	359	0	NO_SP	1.000039	0.000000	0.000000	0.000000	0.000000	4	4	**238110**	**437**	**544,75**	**0,00**
A0A2Z5YCJ4	Carrier domain-containing protein	5,2	**106,3**	1005	0	NO_SP	1.000061	0.000001	0.000000	0.000000	0.000000	1	3	**237716,6667**	**3486**	**68,19**	**0,00**
B2HCT4	30S ribosomal protein S14 type Z	11,7	**6,9**	61	0	NO_SP	1.000049	0.000000	0.000000	0.000000	0.000000	3	1	**237463,3333**	**75914**	**3,13**	**0,32**
A0A117DVA0	Choloylglycine hydrolase	4,6	**33,5**	309	0	NO_SP	1.000062	0.000000	0.000000	0.000000	0.000000	5	3	**237056,6667**	**343**	**690,32**	**0,00**
A0A3E2MZW0	Phthiocerol/phenolphthiocerol synthesis polyketide synthase type I PpsB	4,7	**110,4**	1033	0	NO_SP	1.000060	0.000002	0.000000	0.000000	0.000000	2	1	**236023,3333**	**0**	**#JAKO/0!**	**0,00**
B2HCU8	L-fuculose-1-phosphate aldolase FucA	6,1	**22,9**	218	0	NO_SP	1.000039	0.000013	0.000000	0.000000	0.000000	2	5	**235660**	**0**	**#JAKO/0!**	**0,00**
A0A3E2MX56	tRNA dimethylallyltransferase	6,6	**34,0**	314	0	NO_SP	1.000045	0.000000	0.000000	0.000000	0.000000	4	4	**235596,6667**	**3370**	**69,91**	**0,01**
A0A3E2MR91	Putative ketoacyl reductase	11,6	**25,3**	241	0	NO_SP	1.000032	0.000003	0.000000	0.000000	0.000000	4	4	**234527,3333**	**12553**	**18,68**	**0,05**
A0A2Z5YNU3	5-oxoprolinase subunit A	4,9	**26,4**	252	0	NO_SP	1.000054	0.000000	0.000000	0.000000	0.000000	2	2	**234226,6667**	**4711**	**49,72**	**0,02**
A0A3E2MT49	Oxygen sensor histidine kinase NreB	7,3	**49,7**	464	6	NO_SP	0.996382	0.003633	0.000000	0.000000	0.000000	4	4	**233876,6667**	**0**	**#JAKO/0!**	**0,00**
B2HQG8	MCE-family protein, Mce3C_1	5,7	**46,4**	438	1	NO_SP	0.988632	0.009226	0.000282	0.000113	0.000047	4	4	**233800**	**1992**	**117,36**	**0,00**
B2HGT4	Non-specific serine/threonine protein kinase	5,2	**46,3**	431	2	NO_SP	1.000024	0.000010	0.000000	0.000000	0.000000	4	5	**233439,3333**	**0**	**#JAKO/0!**	**0,00**
A0A2Z5Y922	Uncharacterized protein	5,1	**23,4**	212	0	NO_SP	1.000042	0.000003	0.000000	0.000000	0.000000	1	4	**233410**	**0**	**#JAKO/0!**	**0,00**
A0A2Z5YEK3	Uncharacterized protein	4,5	**14,7**	130	0	NO_SP	1.000073	0.000000	0.000000	0.000000	0.000000	1	4	**233320**	**0**	**#JAKO/0!**	**0,00**
B2HEK9	Conserved hypothetical membrane protein	7,6	**19,7**	172	2	NO_SP	1.000058	0.000003	0.000000	0.000000	0.000000	4	1	**232943,3333**	**0**	**#JAKO/0!**	**0,00**
A0A100I5G6	SseC protein	4,7	**10,4**	100	0	NO_SP	1.000061	0.000002	0.000000	0.000000	0.000000	3	3	**232860**	**0**	**#JAKO/0!**	**0,00**
A0A2Z5YNF8	Peptide ABC transporter ATP-binding protein	9,7	**59,5**	551	0	NO_SP	1.000046	0.000001	0.000000	0.000000	0.000000	4	7	**232636,6667**	**0**	**#JAKO/0!**	**0,00**
A0A117DYY3	ESAT-6-like protein 7	5,6	**9,7**	87	0	NO_SP	1.000047	0.000000	0.000000	0.000000	0.000000	10	4	**232543,3333**	**330**	**704,55**	**0,00**
A0A2Z5Y9T2	Putative transcription antitermination regulator	5,3	**26,7**	240	0	NO_SP	1.000052	0.000010	0.000000	0.000000	0.000000	4	4	**232286,6667**	**0**	**#JAKO/0!**	**0,00**
B2HL16	Diaminopimelate epimerase	5,8	**30,0**	290	0	NO_SP	1.000029	0.000022	0.000001	0.000000	0.000000	4	5	**231524,3333**	**0**	**#JAKO/0!**	**0,00**
A0A3E2N052	Arylsulfatase	4,8	**50,7**	465	0	NO_SP	0.999891	0.000153	0.000002	0.000000	0.000000	4	4	**230696,6667**	**0**	**#JAKO/0!**	**0,00**
A0A2Z5YIX0	Oxidoreductase	6,0	**26,5**	258	0	NO_SP	1.000074	0.000000	0.000000	0.000000	0.000000	3	6	**230553,3333**	**0**	**#JAKO/0!**	**0,00**
A0A2Z5YLM9	Uncharacterized protein	5,2	**40,0**	362	0	NO_SP	1.000091	0.000001	0.000000	0.000000	0.000000	1	7	**229896,6667**	**0**	**#JAKO/0!**	**0,00**
B2HHX6	Enoyl-CoA hydratase EchA13_1	6,1	**34,5**	311	0	NO_SP	0.997774	0.002204	0.000039	0.000006	0.000003	4	2	**229886,6667**	**495**	**464,82**	**0,00**
B2HR38	Chalcone synthase, Pks11_1	4,6	**37,5**	353	0	NO_SP	1.000041	0.000000	0.000000	0.000000	0.000000	4	3	**229313,6667**	**0**	**#JAKO/0!**	**0,00**
A0A3E2MQJ9	Shikimate kinase	10,0	**19,4**	184	0	NO_SP	1.000078	0.000000	0.000000	0.000000	0.000000	4	4	**228236,3333**	**0**	**#JAKO/0!**	**0,00**
A0A2Z5YF17	Uncharacterized protein	9,2	**16,7**	152	3	NO_SP	0.728832	0.256444	0.003622	0.000834	0.000658	4	2	**227926,6667**	**5905**	**38,60**	**0,03**
A0A2Z5YGC1	Bifunctional pyrazinamidase/nicotinamidase	4,7	**19,6**	186	0	NO_SP	0.996194	0.003500	0.000289	0.000015	0.000009	4	3	**227926,6667**	**0**	**#JAKO/0!**	**0,00**
A0A2Z5YG48	Alcohol dehydrogenase	8,8	**61,2**	561	0	NO_SP	0.999581	0.000404	0.000034	0.000001	0.000001	3	6	**226970,6667**	**0**	**#JAKO/0!**	**0,00**
A0A100IA00	Lipoprotein	4,7	**63,1**	606	0	NO_SP	1.000040	0.000000	0.000000	0.000000	0.000000	4	4	**226953,3333**	**0**	**#JAKO/0!**	**0,00**
A0A2Z5YEK6	Segregation and condensation protein B	4,1	**25,6**	235	0	NO_SP	1.000061	0.000000	0.000000	0.000000	0.000000	4	4	**226783,3333**	**0**	**#JAKO/0!**	**0,00**
A0A100I398	Pyridoxamine 5-phosphate oxidase	6,8	**19,4**	170	0	NO_SP	1.000054	0.000000	0.000000	0.000000	0.000000	3	2	**226553,3333**	**1388**	**163,28**	**0,00**
B2HD48	Short-chain type dehydrogenase	6,9	**30,2**	285	0	NO_SP	1.000044	0.000000	0.000000	0.000000	0.000000	3	5	**226246,6667**	**861**	**262,82**	**0,00**
A0A3E2MP99	H(+)/Cl(-) exchange transporter ClcA	8,4	**49,6**	481	10	NO_SP	1.000005	0.000021	0.000000	0.000000	0.000000	4	4	**226013,3333**	**0**	**#JAKO/0!**	**0,00**
B2HI12	Lipid carrier protein or keto acyl-CoA thiolase Ltp3	5,9	**41,4**	392	0	NO_SP	1.000062	0.000000	0.000000	0.000000	0.000000	4	5	**225803,3333**	**0**	**#JAKO/0!**	**0,00**
A0A2Z5YK55	NAD-dependent protein deacylase	4,8	**26,1**	238	0	NO_SP	1.000039	0.000000	0.000000	0.000000	0.000000	4	3	**224936,6667**	**2258**	**99,61**	**0,01**
B2HKY5	Thymidylate synthase	5,8	**30,0**	266	0	NO_SP	1.000055	0.000000	0.000000	0.000000	0.000000	4	4	**224876,6667**	**0**	**#JAKO/0!**	**0,00**
B2HID7	Transferase	6,5	**44,5**	414	0	NO_SP	1.000058	0.000000	0.000000	0.000000	0.000000	4	3	**224856,6667**	**0**	**#JAKO/0!**	**0,00**
A0A117DZB6	LipoLpqN family protein	5,6	**18,2**	169	0	NO_SP	1.000038	0.000000	0.000000	0.000000	0.000000	4	2	**224560**	**0**	**#JAKO/0!**	**0,00**
A0A2Z5Y7S3	Coenzyme A pyrophosphatase	5,0	**26,3**	239	0	NO_SP	1.000059	0.000000	0.000000	0.000000	0.000000	3	3	**224353,3333**	**0**	**#JAKO/0!**	**0,00**
B2HLJ7	APH domain-containing protein	5,2	**36,8**	342	0	NO_SP	1.000051	0.000000	0.000000	0.000000	0.000000	4	4	**224310**	**1239**	**181,03**	**0,00**
A0A2Z5YGB4	RNA polymerase-binding protein RbpA	5,8	**12,9**	111	0	NO_SP	1.000056	0.000001	0.000000	0.000000	0.000000	1	5	**224043,3333**	**5222**	**42,90**	**0,02**
B2HHI3	Conserved hypothetical membrane protein	4,8	**24,1**	224	0	SP	0.000258	0.999116	0.000160	0.000165	0.000144	4	2	**223766,6667**	**0**	**#JAKO/0!**	**0,00**
A0A2Z5YNM5	Membrane protein	4,4	**32,6**	310	0	NO_SP	0.818624	0.179784	0.000660	0.000389	0.000193	4	2	**223309**	**0**	**#JAKO/0!**	**0,00**
A0A2Z5YFG9	PPOX class F420-dependent enzyme	6,2	**16,1**	147	0	NO_SP	1.000013	0.000026	0.000003	0.000000	0.000000	3	2	**223160**	**0**	**#JAKO/0!**	**0,00**
A0A2Z5Y939	Helix-turn-helix transcriptional regulator	6,2	**84,9**	817	0	NO_SP	1.000057	0.000000	0.000000	0.000000	0.000000	4	7	**222025,3333**	**0**	**#JAKO/0!**	**0,00**
A0A2Z5YKF7	Uncharacterized protein	10,6	**18,9**	177	1	NO_SP	0.992293	0.002168	0.000746	0.000024	0.000011	3	5	**221363,3333**	**0**	**#JAKO/0!**	**0,00**
A0A3E2MT47	DNA-binding transcriptional repressor MarR	7,7	**20,7**	183	0	NO_SP	1.000052	0.000000	0.000000	0.000000	0.000000	4	3	**221090**	**0**	**#JAKO/0!**	**0,00**
B2HI36	Cytochrome P450 125A7 Cyp125A7	4,6	**46,7**	417	0	NO_SP	1.000065	0.000000	0.000000	0.000000	0.000000	4	4	**221080**	**0**	**#JAKO/0!**	**0,00**
A0A2Z5YM90	Uncharacterized protein	6,2	**43,7**	404	0	NO_SP	1.000062	0.000001	0.000000	0.000000	0.000000	4	6	**220746,6667**	**0**	**#JAKO/0!**	**0,00**
B2HJT2	Nickel/iron-hydrogenase I large subunit, HyaB	6,6	**67,6**	599	0	NO_SP	1.000058	0.000000	0.000000	0.000000	0.000000	4	7	**220614,3333**	**0**	**#JAKO/0!**	**0,00**
A0A2Z5YHZ6	NAD(P)H nitroreductase	5,0	**36,3**	336	0	NO_SP	1.000068	0.000000	0.000000	0.000000	0.000000	1	1	**220356,6667**	**0**	**#JAKO/0!**	**0,00**
A0A2Z5YA10	RecBCD enzyme subunit RecD	7,5	**63,0**	587	0	NO_SP	1.000015	0.000020	0.000002	0.000000	0.000000	4	6	**220026,6667**	**321**	**685,75**	**0,00**
A0A2Z5YCD8	8-amino-7-oxononanoate synthase	5,3	**45,4**	416	0	NO_SP	1.000056	0.000000	0.000000	0.000000	0.000000	4	4	**220010**	**3381**	**65,06**	**0,02**
A0A117DVC3	Short-chain dehydrogenase	5,0	**26,8**	263	0	NO_SP	1.000043	0.000001	0.000000	0.000000	0.000000	3	5	**219308,3333**	**0**	**#JAKO/0!**	**0,00**
A0A2Z5YA56	HTH tetR-type domain-containing protein	11,6	**15,5**	145	0	NO_SP	1.000039	0.000000	0.000000	0.000000	0.000000	4	4	**219183,3333**	**0**	**#JAKO/0!**	**0,00**
A0A2Z5YD06	Uncharacterized protein	5,7	**15,5**	147	0	NO_SP	0.998458	0.001564	0.000011	0.000002	0.000001	4	3	**217770**	**0**	**#JAKO/0!**	**0,00**
B2HRB9	DNA repair exonuclease SbcD	4,6	**40,7**	383	0	NO_SP	1.000059	0.000004	0.000000	0.000000	0.000000	4	6	**217316,6667**	**0**	**#JAKO/0!**	**0,00**
A0A3E2MNT9	Uncharacterized protein	4,4	**47,9**	441	0	NO_SP	0.955024	0.044410	0.000347	0.000071	0.000045	4	2	**216364,3333**	**0**	**#JAKO/0!**	**0,00**
A0A2Z5YM95	Acyl-CoA dehydrogenase	4,9	**39,9**	386	0	NO_SP	1.000031	0.000001	0.000000	0.000000	0.000000	4	4	**216180**	**0**	**#JAKO/0!**	**0,00**
B2HSF6	RecBCD enzyme subunit RecB	5,5	**121,1**	1110	0	NO_SP	1.000066	0.000000	0.000000	0.000000	0.000000	4	5	**215456,6667**	**4119**	**52,31**	**0,00**
B2HND6	Oxidoreductase	6,8	**43,9**	406	0	NO_SP	0.999477	0.000518	0.000050	0.000001	0.000000	4	5	**215243,3333**	**0**	**#JAKO/0!**	**0,00**
A0A2Z5Y8E4	HTH tetR-type domain-containing protein	6,9	**23,0**	212	0	NO_SP	1.000073	0.000001	0.000000	0.000000	0.000000	4	3	**215233,3333**	**0**	**#JAKO/0!**	**0,00**
A0A100IDV3	Uncharacterized protein	4,6	**11,8**	110	0	NO_SP	1.000040	0.000001	0.000000	0.000000	0.000000	3	1	**214899,3333**	**0**	**#JAKO/0!**	**0,00**
B2HF80	Oxidoreductase	5,8	**55,2**	524	0	NO_SP	1.000074	0.000000	0.000000	0.000000	0.000000	4	3	**214580**	**0**	**#JAKO/0!**	**0,00**
A0A2Z5YLQ6	Proline iminopeptidase	4,7	**31,9**	288	0	NO_SP	1.000050	0.000000	0.000000	0.000000	0.000000	4	4	**214573,3333**	**0**	**#JAKO/0!**	**0,00**
A0A2Z5YM30	AAA_31 domain-containing protein	4,9	**25,3**	233	0	NO_SP	1.000019	0.000001	0.000000	0.000000	0.000000	1	4	**214496,6667**	**1848**	**116,08**	**0,01**
B2HMC2	Transcriptional regulator	6,7	**15,1**	142	0	NO_SP	1.000047	0.000001	0.000000	0.000000	0.000000	4	3	**213616,6667**	**3623**	**58,96**	**0,02**
B2HI27	2-keto-4-pentenoate hydratase	4,3	**28,0**	261	0	NO_SP	1.000053	0.000000	0.000000	0.000000	0.000000	3	3	**212283,3333**	**0**	**#JAKO/0!**	**0,00**
B2HGV3	Uncharacterized protein	7,3	**14,4**	129	0	NO_SP	1.000031	0.000022	0.000000	0.000000	0.000000	4	1	**211726,6667**	**8700**	**24,34**	**0,04**
A0A2Z5YND4	Oxidoreductase	5,9	**33,9**	330	0	NO_SP	1.000050	0.000000	0.000000	0.000000	0.000000	4	4	**211403,3333**	**0**	**#JAKO/0!**	**0,00**
B2HRI9	Conserved transmembrane transport protein, MmpL2	7,3	**108,9**	997	11	NO_SP	1.000006	0.000015	0.000000	0.000000	0.000000	4	3	**211073,3333**	**0**	**#JAKO/0!**	**0,00**
A0A2Z5YKA1	L-glutamate gamma-semialdehyde dehydrogenase	5,9	**58,6**	543	0	NO_SP	1.000033	0.000000	0.000000	0.000000	0.000000	3	9	**211070,6667**	**0**	**#JAKO/0!**	**0,00**
B2HKD9	Recombination protein RecR	4,8	**22,1**	203	0	NO_SP	1.000071	0.000000	0.000000	0.000000	0.000000	3	4	**210983,3333**	**0**	**#JAKO/0!**	**0,00**
B2HHY6	MCE-family protein Mce4F	4,6	**59,0**	560	1	NO_SP	0.984125	0.014621	0.000192	0.000111	0.000057	4	3	**210860**	**2824**	**74,68**	**0,00**
A0A2Z5YF36	Thiol reductant ABC exporter subunit CydD	6,1	**50,3**	480	3	NO_SP	0.999025	0.000913	0.000034	0.000003	0.000001	4	5	**210156,6667**	**0**	**#JAKO/0!**	**0,00**

A0A2Z5Y856	Peptide synthase	5,6	**149,6**	1416	0	NO_SP	1.000013	0.000016	0.000000	0.000000	0.000000	4	9	**210045**	**0**	**#JAKO/0!**	**0,00**
A0A2Z5Y8D4	TetR family transcriptional regulator	7,4	**22,0**	201	0	NO_SP	1.000059	0.000000	0.000000	0.000000	0.000000	4	4	**209846,3333**	**236**	**887,99**	**0,00**
A0A100I7K4	Membrane protein	10,3	**46,3**	419	10	NO_SP	0.998192	0.000558	0.001060	0.000004	0.000002	3	1	**209396,6667**	**4337**	**48,28**	**0,02**
B2HD07	Ribosomal-protein-alanine acetyltransferase, RimI	5,3	**17,4**	158	0	NO_SP	1.000016	0.000016	0.000000	0.000000	0.000000	3	4	**209134**	**1658**	**126,16**	**0,00**
A0A2Z5Y9R6	Thiamine biosynthesis oxidoreductase ThiO	6,3	**35,6**	334	0	NO_SP	0.999088	0.000902	0.000023	0.000001	0.000001	4	5	**208790**	**0**	**#JAKO/0!**	**0,00**
B2HF64	Precorrin-8x methylmutase, CobH	5,3	**21,5**	208	0	NO_SP	1.000056	0.000000	0.000000	0.000000	0.000000	4	4	**208766,6667**	**0**	**#JAKO/0!**	**0,00**
B2HP58	Transcriptional regulatory protein	6,7	**17,3**	160	0	NO_SP	1.000044	0.000001	0.000000	0.000000	0.000000	2	1	**208377,6667**	**0**	**#JAKO/0!**	**0,00**
B2HGL4	Glutaminase	5,0	**33,7**	320	0	NO_SP	1.000071	0.000000	0.000000	0.000000	0.000000	4	4	**208093**	**1316**	**158,15**	**0,00**
B2HEV1	Conserved protein	4,4	**8,5**	81	0	NO_SP	1.000063	0.000000	0.000000	0.000000	0.000000	3	3	**207990**	**17471**	**11,90**	**0,08**
B2HKZ0	Flavin-dependent thymidylate synthase	6,5	**27,6**	250	0	NO_SP	1.000059	0.000000	0.000000	0.000000	0.000000	4	3	**207683,3333**	**230**	**902,25**	**0,00**
A0A2Z5YE11	Lipoprotein LprI	4,4	**22,9**	211	0	LIPO	0.000000	0.000001	1.000060	0.000000	0.000000	4	4	**207295**	**7007**	**29,59**	**0,03**
A0A2Z5YGL9	Carboxylate--amine ligase	4,3	**32,2**	292	0	NO_SP	1.000043	0.000000	0.000000	0.000000	0.000000	3	3	**207150**	**2260**	**91,66**	**0,00**
A0A2Z5YN70	Multidrug efflux ATP-binding/permease protein	7,1	**129,5**	1201	9	NO_SP	1.000053	0.000002	0.000000	0.000000	0.000000	4	7	**206876,6667**	**0**	**#JAKO/0!**	**0,00**
A0A2Z5YCB3	Methionine synthase	4,6	**34,2**	337	0	NO_SP	0.991337	0.008570	0.000081	0.000019	0.000010	3	4	**206863,3333**	**288**	**718,98**	**0,00**
A0A3E2MU22	TfuA-like protein	4,9	**53,9**	500	0	NO_SP	1.000058	0.000001	0.000000	0.000000	0.000000	4	4	**206822,6667**	**0**	**#JAKO/0!**	**0,00**
A0A2Z5Y979	Alpha/beta hydrolase family protein	7,0	**26,9**	253	0	NO_SP	1.000056	0.000001	0.000000	0.000000	0.000000	3	5	**206361,3333**	**0**	**#JAKO/0!**	**0,00**
A0A124BU37	Guanylate kinase	6,9	**20,1**	186	0	NO_SP	1.000067	0.000000	0.000000	0.000000	0.000000	3	3	**205873,3333**	**0**	**#JAKO/0!**	**0,00**
A0A2Z5YMD9	Oxidoreductase	5,8	**94,6**	900	0	NO_SP	1.000057	0.000001	0.000000	0.000000	0.000000	5	6	**205730**	**0**	**#JAKO/0!**	**0,00**
A0A2Z5YIX8	Cytochrome P450	5,3	**45,9**	419	0	NO_SP	1.000038	0.000002	0.000000	0.000000	0.000000	4	4	**205506,6667**	**785**	**261,68**	**0,00**
A0A3E2MN26	DNA replication and repair protein RecF	6,1	**38,7**	359	0	NO_SP	1.000051	0.000006	0.000000	0.000000	0.000000	4	6	**205226,6667**	**0**	**#JAKO/0!**	**0,00**
B2HJM9	L-lactate dehydrogenase (Cytochrome) LldD1_1	4,6	**41,3**	386	0	NO_SP	1.000048	0.000000	0.000000	0.000000	0.000000	4	3	**205078,6667**	**0**	**#JAKO/0!**	**0,00**
B2HN78	Zn-dependent glyoxylase	6,8	**24,6**	224	0	NO_SP	1.000059	0.000000	0.000000	0.000000	0.000000	4	5	**205006,6667**	**0**	**#JAKO/0!**	**0,00**
B2HNT4	Ribosomal RNA small subunit methyltransferase G	9,8	**24,8**	228	0	NO_SP	1.000044	0.000002	0.000000	0.000000	0.000000	4	3	**204380**	**1045**	**195,66**	**0,00**
A0A2Z5YN25	Uncharacterized protein	4,9	**47,9**	435	0	NO_SP	1.000043	0.000001	0.000000	0.000000	0.000000	1	1	**203886,6667**	**2335**	**87,31**	**0,00**
A0A2Z5YD33	Cob(I)alamin adenosyltransferase	9,4	**22,4**	204	0	NO_SP	1.000081	0.000000	0.000000	0.000000	0.000000	3	5	**203766,6667**	**223**	**915,38**	**0,00**
B2HIG2	Conserved hypothetical membrane protein	5,2	**29,5**	282	5	NO_SP	1.000018	0.000019	0.000000	0.000000	0.000000	4	2	**203589,6667**	**0**	**#JAKO/0!**	**0,00**
A0A2Z5YKF4	Uncharacterized protein	4,9	**18,4**	172	0	NO_SP	0.999408	0.000625	0.000002	0.000001	0.000001	4	3	**203271,3333**	**0**	**#JAKO/0!**	**0,00**
A0A3E2MYB9	Polyketide cyclase / dehydrase and lipid transport	10,0	**17,1**	157	0	NO_SP	1.000054	0.000002	0.000000	0.000000	0.000000	4	2	**202706,6667**	**0**	**#JAKO/0!**	**0,00**
A0A2Z5YLV6	Uncharacterized protein	4,3	**27,7**	257	0	NO_SP	1.000036	0.000015	0.000001	0.000000	0.000000	3	5	**201945,6667**	**0**	**#JAKO/0!**	**0,00**
B2HP18	Pyrrolidone-carboxylate peptidase	4,8	**23,2**	222	0	NO_SP	1.000046	0.000000	0.000000	0.000000	0.000000	4	3	**201753,3333**	**0**	**#JAKO/0!**	**0,00**
A0A100HZK3	Membrane protein	9,0	**54,6**	510	8	NO_SP	0.997048	0.002960	0.000000	0.000000	0.000000	3	2	**201665,6667**	**0**	**#JAKO/0!**	**0,00**
A0A2Z5YHN2	Acyl-CoA dehydrogenase	5,8	**41,9**	392	0	NO_SP	0.999773	0.000260	0.000001	0.000000	0.000000	4	5	**199640**	**0**	**#JAKO/0!**	**0,00**
A0A3E2MPR3	Glucose-1-phosphate cytidylyltransferase	6,2	**29,5**	258	0	NO_SP	1.000090	0.000000	0.000000	0.000000	0.000000	1	1	**199470**	**5377**	**37,10**	**0,03**
B2HQJ5	Adenylate cyclase	8,8	**58,4**	539	6	NO_SP	0.999670	0.000325	0.000000	0.000000	0.000000	6	4	**198300**	**0**	**#JAKO/0!**	**0,00**
A0A2Z5YN18	Site-specific DNA-methyltransferase (adenine-specific)	5,0	**58,0**	519	0	NO_SP	1.000060	0.000000	0.000000	0.000000	0.000000	1	5	**198090**	**0**	**#JAKO/0!**	**0,00**
A0A2Z5YGJ0	Sec-independent protein translocase protein TatA	8,8	**9,5**	88	1	NO_SP	0.999328	0.000688	0.000010	0.000002	0.000001	4	2	**198055,6667**	**8957**	**22,11**	**0,00**
B2HD05	t(6)A37 threonylcarbamoyladenosine biosynthesis protein TsaE	4,8	**16,6**	156	0	NO_SP	1.000031	0.000004	0.000000	0.000000	0.000000	4	1	**197773,3333**	**72728**	**2,72**	**0,37**
A0A2Z5YKQ2	DUF5642 domain-containing protein	5,9	**20,3**	193	0	NO_SP	1.000025	0.000001	0.000000	0.000000	0.000000	4	3	**197753,3333**	**0**	**#JAKO/0!**	**0,00**
A0A2Z5YNZ4	Uncharacterized protein	7,0	**19,8**	189	3	NO_SP	0.999019	0.000976	0.000004	0.000004	0.000001	1	4	**197373,3333**	**0**	**#JAKO/0!**	**0,00**
B2HD04	Conserved hypothetical hydrolase	9,9	**40,8**	373	1	TAT	0.005311	0.011652	0.002106	0.784428	0.196328	3	4	**196933,3333**	**0**	**#JAKO/0!**	**0,00**
A0A3E2MNE7	Leucine carboxyl methyltransferase	10,0	**29,8**	266	0	NO_SP	1.000080	0.000001	0.000000	0.000000	0.000000	4	2	**196896,6667**	**9227**	**21,34**	**0,05**
A0A2Z5YH13	TP_methylase domain-containing protein	6,9	**51,4**	490	0	NO_SP	1.000042	0.000003	0.000000	0.000000	0.000000	4	4	**196880**	**666**	**295,72**	**0,00**
A0A2Z5YCE2	Putative 8-oxo-dGTP diphosphatase 1	10,3	**31,9**	290	0	NO_SP	1.000064	0.000001	0.000000	0.000000	0.000000	4	6	**196662,6667**	**0**	**#JAKO/0!**	**0,00**
A0A3E2N0X0	Putative metallo-hydrolase YflN	8,0	**27,0**	250	0	NO_SP	1.000037	0.000003	0.000000	0.000000	0.000000	4	4	**196459**	**0**	**#JAKO/0!**	**0,00**
B2HEB4	Acetyl-/propionyl-CoA carboxylase (Beta subunit) AccD2	5,4	**56,3**	531	0	NO_SP	1.000036	0.000001	0.000000	0.000000	0.000000	4	6	**196422,6667**	**862**	**227,84**	**0,00**
A0A2Z5YHK7	Monooxygenase	10,0	**43,6**	392	0	NO_SP	1.000062	0.000000	0.000000	0.000000	0.000000	4	3	**196286,6667**	**3488**	**56,28**	**0,02**
B2HGQ1	Conserved transmembrane transport protein MmpL4_5	6,6	**103,4**	952	12	NO_SP	0.853580	0.142479	0.000810	0.000734	0.000427	5	4	**195893,3333**	**0**	**#JAKO/0!**	**0,00**
A0A2Z5YC49	Uncharacterized protein	4,9	**38,9**	350	0	NO_SP	1.000077	0.000000	0.000000	0.000000	0.000000	3	2	**195846,6667**	**1193**	**164,20**	**0,00**
A0A3E2MP31	Putative hydrogen peroxide-inducible genes activator	5,6	**33,7**	310	0	NO_SP	1.000062	0.000001	0.000000	0.000000	0.000000	4	6	**195650**	**0**	**#JAKO/0!**	**0,00**
A0A100I0V9	Polyketide synthase	5,0	**220,6**	2074	0	NO_SP	0.999272	0.000313	0.000426	0.000002	0.000001	1	5	**195606,6667**	**0**	**#JAKO/0!**	**0,00**
B2HIB2	Conserved hypothetical membrane protein	6,7	**30,5**	289	1	NO_SP	1.000056	0.000002	0.000000	0.000000	0.000000	4	2	**195566,6667**	**0**	**#JAKO/0!**	**0,00**
A0A2Z5Y9N7	Haloacid dehalogenase	7,2	**158,5**	1527	0	NO_SP	0.926658	0.072751	0.000197	0.000175	0.000085	4	7	**195513,3333**	**0**	**#JAKO/0!**	**0,00**
A0A2Z5YDI6	DUF1990 domain-containing protein	8,5	**18,0**	167	0	NO_SP	1.000037	0.000001	0.000000	0.000000	0.000000	3	2	**195363,6667**	**0**	**#JAKO/0!**	**0,00**
B2HN63	Holliday junction ATP-dependent DNA helicase RuvB	5,3	**36,7**	345	0	NO_SP	1.000029	0.000000	0.000000	0.000000	0.000000	3	3	**194846,6667**	**0**	**#JAKO/0!**	**0,00**
B2HDY4	Transcriptional regulatory protein (Probably AsnC-family)	5,6	**16,4**	150	0	NO_SP	1.000028	0.000009	0.000001	0.000000	0.000000	3	1	**194053,3333**	**3533**	**54,93**	**0,02**
A0A3E2N294	Mce related protein	4,6	**48,8**	467	1	NO_SP	0.953078	0.044922	0.001316	0.000138	0.000099	4	2	**193779,3333**	**0**	**#JAKO/0!**	**0,00**
A0A100I229	Uncharacterized protein	9,6	**44,4**	404	0	NO_SP	1.000051	0.000000	0.000000	0.000000	0.000000	4	3	**193238,3333**	**266193**	**0,73**	**0,00**
B2HDU6	Cytidine deaminase Cdd	5,5	**13,8**	132	0	NO_SP	1.000057	0.000002	0.000000	0.000000	0.000000	3	2	**193006,6667**	**0**	**#JAKO/0!**	**0,00**
B2HS59	Uncharacterized protein	4,3	**13,0**	123	1	NO_SP	0.999858	0.000190	0.000001	0.000001	0.000000	4	1	**192840**	**6705**	**28,76**	**0,03**
A0A3E2N3A8	Cobyric acid synthase	5,6	**51,6**	491	0	NO_SP	1.000059	0.000001	0.000000	0.000000	0.000000	4	5	**192733,3333**	**0**	**#JAKO/0!**	**0,00**
B2HH27	GCV_T domain-containing protein	5,7	**38,6**	363	0	NO_SP	0.998759	0.001267	0.000008	0.000002	0.000001	4	4	**192340**	**0**	**#JAKO/0!**	**0,00**
B2HSX2	Glycine cleavage system H protein	3,7	**14,2**	134	0	NO_SP	1.000040	0.000025	0.000001	0.000000	0.000000	4	1	**192170**	**13643**	**14,09**	**0,07**
A0A2Z5YEP6	Cytidine monophosphate kinase	4,7	**19,9**	188	0	NO_SP	0.992592	0.007338	0.000061	0.000017	0.000008	4	4	**192130**	**11921**	**16,12**	**0,00**
B2HCV0	D-xylulose-kinase (Xylulokinase), XylB	5,3	**47,4**	452	0	NO_SP	1.000057	0.000005	0.000001	0.000000	0.000000	4	5	**192034,3333**	**0**	**#JAKO/0!**	**0,00**
B2HEF0	Putative S-adenosyl-L-methionine-dependent methyltransferase MMAR_4570	4,5	**32,5**	307	0	NO_SP	0.999893	0.000144	0.000001	0.000000	0.000000	4	3	**191730**	**0**	**#JAKO/0!**	**0,00**
B2HHN8	SURF1-like protein	9,7	**30,2**	274	2	NO_SP	0.999978	0.000020	0.000004	0.000000	0.000000	4	4	**191413,3333**	**642**	**297,94**	**0,00**
A0A2Z5Y9L9	Uncharacterized protein	4,4	**14,4**	134	0	NO_SP	1.000050	0.000000	0.000000	0.000000	0.000000	4	3	**191166,6667**	**3216**	**59,45**	**0,02**
A0A2Z5YAT5	S-adenosyl-L-methionine-dependent methyltransferase	4,4	**32,9**	304	0	NO_SP	1.000017	0.000024	0.000000	0.000000	0.000000	3	3	**191040**	**0**	**#JAKO/0!**	**0,00**
A0A2Z5YP90	Uncharacterized protein	5,9	**16,9**	154	0	NO_SP	1.000074	0.000000	0.000000	0.000000	0.000000	4	3	**190530**	**0**	**#JAKO/0!**	**0,00**
B2HIK5	Methyltransferase	4,7	**16,3**	157	0	NO_SP	0.999948	0.000082	0.000003	0.000000	0.000000	4	3	**190046,6667**	**3803**	**49,97**	**0,00**
B2HJ80	Uncharacterized protein	6,9	**20,3**	192	0	NO_SP	1.000058	0.000003	0.000000	0.000000	0.000000	3	2	**189880**	**0**	**#JAKO/0!**	**0,00**
A0A2Z5YMX2	Alcohol dehydrogenase	4,6	**35,4**	337	0	NO_SP	1.000044	0.000000	0.000000	0.000000	0.000000	4	3	**189483,3333**	**4910**	**38,59**	**0,03**
A0A2Z5YKX7	Uncharacterized protein	7,7	**57,2**	528	6	NO_SP	0.999991	0.000030	0.000000	0.000000	0.000000	4	2	**189423,3333**	**10388**	**18,23**	**0,00**
B2HNY6	Uncharacterized protein	4,3	**14,0**	128	0	NO_SP	1.000062	0.000000	0.000000	0.000000	0.000000	3	2	**189422**	**0**	**#JAKO/0!**	**0,00**
A0A100IAR3	Transmembrane transport protein	6,0	**101,9**	936	11	NO_SP	0.562365	0.435376	0.001022	0.000334	0.000284	4	3	**189371**	**0**	**#JAKO/0!**	**0,00**
B2HJ51	7,8-dihydroneopterin aldolase	5,6	**14,5**	133	0	NO_SP	1.000050	0.000001	0.000000	0.000000	0.000000	3	2	**188774,6667**	**0**	**#JAKO/0!**	**0,00**
B2HHL3	Octanoyltransferase	6,0	**25,1**	241	0	NO_SP	0.997261	0.002768	0.000016	0.000004	0.000002	4	3	**187809,3333**	**0**	**#JAKO/0!**	**0,00**

B2HHI0	Enoyl-CoA hydratase EchA17	4,5	27,0	257	0	NO_SP	1.000071	0.000000	0.000000	0.000000	0.000000	4	3	187693,3333	0	#JAKO/0!	0,00
B2HME5	Probable nicotinate-nucleotide adenylyltransferase	5,6	23,7	215	0	NO_SP	1.000069	0.000000	0.000000	0.000000	0.000000	3	4	186818,6667	0	#JAKO/0!	0,00
A0A3E2MTY1	Nuclear transport factor 2 (NTF2) domain protein	8,1	29,2	265	0	NO_SP	1.000049	0.000000	0.000000	0.000000	0.000000	4	4	186629,3333	0	#JAKO/0!	0,00
B2HMW7	Conserved membrane protein	4,4	22,7	215	1	NO_SP	0.999979	0.000054	0.000000	0.000000	0.000000	3	2	186150	0	#JAKO/0!	0,00
B2HJ34	Polyprenol-monophosphomannose synthase Ppm1_2	9,5	35,2	320	0	NO_SP	1.000050	0.000000	0.000000	0.000000	0.000000	2	5	186133,3333	0	#JAKO/0!	0,00
A0A3E2MTZ5	Uncharacterized protein	4,6	27,8	259	0	NO_SP	1.000006	0.000019	0.000000	0.000000	0.000000	3	1	186080	0	#JAKO/0!	0,00
A0A2Z5YBS1	Alpha-acetolactate decarboxylase	4,7	28,7	258	0	NO_SP	1.000047	0.000000	0.000000	0.000000	0.000000	3	4	185666,6667	0	#JAKO/0!	0,00
B2HS72	Transcriptional regulatory protein	5,9	46,6	421	0	NO_SP	1.000068	0.000000	0.000000	0.000000	0.000000	8	2	185003,3333	3615	51,18	0,02
A0A2Z5YDQ7	Uncharacterized protein	10,8	18,8	173	1	NO_SP	1.000042	0.000002	0.000000	0.000000	0.000000	4	2	184630	1294	142,74	0,00
A0A100I2R1	2-amino-3-ketobutyrate coenzyme A ligase	4,9	42,3	383	0	NO_SP	1.000037	0.000000	0.000000	0.000000	0.000000	3	3	184616,6667	0	#JAKO/0!	0,00
B2HRZ0	Sugar transporter	7,1	54,3	503	12	NO_SP	0.999984	0.000036	0.000000	0.000000	0.000000	4	2	184246,6667	2018	91,29	0,00
A0A3E2N1P0	HIT-like protein	7,4	15,6	142	0	NO_SP	1.000055	0.000001	0.000000	0.000000	0.000000	4	2	184049,6667	694	265,12	0,00
A0A2Z5Y9E4	O-succinylhomoserine sulfhydrylase	4,8	44,0	412	1	NO_SP	1.000090	0.000000	0.000000	0.000000	0.000000	4	7	183971,3333	151143	1,22	0,00
A0A2Z5YJS2	Uncharacterized protein	4,9	28,7	276	0	NO_SP	1.000075	0.000000	0.000000	0.000000	0.000000	1	2	183652	0	#JAKO/0!	0,00
B2HM69	Conserved protein	6,4	12,1	114	0	NO_SP	1.000048	0.000001	0.000000	0.000000	0.000000	3	4	183510	0	#JAKO/0!	0,00
A0A2Z5YHQ6	Transcriptional regulatory protein EmbR	6,2	41,0	379	0	NO_SP	1.000048	0.000002	0.000000	0.000000	0.000000	4	5	183401,3333	0	#JAKO/0!	0,00
A0A2Z5YEK9	Uncharacterized protein	9,7	30,9	284	0	NO_SP	1.000041	0.000006	0.000000	0.000000	0.000000	3	4	183106	0	#JAKO/0!	0,00
B2HLE8	Transcriptional regulatory protein (Probably AraC/XylS-family)	9,3	37,0	339	0	NO_SP	1.000034	0.000001	0.000000	0.000000	0.000000	4	5	182817,3333	0	#JAKO/0!	0,00
B2HGT3	Conserved regulatory protein	6,2	14,6	134	0	NO_SP	1.000058	0.000000	0.000000	0.000000	0.000000	3	3	182665,3333	0	#JAKO/0!	0,00
A0A2Z5YC20	Putative oxidoreductase SadH	9,8	31,5	291	0	NO_SP	1.000062	0.000001	0.000000	0.000000	0.000000	3	4	182628,6667	2734	66,81	0,00
B2HEV7	Lipase LipV	4,6	28,2	261	0	NO_SP	1.000074	0.000001	0.000000	0.000000	0.000000	3	1	182610	0	#JAKO/0!	0,00
B2HMF2	Conserved hypothetical membrane protein	10,3	50,2	466	5	NO_SP	1.000055	0.000000	0.000000	0.000000	0.000000	4	4	182518	0	#JAKO/0!	0,00
B2HPU1	Carbon monoxyde dehydrogenase (Large chain), CoxL	4,9	85,6	796	0	NO_SP	1.000031	0.000001	0.000000	0.000000	0.000000	4	12	181932,6667	0	#JAKO/0!	0,00
A0A2Z5Y987	Limonene 1,2-monooxygenase	6,7	39,7	372	0	NO_SP	0.999950	0.000070	0.000000	0.000000	0.000000	3	3	181703,3333	0	#JAKO/0!	0,00
A0A2Z5YFK4	LLM class F420-dependent oxidoreductase	8,3	34,7	314	0	NO_SP	1.000021	0.000005	0.000000	0.000000	0.000000	4	5	181519	0	#JAKO/0!	0,00
A0A2Z5YLF0	Putative pterin-4-alpha-carbinolamine dehydratase	6,0	11,4	103	0	NO_SP	1.000030	0.000000	0.000000	0.000000	0.000000	3	2	181490	2079	87,28	0,00
A0A2Z5YLY0	DUF4357 domain-containing protein	6,5	32,3	299	0	NO_SP	1.000034	0.000000	0.000000	0.000000	0.000000	1	3	181403,3333	0	#JAKO/0!	0,00
B2HM73	4HBT domain-containing protein	8,5	22,7	207	1	NO_SP	1.000057	0.000003	0.000000	0.000000	0.000000	4	3	180640	2677	67,48	0,00
A0A2Z5YA16	Galactokinase	6,2	38,0	366	0	NO_SP	0.999980	0.000044	0.000003	0.000000	0.000000	3	2	180543,3333	0	#JAKO/0!	0,00
A0A2Z5YHA5	Uncharacterized protein	8,2	65,6	645	1	NO_SP	1.000033	0.000000	0.000000	0.000000	0.000000	4	5	179981,3333	0	#JAKO/0!	0,00
A0A100I2M9	Ferredoxin / Ferredoxin--NADP(+) reductase	7,5	57,9	546	0	NO_SP	1.000054	0.000002	0.000000	0.000000	0.000000	4	4	179032,6667	750	238,83	0,00
B2HIY8	PPE family protein	3,9	52,6	537	0	NO_SP	1.000067	0.000001	0.000000	0.000000	0.000000	4	1	178170	0	#JAKO/0!	0,00
A0A2Z5YK59	Non-specific serine/threonine protein kinase	6,3	52,5	493	2	NO_SP	0.794362	0.195837	0.008757	0.000250	0.000265	4	3	178062	0	#JAKO/0!	0,00
B2HQU7	Uncharacterized protein	4,6	19,0	174	0	NO_SP	1.000060	0.000000	0.000000	0.000000	0.000000	4	3	178023,3333	0	#JAKO/0!	0,00
A0A2Z5Y9L2	Deoxyribose-phosphate aldolase	5,2	22,1	226	0	NO_SP	0.999996	0.000061	0.000000	0.000000	0.000000	1	4	177890	0	#JAKO/0!	0,00
A0A124BWT1	NAD-dependent malic enzyme	4,8	58,6	543	0	NO_SP	1.000050	0.000000	0.000000	0.000000	0.000000	4	5	177740	961	185,01	0,00
B2HND8	Beta-lactamase	6,5	44,1	401	0	NO_SP	1.000053	0.000002	0.000000	0.000000	0.000000	4	3	177396,6667	28611	6,20	0,16
B2HMB0	Conserved hypothetical membrane protein	8,8	68,1	667	18	NO_SP	1.000008	0.000001	0.000000	0.000000	0.000000	5	2	177113,3333	0	#JAKO/0!	0,00
A0A2Z5YH39	Putative hydrolase	4,9	53,1	503	0	NO_SP	0.705675	0.293062	0.000432	0.000417	0.000181	4	5	176933,3333	78633	2,25	0,00
A0A2Z5YEJ0	Carrier domain-containing protein	4,8	153,0	1474	2	NO_SP	0.998740	0.001219	0.000036	0.000006	0.000002	1	2	176923,3333	0	#JAKO/0!	0,00
B2HJS4	Uncharacterized protein	4,5	25,2	240	0	NO_SP	0.998238	0.001667	0.000080	0.000010	0.000004	4	5	176124,6667	0	#JAKO/0!	0,00
B2HP89	Transcriptional regulatory protein (Probably GntR-family)	5,3	27,2	242	0	NO_SP	1.000050	0.000001	0.000000	0.000000	0.000000	3	5	175713,3333	194	905,57	0,00
B2HKM5	O-methyltransferase Omt_1	7,2	30,6	269	0	NO_SP	1.000065	0.000003	0.000000	0.000000	0.000000	4	4	175376,3333	1037	169,11	0,00
B2HK94	Sensor-component of a two-component regulator	4,4	80,3	742	0	NO_SP	1.000049	0.000000	0.000000	0.000000	0.000000	5	5	175370,3333	0	#JAKO/0!	0,00
A0A2Z5YB86	ATP-dependent helicase	6,3	164,4	1539	0	NO_SP	1.000042	0.000003	0.000000	0.000000	0.000000	4	8	175341,6667	0	#JAKO/0!	0,00
A0A3E2MQ68	Coproporphyrin III ferrochelatase	5,2	36,4	340	0	NO_SP	1.000048	0.000000	0.000000	0.000000	0.000000	4	2	175313,3333	2372	73,90	0,00
A0A2Z5YKM1	Thiolase_N domain-containing protein	9,9	12,0	117	0	NO_SP	1.000059	0.000000	0.000000	0.000000	0.000000	1	1	174731,6667	0	#JAKO/0!	0,00
A0A2Z5YMM9	Cobalt transporter	8,1	26,9	255	6	NO_SP	0.682171	0.305657	0.010007	0.001036	0.000539	2	1	174455,3333	0	#JAKO/0!	0,00
A0A117DV06	Superfamily I DNA and RNA helicase	6,1	117,3	1073	0	NO_SP	1.000060	0.000000	0.000000	0.000000	0.000000	4	7	174320	0	#JAKO/0!	0,00
A0A100I8M2	Acetoacetate decarboxylase family protein	9,6	24,6	225	0	NO_SP	0.998633	0.001385	0.000010	0.000003	0.000001	4	3	173763,3333	303	573,04	0,00
A0A100IF27	PE family protein	3,7	52,0	490	0	NO_SP	0.954723	0.044957	0.000125	0.000086	0.000044	3	1	173671,3333	0	#JAKO/0!	0,00
A0A2Z5YAW4	N-acetylglucosamine-6-phosphate deacetylase	5,7	39,6	388	0	NO_SP	0.999683	0.000324	0.000022	0.000000	0.000000	4	5	173219,3333	0	#JAKO/0!	0,00
A0A2Z5YJ74	CoA transferase	5,0	86,6	801	0	NO_SP	1.000067	0.000003	0.000000	0.000000	0.000000	4	5	172940	0	#JAKO/0!	0,00
A0A2Z5YMG7	Putative TrmH family tRNA/rRNA methyltransferase	6,3	29,7	279	0	NO_SP	1.000058	0.000001	0.000000	0.000000	0.000000	3	3	172513,3333	1289	133,87	0,00
A0A100ID05	Deazaflavin-dependent nitroreductase family protein	10,9	18,9	173	0	NO_SP	1.000017	0.000034	0.000000	0.000000	0.000000	3	2	172234,3333	0	#JAKO/0!	0,00
A0A2Z5YG68	Ferredoxin	4,3	7,4	68	0	NO_SP	1.000064	0.000000	0.000000	0.000000	0.000000	4	1	171820	0	#JAKO/0!	0,00
B2HIR1	Conserved hypothetical membrane protein	11,9	18,9	170	1	NO_SP	1.000012	0.000004	0.000000	0.000000	0.000000	4	2	171653,3333	1704	100,75	0,00
A0A3E2MU14	DoxX	10,6	27,2	262	0	NO_SP	0.970888	0.028891	0.000118	0.000050	0.000031	4	2	169756,6667	2750	61,74	0,00
B2HQE9	Acyl-CoA dehydrogenase FadE	5,3	41,1	387	0	NO_SP	1.000031	0.000000	0.000000	0.000000	0.000000	4	3	169406,6667	0	#JAKO/0!	0,00
A0A2Z5YAS7	Uncharacterized protein	10,0	14,0	128	0	NO_SP	0.999902	0.000124	0.000002	0.000000	0.000000	3	3	169307	0	#JAKO/0!	0,00
B2HM29	Conserved hypothetical membrane protein	5,1	53,9	523	1	LIPO	0.000000	0.000015	1.000033	0.000000	0.000000	2	1	168657,6667	0	#JAKO/0!	0,00
B2HMW5	Oxidoreductase	5,9	81,1	745	0	NO_SP	1.000076	0.000001	0.000000	0.000000	0.000000	5	3	168632,3333	0	#JAKO/0!	0,00
B2HCV2	Putative S-adenosyl-L-methionine-dependent methyltransferase MMAR_1068	4,5	33,2	302	0	NO_SP	0.999603	0.000415	0.000002	0.000001	0.000000	4	4	168223,3333	0	#JAKO/0!	0,00
A0A124BVY8	ABC transporter ATP-binding protein	8,3	64,9	612	6	NO_SP	1.000031	0.000030	0.000000	0.000000	0.000000	4	3	168006,6667	0	#JAKO/0!	0,00
A0A3E2MNB3	Uncharacterized protein	6,0	74,1	699	0	NO_SP	1.000048	0.000000	0.000000	0.000000	0.000000	4	4	167935,3333	0	#JAKO/0!	0,00
A0A2Z5YGY8	3-5 exoribonuclease	5,8	18,6	159	0	NO_SP	1.000041	0.000001	0.000000	0.000000	0.000000	4	5	167843,3333	0	#JAKO/0!	0,00
A0A2Z5YEF8	NLP/P60 family protein	10,3	27,4	259	1	SP	0.000245	0.999125	0.000146	0.000175	0.000142	3	2	167096,6667	0	#JAKO/0!	0,00
B2HET6	RNA polymerase sigma factor	4,5	28,4	255	0	NO_SP	1.000054	0.000003	0.000000	0.000000	0.000000	3	2	166900	0	#JAKO/0!	0,00
B2HPT9	Carbon monoxyde dehydrogenase (Medium chain), CoxM	4,9	31,5	296	0	NO_SP	1.000042	0.000000	0.000000	0.000000	0.000000	4	5	166889,3333	0	#JAKO/0!	0,00
A0A100I1F0	3-oxoacyl-ACP synthase	5,3	26,0	252	1	NO_SP	1.000044	0.000000	0.000000	0.000000	0.000000	3	2	166820	0	#JAKO/0!	0,00
B2HLB7	Endoribonuclease YbeY	4,0	19,7	178	0	NO_SP	1.000063	0.000000	0.000000	0.000000	0.000000	3	1	166576,6667	0	#JAKO/0!	0,00
A0A2Z5YCD0	DUF1330 domain-containing protein	4,3	11,0	96	0	NO_SP	1.000048	0.000001	0.000000	0.000000	0.000000	3	4	166456,3333	0	#JAKO/0!	0,00
B2HRM3	Conserved hypothetical membrane protein	12,0	11,0	106	2	NO_SP	1.000035	0.000001	0.000000	0.000000	0.000000	4	1	166356,6667	8817	18,87	0,05
B2HM89	Thioesterase TesA	5,3	27,1	252	0	NO_SP	1.000071	0.000000	0.000000	0.000000	0.000000	4	4	165665,6667	0	#JAKO/0!	0,00
B2HLV7	Conserved hypothetical regulatory protein	9,3	19,6	181	0	NO_SP	1.000057	0.000004	0.000000	0.000000	0.000000	3	5	165403,3333	0	#JAKO/0!	0,00
A0A2Z5YP41	Toxin Doc	4,2	13,4	125	0	NO_SP	0.999977	0.000066	0.000004	0.000000	0.000000	1	3	165190	1389	118,89	0,00
B2HI34	Acyl-CoA dehydrogenase FadE29	6,2	42,5	387	0	NO_SP	1.000056	0.000000	0.000000	0.000000	0.000000	4	4	165171	154	1073,14	0,00

B2HN50	Probable transcriptional regulatory protein MMAR_2098	4,3	**26,8**	251	0	NO_SP	1.000064	0.000000	0.000000	0.000000	0.000000	3	4	**165136,6667**	**65944**	**2,50**	**0,40**
A0A2Z5YGJ5	Uncharacterized protein	4,1	**24,5**	223	0	NO_SP	1.000040	0.000001	0.000000	0.000000	0.000000	4	3	**164046,6667**	**0**	**#JAKO/0!**	**0,00**
A2T2X9	Hydrolase	4,7	**41,8**	398	0	NO_SP	1.000057	0.000002	0.000000	0.000000	0.000000	4	2	**163730**	**0**	**#JAKO/0!**	**0,00**
A0A2Z5Y8T7	Transcriptional regulator	5,4	**23,3**	218	0	NO_SP	1.000043	0.000000	0.000000	0.000000	0.000000	4	5	**163317,6667**	**307**	**531,52**	**0,00**
A0A2Z5YE34	Uncharacterized protein	6,5	**36,8**	352	4	NO_SP	0.999337	0.000668	0.000003	0.000002	0.000001	4	4	**163273,6667**	**0**	**#JAKO/0!**	**0,00**
A0A2Z5YGZ3	Thioesterase	4,7	**22,6**	212	0	NO_SP	1.000027	0.000001	0.000000	0.000000	0.000000	3	4	**162970**	**0**	**#JAKO/0!**	**0,00**
A0A2Z5YEC1	Shikimate 5-dehydrogenase	6,2	**28,4**	277	0	NO_SP	0.998845	0.001164	0.000028	0.000003	0.000001	11	5	**162913,6667**	**6522**	**24,98**	**0,04**
A0A2Z5YFE1	PPE family protein	4,3	**39,2**	393	0	NO_SP	1.000056	0.000000	0.000000	0.000000	0.000000	4	2	**162826**	**0**	**#JAKO/0!**	**0,00**
B2HDT1	Conserved integral membrane protein	11,4	**37,3**	342	7	NO_SP	0.999734	0.000275	0.000001	0.000000	0.000000	4	2	**162806,6667**	**4186**	**38,90**	**0,03**
A0A2Z5YJ50	Alpha-glucosidase AglA	4,9	**58,0**	525	0	NO_SP	1.000061	0.000000	0.000000	0.000000	0.000000	4	3	**162705**	**0**	**#JAKO/0!**	**0,00**
A0A2Z5Y7J4	Uncharacterized protein	6,3	**34,6**	322	0	NO_SP	1.000066	0.000000	0.000000	0.000000	0.000000	4	3	**162270**	**6020**	**26,95**	**0,04**
A0A2Z5YI24	Uncharacterized protein	8,5	**21,3**	195	0	NO_SP	0.998852	0.001168	0.000002	0.000001	0.000001	1	3	**162020**	**0**	**#JAKO/0!**	**0,00**
A0A2Z5YDC2	tRNA pseudouridine synthase B	6,4	**31,9**	301	0	NO_SP	0.999893	0.000146	0.000003	0.000000	0.000000	3	3	**161988,3333**	**701**	**230,96**	**0,00**
B2HF60	DUF2236 domain-containing protein	7,5	**34,4**	298	0	NO_SP	0.999865	0.000161	0.000009	0.000000	0.000000	4	3	**161919,3333**	**0**	**#JAKO/0!**	**0,00**
A0A117DTE7	Transcriptional regulator WhiB	5,2	**13,0**	118	0	NO_SP	0.990992	0.008876	0.000110	0.000019	0.000011	4	2	**161802,6667**	**0**	**#JAKO/0!**	**0,00**
B2HQ50	Polyketide synthase, Pks5_1	5,2	**222,3**	2084	0	LIPO	0.448131	0.007293	0.543932	0.000082	0.000080	3	4	**161464**	**0**	**#JAKO/0!**	**0,00**
A0A2Z5YB33	Putative SufE-like protein	4,2	**14,9**	137	0	NO_SP	1.000066	0.000000	0.000000	0.000000	0.000000	4	1	**160796**	**6161**	**26,10**	**0,04**
B2HN66	Hypothetical membrane protein	7,2	**13,0**	128	4	NO_SP	1.000032	0.000000	0.000000	0.000000	0.000000	2	1	**160573,3333**	**2083**	**77,10**	**0,00**
A0A2Z5YA55	Nitroreductase	6,9	**19,1**	174	0	NO_SP	1.000071	0.000000	0.000000	0.000000	0.000000	3	2	**160436,6667**	**0**	**#JAKO/0!**	**0,00**
B2HHI2	Thump_like domain-containing protein	8,7	**37,4**	345	0	NO_SP	0.999669	0.000374	0.000003	0.000001	0.000000	4	3	**160334**	**0**	**#JAKO/0!**	**0,00**
B2HH13	Mycothiol acetyltransferase	5,8	**34,5**	320	0	NO_SP	1.000038	0.000002	0.000000	0.000000	0.000000	4	3	**159823,3333**	**0**	**#JAKO/0!**	**0,00**
B2HF65	Bifunctional protein: CobI-CobJ fusion protein	6,1	**52,3**	489	0	NO_SP	1.000070	0.000000	0.000000	0.000000	0.000000	4	3	**159798**	**0**	**#JAKO/0!**	**0,00**
A0A2Z5YAN1	DUF2510 domain-containing protein	10,1	**17,3**	153	1	NO_SP	0.999856	0.000171	0.000022	0.000000	0.000000	4	3	**159488**	**0**	**#JAKO/0!**	**0,00**
A0A2Z5Y837	NAD-dependent epimerase	10,6	**30,3**	286	0	NO_SP	0.999861	0.000169	0.000001	0.000000	0.000000	4	4	**158666,6667**	**0**	**#JAKO/0!**	**0,00**
A0A2Z5Y971	Ethanolamine ammonia-lyase light chain	6,8	**26,9**	257	0	NO_SP	1.000050	0.000001	0.000000	0.000000	0.000000	4	5	**158613,3333**	**0**	**#JAKO/0!**	**0,00**
B2HR40	ABC transporter, ATP-binding protein	5,7	**64,5**	589	0	NO_SP	1.000047	0.000007	0.000000	0.000000	0.000000	3	1	**158393,3333**	**0**	**#JAKO/0!**	**0,00**
B2HI64	Cell division protein RodA	10,3	**50,6**	469	12	NO_SP	1.000041	0.000004	0.000000	0.000000	0.000000	3	2	**158366**	**0**	**#JAKO/0!**	**0,00**
A0A2Z5YGG0	AMP-binding domain-containing protein	6,4	**18,3**	173	0	NO_SP	1.000040	0.000000	0.000000	0.000000	0.000000	1	1	**158342,3333**	**10033**	**15,78**	**0,06**
A0A2Z5YAB5	Integral membrane protein	10,1	**80,5**	756	13	NO_SP	1.000025	0.000019	0.000000	0.000000	0.000000	4	4	**158200,6667**	**0**	**#JAKO/0!**	**0,00**
B2HLB6	Conserved hypothetical membrane protein	4,5	**47,3**	440	4	NO_SP	0.751641	0.244544	0.000721	0.000615	0.000484	3	2	**157814,3333**	**0**	**#JAKO/0!**	**0,00**
A0A2Z5Y8Y2	Dienelactone hydrolase	6,3	**34,4**	314	0	NO_SP	1.000053	0.000011	0.000000	0.000000	0.000000	4	3	**157733,3333**	**0**	**#JAKO/0!**	**0,00**
A0A2Z5YKM0	PNPLA domain-containing protein	4,4	**29,5**	289	1	NO_SP	0.994127	0.005696	0.000154	0.000011	0.000006	2	3	**156413,3333**	**0**	**#JAKO/0!**	**0,00**
A0A3E2MPF6	Acyl-CoA dehydrogenase	6,9	**21,6**	200	0	NO_SP	1.000053	0.000000	0.000000	0.000000	0.000000	5	2	**156406,6667**	**0**	**#JAKO/0!**	**0,00**
A0A3E2N130	Isoniazid-induced protein IniC	9,5	**52,9**	493	0	NO_SP	1.000062	0.000000	0.000000	0.000000	0.000000	4	6	**156391**	**0**	**#JAKO/0!**	**0,00**
B2HI93	Uncharacterized protein	5,7	**39,7**	368	0	NO_SP	1.000027	0.000009	0.000000	0.000000	0.000000	4	3	**155793,3333**	**2699**	**57,72**	**0,02**
A0A3E2MZM4	Cysteine desulfurase	5,5	**40,7**	393	0	NO_SP	1.000051	0.000000	0.000000	0.000000	0.000000	4	2	**155324**	**0**	**#JAKO/0!**	**0,00**
B2HFW7	Uncharacterized protein	6,5	**27,9**	259	0	NO_SP	0.999917	0.000091	0.000008	0.000000	0.000000	4	3	**154999**	**0**	**#JAKO/0!**	**0,00**
A0A124BV66	Cold-shock protein	4,6	**6,9**	62	0	NO_SP	1.000055	0.000000	0.000000	0.000000	0.000000	3	5	**154967**	**5654**	**27,41**	**0,00**
A0A124BX33	Uncharacterized protein	4,7	**26,1**	240	0	NO_SP	0.876080	0.122936	0.000549	0.000172	0.000102	4	3	**154672**	**0**	**#JAKO/0!**	**0,00**
A0A117DUJ7	ABC transporter substrate-binding protein	5,5	**46,6**	424	0	NO_SP	1.000031	0.000003	0.000000	0.000000	0.000000	4	4	**154528,3333**	**0**	**#JAKO/0!**	**0,00**
A0A3E2MX91	Alpha/beta hydrolase family protein	4,5	**24,7**	239	0	NO_SP	1.000070	0.000000	0.000000	0.000000	0.000000	4	3	**153671,6667**	**0**	**#JAKO/0!**	**0,00**
A0A2Z5YNQ3	Putative glycerophosphoryl diester phosphodiesterase 1	10,1	**28,8**	272	0	NO_SP	1.000047	0.000001	0.000000	0.000000	0.000000	4	4	**153174**	**0**	**#JAKO/0!**	**0,00**
B2HN38	Uncharacterized protein	4,2	**11,1**	98	0	NO_SP	1.000076	0.000000	0.000000	0.000000	0.000000	3	3	**153098,6667**	**1028**	**148,90**	**0,00**
A0A2Z5YCU0	Amidohydro-rel domain-containing protein	5,5	**39,0**	363	0	NO_SP	1.000046	0.000005	0.000000	0.000000	0.000000	4	5	**152772,6667**	**0**	**#JAKO/0!**	**0,00**
B2HIG5	Acetolactate synthase	5,1	**66,1**	618	0	NO_SP	1.000054	0.000001	0.000000	0.000000	0.000000	1	1	**152496,6667**	**9556**	**15,96**	**0,06**
A0A2Z5YBF0	Nucleoside triphosphate pyrophosphatase	4,8	**22,5**	216	0	NO_SP	1.000039	0.000001	0.000000	0.000000	0.000000	4	3	**152319,6667**	**0**	**#JAKO/0!**	**0,00**
A0A2Z5Y817	Rieske domain-containing protein	5,0	**41,8**	376	0	NO_SP	1.000073	0.000000	0.000000	0.000000	0.000000	4	3	**151470,6667**	**0**	**#JAKO/0!**	**0,00**
A0A100I764	Cyclase	4,6	**35,1**	320	0	NO_SP	1.000069	0.000000	0.000000	0.000000	0.000000	4	4	**151410**	**0**	**#JAKO/0!**	**0,00**
A0A2Z5YMR0	Acyl-CoA dehydrogenase	4,7	**69,8**	665	0	NO_SP	0.999152	0.000870	0.000006	0.000001	0.000001	1	7	**151261,3333**	**0**	**#JAKO/0!**	**0,00**
A0A2Z5YFT4	S-adenosyl-L-methionine-dependent methyltransferase	7,3	**31,2**	285	0	NO_SP	0.999967	0.000055	0.000020	0.000000	0.000000	4	2	**151227**	**2179**	**69,40**	**0,01**
B2HLE6	Conserved hypothetical membrane protein	4,8	**18,0**	169	0	NO_SP	1.000036	0.000000	0.000000	0.000000	0.000000	3	5	**151040**	**0**	**#JAKO/0!**	**0,00**
B2HQJ7	Methylated-DNA--protein-cysteine methyltransferase	4,8	**18,7**	172	0	NO_SP	1.000036	0.000011	0.000001	0.000000	0.000000	4	4	**150663,3333**	**404**	**372,96**	**0,00**
A0A2Z5YNB6	DNA-binding response regulator	5,2	**24,8**	224	0	NO_SP	1.000075	0.000000	0.000000	0.000000	0.000000	3	3	**150605,3333**	**0**	**#JAKO/0!**	**0,00**
A0A124BUC1	Membrane protein	5,8	**44,8**	405	0	NO_SP	1.000053	0.000001	0.000000	0.000000	0.000000	4	4	**150601,3333**	**342**	**440,14**	**0,00**
A0A2Z5YAR8	Trehalose 6-phosphate phosphatase	4,8	**41,1**	390	0	NO_SP	1.000063	0.000003	0.000000	0.000000	0.000000	4	2	**149992,6667**	**0**	**#JAKO/0!**	**0,00**
B2HT44	Exodeoxyribonuclease 7 small subunit	4,0	**9,1**	82	0	NO_SP	1.000073	0.000000	0.000000	0.000000	0.000000	4	2	**149893,3333**	**24082**	**6,22**	**0,16**
A0A100I4B7	Lipoprotein	4,5	**33,0**	313	0	SP	0.405378	0.592783	0.000927	0.000438	0.000231	4	3	**149679,3333**	**0**	**#JAKO/0!**	**0,00**
B2HP24	Uncharacterized protein	4,7	**27,3**	251	0	NO_SP	1.000071	0.000000	0.000000	0.000000	0.000000	4	4	**149164,6667**	**0**	**#JAKO/0!**	**0,00**
B2HRP3	Amidohydro_3 domain-containing protein	4,5	**57,5**	534	0	NO_SP	1.000025	0.000014	0.000000	0.000000	0.000000	4	3	**149016,6667**	**320**	**465,98**	**0,00**
A0A2Z5Y9F3	Acyl-CoA dehydrogenase	6,1	**42,2**	382	0	NO_SP	1.000050	0.000001	0.000000	0.000000	0.000000	3	4	**148650,3333**	**0**	**#JAKO/0!**	**0,00**
A0A2Z5YIJ0	SIR2 family protein	5,5	**59,3**	533	0	NO_SP	1.000075	0.000000	0.000000	0.000000	0.000000	3	4	**148647**	**1665**	**89,30**	**0,00**
A0A3E2N243	Phosphotransferase enzyme family protein	5,0	**38,6**	351	0	NO_SP	1.000063	0.000000	0.000000	0.000000	0.000000	4	3	**148620**	**0**	**#JAKO/0!**	**0,00**
A0A2Z5YDS6	Thioredoxin	4,3	**11,9**	109	0	NO_SP	1.000072	0.000000	0.000000	0.000000	0.000000	4	3	**147604,3333**	**5203**	**28,37**	**0,00**
A0A2Z5YEI6	NUDIX hydrolase	4,9	**22,8**	207	0	NO_SP	1.000064	0.000000	0.000000	0.000000	0.000000	4	2	**147135**	**1240**	**118,69**	**0,00**
B2HIQ4	Uncharacterized protein	5,4	**47,3**	422	0	NO_SP	1.000081	0.000000	0.000000	0.000000	0.000000	4	5	**146810,3333**	**0**	**#JAKO/0!**	**0,00**
A0A2Z5YJU0	Putative threonylcarbamoyl-AMP synthase	4,6	**22,2**	216	0	NO_SP	1.000039	0.000004	0.000000	0.000000	0.000000	4	3	**146792,3333**	**0**	**#JAKO/0!**	**0,00**
A0A2Z5YAU7	D-3-phosphoglycerate dehydrogenase	5,0	**33,1**	312	0	NO_SP	1.000081	0.000000	0.000000	0.000000	0.000000	4	4	**146719,6667**	**3917**	**37,46**	**0,00**
B2HDY5	Lysine 6-aminotransferase	5,2	**48,8**	446	0	NO_SP	1.000016	0.000026	0.000001	0.000000	0.000000	4	5	**146308,3333**	**0**	**#JAKO/0!**	**0,00**
A0A3E2MSK3	Putative cation-transporting ATPase F	5,9	**84,3**	791	9	NO_SP	0.999893	0.000122	0.000000	0.000000	0.000000	4	4	**146109,3333**	**468**	**312,22**	**0,00**
A0A117DXR5	Acetaldehyde dehydrogenase	4,6	**31,7**	302	0	NO_SP	1.000028	0.000000	0.000000	0.000000	0.000000	3	1	**146033**	**0**	**#JAKO/0!**	**0,00**
A0A2Z5YAF2	MFS transporter	6,5	**54,6**	526	13	NO_SP	1.000030	0.000001	0.000000	0.000000	0.000000	4	2	**145896,3333**	**0**	**#JAKO/0!**	**0,00**
B2HJ24	Conserved transcriptional regulatory protein	5,4	**17,9**	162	0	NO_SP	1.000037	0.000000	0.000000	0.000000	0.000000	3	4	**145533,6667**	**0**	**#JAKO/0!**	**0,00**
A0A117DXS2	Putative oxidoreductase	5,2	**30,4**	278	0	NO_SP	1.000037	0.000000	0.000000	0.000000	0.000000	3	3	**145518**	**0**	**#JAKO/0!**	**0,00**
B2HF76	Conserved exported or membrane protein	10,9	**22,7**	212	1	NO_SP	1.000036	0.000002	0.000001	0.000000	0.000000	4	2	**145050**	**0**	**#JAKO/0!**	**0,00**
A0A2Z5YMW4	2-C-methyl-D-erythritol 2,4-cyclodiphosphate synthase	6,3	**16,9**	165	0	NO_SP	0.998427	0.001613	0.000005	0.000001	0.000001	4	3	**144614,6667**	**0**	**#JAKO/0!**	**0,00**
B2HR00	DUF402 domain-containing protein	6,3	**17,7**	158	0	NO_SP	1.000079	0.000000	0.000000	0.000000	0.000000	4	3	**144313,6667**	**0**	**#JAKO/0!**	**0,00**
A0A2Z5YIV4	DegV domain-containing protein	7,5	**28,9**	281	0	NO_SP	1.000064	0.000001	0.000000	0.000000	0.000000	4	3	**144247,6667**	**552**	**261,16**	**0,00**

A0A100IAW7	7,8-dihydro-8-oxoguanine-triphosphatase	7,8	**20,4**	189	0	NO_SP	0.997995	0.001893	0.000081	0.000005	0.000006	4	3	**144078,3333**	**0**	**#JAKO/0!**	**0,00**
B2HI40	Short-chain type dehydrogenase/reductase	4,9	**30,5**	301	0	NO_SP	1.000021	0.000012	0.000000	0.000000	0.000000	4	3	**144040,6667**	**0**	**#JAKO/0!**	**0,00**
B2HE97	Nitroreductase	11,2	**23,3**	209	0	NO_SP	1.000070	0.000000	0.000000	0.000000	0.000000	3	2	**143717**	**0**	**#JAKO/0!**	**0,00**
A0A3E2MZY7	Glycerophosphoryl diester phosphodiesterase	7,4	**25,1**	230	0	NO_SP	1.000050	0.000000	0.000000	0.000000	0.000000	4	4	**143463,3333**	**1501**	**95,58**	**0,00**
A0A2Z5YBD8	Thymidylate kinase	4,5	**23,0**	217	0	NO_SP	0.998965	0.000964	0.000058	0.000004	0.000002	3	3	**142036,6667**	**0**	**#JAKO/0!**	**0,00**
B2HN58	Uncharacterized protein	9,9	**15,1**	143	1	NO_SP	0.823223	0.148969	0.007557	0.001370	0.000676	3	2	**141982**	**0**	**#JAKO/0!**	**0,00**
A0A2Z5YCZ2	Putative O-phosphotransferase	5,0	**23,9**	209	0	NO_SP	1.000070	0.000000	0.000000	0.000000	0.000000	3	3	**141925**	**0**	**#JAKO/0!**	**0,00**
B2HJL3	UPF0109 protein MMAR_1800	6,7	**8,6**	80	0	NO_SP	1.000059	0.000000	0.000000	0.000000	0.000000	3	3	**141856**	**22246**	**6,38**	**0,16**
A0A3E2MU29	YcaO-like family protein	6,3	**45,4**	420	0	NO_SP	1.000041	0.000000	0.000000	0.000000	0.000000	4	5	**141726**	**0**	**#JAKO/0!**	**0,00**
A0A2Z5YGF6	Guanylate cyclase domain-containing protein	7,2	**26,4**	248	0	NO_SP	1.000028	0.000003	0.000000	0.000000	0.000000	4	4	**141430**	**0**	**#JAKO/0!**	**0,00**
A0A100IEV3	Uncharacterized protein	5,9	**13,0**	117	0	NO_SP	1.000063	0.000000	0.000000	0.000000	0.000000	3	1	**141415,3333**	**0**	**#JAKO/0!**	**0,00**
B2HD64	Enoyl-CoA hydratase, EchA8_6	5,1	**27,0**	252	1	NO_SP	1.000072	0.000001	0.000000	0.000000	0.000000	3	3	**141273,0333**	**0**	**#JAKO/0!**	**0,00**
A0A2Z5YEW4	Putative Na(+)/H(+) antiporter	7,1	**51,8**	489	12	NO_SP	1.000001	0.000000	0.000000	0.000000	0.000000	4	3	**140996,6667**	**1771**	**79,61**	**0,00**
A0A117DW50	Putative secreted protein	4,4	**15,5**	147	0	NO_SP	1.000049	0.000016	0.000000	0.000000	0.000000	4	2	**140750,3333**	**0**	**#JAKO/0!**	**0,00**
A0A2Z5YFH3	Acetyl-CoA acetyltransferase	5,9	**53,2**	494	0	NO_SP	1.000032	0.000000	0.000000	0.000000	0.000000	3	5	**140696,6667**	**0**	**#JAKO/0!**	**0,00**
A0A2Z5YLR7	Putative enoyl-CoA hydratase/isomerase	6,6	**28,1**	265	0	NO_SP	1.000046	0.000001	0.000000	0.000000	0.000000	4	4	**140399,3333**	**2890**	**48,58**	**0,00**
B2HEM6	dTDP-4-dehydrorhamnose reductase	4,8	**31,6**	308	0	NO_SP	0.999783	0.000251	0.000001	0.000000	0.000000	2	1	**140373,3333**	**0**	**#JAKO/0!**	**0,00**
A0A2Z5YJV8	pH-sensitive adenylate cyclase	4,8	**45,5**	427	0	NO_SP	0.999148	0.000859	0.000010	0.000001	0.000001	4	5	**140293,6667**	**0**	**#JAKO/0!**	**0,00**
A0A3E2N2G2	Putative ATP-dependent DNA ligase YkoU	6,4	**38,4**	346	0	NO_SP	1.000067	0.000000	0.000000	0.000000	0.000000	4	4	**140166,6667**	**0**	**#JAKO/0!**	**0,00**
B2HI16	Conserved hypothetical membrane protein	5,8	**32,9**	312	1	NO_SP	1.000031	0.000002	0.000000	0.000000	0.000000	4	2	**140148**	**37203**	**3,77**	**0,27**
A0A2Z5YJS7	dITP/XTP pyrophosphatase	5,0	**21,0**	204	0	NO_SP	1.000064	0.000000	0.000000	0.000000	0.000000	4	2	**139990**	**0**	**#JAKO/0!**	**0,00**
A0A2Z5YMG0	Pyridoxine/pyridoxamine 5-phosphate oxidase	6,6	**24,2**	219	0	NO_SP	1.000036	0.000002	0.000000	0.000000	0.000000	4	3	**139943,6667**	**0**	**#JAKO/0!**	**0,00**
A0A100I3P6	PPE family protein	3,8	**24,8**	245	0	NO_SP	1.000052	0.000001	0.000000	0.000000	0.000000	4	1	**139933,6667**	**11927**	**11,73**	**0,00**
A0A2Z5YIW0	Hydrolase	5,0	**47,9**	445	0	NO_SP	1.000054	0.000001	0.000000	0.000000	0.000000	4	4	**139393,6667**	**0**	**#JAKO/0!**	**0,00**
A0A2Z5YFP1	Amino acid oxidase	4,9	**35,2**	327	1	NO_SP	0.999886	0.000134	0.000003	0.000000	0.000000	5	4	**139364,3333**	**0**	**#JAKO/0!**	**0,00**
A0A2Z5YA13	Methyltransferase/methylase	5,0	**37,1**	340	0	NO_SP	0.999591	0.000427	0.000006	0.000001	0.000000	4	6	**139345,6667**	**0**	**#JAKO/0!**	**0,00**
A0A100I435	3-oxoacyl-ACP reductase	6,6	**23,6**	224	0	NO_SP	0.997538	0.002298	0.000186	0.000007	0.000003	4	2	**138797,6667**	**0**	**#JAKO/0!**	**0,00**
A0A2Z5Y8R4	Fatty acid metabolism regulator protein	4,6	**22,7**	208	0	NO_SP	1.000042	0.000000	0.000000	0.000000	0.000000	3	5	**138740,3333**	**0**	**#JAKO/0!**	**0,00**
B2HMX3	Conserved transmembrane protein	5,6	**40,6**	389	8	NO_SP	1.000017	0.000003	0.000000	0.000000	0.000000	4	2	**138690**	**1490**	**93,11**	**0,00**
A0A2Z5YL33	Uncharacterized protein	4,3	**22,8**	211	0	NO_SP	0.999827	0.000204	0.000001	0.000000	0.000000	4	2	**138593,3333**	**0**	**#JAKO/0!**	**0,00**
A0A2Z5YDI1	Alpha/beta hydrolase	8,0	**37,0**	340	0	NO_SP	1.000053	0.000000	0.000000	0.000000	0.000000	4	5	**138296,3333**	**0**	**#JAKO/0!**	**0,00**
B2HHW7	Conserved hypothetical secreted protein	8,5	**8,9**	80	1	SP	0.000228	0.999169	0.000154	0.000146	0.000132	1	1	**138134,3333**	**0**	**#JAKO/0!**	**0,00**
A0A2Z5Y892	GCN5-like N-acetyltransferase	8,2	**23,4**	210	0	NO_SP	1.000069	0.000005	0.000000	0.000000	0.000000	4	3	**137751**	**0**	**#JAKO/0!**	**0,00**
B2HNA8	2-dehydropantoate 2-reductase	5,6	**28,5**	268	0	NO_SP	1.000055	0.000003	0.000000	0.000000	0.000000	4	2	**137742,5233**	**0**	**#JAKO/0!**	**0,00**
B2HLY1	UPF0678 fatty acid-binding protein-like protein MMAR_1995	6,2	**17,4**	161	0	NO_SP	1.000051	0.000000	0.000000	0.000000	0.000000	2	1	**137669,3333**	**0**	**#JAKO/0!**	**0,00**
B2HMZ6	Transcriptional regulatory protein	5,1	**21,7**	206	0	NO_SP	1.000049	0.000000	0.000000	0.000000	0.000000	3	3	**137553,3333**	**0**	**#JAKO/0!**	**0,00**
B2HKM6	Conserved membrane protein	7,1	**25,2**	237	4	NO_SP	1.000051	0.000000	0.000000	0.000000	0.000000	2	2	**137457,6667**	**0**	**#JAKO/0!**	**0,00**
B2HJ49	Conserved membrane protein	9,2	**16,7**	158	4	NO_SP	1.000041	0.000001	0.000000	0.000000	0.000000	4	1	**136568**	**0**	**#JAKO/0!**	**0,00**
A0A100IEU3	MerR family transcriptional regulator	11,0	**12,8**	112	0	NO_SP	1.000051	0.000000	0.000000	0.000000	0.000000	3	2	**136303,3333**	**5156**	**26,44**	**0,00**
B2HIY7	Transcriptional regulatory protein (Probably TetR-family)	5,4	**23,0**	200	0	NO_SP	1.000049	0.000001	0.000000	0.000000	0.000000	4	6	**136296,6667**	**0**	**#JAKO/0!**	**0,00**
B2HDX8	Esterase lipoprotein LpqC	8,5	**31,9**	305	0	LIPO	0.000000	0.000008	1.000051	0.000000	0.000000	4	4	**136144,3333**	**0**	**#JAKO/0!**	**0,00**
B2HGE7	NADH-quinone oxidoreductase subunit B	7,2	**20,2**	184	0	NO_SP	1.000081	0.000000	0.000000	0.000000	0.000000	3	3	**135122,3333**	**0**	**#JAKO/0!**	**0,00**
B2HCZ9	Conserved hypothetical transmembrane protein	9,9	**29,9**	280	4	NO_SP	0.587005	0.408005	0.003371	0.000451	0.000349	4	2	**134513**	**0**	**#JAKO/0!**	**0,00**
B2HDY3	DUF1338 domain-containing protein	5,5	**45,3**	423	0	NO_SP	1.000022	0.000024	0.000002	0.000000	0.000000	4	4	**134367**	**0**	**#JAKO/0!**	**0,00**
B2HNE5	Bifunctional protein PyrR	5,1	**20,1**	187	0	NO_SP	1.000057	0.000000	0.000000	0.000000	0.000000	3	2	**134206,3333**	**0**	**#JAKO/0!**	**0,00**
A0A2Z5Y8A1	Putative enoyl-CoA hydratase 1	5,6	**16,0**	151	0	NO_SP	0.998803	0.001178	0.000015	0.000004	0.000002	4	3	**133957**	**0**	**#JAKO/0!**	**0,00**
B2HEN0	DNA methylase	7,4	**57,4**	528	0	NO_SP	1.000076	0.000001	0.000000	0.000000	0.000000	4	2	**133776,6667**	**5885**	**22,73**	**0,04**
A0A3E2MY69	Peptidoglycan-N-acetylglucosamine deacetylase	6,1	**29,4**	277	0	SP	0.062530	0.936100	0.000604	0.000261	0.000233	4	1	**133520,6667**	**0**	**#JAKO/0!**	**0,00**
A0A117DX42	Alanine and valine rich exported protein	6,5	**18,0**	171	0	SP	0.000313	0.999040	0.000161	0.000181	0.000148	4	3	**132830,6667**	**0**	**#JAKO/0!**	**0,00**
B2HIN4	Uncharacterized protein	6,5	**21,2**	185	0	NO_SP	1.000074	0.000002	0.000000	0.000000	0.000000	4	3	**132773,6667**	**0**	**#JAKO/0!**	**0,00**
B2HMB6	FAD-dependent oxidoreductase	10,0	**42,4**	392	0	NO_SP	1.000054	0.000002	0.000000	0.000000	0.000000	4	3	**132435**	**0**	**#JAKO/0!**	**0,00**
A0A2Z5YGS3	Purine nucleoside phosphorylase	5,0	**22,7**	220	0	NO_SP	1.000064	0.000000	0.000000	0.000000	0.000000	2	1	**132403,3333**	**0**	**#JAKO/0!**	**0,00**
B2HRL0	Mandelate racemase	6,2	**41,3**	385	0	NO_SP	1.000066	0.000000	0.000000	0.000000	0.000000	4	4	**131919,3333**	**199**	**664,53**	**0,00**
A0A100I1H6	Membrane protein	4,3	**34,2**	327	1	NO_SP	0.980636	0.017954	0.001355	0.000036	0.000019	4	2	**131663**	**0**	**#JAKO/0!**	**0,00**
A0A2Z5YFW7	Cutinase	4,2	**19,7**	195	0	NO_SP	0.898265	0.100877	0.000266	0.000272	0.000137	4	1	**131632,3333**	**0**	**#JAKO/0!**	**0,00**
B2HPT5	RNA methyltransferase	6,3	**23,4**	214	0	NO_SP	1.000046	0.000000	0.000000	0.000000	0.000000	3	3	**131601,6667**	**2423**	**54,32**	**0,00**
A0A2Z5YNK1	S-adenosyl-L-methionine-dependent methyltransferase	4,4	**34,5**	314	0	NO_SP	1.000041	0.000010	0.000000	0.000000	0.000000	4	5	**130981**	**0**	**#JAKO/0!**	**0,00**
A0A2Z5YG98	Uncharacterized protein	4,9	**37,0**	340	0	NO_SP	1.000058	0.000000	0.000000	0.000000	0.000000	1	4	**130383**	**0**	**#JAKO/0!**	**0,00**
A0A2Z5YA33	DAGKc domain-containing protein	6,3	**29,7**	281	0	NO_SP	0.999852	0.000193	0.000001	0.000000	0.000000	4	3	**130237,3333**	**0**	**#JAKO/0!**	**0,00**
B2HHV2	Conserved protein	5,9	**18,6**	167	0	NO_SP	1.000039	0.000008	0.000001	0.000000	0.000000	4	3	**130078**	**0**	**#JAKO/0!**	**0,00**
A0A2Z5YFT8	TetR family transcriptional regulator	9,4	**20,2**	183	0	NO_SP	1.000015	0.000000	0.000000	0.000000	0.000000	3	3	**129939,3333**	**0**	**#JAKO/0!**	**0,00**
B2HFH7	Glutaredoxin protein	6,2	**8,9**	84	0	NO_SP	1.000081	0.000000	0.000000	0.000000	0.000000	2	1	**129866,6667**	**0**	**#JAKO/0!**	**0,00**
B2HFY3	Monophosphatase CysQ	5,8	**28,5**	263	0	NO_SP	1.000052	0.000001	0.000000	0.000000	0.000000	4	3	**129770,3333**	**0**	**#JAKO/0!**	**0,00**
B2HMW9	Uncharacterized protein	10,1	**19,8**	179	0	NO_SP	1.000058	0.000004	0.000000	0.000000	0.000000	4	2	**129752,3333**	**0**	**#JAKO/0!**	**0,00**
A0A2Z5YCV0	Hydrogenase expression protein HypE	5,7	**34,7**	322	0	NO_SP	1.000041	0.000001	0.000000	0.000000	0.000000	4	3	**129417,3333**	**0**	**#JAKO/0!**	**0,00**
B2HKR5	Integral membrane efflux protein EfpA	9,0	**55,5**	530	14	NO_SP	0.998986	0.000859	0.000060	0.000006	0.000007	2	2	**129329,3333**	**0**	**#JAKO/0!**	**0,00**
B2HHP0	DUF2236 domain-containing protein	10,1	**30,9**	271	0	NO_SP	0.986051	0.013833	0.000063	0.000014	0.000011	3	3	**129106,6667**	**0**	**#JAKO/0!**	**0,00**
A0A2Z5YLU2	Uncharacterized protein	6,3	**34,0**	320	0	NO_SP	1.000071	0.000000	0.000000	0.000000	0.000000	4	3	**128943,3333**	**0**	**#JAKO/0!**	**0,00**
A0A2Z5YI44	F420-dependent glucose-6-phosphate dehydrogenase	4,7	**31,9**	286	0	NO_SP	1.000049	0.000003	0.000000	0.000000	0.000000	3	2	**128645,3333**	**897**	**143,42**	**0,00**
A0A117DTK2	IclR family transcriptional regulator	9,3	**23,2**	220	0	NO_SP	0.999999	0.000042	0.000000	0.000000	0.000000	4	3	**128554**	**0**	**#JAKO/0!**	**0,00**
A0A3E2MVU7	Tetracycline repressor protein class A from transposon 1721	9,9	**29,6**	267	0	NO_SP	1.000035	0.000000	0.000000	0.000000	0.000000	5	2	**128505,3333**	**0**	**#JAKO/0!**	**0,00**
A0A2Z5YEA5	Lipase	5,5	**48,4**	446	0	NO_SP	1.000016	0.000026	0.000000	0.000000	0.000000	3	3	**128308,3333**	**0**	**#JAKO/0!**	**0,00**
B2HL96	Phospholipase C	6,4	**56,0**	516	0	TAT	0.000000	0.000000	0.000000	0.999979	0.000015	7	4	**128218,3333**	**0**	**#JAKO/0!**	**0,00**
A0A100I604	Ribonuclease	7,0	**29,7**	274	4	NO_SP	0.987920	0.011116	0.000627	0.000102	0.000072	4	2	**128087,3333**	**363**	**352,57**	**0,00**
A0A100IAV3	Lipoprotein LprA	4,6	**23,3**	229	0	LIPO	0.000073	0.000002	0.999982	0.000000	0.000000	3	2	**128010,3333**	**464**	**275,65**	**0,00**
B2HEF5	Non-homologous end joining protein Ku	4,7	**32,8**	291	0	NO_SP	0.960390	0.039404	0.000070	0.000059	0.000034	5	4	**127780,3333**	**281**	**455,00**	**0,00**
A0A2Z5Y8F5	Alpha-methylacyl-CoA racemase	4,8	**41,8**	389	0	NO_SP	1.000025	0.000000	0.000000	0.000000	0.000000	5	5	**127681,3333**	**0**	**#JAKO/0!**	**0,00**

B2HLL7	Amidohydro-rel domain-containing protein	5,1	**28,4**	262	0	NO_SP	1.000010	0.000035	0.000001	0.000000	0.000000	4	3	**126637**	**0**	**#JAKO/0!**	**0,00**
A0A2Z5YCD2	Glutamate--tRNA ligase	4,9	**51,5**	468	0	NO_SP	1.000055	0.000000	0.000000	0.000000	0.000000	2	1	**126219,6667**	**0**	**#JAKO/0!**	**0,00**
A0A2Z5Y8X0	TetR_C_16 domain-containing protein	8,0	**16,0**	151	0	NO_SP	1.000057	0.000000	0.000000	0.000000	0.000000	3	2	**126018**	**6087**	**20,70**	**0,00**
A0A2Z5YJ13	SnoaL-like domain-containing protein	6,3	**15,8**	141	0	NO_SP	1.000032	0.000006	0.000000	0.000000	0.000000	4	2	**125875,6667**	**0**	**#JAKO/0!**	**0,00**
B2HLV8	Uncharacterized protein	6,1	**29,7**	271	0	NO_SP	1.000041	0.000001	0.000000	0.000000	0.000000	4	4	**125649**	**0**	**#JAKO/0!**	**0,00**
B2HPS7	Uncharacterized protein	5,1	**31,5**	296	0	NO_SP	0.999975	0.000086	0.000000	0.000000	0.000000	4	3	**125114,3333**	**0**	**#JAKO/0!**	**0,00**
A0A100I3R0	Cold-shock DNA-binding domain protein	7,3	**14,4**	129	0	NO_SP	1.000060	0.000000	0.000000	0.000000	0.000000	3	3	**124838,3333**	**11803**	**10,58**	**0,09**
B2HSG0	Conserved hypothetical transmembrane protein	4,9	**36,5**	334	0	NO_SP	1.000056	0.000000	0.000000	0.000000	0.000000	4	3	**124718**	**0**	**#JAKO/0!**	**0,00**
B2HHX2	Short-chain type dehydrogenase/reductase	5,1	**29,0**	277	0	NO_SP	1.000059	0.000003	0.000000	0.000000	0.000000	4	3	**124703**	**0**	**#JAKO/0!**	**0,00**
A0A2Z5YN42	Uncharacterized protein	6,8	**46,3**	424	0	NO_SP	1.000049	0.000000	0.000000	0.000000	0.000000	4	2	**124600**	**333**	**374,53**	**0,00**
B2HPP9	Acyl-CoA dehydrogenase	6,1	**41,5**	386	0	NO_SP	1.000032	0.000003	0.000000	0.000000	0.000000	3	3	**124507**	**0**	**#JAKO/0!**	**0,00**
B2HER7	Linoleoyl-CoA desaturase, DesA3_1	9,1	**48,3**	428	0	NO_SP	1.000034	0.000005	0.000000	0.000000	0.000000	4	2	**123850,6667**	**0**	**#JAKO/0!**	**0,00**
A0A2Z5YEA9	Glycosyl transferase family 2	9,5	**28,2**	249	0	NO_SP	1.000058	0.000000	0.000000	0.000000	0.000000	3	2	**123814,6667**	**2951**	**41,95**	**0,00**
A0A100IAQ3	Isoniazid inducible protein	5,7	**66,2**	602	0	NO_SP	1.000066	0.000000	0.000000	0.000000	0.000000	4	3	**123780,3333**	**1457**	**84,96**	**0,00**
A0A2Z5YGZ8	AraC family transcriptional regulator	9,7	**37,7**	342	0	NO_SP	1.000051	0.000001	0.000000	0.000000	0.000000	3	2	**123494**	**0**	**#JAKO/0!**	**0,00**
A0A2Z5YJE0	Uncharacterized protein	4,7	**18,5**	162	0	NO_SP	1.000060	0.000000	0.000000	0.000000	0.000000	1	2	**122888,3333**	**0**	**#JAKO/0!**	**0,00**
A0A2Z5YD18	Flavodoxin	5,9	**16,1**	151	0	NO_SP	1.000056	0.000000	0.000000	0.000000	0.000000	3	3	**122558,6667**	**0**	**#JAKO/0!**	**0,00**
B2HDR1	Uncharacterized protein	5,3	**20,3**	182	0	NO_SP	1.000028	0.000014	0.000002	0.000000	0.000000	3	4	**122149,6667**	**0**	**#JAKO/0!**	**0,00**
A0A3E2MYL4	DUF2236 domain-containing protein	6,5	**48,9**	443	0	NO_SP	1.000040	0.000006	0.000000	0.000000	0.000000	2	4	**121885**	**0**	**#JAKO/0!**	**0,00**
B2HEF9	Phosphate transport system permease protein	10,8	**34,2**	324	6	NO_SP	1.000033	0.000020	0.000000	0.000000	0.000000	4	2	**121257,3333**	**0**	**#JAKO/0!**	**0,00**
B2HLA8	Transcriptional regulatory protein (Probably ArsR-family)	6,0	**14,0**	131	0	NO_SP	1.000038	0.000007	0.000000	0.000000	0.000000	4	2	**121023**	**0**	**#JAKO/0!**	**0,00**
A0A2Z5YPD1	Cyclase	5,3	**113,1**	1057	0	NO_SP	1.000043	0.000001	0.000000	0.000000	0.000000	4	6	**120994,3333**	**0**	**#JAKO/0!**	**0,00**
A0A2Z5YDY6	Monooxygenase	8,5	**55,8**	494	0	NO_SP	0.993647	0.005663	0.000664	0.000032	0.000011	4	3	**120727,6667**	**2407**	**50,16**	**0,00**
B2HHW8	Conserved hypothetical membrane protein	4,8	**26,7**	247	1	NO_SP	0.998908	0.001052	0.000021	0.000001	0.000001	3	3	**120423,6667**	**0**	**#JAKO/0!**	**0,00**
A0A100IC27	Methyltransferase domain protein	7,8	**28,6**	259	0	NO_SP	1.000053	0.000000	0.000000	0.000000	0.000000	3	3	**120350,6667**	**0**	**#JAKO/0!**	**0,00**
B2HQ19	Glycosyltransferase, LosA	8,7	**38,6**	344	2	NO_SP	1.000047	0.000000	0.000000	0.000000	0.000000	4	3	**120328,3333**	**0**	**#JAKO/0!**	**0,00**
A0A3E2MTN9	Undecaprenyl-phosphate mannosyltransferase	8,9	**23,0**	218	0	NO_SP	1.000034	0.000018	0.000001	0.000000	0.000000	4	4	**120276,3333**	**180**	**669,22**	**0,00**
A0A100IGP6	Monooxygenase component A	6,0	**41,9**	371	0	NO_SP	1.000067	0.000000	0.000000	0.000000	0.000000	3	1	**120233,3333**	**0**	**#JAKO/0!**	**0,00**
A0A2Z5YMJ3	3-ketoacyl-CoA thiolase	6,0	**41,4**	391	0	NO_SP	1.000079	0.000000	0.000000	0.000000	0.000000	3	3	**120216,6667**	**0**	**#JAKO/0!**	**0,00**
A0A117DV39	Membrane protein	11,8	**13,0**	119	1	NO_SP	0.999876	0.000167	0.000002	0.000000	0.000000	3	2	**119046,3333**	**0**	**#JAKO/0!**	**0,00**
B2HFX5	Phosphoribosyl-ATP pyrophosphatase	4,7	**10,3**	93	0	NO_SP	1.000042	0.000002	0.000000	0.000000	0.000000	3	3	**118955**	**5086**	**23,39**	**0,00**
A0A2Z5YLL3	Methyltransf_21 domain-containing protein	7,7	**35,4**	319	0	NO_SP	1.000072	0.000001	0.000000	0.000000	0.000000	4	3	**118240,6667**	**403**	**293,64**	**0,00**
B2HE99	Conserved hypothetical transmembrane protein	4,4	**43,3**	392	3	NO_SP	1.000052	0.000003	0.000000	0.000000	0.000000	4	2	**118239,3333**	**0**	**#JAKO/0!**	**0,00**
A0A2Z5Y8D9	Putative cytochrome P450 138	10,3	**48,6**	432	0	NO_SP	1.000042	0.000000	0.000000	0.000000	0.000000	4	3	**118233**	**1262**	**93,71**	**0,00**
B2HIT9	Ferredoxin	3,7	**11,8**	108	0	NO_SP	1.000014	0.000064	0.000001	0.000000	0.000000	4	1	**118067,6667**	**4869**	**24,25**	**0,04**
A0A2Z5YPI3	Uncharacterized protein	4,3	**9,7**	91	0	NO_SP	1.000044	0.000014	0.000000	0.000000	0.000000	1	1	**117948,3333**	**0**	**#JAKO/0!**	**0,00**
B2HER6	Linoleoyl-CoA desaturase, DesA3	9,7	**48,7**	428	0	NO_SP	1.000048	0.000006	0.000000	0.000000	0.000000	2	2	**117884,6667**	**0**	**#JAKO/0!**	**0,00**
A0A100I352	Butyryl-CoA dehydrogenase	6,4	**34,9**	322	1	NO_SP	0.999571	0.000437	0.000003	0.000002	0.000001	4	3	**117614**	**127**	**922,56**	**0,00**
A0A100IHF5	Cytochrome P450 steroid C27-monooxygenase	4,8	**48,2**	428	0	NO_SP	1.000030	0.000002	0.000000	0.000000	0.000000	3	1	**117478,3333**	**1511**	**77,76**	**0,00**
A0A2Z5YEV1	SAM-dependent methyltransferase	6,5	**19,3**	185	0	NO_SP	1.000047	0.000000	0.000000	0.000000	0.000000	3	1	**117383,6667**	**0**	**#JAKO/0!**	**0,00**
A0A2Z5YF37	DUF5073 domain-containing protein	6,7	**13,3**	127	1	NO_SP	0.999833	0.000209	0.000003	0.000000	0.000000	4	3	**117236,3333**	**0**	**#JAKO/0!**	**0,00**
A0A2Z5YI01	Acyl-CoA dehydrogenase	6,4	**36,6**	342	0	NO_SP	1.000042	0.000000	0.000000	0.000000	0.000000	4	4	**117225,3333**	**0**	**#JAKO/0!**	**0,00**
B2HLQ7	Uncharacterized protein	4,6	**41,0**	370	0	NO_SP	1.000045	0.000000	0.000000	0.000000	0.000000	4	2	**117114**	**0**	**#JAKO/0!**	**0,00**
A0A2Z5Y997	4HBT domain-containing protein	4,8	**23,3**	219	0	NO_SP	1.000059	0.000001	0.000000	0.000000	0.000000	4	2	**117092**	**0**	**#JAKO/0!**	**0,00**
A0A2Z5YIZ5	Uncharacterized protein	6,5	**8,2**	77	1	NO_SP	1.000064	0.000000	0.000000	0.000000	0.000000	1	2	**116986,3333**	**0**	**#JAKO/0!**	**0,00**
B2HHT8	Conserved transmembrane protein, MmpS5_2	7,1	**11,6**	112	1	SP	0.001438	0.684287	0.312381	0.001304	0.000333	4	1	**116966,6667**	**0**	**#JAKO/0!**	**0,00**
A0A100I9P6	Multidrug resistance protein B	10,1	**107,0**	1015	11	NO_SP	0.999732	0.000266	0.000000	0.000000	0.000000	4	3	**116885**	**0**	**#JAKO/0!**	**0,00**
A0A2Z5YAN2	Putative NAD-dependent oxidoreductase	4,7	**28,0**	264	0	NO_SP	0.977876	0.021883	0.000145	0.000041	0.000022	3	1	**116801**	**0**	**#JAKO/0!**	**0,00**
A0A3E2MZY5	Uncharacterized protein	5,0	**34,1**	306	0	NO_SP	1.000076	0.000001	0.000000	0.000000	0.000000	4	4	**116540,3333**	**0**	**#JAKO/0!**	**0,00**
B2HD95	Dehydrogenase	4,5	**34,4**	341	1	NO_SP	1.000038	0.000002	0.000000	0.000000	0.000000	4	4	**116527**	**0**	**#JAKO/0!**	**0,00**
A0A2Z5YPG9	Serine/threonine-protein kinase RsbT	5,8	**14,6**	136	0	NO_SP	1.000068	0.000001	0.000000	0.000000	0.000000	4	2	**116321,3333**	**0**	**#JAKO/0!**	**0,00**
A0A2Z5Y944	Uncharacterized protein	6,8	**31,2**	297	0	LIPO	0.000000	0.000100	0.999972	0.000000	0.000000	4	3	**116151,6667**	**0**	**#JAKO/0!**	**0,00**
B2HGS8	N-acetyltransferase domain-containing protein	7,5	**23,8**	212	0	NO_SP	1.000066	0.000000	0.000000	0.000000	0.000000	3	3	**116124**	**0**	**#JAKO/0!**	**0,00**
A0A2Z5Y8T2	Iron ABC transporter substrate-binding protein	4,6	**35,1**	330	0	TATLIPO	0.000000	0.000000	0.000128	0.000199	0.999701	3	3	**116111,3333**	**0**	**#JAKO/0!**	**0,00**
B2HEG2	Uncharacterized protein	4,3	**38,1**	362	0	NO_SP	1.000074	0.000000	0.000000	0.000000	0.000000	4	3	**115858,3333**	**0**	**#JAKO/0!**	**0,00**
B2HFS2	Conserved membrane transport protein, MmpS4_1	5,1	**15,2**	143	1	NO_SP	0.861853	0.134324	0.001593	0.000559	0.000288	3	2	**115588,3333**	**0**	**#JAKO/0!**	**0,00**
B2HSH9	Lipase/esterase LipG1	9,2	**34,5**	314	0	NO_SP	0.997377	0.002612	0.000006	0.000003	0.000002	4	3	**115156,3333**	**1464**	**78,64**	**0,00**
A0A124BUX6	Potassium channel protein	7,0	**52,2**	479	3	NO_SP	1.000045	0.000000	0.000000	0.000000	0.000000	4	3	**115125,3333**	**0**	**#JAKO/0!**	**0,00**
A0A2Z5YB78	Uncharacterized protein	9,9	**123,5**	1175	0	NO_SP	0.997169	0.002754	0.000039	0.000019	0.000008	4	5	**114890,3333**	**0**	**#JAKO/0!**	**0,00**
A0A3E2MU53	6-hydroxy-D-nicotine oxidase	5,4	**50,4**	474	0	NO_SP	0.999971	0.000068	0.000006	0.000000	0.000000	4	3	**114687,6667**	**0**	**#JAKO/0!**	**0,00**
B2HHD9	Transcriptional regulatory protein	9,7	**26,0**	244	0	NO_SP	0.991367	0.008303	0.000261	0.000022	0.000010	4	2	**114328**	**0**	**#JAKO/0!**	**0,00**
A0A3E2MQJ1	Putative peptidase	4,9	**36,8**	351	0	NO_SP	1.000041	0.000006	0.000000	0.000000	0.000000	2	1	**114166,6667**	**0**	**#JAKO/0!**	**0,00**
B2HR36	Polyketide synthase, Pks8	4,8	**220,2**	2114	2	NO_SP	0.999579	0.000395	0.000035	0.000003	0.000001	2	3	**113971,3333**	**898**	**126,92**	**0,00**
B2HEW8	HrpA-like helicase	6,9	**145,9**	1313	0	NO_SP	1.000057	0.000000	0.000000	0.000000	0.000000	4	3	**113925,6667**	**0**	**#JAKO/0!**	**0,00**
A0A117DVL3	Cell envelope-associated transcriptional attenuator	6,9	**69,8**	646	1	NO_SP	1.000050	0.000001	0.000000	0.000000	0.000000	4	2	**113832**	**0**	**#JAKO/0!**	**0,00**
B2HQG6	MCE-family protein Mce3A_1	4,9	**51,1**	486	1	NO_SP	1.000069	0.000000	0.000000	0.000000	0.000000	4	3	**113656**	**691**	**164,53**	**0,00**
A0A100HZF1	L-aspartate oxidase	6,3	**51,5**	505	0	NO_SP	0.991317	0.008636	0.000040	0.000014	0.000007	3	2	**113640,6667**	**0**	**#JAKO/0!**	**0,00**
A0A2Z5YH23	Uncharacterized protein	4,7	**13,9**	138	0	SP	0.477200	0.521492	0.000463	0.000415	0.000216	1	2	**113631,6667**	**0**	**#JAKO/0!**	**0,00**
B2HHM7	3-methyl-2-oxobutanoate hydroxymethyltransferase	5,1	**30,9**	297	0	NO_SP	1.000055	0.000000	0.000000	0.000000	0.000000	4	2	**113197,6667**	**0**	**#JAKO/0!**	**0,00**
A0A100IEA2	NADH-quinone oxidoreductase subunit A	6,2	**13,9**	127	3	NO_SP	1.000066	0.000002	0.000000	0.000000	0.000000	4	1	**112936,3333**	**0**	**#JAKO/0!**	**0,00**
A0A2Z5YCT5	Hydrogenobyrinate a,c-diamide synthase	6,7	**46,8**	452	0	NO_SP	1.000044	0.000007	0.000000	0.000000	0.000000	4	4	**112828,6667**	**0**	**#JAKO/0!**	**0,00**
B2HNM5	MCE-family protein Mce5D	5,1	**39,2**	371	1	NO_SP	0.897143	0.096434	0.003808	0.000590	0.000322	4	1	**112195**	**0**	**#JAKO/0!**	**0,00**
A0A2Z5YLR6	Methyltranfer_dom domain-containing protein	6,0	**29,4**	270	0	NO_SP	1.000042	0.000010	0.000000	0.000000	0.000000	4	2	**112041,3333**	**0**	**#JAKO/0!**	**0,00**
B2HHT9	Cytochrome P450 124A1, Cyp124A1	5,0	**48,4**	433	0	NO_SP	0.999990	0.000035	0.000002	0.000000	0.000000	5	2	**111218,3333**	**0**	**#JAKO/0!**	**0,00**
A0A2Z5YG51	LysR_substrate domain-containing protein	4,9	**24,4**	230	0	NO_SP	1.000019	0.000021	0.000000	0.000000	0.000000	6	3	**111189,3333**	**0**	**#JAKO/0!**	**0,00**
A0A2Z5YLS9	Histidine kinase	7,8	**43,9**	406	6	NO_SP	1.000036	0.000001	0.000000	0.000000	0.000000	4	5	**111038,6667**	**0**	**#JAKO/0!**	**0,00**
A0A2Z5YFQ7	Mycobactin synthetase protein B	7,6	**34,9**	316	0	NO_SP	1.000077	0.000000	0.000000	0.000000	0.000000	4	1	**110831**	**2458**	**45,10**	**0,00**

B2HPQ7	Dehydrogenase/decarboxylase protein	5,0	**23,5**	218	0	NO_SP	1.000079	0.000000	0.000000	0.000000	0.000000	3	3	**110588**	**0**	**#JAKO/0!**	**0,00**
A0A2Z5YC34	ABC transporter substrate-binding protein	6,5	**46,9**	421	0	NO_SP	1.000045	0.000001	0.000000	0.000000	0.000000	3	5	**110554,6667**	**0**	**#JAKO/0!**	**0,00**
B2HIJ3	Oxidoreductase	6,2	**32,1**	310	2	NO_SP	0.999973	0.000073	0.000001	0.000000	0.000000	4	2	**110537,6667**	**0**	**#JAKO/0!**	**0,00**
A0A2Z5Y9B5	Thiamine-phosphate synthase	6,9	**23,1**	220	0	NO_SP	1.000023	0.000027	0.000000	0.000000	0.000000	4	4	**110537,3333**	**0**	**#JAKO/0!**	**0,00**
B2HPE0	Conserved membrane protein	10,2	**12,2**	118	2	NO_SP	0.999931	0.000104	0.000015	0.000000	0.000000	4	3	**110506,3333**	**0**	**#JAKO/0!**	**0,00**
B2HLR0	Epoxide hydrolase EphF	5,8	**33,6**	296	0	NO_SP	1.000030	0.000008	0.000000	0.000000	0.000000	4	3	**110136**	**0**	**#JAKO/0!**	**0,00**
B2HND3	Transcription antitermination protein NusB	4,8	**18,6**	173	0	NO_SP	1.000046	0.000000	0.000000	0.000000	0.000000	3	2	**110124,3333**	**0**	**#JAKO/0!**	**0,00**
B2HHE2	Short-chain type dehydrogenase/reductase	5,4	**26,2**	252	0	NO_SP	1.000045	0.000000	0.000000	0.000000	0.000000	3	3	**109945,3333**	**0**	**#JAKO/0!**	**0,00**
A0A3E2MMM0	Uncharacterized protein	9,9	**60,1**	546	9	NO_SP	0.999813	0.000173	0.000006	0.000000	0.000000	4	3	**109926,6667**	**0**	**#JAKO/0!**	**0,00**
B2HLA1	PPE family protein	4,5	**37,1**	387	0	NO_SP	1.000030	0.000026	0.000000	0.000000	0.000000	4	2	**109862,3333**	**0**	**#JAKO/0!**	**0,00**
B2HIR4	Methyltransferase (Methylase)	5,6	**23,7**	219	0	NO_SP	1.000035	0.000015	0.000000	0.000000	0.000000	3	4	**109808,3333**	**0**	**#JAKO/0!**	**0,00**
B2HEE8	Conserved hypothetical oxidoreductase	6,1	**31,9**	289	0	NO_SP	0.999963	0.000068	0.000001	0.000000	0.000000	4	3	**109745**	**0**	**#JAKO/0!**	**0,00**
B2HRX6	Transcriptional regulatory protein (Probably GntR-family)	6,0	**29,3**	270	0	NO_SP	1.000047	0.000000	0.000000	0.000000	0.000000	4	3	**109350**	**0**	**#JAKO/0!**	**0,00**
B2HKB2	Conserved hypothetical membrane protein	7,2	**35,1**	330	6	NO_SP	1.000055	0.000000	0.000000	0.000000	0.000000	4	2	**109259**	**0**	**#JAKO/0!**	**0,00**
B2HDW0	Phosphomannomutase PmmB	5,1	**55,4**	526	0	NO_SP	0.999847	0.000117	0.000029	0.000001	0.000000	4	4	**109191,1333**	**30653**	**3,56**	**0,28**
A0A2Z5YHQ3	Uncharacterized protein	5,2	**30,8**	291	0	NO_SP	1.000038	0.000000	0.000000	0.000000	0.000000	3	2	**108935,3333**	**0**	**#JAKO/0!**	**0,00**
A0A2Z5YH95	Uncharacterized protein	4,4	**15,3**	137	0	NO_SP	1.000058	0.000001	0.000000	0.000000	0.000000	1	1	**108862**	**0**	**#JAKO/0!**	**0,00**
B2HQH1	MCE-family protein Mce3F_1	5,0	**52,1**	485	1	NO_SP	0.999799	0.000111	0.000015	0.000001	0.000001	3	3	**108800**	**0**	**#JAKO/0!**	**0,00**
A0A2Z5YQB2	Uncharacterized protein	6,5	**15,1**	141	0	NO_SP	1.000085	0.000000	0.000000	0.000000	0.000000	1	1	**108721,3333**	**0**	**#JAKO/0!**	**0,00**
A0A2Z5YPJ9	Non-specific serine/threonine protein kinase	8,6	**69,5**	661	1	NO_SP	1.000047	0.000000	0.000000	0.000000	0.000000	1	2	**108662,6667**	**2248**	**48,34**	**0,00**
A0A3E2MVC1	Pseudouridine synthase	7,3	**31,6**	294	0	NO_SP	1.000053	0.000002	0.000000	0.000000	0.000000	4	2	**108329,3333**	**0**	**#JAKO/0!**	**0,00**
B2HQV3	Uncharacterized protein	4,8	**20,6**	183	0	NO_SP	1.000062	0.000000	0.000000	0.000000	0.000000	4	3	**108186,3333**	**0**	**#JAKO/0!**	**0,00**
B2HRB2	Oligopeptide-transport integral membrane protein ABC transporter OppB	11,1	**35,0**	325	6	NO_SP	0.999993	0.000026	0.000001	0.000000	0.000000	4	2	**108174,3333**	**0**	**#JAKO/0!**	**0,00**
A0A100I1A0	Biotin synthesis protein	9,6	**23,2**	208	0	NO_SP	1.000021	0.000002	0.000000	0.000000	0.000000	4	2	**107702**	**0**	**#JAKO/0!**	**0,00**
B2HGW5	Conserved transmembrane protein	11,3	**22,3**	212	4	NO_SP	1.000002	0.000032	0.000000	0.000000	0.000000	3	1	**107629**	**1742**	**61,78**	**0,00**
A0A100IFC7	Thioredoxin ThiX	6,7	**14,7**	138	0	NO_SP	0.701520	0.295058	0.001005	0.001182	0.000536	4	1	**107607,6667**	**0**	**#JAKO/0!**	**0,00**
A0A2Z5YFT7	Uncharacterized protein	4,3	**25,6**	227	0	NO_SP	1.000052	0.000001	0.000000	0.000000	0.000000	1	2	**107344,6667**	**1420**	**75,60**	**0,00**
B2HS84	Uncharacterized protein	5,8	**56,6**	532	0	NO_SP	1.000047	0.000002	0.000000	0.000000	0.000000	4	6	**107088**	**0**	**#JAKO/0!**	**0,00**
B2HM16	Arsenic-transport integral membrane protein ArsA	5,3	**45,7**	429	9	NO_SP	0.999988	0.000000	0.000000	0.000000	0.000000	4	2	**106936,6667**	**117**	**913,31**	**0,00**
B2HE58	Conserved hypothetical membrane protein	4,9	**26,4**	245	1	NO_SP	0.999748	0.000214	0.000015	0.000002	0.000001	4	3	**106927,6667**	**0**	**#JAKO/0!**	**0,00**
B2HI29	Conserved hypothetical dehydratase (MaoC-like)	4,6	**30,5**	289	0	NO_SP	1.000042	0.000001	0.000000	0.000000	0.000000	4	3	**106479,6667**	**0**	**#JAKO/0!**	**0,00**
B2HQT0	Transcriptional regulatory protein	7,1	**53,3**	474	0	NO_SP	1.000071	0.000000	0.000000	0.000000	0.000000	3	2	**106448,6667**	**0**	**#JAKO/0!**	**0,00**
A0A2Z5YIA2	Aminotransferase	7,2	**37,4**	351	0	NO_SP	1.000046	0.000000	0.000000	0.000000	0.000000	3	4	**106443**	**0**	**#JAKO/0!**	**0,00**
B2HGJ8	Conserved hypothetical membrane protein	5,9	**30,9**	288	0	LIPO	0.000000	0.000020	1.000025	0.000000	0.000000	4	2	**106229**	**0**	**#JAKO/0!**	**0,00**
B2HCT2	50S ribosomal protein L24	11,0	**11,7**	107	0	NO_SP	1.000039	0.000000	0.000000	0.000000	0.000000	3	3	**106104**	**3872**	**27,40**	**0,04**
A0A2Z5YA91	Arylsulfatase	4,6	**86,1**	780	0	NO_SP	0.999518	0.000507	0.000026	0.000001	0.000000	4	2	**106069**	**0**	**#JAKO/0!**	**0,00**
B2HD96	Putative S-adenosyl-L-methionine-dependent methyltransferase MMAR_2791	4,5	**33,0**	303	0	NO_SP	0.993259	0.006371	0.000287	0.000033	0.000016	4	2	**105980,3333**	**0**	**#JAKO/0!**	**0,00**
B2HJS2	Hydrogenase maturation factor, HypE	4,5	**38,2**	370	0	NO_SP	1.000061	0.000000	0.000000	0.000000	0.000000	3	2	**105771,3333**	**0**	**#JAKO/0!**	**0,00**
B2HKQ7	Short-chain type dehydrogenase/reductase	9,3	**31,7**	299	0	NO_SP	1.000017	0.000013	0.000000	0.000000	0.000000	3	2	**105734,3333**	**0**	**#JAKO/0!**	**0,00**
A0A117DYS5	Lipoprotein	4,8	**17,6**	169	0	NO_SP	0.993127	0.004614	0.002159	0.000021	0.000011	4	1	**105699**	**0**	**#JAKO/0!**	**0,00**
A0A3E2N0P0	Anti-sigma-K factor RskA	4,6	**24,0**	232	1	NO_SP	1.000020	0.000000	0.000000	0.000000	0.000000	4	2	**105501,6667**	**0**	**#JAKO/0!**	**0,00**
B2HLL8	dTDP-glucose-4,6-dehydratase, RmlB_1	6,8	**36,6**	328	0	NO_SP	1.000084	0.000000	0.000000	0.000000	0.000000	2	2	**105157,3333**	**0**	**#JAKO/0!**	**0,00**
B2HPP4	Conserved metal-dependent hydrolase	5,0	**45,6**	401	0	NO_SP	1.000064	0.000000	0.000000	0.000000	0.000000	6	2	**105035,3333**	**0**	**#JAKO/0!**	**0,00**
A0A3E2N2S8	Uncharacterized protein	10,9	**40,9**	402	11	NO_SP	1.000071	0.000000	0.000000	0.000000	0.000000	1	1	**104450**	**1435**	**72,77**	**0,00**
B2HG46	Short-chain type dehydrogenase/reductase	5,7	**30,0**	283	0	NO_SP	1.000065	0.000000	0.000000	0.000000	0.000000	4	3	**104107,3333**	**0**	**#JAKO/0!**	**0,00**
A0A3E2MXI2	Uncharacterized protein	10,7	**55,8**	514	12	NO_SP	1.000006	0.000036	0.000000	0.000000	0.000000	4	3	**103335,6667**	**0**	**#JAKO/0!**	**0,00**
A0A100I0A9	Low molecular weight antigen MTB12	4,7	**15,8**	160	0	NO_SP	0.890315	0.109230	0.000151	0.000115	0.000079	4	3	**103235**	**0**	**#JAKO/0!**	**0,00**
B2HGD9	NADH-quinone oxidoreductase subunit J	5,7	**27,3**	262	5	NO_SP	1.000033	0.000004	0.000000	0.000000	0.000000	4	2	**103132,3333**	**0**	**#JAKO/0!**	**0,00**
A0A100ID49	Transcriptional regulator, TetR family	5,1	**23,5**	211	0	NO_SP	1.000071	0.000000	0.000000	0.000000	0.000000	4	3	**103105,6667**	**21760**	**4,74**	**0,21**
B2HRJ6	Transmembrane protein	11,7	**9,8**	94	2	NO_SP	0.948584	0.051072	0.000105	0.000034	0.000026	3	1	**102894,6667**	**0**	**#JAKO/0!**	**0,00**
A0A2Z5YKH9	Uncharacterized protein	4,5	**52,9**	469	0	NO_SP	1.000054	0.000002	0.000000	0.000000	0.000000	1	3	**102546**	**0**	**#JAKO/0!**	**0,00**
A0A100HZT7	2-nitropropane dioxygenase	4,7	**31,3**	298	0	NO_SP	0.794383	0.204259	0.000527	0.000273	0.000216	4	3	**102543,3333**	**0**	**#JAKO/0!**	**0,00**
A0A117DWK0	Uncharacterized protein	7,4	**27,9**	258	0	NO_SP	1.000049	0.000000	0.000000	0.000000	0.000000	4	2	**102539,3333**	**0**	**#JAKO/0!**	**0,00**
A0A2Z5Y8S8	KipI antagonist	6,4	**30,7**	289	0	NO_SP	1.000036	0.000006	0.000000	0.000000	0.000000	3	2	**102240**	**1473**	**69,42**	**0,00**
B2HIF1	Regulatory protein	6,5	**21,8**	198	0	NO_SP	1.000062	0.000001	0.000000	0.000000	0.000000	4	2	**102014**	**0**	**#JAKO/0!**	**0,00**
B2HLK6	Conserved transmembrane protein	9,4	**71,2**	657	12	NO_SP	1.000025	0.000000	0.000000	0.000000	0.000000	4	3	**101925**	**2428**	**41,98**	**0,00**
A0A3E2N198	NAD(P)-bd_dom domain-containing protein	5,4	**40,6**	382	0	NO_SP	1.000046	0.000000	0.000000	0.000000	0.000000	3	3	**101720,3333**	**0**	**#JAKO/0!**	**0,00**
A0A2Z5YB80	NUDIX hydrolase	4,8	**19,8**	182	0	NO_SP	1.000047	0.000028	0.000000	0.000000	0.000000	4	2	**101712,3333**	**0**	**#JAKO/0!**	**0,00**
A0A2Z5YNX2	Uncharacterized protein	6,0	**28,8**	273	0	NO_SP	1.000015	0.000030	0.000000	0.000000	0.000000	1	2	**101591,6667**	**0**	**#JAKO/0!**	**0,00**
B2HKS7	SN-glycerol-3-phosphate transport integral membrane protein ABC transporte	10,5	**31,2**	280	6	NO_SP	1.000035	0.000003	0.000000	0.000000	0.000000	4	2	**100752**	**0**	**#JAKO/0!**	**0,00**
A0A3E2MSF0	Adenylate cyclase 2	6,9	**60,7**	565	6	NO_SP	0.999902	0.000101	0.000000	0.000000	0.000000	4	3	**100047,6667**	**0**	**#JAKO/0!**	**0,00**
A0A117DU72	Universal stress protein family	6,0	**27,6**	260	0	NO_SP	1.000053	0.000004	0.000000	0.000000	0.000000	4	3	**99934,66667**	**0**	**#JAKO/0!**	**0,00**
A0A2Z5YEE2	Adenylate cyclase	6,4	**43,2**	398	6	NO_SP	1.000022	0.000014	0.000000	0.000000	0.000000	4	2	**99925,66667**	**0**	**#JAKO/0!**	**0,00**
A0A3E2MPZ0	Uncharacterized protein	4,8	**52,2**	483	0	NO_SP	0.948233	0.051449	0.000184	0.000060	0.000037	3	3	**99898,33333**	**0**	**#JAKO/0!**	**0,00**
A0A2Z5YA97	Uncharacterized protein	4,7	**22,5**	220	0	NO_SP	0.999287	0.000721	0.000004	0.000001	0.000001	4	3	**99809**	**0**	**#JAKO/0!**	**0,00**
A0A2Z5Y7S6	Uncharacterized protein	10,4	**16,1**	138	2	NO_SP	0.999992	0.000038	0.000000	0.000000	0.000000	4	2	**99709,33333**	**0**	**#JAKO/0!**	**0,00**
A0A2Z5YJM4	PPOX class F420-dependent oxidoreductase	6,9	**14,9**	135	0	NO_SP	1.000030	0.000000	0.000000	0.000000	0.000000	3	2	**99648**	**0**	**#JAKO/0!**	**0,00**
A0A2Z5YPF8	FtsK domain-containing protein	4,9	**107,4**	986	2	NO_SP	0.999889	0.000145	0.000000	0.000000	0.000000	1	3	**99645**	**7945**	**12,54**	**0,00**
B2HK32	Transcriptional regulatory protein (Probably Lrp/AsnC-family)	4,8	**18,4**	171	0	NO_SP	1.000046	0.000000	0.000000	0.000000	0.000000	3	2	**99429**	**0**	**#JAKO/0!**	**0,00**
A0A2Z5YM77	Uncharacterized protein	4,7	**60,0**	569	0	NO_SP	1.000048	0.000000	0.000000	0.000000	0.000000	4	3	**99321**	**0**	**#JAKO/0!**	**0,00**
A0A3E2N2L4	Pentalenene oxygenase	10,0	**47,9**	428	0	NO_SP	1.000033	0.000007	0.000001	0.000000	0.000000	4	3	**99033**	**0**	**#JAKO/0!**	**0,00**
A0A2Z5YIF1	Uncharacterized protein	7,4	**14,1**	124	0	NO_SP	1.000048	0.000000	0.000000	0.000000	0.000000	1	3	**98862,66667**	**0**	**#JAKO/0!**	**0,00**
A0A2Z5YIF5	Uncharacterized protein	4,8	**19,7**	198	0	NO_SP	1.000056	0.000000	0.000000	0.000000	0.000000	4	3	**98671,66667**	**0**	**#JAKO/0!**	**0,00**
B2HSM0	Coenzyme PQQ synthesis protein E, PqqE	5,8	**41,9**	391	0	NO_SP	1.000067	0.000001	0.000000	0.000000	0.000000	4	1	**98089**	**0**	**#JAKO/0!**	**0,00**
B2HRB3	Oligopeptide-transport integral membrane protein ABC transporter OppC	10,1	**32,2**	297	6	NO_SP	0.997117	0.000646	0.001572	0.000003	0.000003	3	2	**97739,33333**	**1046**	**93,45**	**0,00**
A0A100IB82	SAM-dependent methyltransferase	8,1	**24,2**	207	0	NO_SP	0.999733	0.000280	0.000006	0.000001	0.000000	4	1	**97544**	**0**	**#JAKO/0!**	**0,00**
B2HT69	Conserved transmembrane protein	4,9	**71,5**	674	0	NO_SP	1.000037	0.000005	0.000000	0.000000	0.000000	4	4	**97500**	**0**	**#JAKO/0!**	**0,00**

B2HLR9	SnoaL-like domain-containing protein	4,1	**14,9**	133	0	NO_SP	1.000084	0.000000	0.000000	0.000000	0.000000	3	2	**97431,23333**	**0**	**#JAKO/0!**	**0,00**
B2HGS7	Conserved hypothetical membrane protein	12,0	**14,6**	134	2	NO_SP	0.999956	0.000050	0.000000	0.000000	0.000000	4	1	**97393,33333**	**3541**	**27,50**	**0,00**
A0A2Z5YL73	Alpha/beta hydrolase	9,8	**30,0**	274	0	NO_SP	1.000048	0.000000	0.000000	0.000000	0.000000	3	4	**97206,33333**	**0**	**#JAKO/0!**	**0,00**
A0A2Z5YMR1	Cytochrome P450	5,2	**45,3**	406	0	NO_SP	1.000067	0.000000	0.000000	0.000000	0.000000	4	3	**96883,33333**	**0**	**#JAKO/0!**	**0,00**
A0A3E2MWJ0	Acetoin dehydrogenase operon transcriptional activator AcoR	6,8	**44,3**	411	0	NO_SP	1.000052	0.000005	0.000000	0.000000	0.000000	4	2	**96786,66667**	**0**	**#JAKO/0!**	**0,00**
B2HK36	Oxidoreductase	9,7	**30,0**	281	0	NO_SP	0.997823	0.001792	0.000393	0.000005	0.000002	4	3	**96691,66667**	**19238**	**5,03**	**0,20**
B2HKC5	Catalase KatE	6,0	**54,7**	482	0	NO_SP	1.000047	0.000000	0.000000	0.000000	0.000000	4	3	**96462,33333**	**0**	**#JAKO/0!**	**0,00**
A0A2Z5Y7N3	Hydrolase	6,5	**23,8**	222	0	NO_SP	1.000062	0.000000	0.000000	0.000000	0.000000	3	1	**96383,33333**	**0**	**#JAKO/0!**	**0,00**
A0A100I196	Metallo-beta-lactamase superfamily protein	7,1	**27,6**	250	0	NO_SP	0.998818	0.001194	0.000017	0.000003	0.000002	4	2	**96293,66667**	**0**	**#JAKO/0!**	**0,00**
A0A3E2MYA1	3-ketoacyl-CoA thiolase	4,8	**42,4**	405	0	NO_SP	1.000053	0.000000	0.000000	0.000000	0.000000	1	1	**96252,1**	**0**	**#JAKO/0!**	**0,00**
A0A2Z5Y929	Glyoxylate reductase	6,3	**34,8**	320	0	NO_SP	1.000041	0.000000	0.000000	0.000000	0.000000	4	3	**96072**	**0**	**#JAKO/0!**	**0,00**
A0A2Z5YKN9	Uncharacterized protein	5,8	**29,3**	261	0	NO_SP	1.000037	0.000000	0.000000	0.000000	0.000000	3	2	**96003,66667**	**0**	**#JAKO/0!**	**0,00**
A0A2Z5YE54	Phosphoribosyl-AMP cyclohydrolase	5,1	**12,1**	111	0	NO_SP	1.000055	0.000000	0.000000	0.000000	0.000000	2	1	**95809**	**0**	**#JAKO/0!**	**0,00**
A0A2Z5YKN7	NAD-dependent dehydratase	6,5	**22,2**	216	0	NO_SP	1.000055	0.000001	0.000000	0.000000	0.000000	1	2	**95767,33333**	**0**	**#JAKO/0!**	**0,00**
B2HF63	Cobalamin biosynthesis protein	5,2	**38,2**	369	0	NO_SP	1.000031	0.000002	0.000000	0.000000	0.000000	4	3	**95719**	**0**	**#JAKO/0!**	**0,00**
B2HEW1	Uncharacterized protein	7,6	**43,3**	387	3	NO_SP	1.000018	0.000000	0.000000	0.000000	0.000000	4	2	**95716,33333**	**0**	**#JAKO/0!**	**0,00**
B2HJK8	D-amino acid aminohydrolase	6,9	**65,3**	597	0	NO_SP	1.000071	0.000001	0.000000	0.000000	0.000000	4	4	**95694**	**0**	**#JAKO/0!**	**0,00**
A0A2Z5Y8E9	S-adenosyl-L-methionine-dependent methyltransferase	4,4	**34,3**	310	0	NO_SP	1.000052	0.000002	0.000000	0.000000	0.000000	4	3	**94723,66667**	**0**	**#JAKO/0!**	**0,00**
A0A2Z5YND3	Uncharacterized protein	4,7	**39,8**	384	0	NO_SP	0.999945	0.000100	0.000001	0.000000	0.000000	3	3	**94616,66667**	**0**	**#JAKO/0!**	**0,00**
A0A117DWP7	Integral membrane protein	6,7	**28,0**	268	5	NO_SP	0.998578	0.001374	0.000012	0.000008	0.000006	4	1	**94490,66667**	**0**	**#JAKO/0!**	**0,00**
B2HLL0	O-antigen/lipopolysaccharide transport integral membrane protein ABC trans	10,1	**31,6**	276	6	NO_SP	0.999796	0.000188	0.000000	0.000000	0.000000	5	2	**94311,33333**	**8206**	**11,49**	**0,09**
B2HLY2	Uncharacterized protein	4,5	**24,6**	228	0	NO_SP	1.000063	0.000000	0.000000	0.000000	0.000000	3	2	**94092,66667**	**0**	**#JAKO/0!**	**0,00**
A0A100HZR8	Lipoprotein	3,9	**10,1**	92	0	NO_SP	1.000070	0.000001	0.000000	0.000000	0.000000	4	1	**94018,33333**	**0**	**#JAKO/0!**	**0,00**
A0A3E2MWY2	NADH dehydrogenase	5,5	**33,0**	314	0	NO_SP	1.000014	0.000006	0.000000	0.000000	0.000000	4	3	**93844**	**0**	**#JAKO/0!**	**0,00**
B2HGE1	NADH-quinone oxidoreductase subunit H	10,2	**46,5**	424	9	NO_SP	1.000097	0.000000	0.000000	0.000000	0.000000	4	1	**93840,66667**	**2420**	**38,77**	**0,00**
B2HKG8	HTH tetR-type domain-containing protein	7,3	**19,7**	183	0	NO_SP	1.000060	0.000000	0.000000	0.000000	0.000000	4	2	**93655,66667**	**0**	**#JAKO/0!**	**0,00**
B2HI58	Cell division protein CrgA	11,9	**10,4**	93	2	NO_SP	1.000012	0.000014	0.000000	0.000000	0.000000	3	1	**93565**	**11730**	**7,98**	**0,13**
A0A2Z5YK02	Membrane protein	10,3	**20,0**	176	2	NO_SP	1.000009	0.000030	0.000000	0.000000	0.000000	1	1	**93063**	**1246**	**74,71**	**0,00**
A0A2Z5YDD1	Putative N-acetyltransferase	7,2	**16,6**	149	0	NO_SP	1.000052	0.000000	0.000000	0.000000	0.000000	4	3	**92898**	**0**	**#JAKO/0!**	**0,00**
A0A100IHC6	2-nitropropane dioxygenase	4,9	**40,2**	375	0	NO_SP	1.000027	0.000000	0.000000	0.000000	0.000000	3	3	**92872,66667**	**0**	**#JAKO/0!**	**0,00**
A0A3E2MZ46	Non-specific serine/threonine protein kinase	8,3	**67,7**	636	0	NO_SP	1.000075	0.000000	0.000000	0.000000	0.000000	3	2	**92689,33333**	**0**	**#JAKO/0!**	**0,00**
A0A2Z5YID3	Uncharacterized protein	3,9	**15,3**	140	0	NO_SP	1.000079	0.000008	0.000000	0.000000	0.000000	3	3	**92614**	**0**	**#JAKO/0!**	**0,00**
B2HRC2	Lipoprotein LprB	5,2	**19,5**	185	0	LIPO	0.000000	0.000044	0.999993	0.000000	0.000000	2	1	**92596,66667**	**0**	**#JAKO/0!**	**0,00**
B2HQ67	Lipoprotein signal peptidase	7,0	**20,9**	196	3	NO_SP	1.000005	0.000012	0.000000	0.000000	0.000000	1	1	**91840**	**0**	**#JAKO/0!**	**0,00**
A0A2Z5YGV3	Phospho-N-acetylmuramoyl-pentapeptide-transferase	8,0	**37,2**	355	10	NO_SP	1.000043	0.000000	0.000000	0.000000	0.000000	4	1	**91498**	**0**	**#JAKO/0!**	**0,00**
A0A3E2MU16	Tyrosine--tRNA ligase	5,6	**46,7**	430	0	NO_SP	1.000078	0.000000	0.000000	0.000000	0.000000	2	1	**91326,33333**	**0**	**#JAKO/0!**	**0,00**
A0A2Z5YDY9	Putative lipoprotein LprH	5,7	**21,0**	198	0	NO_SP	0.999560	0.000455	0.000010	0.000001	0.000001	4	1	**90958,33333**	**0**	**#JAKO/0!**	**0,00**
A0A3E2N367	Phenolphthiocerol synthesis polyketide synthase type I Pks15/1	6,8	**91,7**	858	0	NO_SP	0.990716	0.009198	0.000087	0.000013	0.000008	5	3	**90613,66667**	**0**	**#JAKO/0!**	**0,00**
A0A3E2MZS2	ATP-dependent DNA helicase RecG	5,9	**80,8**	743	0	NO_SP	1.000040	0.000000	0.000000	0.000000	0.000000	4	2	**90595**	**0**	**#JAKO/0!**	**0,00**
A0A117E0W3	Ethanolamine permease	6,8	**37,7**	359	9	NO_SP	1.000005	0.000022	0.000000	0.000000	0.000000	4	1	**90356,66667**	**820**	**110,20**	**0,00**
B2HNG3	Conserved integral membrane transport protein	6,7	**55,8**	524	14	NO_SP	1.000018	0.000000	0.000000	0.000000	0.000000	3	1	**89811**	**0**	**#JAKO/0!**	**0,00**
A0A2Z5YPH6	Hydrolase_4 domain-containing protein	11,3	**44,8**	408	0	NO_SP	1.000060	0.000002	0.000000	0.000000	0.000000	3	3	**89648,33333**	**0**	**#JAKO/0!**	**0,00**
A0A2Z5YPG0	Uncharacterized protein	8,0	**31,1**	278	2	NO_SP	1.000025	0.000020	0.000000	0.000000	0.000000	1	2	**89565**	**0**	**#JAKO/0!**	**0,00**
A0A100I5A2	Methyltransferase domain protein	4,8	**51,1**	460	0	NO_SP	1.000088	0.000001	0.000000	0.000000	0.000000	4	3	**89517,66667**	**0**	**#JAKO/0!**	**0,00**
B2HSA3	Conserved hypothetical membrane protein	7,0	**100,5**	939	0	NO_SP	1.000069	0.000000	0.000000	0.000000	0.000000	4	4	**88868,66667**	**4451**	**19,97**	**0,05**
B2HSW0	Membrane-bound C-5 sterol desaturase Erg3	10,0	**35,0**	302	4	SP	0.469447	0.518683	0.003986	0.001634	0.001632	4	2	**88405,66667**	**0**	**#JAKO/0!**	**0,00**
B2HST2	4-alpha-glucanotransferase	5,3	**80,4**	723	0	NO_SP	1.000052	0.000000	0.000000	0.000000	0.000000	4	2	**88167**	**0**	**#JAKO/0!**	**0,00**
B2HRP7	Conserved hypothetical membrane protein	10,6	**19,2**	174	2	NO_SP	0.999897	0.000145	0.000000	0.000000	0.000000	3	2	**88059,33333**	**0**	**#JAKO/0!**	**0,00**
A0A124BVT7	Uncharacterized protein	6,4	**35,2**	323	0	NO_SP	1.000053	0.000000	0.000000	0.000000	0.000000	4	3	**88016,33333**	**0**	**#JAKO/0!**	**0,00**
A0A2Z5YN02	Transposase	6,3	**14,8**	132	0	NO_SP	1.000048	0.000000	0.000000	0.000000	0.000000	1	2	**87731**	**0**	**#JAKO/0!**	**0,00**
A0A2Z5YAP0	MDMPI_N domain-containing protein	6,4	**19,8**	187	0	NO_SP	1.000061	0.000001	0.000000	0.000000	0.000000	3	1	**87385**	**0**	**#JAKO/0!**	**0,00**
A0A100I8R9	Uncharacterized protein	4,4	**4,5**	42	0	NO_SP	0.999993	0.000001	0.000000	0.000000	0.000000	3	2	**86723**	**2217**	**39,12**	**0,00**
A0A2Z5YE29	Membrane protein	10,6	**64,3**	605	12	NO_SP	0.972614	0.026702	0.000272	0.000217	0.000097	1	1	**86668,33333**	**815**	**106,41**	**0,00**
A0A100I563	Hydrogenase-4 component B	8,1	**67,6**	654	14	NO_SP	0.994591	0.005217	0.000087	0.000055	0.000021	3	2	**86656,33333**	**0**	**#JAKO/0!**	**0,00**
A0A3E2MWR8	S2P endopeptidase	7,1	**42,7**	403	4	NO_SP	0.861193	0.134056	0.002826	0.000405	0.000310	4	2	**86514**	**0**	**#JAKO/0!**	**0,00**
A0A2Z5YLJ6	Uncharacterized protein	4,5	**10,1**	95	2	NO_SP	1.000021	0.000001	0.000000	0.000000	0.000000	1	1	**85924**	**0**	**#JAKO/0!**	**0,00**
B2HR31	Arginine repressor	4,9	**16,9**	166	0	NO_SP	1.000039	0.000000	0.000000	0.000000	0.000000	3	2	**85889**	**0**	**#JAKO/0!**	**0,00**
B2HFA8	Molybdopterin biosynthesis Mog protein	4,4	**16,5**	161	0	NO_SP	1.000045	0.000010	0.000000	0.000000	0.000000	3	2	**85394,66667**	**0**	**#JAKO/0!**	**0,00**
A0A100I9E1	Isomerase	4,8	**37,8**	349	0	NO_SP	1.000053	0.000008	0.000000	0.000000	0.000000	4	3	**85212**	**0**	**#JAKO/0!**	**0,00**
B2HD97	D-aminoacyl-tRNA deacylase	6,2	**15,1**	143	0	NO_SP	1.000050	0.000000	0.000000	0.000000	0.000000	4	1	**84887,33333**	**0**	**#JAKO/0!**	**0,00**
B2HJN5	Transcriptional regulatory protein	10,0	**14,8**	139	0	NO_SP	1.000042	0.000000	0.000000	0.000000	0.000000	4	2	**84827,66667**	**0**	**#JAKO/0!**	**0,00**
B2HDI3	Uncharacterized protein	5,0	**17,3**	163	0	NO_SP	1.000049	0.000000	0.000000	0.000000	0.000000	4	2	**84643**	**0**	**#JAKO/0!**	**0,00**
A0A117DXQ6	Valine--tRNA ligase	4,7	**98,4**	886	0	NO_SP	1.000055	0.000002	0.000000	0.000000	0.000000	1	1	**84618,3**	**0**	**#JAKO/0!**	**0,00**
A0A2Z5YC36	ACT domain-containing protein	4,9	**23,5**	220	0	NO_SP	1.000021	0.000023	0.000000	0.000000	0.000000	3	2	**84230**	**0**	**#JAKO/0!**	**0,00**
B2HM90	Polyketide synthase MbtC	6,1	**45,4**	439	0	NO_SP	0.999541	0.000501	0.000010	0.000001	0.000000	4	3	**84209,66667**	**0**	**#JAKO/0!**	**0,00**
B2HT29	Conserved protein	4,2	**12,3**	109	0	NO_SP	1.000050	0.000000	0.000000	0.000000	0.000000	4	2	**83741,33333**	**0**	**#JAKO/0!**	**0,00**
A0A2Z5YN97	Lipid II isoglutaminyl synthase (glutamine-hydrolyzing) subunit MurT	5,2	**40,5**	384	0	NO_SP	1.000046	0.000001	0.000000	0.000000	0.000000	4	3	**83729**	**0**	**#JAKO/0!**	**0,00**
A0A3E2MT42	Phytoene synthase	6,8	**35,5**	319	0	NO_SP	1.000051	0.000007	0.000000	0.000000	0.000000	16	4	**83642,66667**	**175**	**477,12**	**0,00**
A0A2Z5YMI3	HTH-type transcriptional repressor KstR	9,3	**24,3**	221	0	NO_SP	1.000033	0.000005	0.000000	0.000000	0.000000	3	2	**83462**	**0**	**#JAKO/0!**	**0,00**
A0A124BWF9	Gamma-aminobutyraldehyde dehydrogenase	4,9	**51,8**	492	0	NO_SP	0.999564	0.000410	0.000045	0.000001	0.000000	4	2	**82710,66667**	**0**	**#JAKO/0!**	**0,00**
A0A2Z5YJT0	Dehydratase	4,9	**42,7**	409	0	NO_SP	0.999734	0.000288	0.000003	0.000001	0.000000	4	3	**82507**	**0**	**#JAKO/0!**	**0,00**
B2HME7	Uncharacterized protein	7,9	**52,8**	479	0	NO_SP	1.000062	0.000000	0.000000	0.000000	0.000000	4	3	**82498,66667**	**0**	**#JAKO/0!**	**0,00**
B2HJY1	VOC domain-containing protein	5,1	**13,6**	123	0	NO_SP	1.000051	0.000000	0.000000	0.000000	0.000000	3	1	**82269,66667**	**0**	**#JAKO/0!**	**0,00**
A0A124BWF0	Conserved hypothetical membrane protein	5,1	**10,1**	92	0	SP	0.449506	0.549557	0.000243	0.000258	0.000212	4	2	**82245,33333**	**0**	**#JAKO/0!**	**0,00**
A0A2Z5YG92	Amidohydro_3 domain-containing protein	6,4	**55,4**	534	0	NO_SP	1.000062	0.000002	0.000000	0.000000	0.000000	4	3	**81752,33333**	**0**	**#JAKO/0!**	**0,00**
B2HNX5	Trans-aconitate 2-methyltransferase	6,8	**29,4**	263	0	NO_SP	1.000059	0.000000	0.000000	0.000000	0.000000	4	2	**81538,33333**	**0**	**#JAKO/0!**	**0,00**
B2HI06	Enoyl-CoA hydratase EchA19	6,5	**29,5**	274	0	NO_SP	1.000020	0.000000	0.000000	0.000000	0.000000	4	3	**81305,33333**	**0**	**#JAKO/0!**	**0,00**

D5MTP6	Phosphatidylinositol phosphate synthase	10,4	**24,6**	232	3	NO_SP	0.999506	0.000487	0.000001	0.000000	0.000000	5	1	**81045**	**0**	**#JAKO/0!**	**0,00**
A0A2Z5YNI0	Glycine betaine/carnitine/choline transport ATP-binding protein OpuCA	6,2	**38,7**	362	0	NO_SP	1.000041	0.000000	0.000000	0.000000	0.000000	3	2	**80916,66667**	**0**	**#JAKO/0!**	**0,00**
B2HQN9	Transcriptional regulatory protein (GntR-family)	5,2	**25,0**	223	0	NO_SP	1.000056	0.000000	0.000000	0.000000	0.000000	4	2	**80873,33333**	**0**	**#JAKO/0!**	**0,00**
A0A100ICZ0	Polyketide cyclase	4,4	**12,6**	115	0	NO_SP	1.000056	0.000001	0.000000	0.000000	0.000000	3	2	**80733,33333**	**0**	**#JAKO/0!**	**0,00**
A0A100IH20	EsaT-6 like protein	5,0	**9,8**	87	0	NO_SP	1.000048	0.000000	0.000000	0.000000	0.000000	8	1	**80510,66667**	**2354**	**34,20**	**0,00**
B2HL01	Conserved hypothetical membrane protein	11,7	**29,2**	271	2	NO_SP	0.997993	0.000839	0.000568	0.000002	0.000001	4	2	**80501**	**0**	**#JAKO/0!**	**0,00**
B2HPR0	PE-PGRS family protein	4,5	**12,7**	125	0	NO_SP	0.999980	0.000071	0.000000	0.000000	0.000000	2	1	**80456,33333**	**0**	**#JAKO/0!**	**0,00**
A0A117DUA4	Cyclic pyranopterin monophosphate synthase	5,9	**14,7**	141	0	NO_SP	1.000031	0.000001	0.000000	0.000000	0.000000	3	1	**80309,66667**	**0**	**#JAKO/0!**	**0,00**
A0A3E2MTD9	Glutamyl-tRNA(Gln) amidotransferase subunit A	6,4	**50,0**	469	0	NO_SP	1.000064	0.000000	0.000000	0.000000	0.000000	4	3	**80211**	**0**	**#JAKO/0!**	**0,00**
B2HR78	Uncharacterized protein	5,7	**50,6**	459	0	NO_SP	0.999155	0.000860	0.000010	0.000002	0.000001	4	2	**79959**	**0**	**#JAKO/0!**	**0,00**
B2HKH4	Glycine dehydrogenase (decarboxylating)	6,3	**102,9**	961	0	NO_SP	1.000059	0.000000	0.000000	0.000000	0.000000	4	3	**79893,33333**	**0**	**#JAKO/0!**	**0,00**
A0A100IDN8	Peroxidase	5,6	**28,5**	262	0	NO_SP	1.000071	0.000000	0.000000	0.000000	0.000000	4	1	**79351,33333**	**0**	**#JAKO/0!**	**0,00**
B2HK14	Uncharacterized protein	6,6	**28,1**	260	1	NO_SP	0.999999	0.000009	0.000000	0.000000	0.000000	4	2	**79272,33333**	**0**	**#JAKO/0!**	**0,00**
B2HE68	Cutinase	4,9	**23,9**	231	1	SP	0.104649	0.891725	0.000604	0.001968	0.000586	3	1	**79192**	**0**	**#JAKO/0!**	**0,00**
B2HI43	Enoyl-CoA hydratase EchA20	5,3	**26,4**	247	0	NO_SP	0.999655	0.000401	0.000004	0.000001	0.000000	4	2	**79065,66667**	**0**	**#JAKO/0!**	**0,00**
A0A2Z5YHG1	Phosphofructokinase	6,7	**33,6**	323	0	NO_SP	0.998621	0.001359	0.000029	0.000004	0.000002	4	3	**78948,33333**	**0**	**#JAKO/0!**	**0,00**
B2HK04	ABM domain-containing protein	4,3	**10,8**	97	0	NO_SP	1.000064	0.000003	0.000000	0.000000	0.000000	4	1	**78786,66667**	**5847**	**13,47**	**0,07**
B2HGI5	Uncharacterized protein	7,5	**15,7**	138	0	NO_SP	1.000054	0.000000	0.000000	0.000000	0.000000	4	1	**78757,33333**	**0**	**#JAKO/0!**	**0,00**
B2HKY2	Acyl-CoA dehydrogenase FadE34_1	6,5	**43,2**	397	0	NO_SP	1.000045	0.000000	0.000000	0.000000	0.000000	4	3	**78730,33333**	**0**	**#JAKO/0!**	**0,00**
A0A100I0C3	Acetyl hydrolase	7,3	**29,8**	282	0	NO_SP	0.999883	0.000133	0.000009	0.000000	0.000000	4	2	**78640,33333**	**0**	**#JAKO/0!**	**0,00**
A0A2Z5YIG2	Phospholipase C	6,8	**56,5**	520	0	TAT	0.000000	0.000000	0.000000	0.999982	0.000011	3	3	**78555**	**0**	**#JAKO/0!**	**0,00**
A0A2Z5YBX5	Oxidoreductase	4,2	**20,7**	187	0	NO_SP	0.999964	0.000042	0.000015	0.000000	0.000000	4	2	**78326**	**0**	**#JAKO/0!**	**0,00**
A0A3E2MMN9	Uncharacterized protein	4,6	**37,4**	369	0	NO_SP	1.000047	0.000009	0.000000	0.000000	0.000000	2	1	**78263,33333**	**0**	**#JAKO/0!**	**0,00**
A0A100I922	Transcriptional regulator MraZ	6,0	**16,0**	143	0	NO_SP	1.000072	0.000000	0.000000	0.000000	0.000000	3	2	**77971,33333**	**0**	**#JAKO/0!**	**0,00**
A0A2Z5YH70	Uncharacterized protein	6,5	**30,0**	278	0	LIPO	0.000000	0.000002	1.000072	0.000000	0.000000	3	2	**77918**	**0**	**#JAKO/0!**	**0,00**
A0A2Z5YG74	Precorrin-6Y C(5,15)-methyltransferase [decarboxylating]	6,9	**41,5**	390	0	NO_SP	1.000024	0.000020	0.000000	0.000000	0.000000	3	3	**77799,33333**	**0**	**#JAKO/0!**	**0,00**
A0A3E2N211	Chromosome partition protein Smc	6,6	**41,9**	385	0	NO_SP	1.000054	0.000000	0.000000	0.000000	0.000000	4	3	**77625,66667**	**4059**	**19,13**	**0,05**
A0A3E2MYQ6	MDMPI_N domain-containing protein	4,5	**19,0**	182	0	NO_SP	1.000068	0.000001	0.000000	0.000000	0.000000	1	2	**77536,66667**	**0**	**#JAKO/0!**	**0,00**
B2HCZ5	Coenzyme F420-dependent oxidoreductase	8,6	**37,5**	347	0	NO_SP	1.000077	0.000000	0.000000	0.000000	0.000000	4	2	**77493**	**0**	**#JAKO/0!**	**0,00**
A0A2Z5YG77	Uncharacterized protein	4,2	**15,2**	147	0	NO_SP	1.000041	0.000000	0.000000	0.000000	0.000000	4	1	**77421**	**0**	**#JAKO/0!**	**0,00**
B2HNB9	Uncharacterized protein	6,2	**27,8**	250	0	NO_SP	1.000029	0.000014	0.000000	0.000000	0.000000	3	2	**77361**	**0**	**#JAKO/0!**	**0,00**
A0A100HZF7	NUDIX hydrolase	10,4	**19,4**	176	0	NO_SP	1.000054	0.000001	0.000000	0.000000	0.000000	4	1	**76966,33333**	**2974**	**25,88**	**0,00**
B2HGD5	NADH-quinone oxidoreductase subunit N	9,2	**55,2**	528	13	NO_SP	0.999957	0.000000	0.000000	0.000000	0.000000	4	1	**76696,66667**	**0**	**#JAKO/0!**	**0,00**
A0A2Z5YLP1	NUDIX hydrolase	4,6	**15,4**	141	0	NO_SP	1.000060	0.000000	0.000000	0.000000	0.000000	1	1	**76469,66667**	**0**	**#JAKO/0!**	**0,00**
B2HM17	Conserved protein	6,7	**15,8**	147	0	NO_SP	1.000039	0.000000	0.000000	0.000000	0.000000	3	1	**76460**	**0**	**#JAKO/0!**	**0,00**
B2HLF7	Uncharacterized protein	4,3	**13,9**	128	0	NO_SP	1.000027	0.000022	0.000000	0.000000	0.000000	3	3	**76299,33333**	**0**	**#JAKO/0!**	**0,00**
A0A2Z5YK12	Non-specific serine/threonine protein kinase	9,8	**47,7**	438	0	NO_SP	1.000041	0.000000	0.000000	0.000000	0.000000	4	2	**75691,33333**	**0**	**#JAKO/0!**	**0,00**
B2HL14	Conserved protein	4,4	**23,2**	231	0	NO_SP	0.923382	0.076091	0.000274	0.000108	0.000061	3	2	**75607,4**	**0**	**#JAKO/0!**	**0,00**
B2HMJ5	Conserved hypothetical membrane protein	8,5	**16,5**	155	4	NO_SP	0.992667	0.007348	0.000000	0.000000	0.000000	3	1	**75551,66667**	**0**	**#JAKO/0!**	**0,00**
A0A2Z5YIB0	Lysine N-acyltransferase MbtK	6,1	**21,6**	188	0	NO_SP	1.000071	0.000000	0.000000	0.000000	0.000000	4	2	**75382,33333**	**0**	**#JAKO/0!**	**0,00**
Q6QAG3	65 kDa heat shock protein (Fragment)	4,2	**13,9**	133	0	NO_SP	1.000057	0.000000	0.000000	0.000000	0.000000	3	1	**75353,33333**	**0**	**#JAKO/0!**	**0,00**
A0A3E2MP41	Stearoyl-CoA 9-desaturase	6,6	**42,9**	377	3	NO_SP	1.000044	0.000001	0.000000	0.000000	0.000000	1	1	**75316,33333**	**0**	**#JAKO/0!**	**0,00**
A0A2Z5YDM4	Putative dihydrodipicolinate synthetase DapA	5,2	**31,6**	296	0	NO_SP	1.000031	0.000001	0.000000	0.000000	0.000000	3	2	**75236,33333**	**0**	**#JAKO/0!**	**0,00**
A0A3E2MWR2	tRNA-dihydrouridine synthase	6,3	**38,6**	361	0	NO_SP	1.000047	0.000000	0.000000	0.000000	0.000000	4	2	**75141,33333**	**0**	**#JAKO/0!**	**0,00**
A0A2Z5Y8M1	Putative quercetin 2,3-dioxygenase	6,5	**28,1**	257	0	NO_SP	1.000077	0.000000	0.000000	0.000000	0.000000	1	3	**74909,33333**	**0**	**#JAKO/0!**	**0,00**
B2HEB7	Enoyl-CoA hydratase EchA7	5,1	**26,5**	257	0	NO_SP	1.000068	0.000000	0.000000	0.000000	0.000000	3	1	**74873**	**0**	**#JAKO/0!**	**0,00**
A0A3E2MZ04	S-(Hydroxymethyl)mycothiol dehydrogenase	4,6	**37,9**	361	0	NO_SP	1.000057	0.000000	0.000000	0.000000	0.000000	1	1	**74869,33333**	**0**	**#JAKO/0!**	**0,00**
A0A3E2MV05	DUF2384 domain-containing protein	7,0	**12,8**	120	0	NO_SP	1.000061	0.000001	0.000000	0.000000	0.000000	2	2	**74723,66667**	**0**	**#JAKO/0!**	**0,00**
A0A100I0V1	Transmembrane protein	11,3	**60,2**	602	8	NO_SP	0.999490	0.000502	0.000005	0.000002	0.000002	4	2	**74605,66667**	**0**	**#JAKO/0!**	**0,00**
B2HQM8	Two-component sensor and regulator	5,3	**136,4**	1287	0	NO_SP	0.999998	0.000051	0.000001	0.000000	0.000000	5	4	**74397**	**2169**	**34,29**	**0,00**
B2HPV4	Magnesium transporter MgtE	4,4	**49,4**	462	5	NO_SP	1.000031	0.000001	0.000000	0.000000	0.000000	4	2	**74180**	**0**	**#JAKO/0!**	**0,00**
B2HIX2	Conserved hypothetical regulatory protein	5,0	**20,3**	181	0	NO_SP	1.000090	0.000000	0.000000	0.000000	0.000000	3	1	**74172,66667**	**0**	**#JAKO/0!**	**0,00**
B2HPK2	Conserved hypothetical phage protein	5,0	**21,0**	188	0	NO_SP	1.000056	0.000000	0.000000	0.000000	0.000000	3	4	**73299,33333**	**0**	**#JAKO/0!**	**0,00**
A0A124BVG8	Adenylate cyclase	4,0	**14,6**	139	0	NO_SP	1.000073	0.000000	0.000000	0.000000	0.000000	4	1	**73189,66667**	**0**	**#JAKO/0!**	**0,00**
B2HLP5	Conserved hypothetical transmembrane protein	6,7	**24,0**	226	6	NO_SP	1.000021	0.000001	0.000000	0.000000	0.000000	3	1	**73188,66667**	**0**	**#JAKO/0!**	**0,00**
A0A2Z5YML5	Uncharacterized protein	5,6	**6,7**	60	0	NO_SP	1.000029	0.000000	0.000000	0.000000	0.000000	1	1	**72965,33333**	**0**	**#JAKO/0!**	**0,00**
A0A2Z5YMF1	PE domain-containing protein	6,5	**26,0**	267	0	NO_SP	0.999446	0.000593	0.000004	0.000001	0.000000	1	1	**72786**	**0**	**#JAKO/0!**	**0,00**
A0A100IA48	Conserved exported protein of uncharacterized function	4,6	**5,4**	52	0	NO_SP	1.000014	0.000003	0.000001	0.000000	0.000000	3	2	**72546,66667**	**0**	**#JAKO/0!**	**0,00**
A0A3E2MQ42	UPF0434 protein DAVIS_04722	4,6	**8,7**	77	0	NO_SP	1.000032	0.000000	0.000000	0.000000	0.000000	4	3	**72327,66667**	**0**	**#JAKO/0!**	**0,00**
A0A117DTZ1	Cyclase	5,3	**17,7**	158	0	NO_SP	1.000074	0.000000	0.000000	0.000000	0.000000	4	2	**72191,03333**	**0**	**#JAKO/0!**	**0,00**
B2HNX4	Conserved hypothetical membrane protein	6,9	**44,7**	406	0	NO_SP	1.000063	0.000001	0.000000	0.000000	0.000000	4	3	**71992,66667**	**0**	**#JAKO/0!**	**0,00**
A0A3E2MV83	2-aminoglycoside phosphotransferase	6,7	**67,1**	620	0	NO_SP	0.999943	0.000084	0.000011	0.000000	0.000000	4	4	**71684**	**0**	**#JAKO/0!**	**0,00**
A0A3E2N063	Bile acid 7-alpha dehydratase	5,6	**18,2**	162	0	NO_SP	1.000067	0.000000	0.000000	0.000000	0.000000	4	3	**71453,33333**	**0**	**#JAKO/0!**	**0,00**
B2HFB7	Membrane-associated oxidoreductase	6,9	**43,9**	403	0	NO_SP	1.000050	0.000005	0.000000	0.000000	0.000000	4	3	**71330,33333**	**0**	**#JAKO/0!**	**0,00**
B2HFK6	Uncharacterized protein	5,0	**41,8**	374	0	NO_SP	1.000074	0.000000	0.000000	0.000000	0.000000	4	2	**71184,33333**	**104**	**685,26**	**0,00**
A0A2Z5YPL1	Alcohol dehydrogenase	5,0	**33,6**	322	2	NO_SP	1.000065	0.000000	0.000000	0.000000	0.000000	3	2	**71146**	**0**	**#JAKO/0!**	**0,00**
B2HH31	SCP_3 domain-containing protein	8,1	**13,7**	129	0	NO_SP	1.000049	0.000003	0.000000	0.000000	0.000000	3	2	**70743,66667**	**0**	**#JAKO/0!**	**0,00**
A0A2Z5YQ81	Putative transcriptional regulator, TetR family protein	10,1	**21,0**	191	0	NO_SP	1.000053	0.000000	0.000000	0.000000	0.000000	1	2	**70649,66667**	**0**	**#JAKO/0!**	**0,00**
B2HMZ3	Uncharacterized protein	4,7	**10,2**	98	0	NO_SP	1.000015	0.000008	0.000000	0.000000	0.000000	5	4	**70195**	**0**	**#JAKO/0!**	**0,00**
A0A3E2N3E3	Energy-coupling factor transporter transmembrane protein EcfT	11,9	**30,1**	286	4	NO_SP	0.999864	0.000133	0.000000	0.000000	0.000000	4	2	**69959,33333**	**226**	**309,60**	**0,00**
B2HDL3	Dolichyl-phosphate-mannose--protein mannosyltransferase	9,5	**57,2**	518	9	NO_SP	0.999956	0.000045	0.000000	0.000000	0.000000	4	2	**69742**	**0**	**#JAKO/0!**	**0,00**
A0A2Z5YA68	SGNH_hydro domain-containing protein	5,6	**24,8**	234	1	SP	0.000273	0.999124	0.000146	0.000151	0.000142	4	2	**69621,33333**	**0**	**#JAKO/0!**	**0,00**
A0A2Z5YBQ9	Uncharacterized protein	6,4	**39,1**	369	0	NO_SP	1.000054	0.000000	0.000000	0.000000	0.000000	3	3	**69583**	**0**	**#JAKO/0!**	**0,00**
B2HKA8	DUF4129 domain-containing protein	6,0	**23,4**	216	1	NO_SP	0.999523	0.000483	0.000001	0.000000	0.000000	4	2	**69511,33333**	**0**	**#JAKO/0!**	**0,00**
A0A2Z5YEU9	Serine/threonine-protein kinase PknK	5,4	**120,0**	1109	0	NO_SP	1.000038	0.000001	0.000000	0.000000	0.000000	4	4	**69362**	**0**	**#JAKO/0!**	**0,00**
B2HL61	Ornithine--oxo-acid aminotransferase	4,6	**43,4**	414	0	NO_SP	1.000059	0.000001	0.000000	0.000000	0.000000	4	3	**69162,33333**	**0**	**#JAKO/0!**	**0,00**
B2HIZ0	Acyl-CoA dehydrogenase FadE30	4,9	**42,2**	382	0	NO_SP	1.000056	0.000001	0.000000	0.000000	0.000000	4	3	**69048**	**0**	**#JAKO/0!**	**0,00**

A0A3E2MRZ9	Zinc uptake regulation protein	7,0	**15,2**	137	0	NO_SP	1.000054	0.000000	0.000000	0.000000	0.000000	4	2	**68555**	**0**	**#JAKO/0!**	**0,00**
A0A100I007	Cutinase	6,6	**22,4**	224	0	NO_SP	1.000031	0.000005	0.000000	0.000000	0.000000	4	2	**68301,66667**	**0**	**#JAKO/0!**	**0,00**
B2HH41	Uncharacterized protein	4,4	**35,9**	335	0	NO_SP	0.999976	0.000068	0.000000	0.000000	0.000000	4	1	**67605**	**0**	**#JAKO/0!**	**0,00**
A0A117DXC9	AP endonuclease, family protein 2	6,8	**29,0**	267	0	NO_SP	1.000042	0.000000	0.000000	0.000000	0.000000	4	1	**67439,33333**	**0**	**#JAKO/0!**	**0,00**
A0A3E2N1M8	Cytochrome P450 130	6,2	**42,4**	387	0	NO_SP	1.000044	0.000000	0.000000	0.000000	0.000000	4	3	**67397,33333**	**0**	**#JAKO/0!**	**0,00**
A0A2Z5YNF2	3-beta hydroxysteroid dehydrogenase	6,6	**39,2**	359	0	NO_SP	0.996684	0.003278	0.000056	0.000009	0.000004	3	2	**67187**	**0**	**#JAKO/0!**	**0,00**
B2HQA4	Imidazoleglycerol-phosphate dehydratase	6,6	**22,9**	210	0	NO_SP	1.000047	0.000001	0.000000	0.000000	0.000000	3	2	**66946,33333**	**0**	**#JAKO/0!**	**0,00**
A0A124BV37	Formate hydrogenlyase subunit 7	6,4	**15,5**	152	0	NO_SP	1.000043	0.000009	0.000000	0.000000	0.000000	4	2	**66608,66667**	**0**	**#JAKO/0!**	**0,00**
A0A2Z5YEW5	Uncharacterized protein	4,3	**12,9**	124	0	NO_SP	0.996180	0.003638	0.000119	0.000024	0.000009	2	1	**66458**	**0**	**#JAKO/0!**	**0,00**
B2HK12	Uncharacterized protein	10,6	**48,5**	443	0	NO_SP	1.000063	0.000000	0.000000	0.000000	0.000000	4	3	**66434**	**0**	**#JAKO/0!**	**0,00**
A0A3E2N1P2	Uncharacterized protein	5,0	**20,9**	189	0	NO_SP	0.999593	0.000465	0.000002	0.000001	0.000000	4	1	**66423,66667**	**0**	**#JAKO/0!**	**0,00**
B2HR37	Polyketide synthase Pks9	4,8	**107,4**	1029	0	NO_SP	0.982447	0.013893	0.003390	0.000080	0.000040	4	2	**66388**	**0**	**#JAKO/0!**	**0,00**
A0A100I9F5	Inositol-1-phosphate synthase	5,1	**34,5**	333	0	NO_SP	0.629567	0.332156	0.036895	0.000405	0.000483	4	2	**66298,66667**	**0**	**#JAKO/0!**	**0,00**
B2HFI9	Arylsulfatase AtsD_1	4,9	**84,0**	767	0	NO_SP	0.999879	0.000128	0.000008	0.000001	0.000000	4	1	**66224,66667**	**0**	**#JAKO/0!**	**0,00**
A0A2Z5YG62	ApeA_NTD1 domain-containing protein	6,5	**51,1**	463	0	NO_SP	1.000044	0.000000	0.000000	0.000000	0.000000	1	3	**66019,33333**	**0**	**#JAKO/0!**	**0,00**
A0A2Z5Y9Q0	Putative transport protein MmpL1	6,4	**102,9**	939	12	NO_SP	0.761867	0.237234	0.000320	0.000159	0.000140	5	2	**65525,33333**	**0**	**#JAKO/0!**	**0,00**
A0A117DTI7	Short chain dehydrogenase	4,6	**17,8**	167	0	NO_SP	1.000036	0.000001	0.000000	0.000000	0.000000	4	1	**65479**	**0**	**#JAKO/0!**	**0,00**
A0A2Z5YEG1	Non-specific serine/threonine protein kinase	7,1	**59,9**	560	1	NO_SP	1.000062	0.000000	0.000000	0.000000	0.000000	3	2	**65438**	**0**	**#JAKO/0!**	**0,00**
B2HGB2	Amidohydro-rel domain-containing protein	4,6	**43,9**	404	0	NO_SP	0.999920	0.000135	0.000001	0.000000	0.000000	4	2	**65413,33333**	**0**	**#JAKO/0!**	**0,00**
B2HJU4	Probable malate:quinone oxidoreductase	10,0	**53,7**	493	0	NO_SP	1.000039	0.000005	0.000000	0.000000	0.000000	4	4	**65335,33333**	**189**	**346,13**	**0,00**
B2HPC6	Transcriptional regulatory protein	6,9	**21,4**	187	0	NO_SP	1.000069	0.000000	0.000000	0.000000	0.000000	3	2	**65303**	**134**	**487,64**	**0,00**
A0A3E2MQG6	YhhN-like protein	8,7	**27,9**	261	8	NO_SP	0.999993	0.000055	0.000000	0.000000	0.000000	1	1	**65265**	**0**	**#JAKO/0!**	**0,00**
B2HD47	Probable phosphoketolase	6,3	**89,2**	800	0	NO_SP	1.000057	0.000004	0.000000	0.000000	0.000000	3	4	**65256**	**0**	**#JAKO/0!**	**0,00**
B2HL66	HTH marR-type domain-containing protein	9,9	**16,9**	158	0	NO_SP	1.000049	0.000001	0.000000	0.000000	0.000000	3	2	**65228,66667**	**0**	**#JAKO/0!**	**0,00**
A0A2Z5Y8K0	DUF2786 domain-containing protein	10,4	**26,8**	249	0	NO_SP	1.000032	0.000011	0.000000	0.000000	0.000000	3	2	**65223,33333**	**0**	**#JAKO/0!**	**0,00**
B2HPE3	Two-component regulator-receiver domain	6,1	**27,3**	247	0	NO_SP	1.000040	0.000001	0.000000	0.000000	0.000000	4	3	**64930,33333**	**0**	**#JAKO/0!**	**0,00**
A0A124BUB4	RNA 3-terminal phosphate cyclase	4,6	**20,3**	181	0	NO_SP	1.000058	0.000000	0.000000	0.000000	0.000000	4	1	**64831,66667**	**157**	**414,05**	**0,00**
A0A100I0Z8	Transporter	8,6	**50,1**	485	11	SP	0.164424	0.831196	0.001053	0.001200	0.001102	4	2	**64721**	**0**	**#JAKO/0!**	**0,00**
A0A2Z5YP94	FHA domain-containing protein	9,8	**12,4**	115	0	NO_SP	1.000003	0.000022	0.000000	0.000000	0.000000	1	1	**64554**	**0**	**#JAKO/0!**	**0,00**
B2HMC4	PE-PGRS family protein, PE_PGRS2	3,4	**46,5**	534	0	NO_SP	1.000024	0.000010	0.000000	0.000000	0.000000	4	1	**64343**	**0**	**#JAKO/0!**	**0,00**
B2HML3	Acyltransferase	11,3	**27,5**	251	0	NO_SP	1.000060	0.000000	0.000000	0.000000	0.000000	4	2	**64021,33333**	**0**	**#JAKO/0!**	**0,00**
A0A100IES0	Multidrug ABC transporter permease	11,9	**24,6**	236	6	NO_SP	1.000030	0.000001	0.000000	0.000000	0.000000	4	1	**63973,66667**	**0**	**#JAKO/0!**	**0,00**
B2HSD1	Uncharacterized protein	6,6	**23,4**	212	0	NO_SP	1.000063	0.000000	0.000000	0.000000	0.000000	3	2	**63877,66667**	**0**	**#JAKO/0!**	**0,00**
B2HID0	Beta-lactamase	6,3	**42,4**	402	0	SP	0.003384	0.995573	0.000286	0.000290	0.000221	3	3	**63720,66667**	**0**	**#JAKO/0!**	**0,00**
A0A117DVT8	3-hydroxyacyl-thioester dehydratase HtdY	4,9	**30,5**	290	0	NO_SP	0.999723	0.000331	0.000001	0.000001	0.000000	3	1	**63575,66667**	**9298**	**6,84**	**0,15**
A0A2Z5YNI1	Aminopeptidase N	7,3	**48,5**	435	0	NO_SP	1.000068	0.000000	0.000000	0.000000	0.000000	4	2	**63465**	**0**	**#JAKO/0!**	**0,00**
A0A117DWL8	Membrane protein	10,1	**30,7**	274	2	NO_SP	1.000064	0.000001	0.000000	0.000000	0.000000	3	1	**63230**	**0**	**#JAKO/0!**	**0,00**
A0A2Z5YAV0	Carrier domain-containing protein	6,3	**113,1**	1081	0	NO_SP	0.999258	0.000684	0.000040	0.000005	0.000002	1	1	**63118**	**0**	**#JAKO/0!**	**0,00**
B2HHJ4	Conserved hypothetical MbtH-like protein	4,3	**8,7**	76	0	NO_SP	1.000052	0.000000	0.000000	0.000000	0.000000	1	1	**62967**	**0**	**#JAKO/0!**	**0,00**
A0A2Z5Y8L7	Uncharacterized protein	5,7	**14,2**	133	2	NO_SP	1.000005	0.000047	0.000000	0.000000	0.000000	1	2	**62952**	**0**	**#JAKO/0!**	**0,00**
B2HSC7	Uncharacterized protein	7,0	**18,7**	171	0	NO_SP	1.000044	0.000004	0.000000	0.000000	0.000000	4	1	**62602**	**0**	**#JAKO/0!**	**0,00**
B2HH77	NAD-dependent aldehyde dehydrogenase, AldA	4,6	**51,0**	489	0	NO_SP	1.000037	0.000004	0.000000	0.000000	0.000000	4	2	**62593**	**0**	**#JAKO/0!**	**0,00**
A0A117DWA8	Ribosomal protein	4,0	**16,9**	160	0	NO_SP	1.000063	0.000001	0.000000	0.000000	0.000000	4	2	**62423**	**0**	**#JAKO/0!**	**0,00**
A0A3E2MMT5	Magnesium transport protein CorA	4,7	**36,5**	322	2	NO_SP	1.000043	0.000000	0.000000	0.000000	0.000000	4	2	**62386,33333**	**0**	**#JAKO/0!**	**0,00**
A0A2Z5YGA3	RES domain protein	6,9	**21,3**	184	0	NO_SP	0.976574	0.023329	0.000059	0.000022	0.000020	1	2	**62193,33333**	**0**	**#JAKO/0!**	**0,00**
A0A2Z5YL06	Uncharacterized protein	4,1	**40,6**	405	0	NO_SP	0.999855	0.000202	0.000001	0.000000	0.000000	2	1	**62121,33333**	**0**	**#JAKO/0!**	**0,00**
A0A100I379	Copper resistance protein D	8,6	**69,9**	651	15	NO_SP	1.000022	0.000003	0.000000	0.000000	0.000000	4	2	**62027,66667**	**0**	**#JAKO/0!**	**0,00**
B2HGM4	Conserved hypothetical NADP-dependent oxidoreductase	5,2	**35,4**	335	0	NO_SP	1.000062	0.000000	0.000000	0.000000	0.000000	3	3	**61964,17**	**0**	**#JAKO/0!**	**0,00**
B2HH51	Conserved hypothetical regulatory protein	6,3	**31,9**	304	0	NO_SP	0.982579	0.011459	0.005852	0.000035	0.000024	4	2	**61899**	**0**	**#JAKO/0!**	**0,00**
A0A3E2MZY9	Succinate-semialdehyde dehydrogenase [NADP(+)] 1	4,5	**48,6**	458	0	NO_SP	1.000006	0.000040	0.000000	0.000000	0.000000	3	1	**61793**	**0**	**#JAKO/0!**	**0,00**
A0A2Z5YJ29	Dihydrolipoamide acetyltransferase component of pyruvate dehydrogenase c	5,0	**41,0**	389	0	NO_SP	1.000076	0.000000	0.000000	0.000000	0.000000	4	2	**61745,66667**	**0**	**#JAKO/0!**	**0,00**
A0A100IGM9	Acyl-CoA dehydrogenase	5,6	**34,9**	320	0	NO_SP	1.000062	0.000000	0.000000	0.000000	0.000000	3	2	**61742,33333**	**0**	**#JAKO/0!**	**0,00**
A0A100I9M3	DNA helicase	5,1	**111,6**	1054	0	NO_SP	1.000009	0.000018	0.000000	0.000000	0.000000	3	3	**61733**	**70844**	**0,87**	**1,15**
B2HSF7	RecBCD enzyme subunit RecC	6,6	**121,2**	1099	0	NO_SP	1.000020	0.000013	0.000001	0.000000	0.000000	4	2	**61539,66667**	**0**	**#JAKO/0!**	**0,00**
A0A2Z5YCS3	Uncharacterized protein	6,5	**15,5**	136	2	NO_SP	1.000017	0.000015	0.000000	0.000000	0.000000	1	1	**61510,33333**	**0**	**#JAKO/0!**	**0,00**
B2HL63	Transcriptional regulatory protein (Probably AsnC-family)	5,5	**16,7**	150	0	NO_SP	1.000053	0.000000	0.000000	0.000000	0.000000	3	1	**61418**	**0**	**#JAKO/0!**	**0,00**
A0A100I680	Alternative RNA polymerase sigma-D factor, SigD	6,7	**14,8**	137	0	NO_SP	1.000023	0.000015	0.000001	0.000000	0.000000	3	1	**61340,66667**	**0**	**#JAKO/0!**	**0,00**
A0A100IGD0	Hydrolase	6,5	**57,5**	520	0	NO_SP	1.000049	0.000000	0.000000	0.000000	0.000000	4	3	**61319**	**444121**	**0,14**	**7,24**
A0A2Z5YI49	Uncharacterized protein	5,0	**16,4**	149	0	NO_SP	1.000055	0.000000	0.000000	0.000000	0.000000	4	1	**61270,66667**	**673**	**91,03**	**0,00**
A0A2Z5YMA6	Sodium-independent anion transporter	6,5	**51,5**	490	10	NO_SP	1.000019	0.000001	0.000000	0.000000	0.000000	1	1	**61022,33333**	**0**	**#JAKO/0!**	**0,00**
B2HM48	HTH tetR-type domain-containing protein	6,7	**22,2**	201	0	NO_SP	1.000062	0.000000	0.000000	0.000000	0.000000	4	2	**60863**	**0**	**#JAKO/0!**	**0,00**
A0A2Z5YN24	HTH tetR-type domain-containing protein	6,1	**20,8**	187	0	NO_SP	1.000019	0.000006	0.000000	0.000000	0.000000	4	4	**60848,33333**	**0**	**#JAKO/0!**	**0,00**
A0A2Z5Y9A5	Putative 8-oxo-dGTP diphosphatase 3	5,3	**22,7**	207	0	NO_SP	1.000067	0.000000	0.000000	0.000000	0.000000	3	3	**60644,66667**	**13813**	**4,39**	**0,00**
B2HQJ1	Glyoxalase, GloA_1	5,2	**17,2**	155	0	NO_SP	1.000056	0.000003	0.000000	0.000000	0.000000	3	1	**60476,33333**	**0**	**#JAKO/0!**	**0,00**
B2HT91	Glutamine amidotransferase type-2 domain-containing protein	7,0	**32,1**	290	0	NO_SP	0.983910	0.015875	0.000172	0.000027	0.000014	4	2	**60384,33333**	**0**	**#JAKO/0!**	**0,00**
A0A3E2MY72	Uncharacterized protein	4,7	**20,1**	193	1	LIPO	0.082460	0.123230	0.793591	0.000174	0.000179	4	2	**60327,66667**	**0**	**#JAKO/0!**	**0,00**
A0A3E2N0U9	Phenolphthiocerol synthesis polyketide synthase type I Pks15/1	6,8	**63,7**	607	0	NO_SP	1.000046	0.000001	0.000000	0.000000	0.000000	2	3	**60287**	**0**	**#JAKO/0!**	**0,00**
A0A3E2N246	HTH-type transcriptional repressor KstR2	6,5	**22,2**	206	0	NO_SP	1.000036	0.000010	0.000000	0.000000	0.000000	4	2	**60123**	**0**	**#JAKO/0!**	**0,00**
A0A2Z5YC02	Formate hydrogenase HycE	6,7	**53,8**	498	0	NO_SP	1.000063	0.000002	0.000000	0.000000	0.000000	4	3	**60026**	**0**	**#JAKO/0!**	**0,00**
B2HKY6	Dihydrofolate reductase	6,6	**18,1**	165	0	NO_SP	1.000053	0.000002	0.000000	0.000000	0.000000	3	1	**59924**	**0**	**#JAKO/0!**	**0,00**
A0A100I7E7	Daunorubicin resistance transmembrane protein	10,0	**17,4**	162	4	NO_SP	1.000034	0.000000	0.000000	0.000000	0.000000	4	1	**59608,66667**	**0**	**#JAKO/0!**	**0,00**
B2HF30	Sugar-transport ATP-binding protein ABC transporter	6,2	**39,2**	356	0	NO_SP	1.000058	0.000000	0.000000	0.000000	0.000000	4	2	**59486,33333**	**0**	**#JAKO/0!**	**0,00**
B2HI35	Acyl-CoA dehydrogenase FadE28	5,0	**35,6**	343	0	NO_SP	1.000059	0.000002	0.000000	0.000000	0.000000	4	1	**59289,66667**	**0**	**#JAKO/0!**	**0,00**
A0A2Z5YH09	Uncharacterized protein	6,9	**8,7**	82	0	NO_SP	1.000049	0.000001	0.000000	0.000000	0.000000	3	1	**59204,66667**	**0**	**#JAKO/0!**	**0,00**
A0A2Z5YM94	PBP_domain domain-containing protein	4,3	**32,8**	319	0	NO_SP	1.000080	0.000001	0.000000	0.000000	0.000000	3	1	**59172,66667**	**0**	**#JAKO/0!**	**0,00**
A0A100I945	TetR family transcriptional regulator	5,0	**18,5**	170	0	NO_SP	1.000053	0.000001	0.000000	0.000000	0.000000	4	2	**59131,33333**	**0**	**#JAKO/0!**	**0,00**
A0A2Z5YH45	Uncharacterized protein	4,4	**25,9**	310	0	NO_SP	0.999561	0.000461	0.000000	0.000000	0.000000	2	1	**59090,33333**	**0**	**#JAKO/0!**	**0,00**

A0A2Z5Y7Q8	Uncharacterized protein	5,5	38,6	358	0	NO_SP	1.000048	0.000000	0.000000	0.000000	0.000000	4	3	59035,16667	0	#JAKO/0!	0,00
A0A124BVP6	MCE-family protein Mce4A	5,4	41,6	388	0	SP	0.000400	0.998910	0.000178	0.000178	0.000159	3	2	58892,66667	0	#JAKO/0!	0,00
A0A124BU64	Malto-oligosyltrehalose synthase	4,9	76,4	700	0	NO_SP	1.000063	0.000000	0.000000	0.000000	0.000000	4	3	58015	0	#JAKO/0!	0,00
B2HL59	Uncharacterized protein	6,0	31,6	290	0	NO_SP	1.000044	0.000000	0.000000	0.000000	0.000000	3	1	57900,93333	0	#JAKO/0!	0,00
B2HRR7	ABC transporter, ATP-binding protein	6,9	25,8	244	0	NO_SP	1.000053	0.000001	0.000000	0.000000	0.000000	4	2	57772	0	#JAKO/0!	0,00
A0A2Z5YEB5	16S/23S rRNA (Cytidine-2-O)-methyltransferase TlyA	7,4	29,1	281	0	NO_SP	1.000046	0.000002	0.000000	0.000000	0.000000	1	1	57524,66667	0	#JAKO/0!	0,00
A0A2Z5Y955	Putative phosphoesterase, PA-phosphatase related protein	10,9	52,9	498	0	NO_SP	1.000065	0.000001	0.000000	0.000000	0.000000	3	2	57394	0	#JAKO/0!	0,00
A0A100I778	GCN5 family acetyltransferase	6,9	28,5	269	0	NO_SP	1.000009	0.000026	0.000008	0.000000	0.000000	3	1	57255,33333	0	#JAKO/0!	0,00
A0A2Z5Y961	LysR family transcriptional regulator	7,9	32,4	307	0	NO_SP	1.000055	0.000000	0.000000	0.000000	0.000000	4	3	57033,33333	0	#JAKO/0!	0,00
B2HRH7	F420-dependent glucose-6-phosphate dehydrogenase Fgd2	6,9	41,4	381	0	NO_SP	0.999639	0.000378	0.000015	0.000001	0.000000	4	2	56940	0	#JAKO/0!	0,00
A0A2Z5YNJ4	Bacterial proteasome activator	4,2	19,3	177	0	NO_SP	1.000056	0.000001	0.000000	0.000000	0.000000	1	1	56645	0	#JAKO/0!	0,00
B2HRM5	1,4-dihydroxy-2-naphthoate octaprenyltransferase	10,3	29,6	291	7	NO_SP	0.998891	0.001136	0.000000	0.000000	0.000000	4	1	56456	0	#JAKO/0!	0,00
A0A3E2MPL4	Tetratricopeptide repeat protein	5,1	60,4	555	0	NO_SP	0.999445	0.000527	0.000044	0.000002	0.000001	4	2	56368,5	0	#JAKO/0!	0,00
B2HSC0	Conserved transmembrane protein	10,7	46,1	422	9	NO_SP	0.999966	0.000041	0.000000	0.000000	0.000000	4	1	56267,33333	0	#JAKO/0!	0,00
A0A2Z5YH47	Mycobactin synthetase protein B	6,5	57,1	527	0	NO_SP	1.000043	0.000004	0.000000	0.000000	0.000000	1	1	56258,33333	0	#JAKO/0!	0,00
A0A2Z5YDH6	Transcriptional regulator	7,3	24,0	223	0	NO_SP	1.000049	0.000002	0.000000	0.000000	0.000000	4	2	55783	0	#JAKO/0!	0,00
B2HLD6	Uncharacterized protein	8,8	24,9	219	0	NO_SP	1.000033	0.000001	0.000000	0.000000	0.000000	1	1	55623,66667	0	#JAKO/0!	0,00
A0A2Z5Y7S5	DNA-binding response regulator	7,4	20,4	192	0	NO_SP	1.000051	0.000000	0.000000	0.000000	0.000000	4	2	55591	0	#JAKO/0!	0,00
A0A100I7U7	Uncharacterized protein	4,8	4,9	44	0	NO_SP	1.000002	0.000003	0.000001	0.000000	0.000000	3	1	55586,66667	0	#JAKO/0!	0,00
A0A2Z5Y9N1	Uncharacterized protein	11,7	14,2	148	2	NO_SP	0.972963	0.026758	0.000122	0.000080	0.000037	3	2	55511	7944	6,99	0,14
A0A3E2N0R4	Uncharacterized protein	5,1	10,0	91	0	NO_SP	0.999779	0.000204	0.000006	0.000002	0.000001	4	1	55355,33333	0	#JAKO/0!	0,00
A0A100IF40	Uncharacterized protein	4,4	14,2	137	0	NO_SP	0.999993	0.000059	0.000001	0.000000	0.000000	4	1	55275,33333	0	#JAKO/0!	0,00
A0A2Z5Y998	Nitrilotriacetate monooxygenase component A	5,8	48,8	443	0	NO_SP	1.000044	0.000005	0.000001	0.000000	0.000000	3	4	55037,33333	0	#JAKO/0!	0,00
A0A2Z5YDX2	Heme A synthase	10,6	33,3	318	8	NO_SP	1.000007	0.000014	0.000000	0.000000	0.000000	3	2	54871	0	#JAKO/0!	0,00
A0A100I848	Ribosomal RNA small subunit methyltransferase H	8,1	35,6	328	0	NO_SP	1.000028	0.000002	0.000000	0.000000	0.000000	4	1	54869,66667	4614	11,89	0,08
A0A2Z5YP12	Uncharacterized protein	5,0	79,7	776	0	NO_SP	0.999921	0.000095	0.000015	0.000001	0.000000	1	2	54476,66667	0	#JAKO/0!	0,00
B2HKE1	N-acetylmuramoyl-L-alanine amidase	8,5	24,8	241	0	SP	0.000295	0.999036	0.000153	0.000179	0.000153	4	1	53981	0	#JAKO/0!	0,00
A0A3E2N117	Putative xanthine dehydrogenase subunit A	5,0	40,5	384	0	NO_SP	1.000047	0.000005	0.000000	0.000000	0.000000	4	2	53973,66667	0	#JAKO/0!	0,00
A0A3E2MQ97	Purine catabolism regulatory protein	6,0	46,6	418	0	NO_SP	1.000051	0.000000	0.000000	0.000000	0.000000	4	2	53707,66667	0	#JAKO/0!	0,00
A0A2Z5YKI0	Uncharacterized protein	12,0	21,4	205	3	NO_SP	0.870741	0.128118	0.000624	0.000128	0.000088	4	2	53595,8	0	#JAKO/0!	0,00
B2HHY3	Conserved hypothetical secreted protein	4,9	20,4	193	0	SP	0.000285	0.999037	0.000168	0.000203	0.000145	3	1	52987	0	#JAKO/0!	0,00
B2HFW4	Uncharacterized protein	7,5	11,2	100	0	NO_SP	1.000058	0.000000	0.000000	0.000000	0.000000	3	1	52549	0	#JAKO/0!	0,00
A0A2Z5Y9V5	STAS domain-containing protein	7,0	22,6	207	0	NO_SP	1.000023	0.000039	0.000003	0.000000	0.000000	1	2	52530,33333	0	#JAKO/0!	0,00
A0A2Z5Y954	Oxidoreductase	4,4	31,8	292	0	NO_SP	0.999887	0.000143	0.000001	0.000000	0.000000	4	2	51971,73333	0	#JAKO/0!	0,00
A0A2Z5YME5	Alpha/beta hydrolase family protein	5,6	23,6	233	0	NO_SP	0.998518	0.001430	0.000057	0.000002	0.000001	3	1	51725,66667	0	#JAKO/0!	0,00
B2HK17	Conserved hypothetical membrane protein	8,3	40,3	376	5	NO_SP	1.000053	0.000000	0.000000	0.000000	0.000000	4	1	51634,66667	0	#JAKO/0!	0,00
B2HI59	Conserved hypothetical membrane protein	6,3	26,8	247	1	NO_SP	0.624102	0.358370	0.010978	0.000930	0.000611	2	1	51620,66667	0	#JAKO/0!	0,00
A0A2Z5Y7R1	Glucanase	4,7	34,8	337	1	SP	0.440626	0.555027	0.002574	0.000640	0.000407	3	2	51590,66667	3083	16,74	0,00
B2HMF0	Conserved hypothetical membrane protein	7,5	50,2	464	4	NO_SP	1.000037	0.000000	0.000000	0.000000	0.000000	5	2	51533,66667	0	#JAKO/0!	0,00
B2HHT1	Conserved hypothetical membrane protein	5,9	26,5	249	2	NO_SP	0.999924	0.000079	0.000001	0.000000	0.000000	4	1	51533,33333	0	#JAKO/0!	0,00
A0A2Z5YNV4	tRNA-specific adenosine deaminase	5,8	16,0	152	0	NO_SP	1.000027	0.000006	0.000000	0.000000	0.000000	3	2	51434,66667	0	#JAKO/0!	0,00
B2HLZ4	Uncharacterized protein	6,4	13,4	126	0	NO_SP	1.000058	0.000002	0.000000	0.000000	0.000000	2	1	51387,66667	0	#JAKO/0!	0,00
A0A2Z5YCJ9	Hexuronate transporter	10,3	44,1	415	11	NO_SP	1.000038	0.000000	0.000000	0.000000	0.000000	2	1	51245,66667	0	#JAKO/0!	0,00
A0A117DYU9	Lignin peroxidase	5,5	38,8	362	0	NO_SP	0.998481	0.001552	0.000006	0.000003	0.000002	3	1	51078	0	#JAKO/0!	0,00
A0A100IGV8	Proline dehydrogenase	4,6	31,6	285	0	NO_SP	1.000060	0.000000	0.000000	0.000000	0.000000	3	3	51011,46667	0	#JAKO/0!	0,00
A0A3E2MT84	Uncharacterized protein	7,0	24,7	232	0	NO_SP	0.999774	0.000257	0.000001	0.000001	0.000000	4	2	50993,33333	0	#JAKO/0!	0,00
A0A2Z5YAH1	Bac_luciferase domain-containing protein	8,5	29,5	271	0	NO_SP	0.999945	0.000103	0.000005	0.000000	0.000000	4	2	50903	0	#JAKO/0!	0,00
B2HRU5	Uncharacterized protein	4,4	30,4	275	0	NO_SP	1.000063	0.000000	0.000000	0.000000	0.000000	4	1	50859,33333	0	#JAKO/0!	0,00
A0A3E2N2Z1	Uncharacterized protein	10,0	79,3	711	11	NO_SP	1.000056	0.000002	0.000000	0.000000	0.000000	4	1	50778	0	#JAKO/0!	0,00
A0A2Z5YNS1	DNA ligase (ATP)	6,7	39,2	347	0	NO_SP	1.000040	0.000002	0.000000	0.000000	0.000000	4	2	50632,66667	0	#JAKO/0!	0,00
A0A2Z5YIQ0	TetR family transcriptional regulator	7,5	22,7	211	0	NO_SP	1.000067	0.000000	0.000000	0.000000	0.000000	4	2	50386,66667	0	#JAKO/0!	0,00
B2HH75	Dehydrogenase/reductase	6,0	29,8	288	0	NO_SP	1.000071	0.000000	0.000000	0.000000	0.000000	3	1	50379	0	#JAKO/0!	0,00
A0A2Z5YKG1	Membrane protein	9,4	71,1	673	10	NO_SP	0.977518	0.016562	0.000226	0.000090	0.000065	2	2	50210,66667	792	63,41	0,00
B2HPP0	PPE family protein	3,8	60,9	594	0	NO_SP	1.000072	0.000001	0.000000	0.000000	0.000000	3	1	50094	0	#JAKO/0!	0,00
A0A2Z5YPB6	2-isopropylmalate synthase	4,6	62,0	566	0	NO_SP	1.000054	0.000000	0.000000	0.000000	0.000000	3	1	49806,66667	0	#JAKO/0!	0,00
A0A2Z5Y812	Putative alcohol dehydrogenase D	5,7	40,2	385	0	NO_SP	1.000033	0.000000	0.000000	0.000000	0.000000	2	2	49625	0	#JAKO/0!	0,00
B2HIC3	Uncharacterized protein	5,7	9,4	82	0	NO_SP	1.000063	0.000000	0.000000	0.000000	0.000000	2	1	49573,03333	91381	0,54	1,84
A0A2Z5Y7Q2	Uncharacterized protein	4,6	34,2	330	0	NO_SP	0.999991	0.000071	0.000001	0.000000	0.000000	1	1	49458,33333	0	#JAKO/0!	0,00
A0A100IG23	Alpha/beta hydrolase	6,0	33,2	309	0	NO_SP	1.000074	0.000000	0.000000	0.000000	0.000000	3	1	49328,66667	0	#JAKO/0!	0,00
B2HS80	Keto acyl-CoA thiolase, Ltp2	4,7	41,2	389	0	NO_SP	0.998638	0.001385	0.000005	0.000002	0.000001	4	1	49257,66667	0	#JAKO/0!	0,00
A0A100HZI5	UDP-glycosyltransferase	5,5	40,3	373	0	NO_SP	1.000048	0.000001	0.000000	0.000000	0.000000	4	1	48985,33333	0	#JAKO/0!	0,00
B2HJ66	tRNA(Ile)-lysidine synthase	10,8	33,6	323	0	NO_SP	1.000041	0.000000	0.000000	0.000000	0.000000	3	3	48892	0	#JAKO/0!	0,00
A0A124BX59	ArsR family transcriptional regulator	8,6	6,7	60	0	NO_SP	1.000049	0.000000	0.000000	0.000000	0.000000	4	1	48866	0	#JAKO/0!	0,00
A0A3E2MQJ7	Alanine--tRNA ligase	5,2	96,4	901	0	NO_SP	1.000059	0.000000	0.000000	0.000000	0.000000	2	1	48715,26667	0	#JAKO/0!	0,00
A0A2Z5YEQ4	Uncharacterized protein	10,1	28,0	245	0	NO_SP	1.000058	0.000002	0.000000	0.000000	0.000000	1	2	48654,66667	1695	28,70	0,00
B2HSL0	30S ribosomal protein S12	11,9	13,8	124	0	NO_SP	1.000049	0.000000	0.000000	0.000000	0.000000	3	2	48612,33333	31913	1,52	0,66
A0A2Z5YIL8	WD_REPEATS_REGION domain-containing protein	5,6	45,0	427	0	NO_SP	0.999782	0.000250	0.000002	0.000000	0.000000	1	1	48519	0	#JAKO/0!	0,00
B2HGV0	Uncharacterized protein	4,8	13,2	129	0	NO_SP	1.000072	0.000001	0.000000	0.000000	0.000000	4	1	48345,33333	0	#JAKO/0!	0,00
A0A100HZ54	PE-PGRS family protein	4,3	42,2	404	0	NO_SP	0.999628	0.000425	0.000001	0.000000	0.000000	4	1	48227	0	#JAKO/0!	0,00
B2HRZ7	Non-specific serine/threonine protein kinase	5,8	63,9	608	1	NO_SP	1.000048	0.000000	0.000000	0.000000	0.000000	4	2	48191,33333	0	#JAKO/0!	0,00
A0A3E2MMP9	Uncharacterized protein	4,5	25,3	240	0	NO_SP	0.976481	0.023249	0.000161	0.000055	0.000028	4	2	47785	0	#JAKO/0!	0,00
A0A3E2MN97	Uncharacterized protein	10,1	27,7	242	3	NO_SP	1.000000	0.000012	0.000000	0.000000	0.000000	2	1	47713,33333	0	#JAKO/0!	0,00
B2HQC3	Fatty-acid-CoA ligase FadD19_2	5,6	57,9	547	0	NO_SP	1.000030	0.000001	0.000000	0.000000	0.000000	3	1	47628	0	#JAKO/0!	0,00
A0A117DW63	Benzoylsuccinyl-CoA thiolase	3,8	5,9	53	0	NO_SP	0.999999	0.000005	0.000003	0.000000	0.000000	4	1	47471,33333	0	#JAKO/0!	0,00
B2HEL0	Conserved hypothetical membrane protein	10,1	23,9	214	4	NO_SP	1.000052	0.000000	0.000000	0.000000	0.000000	3	1	47455,66667	0	#JAKO/0!	0,00
A0A2Z5YBP9	DNA ligase	8,1	52,5	501	0	NO_SP	1.000049	0.000001	0.000000	0.000000	0.000000	4	2	47436,33333	0	#JAKO/0!	0,00
A0A2Z5YP47	ABC transporter substrate-binding protein	6,3	49,5	463	2	LIPO	0.015215	0.446469	0.536610	0.000542	0.000832	4	2	47434,66667	0	#JAKO/0!	0,00

B2HQG7	MCE-family protein Mce3B_1	5,2	**36,8**	343	1	NO_SP	0.999976	0.000044	0.000001	0.000000	0.000000	4	1	**47377,66667**	**2850**	**16,62**	**0,06**
A0A124BW91	ATPase	4,4	**12,6**	115	0	NO_SP	0.999674	0.000364	0.000002	0.000001	0.000000	3	3	**47357**	**0**	**#JAKO/0!**	**0,00**
A0A100IBX0	Putative dehydratase	6,1	**13,7**	128	0	NO_SP	1.000058	0.000000	0.000000	0.000000	0.000000	4	2	**46983,96667**	**0**	**#JAKO/0!**	**0,00**
A0A2Z5YHD0	Putative oxidoreductase	7,1	**27,9**	258	0	NO_SP	1.000035	0.000000	0.000000	0.000000	0.000000	4	1	**46852,66667**	**0**	**#JAKO/0!**	**0,00**
B2HI33	Uncharacterized protein	4,5	**34,7**	316	0	NO_SP	1.000047	0.000006	0.000000	0.000000	0.000000	3	1	**46465,33333**	**0**	**#JAKO/0!**	**0,00**
A0A3E2N2D4	DUF3631 domain-containing protein	6,3	**78,8**	726	0	NO_SP	1.000073	0.000000	0.000000	0.000000	0.000000	1	1	**46462,66667**	**207**	**224,32**	**0,00**
A0A100I0J5	Metal dependent hydrolase	4,8	**35,6**	338	0	NO_SP	0.998214	0.001833	0.000003	0.000002	0.000001	4	1	**46360,66667**	**0**	**#JAKO/0!**	**0,00**
B2HN24	Conserved lipoprotein LpqI	4,8	**40,7**	397	0	LIPO	0.000000	0.000000	1.000060	0.000000	0.000000	2	1	**46231,66667**	**0**	**#JAKO/0!**	**0,00**
A0A117DY59	Mycofactocin system glycosyltransferase	9,3	**49,0**	453	0	NO_SP	1.000030	0.000004	0.000000	0.000000	0.000000	4	1	**46132**	**0**	**#JAKO/0!**	**0,00**
A0A2Z5YKJ4	Short-chain dehydrogenase	10,7	**31,0**	297	0	NO_SP	1.000050	0.000000	0.000000	0.000000	0.000000	1	1	**46103**	**1412**	**32,66**	**0,00**
B2HQG2	Transcriptional regulatory protein (Probably Lrp/AsnC-family)	6,8	**17,6**	162	0	NO_SP	1.000076	0.000000	0.000000	0.000000	0.000000	4	1	**45950,78**	**1245**	**36,92**	**0,03**
A0A2Z5YMB7	LLM class F420-dependent oxidoreductase	4,9	**32,3**	293	0	NO_SP	1.000068	0.000000	0.000000	0.000000	0.000000	4	2	**45841,33333**	**0**	**#JAKO/0!**	**0,00**
A0A2Z5YAL9	Transcriptional regulator	6,4	**24,3**	227	0	NO_SP	1.000037	0.000000	0.000000	0.000000	0.000000	1	1	**45789,66667**	**0**	**#JAKO/0!**	**0,00**
A0A2Z5YDW9	Transcriptional regulator	7,0	**24,2**	233	0	NO_SP	1.000062	0.000000	0.000000	0.000000	0.000000	4	2	**45737**	**0**	**#JAKO/0!**	**0,00**
A0A2Z5YMT4	Putative phosphatase	7,1	**30,8**	285	1	NO_SP	0.999895	0.000092	0.000021	0.000001	0.000000	3	1	**45657,33333**	**354**	**129,07**	**0,00**
B2HPC1	Thioredoxin TrxA	6,6	**12,9**	118	0	NO_SP	1.000068	0.000001	0.000000	0.000000	0.000000	3	1	**45645,33333**	**0**	**#JAKO/0!**	**0,00**
B2HP72	UvrABC system protein C	8,9	**72,2**	647	0	NO_SP	1.000061	0.000000	0.000000	0.000000	0.000000	4	2	**45642**	**743**	**61,43**	**0,02**
A0A2Z5YPE6	Putative cytochrome P450 123	6,2	**48,4**	438	0	NO_SP	1.000047	0.000000	0.000000	0.000000	0.000000	1	3	**45545**	**0**	**#JAKO/0!**	**0,00**
A0A2Z5YP46	Acyl-CoA dehydrogenase	4,6	**33,7**	320	0	NO_SP	1.000071	0.000000	0.000000	0.000000	0.000000	4	2	**45534**	**0**	**#JAKO/0!**	**0,00**
B2HIF6	Aspartyl/glutamyl-tRNA(Asn/Gln) amidotransferase subunit C	3,9	**10,6**	99	0	NO_SP	1.000054	0.000000	0.000000	0.000000	0.000000	3	2	**45487**	**0**	**#JAKO/0!**	**0,00**
A0A117DTT1	2,3-dihydroxybenzoate-AMP ligase	5,3	**57,2**	537	0	NO_SP	1.000063	0.000000	0.000000	0.000000	0.000000	3	1	**45429,33333**	**0**	**#JAKO/0!**	**0,00**
A0A117DUL4	Membrane protein	10,6	**28,9**	277	5	NO_SP	0.976978	0.022826	0.000083	0.000042	0.000030	4	1	**45422**	**0**	**#JAKO/0!**	**0,00**
A0A3E2N1U3	Adenine DNA glycosylase	8,0	**32,3**	294	0	NO_SP	1.000066	0.000001	0.000000	0.000000	0.000000	2	2	**45189,33333**	**0**	**#JAKO/0!**	**0,00**
A0A100IF25	Uncharacterized protein	6,5	**6,9**	63	0	NO_SP	1.000073	0.000000	0.000000	0.000000	0.000000	4	3	**44537,66667**	**0**	**#JAKO/0!**	**0,00**
A0A2Z5YPN5	ESX-5 secretion system protein EccA5	5,0	**58,3**	524	0	NO_SP	0.997548	0.002437	0.000042	0.000006	0.000003	1	1	**44320,66667**	**0**	**#JAKO/0!**	**0,00**
B2HGM0	Conserved hypothetical secreted protein	9,6	**42,5**	378	0	TAT	0.000110	0.022052	0.000032	0.977730	0.000058	4	3	**44203,33333**	**0**	**#JAKO/0!**	**0,00**
B2HSD3	Conserved transmembrane transport protein MmpL13	8,5	**81,9**	780	12	NO_SP	0.979138	0.015149	0.000213	0.000084	0.000060	2	1	**44152,66667**	**0**	**#JAKO/0!**	**0,00**
A0A100HZG6	Maltooligosyltrehalose trehalohydrolase, TreZ	4,9	**41,9**	382	0	NO_SP	1.000027	0.000000	0.000000	0.000000	0.000000	4	2	**44017**	**0**	**#JAKO/0!**	**0,00**
A0A100I103	dTDP-glucose 4,6-dehydratase	8,0	**30,8**	286	0	NO_SP	1.000044	0.000001	0.000000	0.000000	0.000000	4	3	**43839,66667**	**0**	**#JAKO/0!**	**0,00**
A0A2Z5YI96	Sensor domain-containing protein	4,8	**26,1**	251	0	LIPO	0.001055	0.182176	0.816441	0.000106	0.000119	4	3	**43586,6**	**0**	**#JAKO/0!**	**0,00**
A0A3E2MXY7	DNA helicase	6,3	**75,8**	699	0	NO_SP	0.999997	0.000041	0.000000	0.000000	0.000000	1	1	**43579**	**0**	**#JAKO/0!**	**0,00**
A0A2Z5Y9P5	DNA-binding protein	10,5	**11,4**	107	0	NO_SP	1.000033	0.000001	0.000000	0.000000	0.000000	4	2	**43170,23333**	**958**	**45,06**	**0,00**
A0A2Z5YIB1	Putative lipoprotein LppP	10,0	**18,1**	167	0	LIPO	0.000000	0.000001	1.000061	0.000000	0.000000	3	2	**43134,73333**	**0**	**#JAKO/0!**	**0,00**
A0A2Z5YFI3	Uncharacterized protein	4,5	**35,5**	339	0	NO_SP	1.000050	0.000012	0.000000	0.000000	0.000000	4	1	**43091,33333**	**0**	**#JAKO/0!**	**0,00**
A0A2Z5YER0	ABC transporter domain-containing protein	8,1	**33,7**	308	0	NO_SP	1.000018	0.000016	0.000001	0.000000	0.000000	1	1	**43067,33333**	**0**	**#JAKO/0!**	**0,00**
A0A124BW69	ATP-binding protein	6,0	**32,4**	308	3	SP	0.000650	0.998629	0.000183	0.000179	0.000159	4	2	**42838,66667**	**0**	**#JAKO/0!**	**0,00**
B2HRV5	Metal cation transporter P-type ATPase	5,2	**79,3**	764	6	NO_SP	1.000028	0.000010	0.000000	0.000000	0.000000	2	3	**42825**	**24246**	**1,77**	**0,57**
B2HIG9	Alanine rich dehydrogenase	7,1	**50,1**	476	0	NO_SP	1.000069	0.000000	0.000000	0.000000	0.000000	4	2	**42788,33333**	**380**	**112,72**	**0,00**
A0A3E2MVD1	Erythromycin 3-O-methyltransferase	5,3	**20,9**	188	0	NO_SP	1.000081	0.000000	0.000000	0.000000	0.000000	4	2	**42602,5**	**0**	**#JAKO/0!**	**0,00**
A0A2Z5Y7Y5	Anti-sigma factor antagonist	4,0	**13,1**	125	0	NO_SP	1.000070	0.000000	0.000000	0.000000	0.000000	1	1	**42593,66667**	**0**	**#JAKO/0!**	**0,00**
A0A2Z5Y8G3	4HBT domain-containing protein	6,9	**15,4**	141	0	NO_SP	0.999766	0.000272	0.000004	0.000000	0.000000	4	2	**42463,33333**	**0**	**#JAKO/0!**	**0,00**
B2HL99	ESAT-6-like protein	4,8	**9,9**	94	0	NO_SP	1.000045	0.000001	0.000000	0.000000	0.000000	2	2	**42455**	**0**	**#JAKO/0!**	**0,00**
A0A100IBI9	Membrane protein	6,2	**21,4**	201	1	NO_SP	0.997736	0.002203	0.000020	0.000001	0.000001	3	1	**42350,33333**	**0**	**#JAKO/0!**	**0,00**
A0A2Z5YHT5	Sensor domain-containing protein	4,6	**21,2**	202	0	LIPO	0.000573	0.355104	0.643738	0.000195	0.000205	2	2	**42339**	**0**	**#JAKO/0!**	**0,00**
A0A3E2MQV1	Uncharacterized protein	6,7	**38,2**	348	0	NO_SP	1.000000	0.000037	0.000004	0.000000	0.000000	1	1	**42313**	**0**	**#JAKO/0!**	**0,00**
A0A3E2MSN8	Glutathionyl-hydroquinone reductase YqjG	6,5	**37,1**	334	0	NO_SP	1.000046	0.000015	0.000001	0.000000	0.000000	4	3	**42127**	**0**	**#JAKO/0!**	**0,00**
B2HIU5	PE family protein	3,7	**56,4**	549	0	NO_SP	0.999312	0.000730	0.000003	0.000001	0.000000	7	1	**42081,66667**	**0**	**#JAKO/0!**	**0,00**
B2HI31	Acetyl-CoA acetyltransferase (PaaJ-like), Ltp2_1	5,4	**41,0**	386	0	NO_SP	1.000029	0.000016	0.000000	0.000000	0.000000	3	1	**42049,66667**	**0**	**#JAKO/0!**	**0,00**
A0A2Z5YAV1	Methyltranfer_dom domain-containing protein	9,9	**14,5**	135	0	NO_SP	1.000069	0.000000	0.000000	0.000000	0.000000	4	2	**41993,66667**	**0**	**#JAKO/0!**	**0,00**
A0A2Z5Y9U2	O-succinylbenzoate synthase	5,4	**26,3**	253	0	NO_SP	1.000034	0.000016	0.000001	0.000000	0.000000	4	1	**41946**	**0**	**#JAKO/0!**	**0,00**
A0A2Z5YGS7	Mycobactin synthetase protein B	4,5	**120,3**	1120	0	NO_SP	1.000039	0.000000	0.000000	0.000000	0.000000	1	1	**41724,33333**	**0**	**#JAKO/0!**	**0,00**
A0A2Z5YLW7	Uncharacterized protein	4,4	**29,3**	278	0	NO_SP	1.000057	0.000000	0.000000	0.000000	0.000000	1	2	**41658**	**0**	**#JAKO/0!**	**0,00**
B2HJ56	Monooxygenase	5,5	**43,7**	396	0	NO_SP	1.000056	0.000001	0.000000	0.000000	0.000000	4	1	**41561,66667**	**0**	**#JAKO/0!**	**0,00**
B2HM59	Diacylglycerol O-acyltransferase	6,4	**51,8**	475	0	NO_SP	1.000046	0.000000	0.000000	0.000000	0.000000	4	3	**41479,66667**	**0**	**#JAKO/0!**	**0,00**
B2HKH2	Glycine cleavage system H protein	3,7	**13,7**	134	0	NO_SP	1.000044	0.000002	0.000000	0.000000	0.000000	4	1	**41403**	**0**	**#JAKO/0!**	**0,00**
B2HNA0	Uncharacterized protein	6,1	**57,2**	527	1	TAT	0.000195	0.000030	0.000012	0.878012	0.121738	4	3	**41292,1**	**0**	**#JAKO/0!**	**0,00**
A0A2Z5YBC2	D-alanyl-D-alanine carboxypeptidase	5,3	**40,5**	391	1	NO_SP	0.959369	0.040366	0.000108	0.000055	0.000039	4	2	**41053,66667**	**0**	**#JAKO/0!**	**0,00**
A0A2Z5YGE5	Uncharacterized protein	7,5	**14,0**	125	0	NO_SP	1.000061	0.000000	0.000000	0.000000	0.000000	3	2	**40789**	**0**	**#JAKO/0!**	**0,00**
B2HME0	Conserved transmembrane transport protein MmpL4_1	6,2	**106,2**	973	12	NO_SP	0.999985	0.000018	0.000000	0.000000	0.000000	4	1	**40544,66667**	**0**	**#JAKO/0!**	**0,00**
A0A2Z5YPY1	Transcriptional regulator	4,9	**28,4**	264	0	NO_SP	1.000051	0.000002	0.000000	0.000000	0.000000	1	1	**40382,66667**	**0**	**#JAKO/0!**	**0,00**
B2HEG1	Short-chain type dehydrogenase/reductase	5,3	**27,0**	263	0	NO_SP	1.000049	0.000001	0.000000	0.000000	0.000000	3	2	**40353**	**0**	**#JAKO/0!**	**0,00**
B2HF93	Transcriptional regulatory protein (Possibly marR-family)	7,0	**10,4**	95	0	NO_SP	1.000071	0.000000	0.000000	0.000000	0.000000	4	2	**40328,33333**	**0**	**#JAKO/0!**	**0,00**
A0A2Z5YGS9	Mycobactin synthetase protein B	4,8	**112,0**	1044	0	NO_SP	1.000069	0.000000	0.000000	0.000000	0.000000	1	1	**40090,66667**	**0**	**#JAKO/0!**	**0,00**
B2HJJ6	Formamidopyrimidine-DNA glycosylase	9,7	**32,0**	292	0	NO_SP	1.000057	0.000002	0.000000	0.000000	0.000000	4	1	**39942**	**0**	**#JAKO/0!**	**0,00**
B2HI10	Uncharacterized protein	4,6	**35,8**	329	0	NO_SP	1.000051	0.000000	0.000000	0.000000	0.000000	4	2	**39846,23333**	**0**	**#JAKO/0!**	**0,00**
A0A100I0X5	Membrane protein	8,6	**55,3**	518	8	NO_SP	1.000019	0.000004	0.000000	0.000000	0.000000	3	1	**39661,33333**	**0**	**#JAKO/0!**	**0,00**
A0A3E2MUJ7	Acetyl-CoA acetyltransferase	4,5	**53,3**	506	0	NO_SP	1.000037	0.000008	0.000000	0.000000	0.000000	4	2	**39656**	**0**	**#JAKO/0!**	**0,00**
A0A100I081	L-seryl-tRNA(Sec) selenium transferase	10,1	**44,4**	428	0	NO_SP	0.999301	0.000738	0.000012	0.000001	0.000001	4	2	**39440,66667**	**0**	**#JAKO/0!**	**0,00**
A0A100HZY9	Glucose dehydrogenase	5,8	**27,4**	257	0	NO_SP	1.000062	0.000000	0.000000	0.000000	0.000000	3	1	**39435,66667**	**0**	**#JAKO/0!**	**0,00**
B2HIY0	PE family protein	3,8	**59,4**	575	0	NO_SP	0.999999	0.000042	0.000000	0.000000	0.000000	2	1	**39426,66667**	**0**	**#JAKO/0!**	**0,00**
A0A2Z5YNH3	Uncharacterized protein	6,4	**5,9**	53	0	NO_SP	1.000017	0.000000	0.000000	0.000000	0.000000	1	1	**39361,8**	**0**	**#JAKO/0!**	**0,00**
B2HHJ9	Mycobactin synthetase protein B	6,4	**481,5**	4431	0	NO_SP	1.000057	0.000000	0.000000	0.000000	0.000000	4	1	**39258,66667**	**0**	**#JAKO/0!**	**0,00**
A0A117DYI2	Putative glycosyltransferase	5,4	**42,2**	392	0	NO_SP	1.000057	0.000000	0.000000	0.000000	0.000000	4	2	**39203**	**1722**	**22,77**	**0,00**
A0A3E2MML9	Uncharacterized protein	10,2	**15,1**	137	0	NO_SP	1.000050	0.000000	0.000000	0.000000	0.000000	4	2	**38685**	**0**	**#JAKO/0!**	**0,00**
A0A100I8S2	Transcriptional regulator PadR-like family	7,5	**10,4**	92	0	NO_SP	1.000069	0.000001	0.000000	0.000000	0.000000	4	1	**38542,66667**	**0**	**#JAKO/0!**	**0,00**
B2HQ04	Uncharacterized protein	4,1	**18,1**	174	1	SP	0.000365	0.998911	0.000172	0.000184	0.000171	3	3	**38484,66667**	**1880**	**20,47**	**0,00**
A0A2Z5YPE1	Integrase	10,6	**38,3**	349	0	NO_SP	0.967333	0.014079	0.010193	0.004030	0.003539	1	2	**38463,33333**	**0**	**#JAKO/0!**	**0,00**

B2HL20	EsaT-6 like protein EsxN_6	6,0	10,0	94	0	NO_SP	1.000049	0.000001	0.000000	0.000000	0.000000	3	2	38110	0	#JAKO/0!	0,00
A0A100I3F0	Transmembrane protein	11,6	21,3	201	4	NO_SP	1.000033	0.000000	0.000000	0.000000	0.000000	4	1	38025,43333	0	#JAKO/0!	0,00
A0A2Z5YM13	Molybdopterin synthase catalytic subunit 2	6,4	14,8	141	0	NO_SP	1.000055	0.000000	0.000000	0.000000	0.000000	4	2	37984,66667	0	#JAKO/0!	0,00
B2HJ08	Conserved hypothetical secreted protein	5,1	19,0	176	0	SP	0.000240	0.999086	0.000180	0.000167	0.000150	2	1	37912,66667	0	#JAKO/0!	0,00
A0A2Z5Y9X5	Putative HTH-type transcriptional regulator	4,7	26,1	245	0	NO_SP	1.000051	0.000001	0.000000	0.000000	0.000000	1	2	37770,33333	0	#JAKO/0!	0,00
A0A117DY44	Invasion protein	10,0	24,7	239	1	SP	0.000405	0.998908	0.000223	0.000166	0.000145	3	1	37471	0	#JAKO/0!	0,00
A0A3E2MZG4	HTH-type transcriptional regulator LutR	8,1	53,2	482	0	NO_SP	1.000041	0.000000	0.000000	0.000000	0.000000	4	1	37378	0	#JAKO/0!	0,00
B2HSW9	Uncharacterized protein	10,3	31,7	306	1	NO_SP	0.999932	0.000053	0.000021	0.000000	0.000000	4	2	37136	0	#JAKO/0!	0,00
A0A100IH66	Betaine aldehyde dehydrogenase	8,6	46,4	430	0	NO_SP	1.000058	0.000000	0.000000	0.000000	0.000000	3	1	37122	0	#JAKO/0!	0,00
B2HEW7	Conserved protein	4,8	17,3	156	0	NO_SP	1.000027	0.000033	0.000000	0.000000	0.000000	3	1	36733,33333	0	#JAKO/0!	0,00
A0A100I0C1	Isochorismate synthase	5,2	47,2	434	0	NO_SP	1.000076	0.000001	0.000000	0.000000	0.000000	3	1	36493,66667	0	#JAKO/0!	0,00
A0A3E2N0J9	Magnesium-protoporphyrin O-methyltransferase	5,0	20,7	194	0	NO_SP	1.000061	0.000001	0.000000	0.000000	0.000000	4	2	36186	0	#JAKO/0!	0,00
A0A2Z5YGH4	Carrier domain-containing protein	7,8	57,9	522	0	NO_SP	1.000050	0.000001	0.000000	0.000000	0.000000	1	1	36039,33333	0	#JAKO/0!	0,00
A0A2Z5YN32	Dihydrodipicolinate reductase	4,5	37,7	358	0	NO_SP	1.000005	0.000040	0.000001	0.000000	0.000000	4	1	35733,66667	0	#JAKO/0!	0,00
A0A124BVF7	Cytochrome d ubiquinol oxidase subunit II	9,6	37,3	341	8	NO_SP	1.000044	0.000000	0.000000	0.000000	0.000000	3	1	35710	0	#JAKO/0!	0,00
B2HF62	Adenylate cyclase	5,1	115,2	1075	0	NO_SP	0.999495	0.000532	0.000001	0.000001	0.000000	3	3	35573,13333	0	#JAKO/0!	0,00
**A0A100I1L1**	**60 kDa GroEL2 chaperonin**	**4,5**	54,2	525	0	NO_SP	1.000048	0.000000	0.000000	0.000000	0.000000	1	1	35563,33333	0	#JAKO/0!	0,00
B2HDJ2	Arsenate reductase	6,5	13,5	122	0	NO_SP	1.000036	0.000000	0.000000	0.000000	0.000000	4	2	35504,66667	0	#JAKO/0!	0,00
A0A2Z5Y8Y8	Uncharacterized protein	6,3	21,5	188	0	NO_SP	1.000071	0.000001	0.000000	0.000000	0.000000	1	2	35431	0	#JAKO/0!	0,00
B2HS60	Glyco_trans_2-like domain-containing protein	4,4	34,7	327	0	NO_SP	1.000014	0.000001	0.000000	0.000000	0.000000	3	1	35350,33333	0	#JAKO/0!	0,00
B2HSK6	Transcriptional regulator	4,9	18,1	166	0	NO_SP	1.000056	0.000001	0.000000	0.000000	0.000000	4	1	35291	0	#JAKO/0!	0,00
A0A124BVT6	Threonine rich protein	4,4	15,0	152	0	LIPO	0.000000	0.000002	1.000043	0.000000	0.000000	3	1	35118,66667	0	#JAKO/0!	0,00
A0A3E2N0V1	Phthiocerol/phenolphthiocerol synthesis polyketide synthase type I PpsA	4,6	58,7	561	0	NO_SP	1.000042	0.000002	0.000000	0.000000	0.000000	2	1	34873,33333	0	#JAKO/0!	0,00
A0A100IA13	50S ribosomal subunit assembly factor BipA	4,9	67,7	628	0	NO_SP	1.000054	0.000000	0.000000	0.000000	0.000000	1	2	34737	0	#JAKO/0!	0,00
B2HJF8	Adenylate cyclase, CyaA	4,5	39,6	374	0	NO_SP	1.000039	0.000000	0.000000	0.000000	0.000000	3	2	34694	0	#JAKO/0!	0,00
A0A2Z5YE33	Adenosylmethionine-8-amino-7-oxononanoate aminotransferase	6,1	45,4	427	0	NO_SP	1.000053	0.000000	0.000000	0.000000	0.000000	4	1	34533,33333	0	#JAKO/0!	0,00
A0A2Z5YN81	Glycine/betaine ABC transporter substrate-binding protein	5,0	33,6	315	0	LIPO	0.000001	0.000266	0.999772	0.000000	0.000000	1	1	34427	0	#JAKO/0!	0,00
A0A2Z5YD78	Putative transporter	5,7	45,3	428	9	NO_SP	1.000022	0.000000	0.000000	0.000000	0.000000	1	1	34271	3287	10,43	0,00
B2HDS5	Methyltransferase	5,1	26,3	244	0	NO_SP	1.000045	0.000002	0.000000	0.000000	0.000000	1	1	34175,33333	0	#JAKO/0!	0,00
A0A2Z5YME2	LAO/AO transport system ATPase	6,1	30,4	292	0	NO_SP	1.000052	0.000000	0.000000	0.000000	0.000000	4	2	34126,33333	0	#JAKO/0!	0,00
A0A2Z5YBV5	NADPH-dependent stearoyl-CoA 9-desaturase	9,7	48,7	428	0	NO_SP	1.000048	0.000006	0.000000	0.000000	0.000000	1	1	33856,33333	0	#JAKO/0!	0,00
A0A100I977	Secreted protein	4,4	5,3	50	0	NO_SP	1.000021	0.000001	0.000000	0.000000	0.000000	4	1	33513,33333	0	#JAKO/0!	0,00
B2HNX9	Conserved protein	6,6	17,4	160	0	NO_SP	1.000064	0.000000	0.000000	0.000000	0.000000	4	1	33477	0	#JAKO/0!	0,00
A0A117DW42	ECF RNA polymerase sigma factor SigK	6,5	19,4	173	0	NO_SP	0.999575	0.000395	0.000034	0.000003	0.000002	4	1	33334,33333	0	#JAKO/0!	0,00
A0A3E2MUA8	ANTAR domain protein	4,5	26,9	251	0	NO_SP	1.000061	0.000000	0.000000	0.000000	0.000000	2	2	33330,66667	0	#JAKO/0!	0,00
A0A2Z5YLQ2	UPF0118 membrane protein	10,5	41,5	385	8	NO_SP	0.999998	0.000000	0.000000	0.000000	0.000000	1	1	33305	576	57,86	0,00
B2HN51	Transcriptional regulator	5,8	44,9	405	0	NO_SP	1.000044	0.000000	0.000000	0.000000	0.000000	4	1	33242	0	#JAKO/0!	0,00
A0A2Z5YH03	Guanylate cyclase domain-containing protein	8,5	36,2	342	0	NO_SP	1.000019	0.000024	0.000000	0.000000	0.000000	1	1	33020,66667	0	#JAKO/0!	0,00
B2HCT9	50S ribosomal protein L30	12,2	8,1	72	0	NO_SP	1.000066	0.000000	0.000000	0.000000	0.000000	3	1	32898	17274	1,90	0,00
A0A117DVB5	DNA repair protein RadA	6,4	46,0	444	0	NO_SP	1.000033	0.000002	0.000000	0.000000	0.000000	4	1	32882,33333	0	#JAKO/0!	0,00
A0A2Z5YCT0	Transcription antitermination regulator	7,4	26,1	250	0	NO_SP	0.999793	0.000212	0.000002	0.000000	0.000000	1	1	32851,33333	0	#JAKO/0!	0,00
A0A2Z5YFI9	Alpha-amylase	6,0	37,9	336	0	NO_SP	1.000061	0.000000	0.000000	0.000000	0.000000	4	1	32764	0	#JAKO/0!	0,00
A0A117DV62	Long-chain-fatty-acid--CoA ligase	5,1	47,8	439	0	NO_SP	1.000049	0.000002	0.000000	0.000000	0.000000	4	1	32537,13333	0	#JAKO/0!	0,00
B2HQZ3	Two-component system transcriptional regulator	4,5	22,7	205	0	NO_SP	1.000074	0.000000	0.000000	0.000000	0.000000	2	1	32485,66667	0	#JAKO/0!	0,00
A0A117DXX7	Amino acid ABC transporter	6,8	24,4	224	0	NO_SP	1.000069	0.000000	0.000000	0.000000	0.000000	4	1	32208,33333	0	#JAKO/0!	0,00
A0A3E2N1A3	Uncharacterized protein	9,6	64,1	573	8	NO_SP	0.999997	0.000037	0.000000	0.000000	0.000000	4	2	32183,33333	0	#JAKO/0!	0,00
B2HNL0	Fatty-acid-CoA ligase FadD35	5,4	59,2	547	0	NO_SP	0.999962	0.000061	0.000000	0.000000	0.000000	3	1	32137,66667	3834	8,38	0,00
A0A2Z5Y9G6	Uncharacterized protein	11,8	24,4	225	0	NO_SP	1.000059	0.000003	0.000000	0.000000	0.000000	1	1	32002,66667	0	#JAKO/0!	0,00
A0A2Z5YJ24	Uncharacterized protein	6,0	91,7	856	0	NO_SP	1.000045	0.000007	0.000000	0.000000	0.000000	5	1	31902	0	#JAKO/0!	0,00
B2HMT1	Conserved hypothetical membrane protein	4,7	62,2	573	0	NO_SP	1.000043	0.000003	0.000000	0.000000	0.000000	3	1	31870	0	#JAKO/0!	0,00
A0A2Z5YIE5	6-carboxy-5,6,7,8-tetrahydropterin synthase	6,6	17,8	159	0	NO_SP	1.000055	0.000000	0.000000	0.000000	0.000000	1	1	31843,66667	0	#JAKO/0!	0,00
A0A2Z5YMU5	Aspartate 1-decarboxylase	4,8	14,5	135	0	NO_SP	1.000037	0.000017	0.000000	0.000000	0.000000	4	1	31799,33333	0	#JAKO/0!	0,00
B2HNE4	NodS-like (Sam)-dependent methyltransferase	5,0	22,6	211	0	NO_SP	1.000059	0.000001	0.000000	0.000000	0.000000	4	1	31798	0	#JAKO/0!	0,00
A0A2Z5YE45	Sugar kinase	7,4	44,8	425	0	NO_SP	1.000053	0.000001	0.000000	0.000000	0.000000	4	2	31692,2	0	#JAKO/0!	0,00
A0A3E2MPI0	Sec-independent protein translocase protein TatC	6,5	31,5	285	6	NO_SP	1.000049	0.000002	0.000000	0.000000	0.000000	4	2	31369,66667	0	#JAKO/0!	0,00
A0A2Z5YNG2	Putative membrane protein	12,5	12,9	125	2	NO_SP	1.000000	0.000023	0.000000	0.000000	0.000000	1	1	31262,66667	0	#JAKO/0!	0,00
B2HT07	Aldehyde dehydrogenase NAD dependent AldA_2	4,6	53,3	497	0	NO_SP	1.000072	0.000000	0.000000	0.000000	0.000000	4	2	30769,33333	0	#JAKO/0!	0,00
B2HMV8	Beta-glucosidase BglS	4,5	74,8	711	0	NO_SP	1.000035	0.000018	0.000000	0.000000	0.000000	4	1	30711,66667	0	#JAKO/0!	0,00
A0A2Z5YLG3	Uncharacterized protein	5,6	32,2	304	0	NO_SP	1.000030	0.000000	0.000000	0.000000	0.000000	1	1	30582,4	0	#JAKO/0!	0,00
A0A3E2N1T7	Fluoroacetate dehalogenase	5,9	28,0	254	0	NO_SP	1.000068	0.000000	0.000000	0.000000	0.000000	4	1	30403,66667	0	#JAKO/0!	0,00
A0A2Z5YC79	Ribosome maturation factor RimM	4,5	18,6	174	0	NO_SP	1.000050	0.000000	0.000000	0.000000	0.000000	1	1	30350	0	#JAKO/0!	0,00
A0A2Z5YND1	Uncharacterized protein	6,8	42,5	399	2	NO_SP	0.999569	0.000382	0.000004	0.000001	0.000001	4	2	30226,33333	0	#JAKO/0!	0,00
A0A2Z5YJY3	2-oxoisovalerate dehydrogenase subunit beta	5,4	35,5	326	0	NO_SP	1.000014	0.000022	0.000001	0.000000	0.000000	4	2	30088	0	#JAKO/0!	0,00
A0A2Z5YK53	Uncharacterized protein	6,8	46,4	424	0	NO_SP	1.000063	0.000000	0.000000	0.000000	0.000000	3	1	30077,33333	0	#JAKO/0!	0,00
B2HJS1	Hydrogenase maturation factor, HypC	3,9	9,8	90	0	NO_SP	0.999942	0.000109	0.000005	0.000000	0.000000	3	1	29970,33333	0	#JAKO/0!	0,00
A0A2Z5YFL2	TetR family transcriptional regulator	7,0	21,7	199	0	NO_SP	1.000065	0.000000	0.000000	0.000000	0.000000	1	1	29941,33333	0	#JAKO/0!	0,00
A0A124BVI8	Transcriptional regulator, TetR family	8,5	17,3	159	0	NO_SP	1.000053	0.000003	0.000000	0.000000	0.000000	3	1	29873,66667	0	#JAKO/0!	0,00
B2HNW8	EsaT-6 like protein EsxG	6,5	9,7	97	0	NO_SP	1.000025	0.000017	0.000000	0.000000	0.000000	3	1	29653	0	#JAKO/0!	0,00
B2HP56	Probable primosomal protein N	8,4	69,2	648	0	NO_SP	1.000038	0.000000	0.000000	0.000000	0.000000	3	1	29648	0	#JAKO/0!	0,00
B2HG98	Phytoene dehydrogenase CrtI	7,0	54,4	509	0	NO_SP	0.990741	0.003089	0.006137	0.000013	0.000007	4	1	29591,33333	0	#JAKO/0!	0,00
B2HHK3	CMD domain-containing protein	5,2	18,4	178	0	NO_SP	1.000015	0.000050	0.000000	0.000000	0.000000	3	1	29580	0	#JAKO/0!	0,00
B2HQG3	Conserved secreted protein	5,1	17,6	166	0	SP	0.000487	0.998477	0.000231	0.000332	0.000237	2	1	29439,66667	0	#JAKO/0!	0,00
A0A2Z5YPD2	Uncharacterized protein	4,5	15,4	186	0	NO_SP	0.998579	0.001472	0.000001	0.000001	0.000001	3	1	29183,56667	0	#JAKO/0!	0,00
B2HT65	Conserved hypothetical transmembrane protein	9,5	48,1	456	9	NO_SP	0.999999	0.000000	0.000000	0.000000	0.000000	4	1	29179	0	#JAKO/0!	0,00
B2HLF2	Uncharacterized protein	6,0	38,0	361	0	NO_SP	1.000056	0.000000	0.000000	0.000000	0.000000	3	1	29117,66667	0	#JAKO/0!	0,00
A0A2Z5YG52	Cupin domain protein	4,7	12,7	116	0	NO_SP	1.000071	0.000000	0.000000	0.000000	0.000000	1	1	28999	0	#JAKO/0!	0,00
B2HN56	Polyamine aminopropyltransferase	6,1	55,3	526	7	NO_SP	1.000018	0.000017	0.000000	0.000000	0.000000	3	1	28920,66667	0	#JAKO/0!	0,00

B2HE33	Transcriptional repressor (Probably TetR-family) Mce3R	7,8	**44,0**	400	0	NO_SP	1.000054	0.000000	0.000000	0.000000	0.000000	4	2	**28859,9**	**0**	**#JAKO/0!**	**0,00**
B2HJL0	D-alanyl-D-alanine carboxypeptidase DacB	4,9	**29,8**	290	0	SP	0.000267	0.998976	0.000189	0.000213	0.000181	3	1	**28648**	**0**	**#JAKO/0!**	**0,00**
B2HJD4	Two-component sensor histidine kinase	5,6	**39,1**	364	0	NO_SP	0.994063	0.005698	0.000162	0.000032	0.000012	3	1	**28622,66667**	**0**	**#JAKO/0!**	**0,00**
B2HI87	Transcriptional regulatory protein	4,9	**28,1**	263	0	NO_SP	1.000002	0.000027	0.000000	0.000000	0.000000	4	1	**28566**	**0**	**#JAKO/0!**	**0,00**
A0A2Z5YPM0	Uncharacterized protein	6,8	**13,5**	127	0	NO_SP	1.000062	0.000000	0.000000	0.000000	0.000000	1	1	**28495**	**0**	**#JAKO/0!**	**0,00**
A0A100I2P7	Phospholipase C 4	7,3	**27,4**	251	0	NO_SP	1.000029	0.000000	0.000000	0.000000	0.000000	11	2	**28485,03333**	**0**	**#JAKO/0!**	**0,00**
A0A2Z5YCM0	Uncharacterized protein	7,2	**6,6**	61	0	NO_SP	0.996538	0.002762	0.000676	0.000010	0.000005	1	1	**28364,26667**	**0**	**#JAKO/0!**	**0,00**
B2HR91	Conserved hypothetical regulatory protein	8,5	**23,6**	215	0	NO_SP	1.000035	0.000008	0.000000	0.000000	0.000000	3	1	**28149**	**0**	**#JAKO/0!**	**0,00**
A0A3E2N0R9	Lon protease	4,1	**23,6**	217	0	NO_SP	1.000069	0.000000	0.000000	0.000000	0.000000	4	2	**28085**	**0**	**#JAKO/0!**	**0,00**
A0A3E2MNN9	Bacterial regulatory protein, tetR family	5,6	**21,1**	199	0	NO_SP	1.000051	0.000001	0.000000	0.000000	0.000000	2	1	**28043,33333**	**0**	**#JAKO/0!**	**0,00**
B2HQJ4	Adenylate cyclase	9,9	**57,7**	534	6	NO_SP	0.999727	0.000297	0.000000	0.000000	0.000000	5	2	**27874,66667**	**0**	**#JAKO/0!**	**0,00**
A0A3E2MN33	Putative mutator protein MutT4	6,4	**17,0**	154	0	NO_SP	1.000058	0.000000	0.000000	0.000000	0.000000	3	1	**27869,66667**	**0**	**#JAKO/0!**	**0,00**
B2HNN4	PE-PGRS family protein	4,0	**26,3**	283	0	NO_SP	1.000037	0.000007	0.000000	0.000000	0.000000	3	1	**27860**	**0**	**#JAKO/0!**	**0,00**
A0A3E2MRS9	HTH-type transcriptional regulator EthR	4,7	**19,2**	174	0	NO_SP	1.000059	0.000003	0.000000	0.000000	0.000000	4	2	**27792,33333**	**0**	**#JAKO/0!**	**0,00**
A0A3E2MQY4	Haloalkane dehalogenase	5,0	**32,9**	293	0	NO_SP	1.000034	0.000000	0.000000	0.000000	0.000000	1	1	**27434,66667**	**0**	**#JAKO/0!**	**0,00**
A0A117DZ02	Alpha/beta hydrolase	6,3	**27,5**	256	0	NO_SP	1.000041	0.000000	0.000000	0.000000	0.000000	3	1	**27405**	**0**	**#JAKO/0!**	**0,00**
A0A2Z5YM86	3-oxoacyl-ACP reductase	4,9	**30,4**	297	0	NO_SP	0.999063	0.000946	0.000004	0.000002	0.000001	3	1	**27296,17667**	**0**	**#JAKO/0!**	**0,00**
B2HJZ0	Oxidoreductase	5,9	**28,5**	271	0	NO_SP	1.000038	0.000001	0.000000	0.000000	0.000000	2	1	**27194,33333**	**0**	**#JAKO/0!**	**0,00**
A0A2Z5YIH8	Uncharacterized protein	4,8	**6,5**	55	0	NO_SP	1.000016	0.000000	0.000000	0.000000	0.000000	1	1	**27136,76667**	**0**	**#JAKO/0!**	**0,00**
A0A3E2MZS1	Uncharacterized protein	4,6	**20,1**	194	1	NO_SP	1.000031	0.000014	0.000000	0.000000	0.000000	4	1	**27119**	**0**	**#JAKO/0!**	**0,00**
B2HNA4	Aldolase_II domain-containing protein	5,8	**28,6**	261	0	NO_SP	1.000024	0.000048	0.000001	0.000000	0.000000	4	2	**26861,46667**	**0**	**#JAKO/0!**	**0,00**
A0A3E2MPU2	Diacylglycerol O-acyltransferase	9,7	**49,8**	462	0	NO_SP	1.000056	0.000000	0.000000	0.000000	0.000000	2	1	**26833,66667**	**0**	**#JAKO/0!**	**0,00**
B2HH28	Uncharacterized protein	4,7	**6,8**	59	0	NO_SP	1.000032	0.000000	0.000000	0.000000	0.000000	3	1	**26505,66667**	**2723**	**9,74**	**0,10**
A0A3E2MRT4	GDT1 family protein	8,7	**24,8**	234	5	NO_SP	1.000021	0.000000	0.000000	0.000000	0.000000	2	1	**26363**	**0**	**#JAKO/0!**	**0,00**
B2HHE4	Transcriptional regulator	8,3	**25,0**	224	0	NO_SP	1.000055	0.000000	0.000000	0.000000	0.000000	4	1	**26335,33333**	**0**	**#JAKO/0!**	**0,00**
A0A100IF59	Nucleoside-diphosphate-sugar epimerase	10,5	**50,1**	458	0	NO_SP	1.000064	0.000000	0.000000	0.000000	0.000000	4	1	**26271,33333**	**0**	**#JAKO/0!**	**0,00**
B2HD21	Uncharacterized protein	4,1	**15,3**	137	0	NO_SP	1.000061	0.000000	0.000000	0.000000	0.000000	3	1	**26035,66667**	**0**	**#JAKO/0!**	**0,00**
A0A2Z5YGW9	Mycobactin synthetase protein B	6,2	**79,1**	732	0	NO_SP	0.999860	0.000181	0.000001	0.000000	0.000000	1	2	**25966,66667**	**0**	**#JAKO/0!**	**0,00**
B2HIA6	ABC transporter permease protein GlnQ	7,9	**62,1**	589	5	SP	0.000221	0.999182	0.000142	0.000162	0.000131	4	1	**25882,33333**	**0**	**#JAKO/0!**	**0,00**
A0A117DY68	Low molecular weight protein tyrosine phosphatase	5,5	**17,1**	154	0	NO_SP	1.000038	0.000000	0.000000	0.000000	0.000000	4	1	**25821,66667**	**0**	**#JAKO/0!**	**0,00**
A0A2Z5YN49	Diacylglycerol acyltransferase	10,3	**29,1**	268	0	NO_SP	1.000031	0.000000	0.000000	0.000000	0.000000	2	1	**25787,66667**	**0**	**#JAKO/0!**	**0,00**
A0A124BVM2	Membrane protein	9,5	**16,6**	163	4	NO_SP	0.993922	0.006057	0.000018	0.000008	0.000004	4	1	**25783,33333**	**0**	**#JAKO/0!**	**0,00**
A0A100I101	Oxidoreductase	9,7	**42,8**	399	0	NO_SP	0.999999	0.000043	0.000000	0.000000	0.000000	4	1	**25781**	**0**	**#JAKO/0!**	**0,00**
B2HI11	Lipid transfer protein or keto acyl-CoA thiolase Ltp4	5,2	**37,7**	353	0	NO_SP	1.000029	0.000020	0.000000	0.000000	0.000000	3	1	**25716,66667**	**0**	**#JAKO/0!**	**0,00**
A0A2Z5YFL3	Uncharacterized protein	10,7	**13,0**	122	0	NO_SP	0.997444	0.002512	0.000062	0.000003	0.000002	4	2	**25447,5**	**0**	**#JAKO/0!**	**0,00**
A0A100IF60	TetR family transcriptional regulator	5,8	**23,6**	215	0	NO_SP	1.000050	0.000000	0.000000	0.000000	0.000000	4	1	**25079,33333**	**0**	**#JAKO/0!**	**0,00**
B2HG54	Cytochrome P450 105Q4 Cyp105Q4	6,1	**45,2**	414	0	NO_SP	1.000056	0.000001	0.000000	0.000000	0.000000	4	2	**24883**	**0**	**#JAKO/0!**	**0,00**
A0A2Z5YKX8	SLT_2 domain-containing protein	4,9	**21,6**	200	0	NO_SP	1.000027	0.000018	0.000000	0.000000	0.000000	4	1	**24615,8**	**0**	**#JAKO/0!**	**0,00**
A0A2Z5YCX9	Uncharacterized protein	9,9	**39,3**	364	10	NO_SP	1.000027	0.000002	0.000000	0.000000	0.000000	3	1	**24534,24667**	**0**	**#JAKO/0!**	**0,00**
B2HFE5	Conserved hypothetical membrane protein	10,4	**19,3**	176	1	NO_SP	1.000021	0.000011	0.000000	0.000000	0.000000	2	1	**24371,33333**	**0**	**#JAKO/0!**	**0,00**
A0A2Z5YLW2	Uncharacterized protein	6,8	**31,2**	281	0	NO_SP	1.000006	0.000033	0.000001	0.000000	0.000000	1	1	**24172,76667**	**0**	**#JAKO/0!**	**0,00**
A0A2Z5YNL1	Uncharacterized protein	4,1	**14,1**	137	0	NO_SP	0.971815	0.027902	0.000123	0.000065	0.000041	4	2	**24116,66667**	**0**	**#JAKO/0!**	**0,00**
B2HLQ9	Uncharacterized protein	5,6	**44,0**	387	0	NO_SP	1.000075	0.000001	0.000000	0.000000	0.000000	2	2	**24102,2**	**0**	**#JAKO/0!**	**0,00**
A0A2Z5YDG8	Ferredoxin	4,1	**12,3**	115	0	NO_SP	1.000061	0.000006	0.000000	0.000000	0.000000	1	1	**24083,33333**	**0**	**#JAKO/0!**	**0,00**
B2HKV1	Lipoprotein LppU	4,6	**18,4**	177	0	LIPO	0.000000	0.000369	0.999678	0.000000	0.000000	3	1	**23972,33333**	**0**	**#JAKO/0!**	**0,00**
B2HQE4	Transcriptional regulatory protein (Probably GntR-family)	7,3	**25,9**	237	0	NO_SP	1.000077	0.000000	0.000000	0.000000	0.000000	3	1	**23635**	**0**	**#JAKO/0!**	**0,00**
A0A117DU11	O-succinylbenzoic acid--CoA ligase	5,9	**34,9**	342	0	NO_SP	0.999877	0.000129	0.000000	0.000000	0.000000	3	1	**23274,56**	**0**	**#JAKO/0!**	**0,00**
A0A3E2MYY5	Sorbitol dehydrogenase	4,4	**38,0**	360	0	NO_SP	1.000018	0.000012	0.000000	0.000000	0.000000	3	1	**23248**	**0**	**#JAKO/0!**	**0,00**
A0A100I004	RNA polymerase sigma factor	6,1	**18,5**	163	0	NO_SP	1.000058	0.000000	0.000000	0.000000	0.000000	4	1	**23138,66667**	**0**	**#JAKO/0!**	**0,00**
B2HSR4	Uncharacterized protein	5,0	**9,3**	89	0	NO_SP	1.000069	0.000000	0.000000	0.000000	0.000000	3	1	**22618**	**4290**	**5,27**	**0,19**
B2HRI8	Conserved membrane protein MmpS-family	8,5	**15,8**	145	1	NO_SP	1.000012	0.000018	0.000005	0.000000	0.000000	3	1	**22187,66667**	**0**	**#JAKO/0!**	**0,00**
A0A3E2MNF4	Haloalkane dehalogenase	7,2	**29,1**	264	0	NO_SP	1.000064	0.000000	0.000000	0.000000	0.000000	4	1	**22067,66667**	**0**	**#JAKO/0!**	**0,00**
B2HH40	Probable M18 family aminopeptidase 2	6,4	**45,6**	430	0	NO_SP	1.000053	0.000000	0.000000	0.000000	0.000000	1	1	**22029**	**0**	**#JAKO/0!**	**0,00**
A0A117DWJ7	LysR family transcriptional regulator	6,0	**32,2**	298	0	NO_SP	1.000033	0.000001	0.000000	0.000000	0.000000	3	2	**21902,9**	**0**	**#JAKO/0!**	**0,00**
A0A3E2MVZ3	Bile acid-coenzyme A ligase	5,9	**53,0**	492	0	NO_SP	1.000071	0.000001	0.000000	0.000000	0.000000	2	1	**21829**	**0**	**#JAKO/0!**	**0,00**
A0A2Z5YGN4	Undecaprenyl-diphosphatase	9,4	**29,8**	276	5	NO_SP	0.999055	0.000960	0.000007	0.000001	0.000001	3	1	**21802,33333**	**0**	**#JAKO/0!**	**0,00**
A0A100I782	Uncharacterized protein	10,9	**11,3**	98	0	NO_SP	1.000055	0.000000	0.000000	0.000000	0.000000	3	1	**21494**	**0**	**#JAKO/0!**	**0,00**
A0A2Z5YJN6	Putative multidrug-efflux transporter	7,3	**41,4**	401	11	NO_SP	1.000040	0.000000	0.000000	0.000000	0.000000	4	1	**21334,36667**	**0**	**#JAKO/0!**	**0,00**
A0A100I7I3	Endopeptidase	4,9	**36,6**	360	0	NO_SP	0.943968	0.055779	0.000104	0.000074	0.000045	4	1	**21164,66667**	**0**	**#JAKO/0!**	**0,00**
B2HHL6	Conserved membrane protein	11,8	**14,6**	140	2	NO_SP	1.000013	0.000024	0.000000	0.000000	0.000000	3	1	**21031,66667**	**0**	**#JAKO/0!**	**0,00**
A0A3E2MVG7	Uncharacterized protein	7,7	**8,0**	74	0	NO_SP	1.000002	0.000036	0.000000	0.000000	0.000000	4	1	**21008,33333**	**0**	**#JAKO/0!**	**0,00**
A0A2Z5YMU1	Protease	4,5	**21,7**	218	0	SP	0.000679	0.998464	0.000206	0.000232	0.000194	4	1	**20941,33333**	**0**	**#JAKO/0!**	**0,00**
B2HHK4	Alternative RNA polymerase sigma factor	6,0	**24,2**	217	0	NO_SP	1.000065	0.000000	0.000000	0.000000	0.000000	2	1	**20842,66667**	**0**	**#JAKO/0!**	**0,00**
B2HFU7	5-3 exonuclease	4,5	**33,5**	318	0	NO_SP	1.000023	0.000002	0.000000	0.000000	0.000000	2	1	**20729,33333**	**0**	**#JAKO/0!**	**0,00**
B2HRH9	Conserved hypothetical membrane protein	6,2	**21,3**	197	0	NO_SP	1.000032	0.000000	0.000000	0.000000	0.000000	4	1	**20580,66667**	**0**	**#JAKO/0!**	**0,00**
A0A2Z5YJW9	ADP-ribose pyrophosphatase	4,7	**20,2**	178	0	NO_SP	1.000054	0.000000	0.000000	0.000000	0.000000	4	2	**20551,33333**	**0**	**#JAKO/0!**	**0,00**
A0A117DXE2	Secreted protein	4,9	**10,6**	106	0	SP	0.212372	0.786194	0.000281	0.000523	0.000336	4	1	**20321,33333**	**0**	**#JAKO/0!**	**0,00**
A0A2Z5YCP6	FhuF_C domain-containing protein	6,1	**22,4**	215	0	NO_SP	1.000055	0.000011	0.000000	0.000000	0.000000	4	1	**20242,33333**	**0**	**#JAKO/0!**	**0,00**
B2HNT8	Ribonuclease P protein component	12,3	**13,6**	120	0	NO_SP	1.000035	0.000000	0.000000	0.000000	0.000000	2	1	**20132**	**9920**	**2,03**	**0,49**
A0A2Z5YJT9	Ribonuclease E	4,1	**107,9**	996	0	NO_SP	1.000046	0.000000	0.000000	0.000000	0.000000	2	2	**19985,66667**	**0**	**#JAKO/0!**	**0,00**
B2HFC0	Aminotransferase	5,4	**42,1**	398	0	NO_SP	1.000054	0.000006	0.000000	0.000000	0.000000	4	1	**19964,03333**	**0**	**#JAKO/0!**	**0,00**
A0A3E2MUI2	Putative ribosomal N-acetyltransferase YdaF	10,2	**22,2**	203	0	NO_SP	0.999936	0.000124	0.000001	0.000000	0.000000	4	1	**19920**	**0**	**#JAKO/0!**	**0,00**
A0A2Z5YNX7	Uncharacterized protein	6,6	**30,3**	282	0	LIPO	0.000000	0.000001	1.000072	0.000000	0.000000	1	1	**19760,23333**	**0**	**#JAKO/0!**	**0,00**
A0A2Z5YM41	Biotin biosynthesis cytochrome P450	5,2	**47,7**	424	0	NO_SP	1.000062	0.000000	0.000000	0.000000	0.000000	1	1	**19585,33333**	**0**	**#JAKO/0!**	**0,00**
A0A100I6X9	PPE family protein	4,0	**36,4**	368	0	NO_SP	1.000031	0.000015	0.000000	0.000000	0.000000	3	1	**19059,66667**	**0**	**#JAKO/0!**	**0,00**
A0A2Z5YHB2	DUF202 domain-containing protein	6,5	**13,2**	125	3	NO_SP	1.000012	0.000014	0.000000	0.000000	0.000000	1	1	**18966,71**	**0**	**#JAKO/0!**	**0,00**
A0A2Z5Y902	SnoaL-like domain-containing protein	4,2	**12,4**	116	0	NO_SP	0.999999	0.000047	0.000000	0.000000	0.000000	1	1	**18909,33333**	**0**	**#JAKO/0!**	**0,00**

A0A100I5Y9	Acyl-CoA dehydrogenase	4,8	**73,9**	702	0	NO_SP	0.998792	0.001235	0.000010	0.000002	0.000001	3	1	**18769,89333**	**0**	**#JAKO/0!**	**0,00**
A0A100I4W1	Short chain dehydrogenase	5,6	**26,0**	247	0	NO_SP	1.000057	0.000000	0.000000	0.000000	0.000000	4	1	**18744,33333**	**0**	**#JAKO/0!**	**0,00**
A0A100IF66	Acyltransferase family protein	8,5	**20,4**	181	0	NO_SP	1.000049	0.000001	0.000000	0.000000	0.000000	4	1	**18735**	**0**	**#JAKO/0!**	**0,00**
B2HE72	Glycine betaine transport integral membrane protein BetP	6,8	**64,2**	598	12	NO_SP	1.000031	0.000003	0.000000	0.000000	0.000000	4	1	**18721,66667**	**0**	**#JAKO/0!**	**0,00**
B2HT26	Guanylate cyclase domain-containing protein	7,9	**30,2**	274	1	NO_SP	1.000048	0.000001	0.000000	0.000000	0.000000	3	1	**18678,4**	**0**	**#JAKO/0!**	**0,00**
B2HLI3	Acyl-CoA dehydrogenase FadE1_1	6,4	**47,4**	435	0	NO_SP	1.000070	0.000000	0.000000	0.000000	0.000000	2	1	**18577**	**0**	**#JAKO/0!**	**0,00**
A0A117DYF0	Short-chain type dehydrogenase/reductase	5,1	**29,3**	277	1	NO_SP	1.000095	0.000002	0.000000	0.000000	0.000000	4	1	**18507,43333**	**0**	**#JAKO/0!**	**0,00**
B2HGX5	Adenosylcobinamide kinase	4,7	**20,0**	186	0	NO_SP	1.000027	0.000011	0.000000	0.000000	0.000000	3	1	**18328,36667**	**0**	**#JAKO/0!**	**0,00**
A0A100I2N2	Uncharacterized protein	4,8	**6,7**	57	0	NO_SP	1.000013	0.000000	0.000000	0.000000	0.000000	3	1	**18312**	**0**	**#JAKO/0!**	**0,00**
B2HFU5	Uncharacterized protein	4,9	**80,4**	747	0	NO_SP	0.999838	0.000182	0.000004	0.000000	0.000000	2	1	**18145,9**	**0**	**#JAKO/0!**	**0,00**
B2HG08	Monooxygenase	8,6	**56,9**	505	0	NO_SP	0.999370	0.000586	0.000064	0.000002	0.000001	1	1	**18087,9**	**37699**	**0,48**	**2,08**
B2HT04	PPE family protein	4,0	**60,0**	621	0	NO_SP	1.000053	0.000004	0.000000	0.000000	0.000000	4	1	**18000,66667**	**0**	**#JAKO/0!**	**0,00**
B2HFK4	Oxidoreductase	6,9	**35,8**	332	0	NO_SP	1.000005	0.000032	0.000000	0.000000	0.000000	3	1	**17771,33333**	**0**	**#JAKO/0!**	**0,00**
A0A100I134	Uncharacterized protein	4,1	**9,1**	79	0	NO_SP	1.000067	0.000000	0.000000	0.000000	0.000000	4	1	**17355,33333**	**0**	**#JAKO/0!**	**0,00**
B2HI45	CoA-transferase (Beta subunit)	5,9	**27,1**	247	0	NO_SP	1.000059	0.000000	0.000000	0.000000	0.000000	4	1	**17341,73333**	**0**	**#JAKO/0!**	**0,00**
A0A2Z5YF53	Cytochrome P450 144	4,7	**43,8**	397	0	NO_SP	1.000035	0.000005	0.000000	0.000000	0.000000	2	1	**17256**	**0**	**#JAKO/0!**	**0,00**
A0A100I1K9	Amidohydrolase	6,0	**40,6**	369	0	NO_SP	1.000061	0.000001	0.000000	0.000000	0.000000	4	1	**17206**	**0**	**#JAKO/0!**	**0,00**
A0A2Z5YCA0	Putative transport protein MmpL1	6,3	**103,2**	953	12	NO_SP	0.996193	0.003596	0.000017	0.000007	0.000006	1	1	**17195,66667**	**0**	**#JAKO/0!**	**0,00**
B2HD44	Alcohol dehydrogenase AdhA	5,7	**35,1**	333	0	NO_SP	1.000033	0.000001	0.000000	0.000000	0.000000	4	1	**17153,33333**	**0**	**#JAKO/0!**	**0,00**
B2HJP1	Phosphatidate cytidylyltransferase	7,3	**32,2**	304	7	NO_SP	1.000004	0.000010	0.000000	0.000000	0.000000	3	1	**17100,2**	**0**	**#JAKO/0!**	**0,00**
B2HMC8	CP_ATPgrasp_1 domain-containing protein	5,2	**62,3**	557	0	NO_SP	1.000081	0.000000	0.000000	0.000000	0.000000	2	1	**17056,66667**	**28067**	**0,61**	**1,65**
A0A3E2MU38	Fluoroacetate dehalogenase	4,8	**36,1**	331	0	NO_SP	1.000068	0.000000	0.000000	0.000000	0.000000	1	1	**17007**	**0**	**#JAKO/0!**	**0,00**
A0A2Z5Y9X1	Glyoxalase	5,6	**15,2**	135	0	NO_SP	1.000017	0.000011	0.000001	0.000000	0.000000	4	2	**16969,33333**	**0**	**#JAKO/0!**	**0,00**
A0A2Z5YJ00	O-methylpimelyl-ACP methylesterase	4,5	**28,2**	265	0	NO_SP	1.000059	0.000000	0.000000	0.000000	0.000000	1	1	**16852,66667**	**0**	**#JAKO/0!**	**0,00**
A0A2Z5YH99	MHB domain-containing protein	4,5	**13,0**	126	2	SP	0.043656	0.933140	0.002052	0.019651	0.001078	3	1	**16849,66667**	**0**	**#JAKO/0!**	**0,00**
B2HM70	Uncharacterized protein	4,7	**31,6**	314	0	NO_SP	0.997547	0.002421	0.000019	0.000003	0.000003	3	1	**16717,33333**	**0**	**#JAKO/0!**	**0,00**
B2HJB2	Oxidoreductase	7,8	**49,0**	479	0	TAT	0.000000	0.000000	0.000000	1.000031	0.000000	3	1	**16570,66667**	**0**	**#JAKO/0!**	**0,00**
A0A2Z5YLV2	Uncharacterized protein	4,9	**6,5**	59	0	NO_SP	1.000049	0.000000	0.000000	0.000000	0.000000	1	1	**16525,63333**	**0**	**#JAKO/0!**	**0,00**
A0A2Z5YCK6	Putative transglutaminase-like protein	4,5	**28,7**	267	0	NO_SP	1.000027	0.000000	0.000000	0.000000	0.000000	1	1	**16259,33333**	**0**	**#JAKO/0!**	**0,00**
A0A2Z5Y7H9	Uncharacterized protein	4,1	**13,2**	118	0	NO_SP	0.999976	0.000049	0.000000	0.000000	0.000000	1	1	**16072,66667**	**1753**	**9,17**	**0,00**
B2HMZ9	Uncharacterized methyltransferase MMAR_0473	9,3	**27,3**	255	0	NO_SP	1.000034	0.000000	0.000000	0.000000	0.000000	3	1	**16061,33333**	**0**	**#JAKO/0!**	**0,00**
B2HD99	Lipoprotein LppD	7,0	**17,0**	164	0	NO_SP	1.000062	0.000002	0.000000	0.000000	0.000000	3	1	**15580,76667**	**0**	**#JAKO/0!**	**0,00**
A0A124BWS6	Metallophosphatase	8,0	**31,5**	280	0	NO_SP	1.000069	0.000001	0.000000	0.000000	0.000000	4	1	**15360,4**	**0**	**#JAKO/0!**	**0,00**
A0A2Z5YD84	Carrier domain-containing protein	5,4	**107,4**	1007	0	NO_SP	1.000052	0.000000	0.000000	0.000000	0.000000	3	1	**15247,66667**	**0**	**#JAKO/0!**	**0,00**
A0A2Z5YIP0	3-methyl-2-oxobutanoate dehydrogenase subunit alpha	5,0	**39,9**	363	0	NO_SP	1.000033	0.000010	0.000000	0.000000	0.000000	1	1	**14946**	**0**	**#JAKO/0!**	**0,00**
A0A100II35	Lipoprotein	4,5	**13,0**	122	0	NO_SP	0.999108	0.000893	0.000018	0.000004	0.000002	4	1	**14876**	**0**	**#JAKO/0!**	**0,00**
B2HH72	Serine/threonine-protein kinase PknF_2	7,2	**53,7**	512	0	NO_SP	1.000055	0.000000	0.000000	0.000000	0.000000	4	1	**14868,33333**	**0**	**#JAKO/0!**	**0,00**
A0A2Z5YII0	Haloacid dehalogenase	5,3	**25,6**	236	0	NO_SP	0.789997	0.209036	0.000439	0.000194	0.000127	1	1	**14605**	**0**	**#JAKO/0!**	**0,00**
B2HD87	NADP-dependent alcohol dehydrogenase AdhC	6,4	**34,8**	337	0	NO_SP	1.000031	0.000002	0.000000	0.000000	0.000000	3	2	**14380,2**	**0**	**#JAKO/0!**	**0,00**
A0A2Z5YLW3	Uncharacterized protein	9,9	**27,4**	251	0	NO_SP	0.999916	0.000097	0.000001	0.000000	0.000000	1	1	**14292,86667**	**0**	**#JAKO/0!**	**0,00**
A0A100I876	Membrane protein	11,5	**18,5**	178	5	NO_SP	1.000005	0.000000	0.000000	0.000000	0.000000	3	1	**14222,66667**	**0**	**#JAKO/0!**	**0,00**
B2HET7	Anti-sigma factor	5,0	**11,3**	101	0	NO_SP	1.000041	0.000005	0.000000	0.000000	0.000000	2	1	**14160,4**	**684**	**20,70**	**0,00**
A0A3E2MSE6	Putative amino acid permease YhdG	6,9	**59,7**	572	13	NO_SP	1.000015	0.000014	0.000000	0.000000	0.000000	3	1	**14122,18333**	**0**	**#JAKO/0!**	**0,00**
A0A2Z5YCL8	Uncharacterized protein	4,5	**23,3**	208	0	NO_SP	1.000068	0.000000	0.000000	0.000000	0.000000	1	1	**13921,2**	**0**	**#JAKO/0!**	**0,00**
B2HDQ5	Putative tRNA (cytidine(34)-2-O)-methyltransferase	6,6	**16,5**	154	0	NO_SP	1.000037	0.000010	0.000000	0.000000	0.000000	3	1	**13680,33333**	**0**	**#JAKO/0!**	**0,00**
B2HG38	Imidazolonepropionase	5,7	**42,8**	406	0	NO_SP	1.000012	0.000047	0.000000	0.000000	0.000000	5	2	**13604,66667**	**0**	**#JAKO/0!**	**0,00**
A0A2Z5YB48	ABC transporter ATP-binding/permease protein	7,3	**88,8**	841	6	NO_SP	0.998923	0.001105	0.000001	0.000001	0.000001	1	1	**13571,26667**	**0**	**#JAKO/0!**	**0,00**
A0A3E2MNE4	Urease subunit gamma	6,0	**11,1**	100	0	NO_SP	1.000041	0.000001	0.000000	0.000000	0.000000	4	1	**13527,66667**	**0**	**#JAKO/0!**	**0,00**
A0A3E2N2D7	Levodione reductase	9,2	**11,0**	101	1	NO_SP	1.000076	0.000000	0.000000	0.000000	0.000000	4	1	**13503**	**0**	**#JAKO/0!**	**0,00**
A0A2Z5Y8J6	MFS transporter	10,9	**40,8**	394	11	NO_SP	1.000021	0.000000	0.000000	0.000000	0.000000	4	1	**13192,66667**	**0**	**#JAKO/0!**	**0,00**
A0A117DWX3	Lipoprotein lprE	4,5	**18,4**	179	0	LIPO	0.000333	0.000064	0.999617	0.000000	0.000001	4	1	**12985,33333**	**231**	**56,22**	**0,00**
A0A3E2MWM2	Phosphoribosylformylglycinamidine synthase subunit PurS	4,1	**8,6**	79	0	NO_SP	1.000050	0.000000	0.000000	0.000000	0.000000	4	2	**12905,66667**	**0**	**#JAKO/0!**	**0,00**
B2HCZ8	Glutamine--fructose-6-phosphate aminotransferase [isomerizing]	5,2	**67,7**	625	0	NO_SP	1.000082	0.000000	0.000000	0.000000	0.000000	1	1	**12717,33333**	**0**	**#JAKO/0!**	**0,00**
B2HRC3	Transmembrane ATP-binding protein ABC transporter	8,3	**62,2**	579	6	SP	0.497033	0.499353	0.001510	0.000576	0.000397	3	1	**12701**	**0**	**#JAKO/0!**	**0,00**
B2HT02	Enoyl-CoA hydratase, EchA1_1	5,4	**28,5**	267	0	NO_SP	1.000040	0.000009	0.000000	0.000000	0.000000	4	1	**12506,66667**	**0**	**#JAKO/0!**	**0,00**
A0A2Z5YPU6	Methanol dehydrogenase transcriptional regulator MoxR	5,7	**35,5**	323	0	NO_SP	0.999905	0.000132	0.000000	0.000000	0.000000	1	1	**12417**	**0**	**#JAKO/0!**	**0,00**
A0A100IFB5	Adenylate cyclase	9,3	**36,6**	346	6	NO_SP	1.000034	0.000001	0.000000	0.000000	0.000000	4	1	**12180**	**0**	**#JAKO/0!**	**0,00**
B2HFG7	Conserved hypothetical metal-dependent hydrolase	6,4	**44,1**	381	0	NO_SP	1.000049	0.000000	0.000000	0.000000	0.000000	4	1	**12071,66667**	**0**	**#JAKO/0!**	**0,00**
A0A2Z5YJA3	Pyruvate dehydrogenase E1 component	5,8	**83,4**	775	0	NO_SP	0.999952	0.000090	0.000003	0.000000	0.000000	1	1	**12036,33333**	**0**	**#JAKO/0!**	**0,00**
B2HH45	Uncharacterized protein	7,4	**17,4**	154	0	NO_SP	1.000075	0.000000	0.000000	0.000000	0.000000	3	1	**11841,23333**	**0**	**#JAKO/0!**	**0,00**
A0A346F5X2	ESAT-6-like protein	5,0	**10,2**	93	0	NO_SP	1.000055	0.000000	0.000000	0.000000	0.000000	3	1	**11832,33333**	**0**	**#JAKO/0!**	**0,00**
B2HK10	Conserved hypothetical membrane protein	9,8	**14,8**	138	4	NO_SP	1.000060	0.000001	0.000000	0.000000	0.000000	3	1	**11344,06667**	**0**	**#JAKO/0!**	**0,00**
A0A3E2MUF8	Acetyltransferase Pat	5,2	**35,6**	330	0	NO_SP	1.000054	0.000010	0.000000	0.000000	0.000000	3	1	**11261,66667**	**0**	**#JAKO/0!**	**0,00**
A0A2Z5YMP9	LacI family transcriptional regulator	8,2	**37,3**	348	0	NO_SP	1.000074	0.000000	0.000000	0.000000	0.000000	4	1	**10931,43333**	**0**	**#JAKO/0!**	**0,00**
B2HPE2	Transcriptional regulator	5,0	**21,5**	204	0	NO_SP	1.000060	0.000000	0.000000	0.000000	0.000000	3	1	**10874,66667**	**0**	**#JAKO/0!**	**0,00**
B2HE74	Ferric uptake regulation protein FurA	4,6	**14,8**	138	0	NO_SP	1.000070	0.000000	0.000000	0.000000	0.000000	5	1	**10847**	**0**	**#JAKO/0!**	**0,00**
B2HR39	Cytochrome P450 139A3 Cyp139A3	9,6	**48,2**	433	0	NO_SP	1.000071	0.000000	0.000000	0.000000	0.000000	4	1	**10777,7**	**0**	**#JAKO/0!**	**0,00**
A0A2Z5YG64	Uncharacterized protein	5,0	**79,7**	735	0	NO_SP	1.000049	0.000000	0.000000	0.000000	0.000000	2	1	**10444**	**0**	**#JAKO/0!**	**0,00**
A0A3E2MZE8	Inositol 2-dehydrogenase/D-chiro-inositol 3-dehydrogenase	6,7	**29,3**	265	0	NO_SP	1.000028	0.000000	0.000000	0.000000	0.000000	2	1	**10319,86667**	**0**	**#JAKO/0!**	**0,00**
A0A2Z5YHL3	DEAD/DEAH box helicase	6,4	**169,7**	1545	0	NO_SP	1.000054	0.000000	0.000000	0.000000	0.000000	1	2	**9759,766667**	**61640**	**0,16**	**6,32**
A0A3E2N314	Allophanate hydrolase	6,5	**32,4**	315	0	NO_SP	0.999566	0.000385	0.000069	0.000002	0.000001	1	1	**9653,333333**	**36186**	**0,27**	**3,75**
B2HDA5	Anti-sigma factor antagonist	9,5	**15,4**	146	0	NO_SP	0.998489	0.001496	0.000019	0.000008	0.000004	2	1	**9499,733333**	**0**	**#JAKO/0!**	**0,00**
A0A3E2MMM5	Penicillin-insensitive transglycosylase	6,1	**89,6**	851	1	NO_SP	1.000016	0.000022	0.000001	0.000000	0.000000	1	1	**9228,333333**	**0**	**#JAKO/0!**	**0,00**
A0A2Z5YPJ2	Uncharacterized protein	6,3	**14,1**	136	0	NO_SP	1.000039	0.000001	0.000000	0.000000	0.000000	1	1	**8834**	**0**	**#JAKO/0!**	**0,00**
B2HFL3	Methanol dehydrogenase transcriptional regulatory protein MoxR3	5,9	**35,5**	322	0	NO_SP	0.999954	0.000060	0.000007	0.000000	0.000000	3	1	**8517,066667**	**0**	**#JAKO/0!**	**0,00**
A0A2Z5YD09	Putative zinc protease	7,0	**44,3**	405	0	NO_SP	1.000038	0.000000	0.000000	0.000000	0.000000	1	1	**8450,066667**	**0**	**#JAKO/0!**	**0,00**
B2HI22	Short-chain alcohol dehydrogenase	5,6	**27,6**	260	0	NO_SP	1.000047	0.000000	0.000000	0.000000	0.000000	2	1	**8284,066667**	**0**	**#JAKO/0!**	**0,00**

B2HR63	Uncharacterized protein	3,9	**6,2**	58	0	NO_SP	1.000037	0.000000	0.000000	0.000000	0.000000	2	2	**8161,233333**	**0**	**#JAKO/0!**	**0,00**
A0A2Z5YET3	Transposase	8,5	**11,1**	97	0	NO_SP	1.000036	0.000000	0.000000	0.000000	0.000000	3	1	**7543**	**0**	**#JAKO/0!**	**0,00**
A0A2Z5YB77	K(+)/H(+) antiporter YhaU	8,5	**39,2**	381	10	NO_SP	1.000025	0.000000	0.000000	0.000000	0.000000	3	2	**7194,366667**	**0**	**#JAKO/0!**	**0,00**
A0A100I1J3	Glutamine synthetase GlnA3	5,1	**47,5**	450	0	NO_SP	0.993771	0.005929	0.000196	0.000021	0.000009	1	1	**6705,133333**	**0**	**#JAKO/0!**	**0,00**
A0A100IEF3	Carbon monoxide dehydrogenase F protein	4,8	**27,9**	277	0	NO_SP	0.999139	0.000949	0.000002	0.000001	0.000001	4	1	**6546,3**	**0**	**#JAKO/0!**	**0,00**
B2HLR8	Conserved protein	4,7	**13,6**	126	0	NO_SP	1.000074	0.000000	0.000000	0.000000	0.000000	3	1	**5860,5**	**0**	**#JAKO/0!**	**0,00**
A0A117DU70	Phosphopantetheine-binding protein	4,0	**8,6**	76	0	NO_SP	0.999970	0.000067	0.000000	0.000000	0.000000	3	1	**5696,866667**	**0**	**#JAKO/0!**	**0,00**
A0A2Z5YG57	Uncharacterized protein	9,0	**36,2**	321	0	NO_SP	1.000022	0.000051	0.000002	0.000000	0.000000	1	1	**4758,033333**	**0**	**#JAKO/0!**	**0,00**
B2HQ43	Conserved hypothetical membrane protein	9,1	**18,3**	166	3	NO_SP	0.999998	0.000001	0.000000	0.000000	0.000000	3	1	**4343,7**	**0**	**#JAKO/0!**	**0,00**
A0A100I2N8	Cytochrome p450 107b1	6,8	**28,5**	259	0	NO_SP	1.000079	0.000000	0.000000	0.000000	0.000000	4	1	**4334,2**	**0**	**#JAKO/0!**	**0,00**
A0A2Z5YDY8	Polyketide synthase	4,9	**169,5**	1598	0	NO_SP	1.000023	0.000001	0.000000	0.000000	0.000000	1	1	**3760,5**	**0**	**#JAKO/0!**	**0,00**
A0A2Z5YD45	Ribosome-binding factor A	4,4	**18,5**	175	0	NO_SP	1.000040	0.000013	0.000001	0.000000	0.000000	1	2	**2181,833333**	**0**	**#JAKO/0!**	**0,00**
A0A3E2MUJ5	Biotin carboxyl carrier protein	5,0	**70,0**	660	0	NO_SP	1.000029	0.000010	0.000000	0.000000	0.000000	2	1	**0**	**68675**	**0,00**	**#JAKO/0!**
A0A100I304	MCE-family protein	10,1	**37,5**	354	1	NO_SP	0.931555	0.067012	0.000345	0.000200	0.000148	1	1	**0**	**373263**	**0,00**	**#JAKO/0!**
A0A100HZV0	Integral membrane protein	9,2	**23,8**	220	5	NO_SP	1.000019	0.000000	0.000000	0.000000	0.000000	3	1	**0**	**22408**	**0,35**	**2,86**
B2HDJ5	Peptidyl-tRNA hydrolase	9,6	**20,7**	191	0	NO_SP	1.000051	0.000000	0.000000	0.000000	0.000000	4	2	**0**	**15479**	**1,25**	**0,80**
B2HR09	Uncharacterized protein	4,1	**9,3**	84	0	NO_SP	1.000054	0.000002	0.000000	0.000000	0.000000	3	2	**0**	**14900**	**0,16**	**6,21**
A0A117DVW1	Secreted protein	9,9	**23,9**	227	6	NO_SP	1.000041	0.000004	0.000000	0.000000	0.000000	3	1	**0**	**11430**	**1,59**	**0,63**
A0A3E2MTS1	ESX-1 secretion-associated protein EspA	5,3	**40,2**	397	2	NO_SP	1.000022	0.000011	0.000000	0.000000	0.000000	1	1	**0**	**9200**	**0,00**	**0,00**
A0A2Z5YFG3	Urease subunit beta	6,8	**11,1**	103	0	NO_SP	1.000065	0.000000	0.000000	0.000000	0.000000	4	2	**0**	**1253**	**4,06**	**0,00**
B2HIE4	NTP_transf_9 domain-containing protein	5,7	**17,9**	166	0	NO_SP	1.000069	0.000001	0.000000	0.000000	0.000000	4	1	**0**	**1109**	**11,08**	**0,00**
