# Supplementary material for: In vitro and ex vivo proteomics of Mycobacterium marinum biofilms and the development of biofilm-binding synthetic nanobodies
Source: mSystems. 2023 May 15;8(3):e01073-22. doi: 10.1128/msystems.01073-22 (PMC10308901; doi:10.1128/msystems.01073-22)
Supplement: Table S2 — List of proteins (n, 448) with significant higher abundancies after biotinylation (cells in intense red) and with predicted molecular weights, pI values, sequence lengths, and secretory motifs/subcellular location. Naturally biotinylated example proteins are in bold letter and shaded in grey. [file msystems.01073-22-s0002.pdf]

Table S2. List of proteins (*n* , 448) with significant higher abundancies after biotinylation (cells in intense red) and with predicted molecular weights, pI values, sequence lengths, and secretory motifs/subcellular location. Naturally biotinylated example proteins are in bold letter and shaded in grey. GroEL

	Predicted secretory/export motifs											Fold-change				
**UniProtKB/AC_ID and Name of protein**	No. TMHM	Pred.	OTHER	SP (Sec/SPI)	LIPO (Sec/SPII)	TAT (Tat/SPI)	TATLIPO (Sec/SPII)	PILIN (Sec/SPIII)	pI	Mw (kDa)	No. amino acids	BIOT-vs-nonBIOT	No. uniq pept	Uniq seq cov [%]	Mol. weight [kDa]	T-test stat BIOT_NON-BIOT
>tr|A0A2Z5YM62|A0A2Z5YM62_MYCMR Universal stress protein	0	NO_SP	1.000042	0.000000	0.000000	0.000000	0.000000	0.000000	5,5	32,0	304	**41,28**	9	57,2	32,0	16,0
>tr|B2HJA8|B2HJA8_MYCMM Uncharacterized protein	0	NO_SP	0.999209	0.000800	0.000015	0.000004	0.000003	0.000005	5,2	37,1	343	**32,40**	1	3,5	37,1	26,9
>tr|A0A2Z5YK33|A0A2Z5YK33_MYCMR ATP-dependent RNA helicase DeaD	0	NO_SP	1.000019	0.000003	0.000000	0.000000	0.000000	0.000000	9,5	62,6	571	**30,55**	22	56,6	62,6	12,4
>tr|A0A2Z5YNY7|A0A2Z5YNY7_MYCMR Universal stress protein	0	NO_SP	1.000053	0.000000	0.000000	0.000000	0.000000	0.000000	5,3	32,4	306	**23,89**	10	56,2	32,4	10,9
>tr|A0A2Z5Y928|A0A2Z5Y928_MYCMR Chaperone protein DnaK	0	NO_SP	1.000067	0.000000	0.000000	0.000000	0.000000	0.000000	4,4	66,4	622	**19,28**	45	72	66,4	6,2
>tr|A0A2Z5YHD7|A0A2Z5YHD7_MYCMR Pyruvate dehydrogenase E1 component	0	NO_SP	1.000037	0.000004	0.000000	0.000000	0.000000	0.000000	6,1	103,4	929	**18,52**	2	4,2	103,4	16,4
>tr|A0A2Z5YJG3|A0A2Z5YJG3_MYCMR TPM_phosphatase domain-containing protein	2	SP	0.001963	0.996857	0.000318	0.000311	0.000260	0.000251	5,4	69,7	662	**18,30**	19	49,1	69,7	9,6
>tr|A0A2Z5YG25|A0A2Z5YG25_MYCMR Proteasome accessory factor PafA2	0	NO_SP	1.000040	0.000002	0.000000	0.000000	0.000000	0.000000	5,3	54,9	502	**18,17**	18	59,6	54,9	11,7
>tr|A0A2Z5YDN3|A0A2Z5YDN3_MYCMR Uncharacterized protein	0	NO_SP	1.000030	0.000015	0.000000	0.000000	0.000000	0.000000	5,1	96,4	891	**16,06**	22	39,3	96,4	9,0
>tr|B2HRD2|B2HRD2_MYCMM Non-specific serine/threonine protein kinase	1	NO_SP	1.000055	0.000000	0.000000	0.000000	0.000000	0.000000	5,3	63,6	596	**15,26**	13	29,7	63,6	7,7
>tr|A0A2Z5YIW6|A0A2Z5YIW6_MYCMR Acyl-CoA dehydrogenase	0	NO_SP	1.000069	0.000000	0.000000	0.000000	0.000000	0.000000	4,8	42,5	388	**15,02**	14	58	42,5	5,6
>tr|A0A3E2MX79|A0A3E2MX79_MYCMR LGFP repeat protein	2	SP	0.000270	0.999044	0.000173	0.000203	0.000160	0.000139	4,3	80,6	766	**14,68**	15	37,1	80,6	21,8
>tr|A0A2Z5YN99|A0A2Z5YN99_MYCMR Phosphoribosylamine--glycine ligase	0	NO_SP	1.000037	0.000014	0.000000	0.000000	0.000000	0.000000	4,9	43,2	422	**14,59**	14	55,2	43,2	6,5
>tr|A0A117DTU2|A0A117DTU2_9MYCO Ferritin	0	NO_SP	1.000034	0.000001	0.000000	0.000000	0.000000	0.000000	4,6	21,5	191	**14,54**	8	53,4	21,5	7,1
>tr|A0A3E2N0M2|A0A3E2N0M2_MYCMR Dihydrolipoyl dehydrogenase	0	NO_SP	1.000071	0.000001	0.000000	0.000000	0.000000	0.000000	6,0	49,4	464	**14,03**	30	76,1	49,4	4,7
>tr|A0A2Z5YAM4|A0A2Z5YAM4_MYCMR Glutamine--fructose-6-phosphate aminotransferase [isomerizing]	0	NO_SP	1.000056	0.000001	0.000000	0.000000	0.000000	0.000000	5,2	68,7	635	**13,89**	1	4,1	68,7	7,3
>tr|A0A3E2MRX1|A0A3E2MRX1_MYCMR UDP-galactopyranose mutase	0	NO_SP	0.999810	0.000133	0.000100	0.000000	0.000000	0.000000	4,9	46,0	402	**13,48**	10	30,8	45,9	14,8
>tr|A0A2Z5YBZ0|A0A2Z5YBZ0_MYCMR CoA ester lyase	0	NO_SP	1.000056	0.000001	0.000000	0.000000	0.000000	0.000000	4,5	32,8	305	**13,33**	13	59,7	32,8	17,5
>tr|A0A2Z5YIC9|A0A2Z5YIC9_MYCMR Assimilatory sulfite reductase (ferredoxin)	0	NO_SP	0.999766	0.000229	0.000008	0.000001	0.000000	0.000004	5,7	61,9	555	**13,15**	17	43,2	61,9	9,1
>tr|A0A2Z5YGI3|A0A2Z5YGI3_MYCMR Uncharacterized protein	0	NO_SP	1.000051	0.000001	0.000000	0.000000	0.000000	0.000000	4,9	67,0	619	**12,69**	1	3,9	67,0	3,9
>tr|B2HP63|B2HP63_MYCMM Riboflavin biosynthesis protein RibD	0	NO_SP	1.000067	0.000000	0.000000	0.000000	0.000000	0.000000	6,7	35,3	339	**12,39**	15	69	35,3	45,6
>tr|A0A2Z5YNT0|A0A2Z5YNT0_MYCMR Putative arabinosyltransferase B	12	NO_SP	1.000038	0.000013	0.000000	0.000000	0.000000	0.000000	9,7	116,1	1075	**12,31**	17	27,3	116,1	6,1
>tr|A0A2Z5Y9V8|A0A2Z5Y9V8_MYCMR CbiA domain-containing protein	0	NO_SP	1.000066	0.000001	0.000000	0.000000	0.000000	0.000000	5,9	47,1	438	**12,26**	13	49,8	47,1	8,6
>tr|A0A2Z5YCV5|A0A2Z5YCV5_MYCMR Mycothione reductase	0	NO_SP	1.000074	0.000001	0.000000	0.000000	0.000000	0.000000	5,4	49,5	459	**12,20**	13	50,3	49,5	7,0
>tr|B2HMA0|B2HMA0_MYCMM Acyl-[acyl-carrier protein] desaturase DesA1_1	0	NO_SP	1.000048	0.000000	0.000000	0.000000	0.000000	0.000000	5,8	39,2	338	**11,91**	2	6,8	39,2	9,5
>tr|A0A2Z5YFW5|A0A2Z5YFW5_MYCMR Nitrate ABC transporter substrate-binding protein	0	NO_SP	1.000080	0.000000	0.000000	0.000000	0.000000	0.000000	5,4	66,4	624	**11,86**	19	50,5	66,4	24,8
>tr|A0A2Z5YMQ6|A0A2Z5YMQ6_MYCMR Cysteine--tRNA ligase	0	NO_SP	1.000079	0.000000	0.000000	0.000000	0.000000	0.000000	5,7	50,1	452	**11,37**	15	50,9	50,1	5,0
>tr|A0A2Z5Y9J9|A0A2Z5Y9J9_MYCMR Calcium dodecin	0	NO_SP	1.000069	0.000000	0.000000	0.000000	0.000000	0.000000	8,6	8,0	71	**11,29**	5	73,2	8,0	38,2
>tr|A0A3E2N170|A0A3E2N170_MYCMR Glucose-6-phosphate 1-dehydrogenase	0	NO_SP	1.000036	0.000000	0.000000	0.000000	0.000000	0.000000	5,6	52,8	469	**10,87**	15	42,6	52,8	17,6
>tr|A0A2Z5Y9Q5|A0A2Z5Y9Q5_MYCMR Fatty acyl-CoA reductase	0	NO_SP	1.000048	0.000000	0.000000	0.000000	0.000000	0.000000	7,7	33,3	312	**10,85**	15	63,8	33,3	3,2
>tr|A0A2Z5YJG4|A0A2Z5YJG4_MYCMR Arginine--tRNA ligase	0	NO_SP	1.000082	0.000000	0.000000	0.000000	0.000000	0.000000	5,2	59,7	550	**10,75**	22	69,3	59,7	8,9
>tr|A7BJ00|A7BJ00_MYCMR Chaperone protein DnaJ	0	NO_SP	1.000063	0.000000	0.000000	0.000000	0.000000	0.000000	7,9	41,6	396	**10,25**	16	61,9	41,6	13,1
>tr|A0A2Z5YDT2|A0A2Z5YDT2_MYCMR Riboflavin biosynthesis protein RibBA	0	NO_SP	1.000038	0.000001	0.000000	0.000000	0.000000	0.000000	5,3	46,0	425	**10,25**	14	50,1	46,0	30,6
>tr|B2HFB2|B2HFB2_MYCMM DNA helicase	0	NO_SP	1.000051	0.000004	0.000001	0.000000	0.000000	0.000000	5,8	60,8	549	**10,04**	14	35,3	60,8	11,3
>tr|A0A2Z5YAH2|A0A2Z5YAH2_MYCMR Ferredoxin reductase	0	NO_SP	1.000066	0.000000	0.000000	0.000000	0.000000	0.000000	4,6	42,4	401	**9,65**	18	62,6	42,4	11,9
>sp|B2HQM5|THIC_MYCMM Phosphomethylpyrimidine synthase	0	NO_SP	0.999315	0.000645	0.000062	0.000001	0.000001	0.000006	5,1	59,4	546	**9,58**	17	51,6	59,4	6,8
>tr|A0A3E2MPT4|A0A3E2MPT4_MYCMR Putative NAD(P)H nitroreductase acg	0	NO_SP	1.000072	0.000000	0.000000	0.000000	0.000000	0.000000	5,4	55,0	494	**9,38**	4	14,2	55,0	20,3
>tr|B2HGH5|B2HGH5_MYCMM Acyl-CoA dehydrogenase FadE24	0	NO_SP	1.000005	0.000042	0.000000	0.000000	0.000000	0.000000	5,4	49,8	465	**9,23**	13	45,6	49,8	12,7
>tr|A0A2Z5YAL3|A0A2Z5YAL3_MYCMR Neutral ceramidase	0	NO_SP	1.000035	0.000002	0.000000	0.000000	0.000000	0.000000	6,3	68,8	637	**9,18**	20	46	68,8	11,4
>tr|A0A3E2MPR5|A0A3E2MPR5_MYCMR Acetolactate synthase	0	NO_SP	1.000020	0.000010	0.000000	0.000000	0.000000	0.000000	4,9	64,5	606	**9,15**	19	45,2	64,5	9,8
>tr|A0A2Z5YAJ1|A0A2Z5YAJ1_MYCMR Inosine-5'-monophosphate dehydrogenase	0	NO_SP	1.000068	0.000002	0.000000	0.000000	0.000000	0.000000	6,4	55,4	532	**9,15**	22	61,1	55,4	7,3
>tr|A0A2Z5YMT0|A0A2Z5YMT0_MYCMR DNA topoisomerase 1	0	NO_SP	1.000073	0.000000	0.000000	0.000000	0.000000	0.000000	5,7	95,8	873	**9,06**	34	56,7	95,8	6,8
>tr|B2HL65|B2HL65_MYCMM Transmembrane ATP-binding protein ABC transorter	4	NO_SP	0.999810	0.000205	0.000000	0.000000	0.000000	0.000000	6,9	72,2	690	**9,01**	10	19,9	72,2	27,1
>tr|B2HGH4|B2HGH4_MYCMM Acyl-CoA dehydrogenase FadE23	0	NO_SP	1.000067	0.000000	0.000000	0.000000	0.000000	0.000000	5,1	43,5	401	**8,97**	15	46,4	43,5	5,4
>tr|A0A2Z5YL20|A0A2Z5YL20_MYCMR Uncharacterized protein	0	LIPO	0.240574	0.060752	0.698307	0.000135	0.000127	0.000128	4,9	60,1	552	**8,83**	11	32,6	60,1	13,8
>tr|A0A3E2MQN4|A0A3E2MQN4_MYCMR 4-aminobutyrate aminotransferase PuuE	0	NO_SP	1.000062	0.000001	0.000000	0.000000	0.000000	0.000000	4,7	47,1	446	**8,80**	23	70,4	47,1	6,4
>tr|A0A2Z5YNP4|A0A2Z5YNP4_MYCMR Aspartate-semialdehyde dehydrogenase	0	NO_SP	1.000083	0.000000	0.000000	0.000000	0.000000	0.000000	4,7	36,9	352	**8,71**	14	66,8	36,9	32,7
>sp|B2HDR8|FOLD_MYCMM Bifunctional protein FolD	0	NO_SP	1.000075	0.000000	0.000000	0.000000	0.000000	0.000000	6,6	29,7	281	**8,56**	13	71,9	29,7	11,0
>tr|A0A2Z5YN85|A0A2Z5YN85_MYCMR Peptidoglycan glycosyltransferase	1	NO_SP	0.966738	0.005773	0.000603	0.000048	0.000024	0.026845	5,3	84,4	803	**8,44**	17	30,3	84,4	8,5
>tr|A0A100IFA3|A0A100IFA3_9MYCO Formaldehyde dehydrogenase	0	NO_SP	1.000037	0.000016	0.000000	0.000000	0.000000	0.000000	4,4	35,8	342	**8,30**	1	3,2	35,8	7,3
>tr|A0A2Z5YJX7|A0A2Z5YJX7_MYCMR Multifunctional fusion protein	0	NO_SP	0.999975	0.000085	0.000000	0.000000	0.000000	0.000000	6,7	68,0	616	**8,12**	17	39,8	68,0	10,5
>tr|A0A2Z5Y9E2|A0A2Z5Y9E2_MYCMR Putative lipoprotein aminopeptidase LpqL	0	LIPO	0.000099	0.000007	0.999930	0.000000	0.000000	0.000000	4,6	51,5	490	**8,05**	11	35,1	51,5	6,2
>tr|B2HD10|B2HD10_MYCMM Chaperonin GroEL1	0	NO_SP	1.000081	0.000000	0.000000	0.000000	0.000000	0.000000	4,5	55,9	539	**7,90**	4	4,8	55,9	13,6
>tr|A0A117DU97|A0A117DU97_9MYCO	0	NO_SP	1.000059	0.000000	0.000000	0.000000	0.000000	0.000000	5,2	23,1	223	**7,79**	16	100	23,1	9,6
>tr|B2HIF9|B2HIF9_MYCMM Aspartyl/glutamyl-tRNA(Asn/Gln) amidotransferase subunit B	0	NO_SP	1.000013	0.000035	0.000000	0.000000	0.000000	0.000000	4,8	54,2	502	**7,75**	21	65,3	54,2	7,3
>sp|B2HDI5|ENO_MYCMM Enolase	0	NO_SP	1.000050	0.000001	0.000000	0.000000	0.000000	0.000000	4,3	44,8	428	**7,74**	15	46,5	44,8	3,7
>tr|A0A2Z5Y9I0|A0A2Z5Y9I0_MYCMR Protein GrpE	0	NO_SP	1.000072	0.000000	0.000000	0.000000	0.000000	0.000000	4,4	22,7	217	**7,52**	12	68,2	22,7	18,8
>tr|B2HEQ0|B2HEQ0_MYCMM Two-component sensory transduction transcriptional regulatory protein MtrA	0	NO_SP	1.000070	0.000000	0.000000	0.000000	0.000000	0.000000	5,4	24,9	225	**7,49**	11	78,7	24,9	6,6
>tr|A0A2Z5YD85|A0A2Z5YD85_MYCMR Ribonuclease D	0	NO_SP	0.999316	0.000684	0.000034	0.000001	0.000001	0.000001	5,1	47,1	435	**7,48**	10	35,9	47,1	16,0
>tr|A0A2Z5YB07|A0A2Z5YB07_MYCMR HAMP domain-containing protein	2	NO_SP	0.998036	0.001979	0.000000	0.000000	0.000000	0.000000	6,3	96,1	909	**7,45**	23	38,5	96,1	8,3
>tr|A0A2Z5YCV2|A0A2Z5YCV2_MYCMR 1-deoxy-D-xylulose-5-phosphate synthase	0	NO_SP	1.000058	0.000000	0.000000	0.000000	0.000000	0.000000	6,0	67,7	637	**7,44**	18	44,9	67,7	4,3
>tr|A0A2Z5Y8F1|A0A2Z5Y8F1_MYCMR Acyl-CoA dehydrogenase	0	NO_SP	1.000063	0.000000	0.000000	0.000000	0.000000	0.000000	6,3	45,1	410	**7,30**	11	38,3	45,1	6,9
>tr|A0A2Z5YEW3|A0A2Z5YEW3_MYCMR Isoleucine--tRNA ligase	0	NO_SP	1.000062	0.000000	0.000000	0.000000	0.000000	0.000000	4,9	119,3	1066	**7,15**	22	30,3	119,3	16,9
>tr|A0A2Z5YBT1|A0A2Z5YBT1_MYCMR LLM class F420-dependent	0	NO_SP	1.000050	0.000001	0.000000	0.000000	0.000000	0.000000	4,5	29,6	275	**7,11**	11	68	29,6	11,3
>tr|A0A2Z5YBC6|A0A2Z5YBC6_MYCMR Uncharacterized protein	0	NO_SP	0.999794	0.000200	0.000008	0.000002	0.000001	0.000001	4,2	49,2	466	**7,07**	11	36,3	49,2	6,3
>sp|B2HFY1|MSHC_MYCMM L-cysteine:1D-myo-inositol 2-amino-2-deoxy-alpha-D-glucopyranoside ligase	0	NO_SP	1.000053	0.000010	0.000001	0.000000	0.000000	0.000000	5,1	44,7	411	**6,95**	14	53,5	44,7	7,3

>tr|B2HIL3|B2HIL3_MYCMM Ketoreductase	0	NO_SP	0.999969	0.000060	0.000003	0.000000	0.000000	0.000000	9,7	41,9	385	6,95	18	52,5	41,9	15,2
>tr|A0A2Z5YJ80|A0A2Z5YJ80_MYCMR Acyl-CoA dehydrogenase	0	NO_SP	1.000065	0.000000	0.000000	0.000000	0.000000	0.000000	5,0	41,4	388	6,94	15	62,1	41,4	5,5
>tr|A0A2Z5YM24|A0A2Z5YM24_MYCMR 6-aminohexanoate-cyclic-dimer hydrolase	0	NO_SP	1.000044	0.000002	0.000000	0.000000	0.000000	0.000000	5,4	52,5	497	6,83	13	45,9	52,5	5,0
>tr|B2HEM8|B2HEM8_MYCMM D-alpha-D-mannose-1-phosphate guanylyltransferase ManB	0	NO_SP	1.000051	0.000000	0.000000	0.000000	0.000000	0.000000	5,2	37,7	358	6,74	14	52	37,7	8,5
>tr|B2HQF7|B2HQF7_MYCMM Ribonuclease PH	0	NO_SP	1.000057	0.000001	0.000000	0.000000	0.000000	0.000000	5,2	28,8	276	6,74	10	55,8	28,8	28,4
>tr|A0A2Z5YNY9|A0A2Z5YNY9_MYCMR Putative coenzyme F420-dependent	0	NO_SP	0.999643	0.000375	0.000015	0.000001	0.000000	0.000001	4,8	37,3	345	6,64	12	50,4	37,3	20,2
>sp|B2HST3|ECCB5_MYCMM ESX-5 secretion system ATPase EccB5	1	NO_SP	0.998405	0.001572	0.000007	0.000001	0.000001	0.000013	6,4	54,1	507	6,54	9	22,9	54,1	14,7
>tr|A0A2Z5YGM0|A0A2Z5YGM0_MYCMR UDP-N-acetylmuramate--L-alanine ligase	0	NO_SP	1.000061	0.000000	0.000000	0.000000	0.000000	0.000000	6,0	49,0	474	6,43	8	30,8	49,0	4,7
>tr|B2HHR6|B2HHR6_MYCMM 3-oxoacyl-[acyl-carrier protein] synthase 1 KasA	0	NO_SP	1.000043	0.000001	0.000000	0.000000	0.000000	0.000000	4,9	43,7	416	6,42	19	69	43,7	6,2
>tr|A0A3E2MZP3|A0A3E2MZP3_MYCMR Acetolactate synthase	0	NO_SP	1.000077	0.000000	0.000000	0.000000	0.000000	0.000000	4,8	64,2	600	6,41	2	3,7	64,2	5,5
>tr|A0A2Z5YHM1|A0A2Z5YHM1_MYCMR F420-dependent	0	NO_SP	1.000088	0.000000	0.000000	0.000000	0.000000	0.000000	6,1	30,3	276	6,41	11	63,4	30,3	5,9
>tr|B2HQZ7|B2HQZ7_MYCMM Ribosomal protein S1 RpsA	0	NO_SP	1.000066	0.000000	0.000000	0.000000	0.000000	0.000000	4,5	53,1	481	6,38	32	62,6	53,1	9,1
>tr|A0A2Z5YNM3|A0A2Z5YNM3_MYCMR Putative arabinosyltransferase A	12	SP	0.002013	0.997102	0.000233	0.000225	0.000194	0.000208	9,8	116,2	1094	6,36	14	23,1	116,2	5,3
>tr|A0A2Z5YAP9|A0A2Z5YAP9_MYCMR Putative	0	NO_SP	0.998677	0.001325	0.000005	0.000002	0.000001	0.000002	5,1	38,5	370	6,35	15	69,5	38,5	6,4
>tr|A0A3E2MS33|A0A3E2MS33_MYCMR Putative decaprenylphosphoryl-beta-D-ribose	0	NO_SP	0.992496	0.007507	0.000031	0.000007	0.000004	0.000007	6,2	50,2	463	6,34	16	53,1	50,2	6,1
>tr|A0A2Z5YKL3|A0A2Z5YKL3_MYCMR Polyketide synthase	0	NO_SP	1.000040	0.000008	0.000000	0.000000	0.000000	0.000000	4,9	58,5	544	6,34	17	47,2	58,5	5,0
>tr|A0A2Z5YH52|A0A2Z5YH52_MYCMR Short-chain dehydrogenase	0	NO_SP	1.000039	0.000002	0.000000	0.000000	0.000000	0.000000	9,1	28,8	275	6,29	7	34,5	28,8	6,5
>tr|A0A100I200|A0A100I200_9MYCO NADH-dependent glutamate synthase	0	NO_SP	1.000034	0.000000	0.000000	0.000000	0.000000	0.000000	4,7	50,6	466	6,21	17	53,6	50,6	7,7
>tr|A0A3E2N046|A0A3E2N046_MYCMR ESX-1 secretion system protein eccB1	1	NO_SP	1.000065	0.000000	0.000000	0.000000	0.000000	0.000000	6,7	55,6	533	6,19	15	49,9	55,6	4,7
>tr|A0A3E2MYG7|A0A3E2MYG7_MYCMR Putative acetyl-CoA acetyltransferase	0	NO_SP	1.000070	0.000000	0.000000	0.000000	0.000000	0.000000	4,7	40,5	393	6,19	1	4,3	40,5	12,2
>tr|A0A2Z5YAY3|A0A2Z5YAY3_MYCMR NAD(P)H-quinone dehydrogenase	0	NO_SP	1.000037	0.000000	0.000000	0.000000	0.000000	0.000000	6,4	49,3	470	6,17	17	61,5	49,3	9,0
>tr|B2HQL7|B2HQL7_MYCMM Threonine synthase	0	NO_SP	1.000039	0.000000	0.000000	0.000000	0.000000	0.000000	6,6	37,4	360	6,16	17	75,3	37,4	3,9
>tr|A0A2Z5YE99|A0A2Z5YE99_MYCMR DNA polymerase I	0	NO_SP	1.000020	0.000000	0.000000	0.000000	0.000000	0.000000	4,7	98,1	899	6,16	29	48,1	98,1	6,8
>tr|A0A2Z5YEL3|A0A2Z5YEL3_MYCMR Phenylalanine--tRNA ligase beta subunit	0	NO_SP	1.000050	0.000000	0.000000	0.000000	0.000000	0.000000	4,8	88,0	829	6,01	26	52,5	88,0	4,8
>tr|A0A2Z5YEQ1|A0A2Z5YEQ1_MYCMR Argininosuccinate synthase	0	NO_SP	1.000044	0.000000	0.000000	0.000000	0.000000	0.000000	4,8	43,9	398	5,97	21	71,6	43,9	4,8
>tr|A0A3E2MZV5|A0A3E2MZV5_MYCMR Doxorubicin resistance ATP-binding protein DrrA	0	NO_SP	1.000053	0.000000	0.000000	0.000000	0.000000	0.000000	5,9	35,8	331	5,94	23	71,6	35,8	4,1
>sp|B2HQV4|GPMA_MYCMM 2,3-bisphosphoglycerate-dependent phosphoglycerate mutase	0	NO_SP	1.000068	0.000001	0.000000	0.000000	0.000000	0.000000	5,0	27,5	251	5,93	16	72,9	27,5	8,7
>tr|A0A117DTI1|A0A117DTI1_9MYCO L-threonine dehydratase	0	NO_SP	1.000047	0.000000	0.000000	0.000000	0.000000	0.000000	4,4	45,0	426	5,91	10	45,8	45,0	4,8
>tr|B2HKV8|B2HKV8_MYCMM Conserved protein	0	NO_SP	1.000046	0.000004	0.000000	0.000000	0.000000	0.000000	8,5	16,7	154	5,90	10	74	16,7	12,8
>tr|A0A2Z5YDG4|A0A2Z5YDG4_MYCMR Pyridoxal 5'-phosphate synthase subunit PdxS	0	NO_SP	1.000033	0.000000	0.000000	0.000000	0.000000	0.000000	5,1	33,4	317	5,90	11	57,1	33,4	9,4
>sp|B2HRU9|DER_MYCMM GTPase Der	0	NO_SP	1.000075	0.000000	0.000000	0.000000	0.000000	0.000000	9,2	50,9	469	5,86	16	51,4	50,9	10,8
>sp|B2HSL3|EFTU_MYCMM Elongation factor Tu	0	NO_SP	1.000068	0.000000	0.000000	0.000000	0.000000	0.000000	5,0	43,8	396	5,78	26	88,4	43,8	5,7
>tr|A0A100I0G8|A0A100I0G8_9MYCO Serine--tRNA ligase	0	NO_SP	1.000036	0.000000	0.000000	0.000000	0.000000	0.000000	4,8	45,3	419	5,70	12	39,9	45,3	7,1
>tr|A0A3E2N045|A0A3E2N045_MYCMR ESX-1 secretion system protein EccA1	0	NO_SP	1.000066	0.000000	0.000000	0.000000	0.000000	0.000000	4,8	64,9	599	5,67	17	49,6	64,7	10,6
>tr|A0A2Z5YNJ0|A0A2Z5YNJ0_MYCMR Putative metallophosphoesterase	1	NO_SP	0.996330	0.003611	0.000030	0.000018	0.000009	0.000017	10,0	36,4	337	5,64	14	62,3	36,4	3,8
>tr|A0A2Z5YJI1|A0A2Z5YJI1_MYCMR Thioredoxin domain-containing protein	0	NO_SP	1.000040	0.000001	0.000000	0.000000	0.000000	0.000000	4,3	33,2	314	5,63	11	65,9	33,2	11,6
>tr|A0A2Z5YDT5|A0A2Z5YDT5_MYCMR Quinone reductase	0	NO_SP	1.000066	0.000000	0.000000	0.000000	0.000000	0.000000	5,0	34,4	336	5,63	3	18,8	34,4	3,2
>tr|B2HSY6|B2HSY6_MYCMM 6-phosphogluconate dehydrogenase, decarboxylating	0	NO_SP	1.000082	0.000000	0.000000	0.000000	0.000000	0.000000	5,2	51,4	483	5,62	18	49,1	51,4	9,9
>tr|A0A100I0J2|A0A100I0J2_9MYCO Integral membrane indolylacetylinositol arabinosyltransferase	14	NO_SP	0.681160	0.317676	0.000297	0.000317	0.000209	0.000361	9,8	115,0	1073	5,57	12	18,8	115,0	7,4
>sp|B2HQX9|TRPA_MYCMM Tryptophan synthase alpha chain	0	NO_SP	1.000062	0.000001	0.000000	0.000000	0.000000	0.000000	4,6	28,0	269	5,56	11	65,8	28,0	6,3
>sp|B2HRM4|FABH_MYCMM 3-oxoacyl-[acyl-carrier-protein] synthase 3	0	NO_SP	0.999987	0.000067	0.000000	0.000000	0.000000	0.000000	4,5	34,8	335	5,55	9	51,6	34,8	8,0
>tr|A0A3E2MZU6|A0A3E2MZU6_MYCMR p-hydroxybenzoic acid--AMP ligase FadD22	0	NO_SP	1.000032	0.000009	0.000000	0.000000	0.000000	0.000000	4,6	75,2	702	5,49	23	54,6	75,1	4,6
>tr|B2HJQ1|B2HJQ1_MYCMM 4-hydroxy-3-methylbut-2-en-1-yl diphosphate synthase (flavodoxin)	0	NO_SP	1.000024	0.000014	0.000000	0.000000	0.000000	0.000000	4,9	40,6	387	5,47	16	68,5	40,6	3,5
>tr|A0A2Z5YNU7|A0A2Z5YNU7_MYCMR DNA integrity scanning protein DisA	0	NO_SP	1.000057	0.000000	0.000000	0.000000	0.000000	0.000000	4,9	39,1	358	5,38	10	50,8	39,1	5,8
>tr|A0A3E2MTE3|A0A3E2MTE3_MYCMR UPF0336 protein DAVIS_03422	0	NO_SP	1.000050	0.000007	0.000000	0.000000	0.000000	0.000000	4,3	17,6	159	5,36	10	84,9	17,6	6,6
>tr|B2HJW1|B2HJW1_MYCMM Heat shock protein HspX_1	0	NO_SP	1.000061	0.000001	0.000000	0.000000	0.000000	0.000000	4,7	15,9	143	5,34	7	49	15,9	6,6
**>tr|A0A2Z5Y992|A0A2Z5Y992_MYCMR Biotin carboxylase**	0	NO_SP	1.000072	0.000000	0.000000	0.000000	0.000000	0.000000	4,7	46,2	446	5,33	13	47,3	46,2	5,0
>tr|B2HQB7|B2HQB7_MYCMM Peroxidoxin BcpB	0	NO_SP	1.000013	0.000040	0.000001	0.000000	0.000000	0.000000	7,2	16,5	152	5,30	5	36,8	16,5	7,5
>tr|A0A2Z5Y7E0|A0A2Z5Y7E0_MYCMR Beta sliding clamp	0	NO_SP	1.000039	0.000021	0.000001	0.000000	0.000000	0.000000	4,4	40,5	385	5,30	10	43,6	40,5	7,7
>tr|B2HD34|B2HD34_MYCMM	0	NO_SP	0.999348	0.000635	0.000041	0.000002	0.000001	0.000004	5,2	33,4	307	5,28	13	48,5	33,4	3,5
>tr|A0A100I9T1|A0A100I9T1_9MYCO Inositol-3-phosphate synthase	0	NO_SP	0.999948	0.000121	0.000000	0.000000	0.000000	0.000000	5,0	40,4	372	5,28	21	54,8	40,4	11,7
>tr|A0A2Z5YGI2|A0A2Z5YGI2_MYCMR ATP phosphoribosyltransferase	0	NO_SP	1.000061	0.000001	0.000000	0.000000	0.000000	0.000000	4,9	30,6	284	5,23	13	57,4	30,6	18,3
>sp|B2HDS6|METXA_MYCMM Homoserine O-acetyltransferase	0	NO_SP	1.000051	0.000001	0.000000	0.000000	0.000000	0.000000	5,8	40,2	379	5,09	14	58,8	40,2	3,2
>tr|B2HM79|B2HM79_MYCMM Chaperone protein DnaJ	0	NO_SP	1.000071	0.000000	0.000000	0.000000	0.000000	0.000000	6,7	40,0	378	5,07	13	38,6	40,0	9,6
>tr|A0A2Z5YKW6|A0A2Z5YKW6_MYCMR Aldehyde-alcohol dehydrogenase	0	NO_SP	0.998906	0.001111	0.000006	0.000002	0.000001	0.000002	4,9	95,1	889	5,07	31	60,9	95,1	10,0
>tr|A0A2Z5YNF4|A0A2Z5YNF4_MYCMR Hydrolase	0	NO_SP	1.000048	0.000004	0.000000	0.000000	0.000000	0.000000	4,7	68,6	624	5,06	16	39,6	68,6	4,5
>tr|B2HDS7|B2HDS7_MYCMM O-acetylhomoserine sulfhydrylase MetC	0	NO_SP	1.000003	0.000022	0.000001	0.000000	0.000000	0.000000	5,2	47,4	449	5,00	20	92,9	47,4	11,5
>sp|B2HIH4|SYE_MYCMM Glutamate--tRNA ligase	0	NO_SP	1.000038	0.000001	0.000000	0.000000	0.000000	0.000000	5,1	53,7	489	4,98	1	3,1	53,7	8,7
>tr|B2HLK1|B2HLK1_MYCMM Enoyl-CoA hydratase, EchA21	0	NO_SP	1.000027	0.000000	0.000000	0.000000	0.000000	0.000000	4,8	29,0	274	4,95	10	52,9	29,0	4,1
>tr|B2HF44|B2HF44_MYCMM Polyketide synthase, Pks12	0	NO_SP	1.000035	0.000006	0.000000	0.000000	0.000000	0.000000	4,7	434,3	4187	4,94	2	0,7	434,3	10,4
>tr|A0A2Z5YDP4|A0A2Z5YDP4_MYCMR TGc domain-containing protein	0	NO_SP	1.000045	0.000000	0.000000	0.000000	0.000000	0.000000	5,0	121,9	1111	4,90	36	53,8	121,9	4,1
>tr|A0A2Z5YCS7|A0A2Z5YCS7_MYCMR Alanine dehydrogenase	0	NO_SP	1.000023	0.000004	0.000000	0.000000	0.000000	0.000000	6,1	38,6	371	4,90	22	78,2	38,6	10,2
>tr|B2HKR4|B2HKR4_MYCMM Multifunctional enzyme siroheme synthase CysG	0	NO_SP	1.000058	0.000000	0.000000	0.000000	0.000000	0.000000	6,6	42,0	401	4,88	3	5,7	42,0	5,9
>tr|A0A2Z5Y9I1|A0A2Z5Y9I1_MYCMR NADH dehydrogenase NdhA	1	NO_SP	1.000058	0.000004	0.000000	0.000000	0.000000	0.000000	8,6	50,8	471	4,85	17	51,2	50,8	6,7
>tr|A0A2Z5Y8F9|A0A2Z5Y8F9_MYCMR NAD(P) transhydrogenase subunit alpha	0	NO_SP	0.999244	0.000780	0.000005	0.000001	0.000001	0.000001	4,4	37,3	362	4,85	15	67,7	37,3	3,6
>tr|A0A2Z5YAI5|A0A2Z5YAI5_MYCMR Transcription termination/antitermination protein NusG	0	NO_SP	1.000056	0.000000	0.000000	0.000000	0.000000	0.000000	4,4	25,6	237	4,83	12	65,8	25,6	4,8
>tr|A0A2Z5Y896|A0A2Z5Y896_MYCMR Acyl-CoA synthetase	0	NO_SP	1.000045	0.000000	0.000015	0.000000	0.000000	0.000000	6,0	56,8	544	4,82	14	50,4	56,8	7,5
>tr|A0A2Z5YH83|A0A2Z5YH83_MYCMR 3-oxoacyl-[acyl-carrier-protein] synthase 2	0	NO_SP	0.998409	0.001623	0.000017	0.000003	0.000001	0.000002	5,8	44,2	417	4,81	20	80,1	44,2	9,5
>tr|B2HP49|B2HP49_MYCMM Coenzyme A biosynthesis bifunctional protein CoaBC	0	NO_SP	1.000041	0.000001	0.000000	0.000000	0.000000	0.000000	6,8	43,4	413	4,80	14	56,7	43,4	5,5
>tr|A0A2Z5YC91|A0A2Z5YC91_MYCMR Long-chain-fatty-acid--AMP ligase FadD29	0	NO_SP	1.000048	0.000001	0.000000	0.000000	0.000000	0.000000	5,2	67,7	622	4,72	20	45,2	67,7	11,4
>tr|A0A2Z5Y8S5|A0A2Z5Y8S5_MYCMR Fumarate reductase	0	NO_SP	1.000053	0.000000	0.000000	0.000000	0.000000	0.000000	6,9	28,6	248	4,66	15	70,2	28,6	16,8
>tr|A0A2Z5Y835|A0A2Z5Y835_MYCMR Ketoacyl reductase	0	NO_SP	1.000045	0.000000	0.000000	0.000000	0.000000	0.000000	7,4	27,6	267	4,66	10	65,2	27,6	4,6
>tr|A0A3E2MY80|A0A3E2MY80_MYCMR Serine hydroxymethyltransferase	0	NO_SP	0.999136	0.000873	0.000014	0.000002	0.000001	0.000001	6,6	49,8	471	4,65	12	48,2	49,8	15,1
>tr|B2HKD3|B2HKD3_MYCMM 2-isopropylmalate synthase	0	NO_SP	1.000081	0.000000	0.000000	0.000000	0.000000	0.000000	4,6	66,1	606	4,63	2	5,9	66,1	10,2

>tr|A0A3E2MSW2|A0A3E2MSW2_MYCMR Putative acyltransferase	0	NO_SP	1.000054	0.000000	0.000000	0.000000	0.000000	0.000000	4,6	48,0	456	4,62	4	18,6	48,0	7,4
>tr|B2HJX9|B2HJX9_MYCMM Uncharacterized protein	0	NO_SP	1.000031	0.000011	0.000000	0.000000	0.000000	0.000000	6,2	35,7	333	4,60	11	58	35,7	4,8
>tr|A0A3E2MWX0|A0A3E2MWX0_MYCMR Proline--tRNA ligase	0	NO_SP	1.000039	0.000000	0.000000	0.000000	0.000000	0.000000	4,7	63,1	578	4,59	26	58,3	63,1	4,6
>tr|A0A2Z5YC93|A0A2Z5YC93_MYCMR Electron transfer flavoprotein subunit alpha	0	NO_SP	1.000041	0.000000	0.000000	0.000000	0.000000	0.000000	4,4	31,7	318	4,56	14	77	31,7	5,9
>tr|A0A2Z5YCW9|A0A2Z5YCW9_MYCMR Glutamyl-tRNA(Gln) amidotransferase subunit A	0	NO_SP	1.000026	0.000009	0.000000	0.000000	0.000000	0.000000	4,7	51,3	493	4,54	12	42,8	51,3	5,6
>tr|B2HCV1|B2HCV1_MYCMM DNA topoisomerase (ATP-hydrolyzing)	0	NO_SP	1.000037	0.000002	0.000000	0.000000	0.000000	0.000000	4,6	23,7	221	4,53	15	86,9	23,7	8,7
>tr|A0A3E2MRB3|A0A3E2MRB3_MYCMR Apolipoprotein N-acyltransferase	8	NO_SP	1.000035	0.000000	0.000000	0.000000	0.000000	0.000000	4,6	73,6	692	4,52	9	24,4	73,6	5,3
>tr|A0A3E2N0V3|A0A3E2N0V3_MYCMR Uncharacterized protein	1	LIPO	0.000000	0.000040	1.000013	0.000000	0.000000	0.000000	4,7	52,3	487	4,50	9	31,2	52,3	5,4
>tr|A0A3E2MVF9|A0A3E2MVF9_MYCMR Phosphoribosyl isomerase A	0	NO_SP	0.999991	0.000046	0.000001	0.000000	0.000000	0.000000	4,5	25,5	244	4,50	11	68,4	25,5	9,1
>tr|A0A2Z5YI92|A0A2Z5YI92_MYCMR Diacylglycerol O-acyltransferase	0	NO_SP	1.000047	0.000000	0.000000	0.000000	0.000000	0.000000	6,1	49,8	463	4,49	10	39,3	49,8	6,4
>tr|A0A3E2MUB7|A0A3E2MUB7_MYCMR Histidine kinase	2	NO_SP	0.859892	0.001034	0.000371	0.000010	0.000005	0.138706	5,8	55,6	514	4,49	15	49	55,6	5,3
>tr|A0A2Z5YHT4|A0A2Z5YHT4_MYCMR Pribosyltran domain-containing protein	0	NO_SP	1.000057	0.000000	0.000000	0.000000	0.000000	0.000000	5,2	71,6	656	4,48	2	5,3	71,6	6,2
>tr|A0A2Z5YNG5|A0A2Z5YNG5_MYCMR Putative acyl-[acyl-carrier-protein] desaturase DesA1	0	NO_SP	1.000045	0.000013	0.000000	0.000000	0.000000	0.000000	6,5	39,0	338	4,47	14	60,1	39,0	5,2
>tr|A0A2Z5YDP2|A0A2Z5YDP2_MYCMR Glyceraldehyde-3-phosphate dehydrogenase	0	NO_SP	1.000045	0.000000	0.000000	0.000000	0.000000	0.000000	5,2	36,9	346	4,44	14	65,3	36,9	3,9
>tr|A0A2Z5YLQ5|A0A2Z5YLQ5_MYCMR Putative enoyl-CoA hydratase echA8	0	NO_SP	1.000055	0.000000	0.000000	0.000000	0.000000	0.000000	5,0	27,1	257	4,39	7	31,1	27,1	5,4
>tr|B2HR07|B2HR07_MYCMM UvrABC system protein A	0	NO_SP	1.000070	0.000000	0.000000	0.000000	0.000000	0.000000	6,5	106,3	971	4,35	22	34,7	106,3	5,6
>tr|A0A2Z5YM82|A0A2Z5YM82_MYCMR DNA-binding response regulator	0	NO_SP	1.000049	0.000000	0.000000	0.000000	0.000000	0.000000	5,4	26,7	240	4,35	12	69,2	26,7	5,1
>tr|A0A2Z5YDG9|A0A2Z5YDG9_MYCMR 35 kDa protein	0	NO_SP	1.000048	0.000000	0.000000	0.000000	0.000000	0.000000	6,1	29,2	271	4,30	17	85,2	29,2	3,9
>tr|B2HN36|B2HN36_MYCMM Succinate dehydrogenase (Iron-sulfur subunit), SdhA_1	0	NO_SP	1.000061	0.000002	0.000000	0.000000	0.000000	0.000000	5,8	70,5	642	4,29	26	50,5	70,5	10,0
>tr|B2HHU8|B2HHU8_MYCMM Conserved hypothetical	0	NO_SP	1.000038	0.000008	0.000000	0.000000	0.000000	0.000000	4,5	41,9	388	4,28	14	47,7	41,9	16,0
>tr|B2HDV2|B2HDV2_MYCMM Probable allantoicase	0	NO_SP	1.000036	0.000001	0.000000	0.000000	0.000000	0.000000	5,7	35,0	319	4,27	12	44,8	35,0	3,2
>tr|A0A2Z5Y9R4|A0A2Z5Y9R4_MYCMR UPF0336 protein MMRN_08410	0	NO_SP	1.000006	0.000047	0.000001	0.000000	0.000000	0.000000	5,0	18,3	166	4,26	6	54,8	18,3	6,8
>tr|A0A2Z5YJS3|A0A2Z5YJS3_MYCMR Adenylate/guanylate cyclase domain-containing protein	2	NO_SP	0.999953	0.000047	0.000000	0.000000	0.000000	0.000000	4,8	79,7	730	4,23	15	32,1	79,7	6,0
>tr|A0A3E2N158|A0A3E2N158_MYCMR Glutamate-pyruvate aminotransferase AlaA	0	NO_SP	1.000056	0.000000	0.000000	0.000000	0.000000	0.000000	4,9	47,0	423	4,22	11	43,3	47,0	6,2
>tr|A0A2Z5YKQ1|A0A2Z5YKQ1_MYCMR 3-hydroxyisobutyryl-CoA hydrolase	0	NO_SP	1.000060	0.000000	0.000000	0.000000	0.000000	0.000000	4,5	36,7	349	4,20	11	42,7	36,7	6,4
>tr|A0A2Z5YPI5|A0A2Z5YPI5_MYCMR Aspartokinase	0	NO_SP	1.000069	0.000000	0.000000	0.000000	0.000000	0.000000	4,8	44,4	421	4,20	13	46,6	44,4	10,0
>tr|A0A100IF67|A0A100IF67_9MYCO 3-hydroxyacyl-CoA dehydrogenase	0	NO_SP	1.000033	0.000007	0.000000	0.000000	0.000000	0.000000	5,4	26,3	253	4,15	16	78,3	26,3	7,2
>tr|A0A2Z5YPL5|A0A2Z5YPL5_MYCMR Thioredoxin reductase	0	NO_SP	1.000000	0.000035	0.000001	0.000000	0.000000	0.000000	4,4	35,2	332	4,14	12	65,4	35,2	10,2
>tr|A0A3E2MQF7|A0A3E2MQF7_MYCMR Ribosomal RNA small subunit methyltransferase B	0	NO_SP	1.000036	0.000002	0.000000	0.000000	0.000000	0.000000	7,0	48,7	457	4,13	15	50,3	48,6	4,2
>tr|A0A2Z5Y7T5|A0A2Z5Y7T5_MYCMR LLM class F420-dependent	0	NO_SP	1.000025	0.000000	0.000000	0.000000	0.000000	0.000000	5,5	37,5	346	4,13	16	71,1	37,5	10,0
>tr|B2HJ41|B2HJ41_MYCMM ATP-dependent protease ATP-binding subunit ClpC1	0	NO_SP	1.000073	0.000001	0.000000	0.000000	0.000000	0.000000	5,6	93,7	848	4,08	48	68,6	93,7	8,0
>tr|A0A100I697|A0A100I697_9MYCO O-phosphoserine phosphohydrolase	0	NO_SP	1.000053	0.000000	0.000000	0.000000	0.000000	0.000000	4,4	42,9	405	4,08	10	38,3	42,9	4,8
>tr|B2HHG7|B2HHG7_MYCMM NADP-dependent alcohol dehydrogenase Adh	0	NO_SP	1.000071	0.000000	0.000000	0.000000	0.000000	0.000000	5,4	37,2	346	4,08	20	59,5	37,2	5,4
>tr|A0A2Z5YH98|A0A2Z5YH98_MYCMR AAA ATPase forming ring-shaped complexes	0	NO_SP	1.000064	0.000000	0.000000	0.000000	0.000000	0.000000	4,5	67,5	609	4,07	19	49,8	67,5	11,7
>tr|A0A2Z5YHY7|A0A2Z5YHY7_MYCMR Universal stress protein	0	NO_SP	1.000063	0.000000	0.000000	0.000000	0.000000	0.000000	4,9	31,8	298	4,06	12	63,4	31,8	6,3
>sp|B2HNC6|AROC_MYCMM Chorismate synthase	0	NO_SP	1.000023	0.000027	0.000003	0.000000	0.000000	0.000000	6,0	42,5	407	4,03	17	73,2	42,5	6,2
>tr|A0A2Z5YIA6|A0A2Z5YIA6_MYCMR CP_ATPgrasp_1 domain-containing protein	0	NO_SP	1.000069	0.000000	0.000000	0.000000	0.000000	0.000000	5,1	57,3	514	4,02	1	6	57,3	7,8
>tr|A0A2Z5YCN7|A0A2Z5YCN7_MYCMR DNA translocase FtsK	6	NO_SP	0.999712	0.000288	0.000000	0.000000	0.000000	0.000000	4,8	86,1	806	4,02	11	18,6	86,1	8,9
>tr|B2HQ15|B2HQ15_MYCMM UDP-glucose 6-dehydrogenase, UdgL	0	NO_SP	1.000062	0.000001	0.000000	0.000000	0.000000	0.000000	5,4	46,2	418	4,01	15	53,3	46,2	5,4
>tr|A0A2Z5YHU6|A0A2Z5YHU6_MYCMR Cysteine synthase	0	NO_SP	1.000066	0.000000	0.000000	0.000000	0.000000	0.000000	4,7	32,5	310	3,98	17	76,1	32,5	8,5
>tr|B2HK30|B2HK30_MYCMM Saccharopine dehydrogenase	0	NO_SP	0.999271	0.000554	0.000203	0.000002	0.000001	0.000001	4,6	44,0	405	3,95	11	39,8	44,0	5,9
>tr|A0A2Z5YLN8|A0A2Z5YLN8_MYCMR Bifunctional purine biosynthesis protein PurH	0	NO_SP	1.000049	0.000000	0.000000	0.000000	0.000000	0.000000	5,6	55,5	523	3,94	15	54,3	55,5	5,0
>tr|A0A2Z5YEI8|A0A2Z5YEI8_MYCMR Triosephosphate isomerase	0	NO_SP	1.000063	0.000000	0.000000	0.000000	0.000000	0.000000	5,3	27,3	261	3,93	17	80,5	27,3	17,3
>tr|A0A2Z5YKQ5|A0A2Z5YKQ5_MYCMR Diaminopimelate decarboxylase	0	NO_SP	0.991561	0.008129	0.000218	0.000031	0.000014	0.000064	5,0	50,4	472	3,91	16	54	50,4	7,5
>tr|B2HI01|B2HI01_MYCMM Acetolactate synthase	0	NO_SP	1.000065	0.000001	0.000000	0.000000	0.000000	0.000000	4,7	52,4	515	3,91	14	47,4	52,4	11,0
>tr|A0A2Z5Y887|A0A2Z5Y887_MYCMR Haloacid dehalogenase	3	NO_SP	0.991236	0.008692	0.000066	0.000015	0.000006	0.000012	5,0	166,9	1620	3,91	23	26,7	166,9	3,4
>tr|B2HPV3|B2HPV3_MYCMM Fructose-bisphosphate aldolase	0	NO_SP	1.000063	0.000000	0.000000	0.000000	0.000000	0.000000	4,8	36,6	344	3,91	16	79,1	36,6	5,4
>sp|B2HCV5|KAD_MYCMM Adenylate kinase	0	NO_SP	1.000068	0.000000	0.000000	0.000000	0.000000	0.000000	4,7	20,1	181	3,89	9	62,4	20,1	3,5
>tr|A0A2Z5YCF8|A0A2Z5YCF8_MYCMR Thioredoxin-like_fold domain-containing protein	1	NO_SP	1.000058	0.000003	0.000000	0.000000	0.000000	0.000000	6,2	27,1	255	3,85	13	63,1	27,1	4,2
>tr|A0A2Z5YCQ5|A0A2Z5YCQ5_MYCMR Polyphosphate kinase	0	NO_SP	0.998429	0.001605	0.000003	0.000002	0.000001	0.000002	5,6	81,4	731	3,85	22	49,1	81,4	4,9
>tr|A0A117DW44|A0A117DW44_9MYCO Chaperonin GroEL2	0	NO_SP	1.000098	0.000000	0.000000	0.000000	0.000000	0.000000	4,6	56,5	541	3,83	0	0	56,5	10,1
>tr|A0A2Z5YGC2|A0A2Z5YGC2_MYCMR Chromosome partition protein Smc	0	NO_SP	1.000030	0.000006	0.000000	0.000000	0.000000	0.000000	5,0	125,0	1120	3,83	36	43,5	125,0	4,3
>tr|B2HQT1|B2HQT1_MYCMM Uncharacterized protein	0	NO_SP	1.000083	0.000000	0.000000	0.000000	0.000000	0.000000	6,0	30,2	264	3,82	7	34,8	30,2	14,5
>sp|B2HNC1|SYA_MYCMM Alanine--tRNA ligase	0	NO_SP	1.000075	0.000000	0.000000	0.000000	0.000000	0.000000	5,2	96,5	901	3,79	1	1,3	96,5	9,9
>tr|A0A100I2N1|A0A100I2N1_9MYCO Phosphoserine aminotransferase	0	NO_SP	1.000053	0.000001	0.000000	0.000000	0.000000	0.000000	4,5	40,0	376	3,79	2	5,3	40,0	8,1
>tr|A0A2Z5YC17|A0A2Z5YC17_MYCMR Putative	0	NO_SP	1.000055	0.000008	0.000000	0.000000	0.000000	0.000000	4,5	29,8	281	3,79	10	44,8	29,8	3,6
>tr|A0A2Z5YAQ5|A0A2Z5YAQ5_MYCMR Putative glycosyl hydrolase	0	NO_SP	1.000087	0.000001	0.000000	0.000000	0.000000	0.000000	5,5	88,4	794	3,77	22	45,7	88,4	4,9
>tr|A0A2Z5YJ26|A0A2Z5YJ26_MYCMR 2-oxoglutarate	0	NO_SP	1.000052	0.000004	0.000000	0.000000	0.000000	0.000000	4,9	69,2	653	3,77	22	53,3	69,2	3,0
>tr|A0A2Z5YJX9|A0A2Z5YJX9_MYCMR ATP-dependent Clp protease ATP-binding subunit ClpX	0	NO_SP	1.000051	0.000000	0.000000	0.000000	0.000000	0.000000	4,8	46,8	426	3,76	20	57	46,8	6,0
>tr|A0A2Z5YJV6|A0A2Z5YJV6_MYCMR Glycogen phosphorylase	0	NO_SP	1.000057	0.000001	0.000000	0.000000	0.000000	0.000000	5,3	96,3	867	3,76	21	40,9	96,3	5,6
>tr|A0A2Z5YH96|A0A2Z5YH96_MYCMR Bifunctional glutamine synthetase adenylyltransferase	0	NO_SP	1.000043	0.000004	0.000000	0.000000	0.000000	0.000000	7,0	108,6	995	3,73	20	31,7	108,6	4,7
>tr|A0A2Z5YJX8|A0A2Z5YJX8_MYCMR Uncharacterized protein	0	NO_SP	1.000043	0.000000	0.000000	0.000000	0.000000	0.000000	4,8	17,9	163	3,68	7	66,9	17,9	17,6
>tr|A0A2Z5YPB5|A0A2Z5YPB5_MYCMR ESAT-6-like protein	0	NO_SP	1.000045	0.000000	0.000000	0.000000	0.000000	0.000000	4,4	10,0	95	3,64	5	93,7	10,0	10,2
>tr|A0A2Z5YDH4|A0A2Z5YDH4_MYCMR Spermidine/putrescine ABC transporter ATP-binding protein	0	NO_SP	1.000036	0.000000	0.000000	0.000000	0.000000	0.000000	4,8	34,3	319	3,63	9	44,5	34,3	14,6
>tr|B2HGR3|B2HGR3_MYCMM Cell division protein FtsZ	0	NO_SP	1.000068	0.000001	0.000000	0.000000	0.000000	0.000000	4,3	39,2	386	3,63	17	64,2	39,2	13,0
>tr|A0A3E2MZE1|A0A3E2MZE1_MYCMR Metallo-beta-lactamase superfamily protein	0	NO_SP	1.000040	0.000000	0.000000	0.000000	0.000000	0.000000	5,0	68,2	626	3,62	12	34	68,2	4,8
>tr|A0A2Z5YK22|A0A2Z5YK22_MYCMR Acetyl-/propionyl-CoA carboxylase subunit beta	0	NO_SP	1.000055	0.000007	0.000000	0.000000	0.000000	0.000000	6,3	56,5	526	3,59	2	6,7	56,5	8,0
>tr|A0A2Z5YMC1|A0A2Z5YMC1_MYCMR Phosphate-binding protein PstS	0	NO_SP	1.000051	0.000003	0.000000	0.000000	0.000000	0.000000	4,7	33,8	329	3,59	10	50,8	33,8	2,8
>tr|B2HHK6|B2HHK6_MYCMM Multimeric flavodoxin WrbA	0	NO_SP	1.000064	0.000000	0.000000	0.000000	0.000000	0.000000	5,0	27,5	250	3,57	14	55,6	27,5	4,1
>tr|A0A2Z5YB44|A0A2Z5YB44_MYCMR ATP-dependent RNA helicase RhlE	0	NO_SP	1.000060	0.000001	0.000000	0.000000	0.000000	0.000000	8,6	55,4	517	3,57	15	55,7	55,4	4,7
>tr|B2HGG3|B2HGG3_MYCMM NADPH quinone	0	NO_SP	1.000060	0.000004	0.000000	0.000000	0.000000	0.000000	5,2	33,3	323	3,57	8	33,4	33,3	8,8
>tr|B2HIN0|B2HIN0_MYCMM Phenolphthiocerol synthesis type-I polyketide synthase PpsB	0	NO_SP	0.987298	0.012357	0.000213	0.000051	0.000026	0.000076	4,7	161,7	1524	3,56	0	0	161,7	5,6
>tr|A0A2Z5YK73|A0A2Z5YK73_MYCMR Transcription termination factor Rho	0	NO_SP	0.999852	0.000166	0.000011	0.000001	0.000000	0.000007	5,9	66,9	621	3,54	41	69,4	66,9	11,2
>tr|A0A2Z5YLX4|A0A2Z5YLX4_MYCMR Phosphoribosylformylglycinamidine synthase subunit PurL	0	NO_SP	1.000044	0.000003	0.000000	0.000000	0.000000	0.000000	4,5	80,6	765	3,53	21	40,5	80,6	6,6

>tr|A0A2Z5YCC6|A0A2Z5YCC6_MYCMR Universal stress protein	0	NO_SP	1.000052	0.000001	0.000000	0.000000	0.000000	0.000000	8,4	27,8	264	**3,51**	1	6,8	27,8	29,6
>tr|B2HF73|B2HF73_MYCMM FAD_binding_2 domain-containing protein	0	NO_SP	1.000007	0.000034	0.000003	0.000000	0.000000	0.000000	6,8	60,6	556	**3,50**	18	38,8	60,6	4,8
>tr|B2HGQ8|B2HGQ8_MYCMM Antigen 84	0	NO_SP	1.000060	0.000001	0.000000	0.000000	0.000000	0.000000	4,4	28,7	264	**3,46**	15	59,5	28,7	6,5
>tr|B2HMG4|B2HMG4_MYCMM Ribonuclease E Rne	0	NO_SP	1.000049	0.000000	0.000000	0.000000	0.000000	0.000000	4,1	110,2	1017	**3,46**	3	3,2	110,2	26,8
>tr|A0A2Z5YPK8|A0A2Z5YPK8_MYCMR ESX-1 secretion system protein eccB1	1	NO_SP	0.999964	0.000051	0.000000	0.000000	0.000000	0.000000	6,5	51,3	481	**3,45**	10	38,7	51,3	5,1
>tr|A0A2Z5YCX2|A0A2Z5YCX2_MYCMR Transcription termination/antitermination protein NusA	0	NO_SP	1.000046	0.000000	0.000000	0.000000	0.000000	0.000000	6,3	37,9	347	**3,44**	12	44,1	37,9	15,2
>tr|B2HIN1|B2HIN1_MYCMM Phenolphthiocerol synthesis type-I polyketide synthase PpsA	0	NO_SP	1.000057	0.000001	0.000000	0.000000	0.000000	0.000000	4,8	169,7	1602	**3,44**	1	1,9	169,8	3,4
>tr|A0A2Z5YJ61|A0A2Z5YJ61_MYCMR Energy-dependent translational throttle protein EttA	0	NO_SP	1.000051	0.000000	0.000000	0.000000	0.000000	0.000000	4,9	61,9	558	**3,43**	23	55	61,9	10,0
>tr|A0A2Z5YD66|A0A2Z5YD66_MYCMR LexA repressor	0	NO_SP	1.000048	0.000007	0.000000	0.000000	0.000000	0.000000	4,5	25,7	244	**3,42**	7	58,6	25,7	5,1
>tr|A0A2Z5Y933|A0A2Z5Y933_MYCMR GH16 domain-containing protein	0	NO_SP	1.000047	0.000000	0.000000	0.000000	0.000000	0.000000	4,3	24,7	218	**3,41**	5	26,6	24,7	5,2
>tr|A0A100I690|A0A100I690_9MYCO GMP synthase [glutamine-hydrolyzing]	0	NO_SP	1.000054	0.000000	0.000000	0.000000	0.000000	0.000000	4,9	56,2	525	**3,39**	17	50,1	56,2	7,8
>tr|B2HQS0|B2HQS0_MYCMM Aldehyde dehydrogenase, PutA_1	0	NO_SP	1.000021	0.000024	0.000001	0.000000	0.000000	0.000000	4,7	54,7	507	**3,35**	14	46	54,7	4,6
>tr|B2HNG4|B2HNG4_MYCMM ATP-dependent Clp protease proteolytic subunit	0	NO_SP	1.000052	0.000000	0.000000	0.000000	0.000000	0.000000	4,8	23,3	211	**3,33**	9	52,6	23,3	4,9
>sp|B2HED1|SUCC_MYCMM Succinate--CoA ligase [ADP-forming] subunit beta	0	NO_SP	1.000076	0.000000	0.000000	0.000000	0.000000	0.000000	4,5	40,8	387	**3,33**	20	80,9	40,8	21,7
>tr|A0A2Z5YAJ7|A0A2Z5YAJ7_MYCMR DNA-directed RNA polymerase subunit alpha	0	NO_SP	0.999043	0.000999	0.000008	0.000002	0.000001	0.000003	4,4	37,7	347	**3,31**	28	80,4	37,7	19,1
>tr|A0A2Z5YCH3|A0A2Z5YCH3_MYCMR Phthiocerol dimycocerosate exporter MmpL7	12	NO_SP	1.000056	0.000004	0.000000	0.000000	0.000000	0.000000	6,9	93,6	919	**3,31**	15	28,5	93,5	5,1
>tr|A0A2Z5YF08|A0A2Z5YF08_MYCMR ESX-5 secretion system protein EccA5	0	NO_SP	0.999743	0.000292	0.000003	0.000000	0.000000	0.000000	5,0	66,2	596	**3,29**	20	41,6	66,2	4,3
>tr|A0A2Z5Y7K7|A0A2Z5Y7K7_MYCMR Leucine--tRNA ligase	0	NO_SP	0.998992	0.000904	0.000083	0.000009	0.000003	0.000020	4,9	109,9	1003	**3,29**	19	32,6	109,9	4,7
>tr|A0A100I248|A0A100I248_9MYCO S-adenosyl-L-methionine-dependent methyltransferase	0	NO_SP	1.000067	0.000000	0.000000	0.000000	0.000000	0.000000	7,9	31,9	277	**3,28**	10	48	31,9	6,4
>tr|A0A2Z5YNZ6|A0A2Z5YNZ6_MYCMR ESX-1 secretion system protein EccCb1	0	NO_SP	1.000048	0.000000	0.000000	0.000000	0.000000	0.000000	6,5	63,6	582	**3,26**	14	44,5	63,6	5,7
>sp|B2HP50|METK_MYCMM S-adenosylmethionine synthase	0	NO_SP	1.000049	0.000001	0.000000	0.000000	0.000000	0.000000	4,8	43,1	403	**3,26**	28	89,8	43,1	12,2
>tr|A0A2Z5YG32|A0A2Z5YG32_MYCMR Uncharacterized protein	0	NO_SP	1.000046	0.000001	0.000000	0.000000	0.000000	0.000000	6,6	94,0	886	**3,24**	17	32,6	94,0	3,0
>tr|A0A2Z5YA32|A0A2Z5YA32_MYCMR Light-repressed protein A	0	NO_SP	1.000015	0.000043	0.000003	0.000000	0.000000	0.000000	7,6	29,6	267	**3,20**	12	57,7	29,6	7,0
>tr|A0A2Z5YM93|A0A2Z5YM93_MYCMR Phosphoribosylformylglycinamidine synthase subunit PurQ	0	NO_SP	1.000062	0.000001	0.000000	0.000000	0.000000	0.000000	4,6	23,6	224	**3,20**	11	68,8	23,6	7,4
>tr|A0A2Z5YGP6|A0A2Z5YGP6_MYCMR Putative	1	NO_SP	1.000068	0.000000	0.000000	0.000000	0.000000	0.000000	9,3	63,9	594	**3,19**	18	56,2	63,9	3,4
>tr|A0A2Z5YH42|A0A2Z5YH42_MYCMR Proteasome subunit alpha	0	NO_SP	0.998220	0.001737	0.000045	0.000002	0.000002	0.000004	4,7	29,4	267	**3,14**	8	44,9	29,4	8,3
>tr|A0A117DY00|A0A117DY00_9MYCO Membrane protein	1	NO_SP	0.999738	0.000273	0.000000	0.000000	0.000000	0.000000	9,4	22,8	213	**3,14**	4	18,3	22,8	3,5
>tr|B2HRJ3|B2HRJ3_MYCMM Uroporphyrin-III C-methyltransferase HemD	0	NO_SP	0.999993	0.000043	0.000001	0.000000	0.000000	0.000000	5,0	58,2	558	**3,13**	16	52,9	58,2	6,4
>tr|A0A2Z5Y8H0|A0A2Z5Y8H0_MYCMR Mammalian cell entry protein	1	NO_SP	0.999951	0.000068	0.000001	0.000001	0.000000	0.000004	5,1	54,4	517	**3,12**	17	55,5	54,4	3,3
>tr|A0A2Z5YIP1|A0A2Z5YIP1_MYCMR 2-oxoglutarate	0	NO_SP	1.000066	0.000000	0.000000	0.000000	0.000000	0.000000	5,4	39,0	363	**3,11**	18	79,3	39,0	9,7
>tr|B2HNL4|B2HNL4_MYCMM Short-chain type dehydrogenase/reductase	0	NO_SP	1.000055	0.000002	0.000000	0.000000	0.000000	0.000000	8,1	27,9	268	**3,11**	18	80,2	27,9	5,1
>tr|A0A2Z5YFU6|A0A2Z5YFU6_MYCMR Catalase-peroxidase	0	NO_SP	0.999925	0.000115	0.000010	0.000000	0.000000	0.000001	4,8	77,5	708	**3,09**	27	47,9	77,5	6,5
>tr|A0A2Z5Y9Q8|A0A2Z5Y9Q8_MYCMR F420-dependent glucose-6-phosphate dehydrogenase	0	NO_SP	1.000076	0.000001	0.000000	0.000000	0.000000	0.000000	5,2	37,3	336	**3,09**	15	68,2	37,3	3,6
>tr|A0A2Z5YK83|A0A2Z5YK83_MYCMR ATPase AAA	0	NO_SP	1.000059	0.000000	0.000000	0.000000	0.000000	0.000000	5,2	73,3	694	**3,08**	2	4	73,3	8,6
>tr|A0A3E2N3B0|A0A3E2N3B0_MYCMR Uncharacterized protein	0	NO_SP	1.000068	0.000001	0.000000	0.000000	0.000000	0.000000	4,7	15,9	155	**3,07**	6	75,5	15,9	7,7
>tr|A0A100HZD7|A0A100HZD7_9MYCO Indole-3-glycerol phosphate synthase	0	NO_SP	1.000036	0.000000	0.000000	0.000000	0.000000	0.000000	5,2	27,7	267	**3,06**	17	68,9	27,7	13,0
>tr|A0A2Z5YBU2|A0A2Z5YBU2_MYCMR Protein translocase subunit SecA	0	NO_SP	1.000026	0.000000	0.000000	0.000000	0.000000	0.000000	4,8	104,9	938	**3,06**	42	54,5	104,9	11,4
>tr|A0A117DTS6|A0A117DTS6_9MYCO Glycosyl hydrolase family 15	0	NO_SP	1.000054	0.000001	0.000000	0.000000	0.000000	0.000000	6,1	67,0	588	**3,04**	21	54,4	67,0	3,1
>tr|A0A2Z5YK79|A0A2Z5YK79_MYCMR GTP-binding protein	0	NO_SP	1.000070	0.000000	0.000000	0.000000	0.000000	0.000000	4,8	63,3	588	**3,04**	1	2	63,3	9,6
>tr|A0A2Z5Y8S3|A0A2Z5Y8S3_MYCMR 3-oxoacyl-ACP reductase	0	NO_SP	1.000046	0.000000	0.000000	0.000000	0.000000	0.000000	6,1	46,8	454	**3,03**	26	72,5	46,8	5,5
>tr|B2HGU5|B2HGU5_MYCMM Phospho-2-dehydro-3-deoxyheptonate aldolase	0	NO_SP	1.000055	0.000000	0.000000	0.000000	0.000000	0.000000	5,4	50,6	462	**3,03**	15	57,8	50,6	9,9
>tr|A0A2Z5YHF1|A0A2Z5YHF1_MYCMR Flavoprotein	0	NO_SP	1.000066	0.000000	0.000000	0.000000	0.000000	0.000000	5,5	55,4	526	**3,03**	17	51,7	55,4	3,7
>tr|A0A2Z5YG04|A0A2Z5YG04_MYCMR Glycine dehydrogenase (decarboxylating)	0	NO_SP	1.000049	0.000002	0.000000	0.000000	0.000000	0.000000	5,1	100,5	945	**3,02**	26	40,7	100,5	7,7
>tr|B2HMA7|B2HMA7_MYCMM Adenosine 5'-phosphosulfate reductase	0	NO_SP	0.999954	0.000104	0.000000	0.000000	0.000000	0.000000	4,5	26,7	249	**3,02**	8	47,4	26,7	4,9
>tr|B2HHN2|B2HHN2_MYCMM RNase H type-1 domain-containing protein	0	NO_SP	1.000028	0.000030	0.000001	0.000000	0.000000	0.000000	8,1	40,6	374	**3,01**	11	44,4	40,6	3,8
>tr|B2HEF7|B2HEF7_MYCMM Non-specific serine/threonine protein kinase	1	NO_SP	1.000049	0.000000	0.000000	0.000000	0.000000	0.000000	6,4	73,4	698	**2,99**	15	38	73,4	14,9
>tr|A0A2Z5YGJ3|A0A2Z5YGJ3_MYCMR Pup--protein ligase	0	NO_SP	1.000043	0.000000	0.000000	0.000000	0.000000	0.000000	6,5	50,6	447	**2,98**	19	55,5	50,6	15,5
>tr|A0A3E2MQB8|A0A3E2MQB8_MYCMR Phosphoglycerate kinase	0	NO_SP	1.000039	0.000000	0.000000	0.000000	0.000000	0.000000	4,7	43,0	416	**2,98**	18	66,1	43,0	6,8
>tr|A0A100I851|A0A100I851_9MYCO Succinate--CoA ligase [ADP-forming] subunit alpha	0	NO_SP	1.000063	0.000000	0.000000	0.000000	0.000000	0.000000	6,1	30,8	300	**2,94**	20	87,3	30,8	15,9
>tr|A0A2Z5YDQ3|A0A2Z5YDQ3_MYCMR Amino acid decarboxylase	0	NO_SP	0.957749	0.042070	0.000076	0.000049	0.000032	0.000044	5,7	105,6	943	**2,93**	21	37,4	105,6	5,7
**>sp|B2HQ93|BIOB_MYCMM Biotin synthase**	0	NO_SP	1.000049	0.000003	0.000000	0.000000	0.000000	0.000000	4,4	37,6	349	**2,92**	12	58,7	37,6	2,8
>tr|A0A2Z5YPV4|A0A2Z5YPV4_MYCMR ESX-1 secretion-associated protein EspG1	0	NO_SP	1.000053	0.000002	0.000000	0.000000	0.000000	0.000000	5,9	29,7	279	**2,90**	11	64,5	29,7	6,2
>tr|A0A2Z5YA35|A0A2Z5YA35_MYCMR 50S ribosomal protein L7/L12	0	NO_SP	1.000060	0.000000	0.000000	0.000000	0.000000	0.000000	4,3	13,4	130	**2,90**	11	48,5	13,4	3,1
>tr|A0A2Z5YCG4|A0A2Z5YCG4_MYCMR Translation initiation factor IF-2	0	NO_SP	1.000022	0.000000	0.000000	0.000000	0.000000	0.000000	6,8	98,3	949	**2,89**	27	42,5	98,3	3,5
>tr|B2HQT2|B2HQT2_MYCMM Isocitrase	0	NO_SP	1.000082	0.000000	0.000000	0.000000	0.000000	0.000000	5,6	47,3	428	**2,89**	19	66,4	47,3	13,7
>tr|A0A2Z5YDE4|A0A2Z5YDE4_MYCMR Threonine--tRNA ligase	0	NO_SP	0.978443	0.021403	0.000065	0.000041	0.000020	0.000039	5,2	77,2	691	**2,86**	16	30,7	77,2	6,5
>tr|A0A2Z5YEG5|A0A2Z5YEG5_MYCMR CTP synthase	0	NO_SP	1.000090	0.000000	0.000000	0.000000	0.000000	0.000000	5,7	63,7	583	**2,85**	20	52	63,7	5,5
>tr|A0A2Z5YB08|A0A2Z5YB08_MYCMR Sulfurtransferase	0	NO_SP	1.000072	0.000001	0.000000	0.000000	0.000000	0.000000	5,0	33,5	301	**2,82**	12	59,1	33,5	6,9
>tr|B2HNT0|B2HNT0_MYCMM Hydrolase	0	NO_SP	1.000058	0.000001	0.000000	0.000000	0.000000	0.000000	6,3	44,1	406	**2,80**	11	40,9	44,1	4,1
>tr|B2HS42|B2HS42_MYCMM Iron-sulfur cluster carrier protein	0	NO_SP	1.000058	0.000000	0.000000	0.000000	0.000000	0.000000	5,5	40,7	386	**2,80**	12	48,2	40,7	6,0
>tr|A0A2Z5YCX7|A0A2Z5YCX7_MYCMR Polyribonucleotide nucleotidyltransferase	0	NO_SP	1.000050	0.000003	0.000000	0.000000	0.000000	0.000000	4,5	80,6	762	**2,80**	36	63,4	80,6	16,0
>tr|A0A2Z5YK99|A0A2Z5YK99_MYCMR 3 beta-hydroxysteroid dehydrogenase/Delta 5-->4-isomerase	0	NO_SP	1.000069	0.000000	0.000000	0.000000	0.000000	0.000000	7,0	39,5	360	**2,79**	8	27,8	39,5	8,4
>tr|A0A2Z5Y8U8|A0A2Z5Y8U8_MYCMR Type VII secretion protein EccC	1	NO_SP	0.999859	0.000174	0.000000	0.000000	0.000000	0.000000	6,2	146,1	1333	**2,79**	36	41,9	146,1	10,4
>tr|A0A2Z5YJW4|A0A2Z5YJW4_MYCMR 2,3,4,5-tetrahydropyridine-2,6-dicarboxylate N-succinyltransferase	0	NO_SP	0.970454	0.029152	0.000280	0.000046	0.000029	0.000062	5,3	32,2	314	**2,78**	16	79	32,2	3,1
>tr|B2HGQ3|B2HGQ3_MYCMM Mycobactin synthetase protein B	0	NO_SP	1.000050	0.000000	0.000000	0.000000	0.000000	0.000000	4,8	741,8	6887	**2,77**	10	4,3	741,8	3,7
>tr|B2HDH8|B2HDH8_MYCMM Two-component transcriptional regulator TrcR	0	NO_SP	1.000050	0.000000	0.000000	0.000000	0.000000	0.000000	7,5	28,7	254	**2,77**	7	33,9	28,7	8,0
>tr|A0A3E2MVH0|A0A3E2MVH0_MYCMR Pyruvate kinase	0	NO_SP	1.000040	0.000000	0.000000	0.000000	0.000000	0.000000	4,9	50,8	472	**2,76**	21	68,2	50,8	11,5
>tr|A0A2Z5YFZ0|A0A2Z5YFZ0_MYCMR Protein translocase subunit SecA	0	NO_SP	1.000046	0.000000	0.000000	0.000000	0.000000	0.000000	4,6	84,9	778	**2,76**	25	40,7	84,9	5,2
>tr|A0A2Z5YKB7|A0A2Z5YKB7_MYCMR SBP_bac_5 domain-containing protein	0	LIPO	0.000000	0.000000	1.000091	0.000000	0.000000	0.000000	5,7	59,0	552	**2,74**	12	36,1	59,0	27,3
>tr|A0A2Z5YED3|A0A2Z5YED3_MYCMR Cyanophycin synthase	0	NO_SP	1.000042	0.000000	0.000000	0.000000	0.000000	0.000000	4,7	97,5	911	**2,74**	41	61,8	97,5	5,6
>tr|B2HDU3|B2HDU3_MYCMM Succinate dehydrogenase flavoprotein subunit	0	NO_SP	1.000008	0.000012	0.000001	0.000000	0.000000	0.000000	6,2	64,3	584	**2,69**	22	52,6	64,3	13,1
>tr|A0A2Z5YIS5|A0A2Z5YIS5_MYCMR Cyclopropane mycolic acid synthase MmaA2	0	NO_SP	1.000062	0.000000	0.000000	0.000000	0.000000	0.000000	5,2	33,2	289	**2,68**	4	18,3	33,2	8,8
>tr|B2HLU7|B2HLU7_MYCMM NAD(P) transhydrogenase subunit beta	9	NO_SP	1.000004	0.000000	0.000000	0.000000	0.000000	0.000000	6,3	48,4	474	**2,68**	10	30,4	48,4	6,1
>tr|A0A2Z5YA62|A0A2Z5YA62_MYCMR UPF0336 protein DAVIS_03420	0	NO_SP	1.000054	0.000002	0.000000	0.000000	0.000000	0.000000	4,6	18,7	166	**2,67**	8	72,3	18,7	5,4

>tr|B2HMC7|B2HMC7_MYCMM Alpha-E domain-containing protein	0	NO_SP	1.000069	0.000000	0.000000	0.000000	0.000000	0.000000	6,0	36,3	325	**2,66**	11	50,8	36,3	3,6
>tr|B2HGY4|B2HGY4_MYCMM Dihydrolipoamide acetyltransferase component of pyruvate dehydrogenase	0	NO_SP	1.000054	0.000000	0.000000	0.000000	0.000000	0.000000	4,4	60,7	588	**2,66**	22	55,8	60,7	16,7
>tr|A0A2Z5YEY2|A0A2Z5YEY2_MYCMR Putative RNA binding protein, contains S1 domain	0	NO_SP	1.000063	0.000000	0.000000	0.000000	0.000000	0.000000	6,0	84,6	787	**2,65**	31	52,1	84,6	9,2
>tr|A0A2Z5YNJ5|A0A2Z5YNJ5_MYCMR 1-acyl-sn-glycerol-3-phosphate acyltransferase	0	NO_SP	1.000063	0.000000	0.000000	0.000000	0.000000	0.000000	10,6	28,5	259	**2,64**	5	33,2	28,5	6,7
>tr|B2HEX1|B2HEX1_MYCMM	0	NO_SP	1.000077	0.000000	0.000000	0.000000	0.000000	0.000000	9,5	33,9	323	**2,64**	8	38,7	33,9	4,3
>tr|A0A2Z5YDK2|A0A2Z5YDK2_MYCMR Sugar ABC transporter ATP-binding protein	0	NO_SP	1.000054	0.000000	0.000000	0.000000	0.000000	0.000000	7,3	39,0	360	**2,61**	15	70,6	39,0	9,7
>tr|A0A124BWX6|A0A124BWX6_9MYCO Acyl-CoA dehydrogenase	0	NO_SP	0.999847	0.000174	0.000006	0.000001	0.000000	0.000002	5,5	64,4	573	**2,61**	26	58,6	64,4	5,4
>tr|B2HEK7|B2HEK7_MYCMM Propionyl-CoA carboxylase beta chain 5 AccD5	0	NO_SP	1.000058	0.000001	0.000000	0.000000	0.000000	0.000000	4,8	58,9	546	**2,61**	21	58,4	58,9	3,2
>tr|B2HSI8|B2HSI8_MYCMM 50S ribosomal protein L10	0	NO_SP	1.000058	0.000000	0.000000	0.000000	0.000000	0.000000	5,0	21,1	206	**2,56**	12	71,8	21,1	6,5
>tr|A0A2Z5YMM5|A0A2Z5YMM5_MYCMR 3-hydroxyacyl-CoA dehydrogenase	0	NO_SP	1.000057	0.000000	0.000000	0.000000	0.000000	0.000000	5,0	75,7	714	**2,54**	41	62,7	75,7	11,0
>tr|A0A2Z5Y8M9|A0A2Z5Y8M9_MYCMR Phosphoenolpyruvate carboxykinase [GTP]	0	NO_SP	1.000025	0.000000	0.000000	0.000000	0.000000	0.000000	4,5	67,8	609	**2,52**	30	74,9	67,8	6,1
>tr|A0A2Z5YC88|A0A2Z5YC88_MYCMR Ketol-acid reductoisomerase (NADP(+))	0	NO_SP	1.000063	0.000000	0.000000	0.000000	0.000000	0.000000	4,7	37,0	343	**2,52**	16	57,1	37,0	6,9
>tr|B2HF84|B2HF84_MYCMM Citrate synthase (unknown stereospecificity)	0	NO_SP	1.000068	0.000001	0.000000	0.000000	0.000000	0.000000	5,3	40,1	373	**2,51**	11	49,6	40,1	3,9
>tr|A0A2Z5YF64|A0A2Z5YF64_MYCMR Uncharacterized protein	0	NO_SP	0.999913	0.000130	0.000001	0.000000	0.000000	0.000000	8,5	22,7	209	**2,50**	7	50,2	22,7	8,8
>tr|A0A2Z5YEV2|A0A2Z5YEV2_MYCMR Quinolinate synthase	0	NO_SP	1.000061	0.000000	0.000000	0.000000	0.000000	0.000000	5,0	37,6	352	**2,48**	11	72,2	37,6	4,2
>tr|B2HKB1|B2HKB1_MYCMM Conserved membrane protein	0	NO_SP	0.995953	0.004004	0.000022	0.000009	0.000006	0.000030	11,8	47,5	440	**2,48**	8	24,3	47,5	6,9
>tr|A0A2Z5Y7L4|A0A2Z5Y7L4_MYCMR Peptidoglycan glycosyltransferase	1	NO_SP	1.000048	0.000000	0.000000	0.000000	0.000000	0.000000	6,2	86,7	823	**2,47**	1	1,6	86,7	15,4
>tr|A0A3E2N1W6|A0A3E2N1W6_MYCMR Carbonic anhydrase	0	NO_SP	1.000074	0.000000	0.000000	0.000000	0.000000	0.000000	5,8	21,7	208	**2,45**	6	48,6	21,7	4,7
>tr|A0A2Z5YDX3|A0A2Z5YDX3_MYCMR Universal stress protein	0	NO_SP	1.000051	0.000000	0.000000	0.000000	0.000000	0.000000	5,0	27,9	264	**2,45**	12	59,5	27,9	9,4
>tr|B2HI51|B2HI51_MYCMM DNA gyrase subunit A	0	NO_SP	1.000072	0.000000	0.000000	0.000000	0.000000	0.000000	5,3	92,8	839	**2,45**	35	61,4	92,8	8,5
>tr|A0A2Z5YD19|A0A2Z5YD19_MYCMR Uncharacterized protein	0	NO_SP	1.000063	0.000013	0.000000	0.000000	0.000000	0.000000	4,3	26,3	247	**2,43**	9	52,6	26,3	8,9
>tr|A0A2Z5YIH3|A0A2Z5YIH3_MYCMR Glycine--tRNA ligase	0	NO_SP	1.000069	0.000002	0.000000	0.000000	0.000000	0.000000	5,2	52,3	459	**2,43**	22	60,6	52,3	20,5
>tr|A0A2Z5YDT4|A0A2Z5YDT4_MYCMR ATPase	0	NO_SP	1.000037	0.000000	0.000000	0.000000	0.000000	0.000000	6,0	40,0	371	**2,41**	17	74,4	40,0	8,4
>tr|A0A2Z5YKU3|A0A2Z5YKU3_MYCMR Glucose-6-phosphate isomerase	0	NO_SP	1.000052	0.000000	0.000000	0.000000	0.000000	0.000000	5,1	60,3	554	**2,41**	17	49,6	60,3	4,6
>tr|B2HKE9|B2HKE9_MYCMM Conserved transcriptional regulator	0	NO_SP	1.000055	0.000000	0.000000	0.000000	0.000000	0.000000	5,4	46,7	428	**2,40**	14	48,4	46,7	6,4
>tr|B2HH30|B2HH30_MYCMM Amidophosphoribosyltransferase	0	NO_SP	1.000070	0.000000	0.000000	0.000000	0.000000	0.000000	5,1	54,3	508	**2,40**	13	45,5	54,3	5,0
>tr|A0A124BW47|A0A124BW47_9MYCO Non-ribosomal peptide synthetase	5	NO_SP	1.000046	0.000000	0.000000	0.000000	0.000000	0.000000	6,8	138,2	1306	**2,39**	19	22,4	138,2	3,3
>tr|B2HQ37|B2HQ37_MYCMM HpcH_HpaI domain-containing protein	0	NO_SP	1.000076	0.000000	0.000000	0.000000	0.000000	0.000000	5,2	29,5	266	**2,38**	9	33,5	29,5	5,3
>tr|A0A2Z5YHP4|A0A2Z5YHP4_MYCMR Putative	0	NO_SP	1.000043	0.000000	0.000000	0.000000	0.000000	0.000000	10,2	34,9	323	**2,36**	9	33,4	34,9	3,4
>tr|A0A2Z5YHM6|A0A2Z5YHM6_MYCMR Phosphoenolpyruvate synthase	0	NO_SP	1.000072	0.000000	0.000000	0.000000	0.000000	0.000000	4,7	88,6	806	**2,35**	29	57,2	88,6	6,0
>tr|A0A2Z5YDI5|A0A2Z5YDI5_MYCMR Acyl-CoA dehydrogenase	0	NO_SP	1.000053	0.000004	0.000000	0.000000	0.000000	0.000000	4,7	65,8	609	**2,35**	21	50,6	65,8	5,8
>tr|B2HGR8|B2HGR8_MYCMM UDP-N-acetylmuramoylalanine--D-glutamate ligase	0	NO_SP	1.000039	0.000019	0.000000	0.000000	0.000000	0.000000	4,8	50,1	488	**2,34**	9	32,4	50,1	4,9
>tr|A0A3E2MU78|A0A3E2MU78_MYCMR Sporulation initiation inhibitor protein Soj	0	NO_SP	1.000041	0.000001	0.000000	0.000000	0.000000	0.000000	5,4	31,2	287	**2,34**	13	55,7	31,2	7,1
>tr|A0A2Z5YPF9|A0A2Z5YPF9_MYCMR Putative ATPase	0	NO_SP	1.000055	0.000012	0.000000	0.000000	0.000000	0.000000	4,4	33,7	318	**2,33**	13	52,5	33,7	3,8
>tr|A0A2Z5YJU6|A0A2Z5YJU6_MYCMR Homoserine dehydrogenase	0	NO_SP	1.000054	0.000000	0.000000	0.000000	0.000000	0.000000	4,5	45,8	441	**2,33**	15	54,9	45,8	7,6
>tr|A0A100I232|A0A100I232_9MYCO Glycosyl transferase	0	NO_SP	0.999277	0.000526	0.000137	0.000011	0.000003	0.000060	6,6	70,3	626	**2,32**	20	48,9	70,3	5,4
>tr|A0A2Z5YE48|A0A2Z5YE48_MYCMR 3-dehydroquinate synthase	0	NO_SP	1.000074	0.000000	0.000000	0.000000	0.000000	0.000000	4,9	39,8	362	**2,32**	12	61	39,8	4,4
>tr|A0A2Z5Y9C0|A0A2Z5Y9C0_MYCMR Phosphate acetyltransferase	0	NO_SP	1.000047	0.000000	0.000000	0.000000	0.000000	0.000000	4,9	73,8	699	**2,32**	21	49,1	73,8	3,5
>tr|A0A3E2MQH1|A0A3E2MQH1_MYCMR Carbamoyl-phosphate synthase large chain	0	NO_SP	0.968586	0.030539	0.000699	0.000074	0.000048	0.000085	4,6	119,7	1121	**2,32**	33	43,2	119,7	5,0
>tr|B2HQX5|B2HQX5_MYCMM Anthranilate synthase component 1	0	NO_SP	0.999985	0.000044	0.000002	0.000000	0.000000	0.000000	4,6	55,4	512	**2,31**	13	37,3	55,4	4,2
>tr|B2HKB0|B2HKB0_MYCMM Methanol dehydrogenase transcriptional regulatory protein MoxR2	0	NO_SP	1.000035	0.000016	0.000000	0.000000	0.000000	0.000000	5,7	34,6	325	**2,29**	11	53,8	34,6	5,6
>tr|B2HD60|B2HD60_MYCMM Alkyl hydroperoxide reductase C	0	NO_SP	1.000027	0.000005	0.000000	0.000000	0.000000	0.000000	4,3	21,5	195	**2,28**	15	84,6	21,5	7,0
>tr|A0A117DVM5|A0A117DVM5_9MYCO ATP-dependent Clp protease proteolytic subunit	0	NO_SP	1.000060	0.000000	0.000000	0.000000	0.000000	0.000000	4,6	21,2	195	**2,28**	7	53,8	21,2	3,4
>tr|A0A2Z5YDX9|A0A2Z5YDX9_MYCMR Methionyl-tRNA formyltransferase	0	NO_SP	1.000044	0.000000	0.000000	0.000000	0.000000	0.000000	7,9	32,6	312	**2,28**	7	42	32,6	4,4
>tr|B2HQ26|B2HQ26_MYCMM Pyridoxal phosphate-dependent enzyme, WecE	0	NO_SP	0.999676	0.000316	0.000014	0.000003	0.000001	0.000006	6,2	42,5	382	**2,27**	9	40,1	42,5	3,2
>tr|A0A2Z5YF88|A0A2Z5YF88_MYCMR Copper transporter MctB	1	NO_SP	0.871124	0.019097	0.008837	0.000210	0.000108	0.100640	4,8	31,9	317	**2,27**	8	55,8	31,9	4,2
>tr|B2HQT3|B2HQT3_MYCMM 3-hydroxybutyryl-CoA dehydrogenase FadB2	0	NO_SP	1.000068	0.000000	0.000000	0.000000	0.000000	0.000000	5,2	30,9	288	**2,27**	15	65,3	30,9	4,6
>tr|B2HLS8|B2HLS8_MYCMM Short-chain type dehydrogenase/reductase	0	NO_SP	1.000044	0.000002	0.000000	0.000000	0.000000	0.000000	5,1	29,8	286	**2,25**	13	83,6	29,8	6,1
>tr|A0A2Z5YNR7|A0A2Z5YNR7_MYCMR ESX-1 secretion-associated protein EspB	0	NO_SP	1.000009	0.000036	0.000000	0.000000	0.000000	0.000000	4,3	46,9	454	**2,25**	16	42,3	46,9	11,3
>tr|A0A2Z5YNJ9|A0A2Z5YNJ9_MYCMR Acyl-CoA dehydrogenase	0	NO_SP	1.000058	0.000000	0.000000	0.000000	0.000000	0.000000	4,6	38,3	350	**2,24**	11	49,4	38,3	6,7
>tr|A0A2Z5YN08|A0A2Z5YN08_MYCMR Uncharacterized protein	2	NO_SP	0.998249	0.001704	0.000044	0.000005	0.000003	0.000010	10,1	18,9	172	**2,23**	3	24,4	18,9	4,0
>tr|A0A2Z5YDP5|A0A2Z5YDP5_MYCMR Polyketide synthase	0	NO_SP	0.878956	0.003429	0.117425	0.000018	0.000011	0.000168	4,6	222,6	2090	**2,23**	43	33,5	222,6	6,6
>tr|A0A2Z5YBI2|A0A2Z5YBI2_MYCMR Chromosome partitioning protein ParA	0	NO_SP	1.000038	0.000001	0.000000	0.000000	0.000000	0.000000	6,3	28,3	266	**2,21**	8	34,2	28,3	3,5
>tr|A0A2Z5YE23|A0A2Z5YE23_MYCMR Uncharacterized protein	0	NO_SP	1.000085	0.000000	0.000000	0.000000	0.000000	0.000000	7,0	55,4	497	**2,21**	15	35,4	55,4	4,8
>tr|A0A3E2MQK9|A0A3E2MQK9_MYCMR Aspartate--tRNA(Asp/Asn) ligase	0	NO_SP	0.999382	0.000595	0.000047	0.000001	0.000001	0.000004	4,9	64,2	591	**2,20**	18	44,2	64,2	7,6
>tr|A0A2Z5YPN8|A0A2Z5YPN8_MYCMR Long-chain-fatty-acid--AMP ligase FadD32	0	NO_SP	1.000089	0.000000	0.000000	0.000000	0.000000	0.000000	6,2	68,9	629	**2,19**	24	61,7	68,9	5,7
>tr|A0A2Z5YBD4|A0A2Z5YBD4_MYCMR Ribosome hibernation promoting factor	0	NO_SP	1.000040	0.000000	0.000000	0.000000	0.000000	0.000000	6,6	26,2	229	**2,19**	7	37,6	26,2	7,6
>tr|A0A2Z5YAZ9|A0A2Z5YAZ9_MYCMR Acid phosphatase	0	NO_SP	0.999070	0.000970	0.000009	0.000002	0.000001	0.000002	6,2	29,1	275	**2,19**	10	67,6	29,1	3,0
>tr|A0A2Z5YJX5|A0A2Z5YJX5_MYCMR ATP synthase subunit alpha	0	NO_SP	0.999903	0.000124	0.000006	0.000000	0.000000	0.000001	4,9	59,4	549	**2,18**	39	73	59,4	19,9
>sp|B2HJN3|EFTS_MYCMM Elongation factor Ts	0	NO_SP	1.000027	0.000003	0.000000	0.000000	0.000000	0.000000	4,8	28,5	271	**2,17**	19	79	28,5	5,4
>tr|A0A2Z5YMV3|A0A2Z5YMV3_MYCMR ATP-dependent zinc metalloprotease FtsH	2	NO_SP	0.960543	0.018415	0.020608	0.000036	0.000028	0.000377	5,4	79,9	741	**2,16**	32	59,9	79,9	5,3
>tr|B2HQZ0|B2HQZ0_MYCMM Integral membrane cytochrome D ubiquinol	9	NO_SP	1.000045	0.000000	0.000000	0.000000	0.000000	0.000000	8,4	54,2	485	**2,14**	8	21,2	54,2	3,9
>tr|A0A2Z5YCW3|A0A2Z5YCW3_MYCMR Phthiocerol/phenolphthiocerol synthesis polyketide synthase I PpsD	0	NO_SP	1.000005	0.000022	0.000000	0.000000	0.000000	0.000000	5,1	191,7	1806	**2,12**	52	48,3	191,7	5,2
>tr|A0A2Z5YJR6|A0A2Z5YJR6_MYCMR Malate dehydrogenase	0	NO_SP	1.000045	0.000000	0.000000	0.000000	0.000000	0.000000	4,6	34,6	329	**2,11**	20	73,3	34,6	10,5
>tr|A0A100IFY9|A0A100IFY9_9MYCO Alanine and proline rich protein	0	NO_SP	1.000072	0.000002	0.000000	0.000000	0.000000	0.000000	5,8	75,8	738	**2,10**	13	30,8	75,8	4,0
>tr|A0A2Z5YC21|A0A2Z5YC21_MYCMR Long-chain-fatty-acid--CoA ligase	0	NO_SP	1.000060	0.000001	0.000000	0.000000	0.000000	0.000000	4,7	53,5	495	**2,09**	10	24,2	53,5	7,0
>tr|A0A2Z5YI00|A0A2Z5YI00_MYCMR Branched-chain-amino-acid aminotransferase	0	NO_SP	1.000040	0.000017	0.000000	0.000000	0.000000	0.000000	4,9	39,8	368	**2,09**	13	50,5	39,8	7,7
>tr|A0A2Z5YEK7|A0A2Z5YEK7_MYCMR Putative transcriptional regulatory protein pdtaR	0	NO_SP	1.000065	0.000000	0.000000	0.000000	0.000000	0.000000	4,5	23,0	208	**2,08**	2	6,2	23,0	3,2
>tr|A0A3E2MZY6|A0A3E2MZY6_MYCMR Carnitinyl-CoA dehydratase	0	NO_SP	1.000049	0.000003	0.000000	0.000000	0.000000	0.000000	5,2	27,1	260	**2,07**	10	47,7	27,1	3,1
>tr|B2HEL3|B2HEL3_MYCMM Acyl-CoA dehydrogenase FadE25	0	NO_SP	1.000054	0.000000	0.000000	0.000000	0.000000	0.000000	4,9	41,5	389	**2,07**	15	55,3	41,5	7,4
>tr|B2HQU4|B2HQU4_MYCMM Conserved membrane protein	1	NO_SP	1.000068	0.000000	0.000000	0.000000	0.000000	0.000000	7,4	36,6	348	**2,05**	17	70,4	36,6	4,9
>tr|A0A2Z5YHY9|A0A2Z5YHY9_MYCMR Adenosine kinase	0	NO_SP	0.998419	0.001557	0.000013	0.000004	0.000002	0.000002	4,4	34,4	324	**2,03**	10	61,4	34,4	3,9
>tr|B2HIH0|B2HIH0_MYCMM D-3-phosphoglycerate dehydrogenase	0	NO_SP	1.000055	0.000008	0.000000	0.000000	0.000000	0.000000	4,6	54,2	528	**2,03**	26	83,1	54,2	9,6
>tr|A0A2Z5YEU3|A0A2Z5YEU3_MYCMR Type VII secretion protein EccC	0	NO_SP	1.000041	0.000000	0.000000	0.000000	0.000000	0.000000	5,1	144,2	1308	**2,02**	35	38,5	144,2	3,1

<table>
<tr><td>>tr|A0A2Z5YCQ2|A0A2Z5YCQ2_MYCMR Putative lipoprotein LppX</td><td>1</td><td>LIPO</td><td>0.000000</td><td>0.000001</td><td>1.000076</td><td>0.000000</td><td>0.000000</td><td>0.000000</td><td>5,0</td><td>24,0</td><td>229</td><td>2,02</td><td>13</td><td>65,9</td><td>24,0</td><td>4,2</td></tr>
<tr><td>>tr|B2HMJ8|B2HMJ8_MYCMM Propionyl-CoA carboxylase beta chain 4 AccD4_1</td><td>0</td><td>NO_SP</td><td>1.000055</td><td>0.000000</td><td>0.000000</td><td>0.000000</td><td>0.000000</td><td>0.000000</td><td>5,2</td><td>56,9</td><td>526</td><td>2,01</td><td>24</td><td>66</td><td>56,9</td><td>4,5</td></tr>
<tr><td>>tr|A0A2Z5YNV9|A0A2Z5YNV9_MYCMR ESX-1 secretion system protein EccA1</td><td>0</td><td>NO_SP</td><td>1.000040</td><td>0.000003</td><td>0.000000</td><td>0.000000</td><td>0.000000</td><td>0.000000</td><td>4,7</td><td>62,2</td><td>573</td><td>2,01</td><td>5</td><td>9,4</td><td>62,2</td><td>5,4</td></tr>
<tr><td>>sp|B2HD09|CH10_MYCMM Co-chaperonin GroES</td><td>0</td><td>NO_SP</td><td>1.000046</td><td>0.000001</td><td>0.000000</td><td>0.000000</td><td>0.000000</td><td>0.000000</td><td>4,3</td><td>10,7</td><td>100</td><td>2,01</td><td>8</td><td>84</td><td>10,7</td><td>4,2</td></tr>
<tr><td>>tr|A0A2Z5YIR9|A0A2Z5YIR9_MYCMR AAA domain-containing protein</td><td>0</td><td>NO_SP</td><td>1.000059</td><td>0.000009</td><td>0.000000</td><td>0.000000</td><td>0.000000</td><td>0.000000</td><td>4,8</td><td>31,9</td><td>291</td><td>1,99</td><td>11</td><td>57,7</td><td>31,9</td><td>4,2</td></tr>
<tr><td>>tr|A0A2Z5YEA0|A0A2Z5YEA0_MYCMR Long-chain-fatty-acid--AMP ligase FadD28</td><td>0</td><td>NO_SP</td><td>1.000061</td><td>0.000001</td><td>0.000000</td><td>0.000000</td><td>0.000000</td><td>0.000000</td><td>5,1</td><td>63,1</td><td>585</td><td>1,99</td><td>29</td><td>74,7</td><td>63,1</td><td>8,3</td></tr>
<tr><td>>tr|A0A3E2MS03|A0A3E2MS03_MYCMR Putative Rieske 2Fe-2S iron-sulfur protein</td><td>0</td><td>NO_SP</td><td>1.000037</td><td>0.000008</td><td>0.000000</td><td>0.000000</td><td>0.000000</td><td>0.000000</td><td>4,7</td><td>58,1</td><td>516</td><td>1,99</td><td>12</td><td>27,9</td><td>58,1</td><td>4,0</td></tr>
<tr><td>>tr|A0A3E2MWT1|A0A3E2MWT1_MYCMR Probable dual-specificity RNA methyltransferase RlmN</td><td>0</td><td>NO_SP</td><td>1.000059</td><td>0.000000</td><td>0.000000</td><td>0.000000</td><td>0.000000</td><td>0.000000</td><td>8,4</td><td>39,7</td><td>364</td><td>1,99</td><td>1</td><td>4,7</td><td>39,7</td><td>3,5</td></tr>
<tr><td>>tr|B2HLZ7|B2HLZ7_MYCMM RNA polymerase sigma factor SigA</td><td>0</td><td>NO_SP</td><td>1.000062</td><td>0.000000</td><td>0.000000</td><td>0.000000</td><td>0.000000</td><td>0.000000</td><td>4,3</td><td>54,7</td><td>501</td><td>1,97</td><td>20</td><td>37,5</td><td>54,7</td><td>13,0</td></tr>
<tr><td>>tr|B2HJ65|B2HJ65_MYCMM Hypoxanthine phosphoribosyltransferase</td><td>0</td><td>NO_SP</td><td>0.999627</td><td>0.000394</td><td>0.000002</td><td>0.000000</td><td>0.000000</td><td>0.000000</td><td>4,5</td><td>22,3</td><td>202</td><td>1,97</td><td>7</td><td>49,5</td><td>22,3</td><td>3,4</td></tr>
<tr><td>>tr|A0A2Z5YFV7|A0A2Z5YFV7_MYCMR Peptidase_M28 domain-containing protein</td><td>0</td><td>NO_SP</td><td>1.000036</td><td>0.000002</td><td>0.000000</td><td>0.000000</td><td>0.000000</td><td>0.000000</td><td>6,4</td><td>43,4</td><td>401</td><td>1,97</td><td>10</td><td>43,6</td><td>43,4</td><td>4,6</td></tr>
<tr><td>>tr|B2HF47|B2HF47_MYCMM Polyprenol-monophosphomannose synthase, Ppm1B</td><td>0</td><td>NO_SP</td><td>1.000069</td><td>0.000000</td><td>0.000000</td><td>0.000000</td><td>0.000000</td><td>0.000000</td><td>7,3</td><td>29,4</td><td>268</td><td>1,96</td><td>11</td><td>57,1</td><td>29,4</td><td>2,8</td></tr>
<tr><td>>tr|A0A100I2P8|A0A100I2P8_9MYCO Enoyl-CoA hydratase EchA6</td><td>0</td><td>NO_SP</td><td>1.000061</td><td>0.000005</td><td>0.000000</td><td>0.000000</td><td>0.000000</td><td>0.000000</td><td>5,4</td><td>26,2</td><td>243</td><td>1,95</td><td>13</td><td>68,7</td><td>26,2</td><td>8,3</td></tr>
<tr><td>>tr|B5TV81|B5TV81_MYCMR ESAT-6-like protein</td><td>0</td><td>NO_SP</td><td>1.000059</td><td>0.000000</td><td>0.000000</td><td>0.000000</td><td>0.000000</td><td>0.000000</td><td>4,3</td><td>10,7</td><td>100</td><td>1,95</td><td>7</td><td>78</td><td>10,7</td><td>3,9</td></tr>
<tr><td>>tr|A0A100I554|A0A100I554_9MYCO Ribonucleoside-diphosphate reductase</td><td>0</td><td>NO_SP</td><td>1.000074</td><td>0.000000</td><td>0.000000</td><td>0.000000</td><td>0.000000</td><td>0.000000</td><td>6,5</td><td>79,2</td><td>693</td><td>1,95</td><td>24</td><td>46,3</td><td>79,2</td><td>3,2</td></tr>
<tr><td>>tr|A0A124BUY4|A0A124BUY4_9MYCO Serine/threonine phosphatase PstP</td><td>1</td><td>NO_SP</td><td>1.000019</td><td>0.000058</td><td>0.000000</td><td>0.000000</td><td>0.000000</td><td>0.000000</td><td>4,7</td><td>54,0</td><td>515</td><td>1,95</td><td>8</td><td>23,7</td><td>54,0</td><td>4,4</td></tr>
<tr><td>>tr|A0A2Z5YLT1|A0A2Z5YLT1_MYCMR Acetyl-CoA acetyltransferase</td><td>0</td><td>NO_SP</td><td>1.000031</td><td>0.000000</td><td>0.000000</td><td>0.000000</td><td>0.000000</td><td>0.000000</td><td>4,6</td><td>40,0</td><td>381</td><td>1,94</td><td>1</td><td>2,4</td><td>40,0</td><td>4,4</td></tr>
<tr><td>>tr|A0A100I1Z7|A0A100I1Z7_9MYCO Acyl-CoA dehydrogenase, short-chain specific</td><td>0</td><td>NO_SP</td><td>1.000069</td><td>0.000001</td><td>0.000000</td><td>0.000000</td><td>0.000000</td><td>0.000000</td><td>4,8</td><td>75,8</td><td>706</td><td>1,94</td><td>27</td><td>58,6</td><td>75,8</td><td>8,3</td></tr>
<tr><td>>tr|B2HNQ0|B2HNQ0_MYCMM PPE family protein</td><td>0</td><td>NO_SP</td><td>0.999863</td><td>0.000193</td><td>0.000002</td><td>0.000000</td><td>0.000000</td><td>0.000000</td><td>3,9</td><td>37,2</td><td>367</td><td>1,92</td><td>3</td><td>13,1</td><td>37,2</td><td>9,0</td></tr>
<tr><td>>sp|B2HSL2|EFG_MYCMM Elongation factor G</td><td>0</td><td>NO_SP</td><td>1.000077</td><td>0.000000</td><td>0.000000</td><td>0.000000</td><td>0.000000</td><td>0.000000</td><td>4,7</td><td>77,0</td><td>701</td><td>1,88</td><td>40</td><td>75,9</td><td>77,0</td><td>4,9</td></tr>
<tr><td>>tr|B2HDW7|B2HDW7_MYCMM Glycerol-3-phosphate dehydrogenase</td><td>0</td><td>NO_SP</td><td>0.999939</td><td>0.000095</td><td>0.000002</td><td>0.000000</td><td>0.000000</td><td>0.000000</td><td>6,2</td><td>63,3</td><td>585</td><td>1,88</td><td>17</td><td>50,4</td><td>63,3</td><td>4,1</td></tr>
<tr><td>>tr|A0A2Z5YE17|A0A2Z5YE17_MYCMR Transketolase</td><td>0</td><td>NO_SP</td><td>1.000034</td><td>0.000001</td><td>0.000000</td><td>0.000000</td><td>0.000000</td><td>0.000000</td><td>4,6</td><td>75,2</td><td>700</td><td>1,87</td><td>25</td><td>53,9</td><td>75,2</td><td>16,1</td></tr>
<tr><td>>tr|A0A2Z5YM52|A0A2Z5YM52_MYCMR Aldehyde dehydrogenase</td><td>0</td><td>NO_SP</td><td>1.000039</td><td>0.000007</td><td>0.000000</td><td>0.000000</td><td>0.000000</td><td>0.000000</td><td>4,9</td><td>54,9</td><td>507</td><td>1,87</td><td>10</td><td>30,4</td><td>54,9</td><td>7,9</td></tr>
<tr><td>>tr|A0A2Z5YBG4|A0A2Z5YBG4_MYCMR Uncharacterized protein</td><td>0</td><td>NO_SP</td><td>1.000042</td><td>0.000001</td><td>0.000000</td><td>0.000000</td><td>0.000000</td><td>0.000000</td><td>4,8</td><td>30,8</td><td>281</td><td>1,87</td><td>10</td><td>58,4</td><td>30,8</td><td>11,3</td></tr>
<tr><td>>tr|B2HN30|B2HN30_MYCMM Acyl-CoA dehydrogenase FadE5</td><td>0</td><td>NO_SP</td><td>1.000051</td><td>0.000000</td><td>0.000000</td><td>0.000000</td><td>0.000000</td><td>0.000000</td><td>4,8</td><td>66,4</td><td>611</td><td>1,87</td><td>28</td><td>58,3</td><td>66,4</td><td>4,0</td></tr>
<tr><td>>tr|A0A3E2MP68|A0A3E2MP68_MYCMR Aldehyde dehydrogenase</td><td>0</td><td>NO_SP</td><td>1.000063</td><td>0.000000</td><td>0.000000</td><td>0.000000</td><td>0.000000</td><td>0.000000</td><td>9,4</td><td>53,4</td><td>493</td><td>1,83</td><td>23</td><td>59,2</td><td>53,4</td><td>5,9</td></tr>
<tr><td>>tr|A0A2Z5YI10|A0A2Z5YI10_MYCMR Chaperone protein HtpG</td><td>0</td><td>NO_SP</td><td>1.000056</td><td>0.000000</td><td>0.000000</td><td>0.000000</td><td>0.000000</td><td>0.000000</td><td>4,5</td><td>72,8</td><td>648</td><td>1,82</td><td>35</td><td>63,6</td><td>72,8</td><td>4,4</td></tr>
<tr><td>>tr|A0A2Z5YF25|A0A2Z5YF25_MYCMR Tryptophan synthase beta chain</td><td>0</td><td>NO_SP</td><td>1.000076</td><td>0.000000</td><td>0.000000</td><td>0.000000</td><td>0.000000</td><td>0.000000</td><td>5,9</td><td>45,1</td><td>425</td><td>1,80</td><td>12</td><td>40,9</td><td>45,1</td><td>4,6</td></tr>
<tr><td>>tr|A0A2Z5YFA5|A0A2Z5YFA5_MYCMR Malate synthase G</td><td>0</td><td>NO_SP</td><td>1.000038</td><td>0.000000</td><td>0.000000</td><td>0.000000</td><td>0.000000</td><td>0.000000</td><td>4,7</td><td>79,0</td><td>727</td><td>1,79</td><td>31</td><td>56,8</td><td>79,0</td><td>5,7</td></tr>
<tr><td>>tr|B2HSP5|B2HSP5_MYCMM Conserved short-chain dehydrogenase</td><td>0</td><td>NO_SP</td><td>1.000070</td><td>0.000000</td><td>0.000000</td><td>0.000000</td><td>0.000000</td><td>0.000000</td><td>9,8</td><td>26,9</td><td>265</td><td>1,79</td><td>7</td><td>31,3</td><td>26,9</td><td>3,4</td></tr>
<tr><td>>tr|A0A2Z5YDI3|A0A2Z5YDI3_MYCMR Chlorite dismutase</td><td>0</td><td>NO_SP</td><td>1.000052</td><td>0.000002</td><td>0.000000</td><td>0.000000</td><td>0.000000</td><td>0.000000</td><td>5,6</td><td>26,4</td><td>231</td><td>1,79</td><td>9</td><td>54,5</td><td>26,4</td><td>4,6</td></tr>
<tr><td>>tr|B2HJ43|B2HJ43_MYCMM Lysine--tRNA ligase</td><td>0</td><td>NO_SP</td><td>1.000061</td><td>0.000000</td><td>0.000000</td><td>0.000000</td><td>0.000000</td><td>0.000000</td><td>4,7</td><td>55,5</td><td>498</td><td>1,78</td><td>19</td><td>49,6</td><td>55,5</td><td>5,5</td></tr>
<tr><td>>tr|A0A2Z5YCI7|A0A2Z5YCI7_MYCMR Acyltransferase PapA5</td><td>0</td><td>NO_SP</td><td>1.000059</td><td>0.000000</td><td>0.000000</td><td>0.000000</td><td>0.000000</td><td>0.000000</td><td>4,6</td><td>45,4</td><td>414</td><td>1,77</td><td>13</td><td>35,7</td><td>45,4</td><td>4,1</td></tr>
<tr><td>>tr|A0A2Z5Y912|A0A2Z5Y912_MYCMR (Fe-S)-binding protein</td><td>3</td><td>NO_SP</td><td>0.998132</td><td>0.001708</td><td>0.000079</td><td>0.000001</td><td>0.000000</td><td>0.000069</td><td>6,7</td><td>102,8</td><td>970</td><td>1,77</td><td>26</td><td>36,5</td><td>102,8</td><td>4,1</td></tr>
<tr><td>>tr|B2HHR8|B2HHR8_MYCMM Acetyl/propionyl-CoA carboxylase (Beta subunit) AccD6</td><td>0</td><td>NO_SP</td><td>0.999938</td><td>0.000090</td><td>0.000001</td><td>0.000000</td><td>0.000000</td><td>0.000000</td><td>6,1</td><td>50,1</td><td>473</td><td>1,76</td><td>23</td><td>61,5</td><td>50,1</td><td>8,9</td></tr>
<tr><td>>tr|A0A2Z5YEM3|A0A2Z5YEM3_MYCMR Tyrosine--tRNA ligase</td><td>0</td><td>NO_SP</td><td>1.000063</td><td>0.000001</td><td>0.000000</td><td>0.000000</td><td>0.000000</td><td>0.000000</td><td>5,8</td><td>44,8</td><td>411</td><td>1,76</td><td>1</td><td>5,8</td><td>44,8</td><td>19,6</td></tr>
<tr><td>>tr|A0A2Z5Y9U7|A0A2Z5Y9U7_MYCMR Cyclopropane mycolic acid synthase</td><td>0</td><td>NO_SP</td><td>1.000057</td><td>0.000000</td><td>0.000000</td><td>0.000000</td><td>0.000000</td><td>0.000000</td><td>6,3</td><td>32,8</td><td>287</td><td>1,75</td><td>16</td><td>69</td><td>32,8</td><td>3,1</td></tr>
<tr><td>>tr|A0A2Z5YHF6|A0A2Z5YHF6_MYCMR Glutamine synthetase</td><td>0</td><td>NO_SP</td><td>1.000043</td><td>0.000000</td><td>0.000000</td><td>0.000000</td><td>0.000000</td><td>0.000000</td><td>4,7</td><td>53,5</td><td>478</td><td>1,75</td><td>23</td><td>69</td><td>53,5</td><td>6,2</td></tr>
<tr><td>>sp|B2HSJ3|RPOB_MYCMM DNA-directed RNA polymerase subunit beta</td><td>0</td><td>NO_SP</td><td>1.000055</td><td>0.000001</td><td>0.000000</td><td>0.000000</td><td>0.000000</td><td>0.000000</td><td>4,7</td><td>129,5</td><td>1176</td><td>1,74</td><td>2</td><td>4,3</td><td>129,6</td><td>4,1</td></tr>
<tr><td>>tr|A0A100I4E0|A0A100I4E0_9MYCO MCE associated membrane protein</td><td>1</td><td>NO_SP</td><td>1.000043</td><td>0.000001</td><td>0.000000</td><td>0.000000</td><td>0.000000</td><td>0.000000</td><td>4,3</td><td>27,7</td><td>264</td><td>1,74</td><td>5</td><td>22,7</td><td>27,7</td><td>3,3</td></tr>
<tr><td>>tr|A0A2Z5YA71|A0A2Z5YA71_MYCMR Putative ABC transporter ATP-binding protein</td><td>0</td><td>NO_SP</td><td>1.000031</td><td>0.000000</td><td>0.000000</td><td>0.000000</td><td>0.000000</td><td>0.000000</td><td>5,0</td><td>35,9</td><td>328</td><td>1,74</td><td>14</td><td>51,5</td><td>35,9</td><td>7,0</td></tr>
<tr><td>>tr|A0A2Z5YE10|A0A2Z5YE10_MYCMR Enoyl-[acyl-carrier-protein] reductase [NADH]</td><td>0</td><td>NO_SP</td><td>1.000068</td><td>0.000000</td><td>0.000000</td><td>0.000000</td><td>0.000000</td><td>0.000000</td><td>5,3</td><td>28,7</td><td>269</td><td>1,73</td><td>11</td><td>57,2</td><td>28,7</td><td>3,3</td></tr>
<tr><td>>tr|A0A2Z5YDR3|A0A2Z5YDR3_MYCMR Probable cell division protein WhiA</td><td>0</td><td>NO_SP</td><td>1.000067</td><td>0.000000</td><td>0.000000</td><td>0.000000</td><td>0.000000</td><td>0.000000</td><td>10,7</td><td>35,1</td><td>327</td><td>1,72</td><td>13</td><td>55,7</td><td>35,1</td><td>3,0</td></tr>
<tr><td>>tr|B2HJW4|B2HJW4_MYCMM Uncharacterized protein</td><td>0</td><td>NO_SP</td><td>1.000046</td><td>0.000001</td><td>0.000000</td><td>0.000000</td><td>0.000000</td><td>0.000000</td><td>4,9</td><td>35,4</td><td>326</td><td>1,72</td><td>1</td><td>3,7</td><td>35,4</td><td>5,0</td></tr>
<tr><td>>tr|A0A2Z5YHT0|A0A2Z5YHT0_MYCMR Uncharacterized protein</td><td>0</td><td>NO_SP</td><td>1.000040</td><td>0.000001</td><td>0.000000</td><td>0.000000</td><td>0.000000</td><td>0.000000</td><td>4,8</td><td>56,2</td><td>520</td><td>1,71</td><td>17</td><td>55,2</td><td>56,2</td><td>3,8</td></tr>
<tr><td>>tr|A0A2Z5YA54|A0A2Z5YA54_MYCMR Methoxy mycolic acid synthase MmaA3</td><td>0</td><td>NO_SP</td><td>1.000072</td><td>0.000002</td><td>0.000000</td><td>0.000000</td><td>0.000000</td><td>0.000000</td><td>6,3</td><td>33,6</td><td>293</td><td>1,70</td><td>16</td><td>74,4</td><td>33,6</td><td>4,8</td></tr>
<tr><td>>tr|A0A2Z5Y8P1|A0A2Z5Y8P1_MYCMR Succinate-semialdehyde dehydrogenase [NADP(+)] 1</td><td>0</td><td>NO_SP</td><td>1.000004</td><td>0.000040</td><td>0.000000</td><td>0.000000</td><td>0.000000</td><td>0.000000</td><td>4,5</td><td>48,6</td><td>458</td><td>1,69</td><td>1</td><td>5,7</td><td>48,6</td><td>3,8</td></tr>
<tr><td>>tr|A0A2Z5YGG4|A0A2Z5YGG4_MYCMR DUF4333 domain-containing protein</td><td>1</td><td>NO_SP</td><td>0.918212</td><td>0.080818</td><td>0.000248</td><td>0.000167</td><td>0.000111</td><td>0.000483</td><td>6,9</td><td>10,9</td><td>103</td><td>1,69</td><td>6</td><td>62,1</td><td>10,9</td><td>5,1</td></tr>
<tr><td>>tr|B2HIM5|B2HIM5_MYCMM Daunorubicin-DIM-transport integral membrane protein ABC transporter DrrB</td><td>6</td><td>NO_SP</td><td>0.999926</td><td>0.000086</td><td>0.000000</td><td>0.000000</td><td>0.000000</td><td>0.000000</td><td>10,7</td><td>31,0</td><td>289</td><td>1,68</td><td>5</td><td>21,8</td><td>31,0</td><td>7,3</td></tr>
<tr><td>>tr|B2HIS9|B2HIS9_MYCMM ATP-dependent 6-phosphofructokinase</td><td>0</td><td>NO_SP</td><td>1.000062</td><td>0.000002</td><td>0.000000</td><td>0.000000</td><td>0.000000</td><td>0.000000</td><td>5,8</td><td>39,8</td><td>377</td><td>1,68</td><td>10</td><td>49,3</td><td>39,8</td><td>3,1</td></tr>
<tr><td>>tr|B2HH22|B2HH22_MYCMM Amino acid aminotransferase, PabC</td><td>0</td><td>NO_SP</td><td>1.000086</td><td>0.000000</td><td>0.000000</td><td>0.000000</td><td>0.000000</td><td>0.000000</td><td>6,2</td><td>31,4</td><td>296</td><td>1,68</td><td>10</td><td>57,8</td><td>31,4</td><td>3,5</td></tr>
<tr><td>>tr|A0A2Z5YG19|A0A2Z5YG19_MYCMR Cytochrome P450</td><td>0</td><td>NO_SP</td><td>1.000062</td><td>0.000000</td><td>0.000000</td><td>0.000000</td><td>0.000000</td><td>0.000000</td><td>5,0</td><td>46,9</td><td>424</td><td>1,66</td><td>10</td><td>37</td><td>46,9</td><td>5,7</td></tr>
<tr><td>>tr|B2HT54|B2HT54_MYCMM Fumarate hydratase class II</td><td>0</td><td>NO_SP</td><td>1.000020</td><td>0.000011</td><td>0.000001</td><td>0.000000</td><td>0.000000</td><td>0.000000</td><td>4,7</td><td>50,2</td><td>476</td><td>1,66</td><td>12</td><td>34,7</td><td>50,2</td><td>3,0</td></tr>
<tr><td>>tr|A0A2Z5YDG5|A0A2Z5YDG5_MYCMR Transaldolase</td><td>0</td><td>NO_SP</td><td>1.000060</td><td>0.000000</td><td>0.000000</td><td>0.000000</td><td>0.000000</td><td>0.000000</td><td>4,4</td><td>40,1</td><td>373</td><td>1,65</td><td>18</td><td>72,1</td><td>40,1</td><td>4,6</td></tr>
<tr><td>>tr|A0A2Z5YF13|A0A2Z5YF13_MYCMR</td><td>0</td><td>NO_SP</td><td>1.000063</td><td>0.000000</td><td>0.000000</td><td>0.000000</td><td>0.000000</td><td>0.000000</td><td>8,9</td><td>41,6</td><td>378</td><td>1,64</td><td>11</td><td>41,3</td><td>41,6</td><td>3,2</td></tr>
<tr><td>>tr|A0A117DWS6|A0A117DWS6_9MYCO UPF0182 protein MPS_3353</td><td>7</td><td>NO_SP</td><td>1.000047</td><td>0.000000</td><td>0.000000</td><td>0.000000</td><td>0.000000</td><td>0.000000</td><td>9,2</td><td>107,3</td><td>987</td><td>1,62</td><td>23</td><td>37,5</td><td>107,3</td><td>6,2</td></tr>
<tr><td>>tr|A0A100I4V0|A0A100I4V0_9MYCO Arylamine N-acetyltransferase</td><td>0</td><td>NO_SP</td><td>1.000068</td><td>0.000000</td><td>0.000000</td><td>0.000000</td><td>0.000000</td><td>0.000000</td><td>4,9</td><td>30,7</td><td>280</td><td>1,62</td><td>8</td><td>33,6</td><td>30,7</td><td>3,8</td></tr>
<tr><td>>tr|A0A2Z5YE68|A0A2Z5YE68_MYCMR Modulator of FtsH protease HflK</td><td>1</td><td>NO_SP</td><td>0.898437</td><td>0.100314</td><td>0.000312</td><td>0.000190</td><td>0.000132</td><td>0.000645</td><td>5,1</td><td>41,8</td><td>384</td><td>1,60</td><td>11</td><td>42,7</td><td>41,8</td><td>5,2</td></tr>
<tr><td>>tr|B2HSZ6|B2HSZ6_MYCMM NADH dehydrogenase Ndh</td><td>1</td><td>NO_SP</td><td>1.000075</td><td>0.000002</td><td>0.000000</td><td>0.000000</td><td>0.000000</td><td>0.000000</td><td>9,2</td><td>49,7</td><td>461</td><td>1,60</td><td>15</td><td>51</td><td>49,7</td><td>4,5</td></tr>
<tr><td>>tr|A0A2Z5YGZ7|A0A2Z5YGZ7_MYCMR Polyketide synthase</td><td>0</td><td>NO_SP</td><td>1.000049</td><td>0.000001</td><td>0.000000</td><td>0.000000</td><td>0.000000</td><td>0.000000</td><td>5,3</td><td>54,3</td><td>511</td><td>1,59</td><td>10</td><td>28,6</td><td>54,3</td><td>5,8</td></tr>
<tr><td>>tr|B2HPT1|B2HPT1_MYCMM Conserved secreted protein</td><td>1</td><td>NO_SP</td><td>1.000086</td><td>0.000000</td><td>0.000000</td><td>0.000000</td><td>0.000000</td><td>0.000000</td><td>10,4</td><td>32,2</td><td>284</td><td>1,58</td><td>8</td><td>37,3</td><td>32,2</td><td>6,7</td></tr>
<tr><td>>tr|A0A2Z5YA28|A0A2Z5YA28_MYCMR Hydroxymycolate synthase MmaA4</td><td>0</td><td>NO_SP</td><td>1.000056</td><td>0.000000</td><td>0.000000</td><td>0.000000</td><td>0.000000</td><td>0.000000</td><td>5,0</td><td>34,4</td><td>299</td><td>1,54</td><td>16</td><td>75,6</td><td>34,4</td><td>5,5</td></tr>
<tr><td>>tr|A0A2Z5YF84|A0A2Z5YF84_MYCMR ESX-5 secretion-associated protein EspG5</td><td>0</td><td>NO_SP</td><td>1.000059</td><td>0.000000</td><td>0.000000</td><td>0.000000</td><td>0.000000</td><td>0.000000</td><td>4,6</td><td>32,4</td><td>300</td><td>1,53</td><td>10</td><td>49</td><td>32,4</td><td>4,3</td></tr>
<tr><td>>tr|B2HS37|B2HS37_MYCMM Conserved hypothetical membrane protein</td><td>2</td><td>NO_SP</td><td>1.000059</td><td>0.000002</td><td>0.000000</td><td>0.000000</td><td>0.000000</td><td>0.000000</td><td>8,9</td><td>18,5</td><td>175</td><td>1,51</td><td>5</td><td>49,7</td><td>18,5</td><td>4,3</td></tr>
<tr><td>>tr|A0A2Z5YEW0|A0A2Z5YEW0_MYCMR L-gulono-1,4-lactone dehydrogenase</td><td>0</td><td>NO_SP</td><td>1.000056</td><td>0.000000</td><td>0.000000</td><td>0.000000</td><td>0.000000</td><td>0.000000</td><td>6,4</td><td>48,1</td><td>428</td><td>1,50</td><td>19</td><td>60,5</td><td>48,1</td><td>10,3</td></tr>
<tr><td>>sp|B2HQK2|ATPB_MYCMM ATP synthase subunit beta</td><td>0</td><td>NO_SP</td><td>1.000035</td><td>0.000000</td><td>0.000000</td><td>0.000000</td><td>0.000000</td><td>0.000000</td><td>4,6</td><td>52,7</td><td>483</td><td>1,50</td><td>30</td><td>82</td><td>52,7</td><td>5,3</td></tr>
<tr><td>>tr|A0A2Z5Y9S2|A0A2Z5Y9S2_MYCMR Mycolic acid methyltransferase MmaA1</td><td>0</td><td>NO_SP</td><td>1.000054</td><td>0.000004</td><td>0.000000</td><td>0.000000</td><td>0.000000</td><td>0.000000</td><td>4,6</td><td>33,0</td><td>286</td><td>1,49</td><td>15</td><td>59,8</td><td>33,0</td><td>3,3</td></tr>
<tr><td>>tr|B2HRY7|B2HRY7_MYCMM Succinate-semialdehyde dehydrogenase [NADP+] dependent (SsdH) GabD2</td><td>0</td><td>NO_SP</td><td>1.000066</td><td>0.000000</td><td>0.000000</td><td>0.000000</td><td>0.000000</td><td>0.000000</td><td>6,5</td><td>55,2</td><td>518</td><td>1,48</td><td>12</td><td>33,4</td><td>55,2</td><td>3,3</td></tr>
<tr><td>>tr|A0A100I1G9|A0A100I1G9_9MYCO UDP-glucose 4-epimerase</td><td>0</td><td>NO_SP</td><td>1.000031</td><td>0.000002</td><td>0.000000</td><td>0.000000</td><td>0.000000</td><td>0.000000</td><td>5,7</td><td>33,6</td><td>314</td><td>1,46</td><td>3</td><td>17,2</td><td>33,6</td><td>8,6</td></tr>
<tr><td>>tr|A0A2Z5YMW9|A0A2Z5YMW9_MYCMR SIMPL domain-containing protein</td><td>0</td><td>LIPO</td><td>0.000000</td><td>0.000001</td><td>1.000072</td><td>0.000000</td><td>0.000000</td><td>0.000000</td><td>4,6</td><td>24,3</td><td>235</td><td>1,45</td><td>5</td><td>34,9</td><td>24,3</td><td>3,2</td></tr>
<tr><td>>tr|A0A117DY41|A0A117DY41_9MYCO Cysteine desulfurase</td><td>0</td><td>NO_SP</td><td>1.000041</td><td>0.000002</td><td>0.000000</td><td>0.000000</td><td>0.000000</td><td>0.000000</td><td>5,8</td><td>41,2</td><td>391</td><td>1,40</td><td>9</td><td>35,3</td><td>41,2</td><td>7,9</td></tr>
<tr><td>>tr|A0A3E2MR68|A0A3E2MR68_MYCMR Transcriptional regulatory protein EmbR</td><td>0</td><td>NO_SP</td><td>1.000053</td><td>0.000000</td><td>0.000000</td><td>0.000000</td><td>0.000000</td><td>0.000000</td><td>7,0</td><td>42,0</td><td>386</td><td>1,39</td><td>12</td><td>40,2</td><td>42,0</td><td>3,7</td></tr>
<tr><td>>tr|B2HGT1|B2HGT1_MYCMM Geranylgeranyl pyrophosphate synthetase IdsA2</td><td>0</td><td>NO_SP</td><td>1.000045</td><td>0.000002</td><td>0.000000</td><td>0.000000</td><td>0.000000</td><td>0.000000</td><td>5,7</td><td>36,4</td><td>340</td><td>1,36</td><td>5</td><td>16,8</td><td>36,4</td><td>3,2</td></tr>
<tr><td>>tr|B2HNV0|B2HNV0_MYCMM Fatty-acid-CoA ligase FadD2</td><td>0</td><td>NO_SP</td><td>1.000087</td><td>0.000000</td><td>0.000000</td><td>0.000000</td><td>0.000000</td><td>0.000000</td><td>5,8</td><td>60,3</td><td>557</td><td>1,36</td><td>18</td><td>49,2</td><td>60,3</td><td>8,6</td></tr>
</table>

>tr|A0A2Z5YHL1|A0A2Z5YHL1_MYCMR Long-chain-fatty-acid--CoA ligase	0	NO_SP	0.999987	0.000061	0.000002	0.000000	0.000000	0.000000	5,6	64,6	600	1,34	29	63,8	64,6	5,4
>tr|A0A2Z5Y9G7|A0A2Z5Y9G7_MYCMR Peptidase	0	NO_SP	1.000060	0.000003	0.000000	0.000000	0.000000	0.000000	4,6	77,4	698	1,33	14	28,1	77,4	3,8
>tr|A0A100I2Q3|A0A100I2Q3_9MYCO Histidine kinase	1	NO_SP	1.000068	0.000000	0.000000	0.000000	0.000000	0.000000	5,9	44,4	411	1,32	13	50,6	44,4	2,8
>tr|A0A100IEF6|A0A100IEF6_9MYCO Medium chain fatty-acid-CoA ligase FadD14	0	NO_SP	0.999874	0.000158	0.000003	0.000000	0.000000	0.000000	5,2	58,9	539	1,29	11	24,7	58,9	3,3
>tr|A0A2Z5YE07|A0A2Z5YE07_MYCMR Carboxymuconolactone decarboxylase family protein	0	NO_SP	1.000041	0.000000	0.000000	0.000000	0.000000	0.000000	4,8	20,6	187	1,28	11	80,7	20,6	15,5
>tr|B2HNQ3|B2HNQ3_MYCMM Conserved hypothetical alanine and proline rich membrane protein	0	NO_SP	1.000064	0.000000	0.000000	0.000000	0.000000	0.000000	5,3	91,8	879	1,26	14	22	91,8	2,9
>tr|B2HF81|B2HF81_MYCMM Citrate synthase	0	NO_SP	1.000022	0.000011	0.000000	0.000000	0.000000	0.000000	5,4	48,2	431	1,25	21	54,8	48,2	2,8
>tr|A0A2Z5YLZ1|A0A2Z5YLZ1_MYCMR TIGR04255 family protein	0	NO_SP	1.000069	0.000000	0.000000	0.000000	0.000000	0.000000	4,8	29,7	270	1,25	13	68,1	29,7	4,9
>tr|B2HJ06|B2HJ06_MYCMM Acyl-CoA dehydrogenase	0	NO_SP	1.000043	0.000001	0.000000	0.000000	0.000000	0.000000	6,1	43,2	394	1,24	6	20,8	43,2	6,8
>tr|A0A3E2MYK8|A0A3E2MYK8_MYCMR 50S ribosomal protein L2	0	NO_SP	1.000067	0.000000	0.000000	0.000000	0.000000	0.000000	11,9	30,4	280	1,11	8	41,1	30,4	3,5
>tr|B2HIM7|B2HIM7_MYCMM Phenolpthiocerol synthesis type-I polyketide synthase PpsE	0	NO_SP	1.000019	0.000020	0.000007	0.000000	0.000000	0.000000	4,9	158,0	1481	1,10	1	1	158,0	3,4
>tr|B2HD57|B2HD57_MYCMM L-lactate dehydrogenase (Cytochrome) LldD2	0	NO_SP	1.000039	0.000000	0.000000	0.000000	0.000000	0.000000	8,7	45,6	414	1,08	24	71,3	45,6	3,0
