# Supplementary material for: In vitro and ex vivo proteomics of Mycobacterium marinum biofilms and the development of biofilm-binding synthetic nanobodies
Source: mSystems. 2023 May 15;8(3):e01073-22. doi: 10.1128/msystems.01073-22 (PMC10308901; doi:10.1128/msystems.01073-22)
Supplement: Table S3 — List of proteins (n, 904) with significantly higher abundancies (unpaired T-test and p < 0.05, cells in deep blue) on biofilm cells in comparison to planktonic cells. Cells shaded in red indicate proteins showing signficantly increased abundance on the 4-week-old biofilm matrices in comparison to their equivalent proteins on the planktonic cell surfaces. Cells shaded in yellow show the LFQ values for GroEL1 on 2-day-, 1-week- and 2-week-old biofilm matrices with T-test statistics for pairwise comparisons. log, planktonic cells; B, submerged-type biofilms; P, pellicle-type biofilms. [file msystems.01073-22-s0003.pdf]

Table S3. List of proteins (n, 904) with significantly higher abundancies (unpaired T-test and $p < 0.05$, cells in deep blue) on biofilm cells in comparison to planktonic cells. Cells shaded in red indicate proteins showing signficantly increased abundance on the 4-week-old biofilm matrices in comparison to their equivalent proteins on the planktonic cell surfaces. Cells shaded in yellow show the LFQ values for GroEL1 on 2-day-, 1-week- and 2-week-old biofilm matrices with T-test statistics for pairwise comparisons. log, planktonic cells; B, submerged-type biofilms; P, pellicle-type biofilms.

Protein ID	UniProtKB/AC_ID	T-test difference (p < 0.05)		T-test Significant	T-test Significant	T-test Difference 4w P_log	T-test Difference 4w B_log	T-test Significant 1w B_log	T-test Significant 2w B_log	T-test Difference 2d B_log	T-test Difference 1w B_log
		more on B than on log (log10)	more on P than on log (log10)	Change 4w B > log sign.	Change 4w P > log sign.						
A0A100HYY8_9MYCO	Protein translocase subunit SecD	1,00	2,98	+	+	0,2845	1,0000	+	+	0,7124	0,9278
A0A100I0M9_9MYCO	Membrane protein	1,46	6,48	+	+	0,9727	1,4572	+	+	0,9274	1,1962
A0A100I205_9MYCO	4-hydroxy-tetrahydrodipicolinate reductase	0,16	2,59	+	+	0,3108	0,1583	+		-0,2760	-0,1865
A0A100I248_9MYCO	S-adenosyl-L-methionine-dependent methyltransferase	0,85	7,80	+	+	0,8602	0,8513	+	+	0,5573	0,8576
A0A100I2Q3_9MYCO	Histidine kinase	0,87	10,13	+	+	0,4427	0,8681	+	+	0,5961	0,8643
A0A100I428_9MYCO	Lipoprotein	0,60	2,70	+	+	0,6467	0,5971	+	+	0,3466	0,7473
A0A100I4E0_9MYCO	MCE associated membrane protein	0,61	3,44	+	+	0,3692	0,6083	+	+	0,4001	0,5758
A0A100I552_9MYCO	Uncharacterized protein	0,85	10,28	+	+	0,7188	0,8507	+	+	0,8523	1,1415
A0A100I697_9MYCO	Phosphoserine phosphatase	0,62	3,14	+	+	0,3684	0,6245	+	+	0,5941	0,5137
A0A100I7F0_9MYCO	Cytochrome bc1 complex cytochrome c subunit	0,91	3,19	+	+	0,5080	0,9115	+	+	0,7410	0,9001
A0A100I7H0_9MYCO	Cytochrome-c oxidase	0,53	2,25	+	+	0,1419	0,5274	+	+	0,2778	0,4921
A0A100I852_9MYCO	Uncharacterized protein	0,45	6,85	+	+	0,6825	0,4504	+	+	0,7198	0,4495
A0A100I8G4_9MYCO	Tuberculin related peptide	0,87	2,40	+	+	0,7448	0,8682	+	+	0,0000	0,6586
A0A100I8J2_9MYCO	Alpha/beta hydrolase	0,62	3,84	+	+	0,3086	0,6162	+	+	0,1907	0,3625
A0A100IA48_9MYCO	Conserved exported protein of uncharacterized function	0,32	13,02	+	+	1,1079	0,3234	+		0,7363	0,4599
A0A100IAW2_9MYCO	Non-specific serine/threonine protein kinase	0,69	4,82	+	+	0,2593	0,6923	+	+	0,3926	0,6141
A0A100IBA5_9MYCO	Aminotransferase	0,38	2,24	+	+	0,4840	0,3787			0,4094	0,3939
A0A100IEF6_9MYCO	Medium chain fatty-acid-CoA ligase FadD14	0,38	3,83	+	+	0,5341	0,3767		+	0,3147	0,2464
A0A100IEI1_9MYCO	Calcium dodecin	0,74	3,21	+	+	0,7176	0,7429	+	+	0,1063	0,5225
A0A100IF17_9MYCO	Type III pantothenate kinase	0,74	13,77	+	+	0,7370	0,7402	+	+	0,0173	0,5468
A0A100IFA3_9MYCO	Formaldehyde dehydrogenase	0,61	5,62	+	+	0,8693	0,6067	+	+	0,0489	0,3847
A0A117DTH3_9MYCO	Asparaginase	0,48	2,90	+	+	0,4246	0,4818	+	+	0,4320	0,3682
A0A117E058_9MYCO	Serine protease	0,43	2,36	+	+	0,4033	0,4285		+	-0,0727	0,2104
A0A124BUJ3_9MYCO	Proteasome subunit beta	0,33	3,60	+	+	0,4477	0,3290	+		-0,0884	0,4027
A0A124BUY4_9MYCO	Serine/threonine phosphatase PstP	0,80	9,25	+	+	0,6101	0,8018	+	+	0,6434	0,8245
A0A124BW47_9MYCO	Non-ribosomal peptide synthetase	1,38	4,42	+	+	0,6198	1,3762	+	+	0,8006	1,1443
A0A124BWV5_9MYCO	L-cysteine:1D-myo-inositol 2-amino-2-deoxy-alpha-D-glucopyranoside ligase	0,61	2,56	+	+	0,4313	0,6056	+	+	0,2267	0,2515
A0A2Z5Y7F8_MYCMR	Cell wall synthesis protein CwsA	0,89	2,56	+	+	0,6078	0,8854	+	+	0,7031	0,8206
A0A2Z5Y7G0_MYCMR	DUF3566 domain-containing protein	0,51	3,40	+	+	0,1814	0,5060	+	+	0,5035	0,6995
A0A2Z5Y7G7_MYCMR	Serine/threonine-protein kinase PknB	0,44	2,93	+	+	0,1808	0,4441	+	+	0,2705	0,4032
A0A2Z5Y7G9_MYCMR	Non-specific serine/threonine protein kinase	0,42	3,75	+	+	0,2375	0,4199	+	+	0,3707	0,3980
A0A2Z5Y7J6_MYCMR	LLM class F420-dependent oxidoreductase	0,48	8,11	+	+	0,8260	0,4752	+	+	0,1864	0,2286
A0A2Z5Y7L1_MYCMR	Transcriptional regulator	0,64	2,95	+	+	0,5042	0,6439	+	+	0,1762	0,3895
A0A2Z5Y7R9_MYCMR	Uncharacterized protein	0,13	7,55	+	+	0,5050	0,1349	+	+	-0,1067	-0,1946
A0A2Z5Y7T5_MYCMR	LLM class F420-dependent oxidoreductase	0,26	6,44	+	+	0,6555	0,2609		+	-0,1570	0,0732
A0A2Z5Y7T8_MYCMR	Isocitrate dehydrogenase [NADP]	0,18	4,63	+	+	0,2310	0,1772	+	+	0,1460	0,1829
A0A2Z5Y830_MYCMR	Adenosylhomocysteine nucleosidase	0,79	2,60	+	+	0,2113	0,7909	+	+	0,2335	0,6968
A0A2Z5Y835_MYCMR	Ketoacyl reductase	0,14	2,43	+	+	0,1170	0,1428	+	+	0,1105	0,2511
A0A2Z5Y849_MYCMR	Enoyl-CoA hydratase	0,30	5,62	+	+	0,7551	0,3033		+	0,0404	0,1865
A0A2Z5Y859_MYCMR	Putative dehydrogenase	1,02	3,69	+	+	0,3320	1,0248	+	+	0,7165	0,9832
A0A2Z5Y884_MYCMR	Polyketide cyclase / dehydrase and lipid transport	0,84	4,16	+	+	0,7735	0,8374	+	+	0,8235	0,9534
A0A2Z5Y8C3_MYCMR	Quinone oxidoreductase	0,64	6,64	+	+	0,6180	0,6352	+	+	0,4625	0,4678
A0A2Z5Y8E3_MYCMR	3-alpha-hydroxysteroid dehydrogenase	0,46	12,20	+	+	0,7262	0,4640	+	+	0,0258	0,2565
A0A2Z5Y8F1_MYCMR	Acyl-CoA dehydrogenase	0,54	2,81	+	+	0,4340	0,5388	+	+	0,2184	0,3370

A0A2Z5Y8J9_MYCMR	Dihydroxy-acid dehydratase	0,27	3,16	+	+	0,2684	0,2740	+	+	0,1299	0,1924
A0A2Z5Y8P3_MYCMR	Lysophospholipase	0,19	6,04	+	+	0,3399	0,1918	+	+	0,0919	0,1707
A0A2Z5Y8S6_MYCMR	Acyl-CoA dehydrogenase	0,28	5,66	+	+	0,2740	0,2824	+	+	-0,0070	0,1428
A0A2Z5Y8T5_MYCMR	Membrane protein	0,61	12,29	+	+	0,3765	0,6129	+	+	0,1580	0,6424
A0A2Z5Y8U2_MYCMR	Diacylglycerol O-acyltransferase	0,20	3,20	+	+	0,5449	0,1957			-0,1927	-0,1481
A0A2Z5Y8V1_MYCMR	Glyco_hydro_3 domain-containing protein	0,91	5,07	+	+	0,5395	0,9103	+	+	0,7187	1,1250
A0A2Z5Y958_MYCMR	Carbon monoxide dehydrogenase subunit G (CoxG)	0,63	3,61	+	+	0,5526	0,6324	+	+	0,3139	0,6347
A0A2Z5Y975_MYCMR	Adenylosuccinate synthetase	0,42	5,47	+	+	0,4395	0,4211		+	-0,3118	-0,1382
A0A2Z5Y992_MYCMR	Biotin carboxylase	0,38	8,92	+	+	0,5990	0,3788	+	+	0,3594	0,3404
A0A2Z5Y9B4_MYCMR	Acetate kinase	0,47	6,47	+	+	0,6152	0,4737		+	-0,1195	0,0996
A0A2Z5Y9C0_MYCMR	Phosphate acetyltransferase	0,86	3,82	+	+	0,8583	0,8605	+	+	0,5783	0,8818
A0A2Z5Y9F6_MYCMR	Phosphatidylserine decarboxylase proenzyme	1,21	5,04	+	+	0,8693	1,2106	+	+	0,5048	1,0238
A0A2Z5Y9G3_MYCMR	Superoxide dismutase [Cu-Zn]	0,84	3,75	+	+	0,1422	0,8361	+	+	0,2859	0,6139
A0A2Z5Y9H5_MYCMR	ATPase	0,56	16,60	+	+	0,8446	0,5577	+	+	0,5156	0,5344
A0A2Z5Y9I1_MYCMR	NADH dehydrogenase NdhA	0,88	9,73	+	+	0,7975	0,8760	+	+	0,5309	0,7977
A0A2Z5Y9L3_MYCMR	Uncharacterized protein	0,35	4,16	+	+	0,3170	0,3500	+	+	0,1549	0,1971
A0A2Z5Y9L4_MYCMR	Uncharacterized protein	1,32	2,85	+	+	0,7455	1,3213	+	+	0,9425	1,1766
A0A2Z5Y9Q5_MYCMR	Fatty acyl-CoA reductase	1,25	6,13	+	+	1,0710	1,2524	+	+	1,1133	1,2897
A0A2Z5Y9Q7_MYCMR	Universal stress protein	0,42	2,91	+	+	0,3673	0,4159	+	+	0,2495	0,4742
A0A2Z5Y9Q8_MYCMR	F420-dependent glucose-6-phosphate dehydrogenase	0,31	4,50	+	+	0,2704	0,3147	+	+	-0,0377	0,1583
A0A2Z5Y9T8_MYCMR	Dihydrolipoyl dehydrogenase	0,40	4,51	+	+	0,8117	0,4024	+		0,0831	0,4156
A0A2Z5Y9W5_MYCMR	SDR family oxidoreductase	0,49	7,30	+	+	0,6194	0,4855	+	+	0,1838	0,3074
A0A2Z5Y9Y3_MYCMR	Putative cytochrome P450 135B1	0,74	4,87	+	+	0,9107	0,7431	+	+	0,8323	0,7643
A0A2Z5YA43_MYCMR	Uncharacterized protein	0,49	5,39	+	+	0,4737	0,4897	+	+	0,1653	0,3244
A0A2Z5YA54_MYCMR	Methoxy mycolic acid synthase MmaA3	0,35	3,56	+	+	0,1965	0,3522	+	+	0,3659	0,3494
A0A2Z5YA72_MYCMR	Mycolic acid methyltransferase MmaA1	0,36	7,33	+	+	0,7401	0,3639	+	+	0,7979	0,7288
A0A2Z5YA98_MYCMR	Membrane protein	0,62	2,92	+	+	0,2796	0,6160	+	+	0,2123	0,4543
A0A2Z5YAF9_MYCMR	30S ribosomal protein S5	0,19	3,34	+	+	0,2023	0,1939	+	+	0,3442	0,3834
A0A2Z5YAJ1_MYCMR	Inosine-5'-monophosphate dehydrogenase	0,41	3,92	+	+	0,2218	0,4096	+	+	0,1014	0,1992
A0A2Z5YAJ4_MYCMR	Protein kinase domain-containing protein	0,63	5,00	+	+	0,4160	0,6327	+	+	0,5991	0,5703
A0A2Z5YAP9_MYCMR	Putative oxidoreductase	1,10	3,84	+	+	1,0249	1,0995	+	+	0,7869	0,9562
A0A2Z5YAQ5_MYCMR	Putative glycosyl hydrolase	0,49	13,05	+	+	0,8653	0,4853	+	+	0,3768	0,5158
A0A2Z5YAT3_MYCMR	N-acyl-L-amino acid amidohydrolase	0,61	8,55	+	+	0,6177	0,6078	+	+	0,2247	0,2284
A0A2Z5YAX6_MYCMR	Succinate dehydrogenase flavoprotein subunit	0,57	5,97	+	+	0,3373	0,5690	+	+	0,3472	0,5246
A0A2Z5YAX8_MYCMR	ABC transporter ATP-binding/permease Rv1747	0,52	3,73	+	+	0,4302	0,5211	+	+	0,4628	0,6219
A0A2Z5YAY9_MYCMR	Malonyl-[acyl-carrier protein] O-methyltransferase	0,98	3,67	+	+	0,8093	0,9822	+	+	0,5657	0,7189
A0A2Z5YB01_MYCMR	Alpha/beta hydrolase	0,59	3,65	+	+	0,1984	0,5945	+	+	0,4709	0,6473
A0A2Z5YB18_MYCMR	Uncharacterized protein	0,30	6,69	+	+	0,6197	0,2970	+	+	0,3633	0,4227
A0A2Z5YBC1_MYCMR	Alcohol dehydrogenase	0,73	12,97	+	+	0,7272	0,7271	+	+	0,5665	0,6847
A0A2Z5YBC6_MYCMR	Uncharacterized protein	0,17	4,90	+	+	0,1734	0,1671	+	+	0,2965	0,2385
A0A2Z5YBF7_MYCMR	Peptide chain release factor 2	0,21	5,62	+	+	0,3935	0,2060	+	+	0,3375	0,2909
A0A2Z5YBG4_MYCMR	Uncharacterized protein	0,12	2,31	+	+	0,1764	0,1221		+	0,0805	0,1313
A0A2Z5YBG8_MYCMR	NADH-quinone oxidoreductase subunit I	1,03	2,27	+	+	0,8471	1,0251	+	+	0,3570	1,0216
A0A2Z5YBI1_MYCMR	Putative L-carnitine dehydratase	0,54	2,49	+	+	0,2238	0,5384	+	+	0,2077	0,4662
A0A2Z5YBQ0_MYCMR	NADH-quinone oxidoreductase subunit F	0,17	6,94	+	+	0,5681	0,1691			0,0000	-0,0749
A0A2Z5YBR8_MYCMR	Choline-sulfatase	0,12	3,82	+	+	0,2009	0,1184	+	+	0,2803	0,3912
A0A2Z5YBS2_MYCMR	4-hydroxyacetophenone monooxygenase	0,82	3,07	+	+	0,2796	0,8248	+	+	0,5864	0,7390
A0A2Z5YBS9_MYCMR	NAD-binding protein of Kef-type K+ transporter	0,76	2,27	+	+	0,5817	0,7588	+	+	0,4650	0,5906
A0A2Z5YBU2_MYCMR	Protein translocase subunit SecA	0,24	14,01	+	+	0,1966	0,2382	+	+	0,2168	0,2582
A0A2Z5YBV7_MYCMR	NADPH-ferredoxin reductase FprA	0,32	5,47	+	+	0,6608	0,3249	+	+	0,3288	0,4524
A0A2Z5YBZ0_MYCMR	CoA ester lyase	0,18	2,26	+	+	0,0833	0,1820	+	+	0,1968	0,2780
A0A2Z5YC08_MYCMR	Acyl-CoA dehydrogenase FadE22	0,47	8,06	+	+	0,7225	0,4697	+	+	0,2553	0,2922
A0A2Z5YC38_MYCMR	FeIII-dicitrate-binding periplasmic lipoprotein	0,69	2,76	+	+	0,6279	0,6916	+	+	0,6073	0,7736
A0A2Z5YC69_MYCMR	NADH-quinone oxidoreductase	0,52	5,01	+	+	0,6484	0,5230		+	-0,3495	0,1809
A0A2Z5YCD2_MYCMR	Glutamate--tRNA ligase	0,22	2,26	+	+	0,1241	0,2249	+	+	0,2222	0,2934
A0A2Z5YCF8_MYCMR	Thioredoxin-like_fold domain-containing protein	1,09	12,35	+	+	0,6061	1,0916	+	+	0,5825	0,8770
A0A2Z5YCG9_MYCMR	Formyltetrahydrofolate deformylase	0,55	3,48	+	+	0,5487	0,5532		+	-0,1708	0,2463

A0A2Z5YCQ5_MYCMR	Polyphosphate kinase	0,32	10,90	+	+	0,3929	0,3236	+	+	0,0908	0,1745
A0A2Z5YCR1_MYCMR	Uncharacterized protein	0,50	2,58	+	+	0,4613	0,5021	+	+	0,7503	0,6277
A0A2Z5YCR3_MYCMR	Transcriptional repressor NrdR	1,12	5,38	+	+	0,8684	1,1191	+	+	0,5373	0,9098
A0A2Z5YCV1_MYCMR	Long-chain-fatty-acid--AMP ligase FadD28	0,37	6,80	+	+	0,1537	0,3702	+	+	0,1661	0,2502
A0A2Z5YCW9_MYCMR	Glutamyl-tRNA(Gln) amidotransferase subunit A	0,39	3,94	+	+	0,2835	0,3931	+	+	0,3744	0,5393
A0A2Z5YCX2_MYCMR	Transcription termination/antitermination protein NusA	0,20	6,16	+	+	0,2517	0,2042		+	-0,0160	0,0069
A0A2Z5YCX7_MYCMR	Polyribonucleotide nucleotidyltransferase	0,06	2,53	+	+	0,0999	0,0578	+	+	0,1564	0,1300
A0A2Z5YCY1_MYCMR	NAD(P)H:quinone oxidoreductase type IV	0,50	2,26	+	+	0,4893	0,5041		+	0,3530	0,3553
A0A2Z5YD08_MYCMR	Signal peptidase I	1,03	4,69	+	+	0,4686	1,0262	+	+	0,4671	0,8010
A0A2Z5YD15_MYCMR	FAD-binding FR-type domain-containing protein	0,28	2,94	+	+	0,7051	0,2756	+	+	0,4572	0,3488
A0A2Z5YD19_MYCMR	Uncharacterized protein	0,39	3,90	+	+	0,2319	0,3858	+	+	0,2237	0,3653
A0A2Z5YD54_MYCMR	Uncharacterized protein	0,94	8,02	+	+	0,7173	0,9357	+	+	0,7733	0,8167
A0A2Z5YD60_MYCMR	Histidine--tRNA ligase	0,46	3,48	+	+	0,3275	0,4572	+	+	0,3629	0,4738
A0A2Z5YD64_MYCMR	Chromosome partition protein Smc	0,76	3,08	+	+	0,5255	0,7574	+	+	0,4131	0,6387
A0A2Z5YD85_MYCMR	Ribonuclease D	0,83	9,56	+	+	0,4649	0,8345	+	+	0,3351	0,6659
A0A2Z5YD88_MYCMR	Inositol-1-monophosphatase	0,34	6,02	+	+	0,4127	0,3392	+	+	0,2948	0,4112
A0A2Z5YD99_MYCMR	Ubiquinone biosynthesis O-methyltransferase	0,84	3,00	+	+	0,5016	0,8432	+	+	0,3253	0,6350
A0A2Z5YDE4_MYCMR	Threonine--tRNA ligase	0,41	2,85	+	+	0,2406	0,4057	+	+	0,2728	0,3828
A0A2Z5YDG9_MYCMR	35 kDa protein	0,32	10,53	+	+	0,2203	0,3225	+	+	0,1329	0,2128
A0A2Z5YDH4_MYCMR	Spermidine/putrescine ABC transporter ATP-binding protein	0,49	3,60	+	+	0,3025	0,4933	+	+	0,4524	0,6007
A0A2Z5YDI2_MYCMR	Elongation factor P	0,52	14,67	+	+	0,9014	0,5152	+	+	0,8172	0,6122
A0A2Z5YDK2_MYCMR	Sugar ABC transporter ATP-binding protein	0,83	14,21	+	+	0,5538	0,8282	+	+	0,1365	0,3617
A0A2Z5YDL0_MYCMR	Multifunctional fusion protein	1,15	5,69	+	+	0,3602	1,1473	+	+	0,6537	1,1569
A0A2Z5YDM6_MYCMR	Uncharacterized protein	0,38	9,13	+	+	0,7897	0,3825		+	0,0000	0,0764
A0A2Z5YDN9_MYCMR	Membrane protein	0,76	4,87	+	+	0,5278	0,7565	+	+	0,7195	0,8004
A0A2Z5YDR4_MYCMR	Preprotein translocase subunit YajC	1,08	5,69	+	+	0,9131	1,0815	+	+	0,8452	0,7875
A0A2Z5YDS2_MYCMR	Putative methyltransferase	0,93	11,24	+	+	0,5814	0,9268	+	+	0,7002	0,8883
A0A2Z5YDV1_MYCMR	Acyl-CoA synthetase	0,92	7,98	+	+	0,6864	0,9224	+	+	0,7870	0,8989
A0A2Z5YDV8_MYCMR	DUF58 domain-containing protein	0,67	3,21	+	+	0,5398	0,6720	+	+	0,4476	0,6130
A0A2Z5YDW8_MYCMR	Glucose-6-phosphate 1-dehydrogenase	0,14	9,55	+	+	0,3847	0,1398	+	+	0,0583	0,0842
A0A2Z5YDX9_MYCMR	Methionyl-tRNA formyltransferase	0,24	3,42	+	+	0,3896	0,2402	+	+	0,3602	0,3493
A0A2Z5YDY0_MYCMR	Oxidoreductase	0,31	5,12	+	+	0,4627	0,3133		+	0,0215	0,1630
A0A2Z5YDZ3_MYCMR	Uncharacterized protein	1,05	4,41	+	+	0,6465	1,0482	+	+	0,6550	0,9981
A0A2Z5YE17_MYCMR	Transketolase	0,18	3,70	+	+	0,1702	0,1763	+	+	-0,1911	0,1757
A0A2Z5YE48_MYCMR	3-dehydroquinate synthase	0,88	3,10	+	+	0,6689	0,8797	+	+	0,7272	0,8686
A0A2Z5YE53_MYCMR	X-Pro dipeptidase	0,12	8,56	+	+	0,3453	0,1190			0,1564	-0,2124
A0A2Z5YE68_MYCMR	Modulator of FtsH protease HflK	0,73	6,02	+	+	0,3958	0,7260	+	+	0,4229	0,6331
A0A2Z5YE69_MYCMR	Succinylglutamate desuccinylase	0,07	7,84	+	+	0,3157	0,0670	+	+	0,1542	0,1667
A0A2Z5YEA6_MYCMR	RNA methyltransferase	0,21	4,53	+	+	0,5146	0,2128	+	+	0,3080	0,3536
A0A2Z5YEG5_MYCMR	CTP synthase	0,87	4,57	+	+	0,8226	0,8725	+	+	0,6932	0,7377
A0A2Z5YEJ6_MYCMR	Acetylglutamate kinase	0,96	8,80	+	+	0,7309	0,9642	+	+	0,8792	0,8920
A0A2Z5YEM0_MYCMR	Phosphatase	0,58	6,93	+	+	0,5841	0,5766	+	+	0,4332	0,5271
A0A2Z5YEN6_MYCMR	UPF0353 protein MMRN_23370	0,94	6,31	+	+	0,3360	0,9361	+	+	0,5905	0,9109
A0A2Z5YEQ1_MYCMR	Argininosuccinate synthase	0,78	5,12	+	+	0,3701	0,7824	+	+	0,6074	0,6648
A0A2Z5YEQ3_MYCMR	Uncharacterized protein	1,02	2,77	+	+	0,6938	1,0229	+	+	0,8181	1,1195
A0A2Z5YER2_MYCMR	GTPase Der	0,99	3,22	+	+	0,7541	0,9855	+	+	0,7909	0,8986
A0A2Z5YEU3_MYCMR	Type VII secretion protein EccC	0,84	4,75	+	+	0,2539	0,8418	+	+	0,6505	0,8598
A0A2Z5YEW6_MYCMR	Imidazole glycerol phosphate synthase subunit HisF	0,42	2,30	+	+	0,2136	0,4229		+	0,0521	0,2434
A0A2Z5YF07_MYCMR	Uncharacterized protein	0,57	3,96	+	+	0,6420	0,5742	+	+	0,5110	0,5673
A0A2Z5YF13_MYCMR	Oxidoreductase	0,61	3,92	+	+	0,2237	0,6137	+	+	0,4215	0,6201
A0A2Z5YF30_MYCMR	Vitamin B12 transport ATP-binding protein BacA	1,24	10,77	+	+	0,6691	1,2433	+	+	0,8772	1,1446
A0A2Z5YF42_MYCMR	ESX-5 secretion system ATPase EccB5	0,84	4,38	+	+	0,4300	0,8416	+	+	0,7135	0,8270
A0A2Z5YFB9_MYCMR	TPR_5 domain-containing protein	0,37	2,30	+	+	0,3526	0,3716		+	0,2961	0,1373
A0A2Z5YFC2_MYCMR	VWFA domain-containing protein	1,51	4,51	+	+	1,0333	1,5063	+	+	0,9859	1,2793
A0A2Z5YFK9_MYCMR	Phenyloxazoline synthase MbtB	1,05	14,42	+	+	0,9546	1,0473	+	+	0,4560	0,8168
A0A2Z5YFM0_MYCMR	Uncharacterized protein	0,63	3,93	+	+	0,9757	0,6297	+	+	0,3648	0,3555
A0A2Z5YFU6_MYCMR	Catalase-peroxidase	0,06	8,94	+	+	0,5042	0,0552	+		-0,3599	-0,1449

A0A2Z5YG87_MYCMR	Apolipoprotein N-acyltransferase	1,03	4,71	+	+	0,6880	1,0273	+	+	0,6860	0,7667
A0A2Z5YG91_MYCMR	Protein PafB	0,65	5,67	+	+	0,7978	0,6479	+	+	0,7051	0,7693
A0A2Z5YGC6_MYCMR	PPOX class F420-dependent oxidoreductase	0,48	2,40	+	+	0,3282	0,4850	+	+	0,4658	0,5426
A0A2Z5YGD3_MYCMR	Putative helicase HelY	0,43	4,41	+	+	0,4864	0,4266	+	+	0,3956	0,3764
A0A2Z5YGI0_MYCMR	Oxidoreductase	0,42	12,72	+	+	0,4913	0,4211	+	+	0,1947	0,3945
A0A2Z5YGI2_MYCMR	ATP phosphoribosyltransferase	0,41	8,68	+	+	0,4185	0,4146	+	+	0,1953	0,2748
A0A2Z5YGJ0_MYCMR	Sec-independent protein translocase protein TatA	0,43	7,21	+	+	0,2967	0,4347	+	+	0,4187	0,3825
A0A2Z5YGJ3_MYCMR	Pup--protein ligase	0,63	6,88	+	+	0,3518	0,6319	+	+	0,4792	0,5318
A0A2Z5YGL5_MYCMR	Phosphoribosyl-ATP pyrophosphatase	0,35	8,70	+	+	0,7621	0,3534	+	+	0,6941	0,7316
A0A2Z5YGN9_MYCMR	Alpha-L-fucosidase	0,34	5,38	+	+	0,6742	0,3433		+	-0,2474	0,1006
A0A2Z5YGP1_MYCMR	Dihydroorotate dehydrogenase (quinone)	0,63	3,17	+	+	0,4130	0,6290	+	+	0,5067	0,7981
A0A2Z5YGQ5_MYCMR	UDP-N-acetylmuramoyl-L-alanyl-D-glutamate--2,6-diaminopimelate ligase	0,14	2,26	+	+	0,1525	0,1376	+	+	0,3025	0,2237
A0A2Z5YH06_MYCMR	Putative asparagine synthetase [glutamine-hydrolyzing]	0,44	4,54	+	+	0,3836	0,4424	+	+	0,4096	0,5390
A0A2Z5YH34_MYCMR	Uncharacterized protein	0,82	3,88	+	+	0,4427	0,8220	+	+	0,2506	0,7532
A0A2Z5YH42_MYCMR	Proteasome subunit alpha	0,72	15,86	+	+	0,8837	0,7156	+	+	0,2242	0,4084
A0A2Z5YH82_MYCMR	Lactamase_B domain-containing protein	0,91	2,48	+	+	0,5548	0,9131	+	+	0,3826	0,8246
A0A2Z5YH85_MYCMR	zf-RING_7 domain-containing protein	0,21	8,76	+	+	0,4234	0,2134	+		-0,0229	0,1660
A0A2Z5YH96_MYCMR	Bifunctional glutamine synthetase adenylyltransferase	0,54	3,43	+	+	0,1908	0,5430	+	+	0,2961	0,5680
A0A2Z5YH98_MYCMR	AAA ATPase forming ring-shaped complexes	0,16	2,77	+	+	0,1077	0,1610		+	0,0966	0,1065
A0A2Z5YHF6_MYCMR	Glutamine synthetase	0,10	3,25	+	+	0,1506	0,0994	+	+	0,0337	0,1329
A0A2Z5YHG4_MYCMR	Putative cystathionine beta-lyase	0,36	5,66	+	+	0,5833	0,3611	+	+	0,1145	0,1878
A0A2Z5YHI6_MYCMR	Diguanylate cyclase	0,58	3,25	+	+	0,2952	0,5757	+	+	0,2277	0,4996
A0A2Z5YHL1_MYCMR	Long-chain-fatty-acid--CoA ligase	0,68	12,35	+	+	0,2742	0,6759	+	+	0,3098	0,6209
A0A2Z5YHP4_MYCMR	Putative oxidoreductase	0,72	7,36	+	+	0,4232	0,7151	+	+	0,3061	0,5321
A0A2Z5YHP6_MYCMR	Probable cytosol aminopeptidase	0,54	14,87	+	+	0,4122	0,5368	+	+	0,2374	0,3900
A0A2Z5YHS5_MYCMR	NADH oxidoreductase	0,66	6,60	+	+	0,8703	0,6574	+	+	-0,1634	0,6160
A0A2Z5YHV0_MYCMR	Uncharacterized protein	0,28	3,67	+	+	0,5046	0,2804			0,0138	0,0071
A0A2Z5YHZ5_MYCMR	Serine O-acetyltransferase	1,20	7,57	+	+	0,8021	1,2041	+	+	0,7912	1,1190
A0A2Z5YI10_MYCMR	Chaperone protein HtpG	0,13	2,17	+	+	0,0330	0,1309	+	+	0,1648	0,2222
A0A2Z5YIB4_MYCMR	Diacylglycerol kinase	0,25	8,24	+	+	0,4508	0,2476	+	+	0,2657	0,4304
A0A2Z5YIE3_MYCMR	Glutamate 5-kinase	0,51	2,76	+	+	0,3146	0,5137	+	+	0,3284	0,4250
A0A2Z5YIG1_MYCMR	Uncharacterized protein	0,37	3,46	+	+	0,5795	0,3721	+	+	0,2446	0,4339
A0A2Z5YII5_MYCMR	GTPase Era	0,58	7,95	+	+	0,6885	0,5770	+	+	0,3888	0,4330
A0A2Z5YIL4_MYCMR	Enoyl reductase	0,67	2,22	+	+	0,4290	0,6728	+	+	0,5099	0,6416
A0A2Z5YIR9_MYCMR	AAA domain-containing protein	0,62	2,98	+	+	0,5515	0,6201	+	+	0,4145	0,5186
A0A2Z5YIW6_MYCMR	Acyl-CoA dehydrogenase	0,27	4,59	+	+	0,2724	0,2742	+	+	-0,0228	0,1409
A0A2Z5YJ17_MYCMR	Glycerol-3-phosphate acyltransferase	1,38	11,25	+	+	1,1541	1,3778	+	+	0,9054	1,2549
A0A2Z5YJ80_MYCMR	Acyl-CoA dehydrogenase	0,57	3,25	+	+	0,2670	0,5692	+	+	0,2183	0,4251
A0A2Z5YJF2_MYCMR	Dipeptidase	0,65	8,58	+	+	0,7873	0,6475	+	+	0,6034	0,6438
A0A2Z5YJG4_MYCMR	Arginine--tRNA ligase	0,18	3,16	+	+	0,3791	0,1812	+	+	0,4271	0,5156
A0A2Z5YJH4_MYCMR	X-Pro dipeptidyl-peptidase	0,62	5,92	+	+	0,9878	0,6189	+	+	0,7810	0,7786
A0A2Z5YJL0_MYCMR	ATP synthase gamma chain	1,20	5,74	+	+	0,3531	1,1996	+	+	0,3979	1,0837
A0A2Z5YJM1_MYCMR	Peptide chain release factor 1	0,36	3,09	+	+	0,4069	0,3642	+	+	0,4341	0,3296
A0A2Z5YJN4_MYCMR	ABC transporter ATP-binding protein	0,88	2,58	+	+	0,5524	0,8756	+	+	0,4631	0,7017
A0A2Z5YJQ4_MYCMR	Acyl-CoA dehydrogenase	0,20	5,71	+	+	0,2642	0,2015		+	-0,0562	0,0559
A0A2Z5YJU6_MYCMR	Homoserine dehydrogenase	0,22	2,47	+	+	0,0635	0,2242	+	+	0,2765	0,3013
A0A2Z5YJX3_MYCMR	L-3-phosphoserine phosphatase	0,65	5,35	+	+	0,3240	0,6464	+	+	0,3334	0,5069
A0A2Z5YJX5_MYCMR	ATP synthase subunit alpha	0,92	8,89	+	+	0,1630	0,9214	+	+	0,2051	0,8062
A0A2Z5YK33_MYCMR	ATP-dependent RNA helicase DeaD	0,37	3,42	+	+	0,2555	0,3711	+	+	0,2438	0,2944
A0A2Z5YK43_MYCMR	Magnesium transport protein CorA	1,08	5,51	+	+	0,8144	1,0823	+	+	0,7171	0,9503
A0A2Z5YK62_MYCMR	Multifunctional fusion protein	0,66	4,08	+	+	0,1457	0,6624	+	+	0,4405	0,6737
A0A2Z5YKA2_MYCMR	3-oxoacyl-[acyl-carrier-protein] reductase FabG	0,22	10,03	+	+	0,2094	0,2154	+	+	0,1617	0,2187
A0A2Z5YKI9_MYCMR	Uncharacterized protein	0,78	9,66	+	+	0,5894	0,7817	+	+	0,4717	0,7752
A0A2Z5YKN2_MYCMR	Uncharacterized protein	0,37	6,80	+	+	0,7274	0,3664	+	+	0,1828	0,2571
A0A2Z5YKP0_MYCMR	Ribonuclease PH	0,56	5,06	+	+	0,5546	0,5610	+	+	0,2360	0,4236
A0A2Z5YKW6_MYCMR	Aldehyde-alcohol dehydrogenase	0,91	2,20	+	+	0,4215	0,9109	+	+	0,3985	0,6876
A0A2Z5YL56_MYCMR	Large-conductance mechanosensitive channel	1,03	5,26	+	+	0,2816	1,0293	+	+	0,5621	0,5606

A0A2Z5YL93_MYCMR	Long-chain-acyl-CoA synthetase	0,99	6,98	+	+	0,8871	0,9887	+	+	0,8441	0,9936
A0A2Z5YL96_MYCMR	Uncharacterized protein	0,17	3,98	+	+	0,3980	0,1677			-0,2755	-0,1427
A0A2Z5YLB1_MYCMR	Magnesium chelatase	1,38	7,58	+	+	1,0160	1,3765	+	+	0,8898	1,2134
A0A2Z5YLI1_MYCMR	Thiolase_N domain-containing protein	0,39	3,24	+	+	0,5004	0,3907	+	+	0,7213	0,5995
A0A2Z5YLN8_MYCMR	Bifunctional purine biosynthesis protein PurH	0,39	3,65	+	+	0,1896	0,3922	+	+	0,3208	0,4163
A0A2Z5YLU1_MYCMR	Phosphate-specific transport system accessory protein PhoU	0,52	4,24	+	+	0,6527	0,5223	+	+	0,5043	0,6871
A0A2Z5YM24_MYCMR	6-aminohexanoate-cyclic-dimer hydrolase	0,44	5,29	+	+	0,4121	0,4390		+	-0,1877	0,1458
A0A2Z5YM67_MYCMR	Molybdopterin molybdenumtransferase	0,63	4,95	+	+	0,2303	0,6337	+	+	0,2276	0,5886
A0A2Z5YM88_MYCMR	Histidine kinase	1,33	7,29	+	+	0,7401	1,3334	+	+	0,9315	1,2705
A0A2Z5YM93_MYCMR	Phosphoribosylformylglycinamidine synthase subunit PurQ	0,77	3,33	+	+	0,6125	0,7702	+	+	0,4724	0,6245
A0A2Z5YME0_MYCMR	Acetolactate synthase	0,26	4,20	+	+	0,3289	0,2604	+	+	0,1378	0,1490
A0A2Z5YMI8_MYCMR	DNA-binding protein	0,24	3,15	+	+	0,3483	0,2377			0,0671	0,0697
A0A2Z5YMN4_MYCMR	Arylamine N-acetyltransferase	0,43	2,30	+	+	0,3192	0,4256	+		-0,0543	0,2412
A0A2Z5YMQ6_MYCMR	Cysteine--tRNA ligase	0,71	2,41	+	+	0,3788	0,7058	+	+	0,4296	0,5543
A0A2Z5YMU4_MYCMR	2-amino-4-hydroxy-6-hydroxymethyldihydropteridine diphosphokinase	0,34	4,66	+	+	0,3351	0,3392		+	0,0300	0,1324
A0A2Z5YMV3_MYCMR	ATP-dependent zinc metalloprotease FtsH	0,66	5,39	+	+	0,1731	0,6563	+	+	0,3543	0,5989
A0A2Z5YN08_MYCMR	Uncharacterized protein	0,75	3,94	+	+	0,1688	0,7477	+	+	0,5928	0,7658
A0A2Z5YN34_MYCMR	Crp/Fnr family transcriptional regulator	0,06	3,86	+	+	0,1481	0,0587	+	+	-0,0028	0,0933
A0A2Z5YN61_MYCMR	Fido domain-containing protein	0,42	4,15	+	+	0,4801	0,4174	+	+	0,3859	0,5622
A0A2Z5YN68_MYCMR	Alpha/beta hydrolase	0,63	2,80	+	+	0,3274	0,6330	+	+	0,3403	0,6179
A0A2Z5YN72_MYCMR	Diacylglycerol O-acyltransferase	0,48	2,62	+	+	0,0832	0,4819	+	+	0,3123	0,4169
A0A2Z5YN92_MYCMR	Anti-sigma factor antagonist	0,30	5,61	+	+	0,5334	0,2959		+	-0,0679	0,0860
A0A2Z5YNC0_MYCMR	Uncharacterized protein	0,75	4,60	+	+	0,9222	0,7501	+	+	0,8902	0,9153
A0A2Z5YNE9_MYCMR	Oxidoreductase	0,78	10,79	+	+	0,6447	0,7809	+	+	0,5277	0,6547
A0A2Z5YNG5_MYCMR	Putative acyl-[acyl-carrier-protein] desaturase DesA1	0,42	3,43	+	+	0,1787	0,4232	+	+	0,3723	0,4687
A0A2Z5YNG8_MYCMR	Phosphatase PAP2 family protein	0,58	9,76	+	+	0,3916	0,5848	+	+	0,3586	0,5438
A0A2Z5YNG9_MYCMR	Acid resistance periplasmic serine protease MarP	0,42	2,86	+	+	0,3552	0,4153	+	+	0,2886	0,6052
A0A2Z5YNH3_MYCMR	Uncharacterized protein	0,69	21,54	+	+	1,4512	0,6863	+	+	0,8016	0,4929
A0A2Z5YNJ0_MYCMR	Putative metallophosphoesterase	0,95	6,14	+	+	0,6340	0,9505	+	+	0,3426	0,8572
A0A2Z5YNL3_MYCMR	Putative cysteine desulfurase	0,69	3,87	+	+	0,8696	0,6880	+	+	0,6596	0,7103
A0A2Z5YNM5_MYCMR	Membrane protein	1,28	6,95	+	+	0,6375	1,2808	+	+	0,9272	1,1887
A0A2Z5YNN1_MYCMR	Decaprenylphosphoryl-beta-D-ribose oxidase	0,64	2,89	+	+	0,3746	0,6396	+	+	0,6733	0,7271
A0A2Z5YNP4_MYCMR	Aspartate-semialdehyde dehydrogenase	0,37	3,48	+	+	0,1789	0,3710	+	+	0,3611	0,3843
A0A2Z5YNR3_MYCMR	Type VII secretion protein EccCa	0,99	8,43	+	+	0,8854	0,9878	+	+	0,6310	0,8371
A0A2Z5YNT0_MYCMR	Putative arabinosyltransferase B	1,05	8,81	+	+	0,4388	1,0497	+	+	0,6258	1,0524
A0A2Z5YNT3_MYCMR	Uncharacterized protein	1,12	7,88	+	+	0,8545	1,1225	+	+	0,8147	1,0309
A0A2Z5YNV9_MYCMR	ESX-1 secretion system protein EccA1	0,71	10,68	+	+	1,1242	0,7057	+	+	0,5110	0,4995
A0A2Z5YNW9_MYCMR	FHA domain-containing protein	0,41	5,55	+	+	0,7132	0,4098	+	+	0,4998	0,5547
A0A2Z5YNX0_MYCMR	ESX-1 secretion system protein EccD1	0,76	5,68	+	+	0,8892	0,7570	+	+	0,5778	0,5507
A0A2Z5YNY0_MYCMR	PPE family protein	0,32	19,98	+	+	0,9245	0,3223		+	0,3183	0,0339
A0A2Z5YNY5_MYCMR	CbiA domain-containing protein	0,83	4,34	+	+	0,3220	0,8281	+	+	0,4608	0,7895
A0A2Z5YNZ0_MYCMR	Type VII secretion protein EccE	0,88	8,94	+	+	0,4923	0,8755	+	+	0,5885	0,8044
A0A2Z5YP05_MYCMR	ESX-1 secretion-associated protein EspL	0,49	10,57	+	+	1,1892	0,4860		+	0,4573	0,2699
A0A2Z5YP09_MYCMR	Membrane protein insertase YidC	0,97	8,98	+	+	0,5624	0,9724		+	0,5969	0,4894
A0A2Z5YP10_MYCMR	EspA_EspE domain-containing protein	1,75	10,49	+	+	3,0364	1,7463	+	+	1,2020	0,8906
A0A2Z5YP17_MYCMR	Putative chromosome-partitioning protein ParB	0,26	7,83	+	+	0,4343	0,2610	+	+	0,3935	0,3356
A0A2Z5YP23_MYCMR	Transferase	0,63	3,94	+	+	0,3669	0,6345	+	+	0,5313	0,7074
A0A2Z5YP33_MYCMR	Adenylate/guanylate cyclase domain-containing protein	1,04	8,23	+	+	0,5754	1,0363	+	+	0,6961	0,9773
A0A2Z5YP90_MYCMR	Uncharacterized protein	0,80	2,17	+	+	0,4378	0,7980	+	+	0,6976	1,4707
A0A2Z5YPA6_MYCMR	ESX-1 secretion-associated protein EspF	0,32	20,52	+	+	2,3054	0,3189		+	0,0293	-0,0478
A0A2Z5YPD0_MYCMR	Putative peptidoglycan biosynthesis protein MviN	0,98	8,49	+	+	0,4929	0,9810	+	+	0,7522	0,9935
A0A2Z5YPF2_MYCMR	Helicase ATP-binding domain-containing protein	0,31	5,47	+	+	0,9086	0,3061			0,0772	0,0221
A0A2Z5YPI3_MYCMR	Uncharacterized protein	0,49	2,86	+	+	0,7977	0,4875	+	+	0,2617	0,5021
A0A2Z5YPV4_MYCMR	ESX-1 secretion-associated protein EspG1	0,31	8,88	+	+	0,7059	0,3072	+	+	0,2112	0,1592
A0A2Z5YPZ4_MYCMR	CbiA domain-containing protein	0,85	7,87	+	+	0,4483	0,8483	+	+	0,5583	0,7686
B2HD25_MYCMM	Cholesterol oxidase ChoD	0,87	3,92	+	+	0,3000	0,8677	+	+	0,0247	0,6721
B2HD34_MYCMM	Oxidoreductase	0,41	3,05	+	+	0,3331	0,4050		+	0,0000	0,0486

B2HD57_MYCMM	L-lactate dehydrogenase (Cytochrome) LldD2	0,68	19,98	+	+	0,6470	0,6803	+	+	0,1246	0,5455
B2HD68_MYCMM	Glutamine synthetase GlnA3	0,52	4,65	+	+	0,5185	0,5199	+	+	0,3793	0,4052
B2HDD1_MYCMM	Lipase LipD	0,85	4,48	+	+	0,5183	0,8478	+	+	0,2565	0,6722
B2HDH9_MYCMM	Histidine kinase	0,99	4,56	+	+	0,9092	0,9927	+	+	0,5683	0,8766
B2HDI9_MYCMM	Transcriptional regulatory protein (Probably TetR-family)	0,55	10,67	+	+	0,7641	0,5457	+	+	0,2583	0,2789
B2HDJ1_MYCMM	Ribose-phosphate pyrophosphokinase	0,36	3,88	+	+	0,2558	0,3574	+	+	-0,1640	0,1389
B2HDS7_MYCMM	O-acetylhomoserine sulfhydrylase MetC	0,07	6,96	+	+	0,4190	0,0698		+	0,1105	0,0683
B2HDT0_MYCMM	Tryptophan--tRNA ligase	0,64	4,10	+	+	0,5536	0,6409	+	+	0,6570	0,8208
B2HDU2_MYCMM	Succinate dehydrogenase (Iron-sulphur protein subunit) SdhB	0,44	3,55	+	+	0,5006	0,4371	+	+	0,2047	0,4598
B2HDV7_MYCMM	Conserved protein	0,27	5,24	+	+	0,2058	0,2742	+	+	0,1777	0,1489
B2HDW7_MYCMM	Glycerol-3-phosphate dehydrogenase	0,82	3,75	+	+	0,3633	0,8179	+	+	0,6604	0,8817
B2HE00_MYCMM	Arylsulfatase AtsD_2	0,22	2,70	+	+	0,3717	0,2225			0,0272	0,1294
B2HEF7_MYCMM	Non-specific serine/threonine protein kinase	0,53	5,48	+	+	0,5249	0,5270	+	+	-0,1524	0,3702
B2HEM7_MYCMM	dTDP-rha:A-D-Glcnac-diphosphoryl polyprenol A-3-L-rhamnosyl transferase, WbbL1	0,78	5,66	+	+	0,4142	0,7831	+	+	0,6956	0,8001
B2HEM8_MYCMM	D-alpha-D-mannose-1-phosphate guanylyltransferase ManB	0,93	4,84	+	+	0,8402	0,9308	+	+	0,4536	0,6437
B2HEQ0_MYCMM	Two-component sensory transduction transcriptional regulatory protein MtrA	0,21	2,68	+	+	0,0820	0,2088	+	+	0,1351	0,2224
B2HEQ5_MYCMM	Ribosome hibernation promoting factor	0,63	4,37	+	+	0,3326	0,6274	+	+	0,0539	0,3995
B2HEQ9_MYCMM	Conserved protein	0,60	28,96	+	+	0,8697	0,5960	+	+	0,4426	0,5284
B2HET5_MYCMM	Iron-regulated short-chain dehydrogenase/reductase	0,29	10,97	+	+	0,3231	0,2907	+	+	-0,0985	0,1177
B2HEW2_MYCMM	Non-specific serine/threonine protein kinase	1,22	8,07	+	+	0,9364	1,2231	+	+	1,0997	1,2248
B2HF74_MYCMM	Two-component response transcriptional regulatory protein PrrA	0,45	12,54	+	+	0,6261	0,4474	+	+	0,4868	0,4345
B2HF81_MYCMM	Citrate synthase	0,08	2,89	+	+	0,0928	0,0800	+	+	-0,1996	0,0796
B2HFB0_MYCMM	Uncharacterized protein	0,75	3,10	+	+	0,7618	0,7479	+	+	0,9227	1,1401
B2HGF1_MYCMM	Two-component system response regulator	0,48	5,96	+	+	0,6612	0,4811	+	+	0,6722	0,7700
B2HGJ5_MYCMM	Cell division ATP-binding protein FtsE	0,72	9,03	+	+	0,4251	0,7202	+	+	0,4088	0,7026
B2HGQ3_MYCMM	Phenyloxazoline synthase MbtB	1,65	4,67	+	+	0,8722	1,6537	+	+	0,8700	1,4124
B2HGV1_MYCMM	Uncharacterized protein	0,92	4,49	+	+	0,2653	0,9243	+	+	0,4917	0,8245
B2HGV2_MYCMM	Conserved protein	0,10	6,32	+	+	0,3487	0,1049	+	+	0,0673	0,2292
B2HGW4_MYCMM	Cytochrome bc1 complex cytochrome b subunit	0,73	3,66	+	+	0,1192	0,7330	+	+	0,3562	0,6424
B2HGX0_MYCMM	Carbohydrate kinase CbhK	0,26	6,61	+	+	0,9907	0,2598			0,3642	0,2355
B2HGY4_MYCMM	Dihydrolipoamide acetyltransferase component of pyruvate dehydrogenase	0,54	3,51	+	+	0,1347	0,5449	+	+	0,2715	0,4231
B2HH94_MYCMM	Uncharacterized protein	0,58	15,05	+	+	0,6425	0,5752	+	+	0,1284	0,5520
B2HH95_MYCMM	Conserved oxidoreductase	0,41	10,03	+	+	0,7207	0,4089	+	+	0,4135	0,4001
B2HHC6_MYCMM	Aldehyde dehydrogenase	0,91	5,20	+	+	0,7474	0,9125	+	+	-0,0500	0,7746
B2HHG9_MYCMM	Cytochrome c oxidase subunit 1	0,84	3,32	+	+	0,2506	0,8429	+	+	0,6351	1,0503
B2HHH8_MYCMM	Conserved ATP-binding protein ABC transporter	0,92	8,88	+	+	0,4975	0,9231	+	+	0,5456	0,8849
B2HHI6_MYCMM	Transferase	1,07	2,18	+	+	0,3034	1,0738	+	+	0,7584	1,0551
B2HHJ2_MYCMM	ATP-binding protein ABC transporter	0,66	4,11	+	+	0,3944	0,6570	+	+	0,3280	0,5722
B2HHJ7_MYCMM	Phenyloxazoline synthase MbtB	0,86	3,42	+	+	0,6090	0,8590	+	+	0,0253	0,6369
B2HHK6_MYCMM	Multimeric flavodoxin WrbA	0,08	3,69	+	+	0,1842	0,0808			-0,0922	0,0003
B2HHM5_MYCMM	Exported protease	0,75	2,57	+	+	0,4944	0,7479	+	+	0,4186	0,7540
B2HHN2_MYCMM	RNase H type-1 domain-containing protein	0,25	3,12	+	+	0,2580	0,2541	+	+	0,2750	0,2898
B2HHU6_MYCMM	Two-component system response phosphate regulon regulator, PhoP	0,39	3,94	+	+	0,3828	0,3860	+	+	0,2965	0,2930
B2HHZ6_MYCMM	Acyl-CoA dehydrogenase FadE26	1,28	2,16	+	+	0,9975	1,2814	+		1,2906	0,9429
DNAA_MYCMM	Chromosomal replication initiator protein DnaA	0,92	3,99	+	+	0,3494	0,9230	+	+	0,4781	0,8738
B2HI60_MYCMM	Anthranilate synthase component II TrpG	0,75	15,21	+	+	0,5173	0,7465	+	+	-0,1772	0,6016
B2HID2_MYCMM	Uncharacterized protein	0,67	10,66	+	+	0,6330	0,6686	+	+	-0,1566	0,3100
B2HIG6_MYCMM	Acetolactate synthase small subunit	0,09	3,31	+	+	0,2007	0,0946	+	+	0,3585	0,2733
B2HII3_MYCMM	DNA-binding protein HU homolog	0,47	30,15	+	+	0,3749	0,4668	+	+	0,3164	0,4392
B2HIN0_MYCMM	Phenolpthiocerol synthesis type-I polyketide synthase PpsB	0,53	2,56	+	+	0,1689	0,5342	+	+	0,3660	0,4080
B2HIN1_MYCMM	Phenolpthiocerol synthesis type-I polyketide synthase PpsA	0,37	3,85	+	+	0,2823	0,3722	+	+	0,3877	0,3777
B2HIQ7_MYCMM	Conserved hypothetical carboxylase	0,49	2,96	+	+	0,4807	0,4888		+	0,1585	0,1505
B2HIY4_MYCMM	Electron transfer protein FdxB	0,79	2,43	+	+	0,3107	0,7950	+	+	0,4973	0,6348
B2HJQ1_MYCMM	4-hydroxy-3-methylbut-2-en-1-yl diphosphate synthase (flavodoxin)	0,25	6,47	+	+	0,3830	0,2484		+	0,1499	0,0934
B2HK57_MYCMM	Phosphoserine phosphatase SerB	1,11	6,01	+	+	0,4120	1,1094	+	+	0,7099	0,9892
B2HK74_MYCMM	Conserved hypothetical Zn-dependent hydrolase	0,66	3,93	+	+	0,4802	0,6597	+	+	-0,0045	0,3033
B2HK77_MYCMM	Anion transporter ATPase	0,54	5,16	+	+	0,3001	0,5352	+	+	0,2832	0,4917

B2HK78_MYCMM	Anion transporter ATPase	1,02	6,80	+	+	0,7851	1,0190	+	+	0,6695	0,9348
B2HKH1_MYCMM	Serine hydroxymethyltransferase	0,39	7,28	+	+	0,3467	0,3860	+	+	0,1147	0,2050
B2HKI0_MYCMM	Glutamine-transport ATP-binding protein ABC transporter	0,93	7,02	+	+	0,6257	0,9263	+	+	0,5149	0,8287
B2HKS8_MYCMM	ABC-type sugar transport protein	0,32	6,98	+	+	0,3153	0,3173	+	+	0,1975	0,3816
B2HKY1_MYCMM	Short-chain type dehydrogenase/reductase	0,33	3,90	+	+	0,3864	0,3307		+	0,0000	0,2020
B2HL85_MYCMM	Cysteine synthase	0,51	4,41	+	+	0,8877	0,5147	+	+	0,3283	0,5126
B2HLC6_MYCMM	Conserved protein	0,49	9,15	+	+	0,7431	0,4865		+	-0,3194	0,0127
B2HLK0_MYCMM	MDMPI_N domain-containing protein	0,83	3,76	+	+	0,6613	0,8318	+	+	0,2864	0,7426
B2HLK8_MYCMM	O-antigen/lipopolysaccharide transport ATP-binding protein ABC transporter RfbE	0,96	4,15	+	+	0,6224	0,9550	+	+	0,8352	1,0692
B2HLL2_MYCMM	Hydrolase_4 domain-containing protein	0,82	4,42	+	+	0,3975	0,8225	+	+	0,5871	0,7315
B2HLR2_MYCMM	Conserved protein	0,32	2,73	+	+	0,5646	0,3196	+	+	0,3142	0,4562
B2HLY3_MYCMM	Conserved hypothetical hydrolase	0,63	2,98	+	+	0,5110	0,6255	+	+	0,5955	0,6518
B2HLZ8_MYCMM	Polyphosphate glucokinase PpgK	0,31	3,15	+	+	0,5427	0,3118	+	+	0,4433	0,4255
B2HM00_MYCMM	Conserved hypothetical secreted protein	1,35	16,39	+	+	0,9211	1,3490	+	+	0,9677	0,8968
B2HM54_MYCMM	Uncharacterized protein	0,90	5,58	+	+	0,6126	0,9026	+	+	0,5150	0,7035
B2HM64_MYCMM	Methyltransferase	0,34	3,27	+	+	0,5823	0,3404	+	+	0,3879	0,3693
B2HMD7_MYCMM	DAP_DH_C domain-containing protein	0,58	6,69	+	+	0,6644	0,5777	+	+	0,1731	0,3753
B2HMG8_MYCMM	Valine--tRNA ligase	0,14	3,54	+	+	0,2951	0,1366	+	+	0,2929	0,3202
B2HMI4_MYCMM	PHB domain-containing protein	0,89	2,51	+	+	0,6764	0,8917	+	+	0,7547	0,8721
B2HMI8_MYCMM	Short-chain type dehydrogenase/reductase	0,61	6,83	+	+	0,6853	0,6083	+	+	0,7595	0,6580
B2HMJ8_MYCMM	Propionyl-CoA carboxylase beta chain 4 AccD4_1	0,33	5,23	+	+	0,0709	0,3283	+	+	0,2091	0,2112
B2HMK4_MYCMM	Conserved transmembrane protein	0,57	2,44	+	+	0,3372	0,5724	+	+	0,5374	0,5730
B2HMK7_MYCMM	Bifunctional UDP-galactofuranosyl transferase GlfT	0,92	8,81	+	+	0,5367	0,9249	+	+	0,6816	0,9176
B2HMT0_MYCMM	Uncharacterized protein	0,31	4,53	+	+	0,7816	0,3149			0,1815	0,0368
B2HMU8_MYCMM	Conserved Mce associated protein	0,87	2,29	+	+	0,3462	0,8663	+	+	0,4287	0,6477
B2HMZ0_MYCMM	Diacylglycerol O-acyltransferase	0,48	3,63	+	+	0,4238	0,4815	+	+	0,1321	0,3589
B2HN27_MYCMM	Enoyl-CoA hydratase 2	0,67	3,27	+	+	0,5465	0,6712	+	+	0,6704	0,7180
B2HN29_MYCMM	Acetyl-CoA acyltransferase FadA2	0,13	3,17	+	+	0,1794	0,1261	+	+	0,2137	0,2733
B2HN35_MYCMM	Succinate dehydrogenase (Iron-sulfur subunit), SdhB_1	0,50	8,37	+	+	0,5851	0,4972	+	+	0,4464	0,4323
B2HN36_MYCMM	Succinate dehydrogenase (Iron-sulfur subunit), SdhA_1	0,35	7,12	+	+	0,3186	0,3517	+	+	0,3414	0,4975
B2HN38_MYCMM	Uncharacterized protein	0,32	12,82	+	+	1,0689	0,3204	+	+	0,0357	0,3193
B2HN47_MYCMM	Acyl-CoA thioesterase II TesB2	0,46	2,23	+	+	0,2712	0,4639	+	+	0,3599	0,4303
B2HN60_MYCMM	Conserved protein	1,23	2,78	+	+	0,5218	1,2319	+	+	0,4767	0,9554
B2HND5_MYCMM	Amino acid decarboxylase	0,88	4,35	+	+	0,8400	0,8785	+	+	0,3927	0,6164
B2HNG4_MYCMM	ATP-dependent Clp protease proteolytic subunit	0,40	6,86	+	+	0,4575	0,3974	+	+	0,2205	0,3234
B2HNJ2_MYCMM	Diacylglycerol O-acyltransferase	0,27	2,30	+	+	0,1802	0,2740	+	+	0,4931	0,2899
B2HNL4_MYCMM	Short-chain type dehydrogenase/reductase	0,28	2,55	+	+	0,1094	0,2796	+	+	0,2350	0,2787
B2HNL7_MYCMM	Oligoribonuclease	0,32	5,30	+	+	0,4942	0,3169	+	+	0,5776	0,5416
B2HNQ3_MYCMM	Conserved hypothetical alanine and proline rich membrane protein	0,77	4,25	+	+	0,6151	0,7681	+	+	0,4488	0,6382
B2HNQ7_MYCMM	Conserved hypothetical alanine and proline rich protein	0,68	9,53	+	+	1,4476	0,6816	+	+	0,3200	0,3784
B2HNT0_MYCMM	Hydrolase	1,33	2,37	+	+	0,2495	1,3255	+	+	0,6648	1,1523
B2HNU4_MYCMM	Aminoglycoside 2'-N-acetyltransferase Aac	0,32	3,56	+	+	0,3406	0,3166		+	0,0000	-0,0722
B2HNV0_MYCMM	Fatty-acid-CoA ligase FadD2	0,83	9,34	+	+	1,3555	0,8301	+	+	0,7625	0,7183
B2HNV2_MYCMM	Acyl-CoA dehydrogenase FadE6	0,25	2,99	+	+	0,1755	0,2496	+	+	0,1550	0,1914
B2HNY7_MYCMM	Conserved membrane protein	0,87	4,90	+	+	0,4805	0,8666	+	+	0,5957	0,6851
B2HP49_MYCMM	Coenzyme A biosynthesis bifunctional protein CoaBC	0,42	7,54	+	+	0,3826	0,4156	+	+	0,2617	0,4043
B2HP95_MYCMM	6-phosphogluconolactonase	0,21	6,71	+	+	0,8262	0,2130		+	0,0854	0,0988
B2HP96_MYCMM	OXPP cycle protein OpcA	0,46	3,62	+	+	0,6404	0,4582	+	+	0,3483	0,3805
B2HPD1_MYCMM	Transcriptional regulatory protein, MoxR1	0,18	2,68	+	+	0,0552	0,1816	+	+	0,1740	0,1180
B2HPL1_MYCMM	Conserved membrane protein	0,85	6,99	+	+	0,6494	0,8546	+	+	0,5448	1,0167
B2HPQ1_MYCMM	Uncharacterized protein	1,09	7,60	+	+	0,6675	1,0945	+	+	0,7622	0,9435
B2HPT1_MYCMM	Conserved secreted protein	0,75	4,19	+	+	0,2842	0,7462	+	+	0,6864	0,9017
B2HPY0_MYCMM	Acyl-CoA dehydrogenase FadE7	0,64	2,38	+	+	0,4468	0,6407	+	+	0,2908	0,3785
B2HQ55_MYCMM	Rhamnosyl transferase WbbL2	0,61	5,02	+	+	0,4887	0,6138	+	+	0,2654	0,4918
B2HQ73_MYCMM	Ketoacyl reductase	0,19	5,65	+	+	0,1754	0,1920	+	+	0,1972	0,3913
B2HQ75_MYCMM	DNA-directed DNA polymerase	1,36	5,42	+	+	0,7302	1,3572	+	+	0,8142	1,2703
B2HQB5_MYCMM	Non-specific serine/threonine protein kinase	0,81	2,85	+	+	0,4727	0,8107	+	+	0,6552	0,8720

B2HQB7_MYCMM	Peroxidoxin BcpB	0,49	3,46	+	+	0,5358	0,4939	+	+	0,1430	0,4082
B2HQF8_MYCMM	Conserved hypothetical metal-dependent hydrolase	0,44	4,19	+	+	0,7289	0,4385			-0,0524	0,0000
B2HQI1_MYCMM	DUF2017 domain-containing protein	0,43	2,54	+	+	0,4630	0,4258	+	+	0,4058	0,5000
B2HQL6_MYCMM	Homoserine kinase	0,39	2,40	+	+	0,5349	0,3911	+	+	0,2785	0,4862
B2HQQ3_MYCMM	Uncharacterized protein	0,13	4,01	+	+	0,2332	0,1312	+	+	0,0516	0,2031
B2HQS0_MYCMM	Aldehyde dehydrogenase, PutA_1	0,44	15,23	+	+	1,1610	0,4413	+	+	-0,1277	0,1913
B2HQS9_MYCMM	Conserved protein	0,74	3,28	+	+	0,4007	0,7420		+	0,4879	0,3446
B2HQU4_MYCMM	Conserved membrane protein	1,05	3,65	+	+	0,7664	1,0516	+	+	0,6598	0,9332
B2HQX5_MYCMM	Anthranilate synthase component 1	0,90	8,45	+	+	0,7726	0,9044	+	+	0,6939	0,8492
B2HR05_MYCMM	Conserved protein	0,32	3,22	+	+	0,6237	0,3190		+	-0,0328	0,0746
B2HR06_MYCMM	Conserved protein	0,48	7,57	+	+	0,9496	0,4806	+	+	0,4509	0,4242
B2HR58_MYCMM	TfuA domain-containing protein	0,43	10,67	+	+	0,5115	0,4271	+	+	-0,0150	0,2398
B2HR59_MYCMM	YcaO domain-containing protein	0,61	3,35	+	+	0,3184	0,6131	+	+	-0,2069	0,3379
B2HRH2_MYCMM	Short-chain type dehydrogenase/reductase	0,45	5,48	+	+	0,2602	0,4495	+	+	0,3293	0,4224
B2HRJ7_MYCMM	Phenyloxazoline synthase MbtB	0,94	8,15	+	+	1,0042	0,9419	+	+	0,1775	0,5941
B2HRN8_MYCMM	Conserved protein	0,35	4,02	+	+	0,3302	0,3478	+	+	0,3705	0,4003
B2HRP5_MYCMM	Bromoperoxidase BpoC	0,57	3,31	+	+	0,5810	0,5732		+	0,1155	0,2693
B2HRQ6_MYCMM	Polyprenyl diphosphate synthetase, GrcC1	0,78	20,49	+	+	0,8588	0,7756	+	+	0,7018	0,8287
B2HRU7_MYCMM	Pseudouridine synthase	1,03	5,43	+	+	0,8673	1,0282	+	+	0,8256	0,9960
B2HS22_MYCMM	Non-specific serine/threonine protein kinase	0,76	12,34	+	+	0,5591	0,7638	+	+	0,4989	0,6856
B2HS42_MYCMM	Iron-sulfur cluster carrier protein	0,47	2,80	+	+	0,1104	0,4686	+	+	0,1660	0,3771
B2HSJ2_MYCMM	Ribonucleotide-transport ATP-binding protein ABC transporter, Mkl	1,12	19,42	+	+	0,9439	1,1219	+	+	0,6809	0,9483
B2HSJ6_MYCMM	Neutral ceramidase	0,38	6,41	+	+	0,2880	0,3760		+	-0,1743	0,1398
B2HSM1_MYCMM	L-lactate dehydrogenase (Cytochrome) LldD1	0,75	2,49	+	+	0,2083	0,7546	+	+	0,4109	0,6704
B2HSR8_MYCMM	Conserved protein	0,86	3,47	+	+	0,4955	0,8606	+	+	0,5325	0,8884
B2HSS0_MYCMM	Oxidoreductase	0,96	11,96	+	+	0,6143	0,9561	+	+	0,3926	0,7996
B2HSS1_MYCMM	Diacylglycerol O-acyltransferase	0,71	10,15	+	+	0,7849	0,7135	+	+	0,2115	0,5017
B2HSX1_MYCMM	Conserved protein	0,90	4,07	+	+	0,3303	0,9032	+	+	0,4991	0,7141
B2HSY5_MYCMM	Inosine-5'-monophosphate dehydrogenase GuaB1	0,32	4,38	+	+	0,4930	0,3185		+	0,1254	0,1159
B2HSY6_MYCMM	6-phosphogluconate dehydrogenase, decarboxylating	0,36	13,50	+	+	0,5429	0,3552	+	+	0,0799	0,1740
B2HT03_MYCMM	Acetyl-CoA acetyltransferase FadA6_3	0,50	11,27	+	+	1,1385	0,4995	+	+	-0,1574	0,3805
B2HT42_MYCMM	Conserved protein	0,50	2,58	+	+	0,6127	0,4987	+	+	0,8502	0,6843
B2HT61_MYCMM	Serine hydroxymethyltransferase	0,20	4,34	+	+	0,5075	0,2036		+	0,1205	0,2149
B2HT71_MYCMM	Mycothiol S-conjugate amidase	0,64	4,17	+	+	0,4963	0,6373	+	+	0,5746	0,7298
B2HT74_MYCMM	Cystathionine gamma-synthase MetB (Cgs)	0,34	10,77	+	+	0,9531	0,3435	+	+	0,0092	0,1685
A0A100HZA7_9MYCO	Biotin carboxylase	-0,18	3,07	+	+	0,1301	-0,1824	+	+	-0,2137	-0,2612
A0A100HZD7_9MYCO	Indole-3-glycerol phosphate synthase	-0,21	1,12		+	0,0516	-0,2060	+	+	-0,0791	-0,1801
A0A100I0J2_9MYCO	Integral membrane indolylacetylinositol arabinosyltransferase	0,98	1,75		+	0,1810	0,9832	+	+	0,0456	0,6264
A0A100I1X1_9MYCO	Metallophosphoesterase	0,43	1,97		+	0,2314	0,4271	+	+	0,3947	0,4158
A0A100I262_9MYCO	tRNA-2-methylthio-N(6)-dimethylallyladenosine synthase	1,01	0,61		+	0,1378	1,0100	+	+	0,2668	0,8408
A0A100I3R0_9MYCO	'Cold-shock' DNA-binding domain protein	-0,61	2,53	+	+	0,0897	-0,6081	+	+	0,1217	-0,2626
A0A100I435_9MYCO	3-oxoacyl-ACP reductase	0,25	0,14		+	0,0119	0,2470		+	0,0979	0,1110
A0A100I666_9MYCO	Lipoprotein, LpqD	-0,17	5,15	+	+	0,2931	-0,1749			-0,0532	-0,1190
A0A100I690_9MYCO	GMP synthase [glutamine-hydrolyzing]	0,13	1,22		+	0,0686	0,1345	+	+	0,2451	0,3354
A0A100I787_9MYCO	Dephospho-CoA kinase	0,51	0,81		+	0,2106	0,5121	+	+	0,2984	0,3979
A0A100I8N3_9MYCO	Alkanesulfonate monooxygenase	0,20	1,60		+	0,1010	0,2040	+	+	-0,0199	0,1277
A0A100I977_9MYCO	Secreted protein	-3,49	0,39		+	0,0424	-3,4853	+	+	-2,7537	-2,7347
A0A100IB09_9MYCO	Immunogenic protein	-0,89	0,56		+	0,2489	-0,8869	+	+	-0,8648	-0,8709
A0A100IB28_9MYCO	Putative glutamine amidotransferase	0,25	0,50		+	0,0564	0,2458		+	-0,0968	-0,0378
A0A100ICM3_9MYCO	6-phosphofructokinase PfkA	0,58	1,71		+	0,4684	0,5764		+	0,6294	0,3563
A0A100IFI0_9MYCO	Metal cation-transporting P-type ATPase F, CtpF	1,04	2,16		+	0,2734	1,0363	+	+	0,3601	0,8961
A0A117DTQ6_9MYCO	Anti-sigma factor antagonist	-0,56	1,25		+	0,1067	-0,5647		+	-0,1919	-0,0682
A0A117DVV7_9MYCO	Secreted protein antigen	-1,28	0,45		+	0,2013	-1,2818	+	+	-1,3614	-1,4754
A0A117DWF4_9MYCO	Membrane protein	0,73	1,15		+	0,1740	0,7284	+	+	0,4852	0,6964
A0A117DX40_9MYCO	Antibiotic biosynthesis monooxygenase	-0,39	3,12	+	+	0,4660	-0,3879			0,0342	0,1115
A0A117DXL2_9MYCO	Type VII secretion protein	1,03	2,02		+	0,4271	1,0339	+	+	0,7692	0,9463
A0A117DYA5_9MYCO	ESAT-6-like protein	-0,28	21,48	+	+	0,7976	-0,2780	+	+	-0,2145	-0,4803

A0A117E045_9MYCO	Bacterial regulatory s, tetR family protein	0,67	0,20		+	0,0407	0,6689	+	+	0,3920	0,5674
A0A124BV66_9MYCO	Cold-shock protein	-0,88	2,18	+	+	0,0923	-0,8849	+	+	-0,2878	-0,7617
A0A2Z5Y7E0_MYCMR	Beta sliding clamp	-0,50	7,51	+	+	0,4393	-0,5024	+	+	-0,6563	-0,6199
A0A2Z5Y7F5_MYCMR	FHA domain-containing protein FhaA	-0,20	0,52		+	0,0454	-0,2048	+	+	0,1748	0,1860
A0A2Z5Y7L5_MYCMR	50S ribosomal protein L9	-1,35	0,97		+	0,0251	-1,3468	+	+	0,2999	-0,4090
A0A2Z5Y7M8_MYCMR	D-Ala-D-Ala carboxypeptidase	-0,50	5,91	+	+	0,2337	-0,5028	+	+	-0,9614	-0,6900
A0A2Z5Y7N5_MYCMR	Enoyl-CoA hydratase 2	1,26	1,40		+	0,3203	1,2637	+	+	0,6875	1,0992
A0A2Z5Y7P2_MYCMR	Uncharacterized protein	0,18	0,53		+	0,0651	0,1818	+	+	0,5388	0,4933
A0A2Z5Y7R8_MYCMR	Putative oxidoreductase	0,54	0,83		+	0,1302	0,5387	+	+	0,0835	0,5224
A0A2Z5Y7V2_MYCMR	Acyl-CoA dehydrogenase	-0,09	1,29		+	0,0988	-0,0897			-0,1075	-0,0111
A0A2Z5Y857_MYCMR	Membrane protein	0,52	0,04		+	0,0072	0,5155	+	+	-0,0214	0,3807
A0A2Z5Y8A0_MYCMR	Epoxide hydrolase EphF	0,39	0,69		+	0,0485	0,3928	+	+	-0,4063	0,1630
A0A2Z5Y8F8_MYCMR	Cyclase	-0,24	0,86		+	0,0632	-0,2361		+	0,0638	-0,0848
A0A2Z5Y8G1_MYCMR	Phosphotyrosine protein phosphatase	0,70	1,72		+	0,3056	0,6968	+	+	0,0870	0,5134
A0A2Z5Y8H0_MYCMR	Mammalian cell entry protein	-0,90	0,54		+	0,0433	-0,8965	+	+	-1,2436	-1,1077
A0A2Z5Y8I6_MYCMR	Mce family protein Mce1C	-1,10	2,57	+	+	0,2153	-1,1037	+	+	-0,7442	-0,9193
A0A2Z5Y8K1_MYCMR	Alcohol dehydrogenase	0,17	0,73		+	0,1351	0,1748			-0,1110	-0,1486
A0A2Z5Y8M9_MYCMR	Phosphoenolpyruvate carboxykinase [GTP]	0,14	0,78		+	0,0355	0,1386	+	+	0,1166	0,1814
A0A2Z5Y8P1_MYCMR	Succinate-semialdehyde dehydrogenase [NADP(+)] 1	-0,25	2,37	+	+	0,1809	-0,2499	+	+	-0,2313	-0,1974
A0A2Z5Y902_MYCMR	SnoaL-like domain-containing protein	0,22	0,63		+	0,1122	0,2205	+	+	0,3917	0,3190
A0A2Z5Y974_MYCMR	Membrane protein	0,31	0,10		+	0,0045	0,3116		+	0,0975	0,1853
A0A2Z5Y981_MYCMR	Glycolipid sulfotransferase	1,08	1,91		+	0,3210	1,0803	+	+	0,7556	1,0512
A0A2Z5Y993_MYCMR	Thiosulfate sulfurtransferase GlpE	0,67	0,80		+	0,1256	0,6722	+	+	0,2626	0,5657
A0A2Z5Y994_MYCMR	Putative aspartate aminotransferase	0,48	0,37		+	0,1225	0,4774	+		0,5626	0,5698
A0A2Z5Y9M6_MYCMR	Phosphoserine phosphatase SerB1	0,18	1,30		+	0,1315	0,1796		+	0,2332	-0,0103
A0A2Z5Y9S8_MYCMR	Putative transport protein MmpL2	0,21	0,60		+	0,1112	0,2104	+	+	-0,2438	0,2222
A0A2Z5Y9V7_MYCMR	Hydrolase	-0,31	0,69		+	0,1070	-0,3114		+	-0,1066	0,1324
A0A2Z5Y9V9_MYCMR	Uncharacterized protein	1,25	1,67		+	0,4220	1,2469	+	+	0,8131	1,1485
A0A2Z5Y9Z7_MYCMR	Uncharacterized protein	0,60	1,96		+	0,1988	0,6043	+	+	0,7170	0,9065
A0A2Z5YA40_MYCMR	3-oxoacyl-[acyl-carrier-protein] synthase 3	0,54	1,59		+	0,4198	0,5447		+	0,3179	0,4284
A0A2Z5YA44_MYCMR	UPF0336 protein MMRN_09930	-0,17	3,10	+	+	0,0778	-0,1681	+	+	-0,0098	-0,0754
A0A2Z5YA53_MYCMR	Glyoxalase II	0,55	1,42		+	0,4662	0,5520	+	+	0,6404	0,7322
A0A2Z5YA77_MYCMR	UPF0234 protein MMRN_09390	-0,51	3,58	+	+	0,0794	-0,5095		+	-0,0857	-0,0194
A0A2Z5YA84_MYCMR	50S ribosomal protein L23	-0,12	1,04		+	0,0291	-0,1161	+	+	0,2344	0,1104
A0A2Z5YAB6_MYCMR	Elongation factor Tu	-0,12	0,98		+	0,0375	-0,1163	+		0,1006	0,1262
A0A2Z5YAD1_MYCMR	50S ribosomal protein L29	-0,46	4,11	+	+	0,1690	-0,4561	+	+	0,0595	-0,1811
A0A2Z5YAG3_MYCMR	Adenylate kinase	-0,40	0,69		+	0,0368	-0,4020	+	+	-0,1173	-0,1805
A0A2Z5YAH2_MYCMR	Ferredoxin reductase	0,28	0,22		+	0,0119	0,2786	+	+	0,0513	0,2972
A0A2Z5YAM6_MYCMR	50S ribosomal protein L6	-0,09	0,74		+	0,0350	-0,0920	+	+	0,2417	0,1193
A0A2Z5YAN8_MYCMR	Elongation factor G	0,16	1,06		+	0,0420	0,1639	+	+	0,1308	0,1202
A0A2Z5YAS4_MYCMR	30S ribosomal protein S11	-0,12	0,85		+	0,0511	-0,1230		+	0,3207	0,0804
A0A2Z5YAX4_MYCMR	dTDP-glucose 4,6-dehydratase	0,62	1,76		+	0,3352	0,6245	+	+	0,5044	0,6222
A0A2Z5YAY3_MYCMR	NAD(P)H-quinone dehydrogenase	0,50	0,16		+	0,0289	0,5029	+	+	0,1096	0,5385
A0A2Z5YAY8_MYCMR	Bifunctional F420 biosynthesis protein FbiB	0,43	0,85		+	0,2086	0,4321	+	+	0,1974	0,2983
A0A2Z5YAZ9_MYCMR	Acid phosphatase	-1,25	1,33		+	0,1471	-1,2519	+	+	-1,2539	-1,3840
A0A2Z5YB07_MYCMR	HAMP domain-containing protein	0,36	1,61		+	0,1407	0,3636	+	+	0,0523	0,3121
A0A2Z5YB67_MYCMR	Adenosylhomocysteinase	-0,34	3,43	+	+	0,1281	-0,3356	+	+	-0,1054	-0,1535
A0A2Z5YBA0_MYCMR	Putative sensor histidine kinase pdtaS	0,24	2,13		+	0,2425	0,2413	+	+	0,0775	0,3370
A0A2Z5YBA5_MYCMR	Uncharacterized protein	-0,65	1,72		+	0,0885	-0,6508	+	+	-0,1670	-0,1479
A0A2Z5YBA6_MYCMR	NADH-quinone oxidoreductase subunit D	-0,31	3,37	+	+	0,4423	-0,3081		+	0,0000	-0,2649
A0A2Z5YBY6_MYCMR	3-isopropylmalate dehydrogenase	0,18	1,01		+	0,1192	0,1846		+	-0,0112	0,0075
A0A2Z5YC02_MYCMR	Formate hydrogenase HycE	0,50	1,07		+	0,2176	0,5046	+	+	0,0000	0,2237
A0A2Z5YC17_MYCMR	Putative oxidoreductase	-0,35	0,11		+	0,0070	-0,3462	+	+	0,1041	-0,1017
A0A2Z5YC19_MYCMR	Nucleoside-diphosphate sugar epimerase	0,44	1,76		+	0,2854	0,4426		+	-0,5276	0,2150
A0A2Z5YC66_MYCMR	tRNA-specific 2-thiouridylase MnmA	0,94	0,19		+	0,0335	0,9442	+	+	0,5580	0,9095
A0A2Z5YC76_MYCMR	Aspartyl/glutamyl-tRNA(Asn/Gln) amidotransferase subunit C	-0,39	0,15		+	0,0199	-0,3894			0,0913	0,1390
A0A2Z5YC98_MYCMR	Elongation factor Ts	-0,08	0,46		+	0,0164	-0,0769	+	+	0,3027	0,2915

A0A2Z5YCC6_MYCMR	Universal stress protein	0,94	1,83		+	0,2601	0,9440	+	+	0,7214	0,8549
A0A2Z5YCE7_MYCMR	D-alanine--D-alanine ligase	0,38	0,59		+	0,1190	0,3815	+	+	0,2313	0,2634
A0A2Z5YCI7_MYCMR	Phthiocerol/phthiodiolone dimycocerosyl transferase	0,60	1,67		+	0,1113	0,6034	+	+	0,2946	0,5942
A0A2Z5YCJ7_MYCMR	Carrier domain-containing protein	0,47	2,11		+	0,2349	0,4717		+	0,5076	0,2242
A0A2Z5YCM5_MYCMR	30S ribosomal protein S16	-0,40	0,42		+	0,0225	-0,3953		+	0,2930	-0,0320
A0A2Z5YCR0_MYCMR	Daunorubicin resistance protein DrrA family ABC transporter ATP-binding protein	0,95	0,23		+	0,0342	0,9470	+	+	0,8958	1,1029
A0A2Z5YCV2_MYCMR	1-deoxy-D-xylulose-5-phosphate synthase	0,35	1,40		+	0,2691	0,3549			0,2167	0,2585
A0A2Z5YCY4_MYCMR	Signal recognition particle receptor FtsY	0,73	2,08		+	0,2082	0,7301	+	+	0,4716	0,6488
A0A2Z5YD26_MYCMR	DLH domain-containing protein	-0,58	1,03		+	0,3336	-0,5752	+	+	-0,3517	-0,7421
A0A2Z5YD42_MYCMR	Multifunctional mycocerosic acid synthase	0,07	1,79		+	0,0584	0,0680	+	+	0,2445	0,0965
A0A2Z5YD49_MYCMR	Carboxylesterase A	0,59	2,09		+	0,3481	0,5855	+	+	0,4328	0,7212
A0A2Z5YD66_MYCMR	LexA repressor	0,46	1,84		+	0,4525	0,4602	+	+	0,4240	0,4517
A0A2Z5YDB9_MYCMR	Ribosome-recycling factor	-0,30	3,82	+	+	0,2783	-0,2982	+		0,0906	0,1178
A0A2Z5YDG4_MYCMR	Pyridoxal 5'-phosphate synthase subunit PdxS	0,35	0,44		+	0,0317	0,3470	+	+	0,2208	0,3398
A0A2Z5YDG5_MYCMR	Transaldolase	-0,11	2,09		+	0,1317	-0,1089	+	+	0,0485	0,0671
A0A2Z5YDK6_MYCMR	Holliday junction ATP-dependent DNA helicase RuvA	-0,31	1,17		+	0,0631	-0,3109	+	+	-0,1982	-0,2223
A0A2Z5YDL7_MYCMR	Aspartate--tRNA(Asp/Asn) ligase	0,16	0,43		+	0,0278	0,1635	+	+	0,1767	0,2839
A0A2Z5YDN3_MYCMR	Uncharacterized protein	0,70	1,89		+	0,4162	0,6992	+	+	0,5860	0,6209
A0A2Z5YDQ8_MYCMR	Lipoarabinomannan carrier protein LprG	-0,09	3,40	+	+	0,0858	-0,0938		+	-0,1836	-0,0612
A0A2Z5YDS6_MYCMR	Thioredoxin	-0,51	1,22		+	0,1972	-0,5148	+	+	0,2979	-0,5677
A0A2Z5YDT6_MYCMR	Protein-export membrane protein SecF	0,90	1,13		+	0,2464	0,8964	+	+	0,5653	0,7876
A0A2Z5YDU0_MYCMR	6,7-dimethyl-8-ribityllumazine synthase	-0,17	1,53		+	0,2319	-0,1725	+		-0,5513	-0,5023
A0A2Z5YDU1_MYCMR	Esterase	0,95	2,05		+	0,4056	0,9507	+	+	0,5873	0,7955
A0A2Z5YE10_MYCMR	Enoyl-[acyl-carrier-protein] reductase [NADH]	0,16	2,01		+	0,0434	0,1556		+	0,1411	0,0797
A0A2Z5YE23_MYCMR	Uncharacterized protein	1,00	1,26		+	0,1402	1,0031	+	+	0,7966	1,0234
A0A2Z5YE39_MYCMR	L-threonine dehydratase	0,47	1,79		+	0,2740	0,4697	+	+	0,4937	0,5454
A0A2Z5YE60_MYCMR	Peptidoglycan endopeptidase RipA	-1,20	0,53		+	0,0861	-1,2048	+	+	-0,8602	-1,6495
A0A2Z5YE61_MYCMR	Phenylalanine--tRNA ligase alpha subunit	0,28	1,18		+	0,0648	0,2753	+	+	0,2004	0,3133
A0A2Z5YEA0_MYCMR	Long-chain-fatty-acid--AMP ligase FadD28	0,60	1,06		+	0,0580	0,5960	+	+	0,3656	0,4466
A0A2Z5YEC0_MYCMR	Cyanophycinase	0,65	1,87		+	0,2261	0,6545	+	+	0,6973	0,8940
A0A2Z5YED0_MYCMR	Tryptophan synthase alpha chain	-0,21	10,60	+	+	0,3144	-0,2063	+	+	-0,0574	-0,0639
A0A2Z5YEH1_MYCMR	Peptidoglycan endopeptidase RipB	-0,51	1,64		+	0,2441	-0,5135	+	+	-0,4532	-0,4557
A0A2Z5YEI8_MYCMR	Triosephosphate isomerase	-0,46	0,36		+	0,0251	-0,4557	+	+	-0,2994	-0,2413
A0A2Z5YEK1_MYCMR	Lysine--tRNA ligase	0,83	2,16		+	0,3108	0,8347	+	+	0,3830	0,5621
A0A2Z5YEK7_MYCMR	Putative transcriptional regulatory protein pdtaR	-0,11	5,59	+	+	0,4050	-0,1108	+	+	0,0862	0,1740
A0A2Z5YEL3_MYCMR	Phenylalanine--tRNA ligase beta subunit	0,45	1,87		+	0,1855	0,4457	+	+	0,3613	0,4774
A0A2Z5YEM3_MYCMR	Tyrosine--tRNA ligase	0,07	0,59		+	0,0251	0,0651	+	+	0,1042	0,0999
A0A2Z5YEZ0_MYCMR	Acyl-CoA thioesterase 2	0,28	0,22		+	0,0370	0,2786			-0,2230	-0,0061
A0A2Z5YF54_MYCMR	UvrABC system protein A	0,50	1,72		+	0,2403	0,4978	+	+	0,4997	0,5199
A0A2Z5YF81_MYCMR	N-acetyl-gamma-glutamyl-phosphate reductase	0,22	1,29		+	0,1224	0,2166		+	0,0797	0,1233
A0A2Z5YFA5_MYCMR	Malate synthase G	-0,17	2,50	+	+	0,1510	-0,1733			0,0231	0,0307
A0A2Z5YFE6_MYCMR	Membrane protein	0,80	0,67		+	0,1260	0,7990	+	+	0,3625	0,5795
A0A2Z5YFH1_MYCMR	O-methyltransferase	0,34	1,79		+	0,2988	0,3417	+	+	0,4470	0,5651
A0A2Z5YFK3_MYCMR	CinA-like protein	0,26	1,27		+	0,2806	0,2640	+	+	0,2508	0,1166
A0A2Z5YFQ2_MYCMR	Isocitrate lyase	-0,10	7,28	+	+	0,3400	-0,0962		+	0,0514	0,0404
A0A2Z5YFR4_MYCMR	LuxR family transcriptional regulator	0,32	0,81		+	0,1974	0,3226	+		0,0166	0,2800
A0A2Z5YFT4_MYCMR	S-adenosyl-L-methionine-dependent methyltransferase	0,49	1,06		+	0,3233	0,4929	+	+	0,6088	0,7288
A0A2Z5YFV7_MYCMR	Peptidase_M28 domain-containing protein	0,83	1,91		+	0,4175	0,8277	+	+	-0,0426	0,6120
A0A2Z5YFW5_MYCMR	Nitrate ABC transporter substrate-binding protein	0,69	1,92		+	0,4337	0,6862	+	+	0,3775	0,5542
A0A2Z5YFZ0_MYCMR	Protein translocase subunit SecA	0,70	2,15		+	0,3176	0,6998	+	+	0,3465	0,6267
A0A2Z5YG04_MYCMR	Glycine dehydrogenase (decarboxylating)	0,81	1,75		+	0,4430	0,8108	+	+	0,6845	0,6681
A0A2Z5YG29_MYCMR	Sulfurtransferase	0,26	0,31		+	0,0690	0,2599		+	0,0000	0,1005
A0A2Z5YGA6_MYCMR	Uncharacterized protein	0,64	0,54		+	0,0929	0,6410		+	-0,1042	0,3738
A0A2Z5YGB4_MYCMR	RNA polymerase-binding protein RbpA	-0,86	0,42		+	0,0239	-0,8566	+	+	-0,1643	-0,3273
A0A2Z5YGD9_MYCMR	Cell division protein SepF	0,11	2,11		+	0,5527	0,1122	+		0,3992	0,3016
A0A2Z5YGU0_MYCMR	NADPH:quinone reductase	0,28	0,73		+	0,0368	0,2798	+	+	0,2249	0,3277
A0A2Z5YH33_MYCMR	Putative dipeptidase PepE	-0,45	0,04		+	0,0060	-0,4465		+	0,0000	0,0000

A0A2Z5YHD7_MYCMR	Pyruvate dehydrogenase E1 component	-0,15	3,63	+	+	0,1143	-0,1487			-0,0246	-0,0036
A0A2Z5YHE9_MYCMR	Diacylglycerol O-acyltransferase	0,94	1,36		+	0,3843	0,9426	+	+	0,4994	0,6135
A0A2Z5YHI0_MYCMR	Enoyl-CoA hydratase	-0,17	0,33		+	0,0371	-0,1738			0,0000	-0,0661
A0A2Z5YHR6_MYCMR	Fatty-acid--CoA ligase	0,60	0,60		+	0,1719	0,5951	+	+	0,4036	0,5859
A0A2Z5YI69_MYCMR	Acetolactate synthase	0,59	1,34		+	0,3770	0,5918	+	+	-0,2563	0,2326
A0A2Z5YI85_MYCMR	Glutathione hydrolase proenzyme	0,51	1,55		+	0,2723	0,5065	+	+	0,5077	0,9257
A0A2Z5YIG9_MYCMR	Ribosomal silencing factor RsfS	0,52	0,58		+	0,1305	0,5228	+	+	0,4211	0,5133
A0A2Z5YIJ5_MYCMR	Heat-inducible transcription repressor HrcA	1,00	2,04		+	0,4945	0,9954	+	+	0,6384	0,9306
A0A2Z5YIV4_MYCMR	DegV domain-containing protein	0,53	1,56		+	0,2447	0,5305	+	+	0,0000	0,2659
A0A2Z5YJ27_MYCMR	Citrate lyase subunit beta-like protein	0,26	1,27		+	0,1389	0,2561		+	-0,0784	0,1666
A0A2Z5YJ90_MYCMR	Iron import ATP-binding/permease protein IrtB	1,29	2,03		+	0,4895	1,2912	+	+	1,1417	1,2517
A0A2Z5YJC5_MYCMR	Endonuclease NucS	0,75	1,01		+	0,1966	0,7497	+	+	0,2419	0,7647
A0A2Z5YJG3_MYCMR	TPM_phosphatase domain-containing protein	0,50	0,99		+	0,1312	0,5031	+	+	0,3239	0,4860
A0A2Z5YJJ4_MYCMR	Thioesterase	-0,17	1,78		+	0,4411	-0,1655	+	+	0,0000	-0,3176
A0A2Z5YJN3_MYCMR	Methyltransf_25 domain-containing protein	0,71	0,06		+	0,0176	0,7096	+	+	0,6377	0,8202
A0A2Z5YJR0_MYCMR	Dihydrofolate synthase	0,31	0,66		+	0,0648	0,3124	+	+	0,1265	0,1995
A0A2Z5YJR6_MYCMR	Malate dehydrogenase	-0,12	10,62	+	+	0,2166	-0,1215	+		-0,0015	-0,0500
A0A2Z5YJS8_MYCMR	ATP synthase epsilon chain	0,98	1,58		+	0,3351	0,9765	+	+	0,5861	1,0623
A0A2Z5YJX1_MYCMR	Oxidoreductase	-0,23	3,41	+	+	0,5736	-0,2335		+	-0,1535	-0,0780
A0A2Z5YJX8_MYCMR	Uncharacterized protein	-0,36	5,36	+	+	0,4223	-0,3594	+	+	-0,6018	-0,5616
A0A2Z5YK19_MYCMR	Putative ABC transporter ATP-binding protein	0,72	1,20		+	0,3058	0,7223	+	+	0,2720	0,6791
A0A2Z5YK61_MYCMR	Epoxide hydrolase	0,62	1,20		+	0,1731	0,6241	+	+	-0,2500	0,2509
A0A2Z5YK73_MYCMR	Transcription termination factor Rho	0,21	0,79		+	0,0125	0,2075	+	+	0,2520	0,2610
A0A2Z5YK93_MYCMR	Acyl-CoA synthetase	0,49	1,58		+	0,1384	0,4925	+	+	0,1685	0,3970
A0A2Z5YKA9_MYCMR	6-phosphogluconate dehydrogenase (Decarboxylating)	-0,23	5,62	+	+	0,6540	-0,2322	+	+	-0,6137	-0,5567
A0A2Z5YKK2_MYCMR	Cystathionine beta-synthase	-0,06	0,15		+	0,0072	-0,0570			-0,0472	-0,0022
A0A2Z5YKN3_MYCMR	Pantothenate kinase	0,57	0,49		+	0,0778	0,5692	+	+	0,2576	0,5218
A0A2Z5YKQ6_MYCMR	Short-chain dehydrogenase	0,84	1,85		+	0,3195	0,8401	+	+	0,5517	0,7731
A0A2Z5YKR3_MYCMR	Molybdenum cofactor biosynthesis protein	-0,52	13,66	+	+	0,7002	-0,5232			-0,4970	-0,2100
A0A2Z5YKT4_MYCMR	Haloacid dehalogenase	-0,40	4,22	+	+	0,3437	-0,3980	+		-0,2170	0,1871
A0A2Z5YKU5_MYCMR	ATP synthase subunit b	0,72	1,98		+	0,1257	0,7162	+	+	0,3710	0,6159
A0A2Z5YKZ7_MYCMR	Dioxygenase	0,53	0,43		+	0,1100	0,5291		+	-0,0839	0,2416
A0A2Z5YL33_MYCMR	Uncharacterized protein	0,40	2,14		+	0,2908	0,4000	+	+	0,0619	0,5013
A0A2Z5YL59_MYCMR	ATP-dependent DNA helicase	1,09	0,59		+	0,0786	1,0875	+	+	0,6412	0,8962
A0A2Z5YLC4_MYCMR	Succinate--CoA ligase [ADP-forming] subunit beta	-0,03	0,61		+	0,0200	-0,0313	+	+	0,0487	0,1708
A0A2Z5YLC5_MYCMR	Uncharacterized protein	-0,33	1,58		+	0,2896	-0,3250	+	+	-0,3062	-0,4294
A0A2Z5YLZ1_MYCMR	TIGR04255 family protein	-0,08	1,22		+	0,0671	-0,0832	+	+	0,0527	0,0651
A0A2Z5YM44_MYCMR	Oligopeptidase B	0,27	0,70		+	0,0373	0,2730		+	-0,1754	0,0744
A0A2Z5YM64_MYCMR	Peroxynitrite isomerase	0,40	1,11		+	0,3083	0,4025	+	+	0,3081	0,4300
A0A2Z5YM80_MYCMR	Long-chain-fatty-acid--CoA/3-oxocholest-4-en-26-o ate--CoA ligase	0,41	2,12		+	0,4021	0,4105	+	+	-0,0048	0,3579
A0A2Z5YMB9_MYCMR	Alpha,alpha-trehalose-phosphate synthase (ADP-forming)	0,23	0,56		+	0,1421	0,2270			0,1361	-0,1792
A0A2Z5YME1_MYCMR	Putative cation-transporting ATPase E	0,59	0,27		+	0,0272	0,5925	+	+	-0,0555	0,2793
A0A2Z5YME3_MYCMR	Phosphoribosylaminoimidazole-succinocarboxamide synthase	-0,18	0,09		+	0,0053	-0,1777	+	+	-0,0673	-0,1290
A0A2Z5YME5_MYCMR	Alpha/beta hydrolase family protein	-0,61	3,35	+	+	0,7617	-0,6062	+	+	-0,4612	-0,6317
A0A2Z5YMP1_MYCMR	Hydroxyacylglutathione hydrolase	-0,15	9,87	+	+	0,7847	-0,1460	+	+	-0,2727	-0,3065
A0A2Z5YN23_MYCMR	DNA polymerase III subunit gamma/tau	0,40	1,84		+	0,1031	0,3981	+	+	0,1172	0,3486
A0A2Z5YN99_MYCMR	Phosphoribosylamine--glycine ligase	0,23	0,80		+	0,0849	0,2279	+	+	0,1506	0,2765
A0A2Z5YNA7_MYCMR	Cytochrome P450	0,58	1,28		+	0,1932	0,5844	+	+	0,1351	0,5324
A0A2Z5YNB0_MYCMR	Probable hercynylcysteine sulfoxide lyase	0,27	0,81		+	0,2445	0,2744			-0,2097	0,0688
A0A2Z5YNC8_MYCMR	Lipid II isoglutaminyl synthase (glutamine-hydrolyzing) subunit GatD	-0,06	0,25		+	0,0133	-0,0567	+	+	-0,2296	-0,2019
A0A2Z5YNJ4_MYCMR	Bacterial proteasome activator	-0,29	1,93		+	0,1392	-0,2860			0,0617	0,0556
A0A2Z5YNJ9_MYCMR	Acyl-CoA dehydrogenase	0,77	1,12		+	0,3287	0,7650	+	+	0,5892	0,6457
A0A2Z5YNK8_MYCMR	Nucleoid-associated protein	-0,87	2,75	+	+	0,0596	-0,8685	+	+	-0,2837	-0,5692
A0A2Z5YNM3_MYCMR	Putative arabinosyltransferase A	1,09	1,58		+	0,2594	1,0853	+	+	0,4871	1,0644
A0A2Z5YNR7_MYCMR	ESX-1 secretion-associated protein EspB	-0,32	11,34	+	+	0,6827	-0,3170	+	+	-0,4441	-0,6071
A0A2Z5YNV0_MYCMR	Thioredoxin	-0,35	0,72		+	0,0537	-0,3485	+		-0,0849	-0,1770
A0A2Z5YNW1_MYCMR	Chromosome partitioning protein ParA	0,69	0,82		+	0,1066	0,6876	+	+	0,4480	0,7422

A0A2Z5YP01_MYCMR	Galactofuranosyl transferase GlfT1	0,63	1,75		+	0,1168	0,6330	+	+	0,4693	0,6151
A0A2Z5YP73_MYCMR	S1 motif domain-containing protein	0,59	0,42		+	0,0687	0,5851	+	+	0,0143	0,3726
A0A2Z5YPB3_MYCMR	GTP cyclohydrolase 1	-0,44	3,10	+	+	0,5406	-0,4412		+	0,3000	-0,1506
A0A2Z5YPB5_MYCMR	ESAT-6-like protein	-0,11	15,75	+	+	1,6058	-0,1116	+	+	-0,1760	-0,6364
A0A2Z5YPB9_MYCMR	Non-specific serine/threonine protein kinase	0,43	0,53		+	0,1203	0,4323	+	+	-0,3326	0,4244
A0A2Z5YPD8_MYCMR	Protein kinase domain-containing protein	0,37	1,54		+	0,2500	0,3715		+	0,1765	0,2155
A0A2Z5YPD9_MYCMR	Ribosomal RNA small subunit methyltransferase G	0,66	0,30		+	0,0718	0,6606		+	-0,0375	0,3183
A0A2Z5YPK8_MYCMR	ESX-1 secretion system protein eccB1	-0,27	0,08		+	0,0064	-0,2657	+	+	-0,4422	-0,3862
A0A2Z5YPY8_MYCMR	R3H domain-containing protein	-0,10	3,93	+	+	0,1661	-0,1043	+		0,2112	0,2098
B2HD63_MYCMM	Conserved protein	0,25	2,14		+	0,3343	0,2471		+	-0,3240	0,1554
B2HD67_MYCMM	CMD domain-containing protein	0,26	0,59		+	0,1738	0,2638		+	0,0000	-0,1155
B2HD88_MYCMM	Cytochrome P450 125A6 Cyp125A6	0,48	0,66		+	0,1398	0,4770	+	+	0,0000	0,3479
B2HD91_MYCMM	Conserved secreted protein	-0,22	5,62	+	+	0,2015	-0,2226	+	+	-0,2710	-0,2565
B2HDC8_MYCMM	Conserved protein	-0,13	4,03	+	+	0,1979	-0,1252		+	0,2325	0,0744
B2HDH8_MYCMM	Two-component transcriptional regulator TrcR	-0,14	0,90		+	0,0722	-0,1402			-0,0496	0,0183
B2HDU4_MYCMM	Succinate dehydrogenase (Hydrophobic membrane anchor subunit) SdhD	0,77	1,05		+	0,2204	0,7746	+	+	0,2167	0,7311
B2HDY4_MYCMM	Transcriptional regulatory protein (Probably AsnC-family)	0,18	0,59		+	0,0570	0,1785			-0,0718	-0,0227
B2HE09_MYCMM	Short-chain type dehydrogenase/reductase	-0,15	2,17	+	+	0,2146	-0,1450		+	0,0000	0,0000
B2HE54_MYCMM	PE family protein	-0,34	5,49	+	+	0,8788	-0,3410			-0,0658	-0,2663
B2HE63_MYCMM	Conserved hypothetical NADP-dependent oxidoreductase	-0,26	1,62		+	0,3205	-0,2624			-0,3467	-0,0516
B2HE90_MYCMM	Ferredoxin reductase	0,88	0,24		+	0,0246	0,8783	+	+	-0,2509	0,6517
B2HEA9_MYCMM	Histidine kinase	0,69	1,35		+	0,1398	0,6939	+	+	0,4451	0,6958
B2HEC0_MYCMM	Uncharacterized protein	0,32	2,10		+	0,1706	0,3211	+	+	0,1116	0,1867
B2HEG3_MYCMM	FMN_red domain-containing protein	0,54	1,83		+	0,4409	0,5442	+	+	0,3912	0,4711
B2HEJ1_MYCMM	Lipid carrier protein or keto acyl-CoA thiolase	0,92	1,08		+	0,2933	0,9235		+	0,6303	0,5125
B2HEK9_MYCMM	Conserved hypothetical membrane protein	0,69	0,40		+	0,0626	0,6852	+	+	0,3627	0,6570
B2HEP5_MYCMM	Transcriptional regulatory protein (Probably TetR-family)	0,70	0,19		+	0,0317	0,6974	+	+	-0,1119	0,4726
B2HES0_MYCMM	Uncharacterized protein	0,24	1,05		+	0,1299	0,2362			-0,2312	0,0508
B2HEU1_MYCMM	DAGKc domain-containing protein	0,26	0,31		+	0,0466	0,2612	+	+	0,0000	0,3392
B2HEV2_MYCMM	Transcriptional regulator, TetR family	0,27	1,61		+	0,2064	0,2716	+	+	0,2207	0,2200
B2HF47_MYCMM	Polyprenol-monophosphomannose synthase, Ppm1B	0,29	1,02		+	0,0470	0,2919	+	+	0,3381	0,3669
B2HF76_MYCMM	Conserved exported or membrane protein	0,58	1,41		+	0,2037	0,5838	+	+	0,5401	0,6292
B2HF96_MYCMM	Conserved protein	0,57	1,70		+	0,3028	0,5729	+	+	0,3877	0,5734
B2HFB9_MYCMM	Acyl-CoA thiolase FadA	0,13	1,84		+	0,1049	0,1288		+	0,2833	0,1087
B2HFC4_MYCMM	Uncharacterized protein	-0,63	3,99	+	+	0,2349	-0,6330	+	+	-0,5090	-0,3302
B2HFS9_MYCMM	Conserved protein	0,55	0,81		+	0,1822	0,5484	+	+	0,3484	0,5646
B2HFU8_MYCMM	Conserved membrane protein	0,69	1,85		+	0,4101	0,6940	+	+	0,1355	0,7063
B2HGI3_MYCMM	Conserved protein	0,15	0,26		+	0,0413	0,1454			-0,2912	-0,0428
B2HGJ3_MYCMM	Conserved transmembrane protein	0,48	1,79		+	0,2321	0,4795	+	+	0,2956	0,4157
B2HGJ6_MYCMM	Cell division protein FtsX	0,67	1,24		+	0,3255	0,6683	+	+	0,6712	0,8141
B2HGP8_MYCMM	Methyltransferase	0,57	0,15		+	0,0339	0,5652	+	+	0,5740	0,5615
B2HGQ1_MYCMM	Conserved transmembrane transport protein MmpL4_5	0,46	0,89		+	0,1375	0,4648	+	+	0,0263	0,4202
B2HGS7_MYCMM	Conserved hypothetical membrane protein	0,45	0,12		+	0,0266	0,4509	+		0,0564	0,3683
B2HGT3_MYCMM	Conserved regulatory protein	-0,30	1,14		+	0,4232	-0,3040		+	-0,0530	-0,1985
B2HGU5_MYCMM	Phospho-2-dehydro-3-deoxyheptonate aldolase	0,26	1,31		+	0,0709	0,2612	+	+	0,2257	0,2511
B2HGU9_MYCMM	1-acylglycerol-3-phosphate O-acyltransferase	1,01	1,43		+	0,3119	1,0075	+	+	0,6538	0,9163
B2HGV0_MYCMM	Uncharacterized protein	0,69	0,65		+	0,1019	0,6940	+	+	0,4742	0,6132
B2HGY3_MYCMM	Short-chain dehydrogenase EphD	0,68	1,48		+	0,2409	0,6812	+	+	0,4327	0,6686
B2HGY5_MYCMM	Uncharacterized protein	0,56	1,07		+	0,2080	0,5644	+	+	-0,1281	0,2456
B2HH00_MYCMM	Deaminase	-0,19	1,79		+	0,1782	-0,1922			-0,0395	0,0000
B2HH29_MYCMM	Phosphoribosylformylglycinamidine cyclo-ligase	-0,06	9,05	+	+	0,2794	-0,0636	+	+	0,0550	0,0905
B2HH30_MYCMM	Amidophosphoribosyltransferase	0,61	0,53		+	0,1726	0,6132	+	+	0,5171	0,5442
B2HH42_MYCMM	29 kDa antigen Cfp29	-0,28	1,53		+	0,2684	-0,2812			0,0000	-0,0786
B2HH90_MYCMM	Conserved hypothetical secreted protein	0,34	0,24		+	0,0421	0,3350			-0,2868	0,0972
B2HH92_MYCMM	Hypothetical alanine and valine rich protein	0,13	0,29		+	0,0211	0,1274	+	+	0,3132	0,3014
B2HHA7_MYCMM	Lysine N-acyltransferase MbtK	0,40	0,08		+	0,0147	0,4050			-0,1171	0,0255
B2HHI1_MYCMM	Methyltransf_11 domain-containing protein	0,75	0,72		+	0,0599	0,7505	+	+	0,7356	0,8056

B2HHK8_MYCMM	Putative 4-hydroxy-4-methyl-2-oxoglutarate aldolase	0,28	0,40		+	0,0379	0,2817	+	+	0,1044	0,3458
B2HHM6_MYCMM	Diacylglycerol O-acyltransferase	0,53	1,40		+	0,3310	0,5267	+	+	0,6809	0,5175
B2HHP6_MYCMM	Uncharacterized protein	-0,49	5,16	+	+	0,3322	-0,4857			0,1722	0,0321
B2HHQ9_MYCMM	Peroxiredoxin AhpE	-0,14	3,21	+	+	0,2538	-0,1448			0,2486	0,0072
B2HHR3_MYCMM	Uncharacterized protein	0,58	0,37		+	0,0307	0,5765	+	+	0,1939	0,4667
B2HHT3_MYCMM	Oxidoreductase	0,06	0,93		+	0,0427	0,0640	+	+	0,2478	0,3615
B2HHW3_MYCMM	Uncharacterized protein	-0,40	2,99	+	+	0,2042	-0,4003		+	-0,1598	-0,0909
B2HI39_MYCMM	Uncharacterized protein	0,26	0,26		+	0,0538	0,2621		+	0,1841	0,0915
B2HI56_MYCMM	Peptidyl-prolyl cis-trans isomerase	-0,52	0,63		+	0,0808	-0,5161	+	+	0,0024	-0,2200
B2HI79_MYCMM	Osmoprotectant transport ATP-binding protein ABC transporter ProV_1	0,65	1,22		+	0,1667	0,6456	+	+	0,1359	0,5250
B2HIB6_MYCMM	Transcriptional regulatory protein	-0,32	2,91	+	+	0,4018	-0,3173			0,0407	-0,0326
B2HIE0_MYCMM	Electron transfer flavoprotein (Beta-subunit) FixA	-0,33	1,56		+	0,0606	-0,3316	+	+	-0,1409	-0,2103
TESA_MYCMM	Thioesterase TesA	0,39	1,40		+	0,3098	0,3873			0,1749	0,3043
B2HJ10_MYCMM	Transcriptional regulatory protein (TetR-family)	0,26	0,40		+	0,0548	0,2596	+	+	0,4040	0,3379
B2HJ24_MYCMM	Conserved transcriptional regulatory protein	-0,38	1,14		+	0,0332	-0,3756	+	+	0,4331	0,1209
B2HJ43_MYCMM	Lysine--tRNA ligase	0,19	1,58		+	0,0382	0,1876	+	+	0,1290	0,1249
B2HJ46_MYCMM	Pantothenate synthetase	0,28	1,75		+	0,2777	0,2757			0,1358	0,2342
B2HJ80_MYCMM	Uncharacterized protein	0,35	1,44		+	0,4333	0,3512	+		-0,0245	0,3451
B2HJE6_MYCMM	Transcriptional regulator	0,85	0,61		+	0,0467	0,8544	+	+	0,0443	0,8251
B2HJJ5_MYCMM	Ribonuclease 3	-0,39	3,45	+	+	0,3348	-0,3925		+	0,1654	0,0301
B2HJL3_MYCMM	RNA-binding protein KhpA	-0,55	4,06	+	+	0,1820	-0,5490	+	+	0,1609	0,1510
B2HJN4_MYCMM	Amidase AmiC	0,91	1,27		+	0,2965	0,9103	+	+	0,3904	0,7552
B2HJT2_MYCMM	Nickel/iron-hydrogenase I large subunit, HyaB	0,42	1,72		+	0,2161	0,4178		+	0,0000	-0,0904
B2HJT4_MYCMM	Nickel cation-binding GTPase, HypB	0,76	0,15		+	0,0330	0,7569		+	0,1494	0,4096
B2HJT8_MYCMM	NADPH-dependent mycothiol reductase Mtr	-0,13	6,28	+	+	0,2882	-0,1333	+	+	-0,2057	-0,1683
B2HJU6_MYCMM	Magnesium chelatase	0,65	1,90		+	0,2914	0,6502	+	+	0,2033	0,5804
B2HJW0_MYCMM	Pribosyltran domain-containing protein	0,40	0,48		+	0,0599	0,3991		+	0,0699	-0,0209
B2HJX0_MYCMM	Uncharacterized protein	0,29	0,79		+	0,1466	0,2863			0,1471	0,1136
B2HJX4_MYCMM	Coenzyme F420-reducing hydrogenase, alpha subunit	-0,31	0,36		+	0,0442	-0,3134			0,0000	0,0000
B2HJZ1_MYCMM	Oxidoreductase	-0,30	0,68		+	0,0779	-0,3042	+	+	-0,4123	-0,5623
B2HK33_MYCMM	Aminotransferase (Adenosylmethionine-8-amino-7-oxononanoate) BioA	0,57	1,69		+	0,5093	0,5658			-0,3107	0,2877
B2HK80_MYCMM	Peptidoglycan glycosyltransferase	0,41	0,47		+	0,0902	0,4061		+	0,0762	0,3125
B2HKB0_MYCMM	Methanol dehydrogenase transcriptional regulatory protein MoxR2	0,78	2,05		+	0,5341	0,7813	+	+	0,5794	0,6836
B2HKB1_MYCMM	Conserved membrane protein	0,50	1,17		+	0,1584	0,4993	+	+	0,3660	0,5023
B2HKE0_MYCMM	Nucleoid-associated protein MMAR_5232	-0,46	3,37	+	+	0,1414	-0,4638	+	+	-0,0882	-0,1465
B2HKH0_MYCMM	L-serine dehydratase	0,24	2,10		+	0,2471	0,2449			-0,2755	0,1772
B2HLB6_MYCMM	Conserved hypothetical membrane protein	0,44	1,71		+	0,2796	0,4412	+	+	0,2476	0,4513
B2HLN1_MYCMM	O-methyltransferase	-0,32	0,41		+	0,0738	-0,3200			0,0084	-0,2089
B2HLN9_MYCMM	Elongation factor G FusA2	-0,22	9,03	+	+	0,5831	-0,2208	+		-0,2891	-0,4774
B2HLS8_MYCMM	Short-chain type dehydrogenase/reductase	-0,28	5,04	+	+	0,2266	-0,2841		+	-0,0277	-0,0491
B2HLU7_MYCMM	NAD(P) transhydrogenase subunit beta	0,89	2,06		+	0,5233	0,8862	+	+	0,6839	0,8106
B2HM10_MYCMM	Conserved integral membrane alanine, valine and leucine rich protein	0,43	0,59		+	0,1503	0,4302	+	+	0,5841	0,6225
B2HM17_MYCMM	Conserved protein	-0,22	4,12	+	+	0,2253	-0,2197		+	0,0606	-0,0620
B2HMC7_MYCMM	Alpha-E domain-containing protein	0,53	1,80		+	0,2240	0,5296	+	+	0,3288	0,5365
B2HMD0_MYCMM	DNA polymerase III, delta subunit HolA	0,76	1,01		+	0,1256	0,7592	+	+	0,1979	0,5940
B2HME2_MYCMM	Uncharacterized protein	0,78	1,16		+	0,2487	0,7804	+	+	0,5351	0,7204
B2HME3_MYCMM	Phosphoglycerate mutase, Gpm_1	0,14	1,64		+	0,0805	0,1368		+	0,1206	0,1383
B2HMN0_MYCMM	Conserved hypothetical transmembrane protein	0,70	0,05		+	0,0150	0,6997	+	+	-0,1503	0,6356
B2HMN2_MYCMM	Superoxide dismutase	-0,36	4,46	+	+	0,3407	-0,3645			-0,5250	-0,3001
B2HMS4_MYCMM	CbiA domain-containing protein	0,58	1,17		+	0,2184	0,5751	+	+	0,5389	0,7409
B2HMU3_MYCMM	MCE-family protein Mce1D	-0,47	2,98	+	+	0,1311	-0,4665	+	+	-0,6965	-0,5626
B2HMU7_MYCMM	Conserved Mce associated transmembrane protein	0,76	1,77		+	0,2750	0,7636	+	+	0,3527	0,6019
B2HMX5_MYCMM	NYN domain-containing protein	0,26	0,50		+	0,0783	0,2561		+	0,0317	0,1243
B2HN37_MYCMM	Succinate dehydrogenase (Membrane anchor subunit)	0,65	1,51		+	0,3283	0,6536			0,2416	0,0801
B2HN76_MYCMM	GTP pyrophosphokinase RelA	0,53	1,38		+	0,3003	0,5280	+	+	0,3493	0,4500
B2HN77_MYCMM	Peptidyl-prolyl cis-trans isomerase	0,90	1,74		+	0,4513	0,8997	+	+	0,3437	0,5157
AROC_MYCMM	Chorismate synthase	0,56	1,80		+	0,2630	0,5589	+	+	0,4753	0,5503

B2HNK7_MYCMM	Acetyl-/propionyl-CoA carboxylase (Beta subunit) AccD1	0,70	0,99		+	0,2597	0,7019	+	+	0,0000	0,6559
B2HNT3_MYCMM	Chromosome partitioning protein ParA	0,40	0,05		+	0,0108	0,4003	+	+	0,2732	0,3867
B2HNY2_MYCMM	SnoaL-like domain-containing protein	-0,36	1,54		+	0,2276	-0,3560			0,0000	0,0000
B2HP43_MYCMM	Carbamoyl-phosphate synthase small chain	0,50	1,64		+	0,2763	0,4965	+	+	0,3893	0,4446
B2HP63_MYCMM	Riboflavin biosynthesis protein RibD	0,28	0,13		+	0,0053	0,2762	+	+	0,1678	0,2808
B2HP84_MYCMM	Glyceraldehyde-3-phosphate dehydrogenase	-0,42	10,29	+	+	0,3134	-0,4227	+	+	-0,0929	-0,3324
B2HPB3_MYCMM	Conserved ATP-binding protein ABC transporter	-0,32	0,19		+	0,0116	-0,3193			0,2397	0,0192
B2HPN1_MYCMM	Transcriptional regulatory protein (FadR family)	0,49	1,09		+	0,1644	0,4926	+	+	0,2398	0,4536
B2HPQ0_MYCMM	Conserved hypothetical alanine-rich protein	0,53	0,50		+	0,0947	0,5336	+	+	0,3534	0,6385
B2HPS8_MYCMM	Flavohemoprotein	0,57	0,47		+	0,0739	0,5686	+	+	0,0203	0,3072
B2HPS9_MYCMM	Chaperone protein ClpB	-0,07	1,51		+	0,0521	-0,0711	+	+	0,2508	0,1868
B2HPV1_MYCMM	Glycosyl hydrolase	0,44	0,23		+	0,0378	0,4405	+	+	-0,2701	0,2226
B2HPV3_MYCMM	Fructose-bisphosphate aldolase	-0,12	4,63	+	+	0,2170	-0,1224	+		-0,0218	-0,0863
B2HQ26_MYCMM	Pyridoxal phosphate-dependent enzyme, WecE	0,60	1,54		+	0,2764	0,5966	+	+	0,0022	0,2891
B2HQ77_MYCMM	Acyltransferase, PlsB1	0,14	0,71		+	0,0248	0,1418	+		0,0257	0,2545
B2HQA0_MYCMM	Nicotinate-nucleotide diphosphorylase (carboxylating)	0,44	0,30		+	0,0617	0,4376			-0,2266	0,0000
B2HQH2_MYCMM	Conserved Mce associated membrane protein	0,23	0,14		+	0,0153	0,2326	+	+	0,1020	0,3310
B2HQI6_MYCMM	Alpha-1,4-glucan:maltose-1-phosphate maltosyltransferase	-0,64	0,70		+	0,0846	-0,6357	+	+	0,0000	-0,7323
B2HQL7_MYCMM	Threonine synthase	-0,21	2,68	+	+	0,2548	-0,2067			0,0176	-0,0830
B2HQU0_MYCMM	Iron-regulated heparin binding hemagglutinin HbhA	-0,18	5,22	+	+	0,3800	-0,1811	+	+	0,4461	0,1943
B2HQZ7_MYCMM	Ribosomal protein S1 RpsA	-0,19	0,95		+	0,0307	-0,1890	+		0,2379	0,1192
B2HR01_MYCMM	UvrABC system protein B	0,63	1,89		+	0,1934	0,6252	+	+	0,3500	0,3532
B2HR04_MYCMM	Iron-regulated conserved protein	-0,59	5,72	+	+	0,1834	-0,5920	+	+	-0,0665	-0,2073
B2HR65_MYCMM	NAD kinase	0,20	2,09		+	0,2837	0,2037	+		-0,0384	0,3105
B2HR80_MYCMM	Diaminopimelate decarboxylase	0,28	1,73		+	0,3652	0,2815	+	+	-0,1741	0,3188
B2HR99_MYCMM	Uncharacterized protein	0,66	0,55		+	0,1056	0,6594	+	+	0,5373	0,6538
B2HRB8_MYCMM	Conserved hydrolase	0,93	0,61		+	0,1752	0,9256	+	+	0,6885	0,8286
B2HRF4_MYCMM	Uncharacterized protein	-0,29	4,80	+	+	0,4299	-0,2908		+	0,2957	-0,0432
B2HRF6_MYCMM	Oxidoreductase	0,81	0,73		+	0,2286	0,8094	+	+	0,5156	0,7264
B2HRY7_MYCMM	Succinate-semialdehyde dehydrogenase [NADP+] dependent (SsdH) GabD2	0,26	0,69		+	0,0381	0,2608	+	+	0,2160	0,3515
B2HS39_MYCMM	Transport transmembrane protein	0,40	1,85		+	0,2724	0,4000		+	0,4376	0,2567
Y4217_MYCMM	Putative O-methyltransferase MMAR_4217	-0,22	6,92	+	+	0,1742	-0,2235		+	-0,0044	-0,0279
B2HS75_MYCMM	AB hydrolase-1 domain-containing protein	0,99	0,48		+	0,0433	0,9917	+	+	0,7159	0,9053
B2HSC6_MYCMM	Conserved protein	0,29	0,97		+	0,2398	0,2941	+	+	0,0410	0,3217
B2HSK9_MYCMM	Transcription regulator, TetR family	0,41	0,07		+	0,0102	0,4141	+	+	0,1255	0,3157
B2HSP2_MYCMM	Uncharacterized protein	-0,23	0,27		+	0,0195	-0,2348	+		-0,0634	-0,0842
B2HSX3_MYCMM	Conserved protein	-0,29	3,83	+	+	0,1926	-0,2870	+	+	-0,2951	-0,2995
B2HSX4_MYCMM	Conserved hypothetical regulatory protein	0,72	0,06		+	0,0256	0,7247		+	0,2784	0,3111
B2HSY3_MYCMM	Uncharacterized protein	0,64	0,66		+	0,1358	0,6405	+	+	0,4393	0,6758
B2HT00_MYCMM	Oxidoreductase	0,86	0,28		+	0,0598	0,8625	+	+	0,0597	0,7239
B2HT21_MYCMM	Transcriptional regulatory protein (Probably TetR-family)	0,13	1,37		+	0,0575	0,1294			-0,0928	0,0148
B2HT53_MYCMM	Fructose-1,6-bisphosphatase	-0,07	0,42		+	0,0256	-0,0736	+	+	0,1581	0,2127
B2HT54_MYCMM	Fumarate hydratase class II	-0,06	1,00		+	0,0527	-0,0611	+	+	0,1317	0,1292
B2HT73_MYCMM	Transcription elongation factor GreA	-0,64	1,05		+	0,0951	-0,6420		+	0,1603	-0,0019
**CH60_MYCMR**	**Chaperonin GroEL2**	-0,44	0,61		+	0,0582	-0,4436		+	0,2986	0,0089
A0A100I003_9MYCO	30S ribosomal protein S13	0,10	-2,39	+	+	-0,1499	0,0961	+	+	0,2122	0,2563
A0A100I0E2_9MYCO	Acyl-ACP desaturase	0,34	-12,41	+	+	-0,7453	0,3361	+	+	0,1763	0,3158
A0A100I1A8_9MYCO	Glycerol-3-phosphate dehydrogenase [NAD(P)+]	0,29	-6,54	+	+	-0,2512	0,2857	+	+	0,3334	0,3238
A0A100I2Z0_9MYCO	50S ribosomal protein L33	0,46	-2,34	+		-0,4296	0,4648	+		-0,6283	-0,7126
A0A100I554_9MYCO	Ribonucleoside-diphosphate reductase	0,18	-6,73	+	+	-1,1792	0,1800		+	0,1130	0,0222
A0A100I907_9MYCO	Uncharacterized protein	0,46	-2,43	+	+	-0,1941	0,4573	+	+	-0,1934	0,4840
A0A100I966_9MYCO	cAMP-binding protein	0,18	3,98	+		0,7925	0,1778			0,1955	0,2220
A0A100I9N6_9MYCO	Linoleoyl-coa desaturase	0,12	-2,41	+		-0,2443	0,1238	+		-0,2579	-0,2851
A0A100IDR1_9MYCO	Nucleoside-diphosphate-sugar epimerase	0,41	2,21	+		0,5937	0,4097	+		0,4965	1,0746
A0A117DWH7_9MYCO	Cell division protein FtsQ	0,11	-6,69	+	+	-0,7596	0,1135	+	+	-0,1512	0,2011
A0A2Z5Y898_MYCMR	CoA transferase	0,30	-2,97	+	+	-0,2379	0,3006		+	0,0319	0,0895
A0A2Z5Y8B3_MYCMR	Maltokinase	0,17	2,45	+		0,5331	0,1652			-0,0914	-0,0196

A0A2Z5Y8C9_MYCMR	Fatty acid desaturase	0,93	-3,93	+	+	-0,9461	0,9285	+	+	0,0257	0,2226
A0A2Z5Y8F9_MYCMR	NAD(P) transhydrogenase subunit alpha	0,07	3,82	+		0,3489	0,0716			0,2646	0,1249
A0A2Z5Y8L1_MYCMR	Putative transport protein MmpL11	1,13	-2,51	+	+	-0,1789	1,1259	+	+	0,5457	0,9602
A0A2Z5Y8S3_MYCMR	3-oxoacyl-ACP reductase	0,04	2,44	+		0,0851	0,0433	+	+	0,2057	0,1019
A0A2Z5Y8U8_MYCMR	Type VII secretion protein EccC	0,53	-4,48	+	+	-0,2296	0,5331	+	+	0,3728	0,5400
A0A2Z5Y948_MYCMR	ESX-3 secretion system protein EccD3	0,09	-9,53	+	+	-0,4377	0,0867	+	+	0,2464	0,3558
A0A2Z5Y9G7_MYCMR	Peptidase	0,13	2,91	+		0,3968	0,1272		+	0,0616	0,1604
A0A2Z5Y9Q3_MYCMR	Ppx-GppA domain-containing protein	0,04	8,44	+		0,3175	0,0426	+	+	0,3299	0,2483
A0A2Z5YA47_MYCMR	Protein translocase subunit SecE	0,36	-2,90	+	+	-0,4443	0,3563		+	0,0635	0,2535
A0A2Z5YA95_MYCMR	DNA-directed RNA polymerase subunit beta'	0,17	-3,09	+	+	-0,0580	0,1694	+	+	0,2100	0,1962
A0A2Z5YAC5_MYCMR	Uncharacterized protein	0,77	-5,64	+	+	-0,6552	0,7704	+	+	0,6078	0,7794
A0A2Z5YAD9_MYCMR	50S ribosomal protein L2	0,04	-5,54	+	+	-0,1237	0,0377	+	+	0,2652	0,2454
A0A2Z5YAG0_MYCMR	Phosphoglucosamine mutase	0,40	-6,97	+	+	-0,6114	0,4034	+	+	0,2512	0,4576
A0A2Z5YAH8_MYCMR	S-adenosyl-L-methionine-dependent methyltransferase	0,36	-2,27	+	+	-0,3995	0,3645	+	+	0,4427	0,4498
A0A2Z5YAR9_MYCMR	30S ribosomal protein S3	0,07	-8,20	+	+	-0,1813	0,0686	+	+	0,2008	0,1010
A0A2Z5YAT1_MYCMR	50S ribosomal protein L14	0,02	-5,45	+		-0,1398	0,0198	+		0,2370	0,1874
A0A2Z5YAW1_MYCMR	Bifunctional NAD(P)H-hydrate repair enzyme	0,18	-2,87	+	+	-0,4477	0,1789	+	+	0,0000	-0,1782
A0A2Z5YB44_MYCMR	ATP-dependent RNA helicase RhlE	0,38	-4,09	+	+	-0,4529	0,3795	+	+	0,5232	0,5143
A0A2Z5YBH5_MYCMR	NADH-quinone oxidoreductase subunit C	0,13	5,26	+		0,8251	0,1254		+	0,0000	0,1423
A0A2Z5YC49_MYCMR	Uncharacterized protein	0,45	-2,43	+	+	-0,1931	0,4479	+	+	0,1395	0,2510
A0A2Z5YC88_MYCMR	Ketol-acid reductoisomerase (NADP(+))	0,10	-8,45	+	+	-0,2985	0,1040	+	+	0,2524	0,1824
A0A2Z5YC91_MYCMR	Long-chain-fatty-acid--AMP ligase FadD29	0,61	-3,91	+	+	-0,6723	0,6056	+	+	0,4795	0,6088
A0A2Z5YCB0_MYCMR	Phthiocerol synthesis polyketide synthase type I PpsC	0,20	-3,31	+	+	-0,2645	0,1988	+	+	0,1714	0,2203
A0A2Z5YCC0_MYCMR	Aspartyl/glutamyl-tRNA(Asn/Gln) amidotransferase subunit B	0,12	-4,76	+	+	-0,1341	0,1179	+	+	0,0455	0,1963
A0A2Z5YCC3_MYCMR	Low molecular weight protein antigen 6	0,29	-3,19	+	+	-0,3870	0,2916		+	0,0011	0,1799
A0A2Z5YCN7_MYCMR	DNA translocase FtsK	1,00	-6,75	+	+	-0,7461	0,9968	+	+	0,9143	1,0845
A0A2Z5YCW3_MYCMR	Phthiocerol/phenolphthiocerol synthesis polyketide synthase type I PpsD	0,18	-7,36	+	+	-0,1338	0,1812	+	+	0,2274	0,2070
A0A2Z5YD02_MYCMR	Alpha/beta hydrolase	0,73	-4,94	+	+	-0,6173	0,7274	+	+	0,5873	0,8085
A0A2Z5YD89_MYCMR	Universal stress protein	0,13	-3,59	+	+	-0,2470	0,1257		+	-0,1192	0,0441
A0A2Z5YDB8_MYCMR	Protein RecA	0,40	-8,12	+	+	-0,5941	0,3986	+	+	0,3779	0,4358
A0A2Z5YDD9_MYCMR	Uncharacterized protein	1,11	-2,84	+	+	-0,4051	1,1072	+	+	0,2776	0,9692
A0A2Z5YDG6_MYCMR	AMP-dependent synthetase	0,38	-2,55	+	+	-0,3436	0,3806		+	-0,0179	0,1145
A0A2Z5YDL8_MYCMR	Carboxylic acid reductase	0,28	-2,69	+		-0,8417	0,2845			0,5991	0,4553
A0A2Z5YDP5_MYCMR	Polyketide synthase	0,37	-9,57	+	+	-0,2790	0,3717	+	+	0,2137	0,3635
A0A2Z5YE46_MYCMR	Acetolactate synthase	0,36	-3,86	+	+	-0,5648	0,3641	+	+	0,2248	0,3665
A0A2Z5YE47_MYCMR	Aconitate hydratase A	0,03	4,15	+		0,3008	0,0315	+		-0,2700	-0,1043
A0A2Z5YE49_MYCMR	8-amino-7-oxononanoate synthase 1	0,52	-3,41	+	+	-0,3170	0,5188	+	+	0,0489	0,5264
A0A2Z5YEA3_MYCMR	Putative glycosyltransferase YkcC	0,25	-6,36	+	+	-0,7681	0,2550	+	+	0,4779	0,5202
A0A2Z5YEA7_MYCMR	Nucleotide-binding protein DAVIS_04514	0,22	2,84	+		0,5177	0,2210		+	0,3232	0,2632
A0A2Z5YED3_MYCMR	Cyanophycin synthetase	0,44	-2,61	+	+	-0,1443	0,4369	+	+	0,2988	0,4291
A0A2Z5YH18_MYCMR	Uncharacterized protein	0,40	-5,25	+	+	-0,2517	0,4005	+	+	0,3532	0,3984
A0A2Z5YHC2_MYCMR	Putative propionyl-CoA carboxylase beta chain 6	0,63	-2,20	+	+	-0,5348	0,6272	+	+	0,2747	0,5521
A0A2Z5YHY7_MYCMR	Universal stress protein	0,28	-2,44	+	+	-0,1956	0,2848	+	+	0,1247	0,2738
A0A2Z5YIF5_MYCMR	Uncharacterized protein	0,12	18,15	+		1,3179	0,1180	+		0,0442	0,2081
A0A2Z5YIS5_MYCMR	Cyclopropane mycolic acid synthase MmaA2	0,09	-5,21	+	+	-0,2993	0,0885	+	+	0,2990	0,2152
A0A2Z5YJ26_MYCMR	2-oxoglutarate oxidoreductase subunit KorA	0,24	-3,51	+	+	-0,2586	0,2405	+	+	0,0482	0,1989
A0A2Z5YJX7_MYCMR	Multifunctional fusion protein	0,39	-3,59	+	+	-0,2617	0,3909	+	+	0,4799	0,4140
A0A2Z5YJX9_MYCMR	ATP-dependent Clp protease ATP-binding subunit ClpX	0,40	-6,05	+	+	-0,4810	0,4045	+	+	0,3248	0,4187
A0A2Z5YKE5_MYCMR	Enoyl-CoA hydratase 2	0,06	-15,20	+	+	-0,2966	0,0626	+	+	0,1157	0,0594
A0A2Z5YKJ3_MYCMR	Uncharacterized protein	0,32	-7,10	+	+	-0,6083	0,3190	+	+	0,2825	0,4258
A0A2Z5YKL3_MYCMR	Polyketide synthase	0,18	-3,66	+	+	-0,7341	0,1844	+	+	0,4482	0,4064
A0A2Z5YKQ1_MYCMR	3-hydroxyisobutyryl-CoA hydrolase	0,06	5,81	+		0,4138	0,0605	+		0,1185	0,1700
A0A2Z5YL55_MYCMR	Glucose-1-phosphate adenylyltransferase	0,12	3,51	+		0,5167	0,1203		+	0,1854	0,1761
A0A2Z5YLZ6_MYCMR	Uncharacterized protein	0,31	-4,05	+	+	-0,2628	0,3084			-0,3160	-0,0455
A0A2Z5YM62_MYCMR	Universal stress protein	0,58	-7,65	+	+	-0,7945	0,5813	+	+	0,0791	0,4225
A0A2Z5YMQ1_MYCMR	Oxidoreductase	0,12	2,23	+		0,2065	0,1174	+	+	0,3130	0,3459
A0A2Z5YMV4_MYCMR	Epoxide hydrolase A	0,32	5,06	+		0,7187	0,3180	+	+	0,2405	0,3600

A0A2Z5YMW7_MYCMR	Uncharacterized protein	0,42	-6,64	+	+	-0,3323	0,4238	+	+	0,3720	0,5940
A0A2Z5YNA2_MYCMR	Uncharacterized protein	0,27	2,30	+		0,4210	0,2651			0,2678	0,2630
A0A2Z5YNJ5_MYCMR	1-acyl-sn-glycerol-3-phosphate acyltransferase	0,63	-4,33	+	+	-0,3781	0,6258	+	+	0,3570	0,6481
A0A2Z5YNT5_MYCMR	Glutamate synthase	0,16	-3,30	+	+	-0,2287	0,1582	+	+	0,2781	0,3218
A0A2Z5YNU9_MYCMR	UDP-galactopyranose mutase	0,02	5,01	+		0,3330	0,0217			-0,5932	-0,0607
A0A2Z5YPL5_MYCMR	Thioredoxin reductase	0,04	-3,31	+		-0,2224	0,0448			-0,0263	0,0047
A0A2Z5YPN8_MYCMR	Long-chain-fatty-acid--AMP ligase FadD32	0,23	-5,50	+	+	-0,2341	0,2257	+	+	0,2100	0,1776
B2HCW1_MYCMM	3-hydroxyisobutyrate dehydrogenase	0,19	2,74	+		0,4090	0,1925			-0,1294	-0,0910
B2HEM3_MYCMM	Conserved protein	0,23	-7,11	+	+	-0,4975	0,2318	+	+	0,3939	0,4653
B2HEV4_MYCMM	Molybdenum cofactor biosynthesis protein MoeB1	0,06	-2,92	+	+	-0,1943	0,0638	+		-0,0150	0,0844
B2HF93_MYCMM	Transcriptional regulatory protein (Possibly marR-family)	0,04	5,70	+		0,6518	0,0354			-0,1396	-0,0393
B2HFI8_MYCMM	Peptidyl-prolyl cis-trans isomerase	0,10	2,90	+		0,7763	0,1039			0,5255	0,4538
B2HFT9_MYCMM	Conserved protein	0,44	-7,18	+	+	-1,2628	0,4379	+	+	0,2093	0,3809
B2HG74_MYCMM	Nitrate/nitrite response regulator protein NarL	0,26	5,74	+		0,9413	0,2597			0,1590	0,2249
B2HGH4_MYCMM	Acyl-CoA dehydrogenase FadE23	0,12	-9,69	+	+	-0,3694	0,1183	+	+	0,1156	0,0842
B2HGH5_MYCMM	Acyl-CoA dehydrogenase FadE24	0,30	-8,67	+	+	-0,2710	0,2972	+	+	0,2017	0,2258
B2HGY0_MYCMM	Aminomethyltransferase	0,25	2,77	+		0,5551	0,2531		+	-0,2001	0,1857
B2HH24_MYCMM	Uncharacterized protein	0,01	-2,71	+		-0,4192	0,0108			-0,1084	0,0383
B2HH70_MYCMM	Molybdopterin biosynthesis protein, MoeY	0,68	-5,80	+	+	-0,7750	0,6812	+	+	0,4854	0,7932
B2HHG1_MYCMM	Transcriptional regulatory protein (Probably AsnC-family)	0,03	-5,32	+		-0,6758	0,0342	+	+	0,1990	0,1712
B2HHL5_MYCMM	Conserved transmembrane protein	0,19	-7,60	+	+	-0,2954	0,1855	+	+	0,2218	0,2931
B2HHR4_MYCMM	Malonyl CoA-acyl carrier protein transacylase FabD	0,27	-4,58	+	+	-0,2806	0,2724	+	+	0,3006	0,3297
B2HHR7_MYCMM	3-oxoacyl-[acyl-carrier protein] synthase 2 KasB	0,30	-3,47	+	+	-0,1135	0,3016	+	+	0,1302	0,1833
B2HIC1_MYCMM	Replicative DNA helicase	0,55	-12,19	+	+	-1,1465	0,5486	+	+	0,2875	0,5080
B2HID7_MYCMM	Transferase	0,43	-2,31	+	+	-0,2089	0,4251	+	+	0,2788	0,5428
B2HIG5_MYCMM	Acetolactate synthase	0,16	-4,96	+	+	-0,3828	0,1602	+	+	0,2679	0,2795
B2HIK4_MYCMM	Pyruvate carboxylase	0,14	-3,95	+	+	-0,0436	0,1437	+	+	0,2097	0,1695
MSL7_MYCMM	Phenolphthiocerol synthesis polyketide synthase type I Pks15/1	0,39	-7,44	+	+	-0,1921	0,3863	+	+	0,2046	0,3970
B2HJ41_MYCMM	ATP-dependent protease ATP-binding subunit ClpC1	0,09	-8,56	+	+	-0,1024	0,0853	+	+	0,1393	0,1074
B2HJK3_MYCMM	Conserved hypothetical alanine and arginine rich protein	0,60	-2,35	+	+	-0,3035	0,6044	+	+	0,6167	0,6928
B2HJW1_MYCMM	Heat shock protein HspX_1	0,92	-7,11	+	+	-0,6182	0,9227	+	+	0,5179	0,8021
B2HJX9_MYCMM	Uncharacterized protein	0,43	-2,67	+	+	-0,6556	0,4298	+	+	0,1479	0,4317
B2HK75_MYCMM	Conserved protein with endoribonuclease L-PSP domain	0,20	3,16	+		0,7725	0,1985			0,2218	0,0791
B2HK82_MYCMM	Cysteine synthase CysK	0,08	3,90	+		0,8065	0,0830			-0,4108	-0,1653
B2HL18_MYCMM	Acyl-CoA dehydrogenase FadE20	0,31	-4,20	+	+	-0,2944	0,3129	+	+	0,3966	0,3988
B2HL69_MYCMM	Membrane protein	0,49	-4,80	+	+	-0,2729	0,4895	+	+	-0,1509	0,3854
B2HLK1_MYCMM	Enoyl-CoA hydratase, EchA21	0,09	-2,61	+	+	-0,0929	0,0879	+	+	0,1752	0,1346
B2HM69_MYCMM	Conserved protein	0,29	4,57	+		1,1327	0,2903	+	+	0,4453	0,6592
B2HML2_MYCMM	Acyltransferase	0,20	-3,45	+	+	-0,4827	0,1982			0,1849	0,1549
B2HMU0_MYCMM	MCE-family protein Mce1A	0,06	12,25	+		1,1358	0,0567			0,0000	0,0435
B2HMW5_MYCMM	Oxidoreductase	0,24	-2,23	+	+	-0,3120	0,2355			-0,2127	-0,1216
B2HN30_MYCMM	Acyl-CoA dehydrogenase FadE5	0,07	-18,88	+	+	-0,4159	0,0678	+	+	0,4020	0,2515
B2HNG9_MYCMM	DNA-(apurinic or apyrimidinic site) lyase	0,42	-4,01	+	+	-0,5113	0,4162	+	+	0,0115	0,4499
B2HP51_MYCMM	Conserved hypothetical secreted protein	0,31	-3,36	+	+	-0,7605	0,3122			-0,8627	0,3175
B2HPC4_MYCMM	ATP-binding protein ABC transporter	0,13	-2,79	+	+	-0,5401	0,1320	+	+	0,2674	0,2579
B2HPS3_MYCMM	Chaperone protein DnaJ	0,54	-3,07	+	+	-0,1655	0,5426	+	+	0,5639	0,6431
B2HQ15_MYCMM	UDP-glucose 6-dehydrogenase, UdgL	0,51	-2,56	+	+	-0,4352	0,5145	+	+	0,4368	0,6666
B2HQ16_MYCMM	UDP-glucose 4-epimerase, GalE5	0,18	-3,93	+	+	-0,2993	0,1767	+	+	0,0526	0,3562
B2HQ37_MYCMM	HpcH_Hpal domain-containing protein	0,43	-8,43	+	+	-0,5871	0,4284	+	+	0,3419	0,5269
B2HQT3_MYCMM	3-hydroxybutyryl-CoA dehydrogenase FadB2	0,09	3,27	+		0,2658	0,0856			-0,1758	-0,0737
B2HQW6_MYCMM	Pyrroline-5-carboxylate reductase	0,07	2,52	+		0,5052	0,0654			0,5575	0,2397
B2HQW9_MYCMM	UDP-glucose 4-epimerase GalE2	0,37	-2,33	+	+	-0,5138	0,3740	+	+	0,4760	0,4225
B2HR29_MYCMM	Acetylornithine aminotransferase	0,22	4,95	+		0,6732	0,2231			0,1953	0,1791
B2HR63_MYCMM	Uncharacterized protein	0,06	6,14	+		0,7516	0,0554			-0,1389	0,0585
B2HRJ1_MYCMM	Glutamyl-tRNA reductase	0,75	-3,17	+	+	-0,4864	0,7483	+	+	0,4019	0,7254
B2HRJ3_MYCMM	Uroporphyrin-III C-methyltransferase HemD	0,67	-3,05	+	+	-0,5424	0,6679	+	+	0,7811	0,7494
B2HRN0_MYCMM	Glycosyl transferase-dolichol-P-sugar synthase	0,47	-2,21	+	+	-0,2560	0,4673		+	-0,0801	0,0838

B2HS01_MYCMM	Transcriptional accessory protein Tex	0,46	-4,92	+	+	-0,7127	0,4588	+	+	0,4480	0,5595
B2HS44_MYCMM	Serine protease HtrA (DegP protein)	0,13	-3,82	+	+	-0,1999	0,1325	+	+	0,1668	0,3215
B2HSE3_MYCMM	Uncharacterized protein	0,19	-5,84	+	+	-0,5934	0,1918	+	+	0,0418	0,2073
B2HSZ6_MYCMM	NADH dehydrogenase Ndh	0,51	-5,78	+	+	-0,2479	0,5129	+	+	0,4908	0,6038
B2HT60_MYCMM	Acyl-[acyl-carrier protein] desaturase DesA2	0,08	-10,45	+	+	-0,4221	0,0840		+	0,0424	0,0714
**B2HD10_MYCMM**	**Chaperonin GroEL1**	**-0,06**	**-0,11**	**+**	**+**	**-0,1067**	**-0,0617**	**+**	**+**	**0,3434**	**0,2262**

#VIITTAUS!
