# Supplementary material for: In vitro and ex vivo proteomics of Mycobacterium marinum biofilms and the development of biofilm-binding synthetic nanobodies
Source: mSystems. 2023 May 15;8(3):e01073-22. doi: 10.1128/msystems.01073-22 (PMC10308901; doi:10.1128/msystems.01073-22)
Supplement: Table S4 — M. marinum proteins shared between the in vitro biofilm surfaces identified using the biotinylation and cell-surface shaving (data from Table S3 and Savijoki et al., 2021) proteomics. [file msystems.01073-22-s0004.pdf]

Table S4. *M. marinum* proteins shared between the in vitro biofilm surface (biotinylation and shaving proteomics) and ex vivo proteomes (see Table above), and proteins commonly identified after the in vitro biotinylation and cell-surface shaving proteomics (See Table below).

UniProt_ID	Protein name
Shared between the in vitro and ex vivo proteomes	
**Q8GAR8**	**Chaperonin GroEL2 60 kDa chaperonin (Fragment) OS=MYCO**
A0A100I242	35 kDa protein OS=MYCO
A0A2Z5YMM5	3-hydroxyacyl-CoA dehydrogenase OS=MYCO
A0A100I319	50S ribosomal protein L10 OS=MYCO
A0A100I336	50S ribosomal protein L7/L12 OS=MYCO
B2HFB9	Acyl-CoA thiolase FadA OS=MYCO
A0A117DW52	Alkyl hydroperoxide reductase C OS=MYCO
A0A3E2MNB5	Alpha-hydroxy-acid oxidizing enzyme OS=MYCO
A0A2Z5YJX5	ATP synthase subunit alpha OS=MYCO
A0A2Z5YJD6	ATP synthase subunit beta OS=MYCO
A0A100I5P3	ATP-dependent Clp protease proteolytic subunit OS=MYCO
B2HE73	Catalase-peroxidase OS=MYCO
A0A124BWP8	Chaperone protein DnaK OS=MYCO
A0A2Z5YI10	Chaperone protein HtpG OS=MYCO
A0A100I1G0	Co-chaperonin GroES OS=MYCO
A0A2Z5YAQ1	DNA topoisomerase (ATP-hydrolyzing) OS=MYCO
A0A100I012	DNA-directed RNA polymerase subunit alpha OS=MYCO
A0A100I9G6	Elongation factor G OS=MYCO
A0A100IDP2	Enoyl-[acyl-carrier-protein] reductase [NADH] OS=MYCO
B2HJI8	ESAT-6-like protein OS=MYCO
A0A117DYA5	ESAT-6-like protein OS=MYCO
A0A2D1N551	ESAT-6-like protein OS=MYCO
A0A117DTU2	Ferritin OS=MYCO
A0A117DWW0	Glutamine synthetase OS=MYCO
A0A100IC61	Glyceraldehyde-3-phosphate dehydrogenase OS=MYCO
A0A2Z5YDP2	Glyceraldehyde-3-phosphate dehydrogenase OS=MYCO
B2HP84	Glyceraldehyde-3-phosphate dehydrogenase OS=MYCO
A0A2Z5YJR6	Malate dehydrogenase OS=MYCO
A0A2Z5YLZ1	Membrane protein OS=MYCO
A0A117DUS2	Methionine ABC transporter ATP-binding protein OS=MYCO
A0A2Z5YCH3	Phthiocerol diMYCO
A0A100I0S1	Serine/threonine kinase OS=MYCO
A0A2Z5YLC4	Succinate--CoA ligase [ADP-forming] subunit beta OS=MYCO
**UniProt_ID**	**Protein name**
Shared between the in vitro biotinylation and cell-surface shaved proteomes	
**A0A117DW44**	**Chaperonin GroEL2 OS=MYCO**
A0A2Z5YHF3	14 kDa antigen OS=Mycobacterium marinum OX=1781 GN=hspX PE=3 SV=1
A0A2Z5YF45	30S ribosomal protein S1 OS=Mycobacterium marinum OX=1781 GN=rpsA PE=4 SV=1
A0A100I242	35 kDa protein OS=Mycobacterium pseudoshottsii JCM 15466 OX=1136880 GN=MPS_1602 PE=3 SV=1
A0A100IF67	3-hydroxyacyl-CoA dehydrogenase OS=Mycobacterium pseudoshottsii JCM 15466 OX=1136880 GN=MPS_5057 PE=3 SV=1
B2HHR6	3-oxoacyl-[acyl-carrier protein] synthase 1 KasA OS=Mycobacterium marinum (strain ATCC BAA-535 / M) OX=216594 GN=kasA PE=3 SV=1
B2HHR7	3-oxoacyl-[acyl-carrier protein] synthase 2 KasB OS=Mycobacterium marinum (strain ATCC BAA-535 / M) OX=216594 GN=kasB PE=3 SV=1
A0A2Z5YF14	3-oxoacyl-[acyl-carrier-protein] reductase FabG OS=Mycobacterium marinum OX=1781 GN=fabG_17 PE=4 SV=1
B2HRM4	3-oxoacyl-[acyl-carrier-protein] synthase 3 OS=Mycobacterium marinum (strain ATCC BAA-535 / M) OX=216594 GN=fabH PE=3 SV=1
B2HJQ1	4-hydroxy-3-methylbut-2-en-1-yl diphosphate synthase (flavodoxin) OS=Mycobacterium marinum (strain ATCC BAA-535 / M) OX=216594 GN=ispG PE=3 SV=1
B2HSI8	50S ribosomal protein L10 OS=Mycobacterium marinum (strain ATCC BAA-535 / M) OX=216594 GN=rplJ PE=3 SV=1
B2HSI9	50S ribosomal protein L7/L12 OS=Mycobacterium marinum (strain ATCC BAA-535 / M) OX=216594 GN=rplL PE=3 SV=1

B2HSY6	6-phosphogluconate dehydrogenase, decarboxylating OS=Mycobacterium marinum (strain ATCC BAA-535 / M) OX=216594 GN=gnd1 PE=3 SV=1
A0A2Z5YH20	8-demethyl-8-aminoriboflavin-5'-phosphate synthase RosB OS=Mycobacterium marinum OX=1781 GN=MMRN_34410 PE=4 SV=1
B2HH05	Acyl-[acyl-carrier protein] desaturase DesA1 OS=Mycobacterium marinum (strain ATCC BAA-535 / M) OX=216594 GN=desA1 PE=3 SV=1
B2HMA0	Acyl-[acyl-carrier protein] desaturase DesA1_1 OS=Mycobacterium marinum (strain ATCC BAA-535 / M) OX=216594 GN=desA1_1 PE=3 SV=1
A0A2Z5Y9Z6	Acyl-ACP thioesterase OS=Mycobacterium marinum OX=1781 GN=DAVIS_00811 PE=4 SV=1
B2HPB8	Acyl-CoA dehydrogenase FadE15 OS=Mycobacterium marinum (strain ATCC BAA-535 / M) OX=216594 GN=fadE15 PE=3 SV=1
B2HMD3	Acyl-CoA dehydrogenase FadE17_1 OS=Mycobacterium marinum (strain ATCC BAA-535 / M) OX=216594 GN=fadE17_1 PE=3 SV=1
B2HGH4	Acyl-CoA dehydrogenase FadE23 OS=Mycobacterium marinum (strain ATCC BAA-535 / M) OX=216594 GN=fadE23 PE=3 SV=1
B2HN30	Acyl-CoA dehydrogenase FadE5 OS=Mycobacterium marinum (strain ATCC BAA-535 / M) OX=216594 GN=fadE5 PE=3 SV=1
B2HJ06	Acyl-CoA dehydrogenase OS=Mycobacterium marinum (strain ATCC BAA-535 / M) OX=216594 GN=MMAR_5065 PE=4 SV=1
A0A2Z5YHY9	Adenosine kinase OS=Mycobacterium marinum OX=1781 GN=adoK PE=4 SV=1
A0A2Z5YAG3	Adenylate kinase OS=Mycobacterium marinum OX=1781 GN=adk PE=3 SV=1
B2HLS7	Aldehyde dehydrogenase OS=Mycobacterium marinum (strain ATCC BAA-535 / M) OX=216594 GN=MMAR_0359 PE=3 SV=1
B2HH61	Aldehyde dehydrogenase OS=Mycobacterium marinum (strain ATCC BAA-535 / M) OX=216594 GN=MMAR_4912 PE=3 SV=1
B2HQS0	Aldehyde dehydrogenase, PutA_1 OS=Mycobacterium marinum (strain ATCC BAA-535 / M) OX=216594 GN=putA_1 PE=3 SV=1
B2HD60	Alkyl hydroperoxide reductase C OS=Mycobacterium marinum (strain ATCC BAA-535 / M) OX=216594 GN=ahpC PE=4 SV=1
A0A2Z5YGW4	All-trans-nonaprenyl-diphosphate synthase (Geranyl-diphosphate specific) OS=Mycobacterium marinum OX=1781 GN=idsA2 PE=3 SV=1
B2HMC7	Alpha-E domain-containing protein OS=Mycobacterium marinum (strain ATCC BAA-535 / M) OX=216594 GN=MMAR_3731 PE=4 SV=1
A0A2Z5YLT8	Amidophosphoribosyltransferase OS=Mycobacterium marinum OX=1781 GN=purF PE=3 SV=1
B2HND5	Amino acid decarboxylase OS=Mycobacterium marinum (strain ATCC BAA-535 / M) OX=216594 GN=MMAR_2184 PE=4 SV=1
A0A124BU85	Antibiotic transporter OS=Mycobacterium pseudoshottsii JCM 15466 OX=1136880 GN=MPS_0533 PE=3 SV=1
B2HR32	Argininosuccinate synthase OS=Mycobacterium marinum (strain ATCC BAA-535 / M) OX=216594 GN=argG PE=3 SV=1
B2HFX4	ATP phosphoribosyltransferase OS=Mycobacterium marinum (strain ATCC BAA-535 / M) OX=216594 GN=hisG PE=3 SV=1
A0A2Z5YN75	ATPase AAA OS=Mycobacterium marinum OX=1781 GN=moxR2 PE=4 SV=1
A0A2Z5YDT4	ATPase OS=Mycobacterium marinum OX=1781 GN=moxR1 PE=4 SV=1
B2HIS9	ATP-dependent 6-phosphofructokinase OS=Mycobacterium marinum (strain ATCC BAA-535 / M) OX=216594 GN=pfkA_1 PE=3 SV=1
A0A100I781	ATP-dependent Clp protease ATP-binding subunit ClpX OS=Mycobacterium pseudoshottsii JCM 15466 OX=1136880 GN=clpX PE=3 SV=1
B2HNG5	ATP-dependent Clp protease proteolytic subunit OS=Mycobacterium marinum (strain ATCC BAA-535 / M) OX=216594 GN=clpP1 PE=3 SV=1
B2HJ41	ATP-dependent protease ATP-binding subunit ClpC1 OS=Mycobacterium marinum (strain ATCC BAA-535 / M) OX=216594 GN=clpC1 PE=4 SV=1
A0A117DYK6	Beta-carbonic anhydrase 1 OS=Mycobacterium pseudoshottsii JCM 15466 OX=1136880 GN=mtcA1 PE=3 SV=1
A0A2Z5YAN6	Bifunctional o-acetylhomoserine/o-acetylserine sulfhydrylase OS=Mycobacterium marinum OX=1781 GN=metC PE=3 SV=1
B2HDR8	Bifunctional protein FolD OS=Mycobacterium marinum (strain ATCC BAA-535 / M) OX=216594 GN=folD PE=3 SV=1
B2HMK7	Bifunctional UDP-galactofuranosyl transferase GlfT OS=Mycobacterium marinum (strain ATCC BAA-535 / M) OX=216594 GN=glfT PE=4 SV=1
B2HQ93	Biotin synthase OS=Mycobacterium marinum (strain ATCC BAA-535 / M) OX=216594 GN=bioB PE=3 SV=1
A0A2Z5Y9J9	Calcium dodecin OS=Mycobacterium marinum OX=1781 GN=secE2 PE=4 SV=1
B2HPS3	Chaperone protein DnaJ OS=Mycobacterium marinum (strain ATCC BAA-535 / M) OX=216594 GN=dnaJ PE=3 SV=1
B2HM79	Chaperone protein DnaJ OS=Mycobacterium marinum (strain ATCC BAA-535 / M) OX=216594 GN=dnaJ PE=3 SV=1
B2HF81	Citrate synthase OS=Mycobacterium marinum (strain ATCC BAA-535 / M) OX=216594 GN=gltA2 PE=3 SV=1
B2HD09	Co-chaperonin GroES OS=Mycobacterium marinum (strain ATCC BAA-535 / M) OX=216594 GN=groES PE=3 SV=1
B2HP49	Coenzyme A biosynthesis bifunctional protein CoaBC OS=Mycobacterium marinum (strain ATCC BAA-535 / M) OX=216594 GN=dfp PE=3 SV=1
B2HKB1	Conserved membrane protein OS=Mycobacterium marinum (strain ATCC BAA-535 / M) OX=216594 GN=MMAR_5203 PE=4 SV=1
A0A100IC89	Conserved membrane protein OS=Mycobacterium pseudoshottsii JCM 15466 OX=1136880 GN=MPS_4251 PE=4 SV=1
B2HH95	Conserved oxidoreductase OS=Mycobacterium marinum (strain ATCC BAA-535 / M) OX=216594 GN=MMAR_1575 PE=4 SV=1
B2HKV8	Conserved protein OS=Mycobacterium marinum (strain ATCC BAA-535 / M) OX=216594 GN=MMAR_1930 PE=4 SV=1
B2HFV8	Conserved protein OS=Mycobacterium marinum (strain ATCC BAA-535 / M) OX=216594 GN=MMAR_3087 PE=3 SV=1
B2HPT1	Conserved secreted protein OS=Mycobacterium marinum (strain ATCC BAA-535 / M) OX=216594 GN=MMAR_0647 PE=4 SV=1
B2HSP5	Conserved short-chain dehydrogenase OS=Mycobacterium marinum (strain ATCC BAA-535 / M) OX=216594 GN=MMAR_2623 PE=3 SV=1
A0A100IDG2	Cyclopropane mycolic acid synthase 3 OS=Mycobacterium pseudoshottsii JCM 15466 OX=1136880 GN=pcaA PE=3 SV=1
B2HL85	Cysteine synthase OS=Mycobacterium marinum (strain ATCC BAA-535 / M) OX=216594 GN=cysK1 PE=1 SV=1
A0A2Z5YE32	Cytochrome bd ubiquinol oxidase subunit 1 OS=Mycobacterium marinum OX=1781 GN=cydA PE=3 SV=1
B2HEM8	D-alpha-D-mannose-1-phosphate guanylyltransferase ManB OS=Mycobacterium marinum (strain ATCC BAA-535 / M) OX=216594 GN=manB PE=4 SV=1
B2HR80	Diaminopimelate decarboxylase OS=Mycobacterium marinum (strain ATCC BAA-535 / M) OX=216594 GN=lysA PE=3 SV=1
B2HFB2	DNA helicase OS=Mycobacterium marinum (strain ATCC BAA-535 / M) OX=216594 GN=ercc3 PE=3 SV=1

A0A2Z5YM82	DNA-binding response regulator OS=Mycobacterium marinum OX=1781 GN=phoP PE=4 SV=1
B2HCX4	DNA-directed RNA polymerase subunit alpha OS=Mycobacterium marinum (strain ATCC BAA-535 / M) OX=216594 GN=rpoA PE=3 SV=1
B2HIE1	Electron transfer flavoprotein (Alpha-subunit) FixB OS=Mycobacterium marinum (strain ATCC BAA-535 / M) OX=216594 GN=fixB PE=3 SV=1
B2HSL2	Elongation factor G OS=Mycobacterium marinum (strain ATCC BAA-535 / M) OX=216594 GN=fusA PE=3 SV=1
B2HJN3	Elongation factor Ts OS=Mycobacterium marinum (strain ATCC BAA-535 / M) OX=216594 GN=tsf PE=3 SV=1
B2HSL3	Elongation factor Tu OS=Mycobacterium marinum (strain ATCC BAA-535 / M) OX=216594 GN=tuf PE=3 SV=1
A0A2Z5YNH4	Enoyl-CoA hydratase OS=Mycobacterium marinum OX=1781 GN=echA21 PE=3 SV=1
B2HJI8	ESAT-6-like protein OS=Mycobacterium marinum (strain ATCC BAA-535 / M) OX=216594 GN=esxB_1 PE=3 SV=1
B2HSU5	ESX-5 secretion-associated protein EspG5 OS=Mycobacterium marinum (strain ATCC BAA-535 / M) OX=216594 GN=espG5 PE=1 SV=1
B2HPV3	Fructose-bisphosphate aldolase OS=Mycobacterium marinum (strain ATCC BAA-535 / M) OX=216594 GN=fba PE=3 SV=1
B2HT54	Fumarate hydratase class II OS=Mycobacterium marinum (strain ATCC BAA-535 / M) OX=216594 GN=fum PE=1 SV=1
A0A2Z5Y8S5	Fumarate reductase OS=Mycobacterium marinum OX=1781 GN=frdB PE=3 SV=1
B2HIF7	Glutamyl-tRNA(Gln) amidotransferase subunit A OS=Mycobacterium marinum (strain ATCC BAA-535 / M) OX=216594 GN=gatA PE=3 SV=1
B2HDW7	Glycerol-3-phosphate dehydrogenase OS=Mycobacterium marinum (strain ATCC BAA-535 / M) OX=216594 GN=glpD2 PE=3 SV=1
A0A100I6Z8	Hemoprotein HemQ OS=Mycobacterium pseudoshottsii JCM 15466 OX=1136880 GN=MPS_2528 PE=4 SV=1
B2HQL8	Homoserine dehydrogenase OS=Mycobacterium marinum (strain ATCC BAA-535 / M) OX=216594 GN=thrA PE=3 SV=1
B2HDS6	Homoserine O-acetyltransferase OS=Mycobacterium marinum (strain ATCC BAA-535 / M) OX=216594 GN=metXA PE=3 SV=1
A0A2Z5YE94	HpcH/HpaI aldolase/citrate lyase family protein OS=Mycobacterium marinum OX=1781 GN=DAVIS_04816 PE=4 SV=1
B2HQX7	Indole-3-glycerol phosphate synthase OS=Mycobacterium marinum (strain ATCC BAA-535 / M) OX=216594 GN=trpC PE=3 SV=1
A0A2Z5Y7K4	Inositol-3-phosphate synthase OS=Mycobacterium marinum OX=1781 GN=ino1 PE=3 SV=1
B2HS42	Iron-sulfur cluster carrier protein OS=Mycobacterium marinum (strain ATCC BAA-535 / M) OX=216594 GN=mrp PE=3 SV=1
B2HQT2	Isocitrate lyase OS=Mycobacterium marinum (strain ATCC BAA-535 / M) OX=216594 GN=icl PE=4 SV=1
B2HKL7	Ketoacyl reductase OS=Mycobacterium marinum (strain ATCC BAA-535 / M) OX=216594 GN=MMAR_0254 PE=3 SV=1
A0A2Z5YEW0	L-gulono-1,4-lactone dehydrogenase OS=Mycobacterium marinum OX=1781 GN=DAVIS_03617 PE=3 SV=1
A0A2Z5YA32	Light-repressed protein A OS=Mycobacterium marinum OX=1781 GN=lrtA PE=4 SV=1
A0A117DUB3	LLM class F420-dependent oxidoreductase OS=Mycobacterium pseudoshottsii JCM 15466 OX=1136880 GN=MPS_1448 PE=4 SV=1
B2HMK0	Long-chain-fatty-acid--AMP ligase FadD32 OS=Mycobacterium marinum (strain ATCC BAA-535 / M) OX=216594 GN=fadD32 PE=1 SV=1
B2HRH5	Malate dehydrogenase OS=Mycobacterium marinum (strain ATCC BAA-535 / M) OX=216594 GN=mdh PE=3 SV=1
A0A100I5M2	Membrane protein OS=Mycobacterium pseudoshottsii JCM 15466 OX=1136880 GN=MPS_2439 PE=4 SV=1
A0A2Z5Y9S2	Mycolic acid methyltransferase MmaA1 OS=Mycobacterium marinum OX=1781 GN=umaA1 PE=3 SV=1
B2HLU7	NAD(P) transhydrogenase subunit beta OS=Mycobacterium marinum (strain ATCC BAA-535 / M) OX=216594 GN=pntB PE=3 SV=1
B2HSZ6	NADH dehydrogenase Ndh OS=Mycobacterium marinum (strain ATCC BAA-535 / M) OX=216594 GN=ndh PE=3 SV=1
B2HHG7	NADP-dependent alcohol dehydrogenase Adh OS=Mycobacterium marinum (strain ATCC BAA-535 / M) OX=216594 GN=adh PE=3 SV=1
B2HRD2	Non-specific serine/threonine protein kinase OS=Mycobacterium marinum (strain ATCC BAA-535 / M) OX=216594 GN=pknH PE=4 SV=1
B2HK80	Peptidoglycan glycosyltransferase OS=Mycobacterium marinum (strain ATCC BAA-535 / M) OX=216594 GN=ponA2 PE=4 SV=1
B2HGU5	Phospho-2-dehydro-3-deoxyheptonate aldolase OS=Mycobacterium marinum (strain ATCC BAA-535 / M) OX=216594 GN=aroG PE=3 SV=1
B2HQA6	Phosphoribosyl isomerase A OS=Mycobacterium marinum (strain ATCC BAA-535 / M) OX=216594 GN=priA PE=3 SV=1
B2HMJ8	Propionyl-CoA carboxylase beta chain 4 AccD4_1 OS=Mycobacterium marinum (strain ATCC BAA-535 / M) OX=216594 GN=accD4_1 PE=3 SV=1
B2HEK7	Propionyl-CoA carboxylase beta chain 5 AccD5 OS=Mycobacterium marinum (strain ATCC BAA-535 / M) OX=216594 GN=accD5 PE=3 SV=1
B2HPS2	Protein GrpE OS=Mycobacterium marinum (strain ATCC BAA-535 / M) OX=216594 GN=grpE PE=3 SV=1
A0A2Z5YA71	Putative ABC transporter ATP-binding protein OS=Mycobacterium marinum OX=1781 GN=mkl PE=4 SV=1
A0A2Z5YGP6	Putative oxidoreductase EphD OS=Mycobacterium marinum OX=1781 GN=ephD PE=4 SV=1
A0A100I5R9	Putative succinate-semialdehyde dehydrogenase [NADP(+)] 2 OS=Mycobacterium pseudoshottsii JCM 15466 OX=1136880 GN=gabD2 PE=3 SV=1
B2HP67	Riboflavin biosynthesis protein RibBA OS=Mycobacterium marinum (strain ATCC BAA-535 / M) OX=216594 GN=ribBA PE=3 SV=1
B2HP63	Riboflavin biosynthesis protein RibD OS=Mycobacterium marinum (strain ATCC BAA-535 / M) OX=216594 GN=ribG PE=3 SV=1
B2HHG0	Ribonucleoside-diphosphate reductase OS=Mycobacterium marinum (strain ATCC BAA-535 / M) OX=216594 GN=nrdE PE=3 SV=1
B2HEQ5	Ribosome hibernation promoting factor OS=Mycobacterium marinum (strain ATCC BAA-535 / M) OX=216594 GN=hpf PE=3 SV=1
A0A100I7W7	RNase H OS=Mycobacterium pseudoshottsii JCM 15466 OX=1136880 GN=MPS_3100 PE=4 SV=1
B2HT61	Serine hydroxymethyltransferase OS=Mycobacterium marinum (strain ATCC BAA-535 / M) OX=216594 GN=glyA1 PE=3 SV=1
A0A100I0S1	Serine/threonine kinase OS=Mycobacterium pseudoshottsii JCM 15466 OX=1136880 GN=MPS_0988 PE=4 SV=1
A0A117DXW7	Short-chain dehydrogenase OS=Mycobacterium pseudoshottsii JCM 15466 OX=1136880 GN=MPS_4312 PE=4 SV=1
B2HN36	Succinate dehydrogenase (Iron-sulfur subunit), SdhA_1 OS=Mycobacterium marinum (strain ATCC BAA-535 / M) OX=216594 GN=sdhA_1 PE=4 SV=1
A0A2Z5YL10	Succinate--CoA ligase [ADP-forming] subunit alpha OS=Mycobacterium marinum OX=1781 GN=sucD PE=3 SV=1

B2HED1	Succinate--CoA ligase [ADP-forming] subunit beta OS=Mycobacterium marinum (strain ATCC BAA-535 / M) OX=216594 GN=sucC PE=3 SV=1
B2HQL7	Threonine synthase OS=Mycobacterium marinum (strain ATCC BAA-535 / M) OX=216594 GN=thrC PE=3 SV=1
B2HSH0	Transcription termination/antitermination protein NusG OS=Mycobacterium marinum (strain ATCC BAA-535 / M) OX=216594 GN=nusG PE=3 SV=1
B2HP86	Triosephosphate isomerase OS=Mycobacterium marinum (strain ATCC BAA-535 / M) OX=216594 GN=tpiA PE=3 SV=1
B2HQX9	Tryptophan synthase alpha chain OS=Mycobacterium marinum (strain ATCC BAA-535 / M) OX=216594 GN=trpA PE=3 SV=1
B2HQ15	UDP-glucose 6-dehydrogenase, UdgL OS=Mycobacterium marinum (strain ATCC BAA-535 / M) OX=216594 GN=udgL PE=3 SV=1
B2HMP7	Uncharacterized protein OS=Mycobacterium marinum (strain ATCC BAA-535 / M) OX=216594 GN=MMAR_5410 PE=3 SV=1
