# Supplementary material for: In vitro and ex vivo proteomics of Mycobacterium marinum biofilms and the development of biofilm-binding synthetic nanobodies
Source: mSystems. 2023 May 15;8(3):e01073-22. doi: 10.1128/msystems.01073-22 (PMC10308901; doi:10.1128/msystems.01073-22)
Supplement: Table S5 — List of all mycobacterial proteins identified fron granulomas using LC-MS/MS. Both the original and normalized raw intensity values (normalized to combined raw intensity values of all identified proteins per replica) for each protein are shown. GroEL chaperones are shown with red letters. [file msystems.01073-22-s0005.pdf]

Table S5. List of all mycobacterial proteins identified fron granulomas using LC-MS/MS. Raw intensity values for each protein are shown. GroEL chaperones are shown with red letters.

UniprotKB/AC_ID	Protein name	Raw intensity values									
		Intensity 1	Intensity 2	Intensity 3	Intensity 4	Intensity 5	Intensity 6	Intensity 7	Intensity 8	Intensity 9	Intensity 10
tr|A0A117DW44|A0A117DW44_9MYCO	**GroEL2 chaperonin**	**2209700**	**585940**	**1333200**	**1284400**	**834220**	**367830**	**476970**	**1038600**	**366790**	**378520**
sp|B2HSL3|EFTU_MYCMM	Elongation factor Tu	1427900	472260	895060	750640	300480	193920	345440	1101100	249760	203240
tr|A0A2Z5YCH3|A0A2Z5YCH3_MYCMR	Phthiocerol dimycocerosate exporter MmpL7	**994520**	**1171300**	**2125700**	**3523800**	**2948300**	**3304700**	**1664400**	**1954600**	**1458400**	**69199**
tr|A0A2Z5YEF1|A0A2Z5YEF1_MYCMR	Integration host factor	867420	343220	460730	539060	161390	217790	414260	198430	215630	
sp|B2HD09|CH10_MYCMM	10 kDa chaperonin	851170	406620	487000	527390	375250	205210	379810	496470	240550	184160
tr|A0A117DYA5|A0A117DYA5_9MYCO	ESAT-6-like protein	804770	189890	586200	562430	695540	383610	642290	624450	247150	68679
tr|B2HR04|B2HR04_MYCMM	Iron-regulated conserved protein	666850	232930	465970	552570	89361	196950	261100	358920	128310	68851
tr|B2HHR5|B2HHR5_MYCMM	Acyl carrier protein	596770	113850	292950	103300	176000	89594	82756	267710	195370	
tr|B2HQM8|B2HQM8_MYCMM	Two-component sensor and regulator	**593220**	**456570**	**3541300**	**420750**	**1309200**	**276760**	**328660**	**2298000**	**199470**	**913800**
tr|A0A2Z5YA35|A0A2Z5YA35_MYCMR	50S ribosomal protein L7/L12	525700	563900	432880	646860	285680	310620	510710	470420	386530	298670
tr|A0A2Z5YFU6|A0A2Z5YFU6_MYCMR	Catalase-peroxidase	481690	230070	323760	453170	254770	40349	236330	355280	187030	73596
tr|B2HGQ8|B2HGQ8_MYCMM	Antigen 84	455740	119380	155570	225230	299800	74230	97584	245230	35150	32947
tr|B2HII3|B2HII3_MYCMM	DNA-binding protein HU homolog	332960	51078	196720	159200	181270	43564	68498	216220	46982	34122
tr|A0A2Z5YBD3|A0A2Z5YBD3_MYCMR	2-methylcitrate dehydratase	294930	20563	478380	50509	279060	15616		425750	7978,2	9836,2
tr|A0A3E2MR45|A0A3E2MR45_MYCMR	ATP synthase subunit alpha	290340	113680	229810	163390	168010	49985	84766	146500	89171	31098
tr|A0A2Z5Y9K5|A0A2Z5Y9K5_MYCMR	Heparin-binding hemagglutinin	277320	122530	414000	216590	210120	13610	133690	355700		8119,9
tr|A0A100I595|A0A100I595_9MYCO	Peptidyl-prolyl cis-trans isomerase	275050	11515	43484	50994	29068		43849	37557		
tr|A0A100I1L1|A0A100I1L1_9MYCO	**GroEL1 chaperonin**	**267350**	**8235,9**	**165500**	**99165**	**71396**	**50896**	**69680**	**39964**	**804,32**	
tr|A0A100I3X5|A0A100I3X5_9MYCO	ATP synthase subunit beta	261460	65705	86414	123910	115910	31365	66267	185210	54488	
tr|A0A100IC61|A0A100IC61_9MYCO	Glyceraldehyde-3-phosphate dehydrogenase	259950	258720	268630	278540	222260	104350	154060	260110	140430	20570
tr|B2HD57|B2HD57_MYCMM	L-lactate dehydrogenase (Cytochrome) LldD2	256880	34085	74190	312020	151810	48161	46410	86653	57017	41007
tr|B2HFB8|B2HFB8_MYCMM	3-hydroxyacyl-CoA dehydrogenase	239580	153530	93310	130250	77415	34504	70199	82596	42724	8942,3
tr|A0A100IF16|A0A100IF16_9MYCO	Nucleoid-associated protein Lsr2	232900	139550	272170	188930	191900	101450	190350	241400	101750	75543
tr|B2HSI8|B2HSI8_MYCMM	50S ribosomal protein L10	212400	33117	128590	69306	24769	31535	19721	48620		225430
tr|B2HIE1|B2HIE1_MYCMM	Electron transfer flavoprotein (Alpha-subunit) FixB	209080	85835	108040	148040	73867	29311	46177	125300	45634	29767
tr|A0A3E2MWQ5|A0A3E2MWQ5_MYCMR	Potential acyltransferase	**192840**	**347360**	**609480**	**1677000**	**449340**	**2041500**	**558640**	**2241600**	**403370**	**11724**
tr|A0A2Z5YPB5|A0A2Z5YPB5_MYCMR	ESAT-6-like protein	188700	82010	118520	181160	154740	86331	140310	196880	2866,6	91919
tr|A0A117DZM6|A0A117DZM6_9MYCO	30S ribosomal protein S2	185250	25808	216090	194800	91162	28475	63375	163250	59350	45946
tr|B2HDH7|B2HDH7_MYCMM	ESAT-6-like protein	170420	7262,3	131740	33578	108980	19743	12219	85983	26682	133440
tr|B2HJ81|B2HJ81_MYCMM	Cold shock protein A CspA_1	164370	84024	211660	126680	111010	90795	95989	216220	73981	1488300
tr|A0A3E2MSW2|A0A3E2MSW2_MYCMR	FadA_5 Putative acyltransferase	161370	51876	95720	1158,8	56311	50131	26330	66663	1373	12786
sp|B2HSN7|RS3_MYCMM	30S ribosomal protein S3	140070	66395	119100	115270	99709	42665	40815	39191	23134	14785
tr|B2HJ41|B2HJ41_MYCMM	ATP-dependent protease ATP-binding subunit ClpC1	135910	95902	104210	160010	53694	23874	40832	132450	57203	
sp|B2HRH5|MDH_MYCMM	Malate dehydrogenase	135470	86510	68989	56780	30564	19764	92040	41336	21426	51380
tr|A0A2Z5YMC1|A0A2Z5YMC1_MYCMR	Phosphate-binding protein PstS	111560	81526	100040	107320	64038	37322	54659	71612	31780	10267
tr|B2HLS8|B2HLS8_MYCMM	Short-chain type dehydrogenase/reductase	106980	23575	9462,7	53148	64206	17238	24198	12767	14041	9214,4
tr|A0A100I9D3|A0A100I9D3_9MYCO	Single-stranded DNA-binding protein	100690	8857,3	47167	66626		15656		62674		16018
tr|B2HHW3|B2HHW3_MYCMM	Uncharacterized protein	99447	25383	67086	103890	36547	30290	68257	69052	42785	6098,6
tr|A0A100I516|A0A100I516_9MYCO	Electron transfer flavoprotein subunit beta	97184	43304	88207	67082	46363	29146	52380	29007	24287	
tr|A0A2Z5YNH3|A0A2Z5YNH3_MYCMR	Uncharacterized protein	91257		120410	27410	20587			63682		
tr|A0A2Z5Y928|A0A2Z5Y928_MYCMR	Chaperone protein DnaK	89033	11324	25869	26190	24127	17711	15272	53661	11722	
sp|B2HEP6|SAHH_MYCMM	Adenosylhomocysteinase	88599	61024	110660	60357		1389,6	11896	36965	2311,6	15957
sp|B2HSH2|RL1_MYCMM	50S ribosomal protein L1	87777	26997	56257	51718	34542	16830	31826	63187	15270	
tr|B2HLI7|B2HLI7_MYCMM	19 kDa lipoprotein antigen LpqH	82490		94163		11261			111720		
tr|B2HK73|B2HK73_MYCMM	Conserved regulatory protein	81368		78496	41209	19689			62287	6492,3	
tr|A0A100I193|A0A100I193_9MYCO	50S ribosomal protein L29	78199	2051,2	56226		28979			51228	2378,1	
tr|B2HD60|B2HD60_MYCMM	Alkyl hydroperoxide reductase C	73733		53857		6346,4			12170		
tr|A0A117DVM5|A0A117DVM5_9MYCO	ATP-dependent Clp protease proteolytic subunit	73186		20487	49877	43601		457740	38610		
tr|A0A100HZA7|A0A100HZA7_9MYCO	Biotin carboxyl carrier protein	73080		26582	52421	26781		12026	53070	4202,3	
tr|B2HK75|B2HK75_MYCMM	Conserved protein with endoribonuclease L-PSP domain	72906		28753					23096		
sp|B2HCT5|RS8_MYCMM	30S ribosomal protein S8	71574	74488	1982,4	110320	50592	34898		78034	28672	45702
tr|A0A2Z5Y9T8|A0A2Z5Y9T8_MYCMR	Dihydrolipoyl dehydrogenase	70416	10143	27179	38137	22016					
tr|B2HD66|B2HD66_MYCMM	Bacterioferritin	64041	17356	46252	27444	38315		16822	39798	10766	
tr|B2HD91|B2HD91_MYCMM	Conserved secreted protein	63797			40050	14929	21009		6949,3	14137	
tr|B2HDC4|B2HDC4_MYCMM	Isocitrase	63625	98310	23271	46236	37938		87934			
tr|B2HGZ8|B2HGZ8_MYCMM	Uncharacterized protein	61472	18168	47309	67828	39034	8025,3	14739	64251	8409	19425
tr|A0A100I911|A0A100I911_9MYCO	3-oxoacyl-ACP synthase	58993	28761	30840	69461	43637	16311	27826	75220		
tr|B2HGV2|B2HGV2_MYCMM	Polyketide cyclase	58901	32993		43194	28926		20930	40020		
sp|B2HSN0|RS10_MYCMM	30S ribosomal protein S10	55244	11108	21448	35340	12839	12447	16127	16555	7914,5	

tr|A0A100I6V1|A0A100I6V1_9MYCO	Succinyl-CoA ligase [ADP-forming] beta chain	46311		11349	18890	10258			16350		
sp|B2HCT2|RL24_MYCMM	50S ribosomal protein L24	44080		9654,1	17979				23192		
tr|B2HDJ4|B2HDJ4_MYCMM	50S ribosomal protein L25	42400		11963		15256			12667		
tr|B2HSJ2|B2HSJ2_MYCMM	Ribonucleotide-transport ATP-binding protein ABC transporter, Mkl	38599	30159	19751		17730			0		
tr|B2HQ06|B2HQ06_MYCMM	Interferon-induced transmembrane protein	37342	11888	33114		11267	6633,4		3413,9		7701,7
tr|B2HJZ8|B2HJZ8_MYCMM	Chaperone protein HtpG	36248		33142	55727	26096	17258	7790	32716		
tr|B2HPS9|B2HPS9_MYCMM	Chaperone protein ClpB	35513		20625		14681			24697		
tr|A0A117DY09|A0A117DY09_9MYCO	D-3-phosphoglycerate dehydrogenase	33005		23948					24953		
sp|B2HIC0|RL9_MYCMM	50S ribosomal protein L9	32785	20462	17151	35069	18066	14085	31277	15001	24082	7427,4
tr|B2HL00|B2HL00_MYCMM	Conserved 35 kDa alanine rich protein	27884		14626					18456	1716,2	
tr|A0A100IH20|A0A100IH20_9MYCO	EsaT-6 like protein	27528							9170,3		
sp|B2HSL2|EFG_MYCMM	Elongation factor G	25704		22380		12248	2176,1		2850,1		
sp|B2HN62|RUVA_MYCMM	Holliday junction ATP-dependent DNA helicase RuvA	25292	1451200	51039	1958200	45964	1984200	1041400	66564	1035500	995300
sp|B2HCV5|KAD_MYCMM	Adenylate kinase	24880	59793	18894	35861	11349	23372	16308	14880	20306	
tr|A0A117DTU2|A0A117DTU2_9MYCO	Ferritin	24628	11384		17758	12343		5562	6568,3		
tr|B2HPD5|B2HPD5_MYCMM	Enoyl-[acyl-carrier-protein] reductase [NADH]	24021	3780,4				3816,5			1348,4	
tr|K7ZRA5|K7ZRA5_MYCMR	Acyl-CoA:diacylglycerol acyltransferase	23246	66060	15247	15255	9323,6	26507	96837	32332	5997,5	
tr|B2HIB7|B2HIB7_MYCMM	30S ribosomal protein S6	22146	20457	21738	29412	14197	8478,7	16461	32829		
tr|B2HMN2|B2HMN2_MYCMM	Superoxide dismutase	20601	75445			29579	27153				
tr|A0A2Z5YK62|A0A2Z5YK62_MYCMR	Multifunctional fusion protein	20210		17294	9476,6				16192		
tr|A0A2Z5YAE1|A0A2Z5YAE1_MYCMR	30S ribosomal protein S4	17259		27314	25815	16384	1215,8		33412		
tr|B2HCX4|B2HCX4_MYCMM	DNA-directed RNA polymerase subunit alpha	16460	126580		132340	43163	23327	40857	31406		
sp|B2HSJ4|RPOC_MYCMM	DNA-directed RNA polymerase subunit beta	7841,9	13741	14977	23430	13304	9390,4	11483	14603	10571	
tr|B2HNG4|B2HNG4_MYCMM	ATP-dependent Clp protease proteolytic subunit	6043,3	44569	11856	38203			26279	21205		32558
tr|B2HQI6|B2HQI6_MYCMM	Alpha-1,4-glucan:maltose-1-phosphate maltosyltransferase		73553		12888		16461	77200		6892,5	176700
tr|A0A124BVK1|A0A124BVK1_9MYCO	D-amino-acid oxidase		40219				21020			56035	31849
tr|B2HHL7|B2HHL7_MYCMM	Glutamine synthetase		39683	33741	78699	39730	22332	40540	48769	31173	
sp|B2HSN8|RL16_MYCMM	50S ribosomal protein L16		24314	25474	103280	762,35	19611	34766		21053	
tr|A0A100I744|A0A100I744_9MYCO	Cell division protein		23438		11261		13229	39434		82879	143360
tr|B2HCV1|B2HCV1_MYCMM	DNA topoisomerase (ATP-hydrolyzing)				26646			154440	12097		
tr|A0A100I9H4|A0A100I9H4_9MYCO	Sensor kinase							18774			1455300
sp|B2HSN1|RL3_MYCMM	50S ribosomal protein L3						53808		96143		
