# Supplementary material for: In vitro and ex vivo proteomics of Mycobacterium marinum biofilms and the development of biofilm-binding synthetic nanobodies
Source: mSystems. 2023 May 15;8(3):e01073-22. doi: 10.1128/msystems.01073-22 (PMC10308901; doi:10.1128/msystems.01073-22)
Supplement: FIG S1 — Recombinant GroEL1 and GroEL2 production in E. coli as tag-free and AVI-tagged biotinylated form. (A) Final samples of GroEL1 protein in biotinylated and tag-free form were analysed via SDS-PAGE and Coomassie staining to verify purity. (B) The biotinylation of GroEL1 was verified by 1 h co-incubation at RT of the protein with tamavidin and SDS-PAGE (without boiling) to see the shift of the band upon the binding of tamavidin. The tag-free form was used as a negative control. (C) On the left, the tamavidin shift assay was performed with biotinylated and tag-free GroEL2 samples as in B. On the right, final samples of biotinylated and tag-free GroEL2 were analysed for purity. (D) A representative graph from the SEC carried out with a Superdex 200 column to verify monodispersity of the purified proteins. (E–H) The folding and thermal stability of GroEL1 and GroEL2 as biotinylated and tag-free forms were verified by nano-DSF in buffer corresponding to the screening conditions (25 mM Tris pH 8.0 and NaCl 150 mM). The inflection point temperatures are indicated with arrows. [file msystems.01073-22-s0006.pdf]

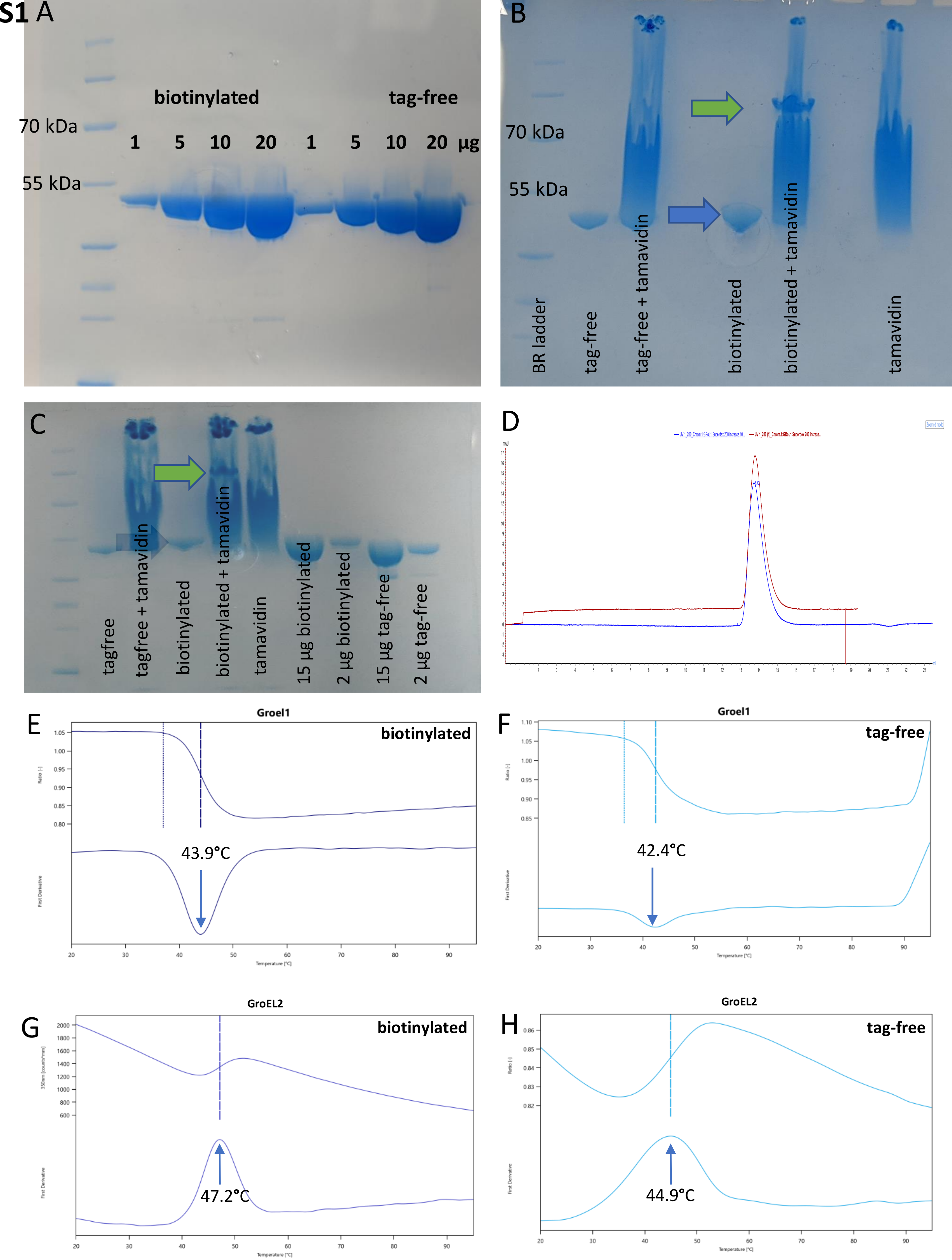
S1
A
biotinylated
tag-free
70 kDa
55 kDa
1 5 10 20 1 5 10 20 µg
B
70 kDa
55 kDa
BR ladder
tag-free
tag-free + tamavidin
biotinylated
biotinylated + tamavidin
tamavidin
C
tagfree
tagfree + tamavidin
biotinylated
biotinylated + tamavidin
tamavidin
15 µg biotinylated
2 µg biotinylated
15 µg tag-free
2 µg tag-free
D
E
Groel1
biotinylated
43.9°C
Ratio [-]
First Derivative
Temperature [°C]
F
Groel1
tag-free
42.4°C
Ratio [-]
First Derivative
Temperature [°C]
G
GroEL2
biotinylated
47.2°C
350nm [counts*mm]
First Derivative
Temperature [°C]
H
GroEL2
tag-free
44.9°C
Ratio [-]
First Derivative
Temperature [°C]

Supplementary figure S1. Recombinant GroEL1 and GroEL2 production in *E. coli* as tag-free and AVI-tagged biotinylated form. **(A)** Final samples of GroEL1 protein in biotinylated and tag-free form were analysed via SDS-PAGE and Coomassie staining to verify purity. **(B)** The biotinylation of GroEL1 was verified by 1 h co-incubation at RT of the protein with tamavidin and SDS-PAGE (without boiling) to see the shift of the band upon the binding of tamavidin. The tag-free form was used as a negative control. **(C)** On the left, the tamavidin shift assay was performed with biotinylated and tag-free GroEL2 samples as in B. On the right, final samples of biotinylated and tag-free GroEL2 were analysed for purity. **(D)** A representative graph from the SEC carried out with a Superdex 200 column to verify monodispersity of the purified proteins. **(E–H)** The folding and thermal stability of GroEL1 and GroEL2 as biotinylated and tag-free forms were verified by nano-DSF in buffer corresponding to the screening conditions (25 mM Tris pH 8.0 and NaCl 150 mM). The inflection point temperatures are indicated with arrows.
