# Supplementary figures and images for: In vitro and ex vivo proteomics of Mycobacterium marinum biofilms and the development of biofilm-binding synthetic nanobodies
Source: mSystems. 2023 May 15;8(3):e01073-22. doi: 10.1128/msystems.01073-22 (PMC10308901; doi:10.1128/msystems.01073-22)

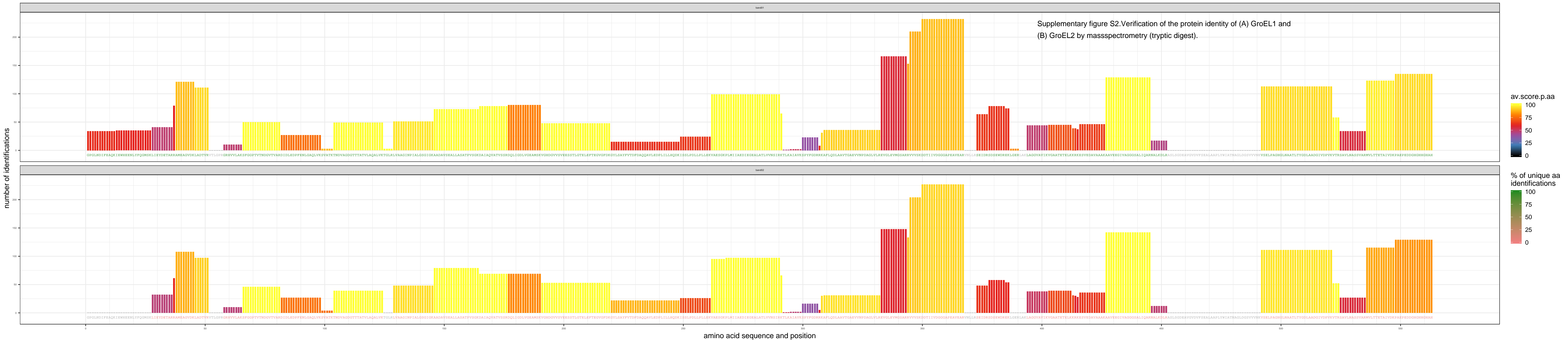

number of identifications

number of identifications

amino acid sequence and position

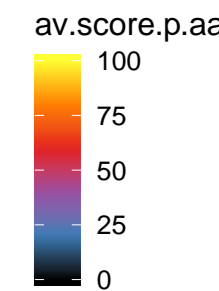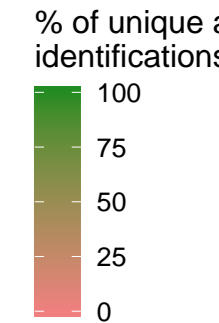

Supplement: FIG S2 — Verification of the protein identity of (A) GroEL1 and (B) GroEL2 by mass spectrometry (tryptic digest). [file msystems.01073-22-s0007.pdf]
