# Supplementary material for: In vitro and ex vivo proteomics of Mycobacterium marinum biofilms and the development of biofilm-binding synthetic nanobodies
Source: mSystems. 2023 May 15;8(3):e01073-22. doi: 10.1128/msystems.01073-22 (PMC10308901; doi:10.1128/msystems.01073-22)
Supplement: FIG S3 — Control experiments for sybody binding (A) As a negative control, 1 µg or 5 µg of His-Myc-tagged unspecific mannose binding protein (MBP) sybody was incubated with one-week-old Mmr biofilms. The sybodies bound to biofilm pellets were run onto SDS-page, and Western blots were stained with anti-Myc antibodies. No unspecific binding of this sybody was detected. On the first lane, 50 ng of MBP sybody was directly loaded onto the gel. (B-D) Two-week-old avirulent Mtb biofilms were stained with DAPI (shown in cyan) for DNA and with Myc-tagged sybodies against GroEL1 (C) or GroEL2 (D) + green-fluorescent anti-Myc antibodies (shown in yellow) or DAPI and anti-Myc antibody as a negative control (B). The stained biofilms were imaged with confocal microscopy. The scale bars are 10 µm. [file msystems.01073-22-s0008.pdf]

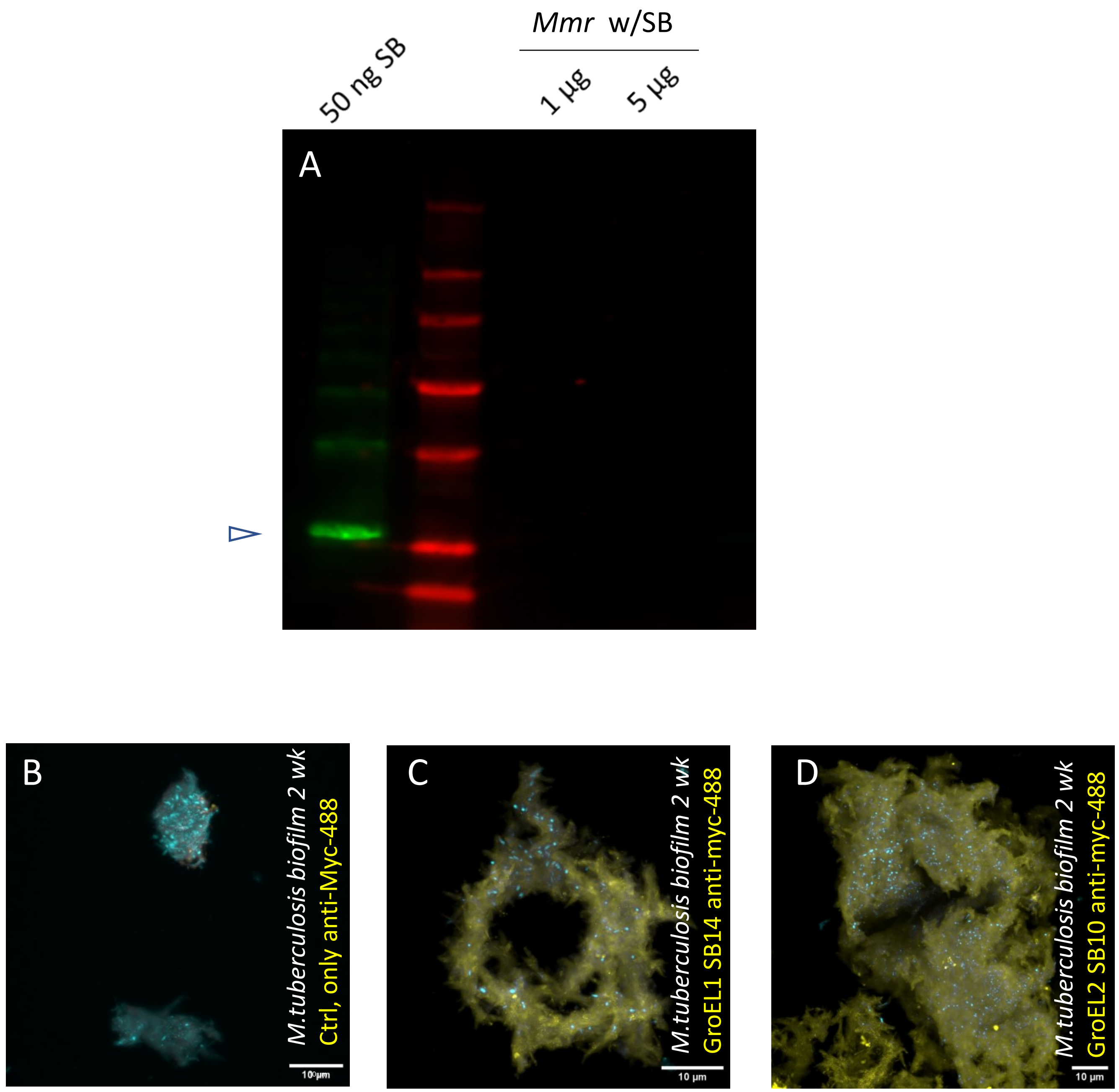

Supplementary figure S3. Control experiments for sybody binding **(A)** As a negative control, 1 µg or 5 µg of His-Myc-tagged unspecific mannose binding protein (MBP) sybody was incubated with one-week-old Mmr biofilms. The sybodies bound to biofilm pellets were run onto SDS-page, and western blots were stained with anti-Myc antibodies. No unspecific binding of this sybody was detected. On the first lane, 50 ng of MBP sybody was directly loaded onto the gel. **(B-D)** Two-week-old avirulent Mtb biofilms were stained with DAPI (shown in cyan) for DNA and with Myc-tagged sybodies against GroEL1 **(C)** or GroEL2 **(D)** + green-fluorescent anti-Myc antibodies (shown in yellow) or DAPI and anti-Myc antibody as a negative control **(B).** The stained biofilms were imaged with confocal microscopy. The scale bars are 10 µm.
